# Umpolung carbonyls enable direct allylation and olefination of carbohydrates

**DOI:** 10.1126/sciadv.abm6840

**Published:** 2022-03-09

**Authors:** Jian Kan, Zhangpei Chen, Zihang Qiu, Leiyang Lv, Chenchen Li, Chao-Jun Li

**Affiliations:** 1 Department of Chemistry and FRQNT Center for Green Chemistry and Catalysis, McGill University, 801 Sherbrooke Street West, Montreal, Quebec H3A 0B8, Canada.; 2 State Key Laboratory of Structural Chemistry, Fujian Institute of Research on the Structure of Matter, Chinese Academy of Sciences, Fuzhou, Fujian 350002, P. R. China.; 3 Center for Molecular Science and Engineering, College of Sciences, Northeastern University, Shenyang 110819, P. R. China.

## Abstract

Mother Nature has its own arts to build a vast number of carbohydrates; however, there is still a lack of tools for selective functionalization of native carbohydrates through C─C bond formation. Such a long-standing challenge for the synthetic community lies into the intrinsic problems related to the innate properties of carbohydrates, e.g., the ease to oligomerization or polymerization, the difficulty of chemoselectivity control in the presence of multiple hydroxyl groups, the great challenge to retain the multiple chiral centers during the transformation, etc. Here, by applying an umpolung strategy of carbohydrate carbonyls, we report a direct deoxygenative allylation and olefination of carbohydrates to tackle the abovementioned issues. The reaction is compatible with a wide range of natural carbohydrates, providing a direct synthetic method to use carbohydrates as multiple C-centered chiral synthons to achieve C─C bond cross-coupling reactions. Furthermore, the synthetic applicability is demonstrated by late-stage modifications of natural products and pharmaceutical derivatives.

## Teaser

Direct use of natural carbohydrates as multiple C-centered chiral synthons has been achieved via the umpolung carbonyl strategy.

## INTRODUCTION

**Fig. 1.**
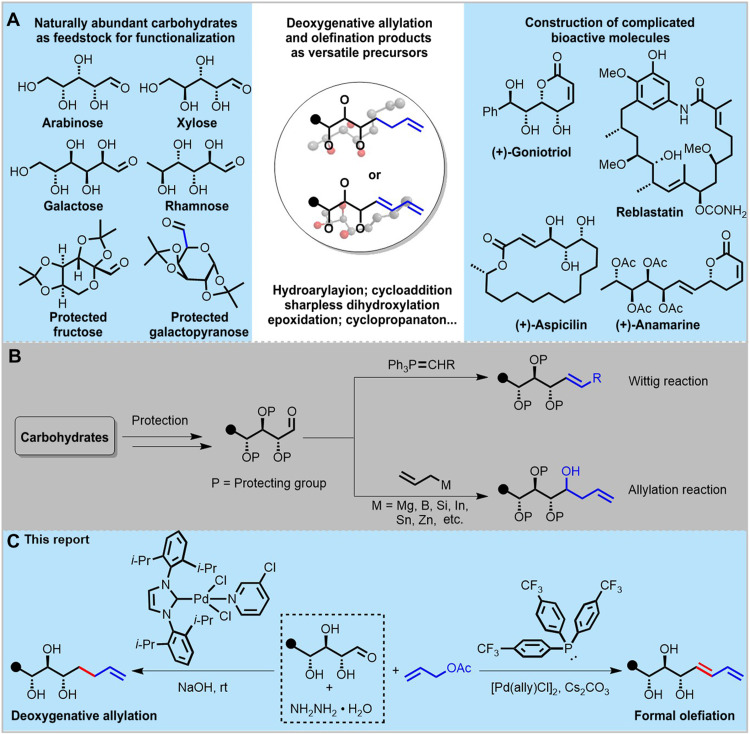
Prevalence of alkenyl carbohydrates and synthetic strategies toward them. (**A**) Great value of alkenyl carbohydrates in biomolecules synthesis and pharmaceutical research. (**B**) Known methods for alkenyl carbohydrates. (**C**) This report: Selective synthesis of alkene and 1,3-diene polyols from native sugars.

Direct and selective functionalization of native carbohydrates via C─C bond formation has been a longstanding challenge in synthetic chemistry. Being the most abundant biomass in nature and resonating across numerous disciplines, carbohydrate-based molecules have been found to have wide applications in chemical biology and medicinal chemistry as diagnostics, therapeutics, vaccines, drug delivery systems, and molecular receptors (*1*). Undoubtedly, chemical tools are indispensable for studies in synthetic carbohydrate-based chemistry, and the investigation of chemical transformations that allow selective functionalization of carbohydrates has been underway for more than a century (*2*). These transformations can be a valuable tool for elucidating the biological roles of natural sugars and may also lead to new discoveries (Fig. 1). However, as structurally complex scaffolds, carbohydrates pose unique synthetic challenges. One of the intrinsic challenges is that carbohydrates normally bear several hydroxyl (OH) groups, and each represents a potential site of chemical reactivity leading to the difficulty of chemoselectivity. Another obstacle refers to the retention of stereochemistry during reactions (*3*). Consequently, reliable methods toward the facile generation of structurally diverse carbohydrate-based molecules would be highly desirable in synthetic chemistry.

Alkenes are basic functionalities, highly versatile building blocks and prevalent units among natural products with a wide range of applications (*4*). In particular, the terminal alkenes and 1,3-dienes (*5*) not only play important roles in the synthesis of complicated structures but also are found in the skeleton of numerous value-added materials and pharmaceutical agents (Fig. 1A). Thus, the synthesis of these compounds is of great significance for the chemistry community. To date, there have been a few methods to incorporate carbon-carbon double bonds into carbohydrates, for example, the condensation-type reaction such as the Wittig reaction or its related transformations (Fig. 1B, top) (*6*) or allylation reaction to produce the homoallylic alcohols (Fig. 1B, bottom) (*7*, *8*). However, these transformations typically require the “preprotection” of hydroxyl groups of carbohydrates and are not capable to build 1,3-dienes within the carbohydrate backbones. Therefore, the development of a reliable and diverse synthetic method to directly assemble carbohydrates, without protecting the free hydroxyl groups, with both terminal alkenes and 1,3-dienes is highly desirable (Fig. 1C).

As a puissant tool for the construction of alkenes, the Tsuji-Trost allylation has been drawing intensive attention in modern synthetic chemistry (*9*, *10*). The recent increase in the exploration of new types of nucleophiles further extends the synthetic utility of this reaction (*11*–*14*). Recently, we have developed the umpolung carbonyls as novel nucleophiles to couple with alkynes (*15*), 1,3-dienes (*16*, *17*), allyl acetates (*18*), and functionalized cyclopropane derivatives (*19*, *20*) and afforded a series of functionalized alkenes. The mild reaction conditions and tolerance of various functional groups (even water) make this strategy a great opportunity for the direct functionalization of carbohydrates. Here, we wish to report the palladium-catalyzed deoxygenative allylation and olefination of carbohydrates with the readily available allyl acetates as the coupling partners enabled by umpolung carbonyls.

## RESULTS AND DISCUSSION

**Fig. 2.**
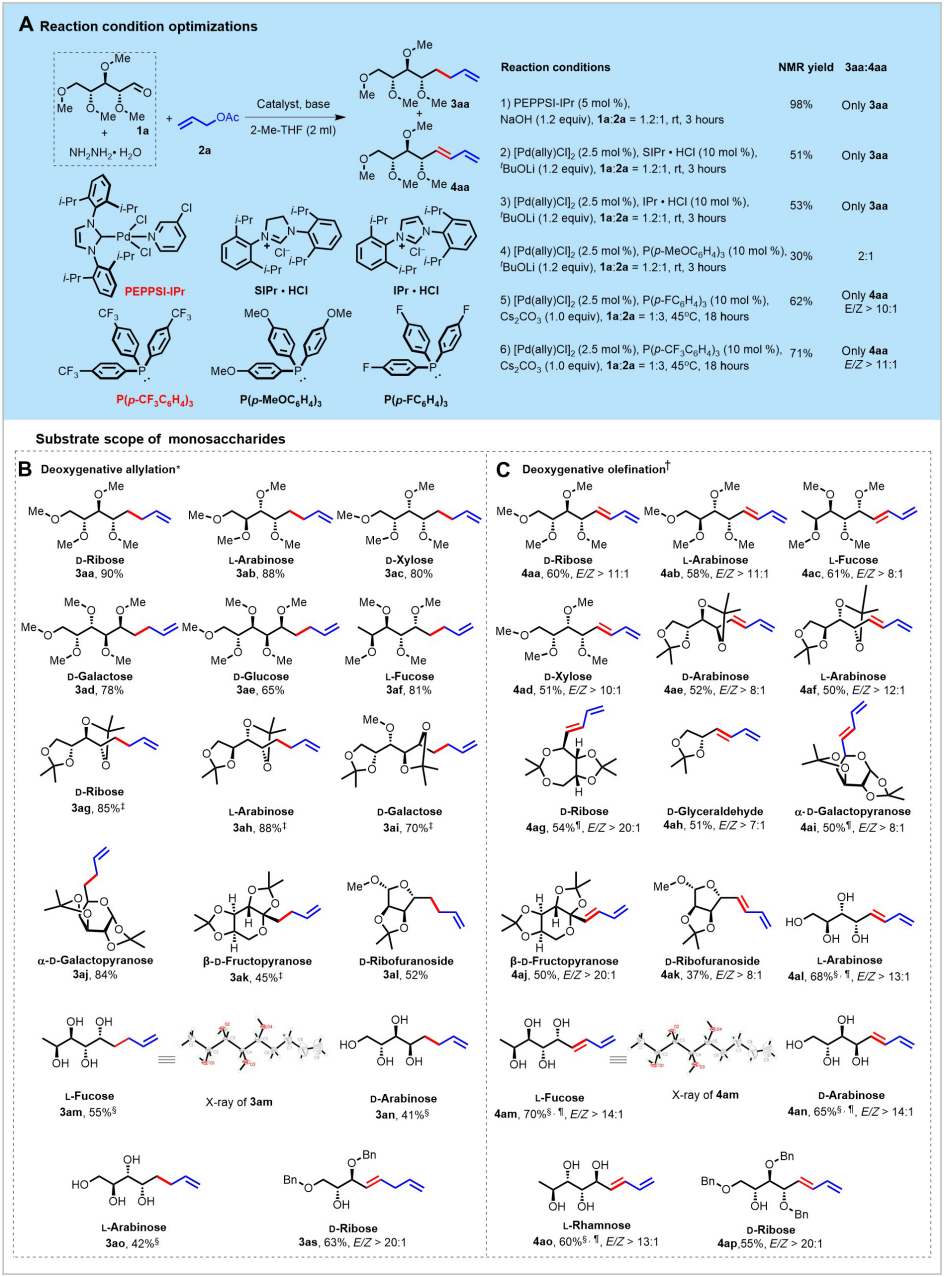
Reaction condition optimizations and substrate scope of monosaccharides. (**A**) Reaction optimization of deoxygenative allylation and olefination reactions. mol %, mole percent. (**B**) Scope of deoxygenative allylation of monosaccharides. (**C**) Scope of deoxygenative olefination of monosaccharides. Reaction conditions: *Hydrazone (0.24 mmol, 1.0 M generated in situ from monosaccharide and hydrazine monohydrate), allyl acetate (0.2 mmol), PEPPSI-IPr (5 mol %), NaOH (1.2 equiv), 2-Me-THF (2 ml), room temperature (rt), 3 hours, N_2_, and yields of isolated products. †Hydrazone (0.2 mmol), allyl acetate (0.6 mmol), [Pd(ally)Cl]_2_ (2.5 mol %), P(*p*-CF_3_C_6_H_4_)_3_ (10 mol %), Cs_2_CO_3_ (1.0 equiv), 2-Me-THF (2 ml), 45°C, 18 hours, N_2_, and yields of isolated products. ‡Run at 45°C. §NaOH (6.0 equiv), 3-Å Molecular Sieves (MS) (100 mg), 1,4-dioxane (2 ml), and 24 hours. ¶Run at 60°C.

As proof of concept, we initiated the exploration with protected carbohydrates. First, a solution of **1a** with 1.1 equiv of hydrazine hydrate in the corresponding solvent and allyl acetate **2a** was selected as the model substrates to optimize the reaction parameters including transition metal catalysts, ligands, ratio of reactants, and additives (detailed experimental data were provided in the Supplementary Materials). Experimental results demonstrated that palladium complexes with *N*-heterocyclic carbene (NHC) ligands are favorable for the generation of deoxygenative allylation product **3aa** (Fig. 2A). Notably, the product **3aa** could be obtained in 98% nuclear magnetic resonance (NMR) yield when the commercially available complex 1,3-bis(2,6-diisopropylphenyl)imidazol-2-ylidene](3-chloropyridyl)palladium(II) dichloride (PEPPSI-IPr) (*21*) was used as the catalyst (Fig. 2A, entry 1). Use of [Pd(ally)Cl]_2_/P(*p*-CF_3_C_6_H_4_)_3_ instead of PEPPSI-IPr gave the 1,3-diene product **4aa** as the formal olefination reaction in 71% yield with good *Z*:*E* selectivity (*E*:*Z* > 11:1; Fig. 2A, entry 6). Control experiments showed that no desired product (neither **3aa** nor **4aa**) was formed in the absence of palladium catalyst or base. With the optimized conditions identified, the substrate scope of monosaccharides in terms of both deoxygenative allylation and olefination reaction was investigated (Fig. 2). As shown in Fig. 2B, a series of methyl-protected monosaccharides could be transformed into the corresponding deoxygenative allylation products with good to excellent yields (**3aa**-**3af**). Similarly, cyclic ketal carbohydrates were also viable in this transformation, providing the desired products **3ag**-**3al** in good yields (45 to 85%). To our delight, the reaction occurred smoothly with free monosaccharides as well, and the corresponding products were afforded in moderate yields (**3an**-**3ao**). Subsequently, the substrate scope of diene was also examined, as summarized in Fig. 2C. In general, moderate to good yields and stereoselectivity were obtained with a broad range of both linear and cyclic monosaccharides. The stereocenter remained untouched, and the newly generated carbon-carbon double bond favored *E*-configuration. Notably, when subjecting the acetone protected d-ribose to this reaction, the product **4ag** was isolated in sole *E*-form. Moreover, the free monosaccharides turned out to be suitable substrates in this transformation, furnishing the 1,3-diene products with good yields and *Z*:*E* selectivities (**4al**-**4ao**). The structures of **3am** and **4am** were well established by x-ray crystallographic analysis (*22*). The absolute configuration of compound **4am** was defined as (2*S*,3*R*,4*S*,5*R*,*E*) using Cu Kα radiation with Hooft parameters of −0.07(6) (*23*).

**Fig. 3.**
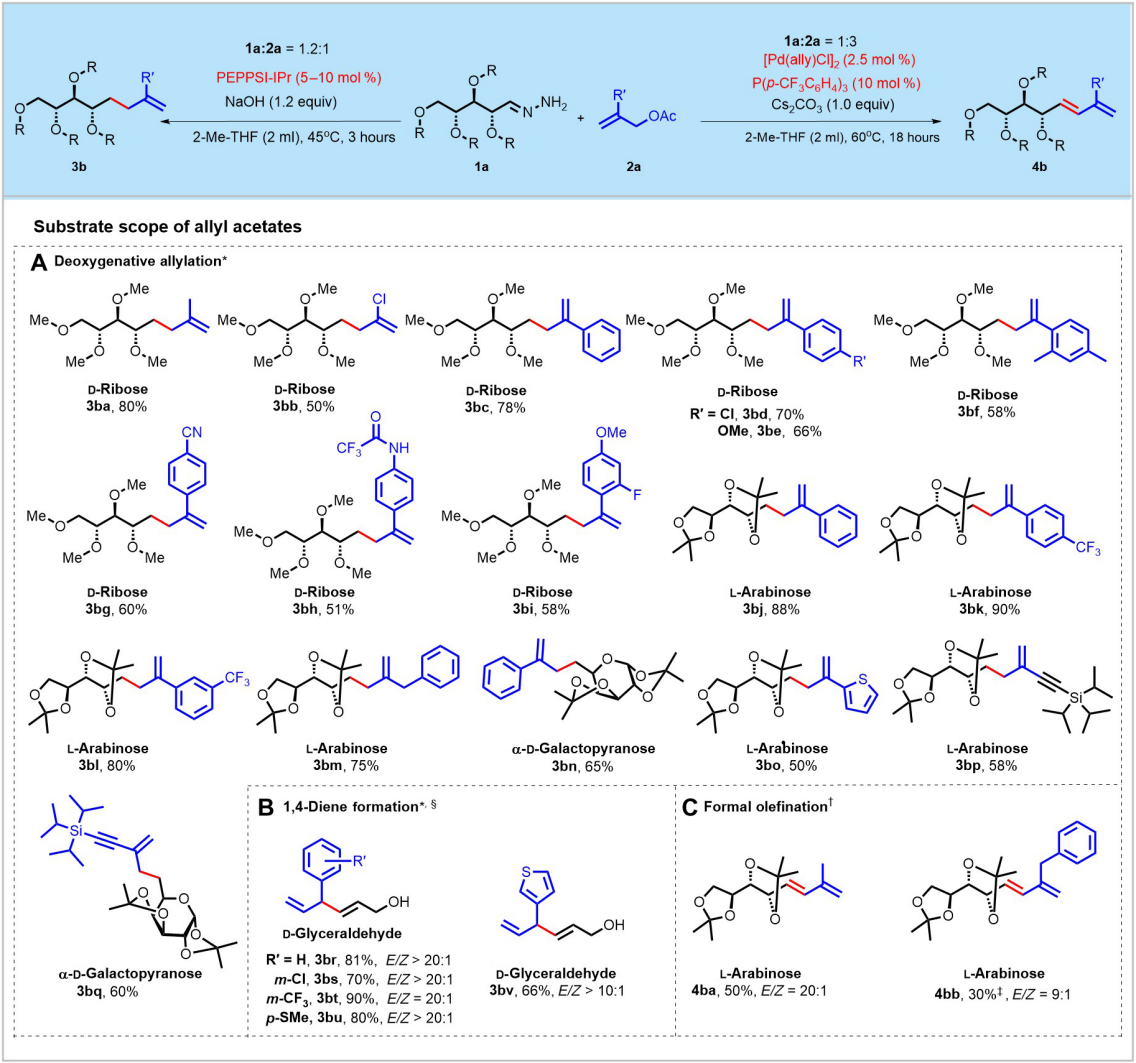
Substrate scope of allyl acetates. (**A**) Deoxygenative allylation. (**B**) 1,4-Diene formation. (**C**) Formal olefination. Reaction conditions: *Hydrazone (0.24 mmol), 1.0 M generated in situ from monosaccharide and hydrazine monohydrate, allyl acetate (0.2 mmol), PEPPSI-IPr (5 mol %), NaOH (1.2 equiv), 2-Me-THF (2 ml), 45°C, 3 hours, N_2_, and yields of isolated products. †Hydrazone (0.2 mmol), allyl acetate (0.6 mmol), [Pd(ally)Cl]_2_ (2.5 mol %), P(*p*-CF_3_C_6_H_4_)_3_ (10 mol %), NaOH (2.0 equiv), 2-Me-THF (2 ml), 60°C, 18 hours, N_2_, and yields of isolated products. ‡*^t^*BuOLi (2.0 equiv). §Run at rt and 2 hours.

Next, the generality of allyl acetates was further investigated, and the results were summarized in Fig. 3. The deoxygenative allylation of protected monosaccharides with 2-methylallyl acetate or 2-chloroallyl acetate was feasible, providing the terminal alkenes in good yields (**3ba**-**3bb**). 2-Aryl–substituted allyl acetates could also smoothly be transformed into the deoxygenative allylation products (**3bc**-**3bo**) with both linear and cyclic monosaccharide derivatives. The functional groups, including chloro (**3bd**), methoxyl (**3be**), nitrile (**3bg**), amide (**3bh**), fluoro (**3bi**), and trifluoromethyl (**3bk**, **3bl**), regardless of the ortho-, meta-, para-, or multiple substitutions, were all tolerated to generate the corresponding products in modest to excellent yields under the standard conditions. Note that the 3-thienyl allyl acetate could also be smoothly transformed into the desired product **3bo** in 50% yield. The reaction of alkynyl allyl acetate was also viable with this protocol, affording **3bp** and **3bq** in 58 and 60% yields, respectively. Unexpectedly, the reaction of d-glyceraldehyde with 2-aryl–substituted allyl acetates under the optimal conditions generated a variety of 1,4-dien-1-ols (**3br**-**3bv**) in high yields and *Z*:*E* selectivity. For the formal olefination reaction, both 2-alkylallyl acetate and 2-arylallyl acetate were tolerated, giving the 1,3-diene products **4ba** and **4bb** in acceptable yields and with high *Z*:*E* selectivity.

**Fig. 4.**
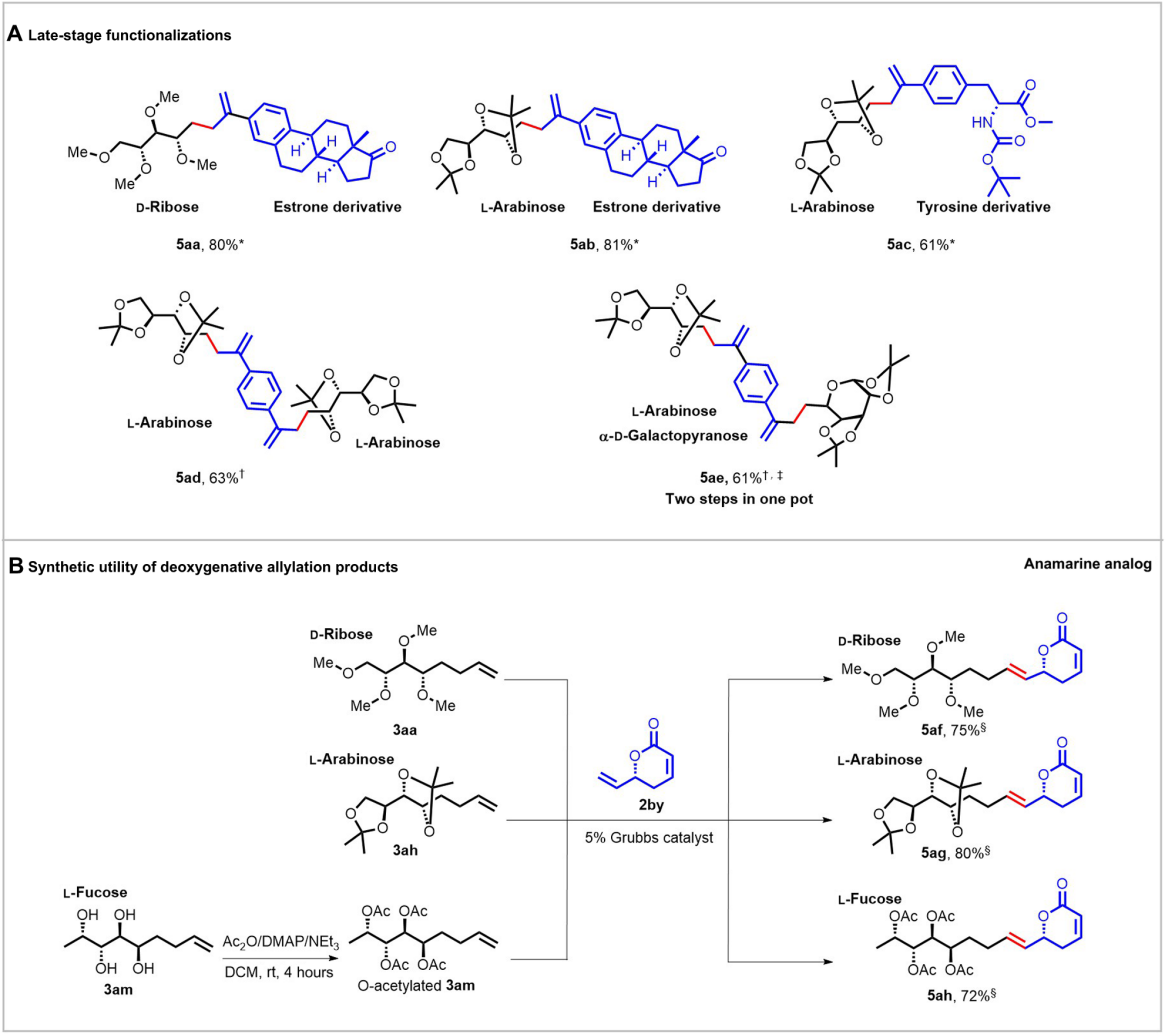
Synthetic applications. (**A**) Late-stage functionalizations. (**B**) Postsynthetic modification of deoxygenative allylation products. Reaction conditions: *Hydrazone (0.24 mmol, 1.0 M generated in situ from monosaccharide and hydrazine monohydrate), allyl acetate (0.2 mmol), PEPPSI-IPr (5 mol %), NaOH (1.2 equiv), 2-Me-THF (2 ml), 45°C, 3 hours, N_2_, and yields of isolated products. †Hydrazone (0.24 mmol, 1 M generated in situ from monosaccharide and hydrazine monohydrate), allyl acetate (0.1 mmol), PEPPSI-IPr (10 mmol %), NaOH (2.4 equiv), 2-Me-THF (1 ml), 45°C, 6 hours, N_2_, and yields of isolated products. ‡For details, see the Supplementary Materials. §The second-generation Grubbs catalyst (5 mol %), **3aa** or **3ah** or **3am** (0.1 mmol), (*R*)-6-vinyl-5,6-dihydro-2*H*-pyran-2-one (0.2 mmol), DCM (20 ml), 40°C, and 15 hours.

To further exploit the practicability of this method, we examined its application in the late-stage modification of natural products and pharmaceutical derivatives. As shown in Fig. 4, the deoxygenative allylation reaction with allyl acetate derivatives bearing an estrone moiety proceeded smoothly under the optimized conditions, leaving the ketone unit of estrone untouched, and provided the desired products **5aa** and **5ab** in good yields. The tyrosine derivative was also tested as a viable candidate, delivering the target product **5ac** in 51% yield. Furthermore, for substrates with two allyl acetate moieties, the sequential double deoxygenative allylation processes occurred to afford the products **5ad** and **5ae** in 63 and 61% yields, respectively. In addition, the synthesized deoxygenative allylation products could be readily transformed to some useful molecules. For example, the products **3aa**, **3ah**, and **3am** could undergo the olefin metathesis reactions with the second-generation Grubbs catalysts to efficiently generate the corresponding products **5af**-**5ah** as anticarcinogen anamarine analogs (*24*, *25*). These successful applications in the modification of complex molecules exemplified the unique chemoselectivity and practicability of this protocol.

**Fig. 5.**
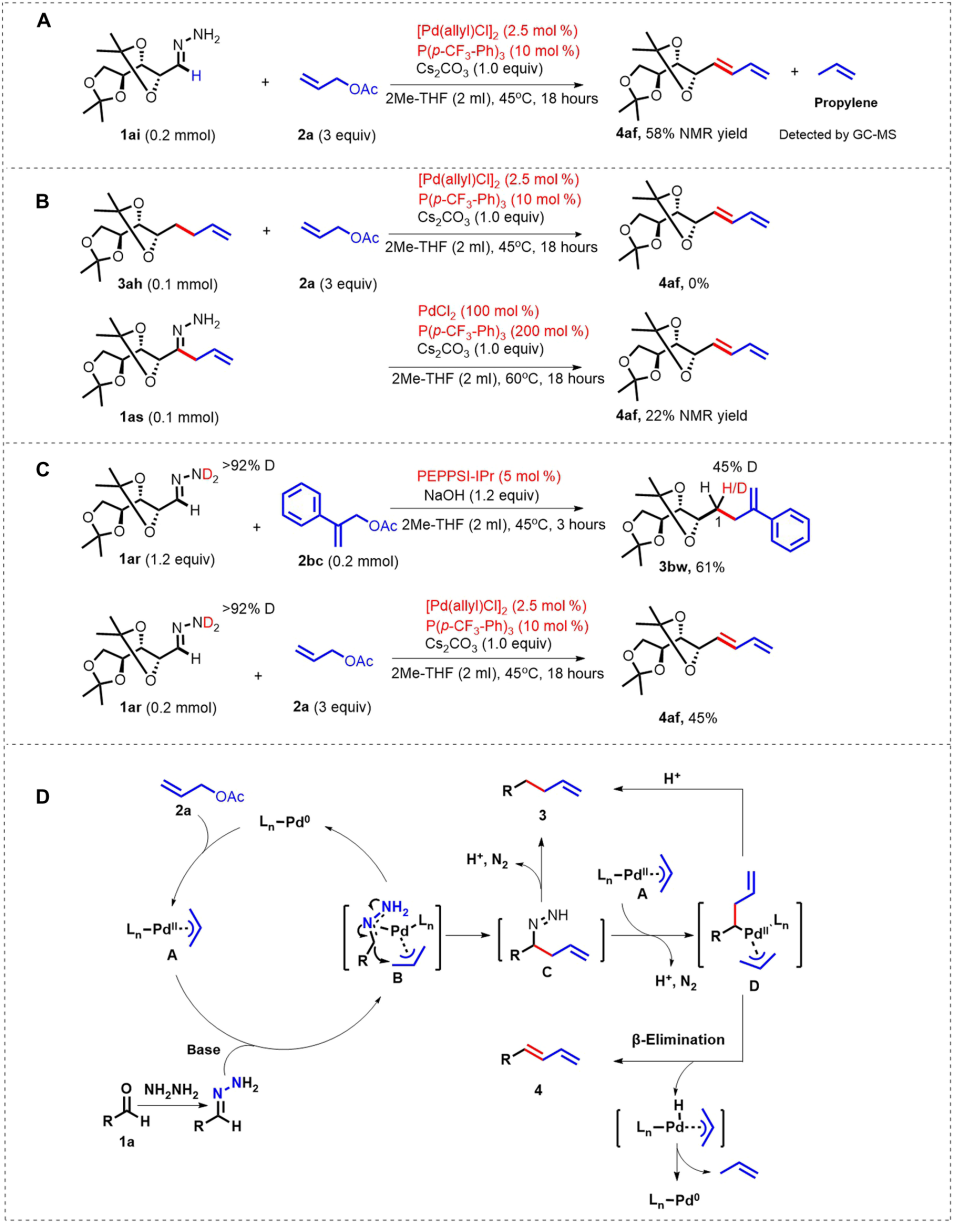
Mechanistic studies and proposed mechanism. (**A**) Detection of propylene experiment. (**B**) Inspection of possible intermediate. (**C**) Deuterium-labeling studies. (**D**) Proposed reaction pathways for the deoxygenative allylation and olefination reactions.

To gain preliminary insights into the mechanism of deoxygenative allylation and olefination reaction, several experiments were subsequently carried out (for details, see the Supplementary Materials). First, several *N*-tosylhydrazones based on the monosaccharide moiety were synthesized and subjected to the optimized reaction conditions (*26*). However, no desired products were generated, indicating that the transformation did not occur through the carbene process (*27*). Notably, propylene could be detected by gas chromatography–mass spectrometry (GC-MS), indicating that a β-elimination process might be involved in the diene formation (Fig. 5A). Exposure of deoxygenative allylation product **3ah** to the formal olefination reaction conditions failed to give the diene product, which ruled out the possibility of diene products being generated from the dehydrogenation of the allylation products (Fig. 5B, top). Moreover, the allyl hydrazone derivative **1as** could be converted into the diene product **4af** under the olefination reaction conditions (Fig. 5B, bottom). Besides, when the deuterated hydrazone **1ar-d**^**2**^ was tested under two reaction conditions accordingly, the deuterated allylation product **3bw-d**^**1**^ with deuterium incorporation at the C1 position (45% D) was afforded, while the completely deuterium-free diene product **4af** was obtained (Fig. 5C). Collectively, these results indicated that the 1,3-diene might be generated from the allyl azo-species **C** (Fig. 5D) rather than the dehydrogenation of allylation product.

On the basis of the above results and previous studies (*15*, *18*, *19*), a plausible mechanism is proposed in Fig. 5D. Initially, Pd(0) was generated from the precatalyst upon reduction possibly by extra hydrazine. Then, π-allylpalladium complex **A** was generated through oxidative addition of allyl acetate. Base-mediated interaction of complex **A** with hydrazone formed intermediate **B**, which rearranged to give the allyl azo-species **C**. Decomposition of **C** with N_2_ extrusion released the product **3** or **4** under different conditions and completed the catalytic cycle. The formation of diene **4** could be explained via a possible β-elimination process with the release of propylene. The product **3** may also have resulted from the denitrogenation and protolysis dissociation of the metal catalyst.

In summary, we have demonstrated an unprecedented Pd-catalyzed selective deoxygenative allylation and olefination of monosaccharides with allyl acetates in their native states or protected forms. These transformations proceed with high chemoselectivity to deliver various terminal alkenes and 1,3-dienes. The reaction is compatible with a wide range of functional groups, including ketone, nitrile, amide, and halide. Furthermore, late-stage modifications of natural products and pharmaceutical derivatives exemplify its synthetic potentials. This study provides enlightenment on the elaborately enabling complicated carbohydrates to construct different functional products via metal-catalyzed C─C bond formation reactions in chirality retention of carbohydrate backbone.

## MATERIALS AND METHODS

All reactants or reagents were obtained from commercial sources and used without further purification unless otherwise specified. All reactions were performed in flame-dried microwave reaction vials sealed with polytetrafluoroethylene-faced silicone septa and aluminum cap, under an atmosphere of nitrogen. All purification procedures were carried out with reagent-grade solvents. Synthetic methods of starting materials are described in the Supplementary Materials.

Flash column chromatography was performed with Biotage Isolera equipped with Biotage SNAP Cartridge KP-Sil columns. NMR spectra were recorded with Bruker Avance III (^1^H, 400 MHz; ^13^C, 101 MHz; and ^19^FNMR, 376 MHz) spectrometer or Bruker AV500 spectrometer equipped with a 60-position SampleXpress sample changer (^1^H, 500 MHz; ^13^C, 125 MHz; and ^19^FNMR, 470 MHz). High-resolution mass spectrometry was conducted by using atmospheric pressure chemical ionization or electrospraying ionization and was performed on a Thermo Scientific Exactive Orbitrap. Optical rotations were measured on a JASCO DIP-140 digital polarimeter or SGW-1 automatic polarimeter.

### General procedure for the deoxygenative allylation of monosaccharides

PEPPSI-IPr catalyst [6.8 mg, 5 mole percent (mol %)], allyl acetate **2** (0.2 mmol), and 2-methyltetrahydrofuran (2-Me-THF) (0.5 ml) were added into a dried microwave vial (10 ml) equipped with a stir bar in the glove box. The reaction mixture was stirred at room temperature for 5 min before hydrazone solution **1** (0.24 mmol, 240 μl, 1 M), NaOH (9.6 mg, 0.24 mmol), and 2-Me-THF (1.26 ml) were added. The reaction tube was sealed and moved out of the glove box. The resulting mixture was stirred at room temperature for 3 hours. After the completion of the reaction, the reaction solution was filtered through a short Celite pad and washed with diethyl ether (60 ml). The combined solution was removed under vacuum, and the residue was purified by flash column chromatography to give the desired product **3**.

### General procedure for the deoxygenative olefination of monosaccharides

[Pd(allyl)Cl]_2_ (1.9 mg, 2.5 mol %), tris(4-trifluoromethylphenyl)phosphine (9.4 mg, 10 mol %), allyl acetate **2** (0.6 mmol), and 2-Me-THF (0.5 ml) were added into a dried microwave vial (10 ml) equipped with a stir bar in the glove box. The reaction mixture was stirred at room temperature for 5 min before hydrazone solution **1** (0.20 mmol, 200 μl, 1 M), Cs_2_CO_3_ (65.2 mg, 0.2 mmol), and 2-Me-THF (1.3 ml) were added. The reaction tube was sealed and moved out of the glove box. The resulting mixture was stirred at 45°C for 18 hours. After the completion of the reaction, the reaction solution was filtered through a short Celite pad and washed with diethyl ether (60 ml). The combined solution was concentrated under vacuum, and the residue was purified by flash column chromatography on silica gel to give the desired product **4** or **5aa-5ae**.

### General procedure for the synthetic utility of deoxygenative allylation products

Grubbs catalyst (the second generation, 4.3 mg, 0.005 mmol) was added to a stirred solution of **2by** (24.8 mg, 0.2 mmol) and **3** (0.1 mmol) in Dichloromethane (DCM) (20 ml). The resulting solution was heated and stirred at 40°C for 15 hours. After the completion of the reaction, the reaction solution was filtered through a short Celite pad and washed with EtOAc (60 ml). The combined solution was concentrated under vacuum, and the residue was purified by flash column chromatography (hexane/EtOAc, 1:1) to give the desired product **5af-5ah**.

## Supplementary Materials

### This PDF file includes:

Supplementary Text Figs. S1 and S2 Tables S1 to S3 Spectral Data References
