# Supplementary material for: Umpolung carbonyls enable direct allylation and olefination of carbohydrates
Source: Sci Adv. 2022 Mar 9;8(10):eabm6840. doi: 10.1126/sciadv.abm6840 (PMC8906572; doi:10.1126/sciadv.abm6840)
Supplement: Supplementary file 1 — Supplementary Text Figs. S1 and S2 Tables S1 to S3 Spectral Data References [file sciadv.abm6840_sm.pdf]

Science Advances
AAAS

# Supplementary Materials for

## Umpolung carbonyls enable direct allylation and olefination of carbohydrates

Jian Kan, Zhangpei Chen, Zihang Qiu, Leiyang Lv, Chenchen Li, Chao-Jun Li*

*Corresponding author. Email: cj.li@mcgill.ca

Published 9 March 2022, *Sci. Adv.* **8**, eabm6840 (2022)
DOI: 10.1126/sciadv.abm6840

**This PDF file includes:**

Supplementary Text
Figs. S1 and S2
Tables S1 to S3
Spectral Data
References

**Supplementary Text**

**I. General experimental information ........ S2**

**II. Optimization of the reaction conditions ........ S3**

**III. General procedure for the starting material ........ S4**

**IV Exprimental procedures for deoxygenative allylation or olefination ........ S22**

**V. Characterization data for the products ........ S23**

**VI. Preliminary mechanistic studies ........ S60**

**VII. Spectral data for representative compounds ........ S68**

**VIII. 2D NOESY spectra ........ S175**

**IV. X-ray crystallographic data ........ S181**

***I. General experimental information***

***Solvents and chemicals:*** Anhydrous 2-methyltetrahydrofuran (2-Me-THF) was purchased from Sigma Aldrich Inc and stored in the glovebox. Tetrahydrofuran (THF) was distilled over sodium/benzophenone under nitrogen and stored under nitrogen. Other solvents used in this work were obtained from commercial sources and were used without further purification, such as dichloromethane ($CH_2Cl_2$) (Fisher, ACS grade), hexane (Fisher, ACS grade), ethyl acetate (EtOAc) (Fisher, ACS grade), methanol (ACS grade) and so on. Most of the reagents used in this work were purchased from Sigma-Aldrich, Alfa Aesar Chemical and Combi-Blocks companies, and were used without further purification unless otherwise specified.

***Spectroscopies (NMR, HRMS):*** Nuclear magnetic resonance ($^1$H, $^{13}$C, and $^{19}$FNMR) spectra were recorded with Bruker Avance III (400 MHz, 101 MHz and 376 MHz, respectively) spectrometer, or Bruker AV500 spectrometer equipped with a 60-position Sample Xpress sample changer ($^1$H, 500 MHz; $^{13}$C, 125 MHz; $^{19}$F, 470 MHz). Chemical shifts were reported in units (ppm) by assigning TMS resonance in the $^1$H NMR spectra as 0.00 ppm (chloroform, 7.26 ppm; deuterium oxide, 4.79 ppm; methanol-$d_4$, 3.31 ppm). Spectra data were reported as follows: chemical shift, multiplicity (s = singlet, d = doublet, t = triplet, q = quarter, m = multiplet, br = broad), coupling ($J$) constant and integration. High resolution mass spectra (HRMS) were conducted by using atmospheric pressure chemical ionization (APCI) or electro-spraying ionization (ESI) and performed by McGill University on a Thermo-Scientific Exactive Orbitrap. Protonated/deprotonated molecular ions $(M\pm H)^+$ or sodium adducts $(M+Na)^+$ were used for empirical formula confirmation.

***Compound name abbreviations:*** 1,3-Bis(2,4,6-trimethylphenyl)imidazolinium chloride (**SIMes•HCl**), 1,3-bis-(2,6-diisopropylphenyl)imidazolium chloride (**IPr•HCl**), 1,3-bis-(2,6-diisopropylphenyl)imidazolinium chloride (**SIPr•HCl**), 1,3-dicyclohexylimidazolium chloride (**ICy•HCl**), 1,1′-ferrocenediyl-bis(diphenylphosphine) (**dppf**), tri(*p*-tolyl)phosphine (**P(*p*-tol)₃**), tris(4-methoxyphenyl)phosphine (**P(*p*-MeOC₆H₄)₃**), tris(4-trifluoromethylphenyl)phosphine (**P(*p*-CF₃C₆H₄)₃**), tris(4-fluorophenyl)phosphine (**P(*p*-FC₆H₄)₃**), tri(2-furyl)phosphine **(P(2-Furyl)₃**), 2-dicyclohexylphosphino-2′,4′,6′-triisopropylbiphenyl (**Xphos**).

## *II. Optimization of the reaction conditions*

**Table S1. Optimization of the reaction conditions***

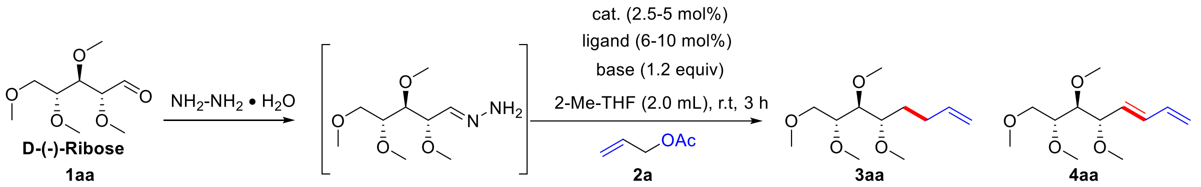


| Entry | **1a**:**2a** | Cat. | Ligand | Base (equiv) | Yield [%] † | **3aa**:**4aa** |
|---|---|---|---|---|---|---|
| 1 | 1.2:1 | $[Pd(ally)Cl]_2$ | $L_1$ or $L_2$ or $L_3$ | $^tBuOLi$ (1.2) | 48-51 | >20:1 |
| 2 | 1.2:1 | $[Pd(ally)Cl]_2$ | $L_4$ | $^tBuOLi$ (1.2) | 53 | >20:1 |
| 3 | 1.2:1 | PEPPSI-IPr | - | $^tBuOLi$ (1.2) | 55 | >20:1 |
| 4 | 1.2:1 | $[Pd(ally)Cl]_2$ | $L_5$ or $L_6$ or $L_7$ or $PPh_3$ | $^tBuOLi$ (1.2) | 18-30 | 1:1-2:1 |
| 5 | 1.2:1 | PEPPSI-IPr | - | DBU (1.2) | 0 | - |
| 6 | 1.2:1 | PEPPSI-IPr | - | $K_3PO_4$ (0.4) | 0 | - |
| 7 | 1.2:1 | PEPPSI-IPr | - | $Cs_2CO_3$ (0.6) | 13 | 12:1 |
| **8** | **1.2:1** | **PEPPSI-IPr** | **-** | **NaOH (1.2)** | **98(90)** # | **>20:1** |
| 9 | 1.2:1 | PEPPSI-IPr | - | KOH (1.2) | 94 | 14:1 |
| 10‡ | 1.2:1 | PEPPSI-IPr | - | NaOH (1.2) | 97 | 18:1 |
| 11§ | 1.2:1 | PEPPSI-IPr | - | NaOH (1.2) | 66 | 3.3:1 |

| | | | | | | |
|---|---|---|---|---|---|---|
| 12[§] | 1.2:1 | $[Pd(ally)Cl]_2$ | $L_8$ | $Cs_2CO_3$ (1.0) | 24 | 1: >20 |
| **13[‡, ¶]** | **1:3** | **$[Pd(ally)Cl]_2$** | **$L_8$** | **$Cs_2CO_3$ (1.0)** | **71(60)[#]** | **1: >20** |
| 14[‡, ¶] | 1:3 | $[Pd(ally)Cl]_2$ | $L_9$ | $Cs_2CO_3$ (1.0) | 62 | 1: >20 |
| 15[‡, ¶] | 1:3 | $[Pd(ally)Cl]_2$ | $L_6$ or $L_7$ or $L_{10}$ or $L_{11}$ | $Cs_2CO_3$ (1.0) | <25 | 1.1-1:5 |

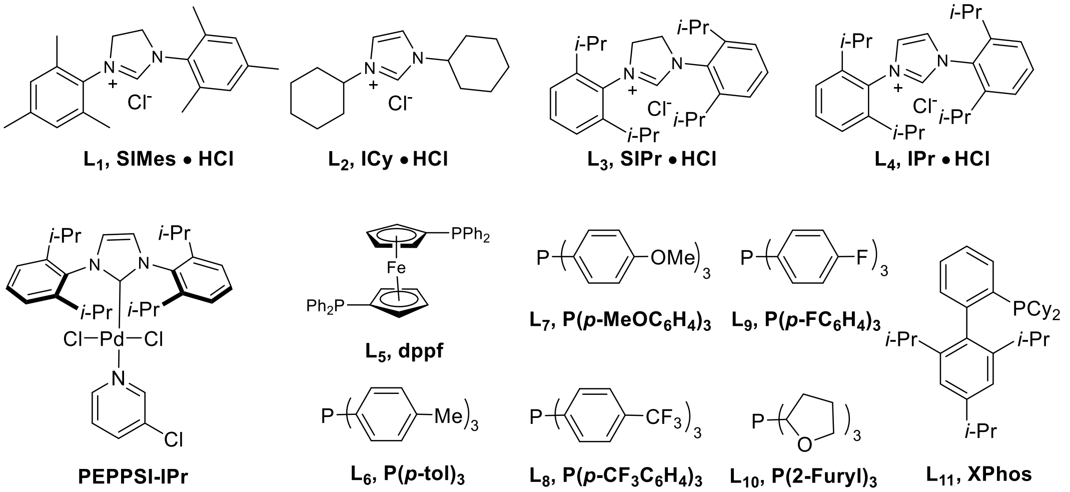


[*]Standard reaction conditions: hydrazone (1M generated in situ from methyl protected D-Ribose and hydrazine monohydrate), allyl acetate (0.2 mmol for allylic alkylation and 0.6 mmol for entries 13-14), $[Pd(ally)Cl]_2$ (2.5 mmol%), ligand (6 mmol% for NHC, 10 mmol% for $L_6$-$L_{11}$), base (1.2 equiv; extra 6 mmol% $^t$BuONa was added when NHC ligand was used), 2-Me-THF (2.0 mL), rt, 3 h, $N_2$. [†]Yields were determined by $^1$H NMR using 1,3,5-trimethoxybenzene as an internal standard. [‡] 45 $^o$C. [§]60 $^o$C. [¶]18 h. [#]Isolated yield.

### *III. General procedure for the starting material*

### 1. General procedure for the synthesis of protected carbohydrates.

Protected monosaccharides were prepared with minor alterations according to the previous literature (*28*).

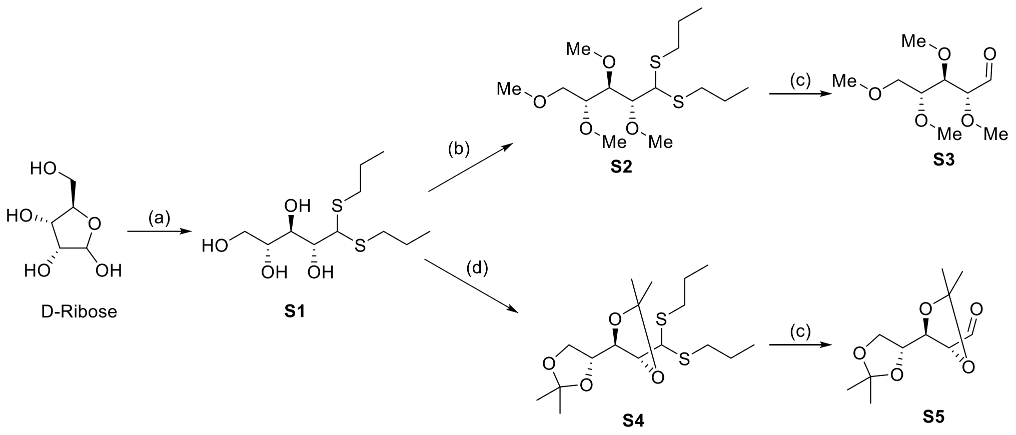


**1) Procedure A:**

(a) 1-Propanethiol (4.9 mL, 54.0 mmol) was added to a solution of D-Ribose (4.0 g, 26.6 mmol) in concentrated hydrochloric acid (4.0 mL), and the reaction mixture was stirred for 10 min. Then the mixture was cooled to 0 $^{o}$C and stirred for an additional 2 hrs at this temperature. Cold water (20.0 mL) was added, and the mixture was stirred for another 1 hr. The white solid **S1** was collected by filtration and washed with cold water (30 mL) and hexane (50 mL).

(b) **S1** (3.0 g, 14.4 mmol) dissolved in 40.0 mL of DMSO was charged into a 250 mL round bottom flask. Then 6.8 mL 50% aqueous NaOH was added. At this point, MeI (4.7 mL, 75.0 mmol) was added dropwise for 20 min. The resulting mixture was allowed to stir for 8 hrs before it was diluted with 80 mL $H_2O$ and extracted with diethyl ether (3 x 100 mL). After the solvent was removed under reduced pressure, the crude residue was purified by flash chromatography to yield the compound **S2**.

**(c)** **S2** (6.0 mmol) was dissolved in an ice-cooled mixture of acetone/water (66 mL, 10:1). $NaHCO_3$ (2.3 g, 27.0 mmol) and $I_2$ (3.4 g, 13.0 mmol) were successively added, and the resulting mixture was stirred for 16 hrs at room temperature. The reaction was quenched with aqueous $Na_2S_2O_3$ (30%, 100 mL) and extracted with diethyl ether (3 x 100 mL). The organic layer was dried and concentrated under reduced pressure and purified via flash chromatography using ethyl acetate/hexane (1:4) as eluent to give **S3** or **S5** as colorless oil.

**2) Procedure B:**

(d) To a solution of **S1** (11.0 mmol), *p*-TsOH•$H_2O$ (210.0 mg, 1.1 mmol) in acetone (60 mL), dimethoxypropane (27.0 mL, 220.0 mmol) was added dropwise for 10 min. The mixture

was then stirred for 2 hr at ambient temperature before a saturated $Na_2CO_3$ solution (20 mL) was added. Then the mixture was extracted with diethyl ether (3 x 100 mL). The organic layers were washed with brine and dried over anhydrous $MgSO_4$. After the solvent was removed under reduced pressure, the crude residue was purified by flash chromatography to yield the compound **S4**.

(c) **S4** (6.0 mmol) was dissolved in an ice-cooled mixture of acetone/water (66 mL, 10:1). $NaHCO_3$ (2.3 g, 27.0 mmol) and $I_2$ (3.4 g, 13.0 mmol) were successively added, and the resulting mixture was stirred for 16 hrs at room temperature. The reaction was quenched with aqueous $Na_2S_2O_3$ (30%, 100 mL) and extracted with diethyl ether (3 x 100 mL). The organic layer was dried and concentrated under reduced pressure and purified via flash chromatography using ethyl acetate/hexane (1:4) as eluent to give **S5** as a colorless oil.

*2,3,4,5-Tetra-O-methyl-D-ribose (**1aa**):*

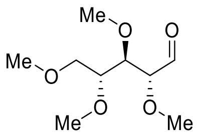


Following the **procedure A**, the title compound was prepared using D-Ribose as starting material, and then purified by flash chromatography with hexanes/ethyl acetate (4:1) as the eluent. **1aa:** colorless oil; **$^1$H NMR** (500 MHz, chloroform-*d*) δ 9.54 (s, 1H), 3.87 (d, *J* = 2.1 Hz, 1H), 3.67 – 3.61 (m, 2H), 3.56 (s, 3H), 3.50 – 3.46 (m, 1H), 3.47 – 3.45 (m, 1H), 3.44 (s, 3H), 3.39 (s, 3H), 3.38 (s, 3H). **$^{13}$C NMR** (126 MHz, chloroform-*d*) δ 200.6, 84.7, 81.7, 78.0, 70.4, 59.6, 59.3, 58.5, 57.9.

*2,3,4,5-Tetra-O-methyl-L-arabinose (**1ab**):*

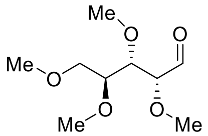


Following the **procedure A**, the title compound was prepared using L-Arabinose as starting material, and then purified by flash chromatography with hexanes/ethyl acetate (4:1) as the eluent. **1ab:** colorless oil; **$^1$H NMR** (500 MHz, chloroform-*d*) δ 9.80 (d, *J* = 1.5 Hz, 1H), 3.83 (dd, *J* = 3.1, 1.5 Hz, 1H), 3.76 (dd, *J* = 8.5, 3.0 Hz, 1H), 3.69 (dd, *J* = 10.6, 2.5 Hz, 1H), 3.52 (s, 3H), 3.49 (dd, *J* = 10.6, 3.4 Hz, 1H), 3.43 – 3.40 (m, 1H), 3.40 (s, 3H), 3.39 (s, 3H), 3.36 (s, 3H). **$^{13}$C NMR** (126 MHz, chloroform-*d*) δ 203.0, 86.1, 79.7, 78.7, 69.5, 60.2, 59.3, 59.1, 57.6.

*2,3,4,5-Tetra-O-methyl-D-xylose (**1ac**):*

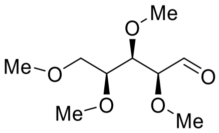


Following the **procedure A**, the title compound was prepared using D-Xylose as starting material, and then purified by flash chromatography with hexanes/ethyl acetate (4:1) as the eluent. **1ac:** colorless oil; **$^{1}$H NMR** (400 MHz, chloroform-*d*) δ 9.69 (s, 1H), 3.77 (d, *J* = 4.6 Hz, 1H), 3.68 (dd, *J* = 4.6, 2.8 Hz, 1H), 3.56 – 3.49 (m, 2H), 3.48 (m, 1H),3.47(s, 3H), 3.46(s, 3H), 3.33(s, 3H), 3.30 (s, 3H). **$^{13}$C NMR** (126 MHz, chloroform-*d*) δ 200.7, 83.3, 81.2, 78.01, 70.8, 60.0, 59.1, 59.0, 58.3.

*2,3,4,5,6-Penta-O-methyl-D-galactose (**1ad**):*

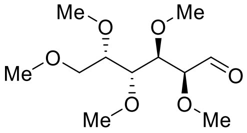

Following the **procedure A**, the title compound was prepared using D-Galactose as starting material, and then purified by flash chromatography with hexanes/ethyl acetate (4:1) as the eluent. **1ad:** colorless oil; **$^{1}$H NMR** (500 MHz, chloroform-*d*) δ 9.86 (s, 1H), 3.85 – 3.80 (m, 2H), 3.65 (dd, *J* = 8.7, 5.1 Hz, 1H), 3.61 – 3.56 (m, 2H), 3.51 – 3.49 (m, 1H), 3.46 (s, 3H), 3.42 (s, 3H), 3.38 (s, 3H), 3.34 (s, 3H). **$^{13}$C NMR** (126 MHz, chloroform-*d*) δ 202.9, 85.9, 79.7, 79.0, 78.0, 71.3, 60.6, 60.0, 59.0, 58.7, 58.5.

*2,3,4,5,6-Penta-O-methyl-D-glucose (**1ae**):*

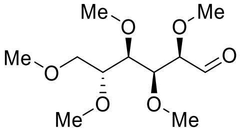

Following the **procedure A**, the title compound was prepared using D-Glucose as starting material, and then purified by flash chromatography with hexanes/ethyl acetate (4:1) as the eluent. **1ae:** colorless oil; **$^{1}$H NMR** (500 MHz, chloroform-*d*) δ 9.74 (s, 1H), 3.88 (dd, *J* = 5.7, 2.3 Hz, 1H), 3.85 (d, *J* = 5.7 Hz, 1H), 3.74 (dd, *J* = 10.6, 2.4 Hz, 1H), 3.57 (s, 3H), 3.53 (m, 1H), 3.51 (s, 3H), 3.49 – 3.44 (m, 2H), 3.43 (s, 3H), 3.39 (s, 3H), 3.25 (s, 3H). **$^{13}$C NMR** (126 MHz, chloroform-*d*) δ 199.6, 82.4, 81.4, 79.0, 77.5, 69.3, 59.6, 59.5, 59.2, 59.0, 57.3.

*6-Deoxy-2,3,4,5-tetra-O-methyl-L-fucose (**1af**):*

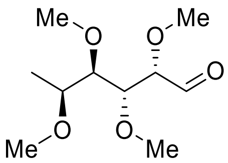


Following the **procedure A**, the title compound was prepared using L-Fucose as starting material, and then purified by flash chromatography with hexanes/ethyl acetate (4:1) as the eluent. **1af:** colorless oil; **$^1$H NMR** (500 MHz, chloroform-*d*) δ 9.86 (s, 1H), 3.87 – 3.79 (m, 2H), 3.56 (dd, *J* = 6.4, 1.9 Hz, 1H), 3.53 (s, 3H), 3.47 (s, 3H), 3.35 (s, 3H), 3.34 (s, 3H), 3.21 (dd, *J* = 8.8, 2.0 Hz, 1H), 1.28 (d, *J* = 6.4 Hz, 3H). **$^{13}$C NMR** (126 MHz, chloroform-*d*) δ 203.1, 85.9, 82.6, 79.8, 74.8, 61.2, 60.2, 58.6, 56.2, 15.0.

*(R)-2,2-dimethyl-1,3-dioxolane-4-carbaldehyde (**1ag**):*

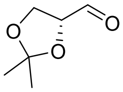

The title compound was prepared according to the procedure reported in the literature using D-Mannitol as starting material (*29*). **1ag:** colorless oil; **$^1$H NMR** (500 MHz, chloroform-*d*) δ 9.72 (d, *J* = 1.9 Hz, 1H), 4.39 (ddd, *J* = 7.1, 4.7, 1.9 Hz, 1H), 4.17 (dd, *J* = 8.8, 7.4 Hz, 1H), 4.10 (dd, *J* = 8.9, 4.7 Hz, 1H), 1.49 (s, 3H), 1.42 (s, 3H). **$^{13}$C NMR** (101 MHz, chloroform-*d*) δ 201.8, 111.3,79.8, 65.6, 26.2, 25.1.

*2,3:4,5-Bis-O-(1-methylethylidene)-D-ribose (**1ah**):*

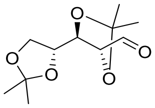

Following the **procedure B**, this compound was prepared using D-Ribose as starting material, and then purified by flash chromatography with hexanes/ethyl acetate (4:1) as the eluent.**1ah:** colorless oil; **$^1$H NMR** (500 MHz, chloroform-*d*) δ 9.72 (d, *J* = 1.9 Hz, 1H), 4.61 (dd, *J* = 6.8, 1.9 Hz, 1H), 4.33 – 4.25 (m, 1H), 4.12 – 4.08 (m, 2H), 3.94 – 3.86 (m, 1H), 1.54 (s, 3H), 1.41 (s, 3H), 1.38 (s, 3H), 1.31 (s, 3H). **$^{13}$C NMR** (126 MHz, chloroform-*d*) δ 197.6, 111.3, 110.2, 81.8, 78.8, 73.6, 67.5, 27.4, 26.7, 25.5, 25.1.

*2,3:4,5-Bis-O-(1-methylethylidene)-L-arabinose (**1ai**):*

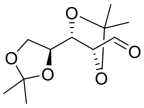

Following the **procedure B**, the title compound was prepared using L-Arabinose as starting material, and then purified by flash chromatography with hexanes/ethyl acetate (4:1) as the eluent. **1ai:** colorless oil; **$^{1}$H NMR** (500 MHz, chloroform-*d*) δ 9.72 (d, *J* = 1.1 Hz, 1H), 4.44 (dd, *J* = 6.0, 1.0 Hz, 1H), 4.11 – 3.93 (m, 3H), 3.60 (t, *J* = 7.5 Hz, 1H), 1.44 (s, 3H), 1.38 (s, 3H), 1.35 (s, 3H), 1.32 (s, 3H). **$^{13}$C NMR** (126 MHz, chloroform-*d*) δ 199.9, 111.9, 110.0, 83.3, 77.7, 76.4, 67.0, 27.0, 26.7, 26.2, 25.1.

*4-O-(methyl)-2,3:5,6-bis-O-(1-methylethylidene)-D-galactose (**1aj**):*

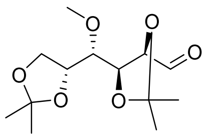

The title compound was prepared according to the procedure reported in the literature using D-Galactose as starting material (*30*). **1aj**: colorless oil; **$^{1}$H NMR** (500 MHz, chloroform-*d*) δ 9.75 (s, 1H), 4.46 (d, *J* = 5.9 Hz, 1H), 4.20 – 4.09 (m, 2H), 3.88 (t, *J* = 7.4 Hz, 1H), 3.61 (dd, *J* = 10.4, 3.2 Hz, 1H), 3.50 (dd, *J* = 10.4, 6.2 Hz, 1H), 3.40 (s, 3H), 1.46 (s, 3H), 1.42 (s, 3H), 1.38 (s, 3H), 1.37 (s, 3H). **$^{13}$C NMR** (126 MHz, chloroform-*d*) δ 199.9, 112.0, 110.2, 83.2, 78.9, 78.0, 77.8, 72.9, 59.5, 27.1, 26.9, 26.9, 26.3.

*1,2:3,4-Bis-O-(1-methylethylidene)-α-D-galacto-hexodialdo-1,5-pyranose (**1ak**):*

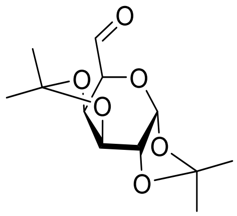

The title compound was prepared according to the procedure reported in the literature using D-Galactose as starting material (*31*). **1ak**: colorless oil; **$^{1}$H NMR** (500 MHz, chloroform-*d*) δ 9.61 (s, 1H), 5.66 (d, *J* = 4.9 Hz, 1H), 4.64 (dd, *J* = 7.9, 2.4 Hz, 1H), 4.59 (dd, *J* = 7.8, 2.2 Hz, 1H), 4.38 (dd, *J* = 5.0, 2.5 Hz, 1H), 4.18 (d, *J* = 2.2 Hz, 1H), 1.50 (s, 3H), 1.43 (s, 3H), 1.34 (s, 3H), 1.31 (s, 3H). **$^{13}$C NMR** (126 MHz, chloroform-*d*) δ 200.3, 110.0, 109.0, 96.2, 73.2, 71.7, 70.5, 70.4, 26.0, 25.8, 24.8, 24.2.

*2,3:4,5-Bis-O-(1-methylethylidene)-β-D-arabino-hexos-2-ulo-2,6-pyranose (**1al**):*

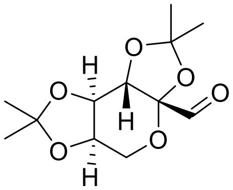

The title compound was prepared according to the procedure reported in the literature using D-Fructose as starting material (*31-32*). **1al:** colorless oil; **$^{1}$H NMR** (500 MHz, chloroform-*d*) δ 9.52 (s, 1H), 4.61 (dd, *J* = 8.0, 2.6 Hz, 1H), 4.49 (d, *J* = 2.5 Hz, 1H), 4.27 (d, *J* = 7.9 Hz, 1H), 3.95 (d, *J* = 12.8 Hz, 1H), 3.88 (d, *J* = 12.8 Hz, 1H), 1.55 (s, 3H), 1.42 (s, 3H), 1.40 (s, 3H), 1.33 (s, 3H). **$^{13}$C NMR** (126 MHz, chloroform-*d*) δ 195.1, 110.3, 109.3, 100.5, 70.9, 70.4, 69.7, 61.5, 26.1, 26.0, 24.8, 24.3.

*Methyl 2,3-O-(1-methylethylidene)-β-D-ribo-pentodialdo-1,4-furanoside (**1am**):*

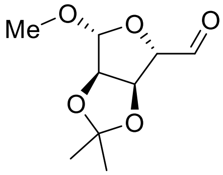


The title compound was prepared according to the procedure reported in the literature using D-ribose as starting material (*33*).**1am:** colorless oil; **$^{1}$H NMR** (500 MHz, chloroform-*d*) δ 9.57 (s, 1H), 5.07 (s, 1H), 5.04 (d, *J* = 5.7 Hz, 1H), 4.49 (d, *J* = 5.9 Hz, 1H), 4.46 (s, 1H), 3.44 (s, 3H), 1.48 (s, 3H), 1.32 (s, 3H). **$^{13}$C NMR** (126 MHz, chloroform-*d*) δ 200.8, 112.7, 109.2, 89.5, 84.0, 80.8, 55.8, 26.2, 24.9.

**2. General procedure for the synthesis of hydrazone or hydrazone solution**.[7]

**1) Synthesis of protected carbohydrates (1aa – 1am):**

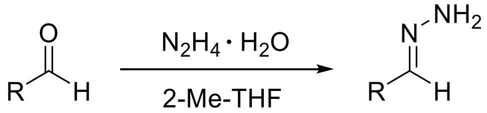


The hydrazone solution was prepared with minor alteration according to the previous literature (*34-36*). Hydrazone solution (**1M**): To a mixture of hydrazine monohydrate (117.0 µL, 2.4 mmol, 1.2 equiv, from Aldrich, $N_2H_4$ 64-65 wt%) in 2-Me-THF (0.5 mL) cooled to 0 °C, protected monosaccharide (2.0 mmol, 1.0 equiv) in 2-Me-THF (0.5 mL) was added dropwise. The mixture was stirred at room temperature for 90 min before anhydrous $Na_2SO_4$ (150 mg) was added. The resulting mixture was further stirred for 15 min. After that, the solution was transferred into a 2.0

mL volumetric flask and was diluted to 2.0 mL with 2-Me-THF. The hydrazone solution was dried by 4 Å Molecular Sieves (200mg) and stored in a freezer.

**2) Synthesis of unprotected carbohydrates (1an – 1aq):**

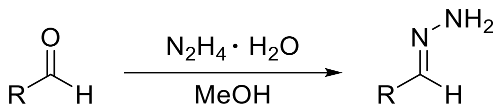


A mixture of monohydrate (5.0 mmol, 1.0 equiv) and hydrazine monohydrate (316.0 µL, 6.5 mmol, 1.3 equiv, from Aldrich, $N_2H_4$ 64-65 wt%) in MeOH (15 mL) was stirred at room temperature for 12 hr. The white precipitate was collected on a funnel and washed with hexane (20 mL). The white solid was dried under reduced pressure for 24 hr before it was stored in a freezer. The hydrazones were stable enough for at least six months.

*2-(E)-L-Fucose hydrazone (**1an**):*

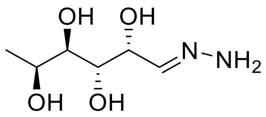


Following the general procedure, the title compound was prepared from L-Fucose, **1an**: white solid; **$^{1}$H NMR** (400 MHz, deuterium oxide) δ 7.35 (d, *J* = 5.3 Hz, 1H), 4.43 (dd, *J* = 5.4, 2.6 Hz, 1H), 4.08 (qd, *J* = 6.5, 1.8 Hz, 1H), 3.72 (dd, *J* = 9.0, 2.6 Hz, 1H), 3.48 (dd, *J* = 9.0, 1.9 Hz, 1H), 1.23 (d, *J* = 6.6 Hz, 3H); **$^{13}$C NMR** (101 MHz, deuterium oxide) δ 149.0, 72.8, 71.8, 70.4, 65.9, 18.6.

*2-(E)-D-Arabinose hydrazone (**1ao**):*

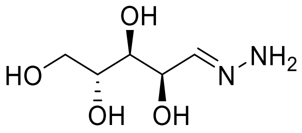


Following the general procedure, the title compound was prepared from D-Arabinose, **1ao**: white solid; **$^{1}$H NMR** (500 MHz, deuterium oxide) δ 7.34 (dd, *J* = 5.5, 2.2 Hz, 1H), 4.41 – 4.36 (m, 1H), 3.84 – 3.79 (m, 1H), 3.75 (ddd, *J* = 8.7, 5.7, 2.9 Hz, 1H), 3.69 – 3.63 (m, 2H) ; **$^{13}$C NMR** (101 MHz, deuterium oxide) δ 148.5, 72.4, 70.8, 70.2, 62.7.

*2-(E)-L-Arabinose hydrazone (**1ap**):*

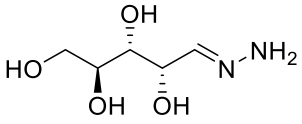

Following the general procedure, the title compound was prepared from L-Arabinose, **1ap**: white solid; **$^{1}$H NMR** (500 MHz, deuterium oxide) δ 7.35 (d, *J* = 5.6 Hz, 1H), 4.43 – 4.34 (m, 1H), 3.81 (m, 1H), 3.76 (m, 1H), 3.67 (m, 2H); **$^{13}$C NMR** (101 MHz, Deuterium oxide) δ 148.6, 72.4, 70.8, 70.2, 62.7.

*2-(E)-L-Rhamnose hydrazone (**1aq**):*

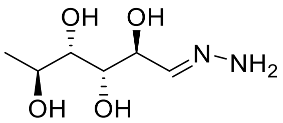

Following the general procedure, the title compound was prepared from L-Rhamnose, **1aq**: white solid; **$^{1}$H NMR** (400 MHz, deuterium oxide) δ 7.35 (dd, *J* = 6.4, 1.4 Hz, 1H), 4.24 – 4.15 (m, 1H), 3.89 (m,1.8 Hz, 2H), 3.57 (m, 1H), 1.27 (d, *J* = 5.8 Hz, 3H); **$^{13}$C NMR** (101 MHz, Deuterium oxide) δ 148.4, 73.3, 70.8, 70.6, 66.9, 18.5.

*1-((4R,4'S,5S)-2,2,2',2'-tetramethyl-[4,4'-bi(1,3-dioxolan)]-5-yl)but-3-en-1-one (**1as**):*

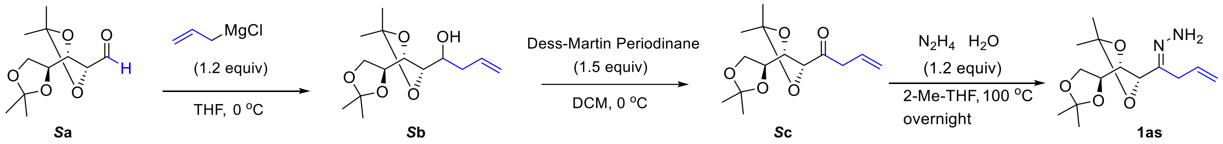


In an argon-filled flask, ***Sa*** (3.0 mmol, 1.0 equiv) in THF (2.0 mL) was added dropwise to a solution of allylmagnesium chloride solution (1.8 mL, 2 M in THF, 3.6 mmol, 1.2 equiv) at 0 °C and the mixture was stirred for 30 min and then 1 hr at room temperature. The reaction was quenched by the addition of a saturated solution of $NH_4Cl$ (5 mL). Then the organic layer was separated, and the aqueous layer was extracted with $CH_2Cl_2$ (3 × 10 mL). The combined organic layers were dried over $Na_2SO_4$. After the volatile were removed, the crude product was purified by flash chromatography. The obtained allyl alcohol ***Sb*** (2.5 mmol, 1.0 equiv) was dissolved in $CH_2Cl_2$ (15 mL) at 0 °C, and then the mixture was treated with DMP (1.59 g, 3.75 mmol, 1.5 equiv). After stirred 2 h, the reaction was quenched by the addition of a saturated aqueous solution of $Na_2S_2O_3$ (5 mL) and a saturated aqueous solution of $NaHCO_3$ (5 mL). After the solid was filtered off, the organic layer was separated, and the aqueous layer was extracted with $CH_2Cl_2$ (3 × 10 mL). The combined organic layers were dried over $Na_2SO_4$. After the volatile were removed, the crude product was dissolved in ether (10 mL), and then hexane (50 mL) was added. After the solid was filtered off, the volatile was removed to yield the colorless oil ***Sc*** which was used for next step directly. **$^{1}$H NMR** (500 MHz, chloroform-*d*) δ 5.98 – 5.87 (m, 1H), 5.23 – 5.09 (m, 2H), 4.38 (d, *J* = 5.5 Hz, 1H), 4.21 – 4.13 (m, 2H), 4.13 – 4.05 (m, 1H), 3.96 (dd, *J* = 8.6, 4.4 Hz, 1H), 3.44 (dd,

$J$ = 6.8, 1.5 Hz, 2H), 1.44 (s, 3H), 1.40 (s, 3H), 1.36 (s, 3H), 1.33 (s, 3H). **$^{13}$C NMR** (126 MHz, chloroform-*d*) δ 207.1, 129.8, 119.1, 111.4, 109.9, 82.5, 78.2, 76.5, 66.7, 43.9, 27.1, 26.5, 26.2, 25.1. Hydrazone Solution of **1as** was prepared by dropping ***Sc*** (1.0 mmol, 1.0 equiv) to hydrazine monohydrate (63 μL, 1.3 mmol, 1.3 equiv, from Aldrich, $N_2H_4$ 64-65 wt%) in THF (0.9 mL). the mixture was stirred at 100 °C overnight. The hydrazone solution was dried by anhydrous $Na_2SO_4$ (150 mg) and stored in a freezer.

**3. General procedure for the synthesis of allylic acetate**.

**1) Synthesis of substrates 2ba – 2bc:**

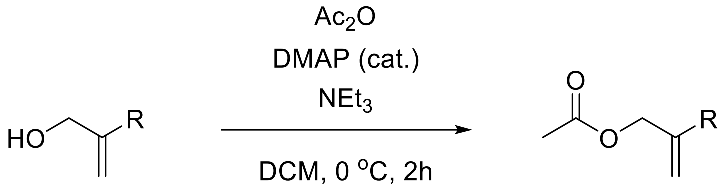


A solution of allyl alcohol (3.0 mmol, 1.0 equiv) in $CH_2Cl_2$ (15 mL) at 0 °C was treated with DMAP (36.7 mg, 0.3 mmol, 0.1 equiv), $NEt_3$ (831μL, 6.0 mmol, 2.0 equiv), and $Ac_2O$ (567 μL, 6.0 mmol, 2.0 equiv). After stirred for 2 hr, the reaction was quenched by the addition of water (2.0 mL). Then the organic layer was separated, and the aqueous layer was extracted with $CH_2Cl_2$ (3 × 10 mL). The combined organic layers were dried over $Na_2SO_4$. After the volatile were removed, the crude product was purified by flash chromatography on silica gel.

*2-methylallyl acetate* (**2ba**)

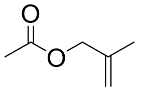


The title compound was prepared according to the general procedure and the spectroscopic data were consistent with the ones reported in the literature (*37*). **2ba**: colorless oil; **$^{1}$H NMR** (500 MHz, chloroform-*d*) δ 4.97 (s, 1H), 4.93 (s, 1H), 4.49 (s, 2H), 2.09 (s, 3H), 1.76 (s, 3H). **$^{13}$C NMR** (126 MHz, chloroform-*d*) δ 170.7, 139.9, 112.9, 67.7, 20.9, 19.5.

*2-chloroallyl acetate* (**2bb**)

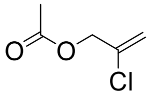


The title compound was prepared according to the general procedure and the spectroscopic data were consistent with the ones reported in the literature (*37*). **2bb**: colorless oil; **$^{1}$H NMR** (500 MHz,

chloroform-*d*) δ 5.46 (s, 1H), 5.40 (s, 1H), 4.64 (s, 2H), 2.12 (s, 3H). **$^{13}$C NMR** (126 MHz, chloroform-*d*) δ 170.1, 135.9, 114.9, 66.0, 20.7.

*2-phenylallyl acetate (**2bc**)*

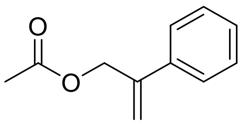

The title compound was prepared according to the general procedure and the spectroscopic data were consistent with the ones reported in the literature (*37*). **2bc**: yellow oil; $R_f$ = 0.60 (hexane/EtOAc, 9:1); **$^{1}$H NMR** (400 MHz, chloroform-*d*) δ 7.49 – 7.40 (m, 2H), 7.35 (m, 3H), 5.56 (s, 1H), 5.37 (s, 1H), 4.99 (s, 2H), 2.08 (s, 3H). **$^{13}$C NMR** (101 MHz, chloroform-*d*) δ 170.8, 142.5, 138.0, 128.5, 128.1, 126.0, 115.3, 65.7, 21.0.

**2) Synthesis of substrates 2bd-2bf, and 2bi-2bo:**

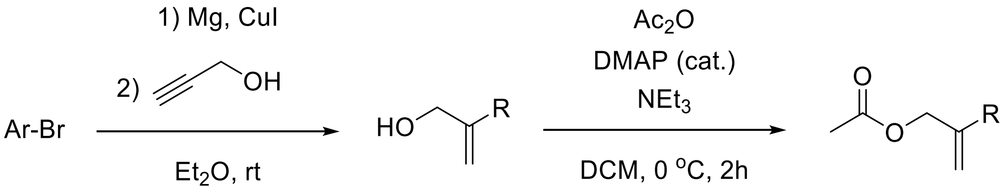


The title compound was prepared according to the procedure reported in the literature (*38*). A two necked round bottom flask under argon was charged with magnesium turning (180 mg, 7.5 mmol) and dry $Et_2O$ (10 mL) at room temperature. A solution of aryl bromide (7.5 mmol) in dry $Et_2O$ (10 mL) was added dropwise over 20 min. The mixture was then heated to reflux for 2 hr. After cooled to room temperature, CuI (83 mg, 0.45 mmol) was added, and the reaction mixture was stirred for another 0.5 hr. Propargyl alcohol (168 mg, 3.0 mmol) in $Et_2O$ (5 mL) was added dropwise. The resulting mixture was refluxed for 6 hr. After the reaction mixture was cooled to 0 °C, saturated aqueous $NH_4Cl$ (5 mL) was added carefully. The organic layer was separated, and the aqueous layer was extracted with $Et_2O$ (3 × 20 mL). the combined organic layers were dried over $MgSO_4$. After concentrated under reduced pressure, the crude product was used in the next step directly. The allyl alcohol was dissolved in $CH_2Cl_2$ (15 mL) at 0 °C, and then the mixture was treated with DMAP (36.7 mg, 0.3 mmol, 0.1 equiv), $NEt_3$ (831 µL, 6.0 mmol, 2.0 equiv), and $Ac_2O$ (567µL, 6.0 mmol, 2.0 equiv). After stirred for 2 hr, the reaction was quenched by the addition of water (2.0 mL). Then the organic layer was separated, and the aqueous layer was extracted with $CH_2Cl_2$ (3 × 10 mL). The combined organic layers were dried over $Na_2SO_4$. After the volatiles were removed, the crude product was purified by flash chromatography on silica gel.

*2-(4-chlorophenyl)allyl acetate (**2bd**):*

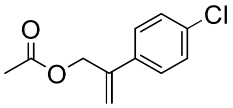

The title compound was prepared according to the general procedure. **2bd**: colorless oil; $R_f$ = 0.55 (hexane/EtOAc, 9:1); **$^{1}$H NMR** (500 MHz, chloroform-*d*) δ 7.36 (d, *J* = 8.7 Hz, 2H), 7.32 (d, *J* = 8.7 Hz, 2H), 5.54 (s, 1H), 5.38 (s, 1H), 4.95 (s, 2H), 2.07 (s, 3H). **$^{13}$C NMR** (126 MHz, chloroform-*d*) δ 170.7, 141.5, 136.5, 133.9, 128.7, 127.3, 116.0, 65.5, 20.9.

*2-(4-methoxyphenyl)allyl acetate (**2be**):*

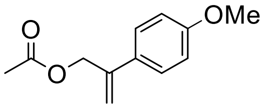

The title compound was prepared according to the general procedure and the spectroscopic data were consistent with the ones reported in the literature (*39*). **2be**: colorless oil; $R_f$ = 0.35 (hexane/EtOAc, 9:1); **$^{1}$H NMR** (500 MHz, chloroform-*d*) δ 7.40 – 7.33 (m, 2H), 6.90 – 6.86 (m, 2H), 5.47 (s, 1H), 5.27 (s, 1H), 4.95 (s, 2H), 3.82 (s, 3H), 2.08 (s, 3H). **$^{13}$C NMR** (126 MHz, chloroform-*d*) δ 170.8, 159.5, 141.8, 130.5, 127.1, 113.8, 113.7, 65.9, 55.3, 21.0.

*2-(2,4-dimethylphenyl)allyl acetate (**2bf**):*

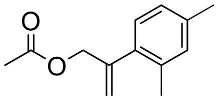

The title compound was prepared according to the general procedure. **2bf**: colorless oil; $R_f$ = 0.63 (hexane/EtOAc, 9:1); **$^{1}$H NMR** (500 MHz, chloroform-*d*) δ 7.00 (m, 3H), 5.42 (s, 1H), 5.07 (s, 1H), 4.74 (s, 2H), 2.32 (s, 3H), 2.28 (s, 3H), 2.07 (s, 3H). **$^{13}$C NMR** (126 MHz, chloroform-*d*) δ 170.6, 144.0, 137.2, 136.2, 135.3, 131.0, 128.6, 126.2, 115.6, 66.8, 21.0, 20.9, 19.6.

*2-(2-fluoro-4-methoxyphenyl)allyl acetate (**2bi**):*

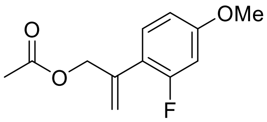

The title compound was prepared according to the general procedure. **2bi**: yellow oil; $R_f$ = 0.38 (hexane/EtOAc, 9:1); **$^{1}$H NMR** (500 MHz, chloroform-*d*) δ 7.22 (t, *J* = 8.7 Hz, 1H), 6.68 (dd, *J* = 8.6, 2.5 Hz, 1H), 6.62 (dd, *J* = 12.8, 2.5 Hz, 1H), 5.43 (s, 1H), 5.40 (s, 1H), 4.90 (s, 2H), 3.81 (s, 3H), 2.05 (s, 3H). **$^{13}$C NMR** (126 MHz, chloroform-*d*) δ 170.6, 160.7 (d, *J* = 249.7Hz), 160.6 (d, *J* = 11.1Hz), 138.8, 130.2 (d, *J* = 6.2 Hz), 118.7 (d, *J* = 14.4 Hz), 117.2 (d, *J* = 3.5 Hz), 110.0 (d,

$J$ = 3.0 Hz), 101.9 (d, $J$ = 26.6 Hz), 66.3 (d, $J$ = 4.4 Hz), 55.6, 20.9. **$^{19}$F NMR** (470 MHz, chloroform-*d*) δ -112.35.

*2-(3-(trifluoromethyl)phenyl)allyl acetate (**2bl**):*

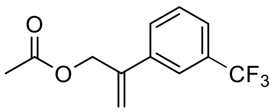


The title compound was prepared according to the general procedure. **2bl**: colorless oil; $R_f$ = 0.42 (hexane/EtOAc, 9:1); **$^1$H NMR** (500 MHz, chloroform-*d*) δ 7.67 (s, 1H), 7.58 (dd, $J$ = 14.1, 7.8 Hz, 2H), 7.48 (t, $J$ = 7.8 Hz, 1H), 5.61 (s, 1H), 5.47 (s, 1H), 4.98 (s, 2H), 2.08 (s, 3H). **$^{13}$C NMR** (126 MHz, chloroform-*d*) δ 170.6, 141.5, 138.9, 130.9 (q, $J$ = 32.2 Hz), 129.3, 129.0, 124.7 (q, $J$ = 3.8 Hz), 124.0 (q, $J$ = 273.4 Hz), 122.9 (q, $J$ = 4.0 Hz), 117.0, 65.4, 20.9.

*2-benzylallyl acetate (**2bm**):*

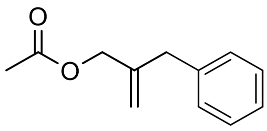


The title compound was prepared according to the general procedure and the spectroscopic data were consistent with the ones reported in the literature (*40*). **2bm**: colorless oil; $R_f$ = 0.5 (hexane/EtOAc, 9:1); **$^1$H NMR** (500 MHz, chloroform-*d*) δ 7.30 (t, $J$ = 7.4 Hz, 2H), 7.25 – 7.14 (m, 3H), 5.13 (s, 1H), 4.97 (s, 1H), 4.49 (s, 2H), 3.41 (s, 2H), 2.05 (s, 3H). **$^{13}$C NMR** (126 MHz, chloroform-*d*) δ 170.7, 143.2, 138.5, 128.9, 128.4, 126.4, 114.3, 66.3, 40.2, 20.9.

*2-(thiophen-2-yl)allyl acetate (**2bo**):*

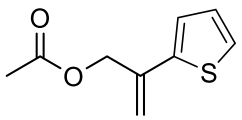


The title compound was prepared according to the general procedure and the spectroscopic data were consistent with the ones reported in the literature (*40*). **2bo**: yellow oil; **$^1$H NMR** (400 MHz, chloroform-*d*) δ 7.22 (d, $J$ = 5.1 Hz, 1H), 7.07 (d, $J$ = 3.7 Hz, 1H), 7.00 (ddd, $J$ = 5.0, 3.6, 1.2 Hz, 1H), 5.59 (s, 1H), 5.26 (s, 1H), 4.93 (s, 2H), 2.12 (s, 3H).

*2-methylene-4-(triisopropylsilyl)but-3-yn-1-yl acetate (**2bp**):*

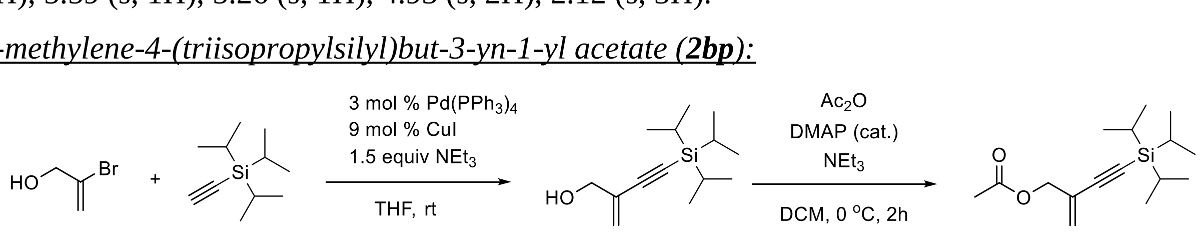

The title compound was prepared according to the procedure reported in the literature (*41*). In an argon flushed two-necked flask, $Pd(PPh_3)_4$ (173 mg, 0.15 mmol) and CuI (85.7 mg, 0.45 mmol) were dissolved in 10 mL of THF. 2-Bromoallyl alcohol (5.0 mmol) and triethylamine (1.05 mL) were added successively. The mixture was stirred at room temperature for 5 min before (triisopropylsilyl)acetylene (1.18 g, 6.5 mmol) was added via syringe. The resulting mixture was stirred at room temperature for 16 hr. The reaction mixture was diluted with 10 mL $Et_2O$ and passed through silica gel. After concentrated under reduced pressure, the liquid was dissolved in $CH_2Cl_2$ (15 mL) at 0 $^oC$, and then the mixture was treated with DMAP (61.2 mg, 0.5 mmol, 0.1 equiv), $NEt_3$ (1.39 mL, 10.0 mmol, 2.0 equiv), and $Ac_2O$ (945 µL, 10.0 mmol, 2.0 equiv). After stirred for 2 hr, the reaction was quenched by the addition of water (2.0 mL). Then the organic layer was separated, and the aqueous layer was extracted with $CH_2Cl_2$ (3 × 10 mL). The combined organic layers were dried over $Na_2SO_4$. After the volatiles were removed, the crude product was purified by flash chromatography on silica gel. **2bp**: yellow oil; $R_f$ = 0.58 (hexane/EtOAc, 9:1); **$^1$H NMR** (500 MHz, chloroform-*d*) δ 5.56 (s, 1H), 5.50 (s, 1H), 4.59 (s, 2H), 2.10 (s, 3H), 1.08 (s, 21H). **$^{13}$C NMR** (126 MHz, chloroform-*d*) δ 170.3, 126.7, 122.9, 104.1, 92.6, 65.8, 20.8, 18.6, 11.2.

**3) Synthesis of substrates 2bq-2bt:**

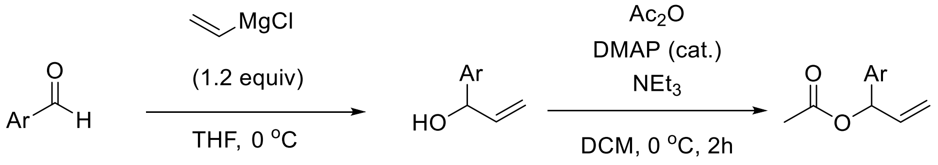


The title compound was prepared with minor alteration according to the procedure reported in the literature (*42*). In an argon-filled flask, aldehyde (3.0 mmol, 1.0 equiv) in THF (2.0 mL) was added dropwise to a solution of vinylmagnesium chloride solution (2.25 mL, 1.6 M in THF, 3.6 mmol, 1.2 equiv) at 0 $^oC$ and the mixture was stirred for 30 min and then another 1hr at room temperature. The reaction was quenched by the addition of a saturated solution of $NH_4Cl$ (5 mL). Then the organic layer was separated, and the aqueous layer was extracted with $CH_2Cl_2$ (3 × 10 mL). The combined organic layers were dried over $Na_2SO_4$. After the volatiles were removed, the crude product was used in the next step directly.

The allyl alcohol was dissolved in $CH_2Cl_2$ (15 mL) at 0 $^oC$, and then the mixture was treated with DMAP (36.7 mg, 0.3 mmol, 0.1 equiv), $NEt_3$ (831 µL, 6.0 mmol, 2.0 equiv), and $Ac_2O$ (567 µL, 6.0 mmol, 2.0 equiv). After stirred for 2 hr, the reaction was quenched by the addition of water

(2.0 mL). Then the organic layer was separated, and the aqueous layer was extracted with $CH_2Cl_2$ (3 × 10 mL). The combined organic layers were dried over $Na_2SO_4$. After the volatiles were removed, the crude product was purified by flash chromatography on silica gel.

*1-phenylallyl acetate (**2bq**):*

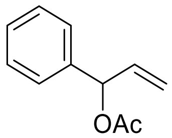

The title compound was prepared according to the general procedure. **2bq**: colorless oil; $R_f$ = 0.5 (hexane/EtOAc, 9:1); **$^1$H NMR** (500 MHz, chloroform-*d*) δ 7.38 – 7.35 (m, 4H), 7.34 – 7.28 (m, 1H), 6.27 (dt, *J* = 6.0, 1.4 Hz, 1H), 6.01 (ddd, *J* = 17.1, 10.4, 5.9 Hz, 1H), 5.30 (dt, *J* = 17.1, 1.4 Hz, 1H), 5.25 (dt, *J* = 10.4, 1.3 Hz, 1H), 2.12 (s, 3H). **$^{13}$C NMR** (126 MHz, chloroform-*d*) δ 170.0, 138.9, 136.3, 128.6, 128.2, 127.1, 116.9, 76.2, 21.3.

*1-(3-chlorophenyl)allyl acetate (**2br**):*

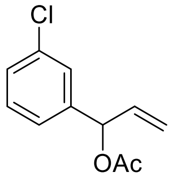

The title compound was prepared according to the general procedure. **2br**: light yellow oil; $R_f$ = 0.55 (hexane/EtOAc, 9:1); **$^1$H NMR** (500 MHz, chloroform-*d*) δ 7.34 (s, 1H), 7.30 – 7.27 (m, 2H), 7.24 – 7.21 (m, 1H), 6.22 (d, *J* = 6.0 Hz, 1H), 6.01 – 5.91 (m, 1H), 5.34 – 5.23 (m, 2H), 2.13 (s, 3H). **$^{13}$C NMR** (126 MHz, chloroform-*d*) δ 169.8, 140.9, 135.7, 134.5, 129.8, 128.3, 127.2, 125.3, 117.5, 75.4, 21.2.

*1-(3-(trifluoromethyl)phenyl)allyl acetate (**2bs**):*

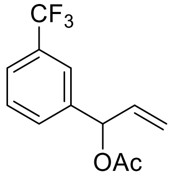

The title compound was prepared according to the general procedure. **2bs**: colorless oil; $R_f$ = 0.5 (hexane/EtOAc, 9:1); **$^1$H NMR** (500 MHz, chloroform-*d*) δ 7.61 (s, 1H), 7.57 (d, *J* = 7.8 Hz, 1H), 7.55 – 7.52 (m, 1H), 7.48 (t, *J* = 7.7 Hz, 1H), 6.30 (d, *J* = 6.0 Hz, 1H), 5.98 (ddd, *J* = 16.8, 10.4, 6.0 Hz, 1H), 5.36 – 5.26 (m, 2H), 2.14 (s, 3H). **$^{13}$C NMR** (126 MHz, chloroform-*d*) δ 169.8, 139.9,

135.6, 131.0 (q, $J$ = 32.2 Hz), 130.5 (q, $J$ = 1.32 Hz), 129.0, 125.0 (q, $J$ = 3.9 Hz), 123.8 (q, $J$ = 3.6 Hz), 123.9 (q, $J$ = 271.9Hz), 117.8, 75.4, 21.2 . **$^{19}$F NMR** (470 MHz, chloroform-*d*) δ -62.65.

*1-(4-(methylthio)phenyl)allyl acetate (**2bt**):*

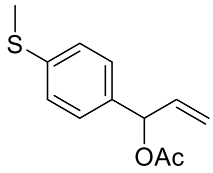


The title compound was prepared according to the general procedure. **2bt**: colorless oil; $R_f$ = 0.37 (hexane/EtOAc, 9:1); **$^1$H NMR** (500 MHz, chloroform-*d*) δ 7.27 (d, $J$ = 8.4 Hz, 2H), 7.23 (d, $J$ = 8.4 Hz, 2H), 6.21 (d, $J$ = 5.5 Hz, 1H), 5.98 (ddd, $J$ = 16.7, 10.5, 5.8 Hz, 1H), 5.32 – 5.20 (m, 2H), 2.47 (s, 3H), 2.09 (s, 3H). **$^{13}$C NMR** (126 MHz, chloroform-*d*) δ 169.9, 138.7, 136.1, 135.7, 127.8, 126.6, 116.9, 75.8, 21.2, 15.7.

*1-(thiophen-3-yl)allyl acetate (**2bu**):*

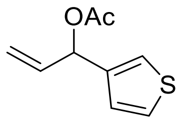


The title compound was prepared according to the general procedure. **2bu**: light yellow oil; $R_f$ = 0.38 (hexane/EtOAc, 9:1); **$^1$H NMR** (500 MHz, chloroform-*d*) δ 7.33 (td, $J$ = 2.9, 1.4 Hz, 1H), 7.29 (s, 1H), 7.08 (d, $J$ = 5.1 Hz, 1H), 6.39 (d, $J$ = 5.8 Hz, 1H), 6.12 – 6.00 (m, 1H), 5.39 – 5.24 (m, 2H), 2.13 (s, 3H). **$^{13}$C NMR** (126 MHz, chloroform-*d*) δ 170.0, 139.9, 135.6, 126.6, 126.2, 123.1, 117.2, 72.1, 21.3.

**4) Synthesis of substrates 2bg, 2bh, and 2bv-2bx:**

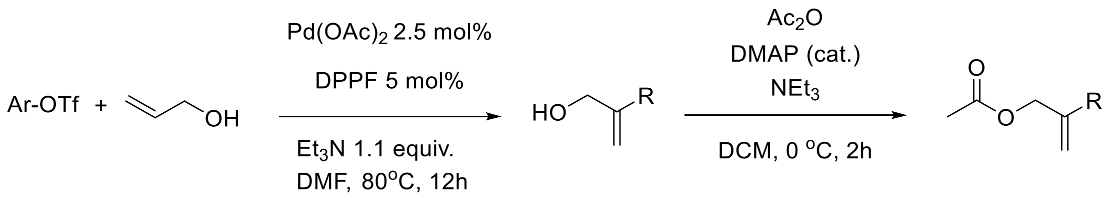


In an argon-filled flask, aryl triflate (3.0 mmol, 1.0 equiv), allyl alcohol (390 µL, 6.0 mmol, 2 equiv), $NEt_3$ (820 µL, 3.3 mmol, 1.1 equiv) and 5 mL of DMF were added and stirred for 5 min. $Pd(OAc)_2$ (16.9 mg, 0.075 mmol, 2.5 mol%) and DPPF (1,1'-bis(diphenylphosphino)ferrocene) (83.2 mg, 0.15 mmol, 5 mol%) were added and the resulting mixture was heated at 80 °C for 12 hr. After the mixture was cooled to rt, 10% HCl (10 mL) aqueous was added carefully and the aqueous layer was extracted with ethyl acetate (3 × 20 mL). The combined organic layers were

dried over $MgSO_4$. After concentrated under reduced pressure, the crude product was used in the next step directly. The allyl alcohol was dissolved in $CH_2Cl_2$ (15 mL) at 0 °C, and then the mixture was treated with DMAP (36.7 mg, 0.3 mmol, 0.1 equiv), $NEt_3$ (831 μL, 6.0 mmol, 2.0 equiv), and $Ac_2O$ (567μL, 6.0 mmol, 2.0 equiv). After stirred for 2 hr, the reaction was quenched by the addition of water (2.0 mL). Then the organic layer was separated, and the aqueous layer was extracted with $CH_2Cl_2$ (3 × 10 mL). The combined organic layers were dried over $Na_2SO_4$. After the volatiles were removed, the crude product was purified by flash chromatography on silica gel.

*2-(4-cyanophenyl)allyl acetate (**2bg**):*

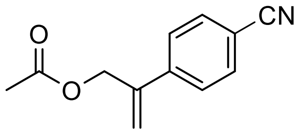


The title compound was prepared according to the general procedure. **2bg**: colorless oil; $R_f$ = 0.15 (hexane/EtOAc, 9:1); **$^1$H NMR** (500 MHz, chloroform-*d*) δ 7.67 – 7.62 (m, 2H), 7.55 – 7.50 (m, 2H), 5.66 (s, 1H), 5.53 (s, 1H), 4.97 (s, 2H), 2.07 (s, 3H). **$^{13}$C NMR** (126 MHz, chloroform-*d*) δ 170.6, 142.5, 141.3, 132.3, 126.7, 118.7, 118.6, 111.7, 65.2, 20.9.

*2-(4-(2,2,2-trifluoroacetamido)phenyl)allyl acetate (**2bh**):*

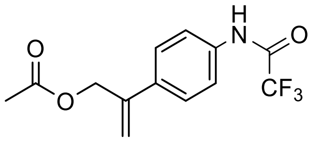


The title compound was prepared according to the general procedure. **2bh**: white solid; $R_f$ = 0.13 (hexane/EtOAc, 9:1); **$^1$H NMR** (500 MHz, chloroform-*d*) δ 7.94 (s, 1H), 7.62 (s, 1H), 7.55 (d, *J* = 10.0 Hz, 1H), 7.39 (t, *J* = 7.9 Hz, 1H), 7.30 (d, *J* = 7.8 Hz, 1H), 5.59 (s, 1H), 5.43 (s, 1H), 4.96 (s, 2H), 2.08 (s, 3H). **$^{13}$C NMR** (126 MHz, chloroform-*d*) δ 170.9, 154.9 (q, *J* = 37.4 Hz), 141.7, 139.5, 135.4, 129.5, 123.9, 120.1, 118.2, 116.7, 115.6 (q, *J* = 288.4 Hz), 65.6, 20.9.

*2-((8R,9S,13S,14S)-13-methyl-17-oxo-7,8,9,11,12,13,14,15,16,17-decahydro-6H-cyclopenta[a]phenanthren-3-yl)allyl acetate (**2bv**):*

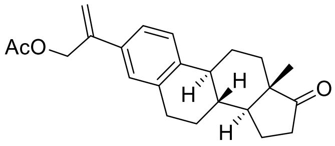


The title compound was prepared according to the general procedure. **2bv**: yellow solid; **$^1$H NMR** (500 MHz, chloroform-*d*) δ 7.28 (dd, *J* = 8.2, 1.0 Hz, 1H), 7.25 – 7.21 (m, 1H), 7.17 (s, 1H), 5.53

(s, 1H), 5.32 (s, 1H), 4.96 (s, 2H), 2.93 (dd, *J* = 9.0, 4.2 Hz, 2H), 2.55 – 2.46 (m, 1H), 2.46 – 2.39 (m, 1H), 2.31 (td, *J* = 10.9, 4.0 Hz, 1H), 2.17 (s, 1H), 2.09 (s, 3H), 2.08 – 2.01 (m, 2H), 2.00 – 1.94 (m, 1H), 1.65 (dd, *J* = 12.4, 3.4 Hz, 1H), 1.62 – 1.59 (m, 2H), 1.57 – 1.46 (m, 3H), 0.91 (s, 3H); **$^{13}$C NMR** (126 MHz, chloroform-*d*) δ 142.1, 139.8, 136.6, 126.5, 125.5, 123.4, 114.5, 65.7, 50.5, 48.0, 44.4, 38.1, 35.9, 31.6, 29.5, 26.5, 25.7, 21.6, 21.1, 13.9.

*methyl (S)-3-(4-(3-acetoxyprop-1-en-2-yl)phenyl)-2-((tert-butoxycarbonyl)amino)propanoate (**2bw**):*

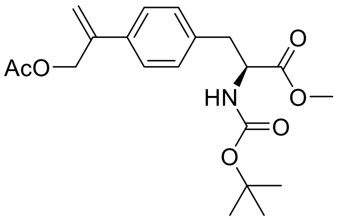


The title compound was prepared according to the general procedure. **2bw**: white solid; **$^{1}$H NMR** (500 MHz, chloroform-*d*) δ 7.35 (d, *J* = 8.1 Hz, 2H), 7.10 (d, *J* = 7.9 Hz, 2H), 5.54 (s, 1H), 5.34 (s, 1H), 4.99 (d, *J* = 8.4 Hz, 1H), 4.95 (s, 2H), 4.58 (q, *J* = 6.7 Hz, 1H), 3.72 (s, 3H), 3.16 – 2.99 (m, 2H), 2.07 (s, 3H), 1.41 (s, 9H); **$^{13}$C NMR** (126 MHz, chloroform-*d*) δ 172.2, 170.7, 155.0, 142.0, 136.7, 136.0, 129.5, 126.1, 115.1, 80.0, 65.7, 54.3, 52.3, 38.0, 28.3, 21.0.

*1,4-phenylenebis(prop-2-ene-2,1-diyl) diacetate (**2bx**):*

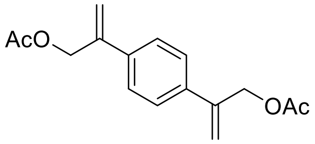


The title compound was prepared according to the general procedure. **2bx**: colorless oil; **$^{1}$H NMR** (500 MHz, chloroform-*d*) δ 7.42 (s, 4H), 5.59 (s, 2H), 5.38 (s, 2H), 4.98 (s, 4H), 2.08 (s, 6H); **$^{13}$C NMR** (126 MHz, chloroform-*d*) δ 170.8, 141.9, 137.6, 126.0, 115.6, 65.6, 21.0.

*(R)-6-vinyl-5,6-dihydro-2H-pyran-2-one (**2by**):*

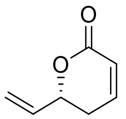


The title compound was prepared according to the procedure reported in the literature (*43*). $[\alpha]_D^{20}$ = +101.6 (c = 0.1, $CHCl_3$); **$^{1}$H NMR** (500 MHz, chloroform-*d*) δ 6.87 (ddd, *J* = 9.8, 5.5, 3.0 Hz, 1H), 6.03 (ddd, *J* = 9.8, 2.5, 1.3 Hz, 1H), 5.93 (ddd, *J* = 17.2, 10.6, 5.7 Hz, 1H), 5.38 (d, *J* = 17.3

Hz, 2H), 5.27 (d, $J$ = 10.6 Hz, 2H), 4.91 (dddd, $J$ = 10.4, 6.1, 4.4, 1.4 Hz, 1H), 2.52 – 2.35 (m, 3H). **$^{13}$C NMR** (126 MHz, chloroform-$d$) δ 163.8, 144.5, 134.8, 121.6, 117.9, 77.8, 29.4.

### *IV Experimental procedures for deoxygenative allylation or olefination*

**1. General procedure for the deoxygenative allylation of monosaccharides (Procedure C).**

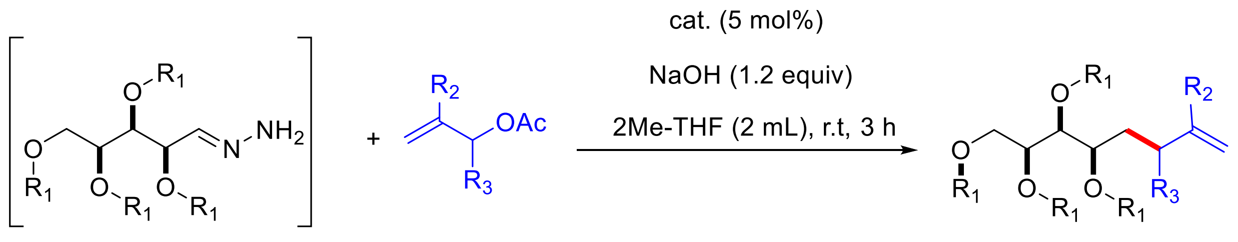


1 2 3

PEPPSI$^{TM}$-IPr catalyst (6.8 mg, 5 mol%), allyl acetate **2** (0.2 mmol), and 2-Me-THF (0.5 mL) were added into a dried microwave vial (10 mL) equipped with a stir bar in the glovebox. The reaction mixture was stirred at room temperature for 5 min before hydrazone solution **1** (0.24 mmol, 240 μL, 1M), NaOH (9.6 mg, 0.24 mmol), and 2-Me-THF (1.26 mL) were added. The reaction tube was sealed and moved out of the glovebox. The resulting mixture was stirred at room temperature for 3 hr. After the completion of the reaction, the reaction solution was filtered through a short celite pad and washed with diethyl ether (60 mL). The combined solution was removed under vacuum, and the residue was purified by flash column chromatography to give the desired product.

**2. General procedure for the deoxygenative olefination of monosaccharides (Procedure D).**

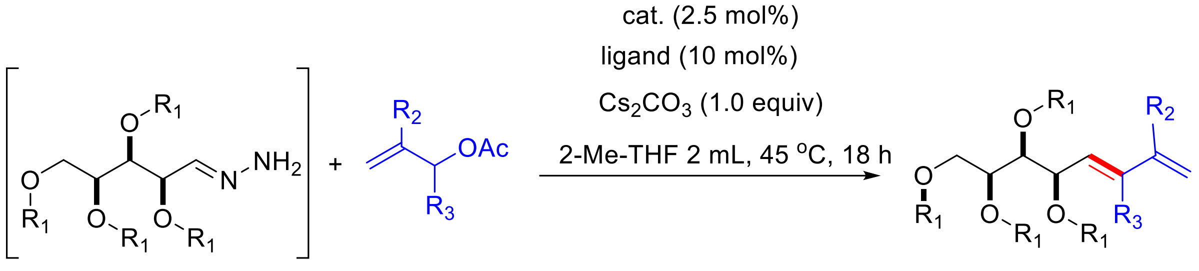


1 2 3

[Pd(allyl)Cl]$_2$ (1.9 mg, 2.5 mol%), tris(4-trifluoromethylphenyl)phosphine (9.4 mg, 10 mol%), allyl acetate **2** (0.6 mmol), and 2-Me-THF (0.5 mL) were added into a dried microwave vial (10 mL) equipped with a stir bar in the glovebox. The reaction mixture was stirred at room temperature for 5 min before hydrazone solution **1** (0.20 mmol, 200 μL, 1M), $Cs_2CO_3$ (65.2 mg, 0.2 mmol), and 2-Me-THF (1.3 mL) were added. The reaction tube was sealed and moved out of the glovebox. The resulting mixture was stirred at 45 °C for 18 hr. After the completion of the reaction, the

reaction solution was filtered through a short celite pad and washed with diethyl ether (60 mL). The combined solution was concentrated under vacuum, and the residue was purified by flash column chromatography on silica gel to give the desired product.

### *V. Characterization data for the products*

*(5S,6S,7R)-5,6,7,8-tetramethoxy-oct-1-ene (**3aa**):*

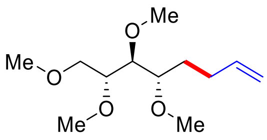


Following the **general procedure C**, the title compound was obtained from the reaction of hydrazone of **1aa** (0.24 mmol, 1.0 M generated *in situ* from **1aa** and hydrazine monohydrate) with allyl acetate (0.2 mmol). The product was further purified by column chromatography on silica gel. **3aa**: yield 90%, colorless oil, $R_f$ = 0.3 (hexane/EtOAc, 9:1); $[\alpha]_D^{22}$ = +11.9 (c = 1.0, $CHCl_3$); **$^1$H NMR** (500 MHz, chloroform-*d*) δ 5.89 – 5.77 (m, 1H), 5.03 (dd, *J* = 17.1, 1.7 Hz, 1H), 4.95 (dd, *J* = 10.1, 1.3 Hz, 1H), 3.65 – 3.60 (m, 1H), 3.49 (dd, *J* = 10.5, 5.0 Hz, 1H), 3.45 (s, 3H), 3.43 (dd, *J* = 6.2, 4.0 Hz, 1H), 3.40 (s, 3H), 3.38 (s, 3H), 3.37 (s, 3H), 3.35 – 3.30 (m, 2H), 2.23 (m, 1H), 2.16 – 2.06 (m, 1H), 1.68 – 1.66 (m, 1H), 1.59 – 1.57 (m, 1H); **$^{13}$C NMR** (126 MHz, chloroform-*d*) δ 138.8, 114.6, 80.7, 80.3, 80.2, 71.5, 59.9, 59.1, 57.9, 57.7, 29.9, 29.0; **HRMS**: calcd for $C_{12}H_{24}O_4$([M + Na]$^+$): 255.1567, found: 255.1558.

*(5S,6R,7S)-5,6,7,8-tetramethoxy-oct-1-ene (**3ab**):*

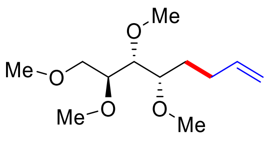


Following the **general procedure C**, the title compound was obtained from the reaction of hydrazone of **1ab** (0.24 mmol, 1.0 M generated *in situ* from **1ab** and hydrazine monohydrate) with allyl acetate (0.2 mmol). The product was further purified by column chromatography on silica gel. **3ab**: yield 88%, colorless oil, $R_f$ = 0.3 (hexane/EtOAc, 9:1); $[\alpha]_D^{22}$ = +7.6 (c = 1.0, $CHCl_3$); **$^1$H NMR** (500 MHz, chloroform-*d*) δ 5.83 (m, 1H), 5.04 (dd, *J* = 17.1, 1.8 Hz, 1H), 4.97 (dd, *J* = 10.2, 1.7 Hz, 1H), 3.69 (dd, *J* = 10.6, 2.4 Hz, 1H), 3.51 (m, 1H), 3.45 (s, 3H), 3.41 (m, 1H), 3.40 – 3.38 (m, 9H), 3.38 – 3.35 (m, 1H), 3.28 (dd, *J* = 7.8, 2.6 Hz, 1H), 2.19 – 2.11 (m, 2H), 1.76 (m, 1H), 1.64 (m, 1H); **$^{13}$C NMR** (126 MHz, chloroform-*d*) δ 138.4, 114.7, 80.7, 79.8, 79.6, 70.2, 60.8, 59.0, 58.5, 57.4, 30.1, 29.6; **HRMS**: calcd for $C_{12}H_{24}O_4$([M + Na]$^+$): 255.1567, found: 255.1562.

*(5S,6R,7R)-5,6,7,8-tetramethoxyoct-1-ene (**3ac**):*

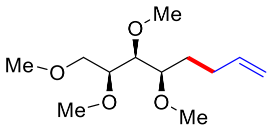

Following the **general procedure C**, the title compound was obtained from the reaction of hydrazone of **1ac** (0.24 mmol, 1.0 M generated *in situ* from **1ac** and hydrazine monohydrate) with allyl acetate (0.2 mmol). The product was further purified by column chromatography on silica gel. **3ac**: yield 80%, colorless oil, $R_f$ = 0.3 (hexane/EtOAc, 9:1); $[\alpha]_D^{22}$ = −8.5 (c = 1.0, $CHCl_3$); **$^1$H NMR** (500 MHz, chloroform-*d*) δ 5.80 (ddt, *J* = 16.9, 10.2, 6.7 Hz, 1H), 5.05 – 4.92 (m, 2H), 3.57 (dd, *J* = 5.5, 1.2 Hz, 1H), 3.50 (s, 3H), 3.48 – 3.45 (m, 2H), 3.43 (s, 3H), 3.38 (s, 3H), 3.35 (s, 3H), 3.33 – 3.30 (m, 1H), 3.29 – 3.26 (m, 1H), 2.18 – 2.06 (m, 2H), 1.74 – 1.63 (m, 1H), 1.62 – 1.51 (m, 1H); **$^{13}$C NMR** (126 MHz, chloroform-*d*) δ 138.4, 114.8, 81.9, 80.3, 80.2, 71.8, 60.7, 59.1, 58.6, 58.2, 29.9, 29.3; **HRMS**: calcd for $C_{12}H_{24}O_4$([M + Na]$^+$): 255.1567, found: 255.1567.

*(5S,6R,7S,8R)-5,6,7,8,9-pentamethoxynon-1-ene (**3ad**):*

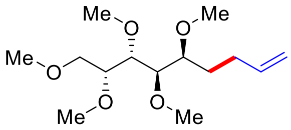

Following the **general procedure C**, the title compound was obtained from the reaction of hydrazone of **1ad** (0.24 mmol, 1.0 M generated *in situ* from **1ad** and hydrazine monohydrate) with allyl acetate (0.2 mmol). The product was further purified by column chromatography on silica gel. **3ad**: yield 78%, colorless oil, $R_f$ = 0.18 (hexane/EtOAc, 9:1); $[\alpha]_D^{22}$ = +25.6 (c = 0.75, $CHCl_3$); **$^1$H NMR** (500 MHz, chloroform-*d*) δ 5.85 (ddt, *J* = 16.8, 10.2, 6.5 Hz, 1H), 5.06 (dd, *J* = 17.2, 1.8 Hz, 1H), 4.98 (dd, *J* = 10.2, 1.7 Hz, 1H), 3.68 (dd, *J* = 8.9, 5.3 Hz, 1H), 3.61 (td, *J* = 5.7, 5.3, 1.9 Hz, 1H), 3.57 (dd, *J* = 8.9, 6.1 Hz, 1H), 3.49 (s, 3H), 3.45 (s, 3H), 3.45 (m, 1H), 3.43 (s, 3H), 3.40 (m, 1H), 3.39 (s, 3H), 3.38 (s, 3H), 3.36 (m, 1H), 2.22 – 2.08 (m, 2H), 1.85 (m, 1H), 1.72 (m, 1H); **$^{13}$C NMR** (126 MHz, chloroform-*d*) δ 138.2, 114.8, 80.1, 79.5, 79.4, 78.5, 72.0, 60.8, 60.5, 59.0, 58.1, 56.9, 30.1, 28.4; **HRMS**: calcd for $C_{14}H_{28}O_5$([M + Na]$^+$): 299.1829, found: 299.1821.

*(5S,6R,7R,8R)-5,6,7,8,9-pentamethoxynon-1-ene (**3ae**):*

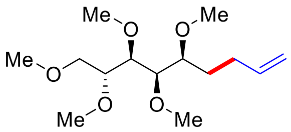

Following the **general procedure C**, the title compound was obtained from the reaction of hydrazone of **1ae** (0.24 mmol, 1.0 M generated *in situ* from **1ae** and hydrazine monohydrate) with allyl acetate (0.2 mmol). The product was further purified by column chromatography on silica gel. **3ae**: yield 65%, colorless oil, $R_f$ = 0.15 (hexane/EtOAc, 9:1); $[\alpha]_D^{22}$ = +11.4 (c = 1.0, $CHCl_3$); **$^1$H NMR** (500 MHz, chloroform-*d*) δ 5.83 (ddt, *J* = 16.9, 10.2, 6.6 Hz, 1H), 5.05 (dd, *J* = 17.1, 1.8 Hz, 1H), 4.97 (dd, *J* = 10.2, 2.0 Hz, 1H), 3.68 (dd, *J* = 10.6, 2.7 Hz, 1H), 3.53 (s, 3H), 3.50 (dd, *J* = 4.4, 3.4 Hz, 2H), 3.46 (s, 3H), 3.45 (s, 3H), 3.43 (s, 3H), 3.42 – 3.39 (m, 1H), 3.39 (s, 3H), 3.37 (d, *J* = 2.3 Hz, 1H), 3.36 – 3.33 (m, 1H), 2.27 – 2.09 (m, 2H), 1.71 (m, 1H), 1.60 – 1.49 (m, 1H); **$^{13}$C NMR** (126 MHz, chloroform-*d*) δ 138.5, 114.8, 82.6, 81.0, 79.9, 79.5, 69.9, 60.8, 60.3, 59.0, 59.0, 57.3, 30.1, 29.9; **HRMS**: calcd for $C_{14}H_{28}O_5$([M + Na]$^+$): 299.1829, found: 299.1835.

*(5R,6S,7R,8S)-5,6,7,8-tetramethoxynon-1-ene (**3af**):*

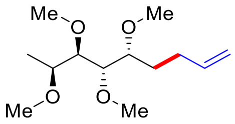


Following the **general procedure C**, the title compound was obtained from the reaction of hydrazone of **1af** (0.24 mmol, 1.0 M generated *in situ* from **1af** and hydrazine monohydrate) with allyl acetate (0.2 mmol). The product was further purified by column chromatography on silica gel. **3af**: yield 81%, colorless oil, $R_f$ = 0.3 (hexane/EtOAc, 9:1); $[\alpha]_D^{22}$ = -5.6 (c = 1.0, $CHCl_3$); **$^1$H NMR** (500 MHz, chloroform-*d*) δ 5.85 (ddt, *J* = 16.9, 10.2, 6.6 Hz, 1H), 5.05 (dd, *J* = 17.1, 1.7 Hz, 1H), 4.98 (dd, *J* = 10.1, 1.4 Hz, 1H), 3.58 (m, 1H), 3.47 (s, 3H), 3.45 (s, 3H), 3.38 (s, 3H), 3.37 – 3.35 (m, 1H), 3.34 (m, 1H), 3.34 (s, 3H), 3.20 (dd, *J* = 9.1, 1.7 Hz, 1H), 2.21 – 2.06 (m, 2H), 1.86 (m, 1H), 1.77 – 1.68 (m, 1H), 1.28 (d, *J* = 6.5 Hz, 3H); **$^{13}$C NMR** (126 MHz, chloroform-*d*) δ 138.2, 114.8, 83.0, 80.0, 79.3, 75.2, 61.2, 60.9, 56.7, 55.8, 30.1, 28.1, 15.0; **HRMS**: calcd for $C_{13}H_{26}O_4$ ([M + Na]$^+$): 269.1723, found: 269.1726.

*(4S,4'R,5R)-5-(but-3-en-1-yl)-2,2,2',2'-tetramethyl-4,4'-bi(1,3-dioxolane) (**3ag**):*

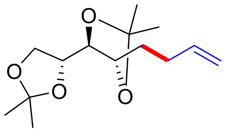


Following the **general procedure C**, the title compound was obtained from the reaction of hydrazone of **1ah** (0.24 mmol, 1.0 M generated *in situ* from **1ah** and hydrazine monohydrate) with allyl acetate (0.2 mmol). The resulting mixture was stirred at 45 °C for 3 hr. After the completion of the reaction, the reaction solution was filtered through a short celite pad and washed with diethyl

ether (60 mL). The combined solution was removed under vacuum, and the residue was purified by flash column chromatography to give **3ag**: yield 85%, colorless oil, $R_f$ = 0.55 (hexane/EtOAc, 9:1); $[\alpha]_D^{22}$ = -9.5 (c = 1.0, $CHCl_3$); **$^1$H NMR** (400 MHz, chloroform-*d*) δ 5.86 (ddt, *J* = 17.0, 10.2, 6.7 Hz, 1H), 5.05 (m, 1H), 4.98 (m, 1H), 4.22 – 4.14 (m, 1H), 4.09 (m, 2H), 3.92 (m, 2H), 2.28 (m, 1H), 2.17 (m, 1H), 1.79 (m, 1H), 1.68 (m, 1H), 1.39 (s, 3H), 1.38 (s, 3H). 1.33 (s, 3H), 1.32 (s, 3H); **$^{13}$C NMR** (126 MHz, chloroform-*d*) δ 138.3, 114.8, 109.6, 108.0, 78.8, 77.2, 73.5, 30.6, 28.6, 28.2, 26.8, 25.6, 25.6; **HRMS**: calcd for $C_{14}H_{24}O_4$ ([M + Na]$^+$): 279.1567, found: 279.1567.

*(4R,4'S,5S)-5-(but-3-en-1-yl)-2,2,2',2'-tetramethyl-4,4'-bi(1,3-dioxolane) (**3ah**):*

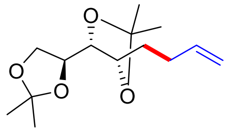

Following the **general procedure C**, the title compound was obtained from the reaction of hydrazone of **1ai** (0.24 mmol, 1.0 M generated *in situ* from **1ai** and hydrazine monohydrate) with allyl acetate (0.2 mmol). The resulting mixture was stirred at 45 °C for 3 hr. After the completion of the reaction, the reaction solution was filtered through a short celite pad and washed with diethyl ether (60 mL). The combined solution was removed under vacuum, and the residue was purified by flash column chromatography to give **3ah**: yield 88%, colorless oil, $R_f$ = 0.5 (hexane/EtOAc, 9:1); $[\alpha]_D^{22}$ = +8.4 (c = 1.0, $CHCl_3$); **$^1$H NMR** (500 MHz, chloroform-*d*) δ 5.85 (ddt, *J* = 17.0, 10.3, 6.6 Hz, 1H), 5.06 (dd, *J* = 17.1, 1.9 Hz, 1H), 4.98 (dd, *J* = 10.2, 1.6 Hz, 1H), 4.12 (dd, *J* = 8.4, 6.1 Hz, 1H), 4.02 (dt, *J* = 8.3, 5.6 Hz, 1H), 3.94 (ddd, *J* = 14.7, 8.2, 4.2 Hz, 2H), 3.57 (t, *J* = 7.8 Hz, 1H), 2.33 – 2.23 (m, 1H), 2.23 – 2.14 (m, 1H), 1.85 (dddd, *J* = 13.7, 9.9, 6.2, 3.3 Hz, 1H), 1.65 (dtd, *J* = 14.1, 9.2, 5.3 Hz, 1H), 1.41 (s, 3H), 1.39 (s, 3H), 1.35 (d, *J* = 4.1 Hz, 6H); **$^{13}$C NMR** (126 MHz, chloroform-*d*) δ 138.2, 114.7, 109.6, 108.8, 81.1, 79.9, 67.7, 32.9, 30.1, 27.4, 27.0, 26.7, 25.3; **HRMS**: calcd for $C_{14}H_{24}O_4$ ([M + Na]$^+$): 279.1567, found: 279.1568.

*(4S,5R)-4-(but-3-en-1-yl)-5-((S)-((R)-2,2-dimethyl-1,3-dioxolan-4-yl)(methoxy)methyl)-2,2-dimethyl-1,3-dioxolane (**3ai**):*

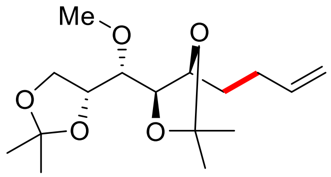

Following the **general procedure C**, the title compound was obtained from the reaction of hydrazone of **1aj** (0.24 mmol, 1.0 M generated *in situ* from **1aj** and hydrazine monohydrate) with

allyl acetate (0.2 mmol). The resulting mixture was stirred at 45 °C for 3 hr. After the completion of the reaction, the reaction solution was filtered through a short celite pad and washed with diethyl ether (60 mL). The combined solution was removed under vacuum, and the residue was purified by flash column chromatography to give **3ai**: yield 70%, colorless oil, $R_f$ = 0.3 (hexane/EtOAc, 5:1); $[\alpha]_D^{22}$ = +37.8 (c = 0.75, $CHCl_3$); **$^1$H NMR** (500 MHz, chloroform-*d*) δ 5.85 (ddt, *J* = 16.8, 10.2, 6.6 Hz, 1H), 5.06 (dd, *J* = 17.1, 1.8 Hz, 1H), 4.98 (dd, *J* = 10.3, 1.8 Hz, 1H), 4.16 (dd, *J* = 7.2, 2.5 Hz, 1H), 3.97 (ddd, *J* = 8.5, 7.1, 3.4 Hz, 1H), 3.70 – 3.64 (m, 2H), 3.61 (dd, *J* = 8.2, 7.1 Hz, 1H), 3.47 (dd, *J* = 10.3, 6.9 Hz, 1H), 3.41 (s, 3H), 2.27 (m, 1H), 2.22 – 2.17 (m, 1H), 1.84 (m, 1H), 1.65 – 1.61 (m, 1H), 1.42 (s, 3H), 1.39 (s, 3H), 1.37 (s, 3H), 1.34 (s, 3H); **$^{13}$C NMR** (126 MHz, chloroform-*d*) δ 138.2, 114.7, 109.9, 109.1, 81.7, 80.2, 79.8, 73.4, 59.4, 32.7, 30.1, 27.4, 27.1, 27.0, 26.9.

*(3aR,5R,5aS,8aS,8bR)-5-(but-3-en-1-yl)-2,2,7,7-tetramethyltetrahydro-5H-bis([1,3]dioxolo)[4,5-b:4',5'-d]pyran (**3aj**):*

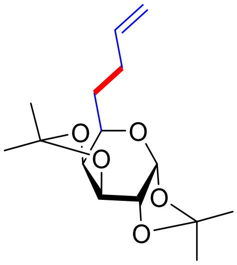


α-D-Galactopyranose

Following the **general procedure C**, the title compound was obtained from the reaction of hydrazone of **1ak** (0.24 mmol, 1.0 M generated *in situ* from **1ak** and hydrazine monohydrate) with allyl acetate (0.2 mmol). The product was further purified by column chromatography on silica gel. **3aj**: yield 84%, colorless oil, $R_f$ = 0.35 (hexane/EtOAc, 9:1); $[\alpha]_D^{21}$ = -31.1 (c = 0.5, $CHCl_3$);**$^1$H NMR** (500 MHz, chloroform-*d*) δ 5.82 (ddt, *J* = 17.0, 10.2, 6.6 Hz, 1H), 5.53 (d, *J* = 5.1 Hz, 1H), 5.06 (dd, *J* = 17.2, 1.8 Hz, 1H), 4.97 (dd, *J* = 10.2, 2.1 Hz, 1H), 4.58 (dd, *J* = 7.9, 2.4 Hz, 1H), 4.28 (dd, *J* = 5.1, 2.3 Hz, 1H), 4.11 (dd, *J* = 7.9, 1.9 Hz, 1H), 3.74 (ddd, *J* = 9.0, 4.6, 1.8 Hz, 1H), 2.28 – 2.19 (m, 1H), 2.19 – 2.09 (m, 1H), 1.80 (m, 1H), 1.62 (m, 1H), 1.50 (s, 3H), 1.46 (s, 3H), 1.34 (s, 3H), 1.32 (s, 3H); **$^{13}$C NMR** (126 MHz, chloroform-*d*) δ 138.1, 115.2, 109.0, 108.3, 96.6, 72.9, 71.0, 70.6, 66.6, 29.7, 29.3, 26.0, 25.0, 24.4; **HRMS**: calcd for $C_{15}H_{24}O_4$ ($[M + Na]^+$): 307.1516, found: 307.1515.

*(3aS,5aR,8aR,8bS)-3a-(but-3-en-1-yl)-2,2,7,7-tetramethyltetrahydro-5H-bis([1,3]dioxolo)[4,5-b:4',5'-d]pyran (**3ak**):*

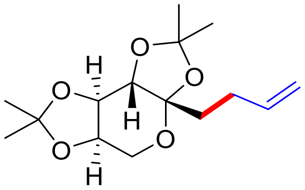


**D-Fructose**

Following the **general procedure C**, the title compound was obtained from the reaction of hydrazone of **1al** (0.24 mmol, 1.0 M generated *in situ* from **1al** and hydrazine monohydrate) with allyl acetate (0.2 mmol). The resulting mixture was stirred at 45 °C for 8 hr. After the completion of the reaction, the reaction solution was filtered through a short celite pad and washed with diethyl ether (60 mL). The combined solution was removed under vacuum, and the residue was purified by flash column chromatography to give **3ak**: yield 45%, colorless oil, $R_f$ = 0.43 (hexane/EtOAc, 9:1); $[\alpha]_D^{21}$ = -9.1 (c = 1.0, $CHCl_3$); **$^1$H NMR** (500 MHz, chloroform-*d*) δ 5.86 (ddt, *J* = 16.9, 10.2, 6.5 Hz, 1H), 5.03 (dd, *J* = 17.1, 1.8 Hz, 1H), 4.95 (dd, *J* = 10.2, 1.7 Hz, 1H), 4.56 (dd, *J* = 8.0, 2.4 Hz, 1H), 4.22 (dd, *J* = 8.0, 1.8 Hz, 1H), 4.11 (d, *J* = 2.4 Hz, 1H), 3.86 (dd, *J* = 13.1, 1.9 Hz, 1H), 3.73 (d, *J* = 13.1 Hz, 1H), 2.43 – 2.33 (m, 1H), 2.33 – 2.23 (m, 1H), 1.94 (m, 1H), 1.81 (m, 1H), 1.50 (d, *J* = 16.1 Hz, 6H), 1.35 (d, *J* = 2.8 Hz, 6H); **$^{13}$C NMR** (126 MHz, chloroform-*d*) δ 138.6, 114.2, 108.9, 107.4, 103.9, 73.7, 70.8, 70.6, 60.9, 40.1, 27.5, 26.4, 25.8, 25.2, 24.1;

*(3aR,4R,6R,6aR)-4-(but-3-en-1-yl)-6-methoxy-2,2-dimethyltetrahydrofuro[3,4-d][1,3]dioxole (**3al**):*

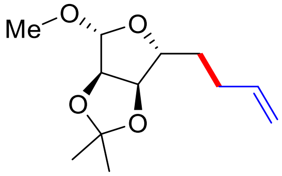


Following the **general procedure C**, the title compound was obtained from the reaction of hydrazone of **1am** (0.24 mmol, 1.0 M generated *in situ* from **1am** and hydrazine monohydrate) with allyl acetate (0.2 mmol). The product was further purified by column chromatography on silica gel. **3al**: yield 52%, colorless oil, $R_f$ = 0.4 (hexane/EtOAc, 20:1); $[\alpha]_D^{21}$ = -38.6 (c = 1.0, $CHCl_3$); **$^1$H NMR** (500 MHz, chloroform-*d*) δ 5.82 (ddt, *J* = 16.9, 10.2, 6.6 Hz, 1H), 5.05 (dd, *J* = 17.2, 1.7 Hz, 1H), 4.99 (dd, *J* = 10.2, 1.4 Hz, 1H), 4.94 (s, 1H), 4.60 (d, *J* = 5.9 Hz, 1H), 4.53 (dd,

*J* = 5.9, 1.0 Hz, 1H), 4.16 (ddd, *J* = 9.0, 6.4, 1.0 Hz, 1H), 3.34 (s, 3H), 2.26 – 2.11 (m, 2H), 1.74 – 1.66 (m, 1H), 1.63 – 1.55 (m, 1H), 1.48 (s, 3H), 1.31 (s, 3H); **$^{13}$C NMR** (126 MHz, chloroform-*d*) δ 137.6, 115.2, 112.2, 109.5, 86.6, 85.6, 84.2, 54.9, 34.3, 30.4, 26.5, 25.0; **HRMS**: calcd for $C_{12}H_{20}O_4$ ([M + Na]$^+$): 251.1254, found: 251.1250.

*(2S,3R,4S,5R)-non-8-ene-2,3,4,5-tetraol (**3am**):*

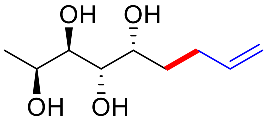


**L-Fucose**

PEPPSI$^{TM}$-IPr catalyst (6.8 mg, 5 mol%), allyl acetate **2** (0.2 mmol), and 1,4-dioxane (0.5 mL) were added into a dried microwave vial (10 mL) equipped with a stir bar in the glovebox. The reaction mixture was stirred at room temperature for 5 min before hydrazone **1an** (42.8 mg, 0.24 mmol), 3Å MS (100 mg), NaOH (48 mg, 1.2 mmol), and 1,4-dioxane (1.5 mL) were added. The reaction tube was sealed and moved out of the glovebox. The resulting mixture was stirred at room temperature for 24 hr. After the completion of the reaction, the reaction solution was added 1.0 mL of water and stirred for 1 hr. The solution was then filtered through a short celite pad and washed with DCM/MeOH (1:2, 60 mL). The combined solution was concentrated under vacuum, and the residue was purified by flash column chromatography to give the desired product. **3am**: yield 55%, white solid, $R_f$ = 0.4 (DCM/MeOH, 10:1); $[\alpha]_D^{21}$ = +13.1 (c = 0.5, MeOH); **$^1$H NMR** (500 MHz, methanol-*d*$_4$) δ 5.88 (ddt, *J* = 17.0, 10.4, 6.7 Hz, 1H), 5.05 (d, *J* = 17.2 Hz, 1H), 4.95 (d, *J* = 10.2 Hz, 1H), 4.05 (q, *J* = 6.4, 5.8 Hz, 1H), 3.86 (dd, *J* = 9.0, 4.8 Hz, 1H), 3.48 (d, *J* = 8.8 Hz, 1H), 3.42 (d, *J* = 9.6 Hz, 1H), 2.25 (m, 1H), 2.14 (m, 1H), 1.72 (m, 1H), 1.61 (m, 1H), 1.24 (d, *J* = 6.6 Hz, 3H); **$^{13}$C NMR** (101 MHz, methanol-*d*$_4$) δ 138.5, 113.6, 73.5, 72.4, 69.7, 66.4, 32.8, 30.0, 18.5; **HRMS**: calcd for $C_9H_{18}O_4$ ([M + Na]$^+$): 213.1097, found: 213.1095.

*(2R,3S,4R)-oct-7-ene-1,2,3,4-tetraol (**3an**):*

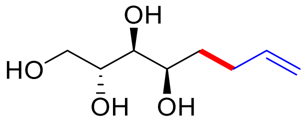


**D-Arabinose**

PEPPSI$^{TM}$-IPr catalyst (6.8 mg, 5 mol%), allyl acetate **2** (0.2 mmol), and 1,4-dioxane (0.5 mL) were added into a dried microwave vial (10 mL) equipped with a stir bar in the glovebox. The

reaction mixture was stirred at room temperature for 5 min before hydrazone **1ao** (39.5 mg, 0.24 mmol), 3Å MS (100 mg), NaOH (48 mg, 1.2 mmol), and 1,4-dioxane (1.5 mL) were added. The reaction tube was sealed and moved out of the glovebox. The resulting mixture was stirred at room temperature for 24 hr. After the completion of the reaction, the reaction solution was added 1.0 mL of water and stirred for 1 hr. The solution was then filtered through a short celite pad and washed with DCM/MeOH (1:2, 60 mL). The combined solution was concentrated under vacuum, and the residue was purified by flash column chromatography to give the desired product. **3an**: yield 41%, white solid, $R_f$ = 0.36 (DCM/MeOH, 6:1); $[\alpha]_D^{21}$ = +62.4 (c = 0.5, MeOH); $^1$**H NMR** (500 MHz, methanol-$d_4$) δ 5.87 (ddt, *J* = 17.0, 10.3, 6.7 Hz, 1H), 5.04 (dd, *J* = 17.1, 1.8 Hz, 1H), 4.98 – 4.92 (dd, *J* = 10.1, 1.9 Hz, 1H), 3.86 – 3.80 (m, 1H), 3.78 (dd, *J* = 11.1, 3.7 Hz, 1H), 3.70 – 3.64 (m, 1H), 3.61 (dd, *J* = 11.1, 6.0 Hz, 1H), 3.36 (dd, *J* = 7.9, 1.9 Hz, 1H), 2.29 – 2.18 (m, 1H), 2.18 – 2.07 (m, 1H), 1.76 – 1.64 (m, 1H), 1.59 (m, 1H); $^{13}$**C NMR** (126 MHz, methanol-$d_4$) δ 138.4, 113.6, 73.2, 71.8, 69.5, 63.7, 32.7, 29.9; **HRMS**: calcd for $C_8H_{16}O_4$ ($[M + Na]^+$): 199.0941, found: 199.0937.

*(2S,3R,4S)-oct-7-ene-1,2,3,4-tetraol (**3ao**):*

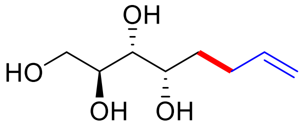


**L-Arabinose**

PEPPSI™-IPr catalyst (6.8 mg, 5 mol%), allyl acetate **2** (0.2 mmol), and 1,4-dioxane (0.5 mL) were added into a dried microwave vial (10 mL) equipped with a stir bar in the glovebox. The reaction mixture was stirred at room temperature for 5 min before hydrazone **1ap** (39.5 mg, 0.24 mmol), 3Å MS (100 mg), NaOH (48 mg, 1.2 mmol), and 1,4-dioxane (1.5 mL) were added. The reaction tube was sealed and moved out of the glovebox. The resulting mixture was stirred at room temperature for 24 hr. After the completion of the reaction, the reaction solution was added 1.0 mL of water and stirred for 1hr. The solution was then filtered through a short celite pad and washed with DCM/MeOH (1:2, 60 mL). The combined solution was removed under vacuum, and the residue was purified by flash column chromatography to give the desired product. **3ao**: yield 42%, white solid, $R_f$ = 0.36 (DCM/MeOH, 6:1); $[\alpha]_D^{21}$ = -46.7 (c = 0.5, MeOH); $^1$**H NMR** (500 MHz, methanol-$d_4$) δ 5.87 (ddt, *J* = 16.9, 10.3, 6.6 Hz, 1H), 5.04 (dd, *J* = 17.1, 1.9 Hz, 1H), 4.95 (dd, *J* = 10.2, 2.0 Hz, 1H), 3.86 – 3.80 (m, 1H), 3.78 (dd, *J* = 11.1, 3.6 Hz, 1H), 3.68 (m, 1H), 3.61

(dd, $J$ = 11.1, 6.0 Hz, 1H), 3.36 (dd, $J$ = 7.9, 2.0 Hz, 1H), 2.29 – 2.19 (m, 1H), 2.13 (m, 1H), 1.77 – 1.65 (m, 1H), 1.59 (m, 1H); $^{13}$**C NMR** (126 MHz, methanol-$d_4$) δ 138.4, 113.6, 73.2, 71.8, 69.5, 63.7, 32.7, 29.9; **HRMS**: calcd for $C_8H_{16}O_4$ ([M + Na]$^+$): 199.0941, found: 199.0937.

*(2R,3S, E)-1,3-bis(benzyloxy)octa-4,7-dien-2-ol (**3as**):*

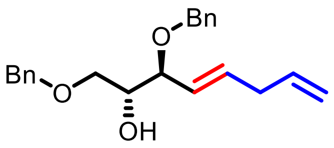


**D-Ribose**

Following the **general procedure C**, the title compound was obtained from the reaction of hydrazone of 2,3,5-Tri-O-benzyl-D-ribose (0.24 mmol, 1.0 M generated *in situ* from 2,3,5-Tri-O-benzyl-D-ribose and hydrazine monohydrate) with allyl acetate (0.2 mmol). The resulting mixture was stirred at room temperature for 12 hr. After the completion of the reaction, the reaction solution was filtered through a short celite pad and washed with diethyl ether (60 mL). The combined solution was removed under vacuum, and the residue was purified by flash column chromatography to give **3as**: yield 63%, colorless oil, $R_f$ = 0.32 (hexane/EtOAc, 9:1); E/Z > 20:1 (The E/Z ratio was determined based on analysis of the $^1$H-NMR spectrum); $[\alpha]_D^{20}$ = 9.5(c = 1.0, $CHCl_3$); $^1$**H NMR** (400 Hz, chloroform-*d*) δ 7.38 – 7.27 (m, 10H), 5.91 – 5.70 (m, 2H), 5.51 (dd, J = 15.5, 9.5 Hz, 1H), 5.13 – 4.97 (m, 2H), 4.61 (d, J = 11.9 Hz, 1H), 4.53 (d, J = 3.0 Hz, 2H), 4.36 (d, J = 11.8 Hz, 1H), 3.91 (s, 1H), 3.85 (dd, J = 8.3, 5.4 Hz, 1H), 3.63 – 3.52 (m, 2H), 2.86 (td, J = 6.4, 1.6 Hz, 2H), 2.37 (d, J = 4.2 Hz, 1H). $^{13}$**C NMR** (101 MHz, Chloroform-d) δ 138.3, 138.1, 136.2, 134.5, 128.4, 128.4, 127.8, 127.7, 127.7, 127.6, 115.8, 80.4, 73.4, 72.4, 70.9, 70.1, 36.5.

*(5S,6S,7R, E)-5,6,7,8-tetramethoxyocta-1,3-diene (**4aa**):*

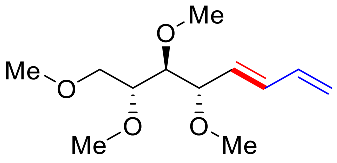


**D-Ribose**

Following the **general procedure D**, the title compound was obtained from the reaction of hydrazone of **1aa** (0.20 mmol, 1.0 M generated *in situ* from **1aa** and hydrazine monohydrate) with allyl acetate (0.6 mmol). The product was further purified by column chromatography on silica gel. **4aa**: yield 60%, colorless oil, $R_f$ = 0.3 (hexane/EtOAc, 9:1); E/Z > 11:1 (The E/Z ratio was determined based on analysis of the $^1$H-NMR spectrum); $[\alpha]_D^{21}$ = -59.9 (c = 0.5, $CHCl_3$); $^1$**H NMR**

(500 MHz, chloroform-*d*) δ 6.38 (dt, *J* = 16.9, 10.3 Hz, 1H), 6.23 (dd, *J* = 15.4, 10.5 Hz, 1H), 5.69 (dd, *J* = 15.4, 8.4 Hz, 1H), 5.21 (d, *J* = 17.0 Hz, 1H), 5.09 (d, *J* = 10.1 Hz, 1H), 3.82 (dd, *J* = 8.5, 3.9 Hz, 1H), 3.63 (d, *J* = 10.4 Hz, 1H), 3.52 – 3.48 (m, 1H), 3.46 (d, *J* = 1.3 Hz, 3H), 3.45 (d, *J* = 1.1 Hz, 1H), 3.37 (d, *J* = 3.0 Hz, 6H), 3.27 (s, 3H), 3.26 – 3.21 (m, 1H); **$^{13}$C NMR** (126 MHz, chloroform-*d*) δ 136.4, 135.1, 130.5, 117.5, 82.7, 81.9, 80.1, 71.0, 60.3, 59.1, 57.7, 56.6; **HRMS**: calcd for $C_{12}H_{22}O_4$ ([M + Na]$^+$): 253.1410, found: 253.1411.

**(***5S,6R,7S, E)-5,6,7,8-tetramethoxyocta-1,3-diene (**4ab**):*

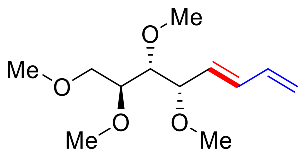


**L-Arabinose**

Following the **general procedure D**, the title compound was obtained from the reaction of hydrazone of **1ab** (0.20 mmol, 1.0 M generated *in situ* from **1ab** and hydrazine monohydrate) with allyl acetate (0.6 mmol). The product was further purified by column chromatography on silica gel. **4ab**: yield 58%, colorless oil, $R_f$ = 0.3 (hexane/EtOAc, 9:1); E/Z > 11:1 (The E/Z ratio was determined based on analysis of the $^1$H-NMR spectrum); $[\alpha]_D^{22}$ = +9.1 (c = 1.0, $CHCl_3$); **$^1$H NMR** (500 MHz, chloroform-*d*) δ 6.42 (dt, *J* = 16.7, 10.2 Hz, 1H), 6.32 (dd, *J* = 15.4, 10.3 Hz, 1H), 5.76 (dd, *J* = 15.2, 7.9 Hz, 1H), 5.26 (dd, *J* = 16.6, 1.6 Hz, 1H), 5.14 (dd, *J* = 10.0, 1.6 Hz, 1H), 3.87 (dd, *J* = 8.0, 3.0 Hz, 1H), 3.69 (dd, *J* = 10.4, 2.4 Hz, 1H), 3.52 (dd, *J* = 10.4, 3.9 Hz, 1H), 3.50 – 3.45 (m, 1H), 3.45 (s, 3H), 3.44 (s, 3H), 3.41 (s, 3H), 3.32 (s, 3H), 3.30 (dd, *J* = 7.6, 2.8 Hz, 1H); **$^{13}$C NMR** (126 MHz, chloroform-*d*) δ 136.3, 133.8, 131.5, 117.6, 82.4, 80.9, 79.4, 70.5, 61.0, 59.1, 57.9, 56.8; **HRMS**: calcd for $C_{12}H_{22}O_4$ ([M + Na]$^+$): 253.1410, found: 253.1411.

*(5R,6S,7R,8S, E)-5,6,7,8-tetramethoxynona-1,3-diene (**4ac**):*

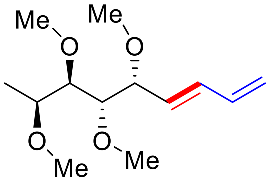


**L-Fucose**

Following the **general procedure D**, the title compound was obtained from the reaction of hydrazone of **1af** (0.20 mmol, 1.0 M generated *in situ* from **1af** and hydrazine monohydrate) with allyl acetate (0.6 mmol). The product was further purified by column chromatography on silica

gel. **4ac**: yield 61%, colorless oil, $R_f$ = 0.3 (hexane/EtOAc, 9:1); E/Z > 8:1 (The E/Z ratio was determined based on analysis of the $^1$H-NMR spectrum); $[\alpha]_D^{22}$ = -16.9 (c = 1.0, $CHCl_3$); **$^1$H NMR** (500 MHz, chloroform-*d*) δ 6.41 (dt, *J* = 16.5, 10.1 Hz, 1H), 6.31 (dd, *J* = 15.4, 10.5 Hz, 1H), 5.81 (dd, *J* = 15.3, 8.2 Hz, 1H), 5.24 (d, *J* = 17.4 Hz, 1H), 5.12 (d, *J* = 9.2 Hz, 1H), 3.85 (d, *J* = 8.9 Hz, 1H), 3.59 – 3.53 (m, 1H), 3.48 (s, 3H), 3.40 (s, 3H), 3.33 (s, 3H), 3.31 (dd, *J* = 1.8, 0.6 Hz, 1H), 3.29 (s, 3H), 3.24 – 3.20 (m, 1H), 1.27 (d, *J* = 6.4 Hz, 3H); **$^{13}$C NMR** (126 MHz, chloroform-*d*) δ 136.4, 133.9, 132.1, 117.5, 82.8, 82.6, 80.6, 75.0, 61.3, 61.1, 56.1, 55.9, 14.9; **HRMS**: calcd for $C_{13}H_{24}O_4$ ([M + Na]$^+$): 267.1567, found: 267.1557.

*(5S,6R,7R, E)-5,6,7,8-tetramethoxyocta-1,3-diene (**4ad**):*

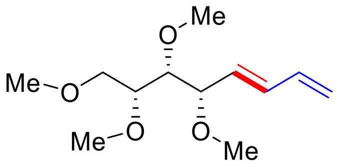


**D-Xylose**

Following the **general procedure D**, the title compound was obtained from the reaction of hydrazone of **1ac** (0.20 mmol, 1.0 M generated *in situ* from **1ac** and hydrazine monohydrate) with allyl acetate (0.6 mmol). The product was further purified by column chromatography on silica gel. **4ad**: yield 51%, colorless oil, $R_f$ = 0.3 (hexane/EtOAc, 9:1); E/Z > 10:1 (The E/Z ratio was determined based on analysis of the $^1$H-NMR spectrum); $[\alpha]_D^{22}$ = +10.9 (c = 1.0, $CHCl_3$); **$^1$H NMR** (500 MHz, chloroform-*d*) δ 6.36 (dt, *J* = 16.8, 10.2 Hz, 1H), 6.29 – 6.22 (m, 1H), 5.63 (dd, *J* = 15.3, 8.1 Hz, 1H), 5.23 (dd, *J* = 16.8, 1.6 Hz, 1H), 5.11 (dd, *J* = 9.9, 1.6 Hz, 1H), 3.83 – 3.78 (m, 1H), 3.55 (dd, *J* = 9.9, 4.8 Hz, 1H), 3.51 (s, 3H), 3.48 (t, *J* = 4.9 Hz, 1H), 3.44 – 3.41 (m, 1H), 3.39 (s, 3H), 3.36 (s, 3H), 3.27 (s, 3H), 3.26 – 3.24 (m, 1H); **$^{13}$C NMR** (126 MHz, chloroform-*d*) δ 136.3, 134.3, 130.9, 117.8, 83.3, 82.6, 80.1, 71.8, 61.1, 59.1, 58.7, 56.7; **HRMS**: calcd for $C_{12}H_{22}O_4$ ([M + Na]$^+$): 253.1410, found: 253.1410.

*(4S,4'R,5R)-5-((E)-buta-1,3-dien-1-yl)-2,2,2',2'-tetramethyl-4,4'-bi(1,3-dioxolane) (**4ae**):*

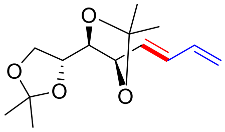


**D-Arabinose**

Following the **general procedure D**, the title compound was obtained from the reaction of hydrazone (0.20 mmol, 1.0 M generated *in situ* from the acetonide protected D-Arabinose and

hydrazine monohydrate) with allyl acetate (0.6 mmol). The product was further purified by column chromatography on silica gel. **4ae**: yield 52%, colorless oil, $R_f$ = 0.5 (hexane/EtOAc, 9:1); E/Z > 8:1 (The E/Z ratio was determined based on analysis of the $^1$H-NMR spectrum); $[\alpha]_D^{21}$ = -32.8 (c = 0.5, $CHCl_3$); **$^1$H NMR** (500 MHz, chloroform-*d*) δ 6.43 – 6.29 (m, 2H), 5.74 (dd, *J* = 14.5, 6.8 Hz, 1H), 5.24 (d, *J* = 16.3 Hz, 1H), 5.12 (d, *J* = 9.8 Hz, 1H), 4.41 (t, *J* = 7.0 Hz, 1H), 4.14 (td, *J* = 6.6, 4.8 Hz, 1H), 4.09 (dd, *J* = 8.3, 6.3 Hz, 1H), 3.95 (dd, *J* = 8.4, 4.7 Hz, 1H), 3.74 – 3.68 (m, 1H), 1.42 (s, 3H), 1.41 (s, 3H), 1.39 (s, 3H), 1.34 (s, 3H); **$^{13}$C NMR** (126 MHz, chloroform-*d*) δ 136.1, 133.2, 130.8, 118.2, 109.7, 109.5, 81.2, 79.7, 67.0, 27.0, 26.9, 26.7, 25.3; **HRMS**: calcd for $C_{14}H_{22}O_4$ ($[M + Na]^+$): 277.1410, found: 277.1407.

*(4R,4'S,5S)-5-((E)-buta-1,3-dien-1-yl)-2,2,2',2'-tetramethyl-4,4'-bi(1,3-dioxolane) (**4af**):*

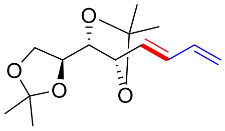

**L-Arabinose**

Following the **general procedure D**, the title compound was obtained from the reaction of hydrazone of **1ai** (0.20 mmol, 1.0 M generated *in situ* from **1ai** and hydrazine monohydrate) with allyl acetate (0.6 mmol). The product was further purified by column chromatography on silica gel. **4af**: yield 50%, colorless oil, $R_f$ = 0.5 (hexane/EtOAc, 9:1); E/Z > 12:1 (The E/Z ratio was determined based on analysis of the $^1$H-NMR spectrum); $[\alpha]_D^{21}$ = +23.3 (c = 1.0, $CHCl_3$); **$^1$H NMR** (500 MHz, chloroform-*d*) δ 6.43 – 6.29 (m, 2H), 5.73 (dd, *J* = 14.4, 6.7 Hz, 1H), 5.24 (d, *J* = 16.3 Hz, 1H), 5.12 (d, *J* = 10.0 Hz, 1H), 4.45 – 4.36 (m, 1H), 4.16 – 4.11 (m, 1H), 4.11 – 4.06 (m, 1H), 3.95 (dd, *J* = 8.3, 4.7 Hz, 1H), 3.71 (t, *J* = 7.3 Hz, 1H), 1.42 (s, 3H), 1.41 (s, 3H), 1.39 (s, 3H), 1.34 (s, 3H); **$^{13}$C NMR** (126 MHz, chloroform-*d*) δ 136.1, 133.2, 130.8, 118.2, 109.67, 109.5, 81.2, 79.7, 76.7, 67.0, 27.0, 26.9, 26.7, 25.3; **HRMS**: calcd for $C_{14}H_{22}O_4$ ($[M + Na]^+$): 277.1410, found: 277.1400.

*(3aS,4S,8aR)-4-((E)-buta-1,3-dien-1-yl)-2,2,6,6-tetramethyltetrahydro-[1,3]dioxolo[4,5-e][1,3]dioxepine (**4ag**):*

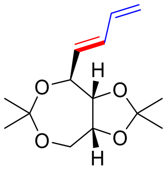

**D-Ribose**

Following the **general procedure D**, the title compound was obtained from the reaction of hydrazone (0.20 mmol, 1.0 M generated *in situ* from the acetonide protected D-Ribose and hydrazine monohydrate) with allyl acetate (0.6 mmol). the reaction was stirred at 60 °C for 18 hr and isolated by column chromatography on silica gel. **4ag**: yield 54%, colorless oil, $R_f$ = 0.45 (hexane/EtOAc, 9:1); E/Z > 20:1 (The E/Z ratio was determined based on analysis of the $^1$H-NMR spectrum); $[\alpha]^{21}_{D}$ = -7.8 (c = 0.5, $CHCl_3$); **$^1$H NMR** (500 MHz, chloroform-*d*) δ 6.43 – 6.23 (m, 2H), 5.84 (dd, *J* = 15.0, 5.0 Hz, 1H), 5.21 (dd, *J* = 16.7, 1.7 Hz, 1H), 5.08 (dd, *J* = 9.8, 1.6 Hz, 1H), 4.31 (dd, *J* = 9.7, 5.3 Hz, 1H), 4.12 (dt, *J* = 5.4, 1.8 Hz, 1H), 4.03 – 3.94 (m, 2H), 3.78 (dd, *J* = 9.8, 5.4 Hz, 1H), 1.53 (s, 3H), 1.40 (s, 3H), 1.37 (s, 3H), 1.36 (s, 3H); **$^{13}$C NMR** (126 MHz, chloroform-*d*) δ 136.6, 131.7, 117.2, 108.5, 101.7, 79.3, 69.4, 58.5, 28.4, 25.7, 24.7, 23.7; **HRMS**: calcd for $C_{14}H_{22}O_4$ ($[M + Na]^+$): 277.1410, found: 277.1397.

*(S, E)-4-(buta-1,3-dien-1-yl)-2,2-dimethyl-1,3-dioxolane (**4ah**):*

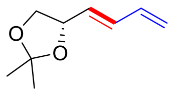

**D-Glyceraldehyde**

Following the **general procedure D**, the title compound was obtained from the reaction of hydrazone of **1ag** (0.20 mmol, 1.0 M generated *in situ* from **1ag** and hydrazine monohydrate) with allyl acetate (0.6 mmol). The product was further purified by column chromatography on silica gel. **4ah**: yield 51%, colorless oil, $R_f$ = 0.8 (hexane/EtOAc, 9:1); E/Z > 7:1 (The E/Z ratio was determined based on analysis of the $^1$H-NMR spectrum); **$^1$H NMR** (500 MHz, chloroform-*d*) δ 6.39 – 6.26 (m, 2H), 5.66 (dd, *J* = 14.1, 7.6 Hz, 1H), 5.24 (dd, *J* = 16.4, 1.0 Hz, 1H), 5.13 (dd, *J* = 9.0, 1.9 Hz, 1H), 4.54 (q, *J* = 7.5 Hz, 1H), 4.10 (dd, *J* = 8.1, 6.1 Hz, 1H), 3.60 (t, *J* = 8.0 Hz, 1H), 1.43 (s, 3H), 1.39 (s, 3H); **$^{13}$C NMR** (126 MHz, chloroform-*d*) δ 135.9, 134.0, 130.60, 118.5, 109.4, 76.7, 69.4, 26.7, 25.9.

*(3aR,5R,5aS,8aS,8bR)-5-((E)-buta-1,3-dien-1-yl)-2,2,7,7-tetramethyltetrahydro-5H-bis([1,3]dioxolo)[4,5-b:4',5'-d]pyran (**4ai**):*

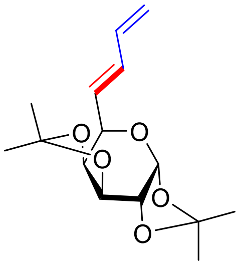

α-D-Galactopyranose

Following the **general procedure D**, the title compound was obtained from the reaction of hydrazone of **1ak** (0.20 mmol, 1.0 M generated *in situ* from **1ak** and hydrazine monohydrate) with allyl acetate (0.6 mmol). The reaction was stirred at 60 °C for 18 hr and isolated by column chromatography on silica gel. **4ai**: yield 50%, colorless oil, $R_f$ = 0.35 (hexane/EtOAc, 9:1); E/Z > 8:1 (The E/Z ratio was determined based on analysis of the $^1$H-NMR spectrum); $[\alpha]_D^{22}$ = -138.4 (c = 1.0, $CHCl_3$); **$^1$H NMR** (500 MHz, chloroform-*d*) δ 6.42 – 6.25 (m, 2H), 5.79 (dd, *J* = 14.5, 6.7 Hz, 1H), 5.21 (dd, *J* = 15.6, 2.2 Hz, 1H), 5.13 – 5.06 (m, 1H), 4.60 (dd, *J* = 7.9, 2.4 Hz, 1H), 4.31 (td, *J* = 5.8, 5.2, 2.2 Hz, 2H), 4.20 (dd, *J* = 7.8, 2.0 Hz, 1H), 1.53 (s, 3H), 1.46 (s, 3H), 1.33 (s, 6H); **$^{13}$C NMR** (126 MHz, chloroform-*d*) δ 136.4, 133.6, 128.9, 117.9, 109.3, 108.5, 96.5, 73.5, 70.9, 70.4, 68.5, 26.2, 26.0, 24.9, 24.3.

*(3aS,5aR,8aR,8bS)-3a-((E)-buta-1,3-dien-1-yl)-2,2,7,7-tetramethyltetrahydro-5H-bis([1,3]dioxolo)[4,5-b:4',5'-d]pyran (**4aj**):*

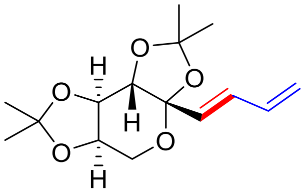

**D-Fructose**

Following the **general procedure D**, the title compound was obtained from the reaction of hydrazone of **1al** (0.20 mmol, 1.0 M generated *in situ* from **1al** and hydrazine monohydrate) with allyl acetate (0.6 mmol). The reaction was stirred at 45 °C for 18 hr and isolated by column chromatography on silica gel. **4aj**: yield 50%, colorless oil, $R_f$ = 0.43 (hexane/EtOAc, 9:1); E/Z > 20:1 (The E/Z ratio was determined based on analysis of the $^1$H-NMR spectrum); $[\alpha]_D^{22}$ = -30.2 (c = 1.0, $CHCl_3$); **$^1$H NMR** (500 MHz, chloroform-*d*) δ 6.57 (dd, *J* = 15.3, 10.7 Hz, 1H), 6.37 (dt, *J* = 17.0, 10.4 Hz, 1H), 5.78 (d, *J* = 15.2 Hz, 1H), 5.29 (d, *J* = 16.9 Hz, 1H), 5.17 (d, *J* = 10.1 Hz, 1H), 4.61 (dd, *J* = 7.9, 2.5 Hz, 1H), 4.25 (d, *J* = 1.8 Hz, 1H), 4.20 (d, *J* = 2.4 Hz, 1H), 3.92 (dd, *J*

= 13.0, 1.9 Hz, 1H), 3.79 (d, *J* = 12.9 Hz, 1H), 1.57 (s, 3H), 1.50 (s, 3H), 1.39 (s, 3H), 1.36 (s, 3H); **$^{13}$C NMR** (126 MHz, chloroform-*d*) δ 135.9, 132.8, 131.9, 119.2, 109.1, 108.2, 102.1, 74.0, 70.6, 70.4, 61.1, 26.2, 25.9, 24.9, 24.2.

*(3aR,4R,6R,6aR)-4-((E)-buta-1,3-dien-1-yl)-6-methoxy-2,2-dimethyltetrahydrofuro[3,4-d][1,3]dioxole (**4ak**):*

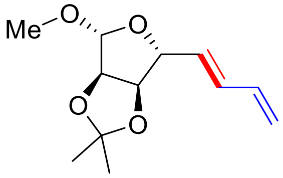


**D-Ribose**

Following the **general procedure D**, the title compound was obtained from the reaction of hydrazone of **1am** (0.20 mmol, 1.0 M generated *in situ* from **1am** and hydrazine monohydrate) with allyl acetate (0.6 mmol). The reaction was stirred at 45 °C for 18 hr and isolated by column chromatography on silica gel. **4ak**: yield 37%, colorless oil, $R_f$ = 0.4 (hexane/EtOAc, 20:1); E/Z > 8:1 (The E/Z ratio was determined based on analysis of the $^1$H-NMR spectrum); $[\alpha]_D^{21}$ = -14.4 (c = 0.5, $CHCl_3$); **$^1$H NMR** (500 MHz, chloroform-*d*) δ 6.36 – 6.17 (m, 2H), 5.71 (dd, *J* = 14.5, 8.7 Hz, 1H), 5.24 (d, *J* = 15.8 Hz, 1H), 5.13 (d, *J* = 9.3 Hz, 1H), 4.98 (s, 1H), 4.66 (d, *J* = 8.6 Hz, 1H), 4.62 (s, 2H), 3.34 (s, 3H), 1.49 (s, 3H), 1.31 (s, 3H); **$^{13}$C NMR** (126 MHz, chloroform-*d*) δ 136.0, 133.4, 132.6, 118.4, 109.3, 87.7, 85.6, 84.7, 54.6, 26.5, 25.0.

*(2S,3R,4S, E)-octa-5,7-diene-1,2,3,4-tetraol (**4al**):*

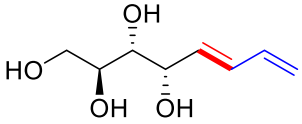


**L-Arabinose**

$[Pd(allyl)Cl]_2$ (1.9 mg, 2.5 mol%), tris(4-trifluoromethylphenyl)phosphine (9.4 mg, 10 mol%), allyl acetate **2** (0.6 mmol), and 1,4-dioxane (0.5 mL) were added into a dried microwave vial (10 mL) equipped with a stir bar in the glovebox. The reaction mixture was stirred at room temperature for 5 min before hydrazone **1ap** (33 mg, 0.2 mmol), 3Å MS (100 mg), NaOH (48 mg, 1.2 mmol), and 1,4-dioxane (1.5 mL) were added. The reaction tube was sealed and moved out of the glovebox. The resulting mixture was stirred at 60 °C for 24 hr. After the completion of the reaction, to the reaction solution was added 1.0 mL of water and stirred for 1 hr. The solution was then filtered through a short celite pad and washed with DCM/MeOH (1:2, 60 mL). The combined solution was

concentrated under vacuum, and the residue was purified by flash column chromatography to give the desired product. **4al**: yield 68%, white solid, $R_f$ = 0.36 (DCM/MeOH, 6:1); E/Z > 13:1 (The E/Z ratio was determined based on analysis of the $^1$H-NMR spectrum); $[\alpha]_D^{21}$ = -27.2 (c = 0.5, MeOH); **$^1$H NMR** (500 MHz, methanol-*d*$_4$) δ 6.45 – 6.28 (m, 2H), 5.87 (dd, *J* = 14.9, 6.4 Hz, 1H), 5.20 (dd, *J* = 16.7, 1.9 Hz, 1H), 5.06 (dd, *J* = 9.8, 1.7 Hz, 1H), 4.41 – 4.33 (m, 1H), 3.77 (dd, *J* = 11.1, 3.5 Hz, 1H), 3.69 (ddd, *J* = 7.6, 5.9, 3.4 Hz, 1H), 3.62 (dd, *J* = 11.1, 6.0 Hz, 1H), 3.46 (dd, *J* = 7.7, 2.8 Hz, 1H); **$^{13}$C NMR** (126 MHz, methanol-*d*$_4$) δ 136.7, 134.1, 131.5, 115.8, 74.1, 71.7, 71.1, 63.4; **HRMS**: calcd for $C_8H_{14}O_4$ ([M + Na]$^+$): 197.0784, found: 197.0778.

*(2S,3R,4S,5R, E)-nona-6,8-diene-2,3,4,5-tetraol (**4am**):*

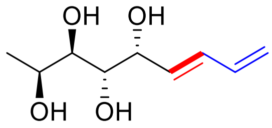


**L-Fucose**

$[Pd(allyl)Cl]_2$ (1.9 mg, 2.5 mol%), tris(4-trifluoromethylphenyl)phosphine (9.4 mg, 10 mol%), allyl acetate **2** (0.6 mmol), and 1,4-dioxane (0.5 mL) were added into a dried microwave vial (10 mL) equipped with a stir bar in the glovebox. The reaction mixture was stirred at room temperature for 5 min before hydrazone **1an** (36 mg, 0.2 mmol), 3Å MS (100 mg), NaOH (48 mg, 1.2 mmol), and 1,4-dioxane (1.5 mL) were added. The reaction tube was sealed and moved out of the glovebox. The resulting mixture was stirred at 60 °C for 24 hr. After the completion of the reaction, to the reaction solution was added 1.0 mL of water and stirred for 1 hr. The solution was then filtered through a short celite pad and washed with DCM/MeOH (1:2, 60 mL). The combined solution was concentrated under vacuum, and the residue was purified by flash column chromatography to give the desired product. **4am**: yield 70%, white solid, $R_f$ = 0.4 (DCM/MeOH, 10:1); E/Z > 14:1 (The E/Z ratio was determined based on analysis of the $^1$H-NMR spectrum); $[\alpha]_D^{21}$ = +11.6 (c = 0.5, MeOH); **$^1$H NMR** (500 MHz, methanol-*d*$_4$) δ 6.46 – 6.26 (m, 2H), 5.89 (dd, *J* = 15.1, 6.3 Hz, 1H), 5.19 (dd, *J* = 16.2, 1.7 Hz, 1H), 5.05 (dd, *J* = 9.6, 1.8 Hz, 1H), 4.41 (d, *J* = 6.4 Hz, 1H), 4.04 (qd, *J* = 6.5, 2.0 Hz, 1H), 3.56 (dd, *J* = 8.6, 2.4 Hz, 1H), 3.43 (dd, *J* = 8.7, 2.0 Hz, 1H), 1.23 (d, *J* = 6.5 Hz, 3H); **$^{13}$C NMR** (126 MHz, methanol-*d*$_4$) δ 136.8, 134.5, 131.3, 115.7, 73.3, 73.3, 71.3, 66.2, 18.5; **HRMS**: calcd for $C_9H_{16}O_4$ ([M + Na]$^+$): 211.0941, found: 211.0934.

*(2R,3S,4R, E)-octa-5,7-diene-1,2,3,4-tetraol (**4an**):*

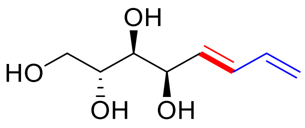

**D-Arabinose**

$[Pd(allyl)Cl]_2$ (1.9 mg, 2.5 mol%), tris(4-trifluoromethylphenyl)phosphine (9.4 mg, 10 mol%), allyl acetate **2** (0.6 mmol),and 1,4-dioxane (0.5 mL) were added into a dried microwave vial (10 mL) equipped with a stir bar in the glovebox. The reaction mixture was stirred at room temperature for 5 min before hydrazone **1ao** (33 mg, 0.2 mmol), 3Å MS (100 mg), NaOH (48 mg, 1.2 mmol), and 1,4-dioxane (1.5 mL) were added. The reaction tube was sealed and moved out of the glovebox. The resulting mixture was stirred at 60 °C for 24 hr. After the completion of the reaction, to the reaction solution was added 1.0 mL of water and stirred for 1h. The solution was then filtered through a short celite pad and washed with DCM/MeOH (1:2, 60 mL). The combined solution was concentrated under vacuum, and the residue was purified by flash column chromatography to give the desired product. **4an**: yield 65%, white solid, $R_f$ = 0.36 (DCM/MeOH, 6:1); E/Z > 14:1 (The E/Z ratio was determined based on analysis of the $^1$H-NMR spectrum); $[\alpha]_D^{21}$ = +39.6 (c = 0.5, MeOH); **$^1$H NMR** (500 MHz, methanol-*d*$_4$) δ 6.47 – 6.26 (m, 2H), 5.87 (dd, *J* = 15.0, 6.4 Hz, 1H), 5.20 (dd, *J* = 16.7, 1.9 Hz, 1H), 5.06 (dd, *J* = 9.7, 1.8 Hz, 1H), 4.37 (dd, *J* = 6.5, 1.5 Hz, 1H), 3.77 (dd, *J* = 11.1, 3.5 Hz, 1H), 3.69 (ddd, *J* = 7.7, 5.9, 3.5 Hz, 1H), 3.62 (dd, *J* = 11.2, 6.0 Hz, 1H), 3.46 (dd, *J* = 7.7, 2.8 Hz, 1H); **$^{13}$C NMR** (126 MHz, methanol-*d*$_4$) δ 136.7, 134.1, 131.5, 115.8, 74.1, 71.7, 71.1, 63.5; **HRMS**: calcd for $C_8H_{14}O_4$ ($[M + Na]^+$): 197.0784, found: 197.0781.

*(2S,3S,4S,5S, E)-nona-6,8-diene-2,3,4,5-tetraol (**4ao**):*

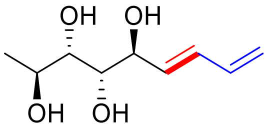

**L-Rhamnose**

$[Pd(allyl)Cl]_2$ (1.9 mg, 2.5 mol%), tris(4-trifluoromethylphenyl)phosphine (9.4 mg, 10 mol%), allyl acetate **2** (0.6 mmol), and 1,4-dioxane (0.5 mL) were added into a dried microwave vial (10 mL) equipped with a stir bar in the glovebox. The reaction mixture was stirred at room temperature for 5 min before hydrazone **1aq** (36 mg, 0.2 mmol), 3Å MS (100 mg), NaOH (48 mg, 1.2 mmol), and 1,4-dioxane (1.5 mL) were added. The reaction tube was sealed and moved out of the glovebox. The resulting mixture was stirred at 60 °C for 24 hr. After the completion of the reaction, to the

reaction solution was added 1.0 mL of water and stirred for 1 hr. The solution was then filtered through a short celite pad and washed with DCM/MeOH (1:2, 60 mL). The combined solution was concentrated under vacuum, and the residue was purified by flash column chromatography to give the desired product. **4ao**: yield 70%, white solid, $R_f$ = 0.4 (DCM/MeOH, 10:1); E/Z > 13:1 (The E/Z ratio was determined based on analysis of the $^1$H-NMR spectrum); $[\alpha]_D^{21}$ = -59.5 (c = 0.5, MeOH); **$^1$H NMR** (500 MHz, Methanol-$d_4$) δ 6.44 – 6.24 (m, 2H), 5.85 (dd, *J* = 14.9, 6.2 Hz, 1H), 5.18 – 5.11 (dd, *J* = 9.6, 1.8 Hz, 1H), 5.02 (dd, *J* = 9.6, 1.8 Hz, 1H), 4.21 – 4.12 (m, 1H), 3.75 (dt, *J* = 7.5, 6.2 Hz, 1H), 3.63 (dd, *J* = 7.3, 1.7 Hz, 1H), 3.50 (dd, *J* = 7.6, 1.7 Hz, 1H), 1.21 (d, *J* = 6.3 Hz, 3H); **$^{13}$C NMR** (126 MHz, Methanol-$d_4$) δ 136.8, 134.5, 131.7, 115.7, 73.8, 72.3, 72.0, 67.4, 18.9; **HRMS**: calcd for $C_9H_{16}O_4$ ([M + Na]$^+$): 211.0941, found: 211.0934.

*(2R,3R,4S, E)-1,3,4-tris(benzyloxy)octa-5,7-dien-2-ol (**4ap**):*

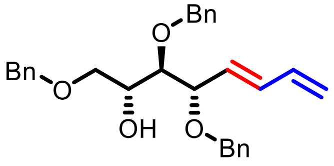


**D-Ribose**

Following the **general procedure D**, the title compound was obtained from the reaction of hydrazone of 2,3,5-Tri-O-benzyl-D-ribose (0.20 mmol, 1.0 M generated *in situ* from 2,3,5-Tri-O-benzyl-D-ribose and hydrazine monohydrate) with allyl acetate (0.6 mmol). The reaction was stirred at 45 °C for 18 hr and isolated by column chromatography on silica gel. **4ap**: yield 55%, colorless oil, $R_f$ = 0.33 (hexane/EtOAc, 9:1); E/Z > 20:1 (The E/Z ratio was determined based on analysis of the $^1$H-NMR spectrum); $[\alpha]_D^{20}$ = 16.5 (c = 1.0, $CHCl_3$); **$^1$H NMR** (400 MHz, chloroform-*d*) δ 7.35 – 7.23 (m, 15H), 6.54 – 6.17 (m, 2H), 5.79 (dd, J = 15.0, 8.4 Hz, 1H), 5.24 (dd, J = 16.5, 1.7 Hz, 1H), 5.18 – 5.09 (m, 1H), 4.78 (d, J = 11.1 Hz, 1H), 4.64 (d, J = 11.9 Hz, 1H), 4.57 (d, J = 11.2 Hz, 1H), 4.54 – 4.46 (m, 2H), 4.37 (d, J = 11.8 Hz, 1H), 4.19 (dd, J = 8.4, 4.0 Hz, 1H), 3.80 (ddt, J = 8.1, 5.1, 2.6 Hz, 1H), 3.72 (dd, J = 7.9, 4.0 Hz, 1H), 3.67 – 3.56 (m, 2H), 2.72 (d, J = 4.8 Hz, 1H); **$^{13}$C NMR** (101 MHz, chloroform-*d*) δ 138.4, 138.4, 138.0, 136.3, 135.6, 130.5, 128.5, 128.4, 128.3, 128.2, 127.9, 127.8, 127.7, 127.6, 127.5, 118.1, 81.4, 81.1, 74.3, 73.4, 71.1, 70.9, 70.5.

*(5S,6S,7R)-5,6,7,8-tetramethoxy-2-methyloct-1-ene (**3ba**):*

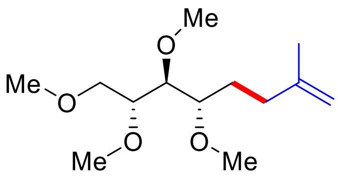


**D-Ribose**

Following the **general procedure C**, the title compound was obtained from the reaction of hydrazone of **1aa** (0.24 mmol, 1.0 M generated *in situ* from **1aa** and hydrazine monohydrate) with **2ba** (0.2 mmol). The reaction mixture was stirred at 45 °C for 3 hr and the product was isolated by column chromatography on silica gel. **3ba**: yield 80%, colorless oil, $R_f$ = 0.37 (hexane/EtOAc, 9:1); $[\alpha]_D^{22}$ = +4.3 (c = 2.0, $CHCl_3$); **H NMR** (500 MHz, chloroform-*d*) δ 4.70 (s, 2H), 3.63 (dd, *J* = 10.5, 2.7 Hz, 1H), 3.50 (dd, *J* = 10.5, 5.0 Hz, 1H), 3.46 (s, 3H), 3.44 (dd, *J* = 6.3, 3.8 Hz, 1H), 3.40 (s, 3H), 3.38 (s, 3H), 3.37 (s, 3H), 3.35 – 3.31 (m, 2H), 2.18 (ddd, *J* = 14.9, 9.9, 5.3 Hz, 1H), 2.08 (ddd, *J* = 14.9, 9.5, 6.7 Hz, 1H), 1.72 (s, 3H), 1.71 – 1.59 (m, 2H); **$^{13}$C NMR** (126 MHz, chloroform-*d*) δ 145.9, 110.0, 80.9, 80.4, 80.2, 71.5, 60.0, 59.2, 57.80, 57.75, 33.7, 27.7, 22.5. **HRMS**: calcd for $C_{13}H_{26}O_4$ ($[M + Na]^+$): 269.1723, found: 269.1717.

*(5S,6S,7R)-2-chloro-5,6,7,8-tetramethoxyoct-1-ene (**3bb**):*

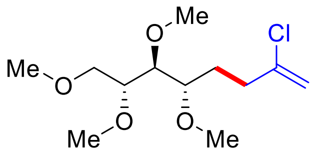


**D-Ribose**

Following the **general procedure C**, the title compound was obtained from the reaction of hydrazone of **1aa** (0.24 mmol, 1.0 M generated *in situ* from **1aa** and hydrazine monohydrate) with **2bb** (0.2 mmol). The reaction mixture was stirred at 45 °C for 3 hr and the product was isolated by column chromatography on silica gel. **3bb**: yield 50%, colorless oil, $R_f$ = 0.5 (hexane/EtOAc, 85:15); $[\alpha]_D^{22}$ = +11.1 (c = 2.0, $CHCl_3$); **$^1$H NMR** (500 MHz, chloroform-*d*) δ 5.16 (s, 2H), 3.64 (dd, *J* = 10.5, 2.8 Hz, 1H), 3.52 – 3.47 (m, 2H), 3.46 (s, 3H), 3.41 (s, 3H), 3.39 (s, 3H), 3.39 – 3.38 (m, 1H), 3.37 (s, 3H), 3.31 – 3.27 (m, 1H), 2.56 – 2.40 (m, 2H), 1.87 – 1.75 (m, 2H); **$^{13}$C NMR** (126 MHz, chloroform-*d*) δ 143.0, 112.3, 80.2, 80.1, 79.6, 71.2, 60.1, 59.2, 57.9, 57.6, 35.3, 27.2; **HRMS**: calcd for $C_{12}H_{23}ClO_4$ ($[M + Na]^+$): 289.1177, found: 289.1177.

*((5S,6S,7R)-5,6,7,8-tetramethoxyoct-1-en-2-yl) benzene (**3bc**):*

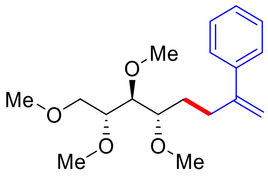

**D-Ribose**

Following the **general procedure C**, the title compound was obtained from the reaction of hydrazone of **1aa** (0.24 mmol, 1.0 M generated *in situ* from **1aa** and hydrazine monohydrate) with **2bc** (0.2 mmol). The reaction mixture was stirred at 45 °C for 3 hr and the product was isolated by column chromatography on silica gel. **3bc**: yield 78%, colorless oil, $R_f$ = 0.43 (hexane/EtOAc, 85:15); $[\alpha]_D^{22}$ = +9.5 (c = 2.0, $CHCl_3$); **$^1$H NMR** (500 MHz, chloroform-d) δ 7.47 – 7.42 (m, 2H), 7.36 – 7.31 (m, 2H), 7.29 – 7.24 (m, 1H), 5.32 (s, 1H), 5.13 (s, 1H), 3.62 (dd, J = 10.5, 2.7 Hz, 1H), 3.52 – 3.48 (m, 2H), 3.48 (s, 3H), 3.42 (d, J = 3.6 Hz, 1H), 3.40 (d, J = 1.7 Hz, 6H), 3.30 (s, 3H), 3.24 (m, 1H), 2.76 (dddd, J = 14.7, 9.6, 5.0, 1.4 Hz, 1H), 2.61 (dddd, J = 14.6, 9.2, 6.7, 1.1 Hz, 1H), 1.79 (m, 1H), 1.70 – 1.62 (m, 1H). **$^{13}$C NMR** (126 MHz, chloroform-d) δ 143.0, 112.3, 80.2, 80.1, 79.6, 71.2, 60.1, 59.2, 57.9, 57.6, 35.3, 27.2. **HRMS**: calcd for $C_{12}H_{23}ClO_4$ ($[M + Na]^+$): 289.1177, found: 289.1177.

<u>*1-chloro-4-((5S,6S,7R)-5,6,7,8-tetramethoxyoct-1-en-2-yl) benzene (**3bd**):*</u>

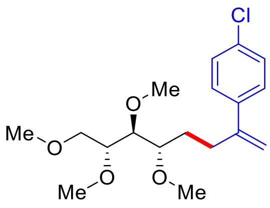

**D-Ribose**

Following the **general procedure C**, the title compound was obtained from the reaction of hydrazone of **1aa** (0.24 mmol, 1.0 M generated *in situ* from **1aa** and hydrazine monohydrate) with **2bd** (0.2 mmol). The reaction mixture was stirred at 45 °C for 3 hr and the product was isolated by column chromatography on silica gel. **3bd**: yield 70%, colorless oil, $R_f$ = 0.43 (hexane/EtOAc, 85:15); $[\alpha]_D^{22}$ = +12.3 (c = 1.0, $CHCl_3$); **$^1$H NMR** (500 MHz, chloroform-*d*) δ 7.37 – 7.32 (m, 2H), 7.30 – 7.26 (m, 2H), 5.28 (s, 1H), 5.11 (s, 1H), 3.60 (dd, *J* = 10.5, 2.7 Hz, 1H), 3.49 – 3.45 (m, 2H), 3.45 (s, 3H), 3.37 (d, *J* = 2.1 Hz, 6H), 3.36 – 3.34 (m, 1H), 3.29 (s, 3H), 3.23 – 3.20 (m, 1H), 2.74 – 2.65 (m, 1H), 2.54 (dddd, *J* = 14.7, 9.4, 6.7, 1.1 Hz, 1H), 1.80 – 1.69 (m, 1H), 1.62 – 1.55 (m, 1H); **$^{13}$C NMR** (126 MHz, chloroform-*d*) δ 147.3, 139.6, 128.4, 127.5, 113.0, 80.6, 80.2, 79.7,

71.2, 60.0, 59.2, 57.70, 57.66, 31.1, 28.1. **HRMS**: calcd for $C_{18}H_{27}ClO_4$ ([M + Na]$^+$): 365.1490, found: 365.1491.

*1-methoxy-4-((5S,6S,7R)-5,6,7,8-tetramethoxyoct-1-en-2-yl) benzene (**3be**):*

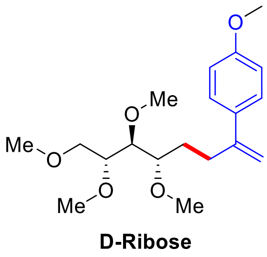


Following the **general procedure C**, the title compound was obtained from the reaction of hydrazone of **1aa** (0.24 mmol, 1.0 M generated *in situ* from **1aa** and hydrazine monohydrate) with **2be** (0.2 mmol). The reaction mixture was stirred at 45 °C for 3 hr and the product was isolated by column chromatography on silica gel. **3be**: yield 66%, colorless oil, $R_f$ = 0.35 (hexane/EtOAc, 85:15); $[\alpha]_D^{22}$ = +14.8 (c = 1.0, $CHCl_3$); **$^1$H NMR** (500 MHz, chloroform-*d*) δ 7.41 – 7.33 (m, 2H), 6.88 – 6.81 (m, 2H), 5.23 (d, *J* = 1.6 Hz, 1H), 5.02 (d, *J* = 1.4 Hz, 1H), 3.80 (s, 3H), 3.60 (dd, *J* = 10.6, 2.7 Hz, 1H), 3.49 – 3.45 (m, 2H), 3.45 (s, 3H), 3.39 (dd, *J* = 5.3, 2.9 Hz, 1H), 3.37 (d, *J* = 1.2 Hz, 6H), 3.30 (s, 3H), 3.22 (ddd, *J* = 6.4, 4.9, 2.7 Hz, 1H), 2.70 (dddd, *J* = 14.6, 9.5, 5.1, 1.3 Hz, 1H), 2.55 (dddd, *J* = 14.7, 9.4, 6.7, 1.0 Hz, 1H), 1.75 (dtd, *J* = 14.1, 9.0, 5.1 Hz, 1H), 1.66 – 1.60 (m, 1H); **$^{13}$C NMR** (126 MHz, chloroform-*d*) δ 147.6, 133.5, 127.2, 113.6, 110.9, 80.7, 80.3, 79.9, 71.4, 60.0, 59.2, 57.8, 57.7, 55.3, 31.2, 28.2; **HRMS**: calcd for $C_{19}H_{30}O_5$ ([M + Na]$^+$): 361.1985, found: 361.1989.

*2,4-dimethyl-1-((5S,6S,7R)-5,6,7,8-tetramethoxyoct-1-en-2-yl) benzene (**3bf**):*

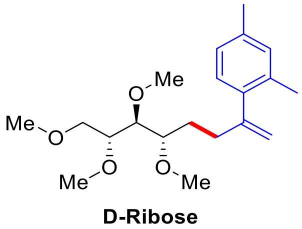


Following the **general procedure C**, the title compound was obtained from the reaction of hydrazone of **1aa** (0.24 mmol, 1.0 M generated *in situ* from **1aa** and hydrazine monohydrate) with **2bf** (0.2 mmol). The reaction mixture was stirred at 45 °C for 3 hr and the product was isolated by column chromatography on silica gel. **3bf**: yield 58%, colorless oil, $R_f$ = 0.45 (hexane/EtOAc, 85:15); **$^1$H NMR** (500 MHz, chloroform-*d*) δ 7.01 – 6.97 (m, 2H), 6.94 (d, *J* = 8.4 Hz, 1H), 5.19

(d, $J$ = 1.8 Hz, 1H), 4.86 (d, $J$ = 2.0 Hz, 1H), 3.62 (dd, $J$ = 10.6, 2.8 Hz, 1H), 3.48 (dd, $J$ = 10.5, 5.0 Hz, 1H), 3.44 (s, 3H), 3.43 – 3.41 (m, 1H), 3.38 (s, 3H), 3.37 – 3.36 (m, 1H), 3.35 (s, 3H), 3.34 (s, 3H), 3.30 (ddd, $J$ = 6.1, 5.0, 2.7 Hz, 1H), 2.56 – 2.47 (m, 1H), 2.38 (ddd, $J$ = 18.1, 9.5, 4.5 Hz, 1H), 2.30 (s, 3H), 2.27 (s, 3H), 1.70 (dddd, $J$ = 13.6, 10.0, 8.4, 5.1 Hz, 1H), 1.61 (ddt, $J$ = 7.7, 6.6, 3.8 Hz, 1H); **$^{13}$C NMR** (126 MHz, chloroform-*d*) δ 149.8, 140.2, 130.9, 128.3, 126.0, 113.7, 80.8, 80.3, 80.1, 71.4, 60.0, 59.1, 57.8, 57.7, 33.5, 27.8, 21.0, 19.9; **HRMS**: calcd for $C_{20}H_{32}O_4$ ([M + Na]$^+$): 359.2193, found: 359.2206.

*4-((5S,6S,7R)-5,6,7,8-tetramethoxyoct-1-en-2-yl)benzonitrile (**3bg**):*

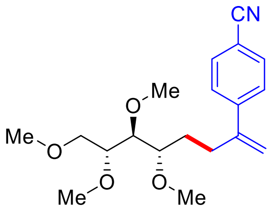


**D-Ribose**

Following the **general procedure C**, the title compound was obtained from the reaction of hydrazone of **1aa** (0.24 mmol, 1.0 M generated *in situ* from **1aa** and hydrazine monohydrate) with **2bg** (0.2 mmol). The reaction mixture was stirred at 45 °C for 3 hr and the product was isolated by column chromatography on silica gel. **3bg**: yield 60%, colorless oil, $R_f$ = 0.3 (hexane/EtOAc, 85:15); **$^1$H NMR** (500 MHz, chloroform-*d*) δ 7.61 (d, $J$ = 8.5 Hz, 2H), 7.51 (d, $J$ = 8.4 Hz, 2H), 5.39 (s, 1H), 5.25 (s, 1H), 3.60 (dd, $J$ = 10.5, 2.8 Hz, 1H), 3.47 (dd, $J$ = 6.6, 4.0 Hz, 2H), 3.45 (s, 3H), 3.38 (s, 3H), 3.37 (s, 3H), 3.36 – 3.34 (m, 1H), 3.29 (s, 3H), 3.21 (td, $J$ = 4.5, 2.3 Hz, 1H), 2.76 – 2.68 (m, 1H), 2.61 – 2.52 (m, 1H), 1.74 (m, 1H), 1.61 – 1.56 (m, 1H); **$^{13}$C NMR** (126 MHz, chloroform-*d*) δ 147.1, 145.8, 132.1, 126.8, 118.9, 115.3, 110.8, 80.6, 80.1, 79.5, 71.1, 60.0, 59.2, 57.7, 30.9, 28.0; **HRMS**: calcd for $C_{19}H_{27}NO_4$ ([M + Na]$^+$): 356.1832, found: 356.1818.

*2,2,2-trifluoro-N-(4-((5S,6S,7R)-5,6,7,8-tetramethoxyoct-1-en-2-yl)phenyl)acetamide (**3bh**):*

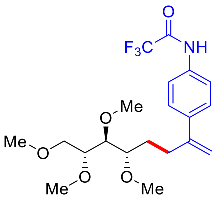


**D-Ribose**

Following the **general procedure C**, the title compound was obtained from the reaction of hydrazone of **1aa** (0.24 mmol, 1.0 M generated *in situ* from **1aa** and hydrazine monohydrate) with

**2bh** (0.2 mmol). The reaction mixture was stirred at 45 °C for 3 hr and the product was isolated by column chromatography on silica gel. **3bh**: yield 78%, white solid; $R_f$ = 0.25 (hexane/EtOAc, 85:15); **$^1$H NMR** (500 MHz, chloroform-*d*) δ 7.94 (s, 1H), 7.59 – 7.51 (m, 2H), 7.38 – 7.28 (m, 2H), 5.33 (s, 1H), 5.16 (s, 1H), 3.61 (dd, *J* = 10.5, 2.8 Hz, 1H), 3.50 – 3.46 (m, 2H), 3.45 (s, 3H), 3.43 (q, *J* = 2.1 Hz, 1H), 3.38 (d, *J* = 2.6 Hz, 6H), 3.29 (s, 3H), 3.26 – 3.22 (m, 1H), 2.74 – 2.68 (m, 1H), 2.61 – 2.49 (m, 1H), 1.80 – 1.72 (m, 1H), 1.67 – 1.62 (m, 1H); **$^{13}$C NMR** (126 MHz, chloroform-*d*) δ 154.7 (q, *J* = 36.9 Hz), 147.4, 142.6, 135.2, 129.3, 124.1, 119.4, 118.3, δ 115.7 (q, *J* = 288.4Hz), 113.6, 80.7, 80.1, 79.6, 71.2, 59.9, 59.1, 57.71, 57.65, 31.2, 28.2; **HRMS**: calcd for $C_{20}H_{28}F_3NO_5$ ([M + Na]$^+$): 442.1812, found: 442.1801.

*2-fluoro-4-methoxy-1-((5S,6S,7R)-5,6,7,8-tetramethoxyoct-1-en-2-yl)benzene (**3bi**):*

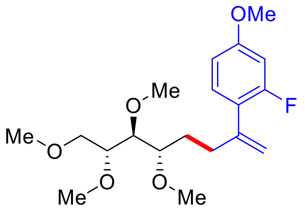


**D-Ribose**

Following the **general procedure C**, the title compound was obtained from the reaction of hydrazone of **1aa** (0.24 mmol, 1.0 M generated *in situ* from **1aa** and hydrazine monohydrate) with **2bi** (0.2 mmol). The reaction mixture was stirred at 45 °C for 3 hr and the product was isolated by column chromatography on silica gel. **3bi**: yield 58%, colorless oil; $R_f$ = 0.25 (hexane/EtOAc, 85:15); $[\alpha]_D^{22}$ = +21.6 (c = 1.0, $CHCl_3$); **$^1$H NMR** (400 MHz, chloroform-*d*) δ 7.18 (t, *J* = 8.7 Hz, 1H), 6.67 – 6.56 (m, 2H), 5.14 (s, 1H), 3.79 (s, 3H), 3.60 (dd, *J* = 10.6, 2.7 Hz, 1H), 3.49 – 3.46 (m, 1H), 3.44 (s, 3H), 3.43 – 3.39 (m, 2H), 3.37 (s, 3H), 3.36 (s, 3H), 3.32 (s, 3H), 3.24 (ddd, *J* = 6.3, 4.9, 2.7 Hz, 1H), 2.67 (ddd, *J* = 14.7, 9.5, 5.2 Hz, 1H), 2.52 (dt, *J* = 15.1, 8.0 Hz, 1H), 1.73 – 1.61 (m, 2H); **$^{13}$C NMR** (126 MHz, chloroform-*d*) δ 160.5 (d, *J* = 247.80 Hz), 159.9 (d, *J* = 11.0 Hz), 144.4 (d, *J* = 1.7 Hz), 130.3 (d, *J* = 6.3 Hz), 121.9 (d, *J* = 14.6 Hz), 115.1 (d, *J* = 3.1 Hz), 109.7 (d, *J* = 2.9 Hz), 101.8 (d, *J* = 26.9 Hz), 80.6, 80.2, 79.9, 71.4, 60.0, 59.1, 57.7, 57.7, 55.5, 32.4 (d, *J* = 2.9 Hz), 28.1. **$^{19}$F NMR** (471 MHz, chloroform-*d*) δ -112.73; **HRMS**: calcd for $C_{19}H_{29}O_5F$ ([M + Na]$^+$): 379.1891, found: 379.1895.

*(4R,4'S,5S)-2,2,2',2'-tetramethyl-5-(3-phenylbut-3-en-1-yl)-4,4'-bi(1,3-dioxolane) (**3bj**):*

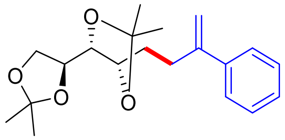

**L-Arabinose**

Following the **general procedure C**, the title compound was obtained from the reaction of hydrazone of **1ai** (0.24 mmol, 1.0 M generated *in situ* from **1ai** and hydrazine monohydrate) with **2bc** (0.2 mmol). The reaction mixture was stirred at 45 °C for 3 hr and the product was isolated by column chromatography on silica gel. **3bj**: yield 88%, colorless oil; $R_f$ = 0.63 (hexane/EtOAc, 85:15); $[\alpha]_D^{22}$ = +22.9 (c = 1.0, $CHCl_3$); **$^1$H NMR** (500 MHz, chloroform-*d*) δ 7.48 – 7.39 (m, 2H), 7.36 – 7.29 (m, 2H), 7.29 – 7.23 (m, 1H), 5.31 (d, *J* = 1.2 Hz, 1H), 5.11 (d, *J* = 1.5 Hz, 1H), 4.10 (dd, *J* = 8.5, 6.1 Hz, 1H), 4.04 – 3.95 (m, 2H), 3.92 (dd, *J* = 8.5, 5.2 Hz, 1H), 3.57 (t, *J* = 7.8 Hz, 1H), 2.76 (dddd, *J* = 15.4, 10.7, 4.9, 1.4 Hz, 1H), 2.68 – 2.56 (m, 1H), 1.93 (dddd, *J* = 14.0, 10.8, 5.9, 3.4 Hz, 1H), 1.80 – 1.69 (m, 1H), 1.40 (s, 3H), 1.36 (s, 3H), 1.33 (s, 3H), 1.32 (s, 3H); **$^{13}$C NMR** (126 MHz, chloroform-*d*) δ 148.0, 141.2, 128.2, 127.3, 126.1, 112.2, 109.5, 108.9, 81.1, 80.0, 77.2, 67.7, 32.5, 31.5, 27.4, 27.1, 26.7, 25.3; **HRMS**: calcd for $C_{20}H_{28}O_4$ ([M + Na]$^+$): 355.1880, found: 355.1876.

*(4R,4'S,5S)-2,2,2',2'-tetramethyl-5-(3-(4-(trifluoromethyl)phenyl)but-3-en-1-yl)-4,4'-bi(1,3-dioxolane) (**3bk**):*

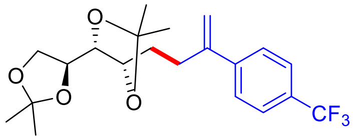

**L-Arabinose**

Following the **general procedure C**, the title compound was obtained from the reaction of hydrazone of **1ai** (0.24 mmol, 1.0 M generated *in situ* from **1ai** and hydrazine monohydrate) with 2-(4-(trifluoromethyl)phenyl)allyl acetate (0.2 mmol). The reaction mixture was stirred at 45 °C for 3 hr and the product was isolated by column chromatography on silica gel. **3bk**: yield 90%, colorless oil; $R_f$ = 0.65 (hexane/EtOAc, 85:15); $[\alpha]_D^{22}$ = +33.1(c = 1.0, $CHCl_3$); **$^1$H NMR** (500 MHz, chloroform-*d*) δ 7.57 (d, *J* = 8.2 Hz, 2H), 7.52 (d, *J* = 8.2 Hz, 2H), 5.37 (s, 1H), 5.21 (s, 1H), 4.11 (dd, *J* = 8.5, 6.1 Hz, 1H), 4.02 – 3.94 (m, 2H), 3.92 (dd, *J* = 8.5, 5.1 Hz, 1H), 3.54 (t, *J* = 7.8 Hz, 1H), 2.76 (ddd, *J* = 15.3, 10.6, 4.9 Hz, 1H), 2.63 (ddd, *J* = 15.5, 10.2, 6.1 Hz, 1H), 1.94 – 1.88 (m, 1H), 1.75 – 1.67 (m, 1H), 1.39 (s, 3H), 1.36 (s, 3H), 1.31 (s, 3H), 1.30 (s, 3H); **$^{13}$C NMR** (126

MHz, chloroform-*d*) δ 146.9, 144.8, 129.3 (q, *J* = 32.1) 126.5, 125.2 (q, *J* = 3.1), 124.1 (q, *J* = 272.1), 114.2, 109.5, 109.0, 81.1, 79.9, 77.2, 67.8, 32.3, 31.4, 27.4, 27.0, 26.6, 25.2; **$^{19}$F NMR** (471 MHz, chloroform-*d*) δ -62.51; **HRMS**: calcd for $C_{21}H_{27}F_3O_4$ ([M + Na]$^+$): 423.1754, found: 423.1741.

*(4R,4'S,5S)-2,2,2',2'-tetramethyl-5-(3-(3-(trifluoromethyl)phenyl)but-3-en-1-yl)-4,4'-bi(1,3-dioxolane) (**3bl**):*

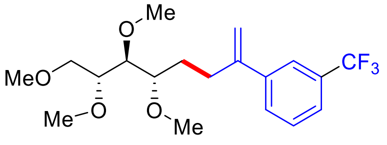


**D-Ribose**

Following the **general procedure C**, the title compound was obtained from the reaction of hydrazone of **1aa** (0.24 mmol, 1.0 M generated *in situ* from **1aa** and hydrazine monohydrate) with **2bl** (0.2 mmol). The reaction mixture was stirred at 45 °C for 3 hr and the product was isolated by column chromatography on silica gel. **3bl**: yield 80%, colorless oil; $R_f$ = 0.5 (hexane/EtOAc, 85:15); **$^1$H NMR** (500 MHz, chloroform-*d*) δ 7.66 (s, 1H), 7.60 (d, *J* = 7.7 Hz, 1H), 7.51 (d, *J* = 6.9 Hz, 1H), 7.43 (t, *J* = 7.7 Hz, 1H), 5.35 (s, 1H), 5.20 (s, 1H), 3.60 (dd, *J* = 10.5, 2.8 Hz, 1H), 3.48 (dd, *J* = 4.2, 1.4 Hz, 1H), 3.45 (d, *J* = 2.8 Hz, 3H), 3.44 (t, *J* = 2.9 Hz, 1H), 3.40 (dd, *J* = 8.1, 3.7 Hz, 1H), 3.37 (s, 6H), 3.28 (s, 3H), 3.20 (m, 1H), 2.78 – 2.69 (m, 1H), 2.60 (m, 1H), 1.80 – 1.70 (m, 1H), 1.63 – 1.57 (m, 1H); **$^{13}$C NMR** (126 MHz, chloroform-*d*) δ 147.3, 142.0, 130.4 (q, *J* = 32.50 Hz), 129.5 (2x), 128.7, 124.2 (q, *J* = 272.43 Hz), 124.0 (q, *J* = 3.8 Hz), 122.9 (q, *J* = 3.7 Hz), 114.1, 80.5, 80.2, 79.5, 71.1, 60.0, 59.2, 57.7, 57.6, 31.0, 27.9; **$^{19}$F NMR** (471 MHz, chloroform-*d*) δ -62.62. **HRMS**: calcd for $C_{19}H_{27}F_3O_4$ ([M + Na]$^+$): 399.1754, found: 399.1761.

*(4R,4'S,5S)-5-(3-benzylbut-3-en-1-yl)-2,2,2',2'-tetramethyl-4,4'-bi(1,3-dioxolane) (**3bm**):*

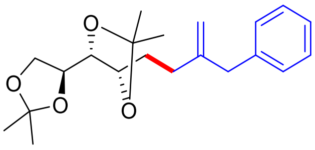


**L-Arabinose**

Following the **general procedure C**, the title compound was obtained from the reaction of hydrazone of **1ai** (0.24 mmol, 1.0 M generated *in situ* from **1ai** and hydrazine monohydrate) with **2bm** (0.2 mmol). The reaction mixture was stirred at 45 °C for 3 hr and the product was isolated

by column chromatography on silica gel. **3bm**: yield 75%, colorless oil; $R_f$ = 0.73 (hexane/EtOAc, 85:15); **$^1$H NMR** (500 MHz, chloroform-*d*) δ 7.29 (dd, *J* = 8.6, 6.5 Hz, 2H), 7.22 – 7.17 (m, 3H), 4.87 (d, *J* = 1.7 Hz, 1H), 4.77 (d, *J* = 1.7 Hz, 1H), 4.14 – 4.09 (m, 1H), 4.00 (dt, *J* = 8.2, 5.6 Hz, 1H), 3.96 – 3.85 (m, 2H), 3.55 (t, *J* = 7.8 Hz, 1H), 3.42 – 3.31 (m, 2H), 2.21 (ddt, *J* = 14.5, 8.9, 4.4 Hz, 1H), 2.11 (ddd, *J* = 15.5, 10.1, 6.1 Hz, 1H), 1.93 (dddt, *J* = 13.8, 10.6, 6.1, 2.6 Hz, 1H), 1.74 – 1.66 (m, 1H), 1.39 (s, 3H), 1.37 (s, 3H), 1.34 (d, *J* = 3.3 Hz, 5H); **$^{13}$C NMR** (126 MHz, chloroform-*d*) δ 148.5, 129.0, 128.3, 126.1, 111.2, 109.6, 108.9, 81.2, 80.0, 77.2, 67.8, 43.2, 31.7, 31.7, 27.4, 27.0, 26.7, 25.3; **HRMS**: calcd for $C_{21}H_{30}O_4$ ([M + Na]$^+$): 369.2036, found: 369.2040.

*(3aR,5R,5aS,8aS,8bR)-2,2,7,7-tetramethyl-5-(3-phenylbut-3-en-1-yl)tetrahydro-5H-bis([1,3]dioxolo)[4,5-b:4',5'-d]pyran (**3bn**):*

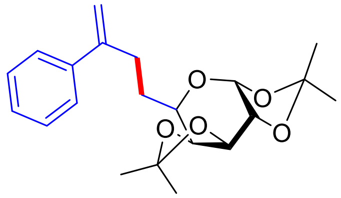


**α-D-Galactopyranose**

Following the **general procedure C**, the title compound was obtained from the reaction of hydrazone of **1ak** (0.24 mmol, 1.0 M generated *in situ* from **1ak** and hydrazine monohydrate) with **2bc** (0.2 mmol). The reaction mixture was stirred at 60 °C for 3 hr and the product was isolated by column chromatography on silica gel. **3bn**: yield 65%, colorless oil; $R_f$ = 0.58 (hexane/EtOAc, 9:1); $[\alpha]_D^{22}$ = -55.0 (c = 0.4, $CHCl_3$); **$^1$H NMR** (500 MHz, chloroform-*d*) δ 7.44 – 7.37 (m, 2H), 7.35 – 7.28 (m, 2H), 7.27 – 7.23 (m, 1H), 5.55 (d, *J* = 5.1 Hz, 1H), 5.30 (d, *J* = 1.4 Hz, 1H), 5.14 (d, *J* = 1.4 Hz, 1H), 4.57 (dd, *J* = 7.9, 2.3 Hz, 1H), 4.29 (dd, *J* = 5.1, 2.3 Hz, 1H), 4.07 (dd, *J* = 7.9, 1.8 Hz, 1H), 3.76 (ddd, *J* = 9.2, 4.2, 1.9 Hz, 1H), 2.81 – 2.68 (m, 1H), 2.57 (m, 1H), 1.87 (m, 1H), 1.68 (m, 1H), 1.52 (s, 3H), 1.45 (s, 3H), 1.33 (d, *J* = 5.6 Hz, 6H); **$^{13}$C NMR** (126 MHz, chloroform-*d*) δ 147.6, 141.0, 128.3, 127.3, 126.2, 113.0, 109.0, 108.3, 96.6, 73.0, 71.0, 70.5, 66.6, 31.1, 28.5, 26.1, 26.0, 25.0, 24.4; **HRMS**: calcd for $C_{21}H_{28}O_5$ ([M + Na]$^+$): 383.1829, found: 383.1817.

*(4R,4'S,5S)-2,2,2',2'-tetramethyl-5-(3-(thiophen-2-yl)but-3-en-1-yl)-4,4'-bi(1,3-dioxolane) (**3bo**):*

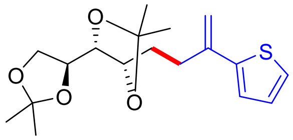


**L-Arabinose**

Following the **general procedure C**, the title compound was obtained from the reaction of hydrazone of **1ai** (0.24 mmol, 1.0 M generated *in situ* from **1ai** and hydrazine monohydrate) with **2bo** (0.2 mmol). The reaction mixture was stirred at 60 °C for 3 hr and the product was isolated by column chromatography on silica gel. **3bo**: yield 50%, colorless oil; $R_f$ = 0.55 (hexane/EtOAc, 85:15); **$^1$H NMR** (500 MHz, chloroform-*d*) δ 7.16 (dd, *J* = 5.1, 1.1 Hz, 1H), 7.08 (dd, *J* = 3.6, 1.2 Hz, 1H), 6.97 (dd, *J* = 5.1, 3.6 Hz, 1H), 5.41 (s, 1H), 5.00 (s, 1H), 4.12 (dd, *J* = 8.4, 6.1 Hz, 1H), 4.04 – 3.98 (m, 2H), 3.94 (dd, *J* = 8.5, 5.2 Hz, 1H), 3.59 (dd, *J* = 8.1, 7.4 Hz, 1H), 2.75 – 2.69 (m, 1H), 2.55 – 2.63 (m, 1H), 2.09 – 2.00 (m, 1H), 1.88 – 1.80 (m, 1H), 1.41 (s, 3H), 1.37 (s, 6H), 1.33 (s, 3H); **$^{13}$C NMR** (126 MHz, chloroform-*d*) δ 145.2, 141.3, 127.3, 124.1, 123.5, 110.8, 109.6, 109.0, 81.1, 80.0, 77.2, 67.8, 32.7, 31.7, 27.4, 27.1, 26.7, 25.3.

*Triisopropyl(3-methylene-5-((4R,4'S,5S)-2,2,2',2'-tetramethyl-[4,4'-bi(1,3-dioxolan)]-5-yl)pent-1-yn-1-yl)silane (**3bp**):*

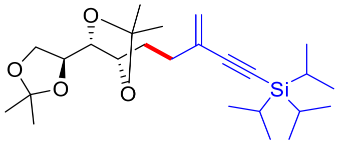

**L-Arabinose**

Following the **general procedure C**, the title compound was obtained from the reaction of hydrazone of **1ai** (0.24 mmol, 1.0 M generated *in situ* from **1ai** and hydrazine monohydrate) with **2bp** (0.2 mmol). The title compound was isolated by column chromatography on silica gel. **3bp**: yield 58%, colorless oil; $R_f$ = 0.72 (hexane/EtOAc, 85:15); **$^1$H NMR** (500 MHz, chloroform-*d*) δ 5.38 (s, 1H), 5.29 (s, 1H), 4.11 (dd, *J* = 8.3, 6.1 Hz, 1H), 4.06 – 3.98 (m, 1H), 3.98 – 3.88 (m, 2H), 3.58 (t, *J* = 7.6 Hz, 1H), 2.43-2.37 (m, 1H), 2.31-2.25 (m, 1H), 2.04-1.97 (m, 1H), 1.81-1.74 (m, 1H), 1.39 (s, 3H), 1.38 (s, 3H), 1.35 (s, 3H), 1.33 (s, 3H), 1.08 (m, 21H); **$^{13}$C NMR** (126 MHz, chloroform-*d*) δ 131.3, 121.8, 109.6, 108.9, 107.4, 90.2, 81.3, 79.7, 77.1, 67.56, 33.8, 32.5, 27.4, 27.1, 26.7, 25.3, 18.7, 11.3; **HRMS**: calcd for $C_{25}H_{44}O_4Si$ ($[M + Na]^+$): 459.2901, found: 459.2900.

*triisopropyl(3-methylene-5-((3aR,5R,5aS,8aS,8bR)-2,2,7,7-tetramethyltetrahydro-5H-bis([1,3]dioxolo)[4,5-b:4',5'-d]pyran-5-yl)pent-1-yn-1-yl)silane (**3bq**):*

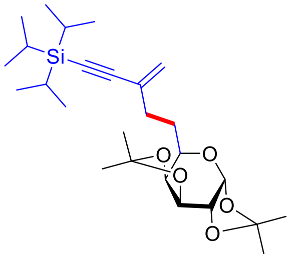

α-D-Galactopyranose

Following the **general procedure C**, the title compound was obtained from the reaction of hydrazone of **1ak** (0.24 mmol, 1.0 M generated *in situ* from **1ak** and hydrazine monohydrate) with **2bp** (0.2 mmol). The reaction mixture was stirred at 45 $^{o}$C for 3 hr and the product was isolated by column chromatography on silica gel. **3bq**: yield 60%, colorless oil; $R_f$ = 0.7 (hexane/EtOAc, 85:15); $[\alpha]_D^{22}$ = -48.1 (c = 0.3, $CHCl_3$); **$^1$H NMR** (500 MHz, chloroform-*d*) δ 5.52 (d, *J* = 5.1 Hz, 1H), 5.38 (s, 1H), 5.33 (s, 1H), 4.57 (dd, *J* = 7.9, 2.3 Hz, 1H), 4.28 (dd, *J* = 5.1, 2.3 Hz, 1H), 4.09 (dd, *J* = 7.9, 1.8 Hz, 1H), 3.72 (ddd, *J* = 9.3, 4.3, 1.8 Hz, 1H), 2.30 (m, 2H), 1.93 – 1.79 (m, 2H), 1.49 (s, 3H), 1.45 (s, 3H), 1.34 (s, 3H), 1.31 (s, 3H), 1.10 – 1.02 (m, 21H). **$^{13}$C NMR** (126 MHz, chloroform-*d*) δ 130.8, 122.9, 109.0, 108.3, 107.3, 96.6, 90.3, 73.1, 71.0, 70.5, 66.0, 32.9, 28.1, 26.01, 25.98, 25.0, 24.4, 18.6, 11.3. **HRMS**: calcd for $C_{26}H_{44}O_5Si$ ($[M + Na]^+$): 487.2850, found: 487.2847.

*(E)-4-phenylhexa-2,5-dien-1-ol (**3br**):*

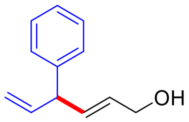


$Pd(MeCN)_4(BF_4)_2$ (4.4 mg, 5 mol%), 1,3-bis-(2,6-diisopropylphenyl)imidazolium chloride (**IPr•HCl**) (5.1 mg, 6 mol%), $^t$BuONa (1.2 mg, 6 mol%), and 2-Me-THF (0.5 mL) were added into a dried microwave vial (10 mL) equipped with a stir bar in the glovebox. The mixture was stirred at room temperature for 1 hr before allyl acetate **2bq** (0.2 mmol) was added. The mixture was stirred for another 0.5 hr. Then hydrazone solution of **1ag** (0.24 mL, 1 M), NaOH (12 mg, 0.3 mmol), and 2-Me-THF (1.25 mL) were added. The resulting solution was stirred at room temperature for 2 hr. After the completion of the reaction, the reaction solution was filtered through a short celite pad and washed with diethyl ether (60 mL). The combined solution was concentrated under vacuum, and the residue was purified by flash column chromatography with hexanes/ethyl acetate (4:1) as the eluent to give the desired product. **3br**: yield 81%, colorless oil; E/Z > 20:1 (The E/Z ratio was determined based on analysis of the $^1$H-NMR spectrum); **$^1$H NMR** (500 MHz,

chloroform-*d*) δ 7.32 (t, *J* = 7.5 Hz, 2H), 7.23 (t, *J* = 7.8 Hz, 3H), 6.04 (ddd, *J* = 17.1, 10.2, 6.8 Hz, 1H), 5.97 – 5.88 (m, 1H), 5.70 (dtd, *J* = 15.5, 5.7, 1.2 Hz, 1H), 5.15 (d, *J* = 10.3 Hz, 1H), 5.09 (d, *J* = 17.1 Hz, 1H), 4.16 (d, *J* = 5.4 Hz, 2H), 4.08 (t, *J* = 6.8 Hz, 1H), 1.47 (s, 1H). **$^{13}$C NMR** (126 MHz, chloroform-*d*) δ 142.5, 140.0, 133.8, 130.1, 128.5, 127.9, 126.5, 115.4, 63.5, 51.8. **HRMS**: calcd for $C_{12}H_{14}O$ ($[M + Na]^+$): 197.0937, found: 197.0929.

*(E)-4-(3-chlorophenyl)hexa-2,5-dien-1-ol (**3bs**):*

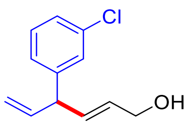


$Pd(MeCN)_4(BF_4)_2$ (4.4 mg, 5 mol%), 1,3-bis-(2,6-diisopropylphenyl)imidazolium chloride (**IPr•HCl**) (5.1 mg, 6 mol%), $^t$BuONa (1.2 mg, 6 mol%), and 2-Me-THF (0.5 mL) were added into a dried microwave vial (10 mL) equipped with a stir bar in the glovebox. The mixture was stirred at room temperature for 1 hr before allyl acetate **2br** (0.2 mmol) was added. The mixture was stirred for another 0.5 h. Then hydrazone solution of **1ag** (0.24 mL, 1 M), NaOH (12 mg, 0.3 mmol), and 2-Me-THF (1.25 mL) were added. The resulting solution was stirred at room temperature for 2 hr. After the completion of the reaction, the reaction solution was filtered through a short celite pad and washed with diethyl ether (60 mL). The combined solution was concentrated under vacuum, and the residue was purified by flash column chromatography with hexanes/ethyl acetate (4:1) as the eluent to give the desired product. **3bs**: yield 70%, colorless oil; E/Z > 20:1 (The E/Z ratio was determined based on analysis of the $^1$H-NMR spectrum); **$^1$H NMR** (500 MHz, chloroform-*d*) δ 7.32 – 7.19 (m, 3H), 7.11 (d, *J* = 7.4 Hz, 1H), 6.00 (ddd, *J* = 17.1, 10.2, 6.8 Hz, 1H), 5.95 – 5.85 (m, 1H), 5.71 (dd, *J* = 15.5, 5.6 Hz, 1H), 5.19 (d, *J* = 10.2 Hz, 1H), 5.11 (d, *J* = 17.1 Hz, 1H), 4.18 (d, *J* = 5.5 Hz, 2H), 4.06 (t, *J* = 7.0 Hz, 1H); **$^{13}$C NMR** (126 MHz, chloroform-*d*) δ 144.5, 139.2, 134.3, 132.8, 130.7, 129.8, 128.1, 126.7, 126.2, 116.1, 63.4, 51.5;

*(E)-4-(3-(trifluoromethyl)phenyl)hexa-2,5-dien-1-ol (**3bt**)*:

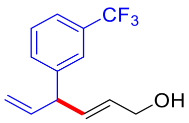


$Pd(MeCN)_4(BF_4)_2$ (4.4 mg, 5 mol%), 1,3-bis-(2,6-diisopropylphenyl)imidazolium chloride (**IPr•HCl**) (5.1 mg, 6 mol%), $^t$BuONa (1.2 mg, 6 mol%), and 2-Me-THF (0.5 mL) were added into a dried microwave vial (10 mL) equipped with a stir bar in the glovebox. The mixture was stirred at room temperature for 1 hr before allyl acetate **2bs** (0.2 mmol) was added. The mixture

was stirred for another 0.5 hr. Then hydrazone solution of **1ag** (0.24 mL, 1 M), NaOH (12 mg, 0.3 mmol), and 2-Me-THF (1.25 mL) were added. The resulting solution was stirred at room temperature for 2 hr. After the completion of the reaction, the reaction solution was filtered through a short celite pad and washed with diethyl ether (60 mL). The combined solution was concentrated under vacuum, and the residue was purified by flash column chromatography with hexanes/ethyl acetate (4:1) as the eluent to give the desired product. **3bt**: yield 90%, colorless oil; E/Z = 20:1(The E/Z ratio was determined based on analysis of the $^{1}$H-NMR spectrum); **$^{1}$H NMR** (500 MHz, chloroform-*d*) δ 7.52 – 7.37 (m, 4H), 6.01 (ddd, *J* = 17.1, 10.2, 6.8 Hz, 1H), 5.90 (ddt, *J* = 15.5, 7.0, 1.5 Hz, 1H), 5.71 (dtd, *J* = 15.5, 5.5, 1.3 Hz, 1H), 5.19 (dt, *J* = 10.2, 1.4 Hz, 1H), 5.10 (dt, *J* = 17.2, 1.5 Hz, 1H), 4.18 (d, *J* = 5.5 Hz, 2H), 4.14 (t, *J* = 7.0 Hz, 1H); **$^{13}$C NMR** (126 MHz, chloroform-*d*) δ 143.4, 139.1, 132.6, 131.5, 131.0, 130.9 (q, *J* = 32.1), 129.0, 124.7 (q, *J* = 3.8 Hz), 124.2 (q, *J* = 272.3 Hz), 123.4 (q, *J* = 3.8 Hz), 116.3, 63.3, 51.5; **$^{19}$F NMR** (471 MHz, chloroform-*d*) δ -62.51.

*(E)-4-(3-(trifluoromethyl)phenyl)hexa-2,5-dien-1-ol (**3bu**):*

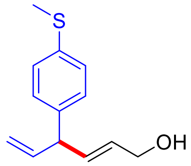


Pd(MeCN)$_4$(BF$_4$)$_2$ (4.4 mg, 5 mol%), 1,3-bis-(2,6-diisopropylphenyl)imidazolium chloride (**IPr•HCl**) (5.1 mg, 6 mol%), $^t$BuONa (1.2 mg, 6 mol%), and 2-Me-THF (0.5 mL) were added into a dried microwave vial (10 mL) equipped with a stir bar in the glovebox. The mixture was stirred at room temperature for 1 hr before allyl acetate **2bt** (0.2 mmol) was added. The mixture was stirred for another 0.5 hr. Then hydrazone solution of **1ag** (0.24 mL, 1 M), NaOH (12 mg, 0.3 mmol), and 2-Me-THF (1.25 mL) were added. The resulting solution was stirred at room temperature for 2 hr. After the completion of the reaction, the reaction solution was filtered through a short celite pad and washed with diethyl ether (60 mL). The combined solution was concentrated under vacuum, and the residue was purified by flash column chromatography with hexanes/ethyl acetate (4:1) as the eluent to give the desired product. **3bu**: yield 80%, colorless oil; E/Z > 20:1 (The E/Z ratio was determined based on analysis of the $^{1}$H-NMR spectrum); **$^{1}$H NMR** (500 MHz, chloroform-*d*) δ 7.22 (d, *J* = 8.3 Hz, 2H), 7.13 (d, *J* = 8.3 Hz, 2H), 6.00 (ddd, *J* = 17.0, 10.2, 6.7 Hz, 1H), 5.89 (dd, *J* = 15.5, 7.0 Hz, 1H), 5.72 – 5.64 (m, 1H), 5.14 (d, *J* = 10.2 Hz, 1H), 5.07 (d,

$J$ = 17.1 Hz, 1H), 4.16 (t, $J$ = 5.5 Hz, 2H), 4.03 (t, $J$ = 7.0 Hz, 1H), 2.47 (s, 3H); **$^{13}$C NMR** (126 MHz, chloroform-*d*) δ 139.8, 139.5, 136.4, 133.6, 130.2, 128.5, 127.1, 115.6, 63.5, 51.3, 16.2.

*(E)-4-(thiophen-3-yl)hexa-2,5-dien-1-ol (**3bv**):*

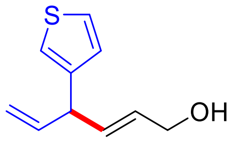


$Pd(MeCN)_4(BF_4)_2$ (4.4 mg, 5 mol%), 1,3-bis-(2,6-diisopropylphenyl)imidazolium chloride (**IPr•HCl**) (5.1 mg, 6 mol%), $^t$BuONa (1.2 mg, 6 mol%), and 2-Me-THF (0.5 mL) were added into a dried microwave vial (10 mL) equipped with a stir bar in the glovebox. The mixture was stirred at room temperature for 1 hr before allyl acetate **2bu** (0.2 mmol) was added. The mixture was stirred for another 0.5 hr. Then hydrazone solution of **1ag** (0.24 mL, 1 M), NaOH (12 mg, 0.3 mmol), and 2-Me-THF (1.25 mL) were added. The resulting solution was stirred at room temperature for 2 h. After the completion of the reaction, the reaction solution was filtered through a short celite pad and washed with diethyl ether (60 mL). The combined solution was concentrated under vacuum, and the residue was purified by flash column chromatography with hexanes/ethyl acetate (4:1) as the eluent to give the desired product. **3bv**: yield 66%, colorless oil; E/Z > 10:1 (The E/Z ratio was determined based on analysis of the $^1$H-NMR spectrum); **$^1$H NMR** (500 MHz, chloroform-*d*) δ 7.28 (dd, $J$ = 5.0, 3.0 Hz, 1H), 6.99 (d, $J$ = 2.6 Hz, 1H), 6.95 (dd, $J$ = 5.1, 1.3 Hz, 1H), 6.01 (ddd, $J$ = 17.1, 10.1, 7.0 Hz, 1H), 5.90 (dd, $J$ = 15.5, 7.2 Hz, 1H), 5.70 (dtd, $J$ = 15.2, 5.7, 1.2 Hz, 1H), 5.13 (d, $J$ = 10.2 Hz, 1H), 5.09 (d, $J$ = 17.1 Hz, 1H), 4.15 (dd, $J$ = 9.2, 6.2 Hz, 3H); **$^{13}$C NMR** (126 MHz, chloroform-*d*) δ 143.1, 139.5, 133.3, 130.0, 127.6, 125.6, 120.7, 115.36, 63.5, 47.5.

*(4R,4'S,5S)-2,2,2',2'-tetramethyl-5-((E)-3-methylbuta-1,3-dien-1-yl)-4,4'-bi(1,3-dioxolane) (**4ba**):*

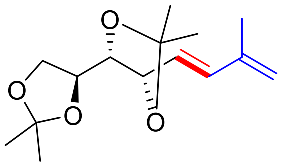


**L-Arabinose**

Following the **general procedure D**, the title compound was obtained from the reaction of hydrazone of **1ai** (0.2 mmol, 1.0 M generated *in situ* from **1ai** and hydrazine monohydrate) with **2ba** (0.6 mmol). $Cs_2CO_3$ was replaced by NaOH (16 mg, 0.4 mmol) and the reaction was

performed at 60 °C for 18 hr and the product was isolated by column chromatography on silica gel. **4ba**: yield 50%, colorless oil; $R_f$ = 0.28 (hexane/EtOAc, 20:1); E/Z = 20:1 (The E/Z ratio was determined based on analysis of the $^1$H-NMR spectrum); $[\alpha]_D^{22}$ = +19.2 (c = 0.5, $CHCl_3$); **$^1$H NMR** (500 MHz, chloroform-*d*) δ 6.45 (d, *J* = 15.7 Hz, 1H), 5.67 (dd, *J* = 15.7, 6.7 Hz, 1H), 5.01 (s, 2H), 4.42 (t, *J* = 7.6 Hz, 1H), 4.14 (m, 1H), 4.09 (dd, *J* = 8.5, 6.3 Hz, 1H), 3.97 (dd, *J* = 8.4, 4.7 Hz, 1H), 3.73 (dd, *J* = 7.9, 7.0 Hz, 1H), 1.85 (s, 3H), 1.43 (s, 3H), 1.42 (s, 3H), 1.39 (s, 3H), 1.34 (s, 3H); **$^{13}$C NMR** (126 MHz, chloroform-*d*) δ 141.2, 135.5, 126.7, 117.4, 109.6, 109.4, 81.3, 80.1, 66.9, 27.1, 26.9, 26.7, 25.3, 18.5; **HRMS**: calcd for $C_{15}H_{24}O_4$ ($[M + Na]^+$): 291.1567, found: 291.1572.

*(4R,4'S,5S)-5-((E)-3-benzylbuta-1,3-dien-1-yl)-2,2,2',2'-tetramethyl-4,4'-bi(1,3-dioxolane) (**4bb**):*

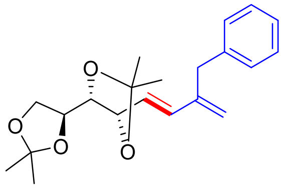


**L-Arabinose**

Following the **general procedure D**, the title compound was obtained from the reaction of hydrazone of **1ai** (0.2 mmol, 1.0 M generated *in situ* from **1ai** and hydrazine monohydrate) with **2bm** (0.6 mmol). $Cs_2CO_3$ was replaced by $^t$BuOLi (32 mg, 0.4 mmol) and the reaction was performed at 60 °C for 18 hr and the product was isolated by column chromatography on silica gel. **4bb**: yield 30%, colorless oil; $R_f$ = 0.25 (hexane/EtOAc, 20:1); E/Z = 9:1 (The E/Z ratio was determined based on analysis of the $^1$H-NMR spectrum); **$^1$H NMR** (500 MHz, chloroform-*d*) δ 7.28 – 7.25 (m, 2H), 7.21 – 7.16 (m, 3H), 6.45 (d, *J* = 15.8 Hz, 1H), 5.74 (dd, *J* = 15.9, 6.5 Hz, 1H), 5.19 (s, 1H), 4.91 (s, 1H), 4.40 (t, *J* = 7.1 Hz, 1H), 4.10 (td, *J* = 6.6, 4.9 Hz, 1H), 4.04 (dd, *J* = 8.5, 6.3 Hz, 1H), 3.86 (dd, *J* = 8.5, 4.8 Hz, 1H), 3.62 (t, *J* = 7.3 Hz, 1H), 3.55 (s, 2H), 1.40 (s, 3H), 1.39 (s, 3H), 1.32 (s, 3H), 1.431 (s, 3H); **$^{13}$C NMR** (126 MHz, chloroform-*d*) δ 144.1, 134.2, 128.8, 128.3, 127.3, 126.1, 118.8, 109.4, 81.3, 79.9, 66.9, 38.6, 27.0, 26.9, 26.6, 25.2; **HRMS**: calcd for $C_{21}H_{28}O_4$ ($[M + Na]^+$): 367.1880, found: 367.1871.

*Estrone derivative (**5aa**):*

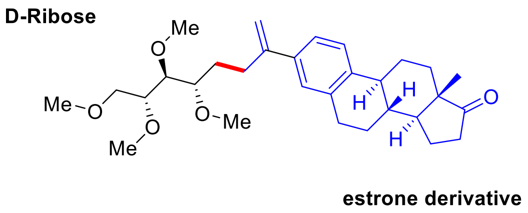


Following the **general procedure C**, the title compound was obtained from the reaction of hydrazone of **1aa** (0.24 mmol, 1.0 M generated *in situ* from **1aa** and hydrazine monohydrate) with **2bv** (0.2 mmol). The reaction mixture was stirred at 45 °C for 3 h and the product was isolated by column chromatography on silica gel. **5aa**: yield 80%, white solid, $R_f$ = 0.45 (hexane/EtOAc, 3:1); **$^1$H NMR** (500 MHz, chloroform-*d*) δ 7.26 – 7.18 (m, 2H), 7.16 (s, 1H), 5.27 (d, *J* = 1.6 Hz, 1H), 5.05 (d, *J* = 1.5 Hz, 1H), 3.61 (dd, *J* = 10.5, 2.7 Hz, 1H), 3.50 – 3.47 (m, 1H), 3.46 (s, 3H), 3.45 (d, *J* = 4.3 Hz, 1H), 3.41 – 3.39 (m, 1H), 3.40 (s, 3H), 3.38 (s, 3H), 3.32 (s, 3H), 3.25 (m, 1H), 2.95 – 2.86 (m, 2H), 2.69 (ddd, *J* = 14.7, 9.7, 5.1 Hz, 1H), 2.59 – 2.47 (m, 2H), 2.46 – 2.38 (m, 1H), 2.30 (m, 1H), 2.18 – 2.10 (m, 1H), 2.04 (m, 2H), 1.99 – 1.94 (m, 1H), 1.76 (m, 1H), 1.68 – 1.58 (m, 3H), 1.56 – 1.42 (m, 4H), 0.91 (s, 3H); **$^{13}$C NMR** (126 MHz, chloroform-*d*) δ 148.1, 138.9, 138.7, 136.2, 126.7, 125.3, 123.6, 111.8, 80.7, 80.3, 79.9, 71.5, 60.0, 59.2, 57.8, 57.7, 50.5, 48.0, 44.4, 38.2, 35.9, 31.6, 31.0, 29.5, 28.3, 26.6, 25.7, 21.6, 13.9; **HRMS**: calcd for $C_{30}H_{44}O_5$ ($[M + Na]^+$): 507.3081, found: 507.3090.

<u>*Estrone derivative (**5ab**):*</u>

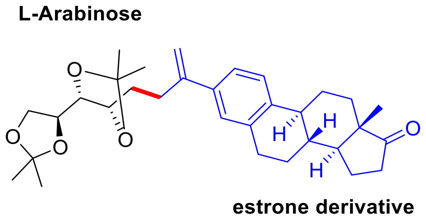


Following the **general procedure C**, the title compound was obtained from the reaction of hydrazone of **1ai** (0.24 mmol, 1.0 M generated *in situ* from **1ai** and hydrazine monohydrate) with **2bv** (0.2 mmol). The reaction mixture was stirred at 45 °C for 3 hr and the product was isolated by column chromatography on silica gel. **5ab**: yield 81%, white solid, $R_f$ = 0.64 (hexane/EtOAc, 3:1); **$^1$H NMR** (500 MHz, chloroform-*d*) δ 7.26 – 7.20 (m, 2H), 7.17 (s, 1H), 5.30 (d, *J* = 1.3 Hz, 1H), 5.06 (d, *J* = 1.5 Hz, 1H), 4.10 (dd, *J* = 8.4, 6.1 Hz, 1H), 4.04 – 3.95 (m, 2H), 3.93 (dd, *J* = 8.4, 5.2 Hz, 1H), 3.58 (t, *J* = 7.7 Hz, 1H), 2.92 (dd, *J* = 9.1, 4.2 Hz, 2H), 2.73 (ddd, *J* = 15.4, 10.7, 4.8 Hz, 1H), 2.58 (td, *J* = 10.1, 5.2 Hz, 1H), 2.51 (dd, *J* = 19.0, 8.7 Hz, 1H), 2.46 – 2.39 (m, 1H), 2.31 (td,

*J* = 10.9, 4.1 Hz, 1H), 2.17 – 2.10 (m, 1H), 2.10 – 2.01 (m, 2H), 2.00 – 1.89 (m, 2H), 1.75 (dddd, *J* = 13.5, 10.5, 8.4, 4.8 Hz, 1H), 1.68 – 1.57 (m, 3H), 1.56 – 1.46 (m, 4H), 1.40 (s, 3H), 1.36 (d, *J* = 6.6 Hz, 6H), 1.32 (s, 3H); **$^{13}$C NMR** (126 MHz, chloroform-*d*) δ 147.6, 139.0, 138.7, 136.2, 126.6, 125.3, 123.6, 111.6, 109.5, 108.9, 81.1, 80.0, 77.2, 67.7, 50.5, 48.0, 44.4, 38.2, 35.9, 32.6, 31.6, 31.4, 29.5, 27.4, 27.1, 26.7, 26.6, 25.7, 25.3, 21.6, 13.9; **HRMS**: calcd for $C_{32}H_{44}O_5$ ([M + Na]$^+$): 531.3081, found: 531.3095.

*Tyrosine derivative (**5ac**):*

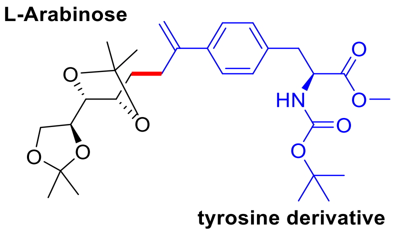


Following the **general procedure C**, the title compound was obtained from the reaction of hydrazone of **1ai** (0.24 mmol, 1.0 M generated *in situ* from **1ai** and hydrazine monohydrate) with **2bw** (0.2 mmol), NaOH (19.2 mg, 0.48 mmol), the reaction mixture was stirred at 45 °C for 3 hr and the product was isolated by column chromatography on silica gel. **5ac**: yield 61%, colorless oil; $R_f$ = 0.35 (hexane/EtOAc, 4:1);**$^1$H NMR** (500 MHz, chloroform-*d*) δ 7.36 (d, *J* = 8.2 Hz, 2H), 7.07 (d, *J* = 7.9 Hz, 2H), 5.30 (s, 1H), 5.08 (s, 1H), 4.96 (d, *J* = 7.7 Hz, 1H), 4.58 (q, *J* = 5.6 Hz, 1H), 4.10 (dd, *J* = 8.4, 6.1 Hz, 1H), 4.03 – 3.95 (m, 2H), 3.92 (dd, *J* = 8.5, 5.2 Hz, 1H), 3.72 (s, 3H), 3.57 (t, *J* = 7.7 Hz, 1H), 3.07 (qd, *J* = 13.8, 5.9 Hz, 2H), 2.72 (ddd, *J* = 15.3, 10.6, 4.8 Hz, 1H), 2.58 (ddd, *J* = 15.6, 10.5, 5.8 Hz, 1H), 1.92 (dddd, *J* = 14.1, 10.8, 5.8, 3.4 Hz, 1H), 1.73 (dddd, *J* = 13.5, 10.5, 8.4, 4.9 Hz, 1H), 1.42 (s, 9H), 1.39 (s, 3H), 1.36 (s, 3H), 1.35 (s, 3H), 1.32 (s, 3H); **$^{13}$C NMR** (126 MHz, chloroform-*d*) δ 172.3, 155.1, 147.4, 139.8, 135.1, 129.2, 126.2, 112.0, 109.5, 108.9, 81.1, 80.0, 67.7, 54.4, 52.2, 37.9, 32.5, 31.3, 28.3, 27.4, 27.1, 26.1, 25.3; **HRMS**: calcd for $C_{29}H_{43}NO_8$ ([M + Na]$^+$): 556.2881, found: 556.2868.

*1-(4-((4R,4'S,5S)-2,2,2',2'-tetramethyl-[4,4'-bi(1,3-dioxolan)]-5-yl)but-1-en-2-yl)-4-(4-((4S,4'S,5R)-2,2,2',2'-tetramethyl-[4,4'-bi(1,3-dioxolan)]-5-yl)but-1-en-2-yl)benzene (**5ad**):*

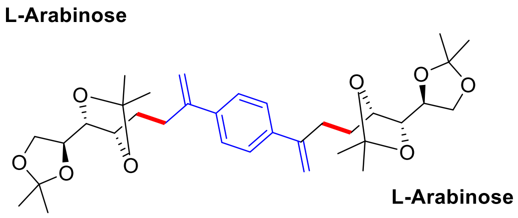

PEPPSI$^{TM}$-IPr catalyst (6.8 mg, 10 mol%), allyl acetate **2bx** (0.1 mmol), and 2-Me-THF (0.26 mL) were added into a dried microwave vial (10 mL) equipped with a stir bar in the glovebox. The reaction mixture was stirred at room temperature for 5 min before hydrazone solution of **1ai** (0.24 mmol, 240 µL, 1 M), NaOH (9.6 mg, 0.24 mmol), and 2-Me-THF (0.5 mL) were added. The reaction tube was sealed and moved out of the glovebox. The resulting mixture was stirred at 45 $^{o}$C for 6 h. After the completion of the reaction, the reaction solution was filtered through a short celite pad and washed with diethyl ether (60 mL). The combined solution was concentrated under vacuum, and the residue was purified by flash column chromatography to give the desired product. **5ad**: yield 63%, colorless oil; $R_f$ = 0.4 (hexane/EtOAc, 4:1); $^{1}$**H NMR** (500 MHz, chloroform-*d*) δ 7.39 (s, 4H), 5.33 (s, 2H), 5.09 (s, 2H), 4.11 (dd, *J* = 8.4, 6.1 Hz, 2H), 4.03 – 3.95 (m, 4H), 3.93 (dd, *J* = 8.4, 5.2 Hz, 2H), 3.57 (t, *J* = 7.8 Hz, 2H), 2.78-2.72 (m, 2H), 2.64-2.58 (m, 2H), 1.97-1.90 (m, 2H), 1.79-1.71 (m, 2H), 1.40 (s, 6H), 1.36 (s, 6H), 1.34 (s, 6H), 1.32 (s, 6H); $^{13}$**C NMR** (126 MHz, chloroform-*d*) δ 147.5, 140.1, 125.9, 112.0, 109.5, 108.9, 81.1, 80.0, 77.2, 67.7, 32.5, 31.4, 27.4, 27.1, 26.7, 25.3; **HRMS**: calcd for $C_{34}H_{50}O_8$ ([M + Na]$^+$): 609.3398, found: 609.3377.

*(3aR,5aS,8aS,8bR)-2,2,7,7-tetramethyl-5-(3-(4-(4-((4R,4'S,5S)-2,2,2',2'-tetramethyl-[4,4'-bi(1,3-dioxolan)]-5-yl)but-1-en-2-yl)phenyl)but-3-en-1-yl)tetrahydro-5H-bis([1,3]dioxolo)[4,5-b:4',5'-d]pyran (**5ae**):*

L-Arabinose

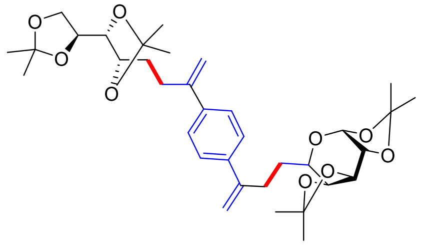

α-D-Galactopyranose

PEPPSI$^{TM}$-IPr catalyst (3.4 mg, 5 mol%), allyl acetate **2bx** (0.1 mmol), and 2-Me-THF (0.26 mL) were added into a dried microwave vial (10 mL) equipped with a stir bar in the glovebox. The reaction mixture was stirred at room temperature for 5 min before hydrazone solution of **1ak** (0.12 mmol, 120 µL, 1 M), NaOH (4.8 mg, 0.12 mmol), and 2-Me-THF (0.5 mL) were added. The reaction tube was sealed and moved out of the glovebox. The mixture was stirred at 45 $^{o}$C for 3 hr. After the reaction tube cooled to room temperature, it was transferred into the glovebox. PEPPSI$^{TM}$-IPr catalyst (3.4 mg, 5 mol%), hydrazone solution of **1ai** (0.12 mmol, 120 µL, 1 M), NaOH (4.8 mg, 0.12 mmol), and 2-Me-THF (0.5 mL) were added. The tube was sealed and moved out of the glovebox. The resulting mixture was stirred at 45 $^{o}$C for 3 hr. After the completion of

the reaction, the reaction solution was filtered through a short celite pad and washed with diethyl ether (60 mL). The combined solution was concentrated under vacuum, and the residue was purified by flash column chromatography to give the desired product. **5ae**: yield 61%, colorless oil; $R_f$ = 0.42 (hexane/EtOAc, 85:15); **$^1$H NMR** (500 MHz, chloroform-*d*) δ 7.38 (d, *J* = 2.1 Hz, 4H), 5.55 (d, *J* = 5.1 Hz, 1H), 5.32 (s, 2H), 5.13 (s, 1H), 5.09 (s, 1H), 4.58 – 4.54 (m, 1H), 4.29 (dd, *J* = 5.2, 2.2 Hz, 1H), 4.13 – 4.06 (m, 2H), 3.99 (m, 2H), 3.92 (dd, *J* = 8.5, 5.2 Hz, 1H), 3.75 (dd, *J* = 9.5, 4.0 Hz, 1H), 3.58 (t, *J* = 7.7 Hz, 1H), 2.78 – 2.71 (m, 2H), 2.64 – 2.53 (m, 2H), 1.96 – 1.84 (m, 2H), 1.79 – 1.73 (m, 1H), 1.71 – 1.65 (m, 1H), 1.52 (s, 3H), 1.45 (s, 3H), 1.40 (s, 3H), 1.36 (s, 3H), 1.34 (s, 6H), 1.32 (s, 3H), 1.31 (s, 3H); **$^{13}$C NMR** (126 MHz, chloroform-*d*) δ 147.5, 147.1, 140.1, 139.9, 125.98, 125.96, 112.8, 112.0, 109.5, 109.0, 108.9, 108.3, 96.6, 81.1, 80.0, 77.2, 73.0, 71.0, 70.5, 67.7, 66.6, 32.5, 31.4, 31.0, 28.6, 27.4, 27.1, 26.7, 26.1, 26.0, 25.3, 25.0, 24.4; **HRMS**: calcd for $C_{35}H_{50}O_9$ ([M + Na]$^+$): 637.3347, found: 637.3332.

*(R)-6-((5S,6S,7R,E)-5,6,7,8-tetramethoxyoct-1-en-1-yl)-5,6-dihydro-2H-pyran-2-one (**5af**):*

**D-Ribose**

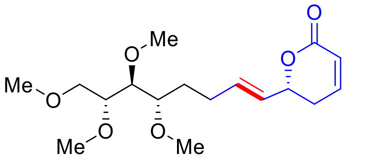

Grubbs catalyst 2$^{nd}$ generation (4.3 mg, 0.005 mmol) was added to stirred solution of **2by** (24.8 mg, 0.2 mmol) and **3aa** (23.2 mg, 0.1 mmol) in DCM (20 mL). The resulting solution was heated and stirred at 40 $^o$C for 15 hr. After the completion of the reaction, the reaction solution was filtered through a short celite pad and washed with EtOAc (60 mL). The combined solution was concentrated under vacuum, and the residue was purified by flash column chromatography to give the desired product. **5af**: yield 75%, colorless oil; $R_f$ = 0.25 (hexane/EtOAc, 1:1); $[\alpha]_D^{20}$ = +53.3 (c = 0.2, $CHCl_3$); **$^1$H NMR** (500 MHz, chloroform-*d*) δ 6.86 (dt, *J* = 9.7, 4.2 Hz, 1H), 6.03 (dt, *J* = 9.7, 1.8 Hz, 1H), 5.86 (dq, *J* = 13.9, 7.6, 7.1 Hz, 1H), 5.62 (dd, *J* = 15.4, 6.8 Hz, 1H), 4.87 (q, *J* = 7.3 Hz, 1H), 3.63 (dd, *J* = 10.5, 2.8 Hz, 1H), 3.50 (dd, *J* = 10.6, 4.8 Hz, 1H), 3.46 (s, 3H), 3.45 – 3.43 (m, 1H), 3.40 (s, 3H), 3.39 (s, 3H), 3.37 (s, 3H), 3.36 – 3.29 (m, 2H), 2.42 (ddd, *J* = 7.4, 3.9, 1.9 Hz, 2H), 2.31 – 2.23 (m, 1H), 2.14 (m, 1H), 1.68 (m, 1H), 1.58 (m, 1H). **$^{13}$C NMR** (126 MHz, chloroform-*d*) δ 164.1, 144.6, 135.4, 126.9, 121.6, 80.7, 80.2, 80.0, 78.2, 71.30, 60.0, 59.2, 57.8, 57.7, 29.8, 28.8, 28.3. **HRMS**: calcd for $C_{17}H_{28}O_6$ ([M + Na]$^+$): 351.1778, found: 351.1784.

*(R)-6-((E)-4-((4R,4'S,5S)-2,2,2',2'-tetramethyl-[4,4'-bi(1,3-dioxolan)]-5-yl)but-1-en-1-yl)-5,6-dihydro-2H-pyran-2-one (**5ag**):*

L-Arabinose

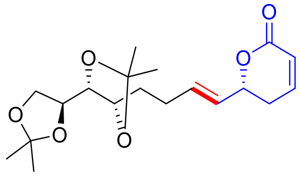


Grubbs catalyst 2nd generation (4.3 mg, 0.005 mmol) was added to stirred solution of **2by** (24.8 mg, 0.2 mmol) and **3ah** (25.6 mg, 0.1 mmol) in DCM (20 mL). The resulting solution was heated and stirred at 40 °C for 15 hr. After the completion of the reaction, the reaction solution was filtered through a short celite pad and washed with EtOAc (60 mL). The combined solution was concentrated under vacuum, and the residue was purified by flash column chromatography to give the desired product. **5ag**: yield 80%, colorless oil; $R_f$ = 0.48 (hexane/EtOAc, 1:1); $[\alpha]_D^{20}$ = +36.1 (c = 0.1, $CHCl_3$); **$^1$H NMR** (500 MHz, chloroform-*d*) δ 6.87 (dt, *J* = 9.7, 4.2 Hz, 1H), 6.04 (dt, *J* = 9.8, 1.8 Hz, 1H), 5.91 – 5.82 (m, 1H), 5.63 (ddt, *J* = 15.5, 6.7, 1.5 Hz, 1H), 4.88 (q, *J* = 7.3 Hz, 1H), 4.12 (dd, *J* = 8.4, 6.1 Hz, 1H), 4.03 – 3.97 (m, 1H), 3.97 – 3.88 (m, 2H), 3.53 (t, *J* = 7.9 Hz, 1H), 2.42 (ddd, *J* = 7.6, 4.7, 1.9 Hz, 2H), 2.35 – 2.27 (m, 1H), 2.25 – 2.16 (m, 1H), 1.85 (dddd, *J* = 13.6, 9.8, 6.2, 3.3 Hz, 1H), 1.69 – 1.61 (m, 1H), 1.40 (s, 3H), 1.38 (s, 3H), 1.34 (s, 6H). **$^{13}$C NMR** (126 MHz, chloroform-*d*) δ 164.0, 144.6, 134.8, 127.1, 121.6, 109.6, 108.9, 81.1, 79.9, 78.1, 77.2, 67.8, 32.7, 29.8, 28.6, 27.4, 27.0, 26.8, 25.3. **HRMS**: calcd for $C_{19}H_{28}O_6$ ([M + Na]$^+$): 375.1778, found: 375.1765.

*(2R,3S,4R,5S,E)-9-((R)-6-oxo-3,6-dihydro-2H-pyran-2-yl)non-8-ene-2,3,4,5-tetrayl tetraacetate* ***(5ah)****:*

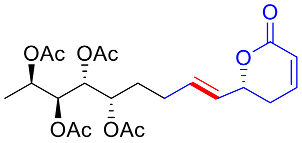


Grubbs catalyst (the 2nd generation, 4.3 mg, 0.005 mmol) was added to stirred solution of **2by** (24.8 mg, 0.2 mmol) and O-acetylated **3am** (35.8 mg, 0.1 mmol) in DCM (20 mL). The resulting solution was heated and stirred at 40 °C for 15 hr. After the completion of the reaction, the reaction solution was filtered through a short celite pad and washed with EtOAc (60 mL). The combined solution was concentrated under vacuum, and the residue was purified by flash column chromatography to give the desired product. **5ah**: yield 72%, colorless oil; $R_f$ = 0.26 (hexane/EtOAc, 1:1); $[\alpha]_D^{20}$ = +52.6 (c = 0.1, $CHCl_3$); **$^1$H NMR** (500 MHz, chloroform-*d*) δ 6.86 (dt, *J* = 9.7, 4.3 Hz, 1H), 6.03 (dt, *J* = 9.7, 1.8 Hz, 1H), 5.81 – 5.73 (m, 1H), 5.59 (ddt, *J* = 13.9,

4.9, 1.3 Hz, 1H), 5.27 (dd, $J$ = 10.0, 1.9 Hz, 1H), 5.13 (dd, $J$ = 10.0, 2.0 Hz, 1H), 5.11 – 5.03 (m, 2H), 4.85 (q, $J$ = 7.1 Hz, 1H), 2.41 (ddd, $J$ = 8.4, 3.9, 2.0 Hz, 2H), 2.11 (t, $J$ = 2.6 Hz, 6H), 2.06 (d, $J$ = 7.2 Hz, 6H), 2.03 (d, $J$ = 4.7 Hz, 1H), 1.58 – 1.46 (m, 2H), 1.12 (d, $J$ = 6.5 Hz, 3H). **$^{13}$C NMR** (126 MHz, chloroform-*d*) δ 170.7, 170.4, 170.1, 170.0, 144.5, 133.40, 127.8, 121.6, 77.8, 70.5, 69.8, 69.7, 67.1, 30.1, 29.7, 27.9, 21.1, 21.0, 20.70, 20.66, 16.3. **HRMS**: calcd for $C_{22}H_{30}O_{10}$ ([M + K]$^+$): 493.1471, found: 493.1464.

## *VI. Preliminary mechanistic studies*

### 1. Verification experiment of carbene process

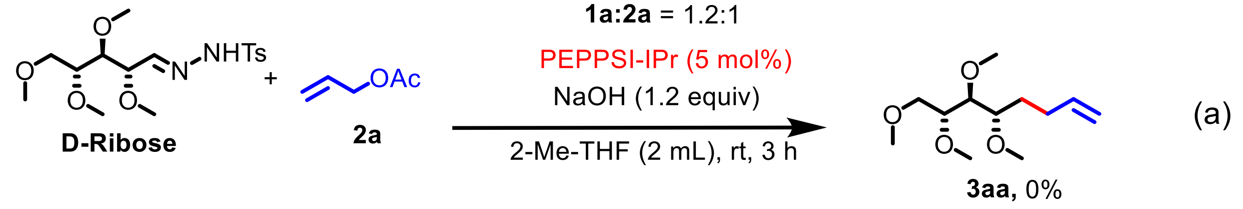


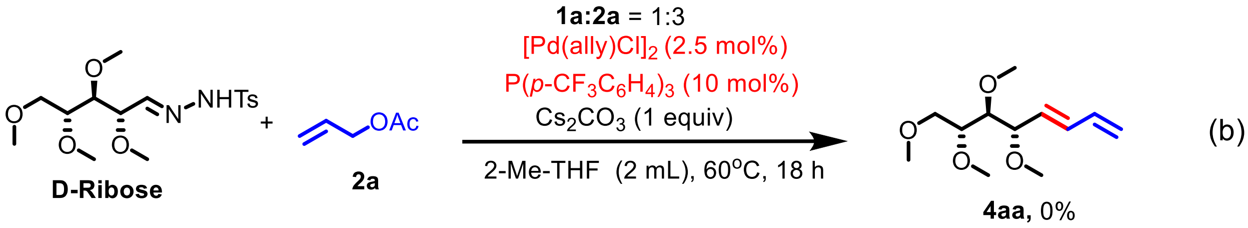


To verify whether the palladium-catalyzed deoxygenative allylation and olefination went through a carbene process, two experiments were carried out using *N*-tosylhydrazone of **1aa** to react with allyl acetate **2a** following **general procedure C** (Eq. a**)** and **general procedure D** **(**Eq. b**)**. No desired product **3aa** or **4aa** was observed, suggesting the unlikely involvement of a carbene process for the two reactions.

### 2. Verification experiment of C-H activation

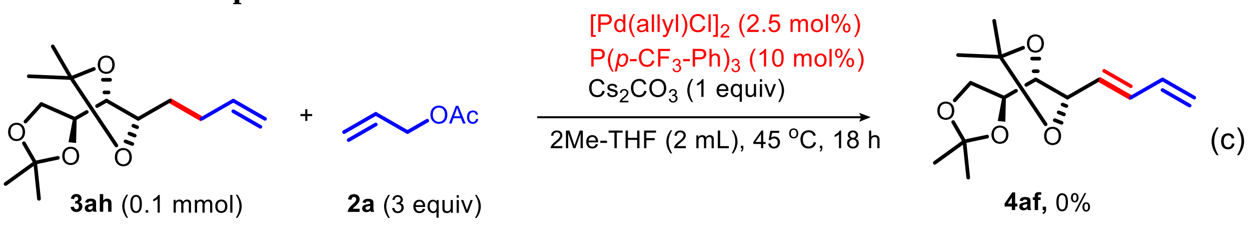

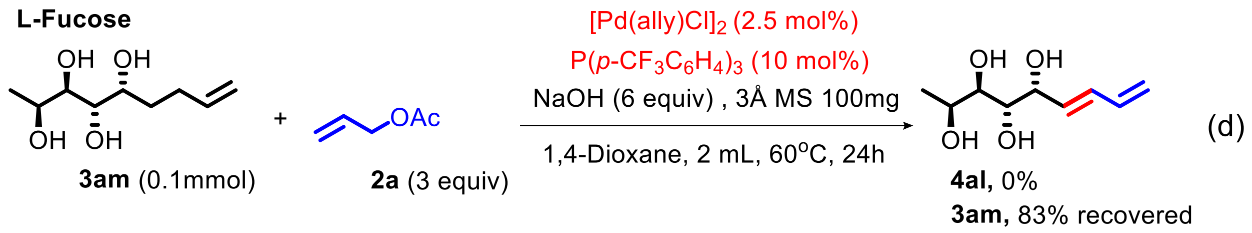


Another possible pathway to form diene product is the palladium-catalyzed C-H activation at the α position of the alkene double bond, then generate the desired product by β-H elimination. Experiments were carried out employing **3ah** and **3am** as substrates to react with allyl acetate **2a** following **general procedure D (**Eq. c and d**)**. No desired product was observed, suggesting the unlikely involvement of a C-H activation process for the reaction.

### 3. Deuterium-labelling study

Hydrazone **1ar** solution was prepared according to the following procedure: In a glovebox, to a mixture of hydrazine hydrate-$d_6$ (73 mg, 1.3 mmol, 1.2 equiv) in 2-Me-THF (0.4 mL), protected monosaccharide **1ai** (1.0 mmol, 1.0 equiv) in 2-Me-THF (0.3 mL) was added dropwise. The mixture was stirred at room temperature for 90 min before anhydrous $Na_2SO_4$ (100 mg) was added. The resulting mixture was further stirred for 15 min. After that, the solution was transferred into a 1.0 mL volumetric flask and was diluted to 1.0 mL with 2-Me-THF. The hydrazone solution was stored in a freezer and used directly for the next step.

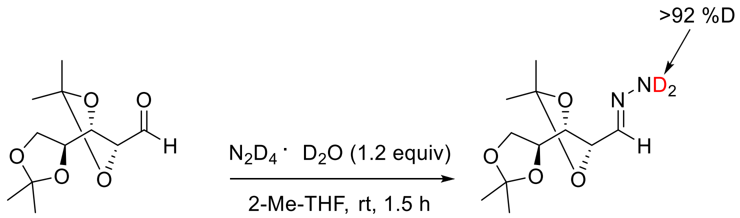


**L-Arabinose**
**1ai**

**L-Arabinose**
**1ar**

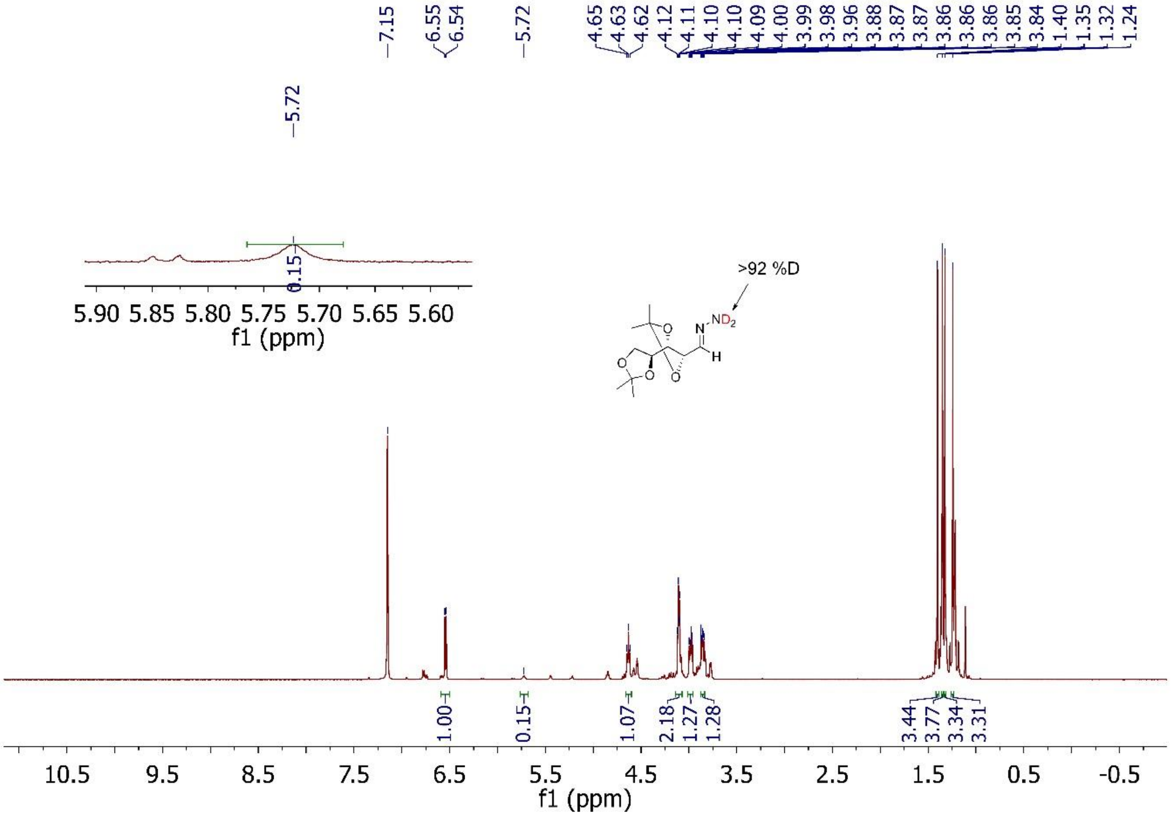

**Palladium-catalyzed deoxygenative allylation of 1ar with 2bc.** Following the **general procedure C**, the reaction was stirred at 45 °C for 3 hr and the product was isolated by column chromatography on silica gel. **3bw**: yield 61%; **$^1$H NMR** (500 MHz, Acetone-$d_6$) δ 7.47 (d, *J* = 7.2 Hz, 2H), 7.34 (t, *J* = 7.6 Hz, 2H), 7.27 (t, *J* = 7.3 Hz, 1H), 5.32 (s, 1H), 5.12 (s, 1H), 4.07 (dd, *J* = 8.3, 6.2 Hz, 1H), 4.03 – 3.93 (m, 2H), 3.84 (dd, *J* = 8.4, 5.2 Hz, 1H), 3.55 (t, *J* = 7.7 Hz, 1H), 2.75 (dd, *J* = 14.6, 9.3 Hz, 1H), 2.64 (t, *J* = 7.5 Hz, 1H), 1.90 (d, *J* = 3.4 Hz, 0.79H), 1.70 (tdd, *J* = 10.5, 8.5, 5.1 Hz, 0.76H), 1.33 (d, *J* = 13.2 Hz, 6H), 1.27 (d, *J* = 9.0 Hz, 6H); Deuterium incorporation was determined by $^1$HNMR.

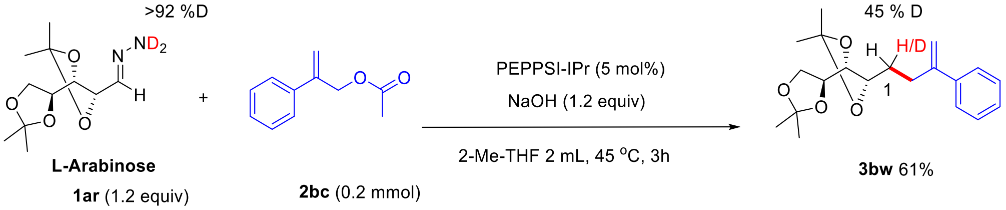

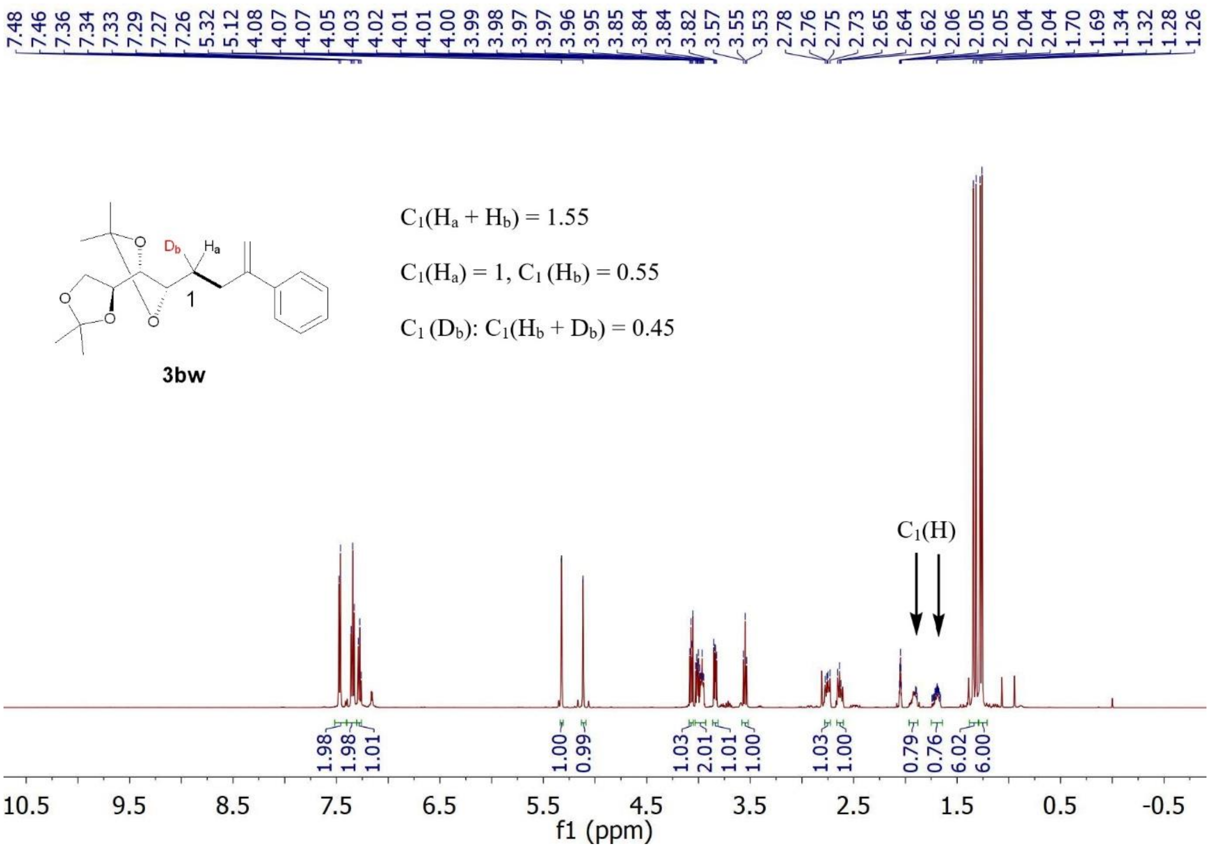


**Palladium-catalyzed deoxygenative olefination of 1ar with 2a.** Following the **general procedure D**, the product was isolated by column chromatography on silica gel. **4af**: yield 45%, colorless oil; **3aq**: yield 14%; colorless oil. No obvious deuterium incorporation was observed by $^{1}$HNMR.

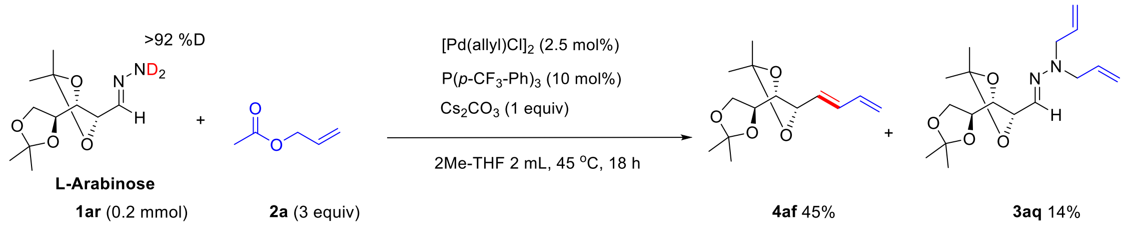

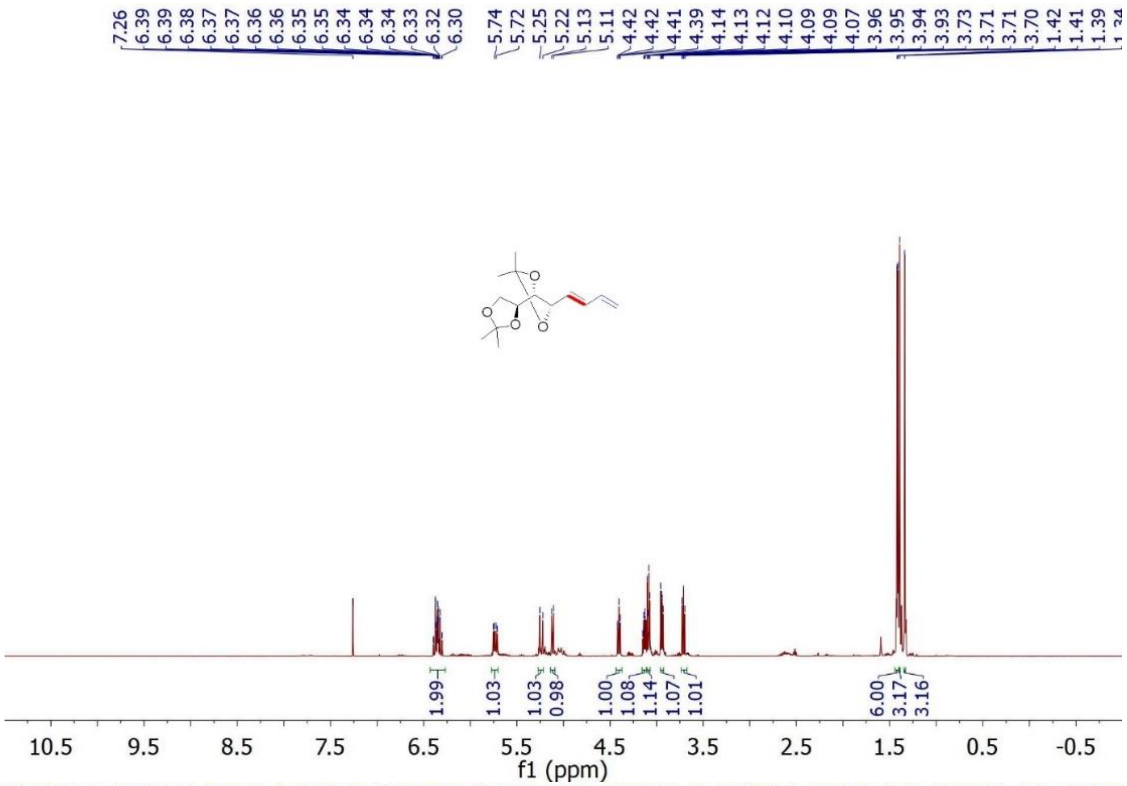
7.26 6.39 6.39 6.38 6.37 6.37 6.36 6.36 6.35 6.34 6.34 6.34 6.33 6.32 6.30 5.74 5.72 5.25 5.22 5.13 5.11 4.42 4.42 4.41 4.39 4.14 4.13 4.12 4.10 4.09 4.07 3.96 3.95 3.94 3.93 3.73 3.71 3.71 3.70 1.42 1.41 1.39
1.99 1.03 1.03 0.98 1.00 1.08 1.14 1.07 1.01 6.00 3.17 3.16
10.5 9.5 8.5 7.5 6.5 5.5 4.5 3.5 2.5 1.5 0.5 -0.5
f1 (ppm)

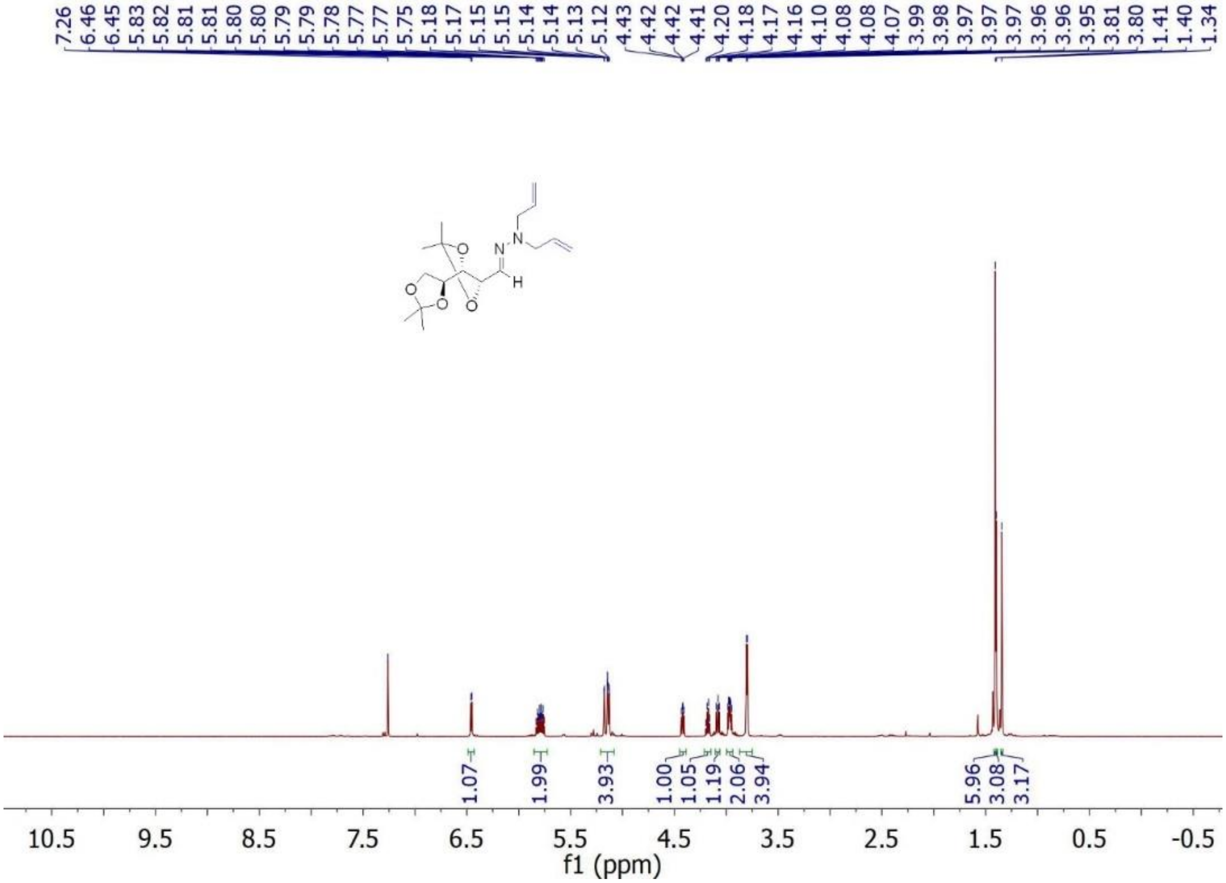
7.26 6.46 6.45 5.83 5.82 5.81 5.81 5.80 5.80 5.79 5.79 5.78 5.77 5.77 5.75 5.18 5.17 5.15 5.15 5.14 5.13 5.12 4.43 4.42 4.42 4.41 4.20 4.18 4.17 4.16 4.10 4.08 4.08 4.07 3.99 3.98 3.97 3.97 3.97 3.96 3.95 3.81 3.80 1.41 1.40 1.34
1.07 1.99 3.93 1.00 1.05 1.19 2.06 3.94 5.96 3.08 3.17
10.5 9.5 8.5 7.5 6.5 5.5 4.5 3.5 2.5 1.5 0.5 -0.5
f1 (ppm)

**Palladium-catalyzed deoxygenative allylation of 1ar with 2a.** Following the **general procedure C**, the reaction mixture was stirred at 45 °C for 3 hr and the product was isolated by column chromatography on silica gel to give a mixture of **3ap** and **4af**: yield 61%; Deuterium incorporation was observed for **3ap** by ¹HNMR.

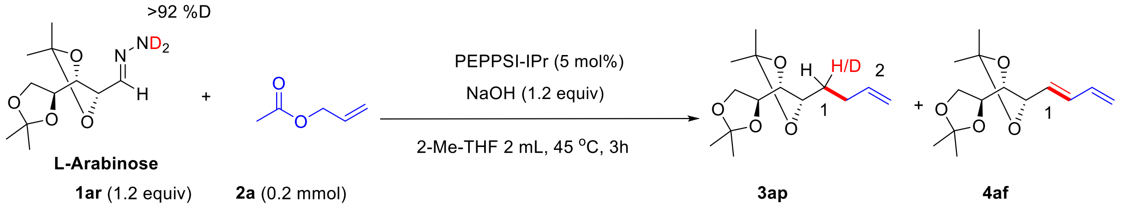


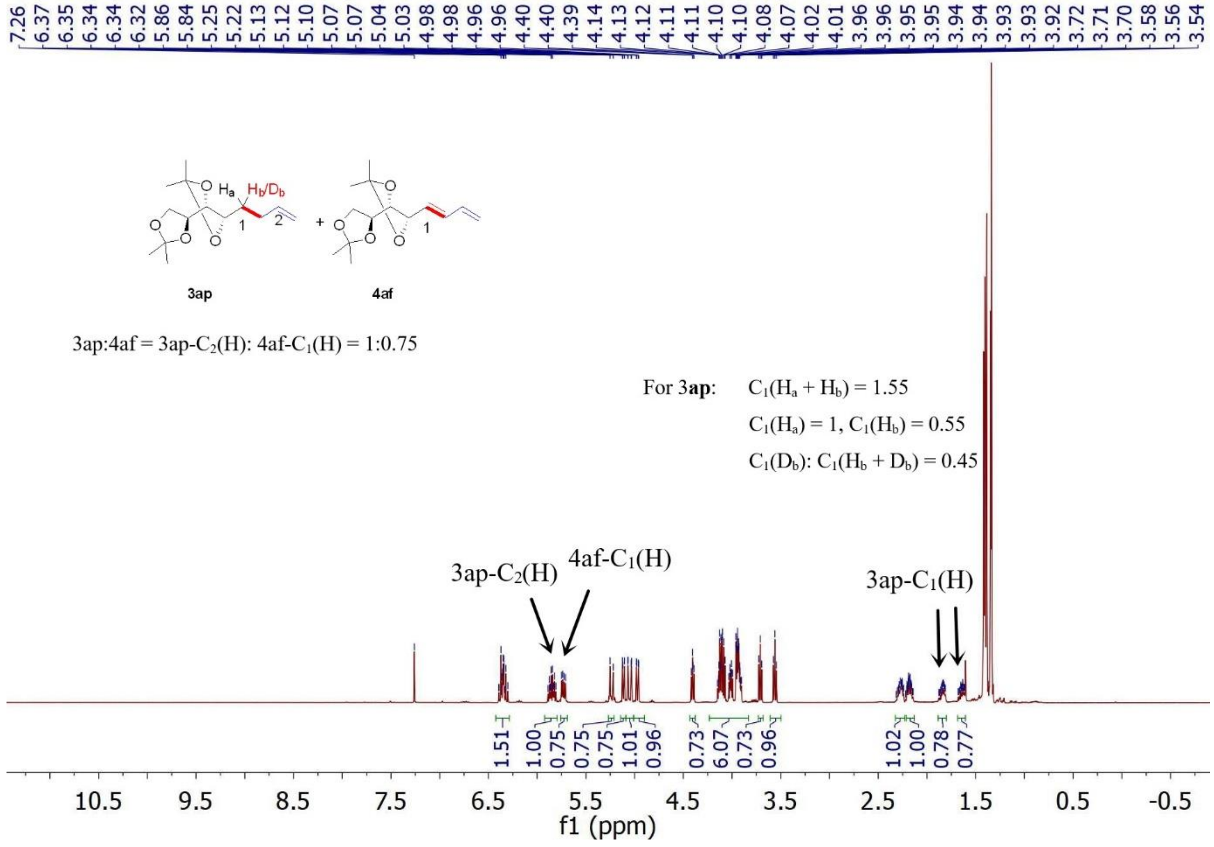

## 4. Propylene Detecting Experiments

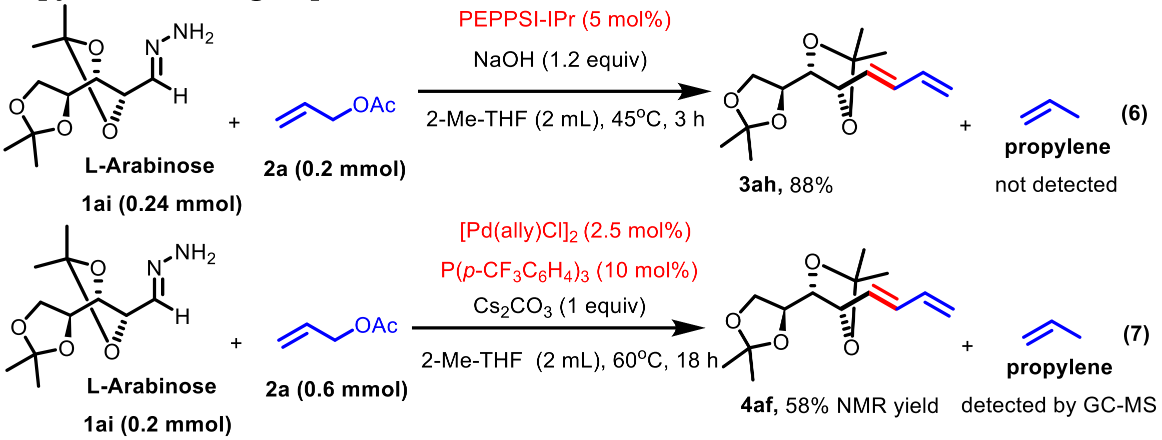

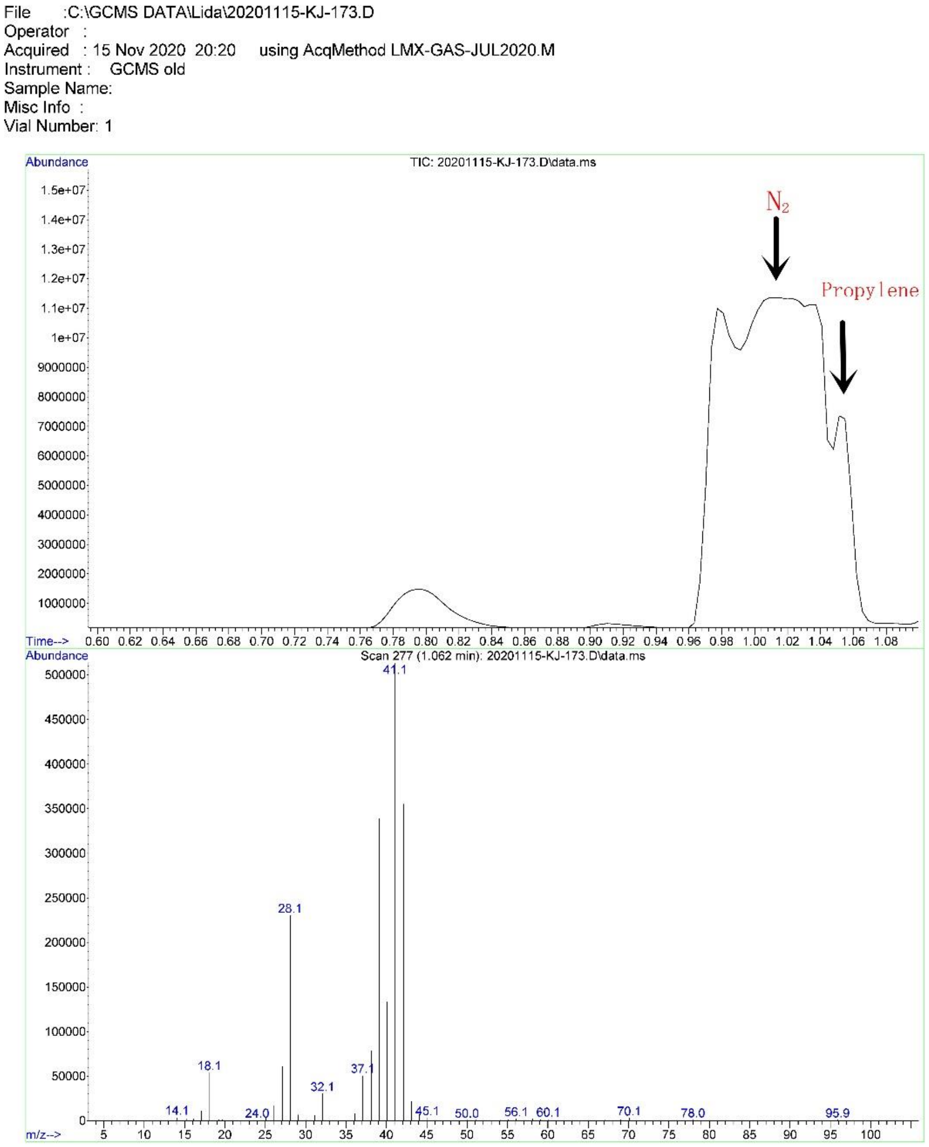


## 5. Possible intermediate

$PdCl_2$ (178 mg, 0.1 mmol, 100 mol%), tris(4-trifluoromethylphenyl)phosphine (93.3 mg, 0.2 mmol, 200 mol%), 2-Me-THF (0.5 mL) were added into a dried microwave vial (10 mL) equipped with a stir bar in the glovebox. The mixture was stirred at room temperature for 15 min before hydrazine solution of **1as** or **1at** (0.1 mmol, 1M) and $Cs_2CO_3$ (32.6 mg, 0.1 mmol) were added. The reaction tube was sealed and moved out of the glovebox. The resulting mixture was stirred at 60 $^{o}$C for 18 hr. After the completion of the reaction, the reaction solution was filtered through a short celite pad and washed with ether (60 mL). The combined solution was concentrated under vacuum, and the residue was diluted by $CDCl_3$ to run the $^1$HNMR test to determine the yield.

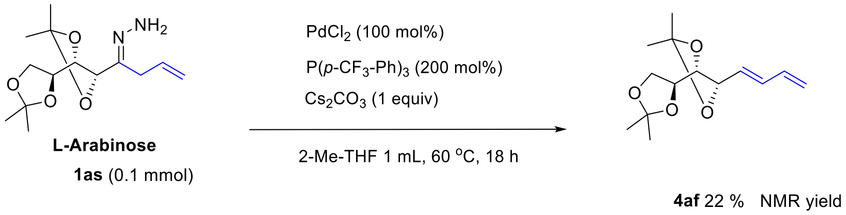


## *VII. Spectral data for representative compounds*

## 2,3,4,5-Tetra-*O*-methyl-D-ribose (1aa):

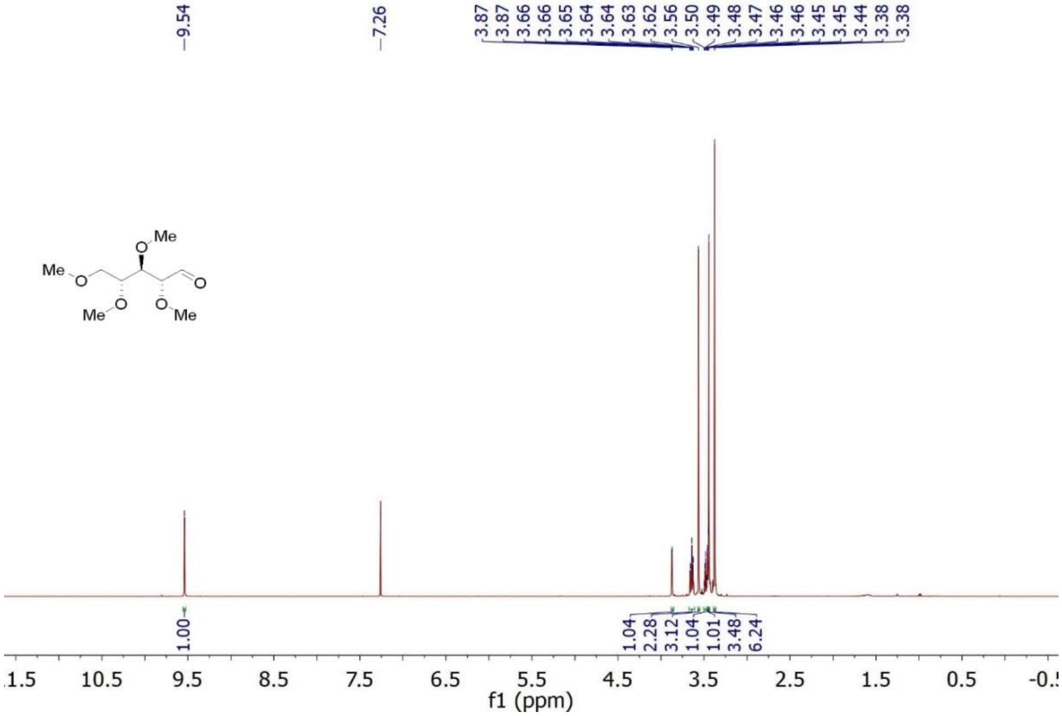

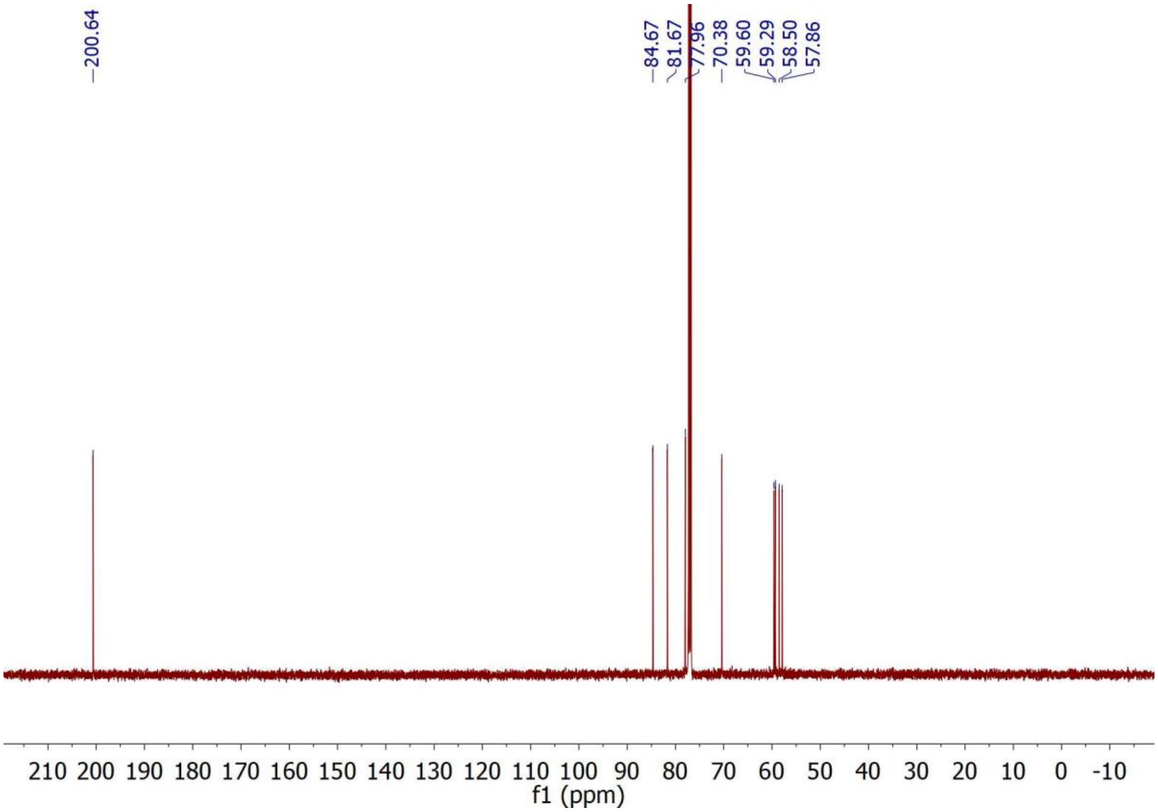


**2,3,4,5-Tetra-*O*-methyl-L-arabinose (1ab):**

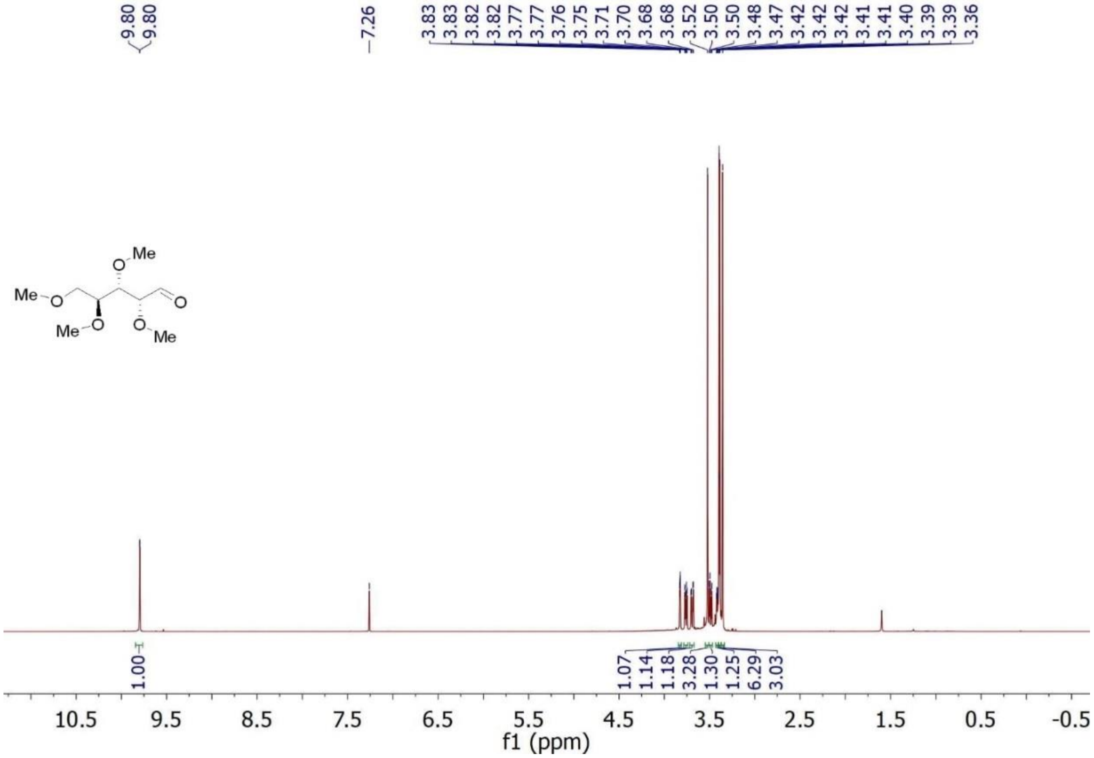

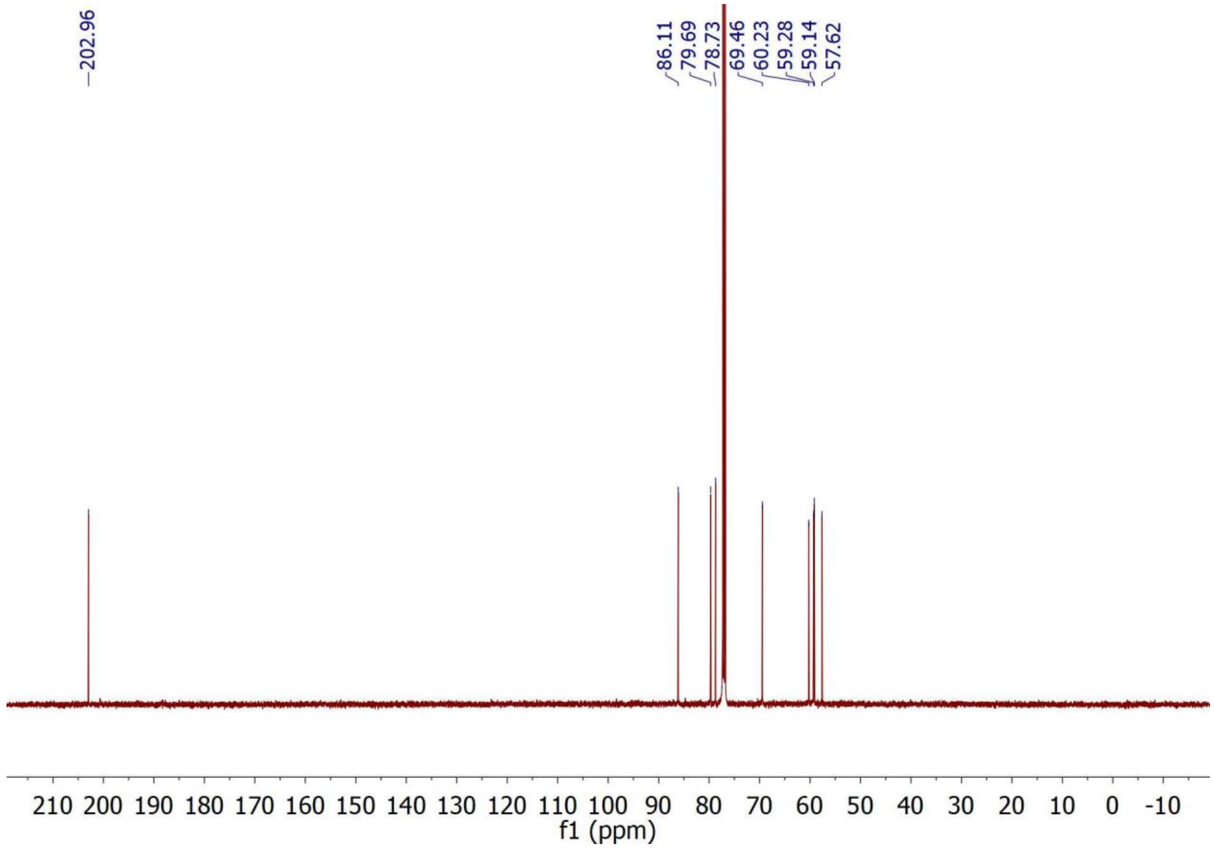


**2,3,4,5-Tetra-*O*-methyl-D-xylose (1ac):**

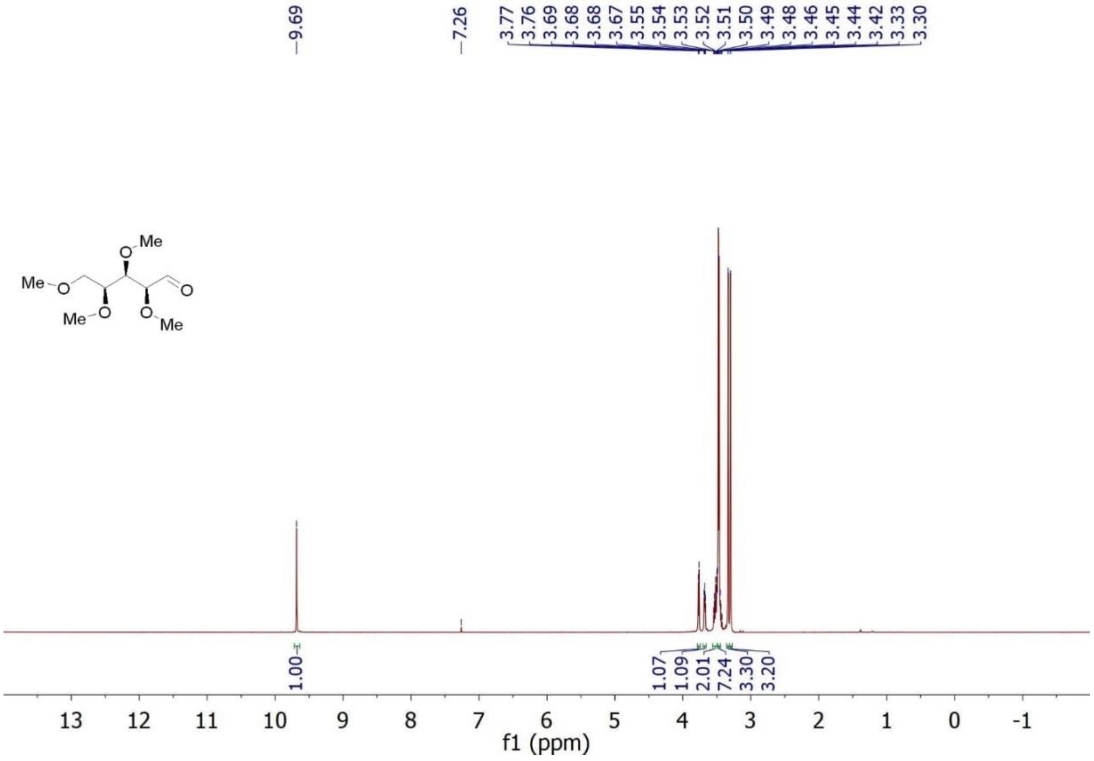

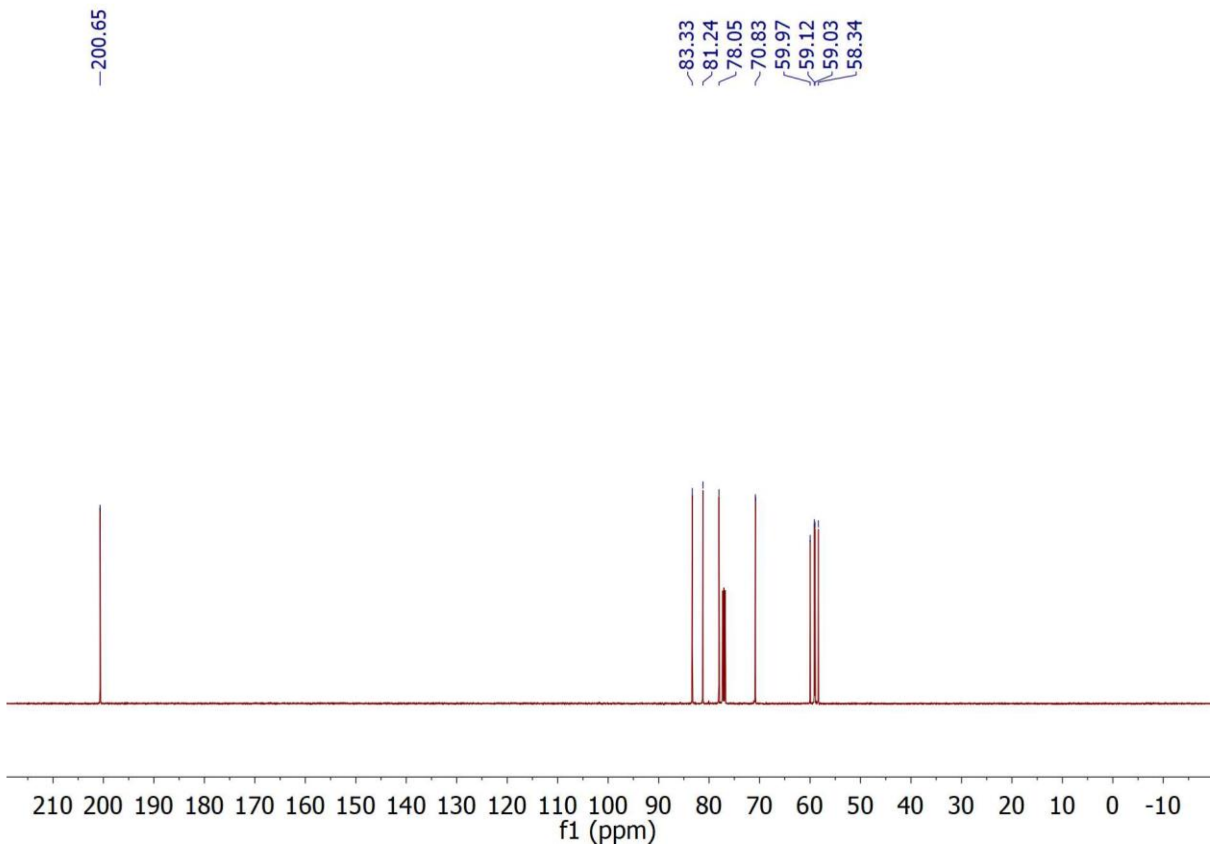


**2,3,4,5,6-Penta-*O*-methyl-D-galactose (1ad):**

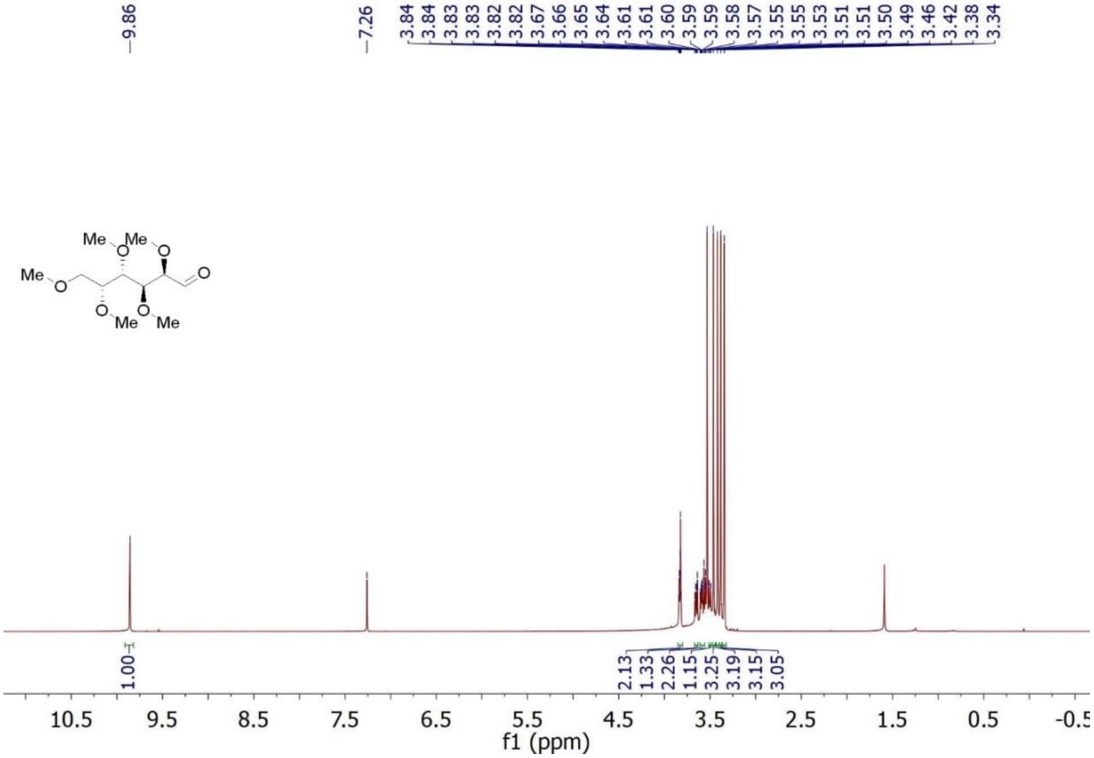

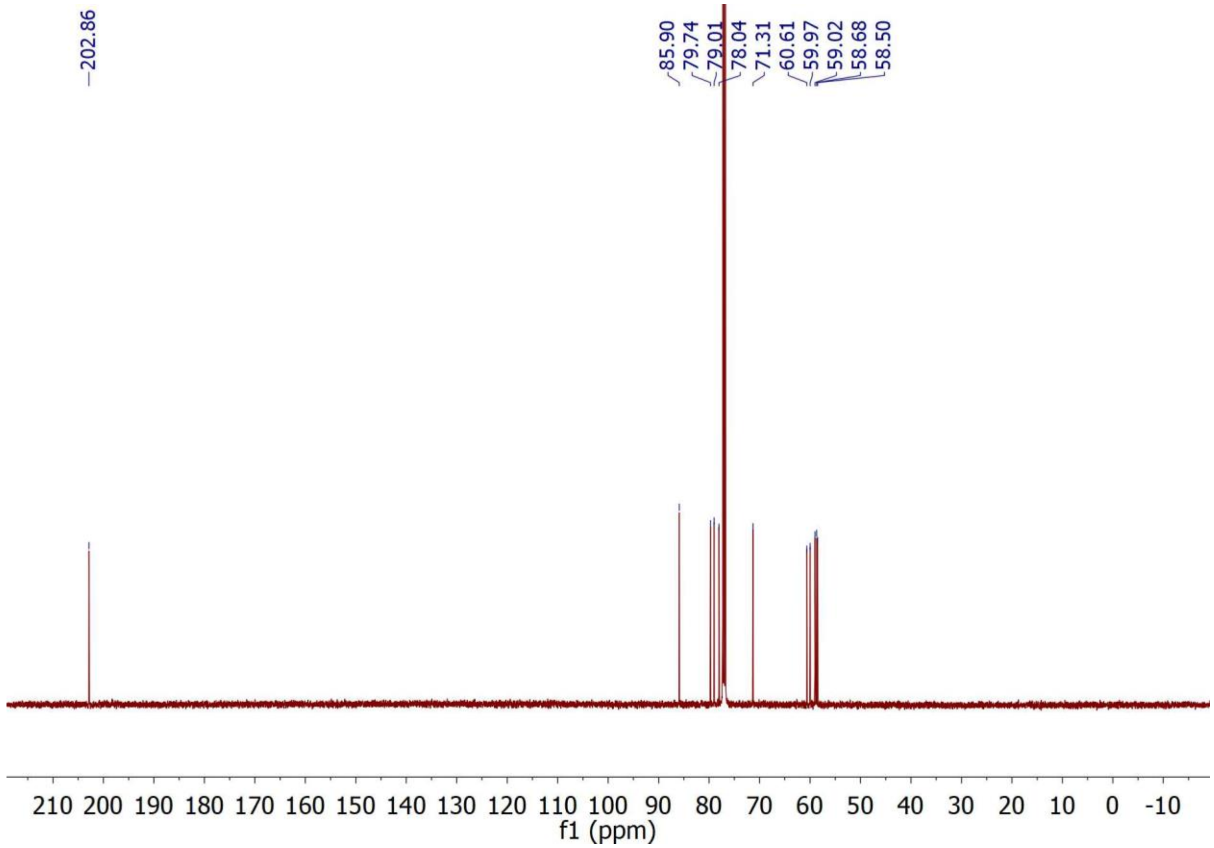


**2,3,4,5,6-Penta-*O*-methyl-D-glucose (1ae):**

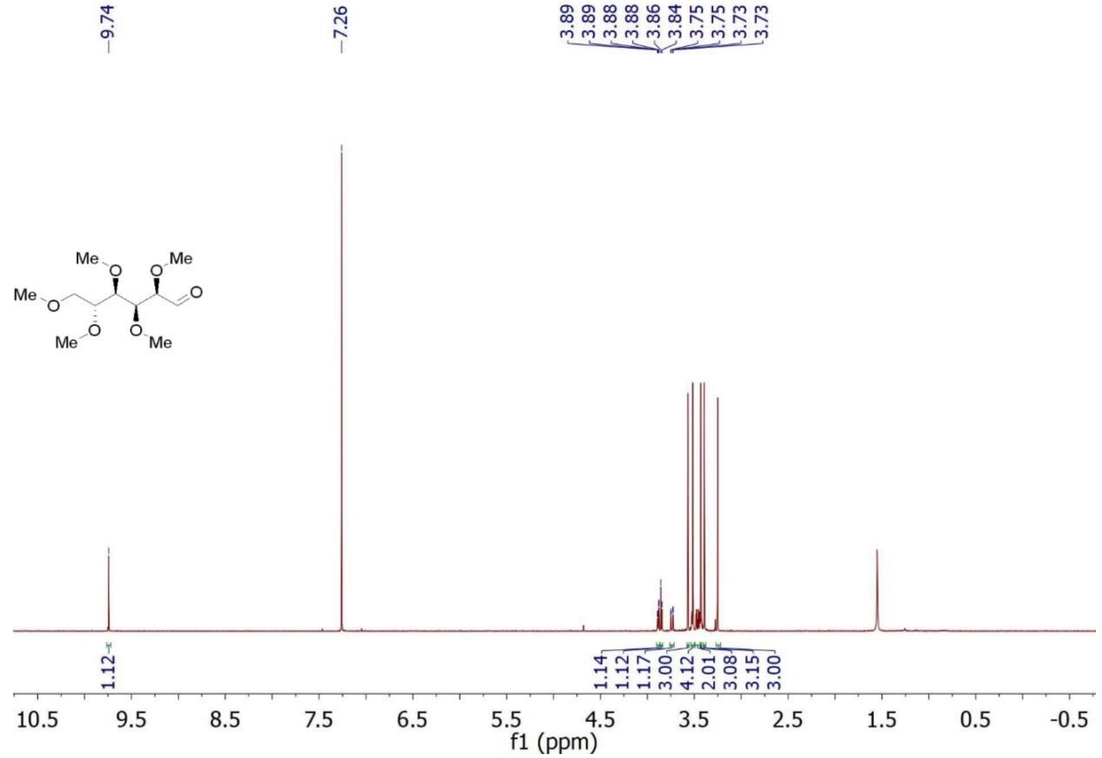

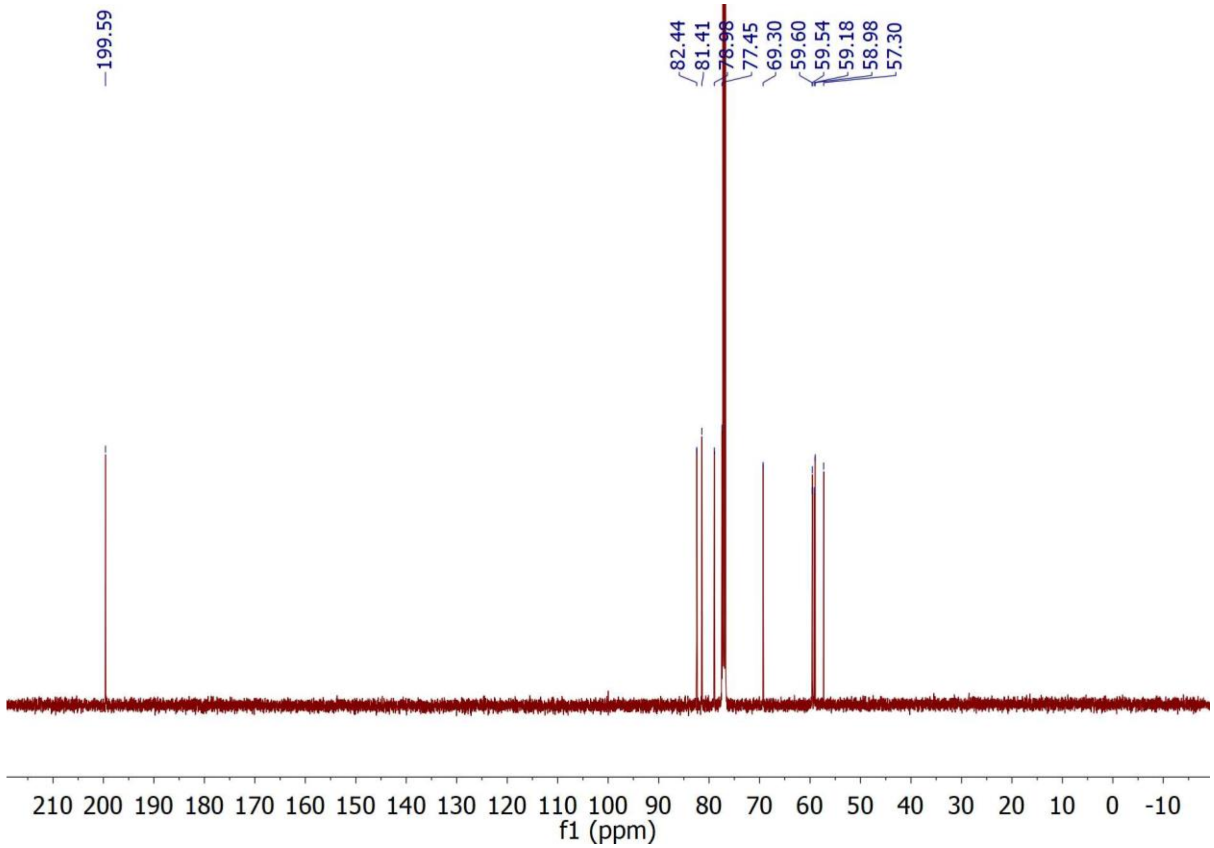


**6-Deoxy-2,3,4,5-tetra-*O*-methyl-L-fucose (1af):**

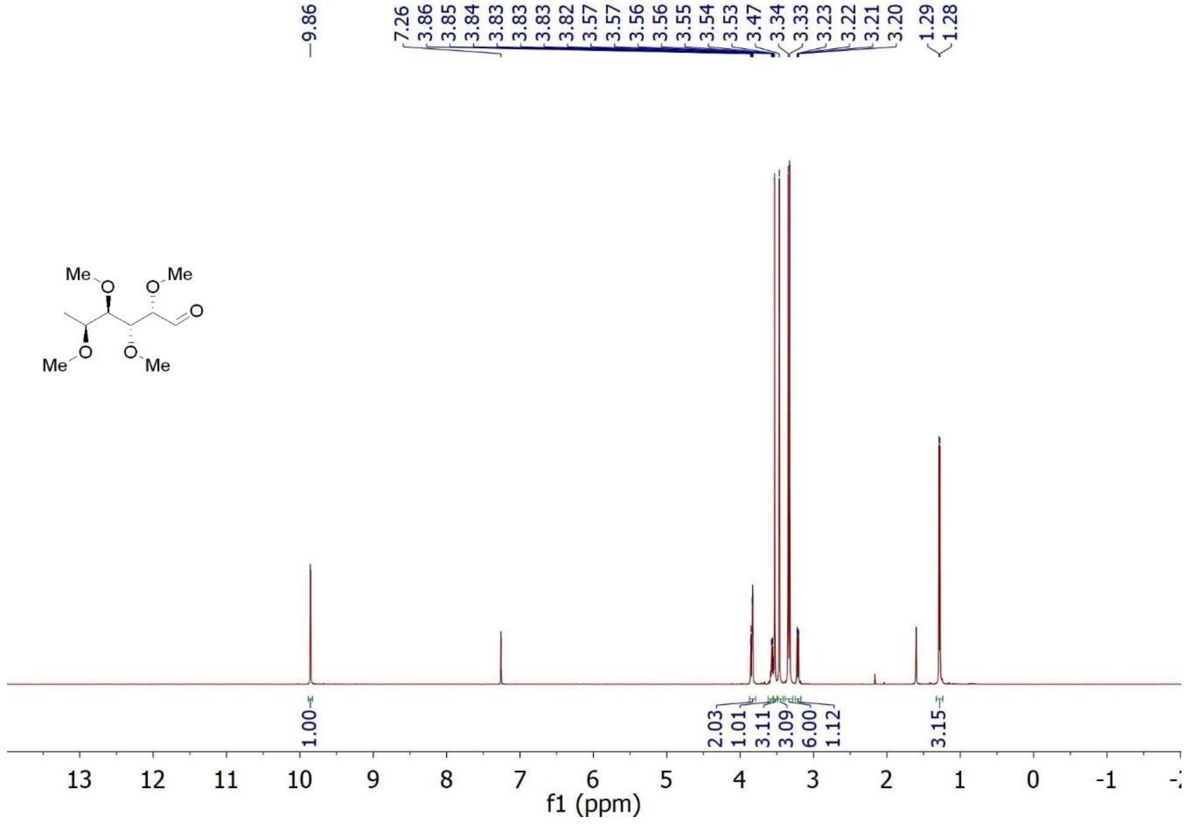

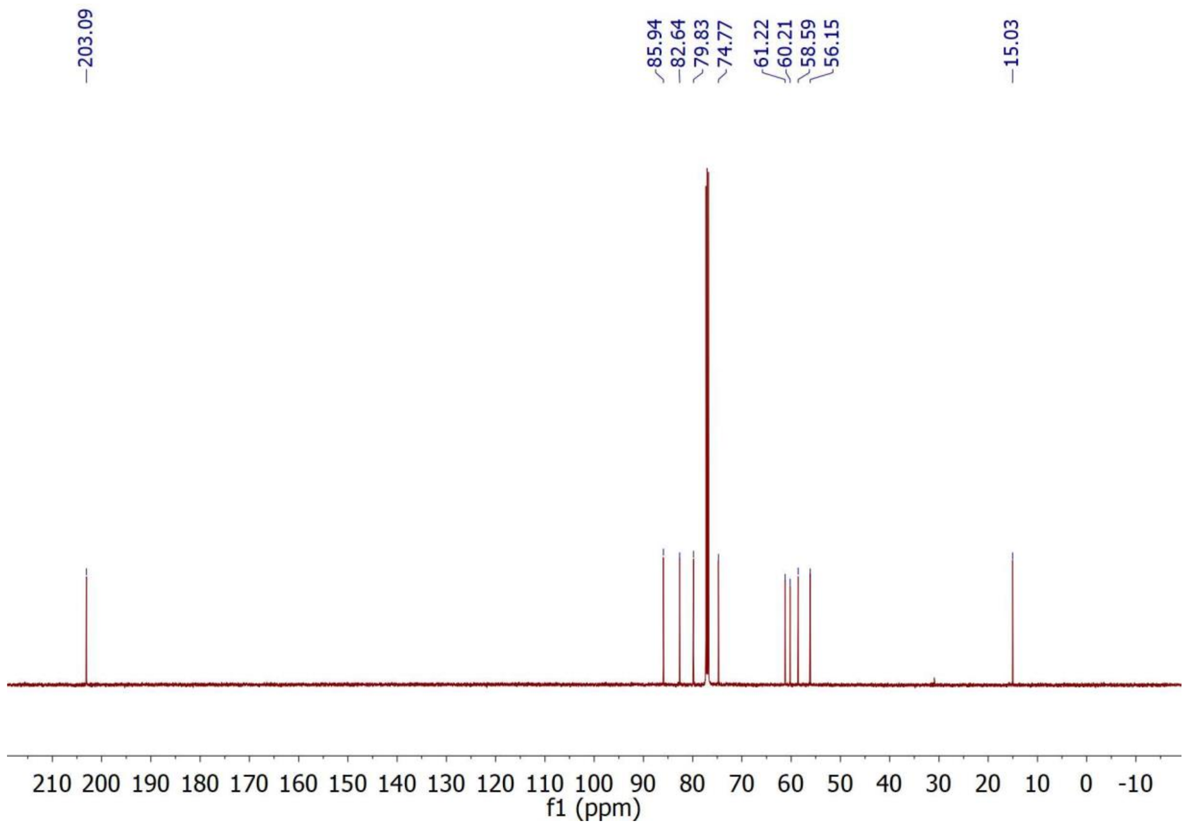

**(*R*)-2,2-dimethyl-1,3-dioxolane-4-carbaldehyde (1ag):**

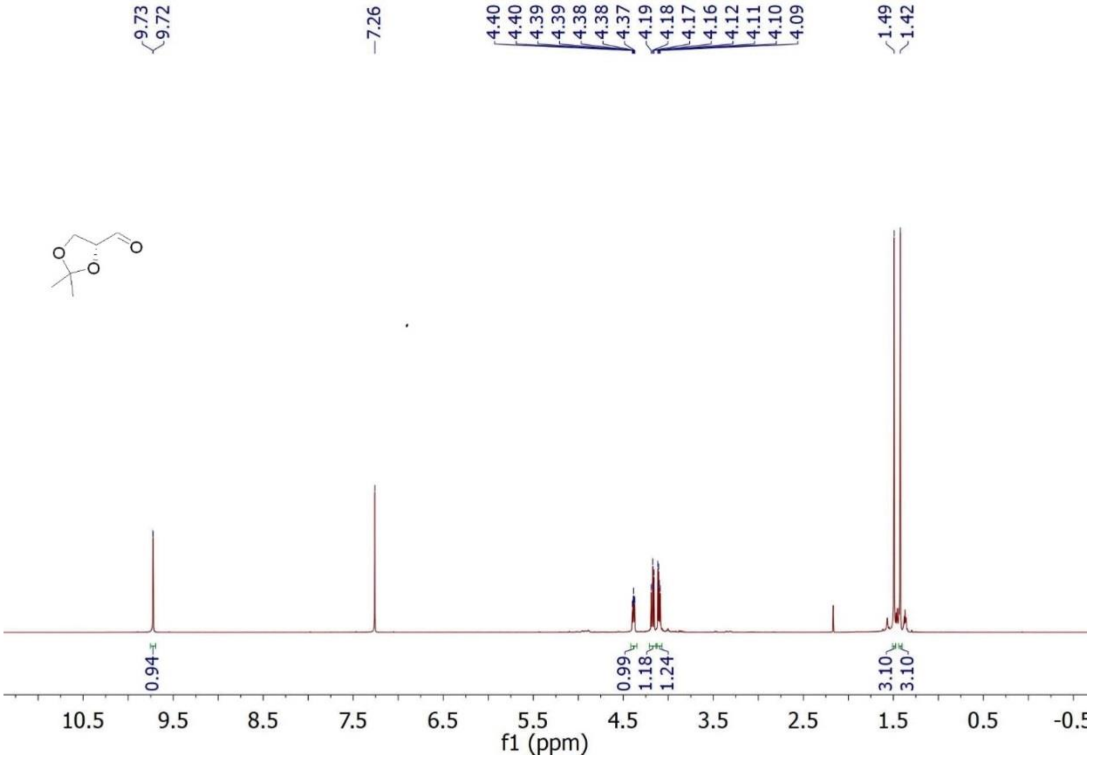

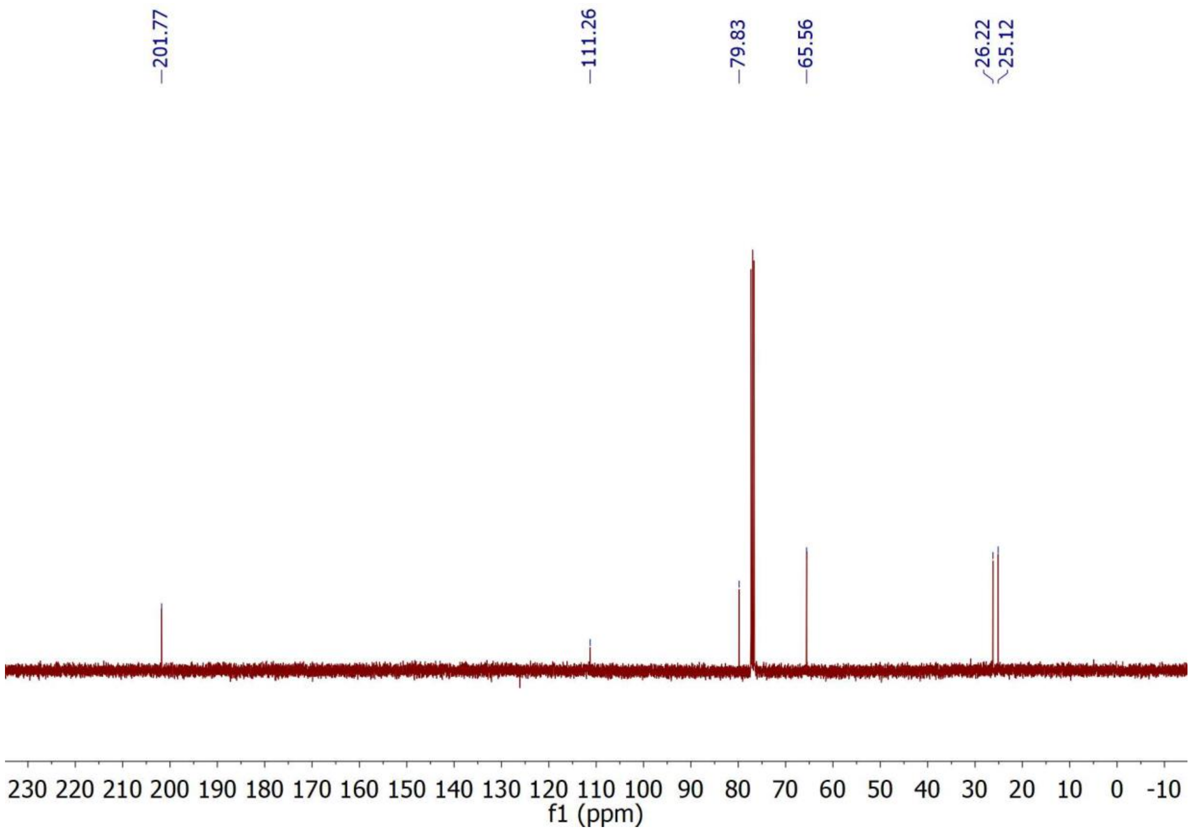


**2,3:4,5-Bis-O-(1-methylethylidene)-D-ribose (1ah):**

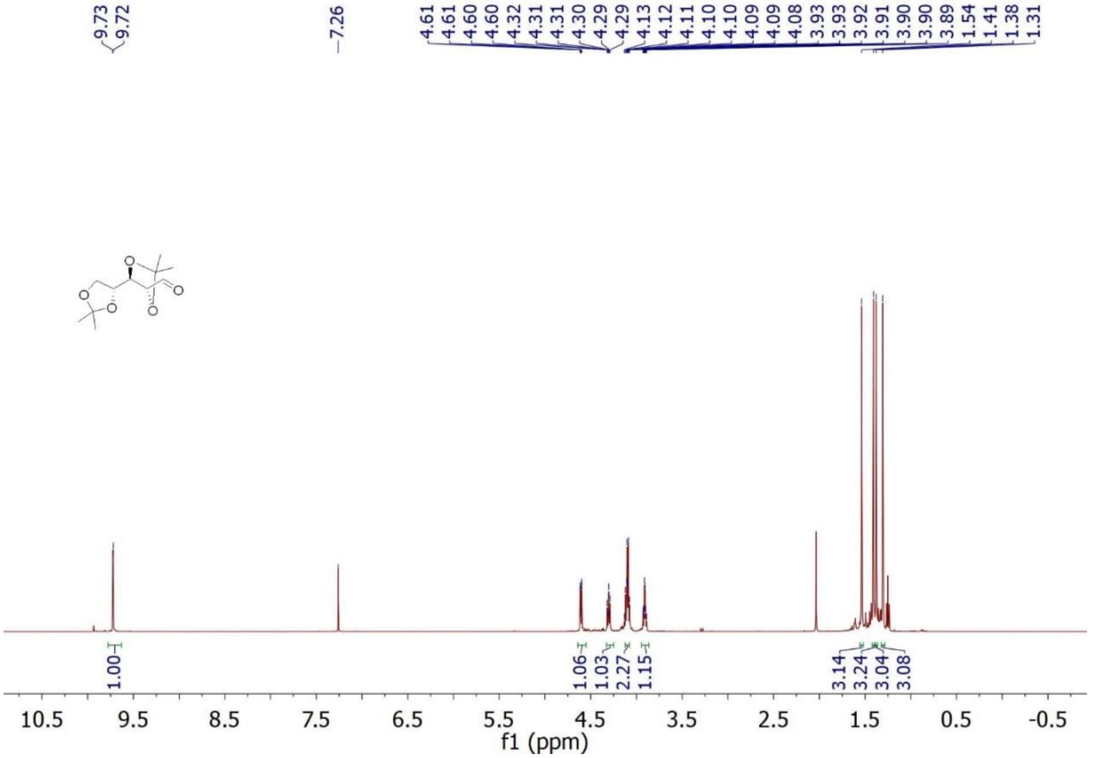

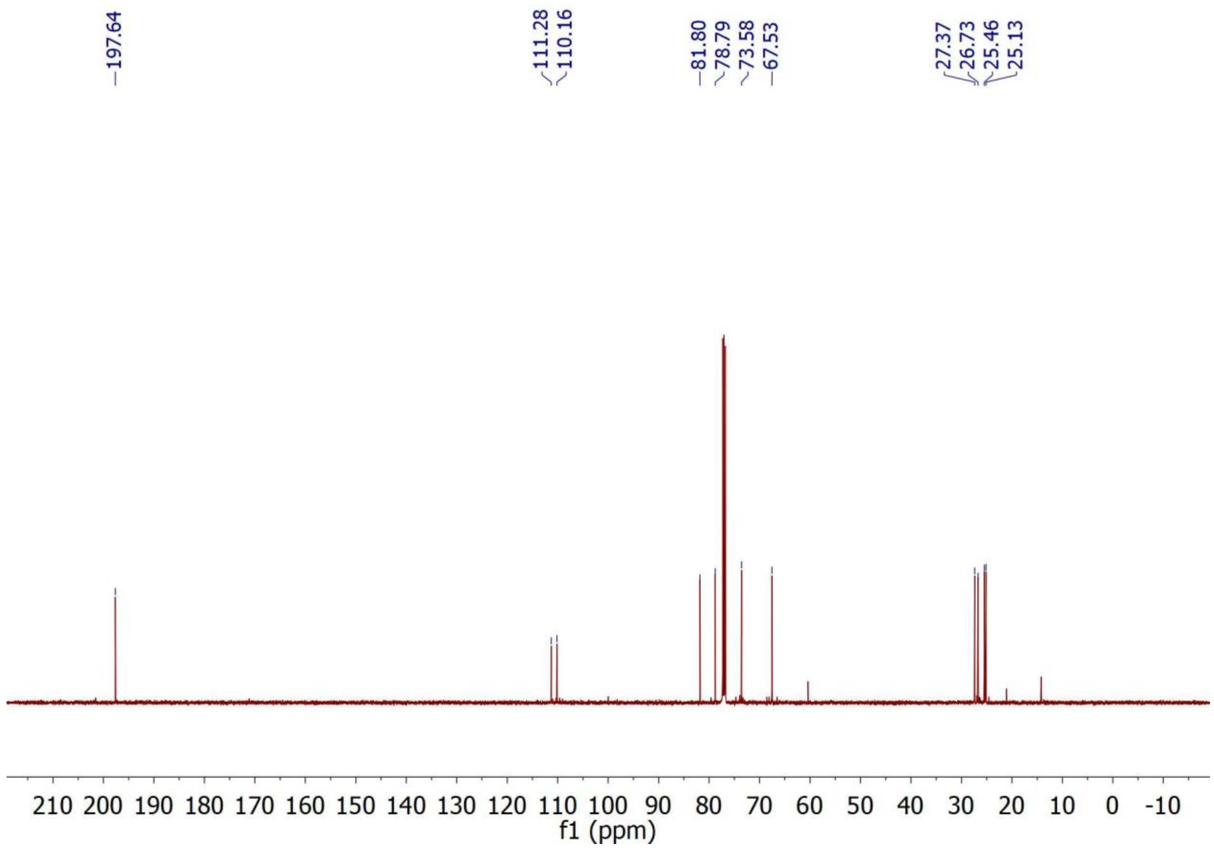


**2,3:4,5-Bis-*O*-(1-methylethylidene)-L-arabinose (1ai):**

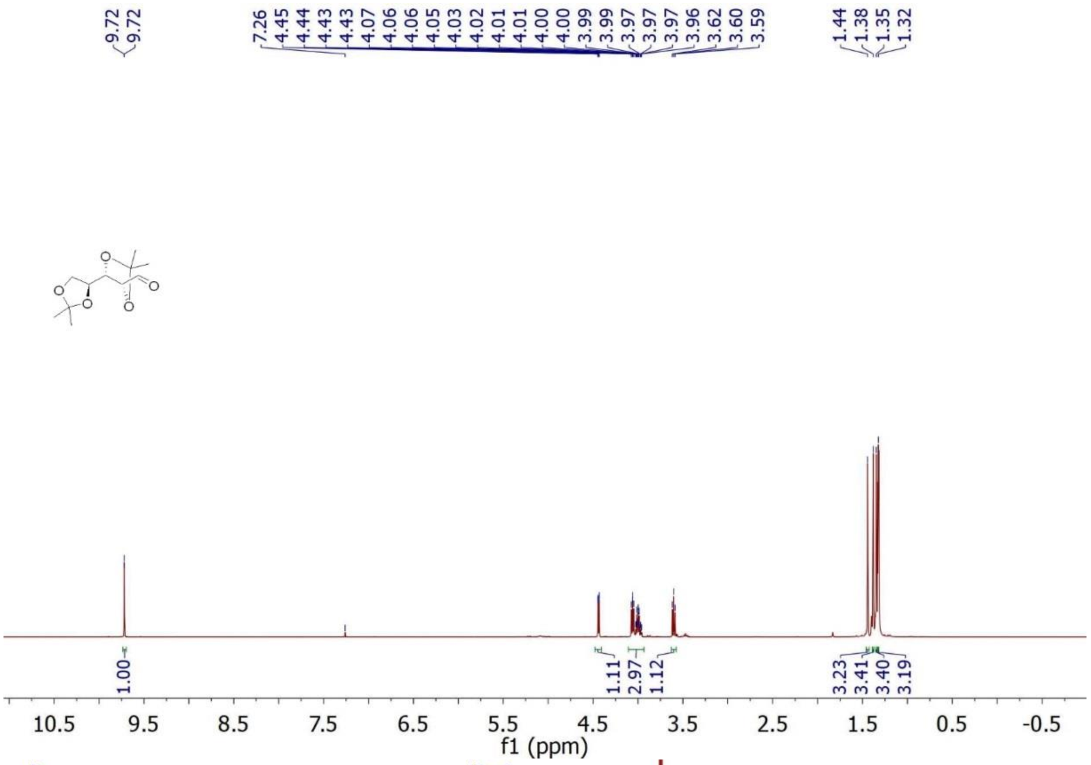


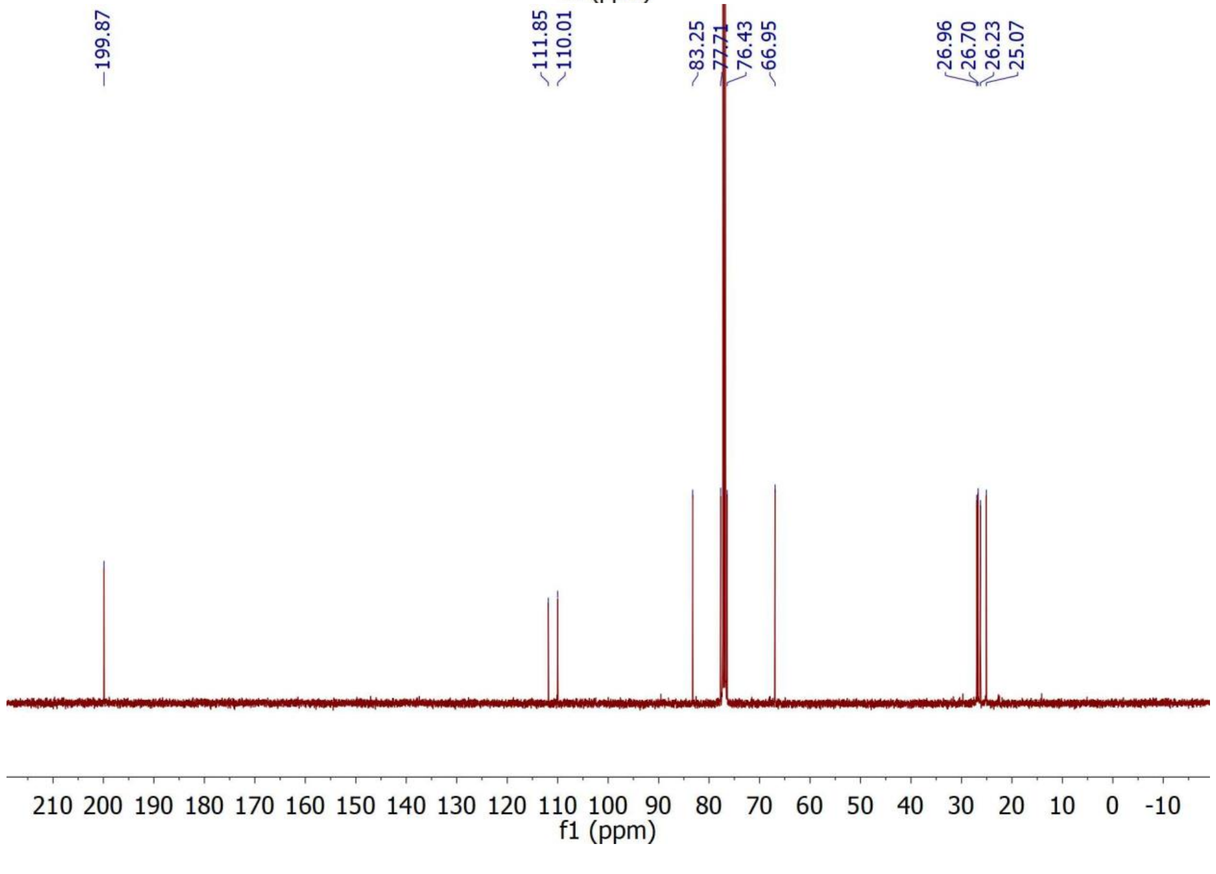


**4-*O*-(methyl)-2,3:5,6-bis-*O*-(1-methylethylidene)-D-galactose (1aj):**

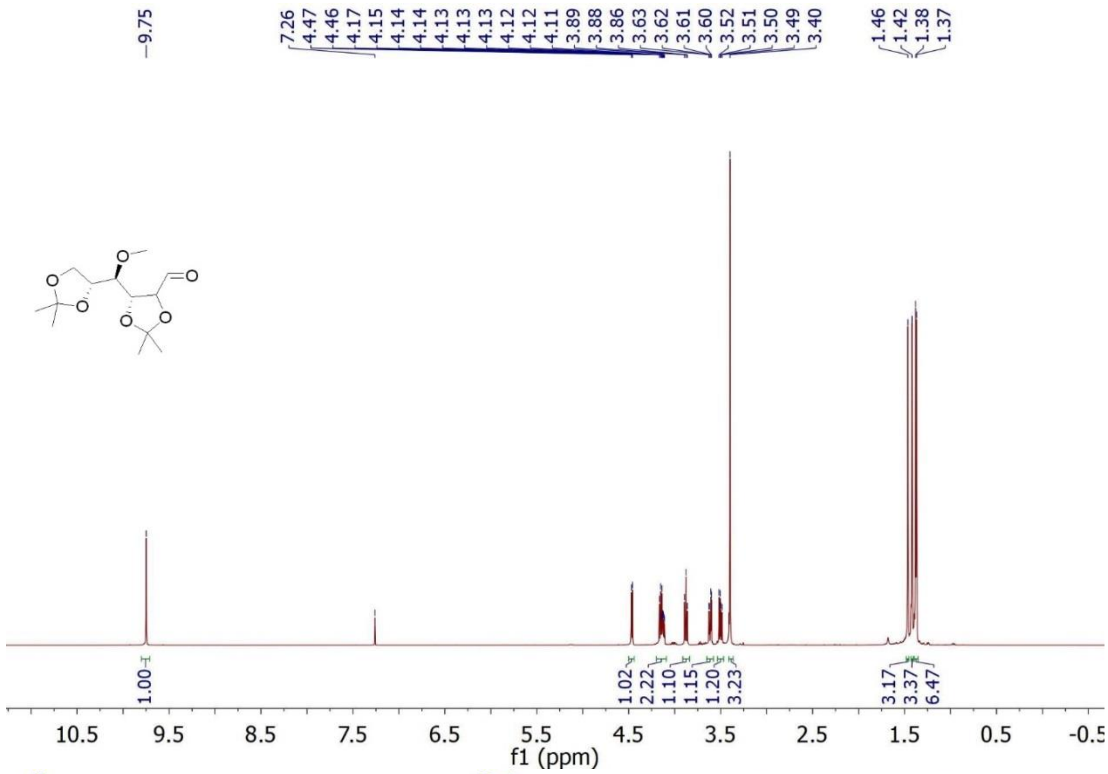


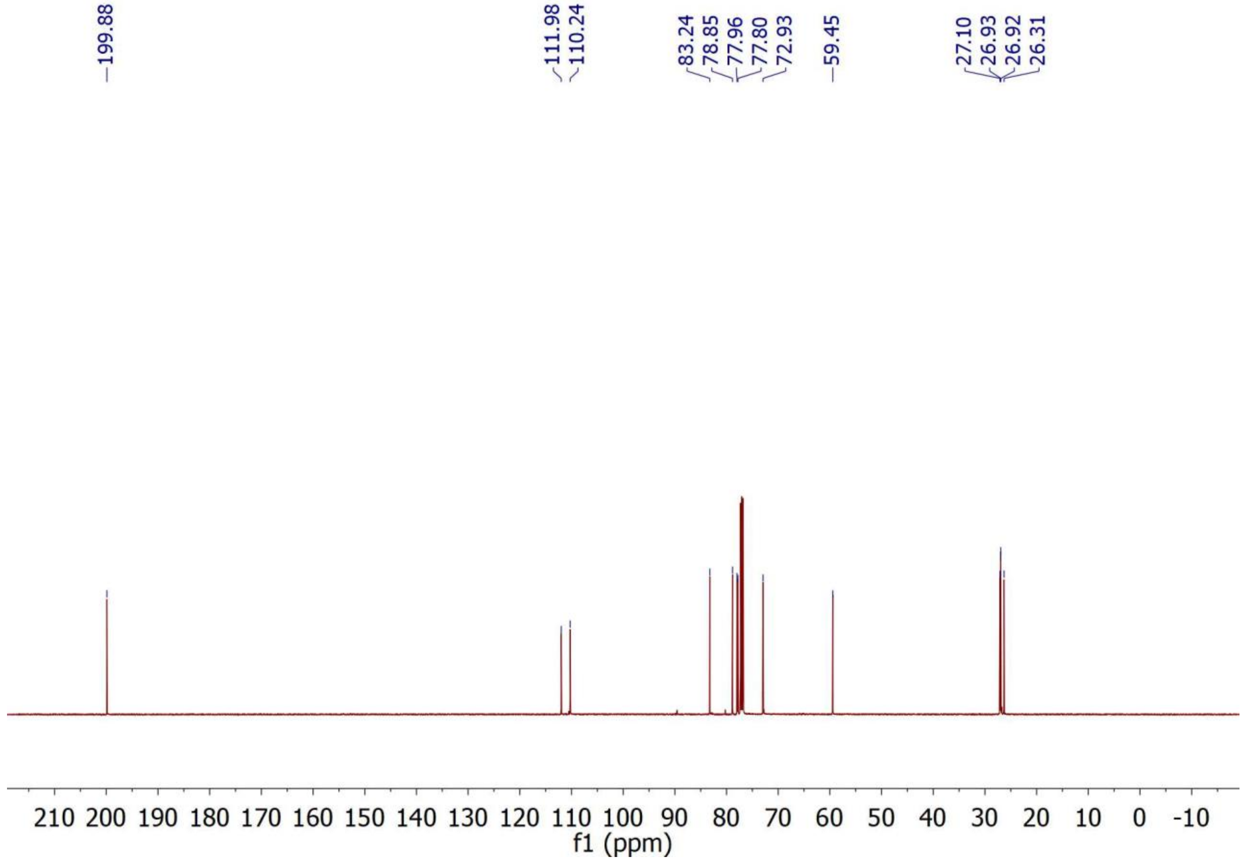


**1,2:3,4-Bis-*O*-(1-methylethylidene)-$\alpha$-D-galacto-hexodialdo-1,5-pyranose (1ak):**

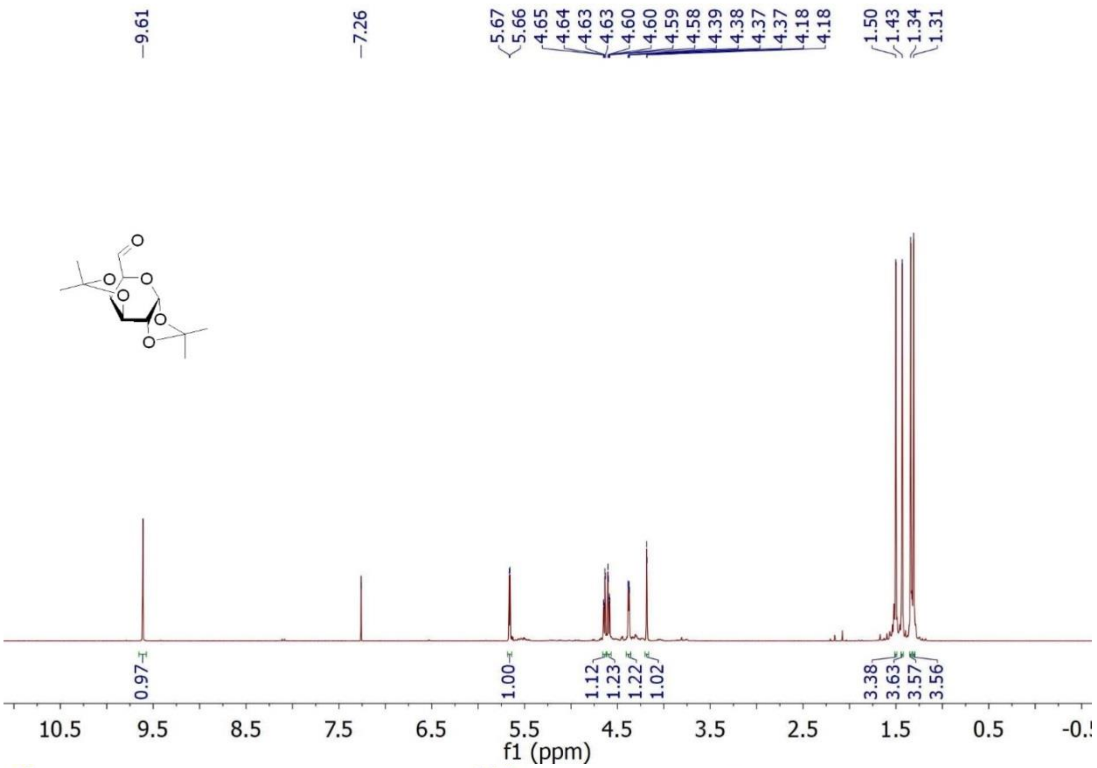
9.61
7.26
5.67
5.66
4.65
4.64
4.63
4.63
4.60
4.60
4.59
4.58
4.39
4.38
4.37
4.37
4.18
4.18
1.50
1.43
1.34
1.31
0.97
1.00
1.12
1.23
1.22
1.02
3.38
3.63
3.57
3.56
10.5 9.5 8.5 7.5 6.5 5.5 4.5 3.5 2.5 1.5 0.5
f1 (ppm)

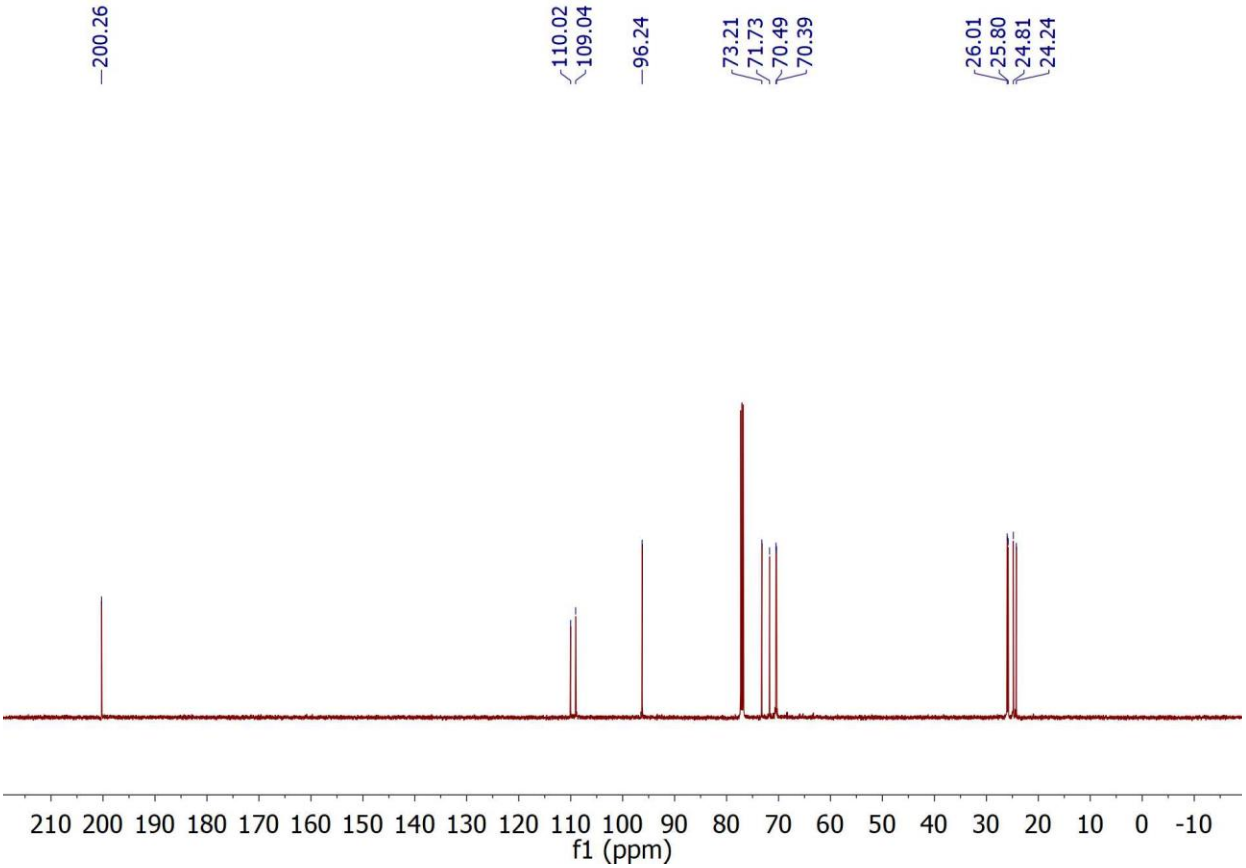
200.26
110.02
109.04
96.24
73.21
71.73
70.49
70.39
26.01
25.80
24.81
24.24
210 200 190 180 170 160 150 140 130 120 110 100 90 80 70 60 50 40 30 20 10 0 -10
f1 (ppm)

**2,3:4,5-Bis-O-(1-methylethylidene)-β-D-arabino-hexos-2-ulo-2,6-pyranose (1al):**

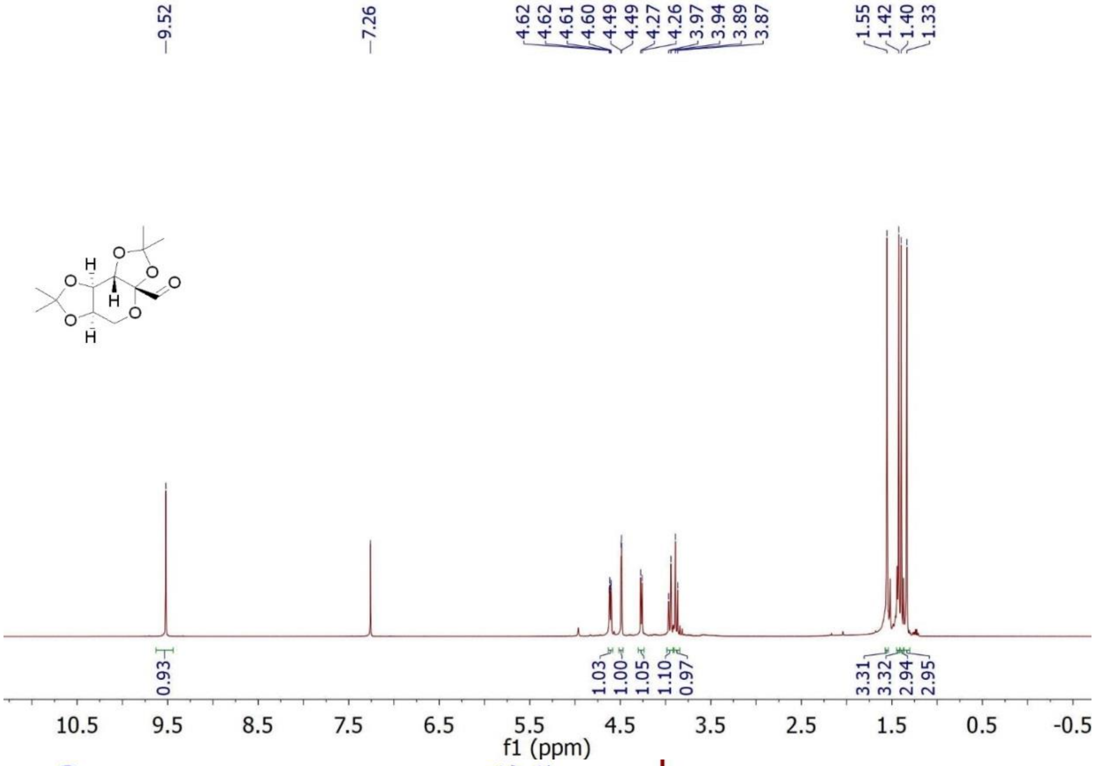


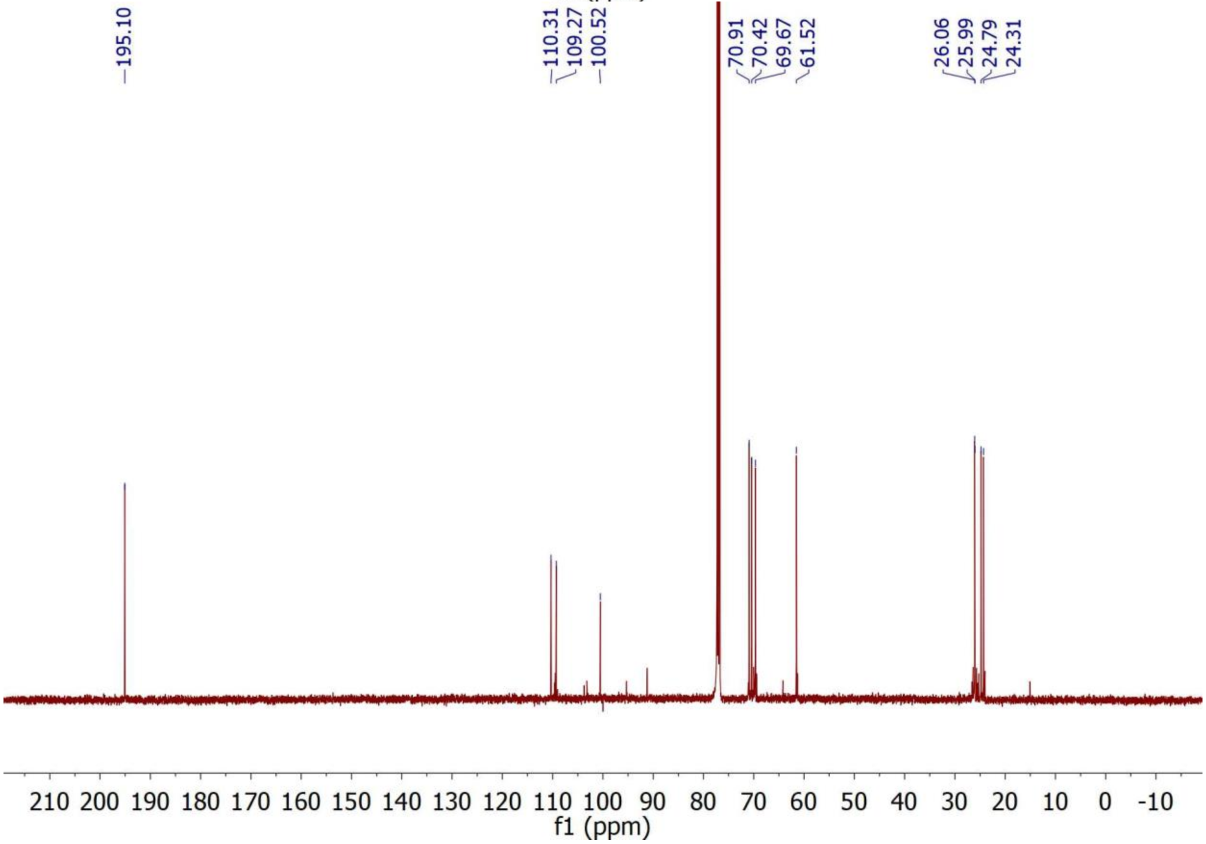


**Methyl 2,3-O-(1-methylethylidene)-β-D-ribo-pentodialdo-1,4-furanoside (1am):**

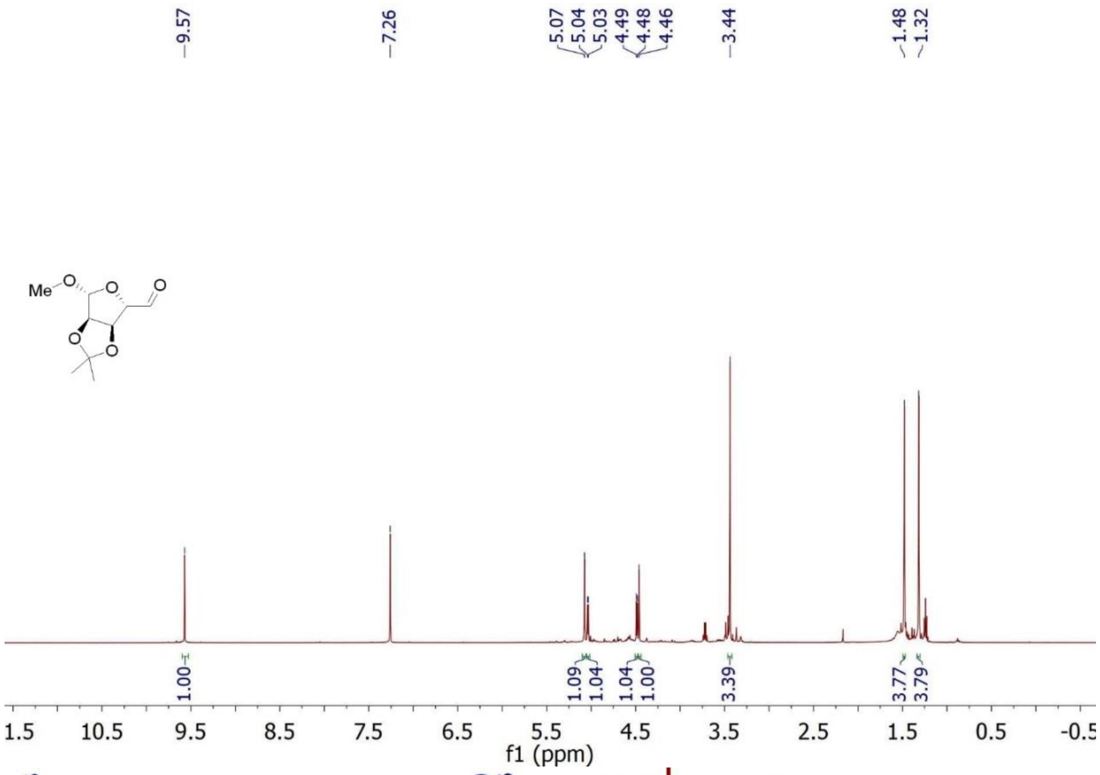


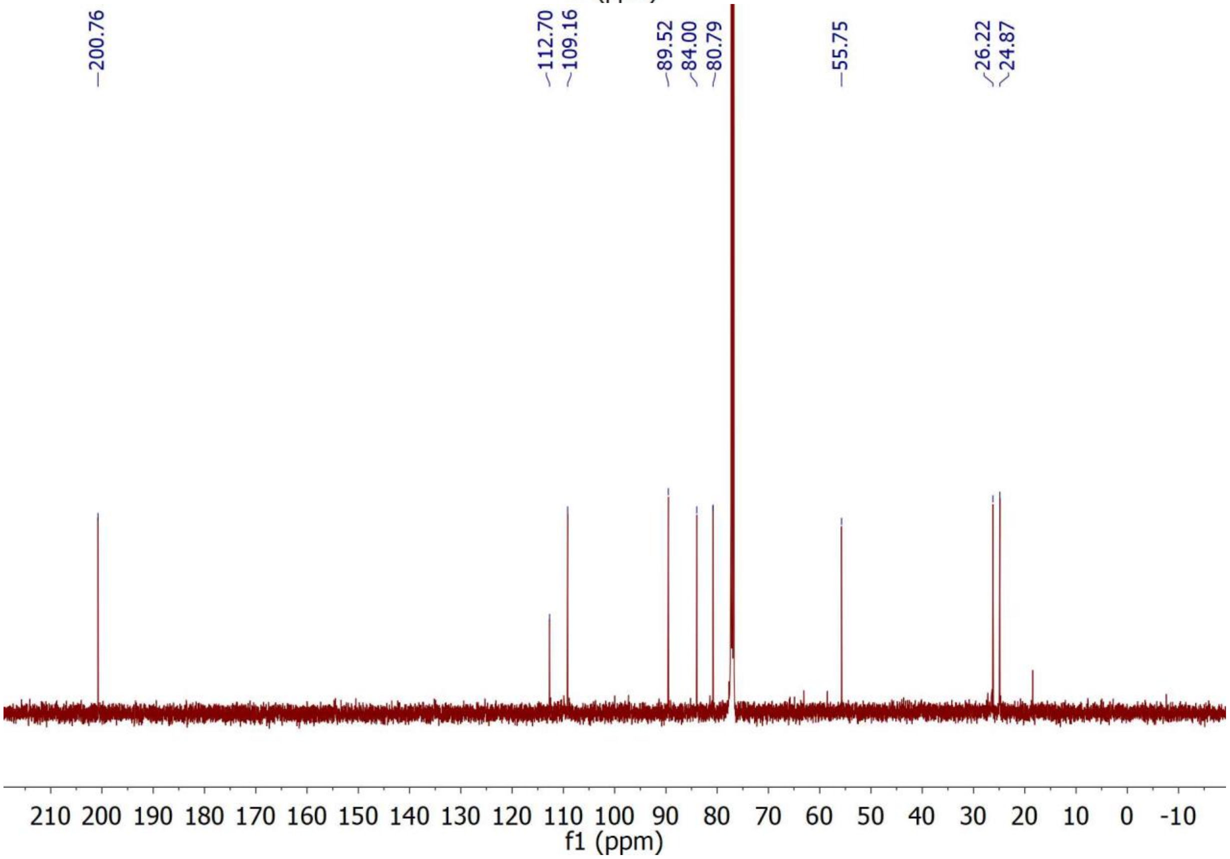


**(2*R*,3*S*,4*R*,5*S*,*E*)-1-hydrazineylidenehexane-2,3,4,5-tetraol (1an):**

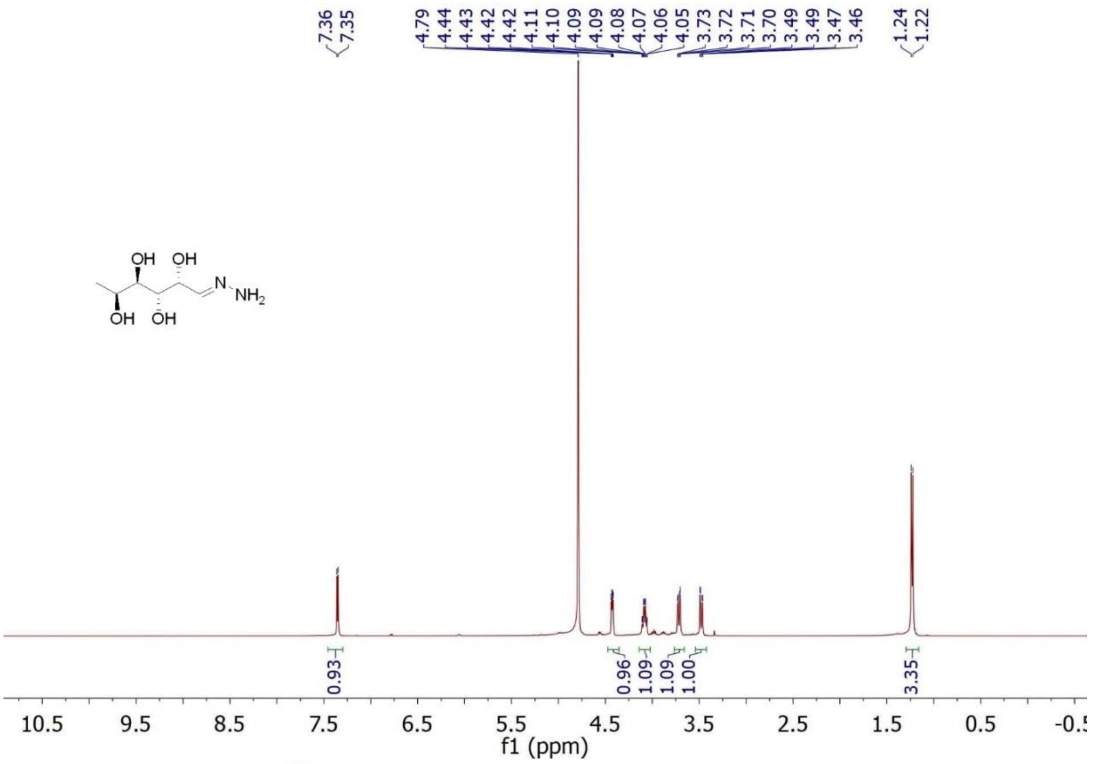


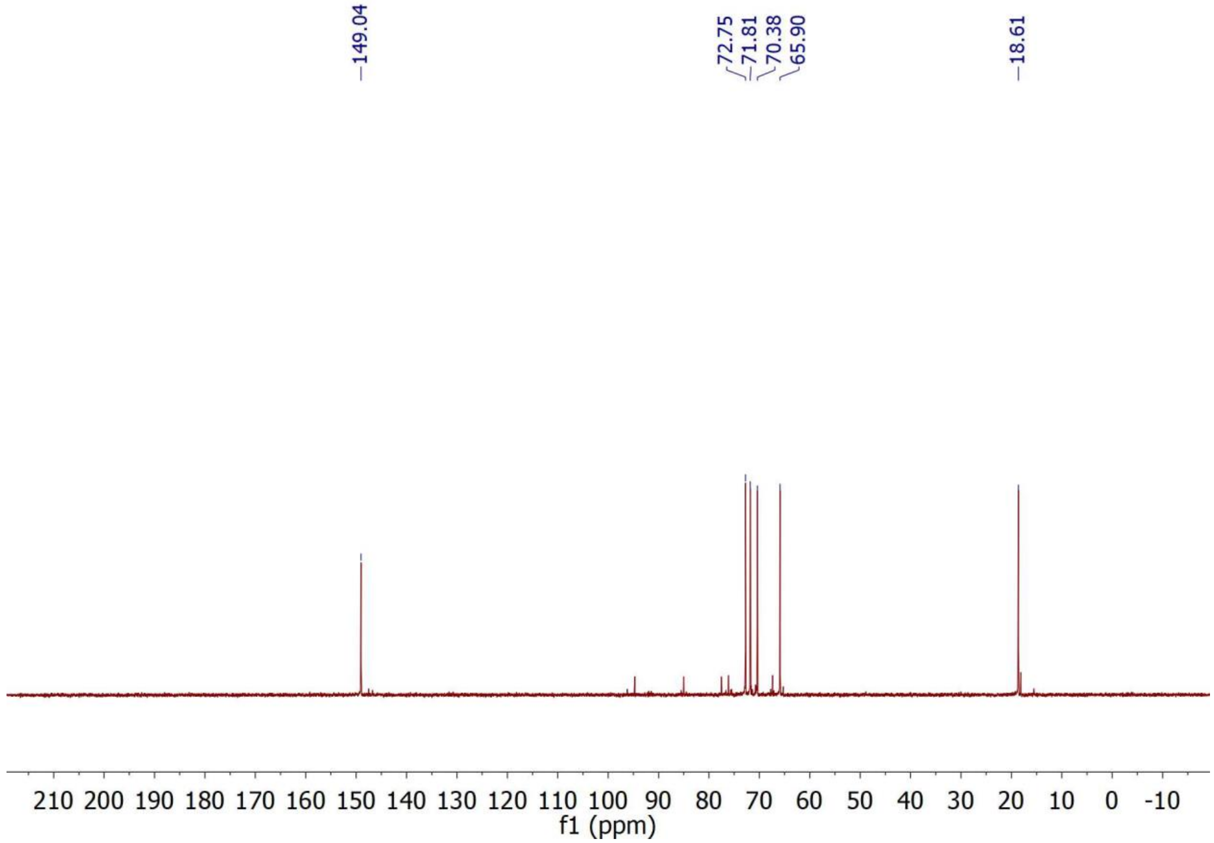


**(2*R*,3*S*,4*R*,*E*)-5-hydrazineylidenepentane-1,2,3,4-tetraol (1ao):**

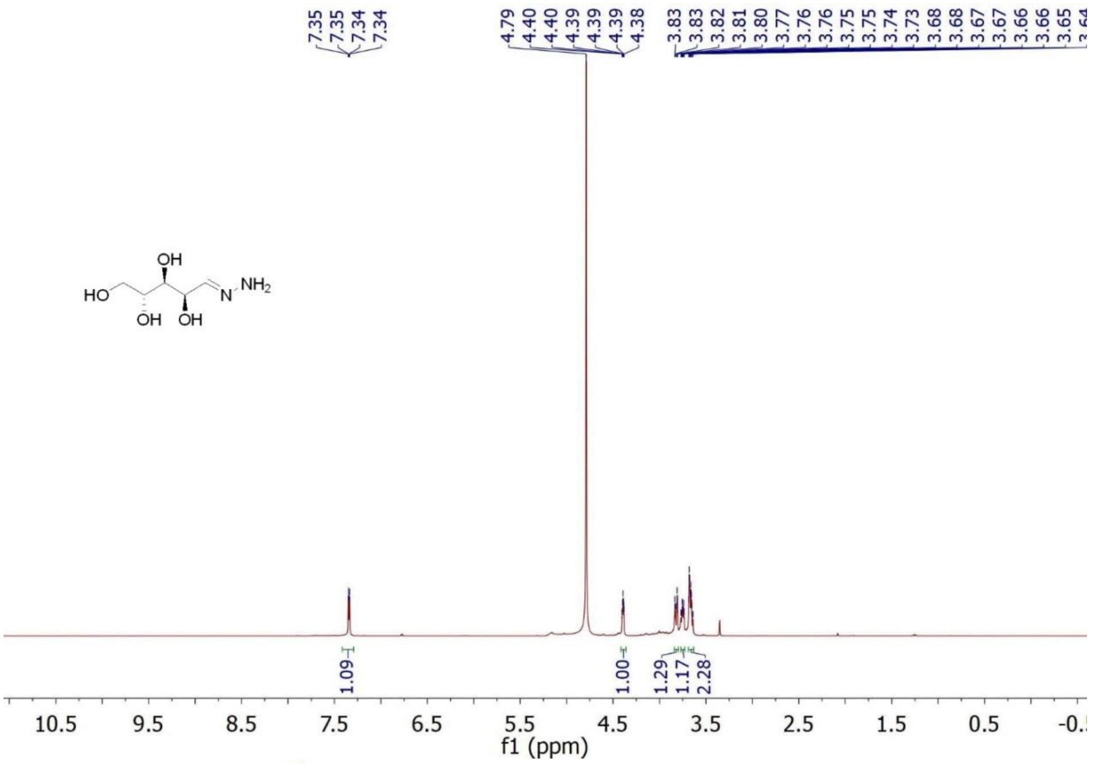


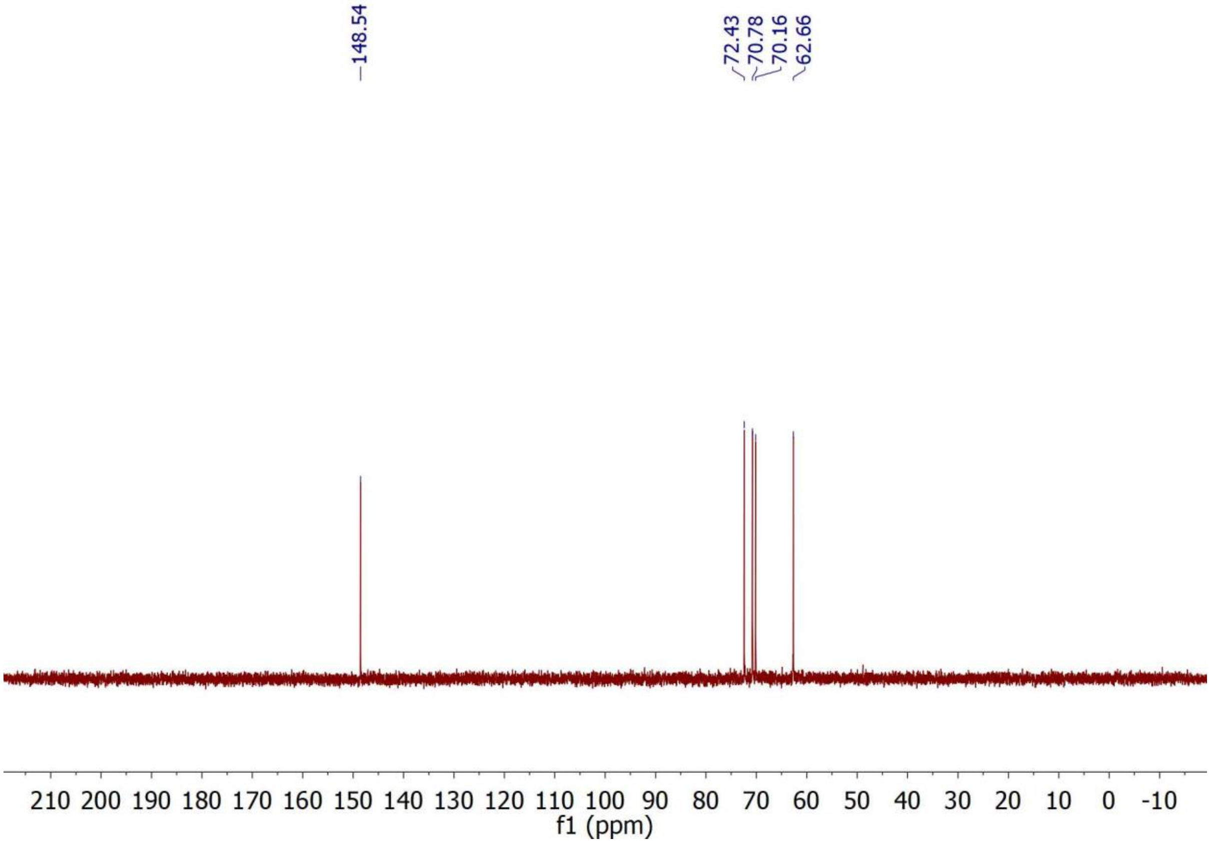


**(2*S*,3*R*,4*S*,*E*)-5-hydrazineylidenepentane-1,2,3,4-tetraol (1ap):**

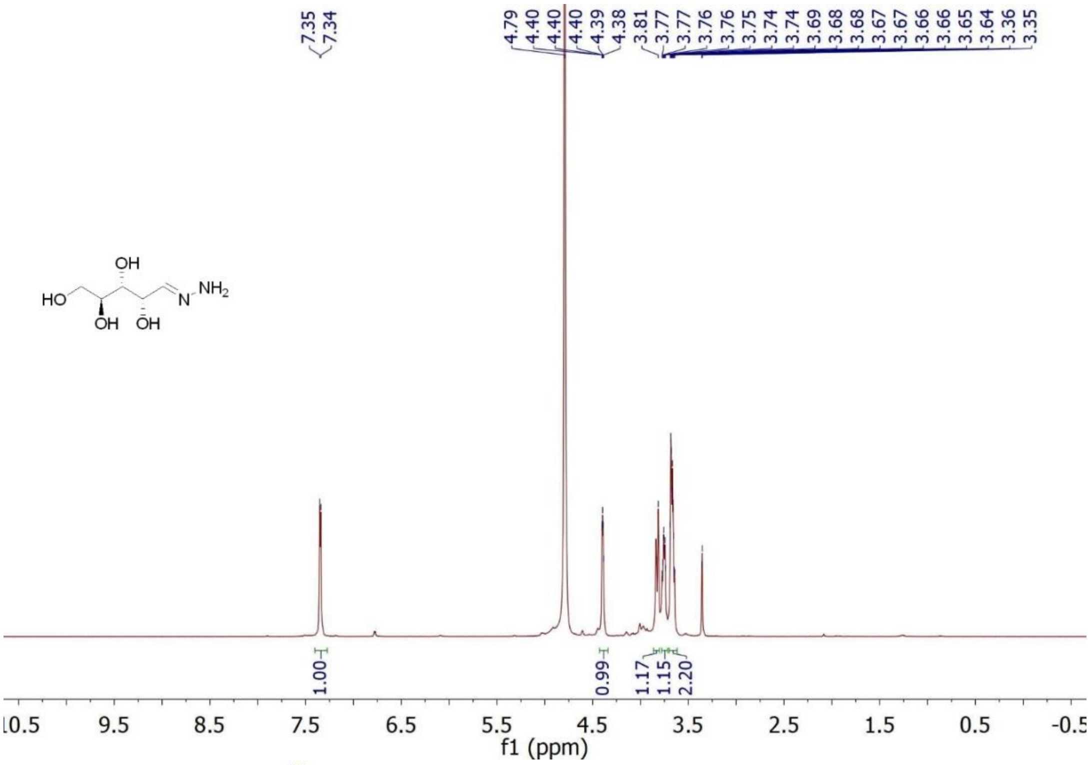


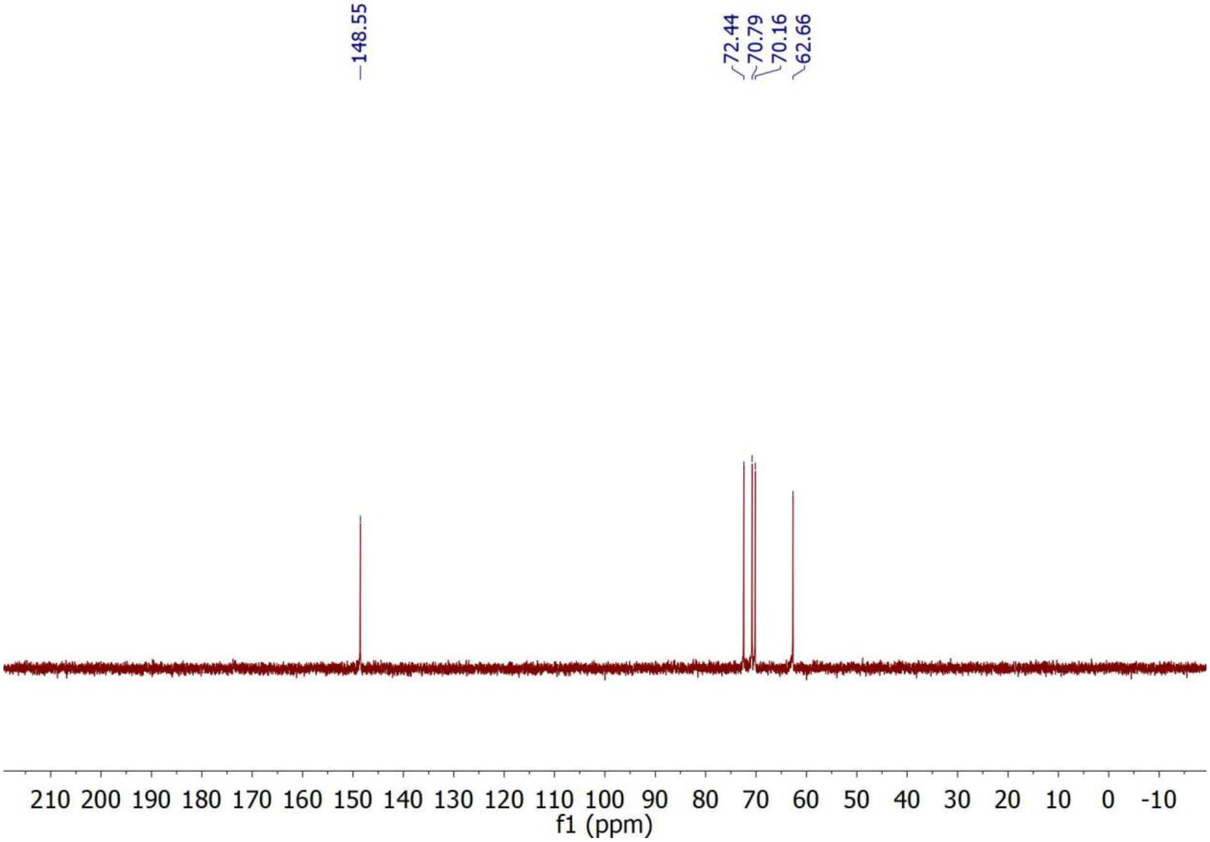


**(2*S*,3*S*,4*S*,5*S*,*E*)-1-hydrazineylidenehexane-2,3,4,5-tetraol (1aq):**

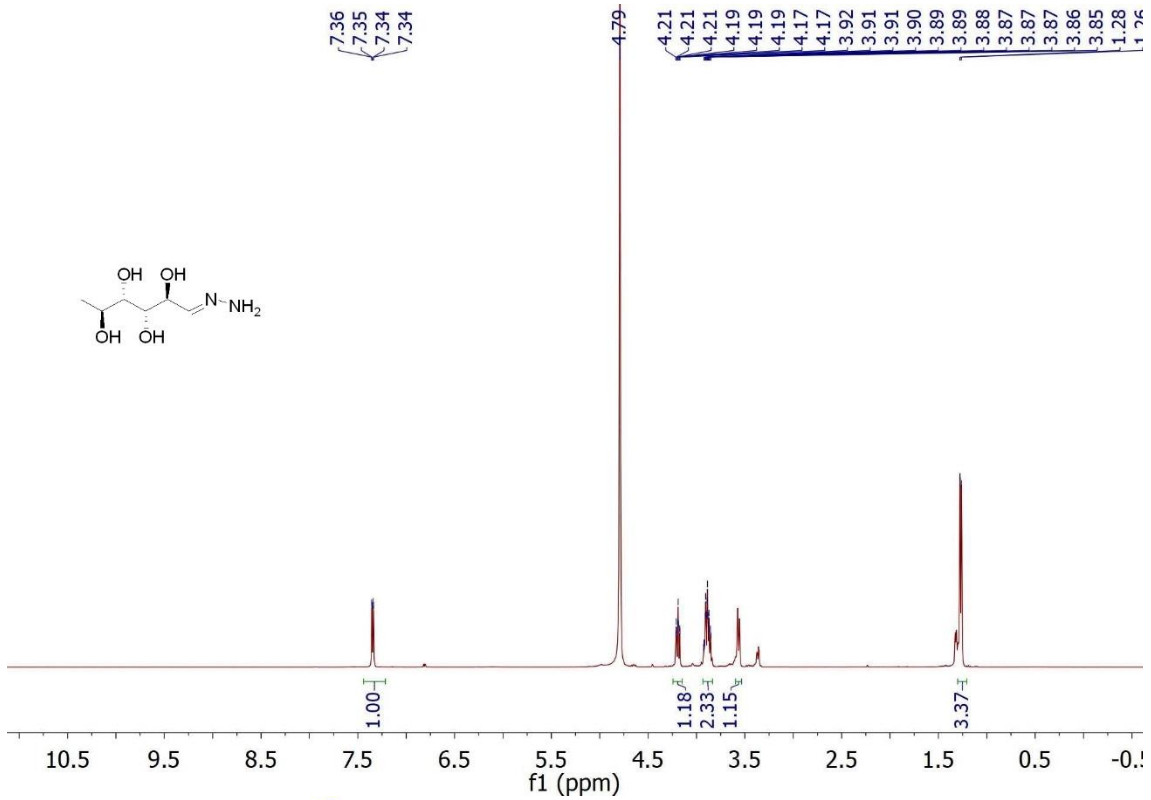


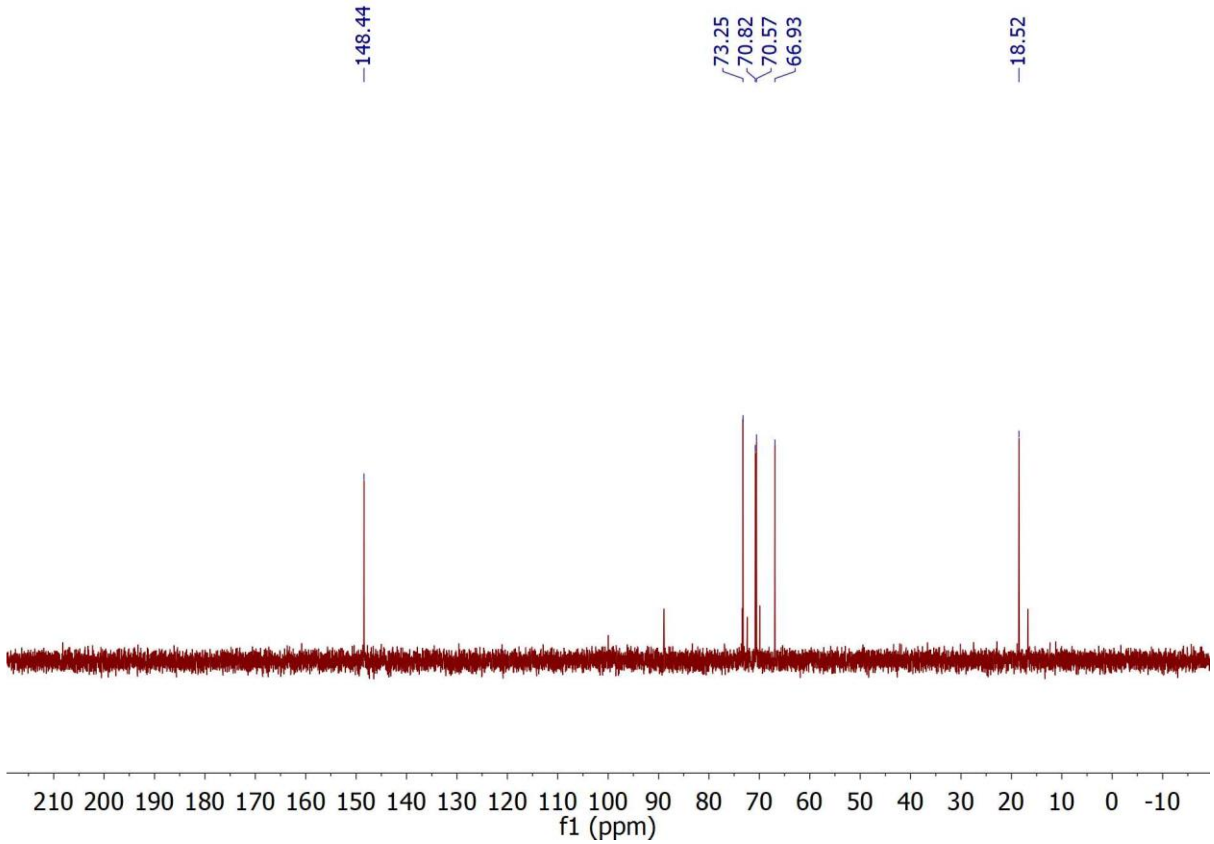


**2-methylallyl acetate (2ba):**

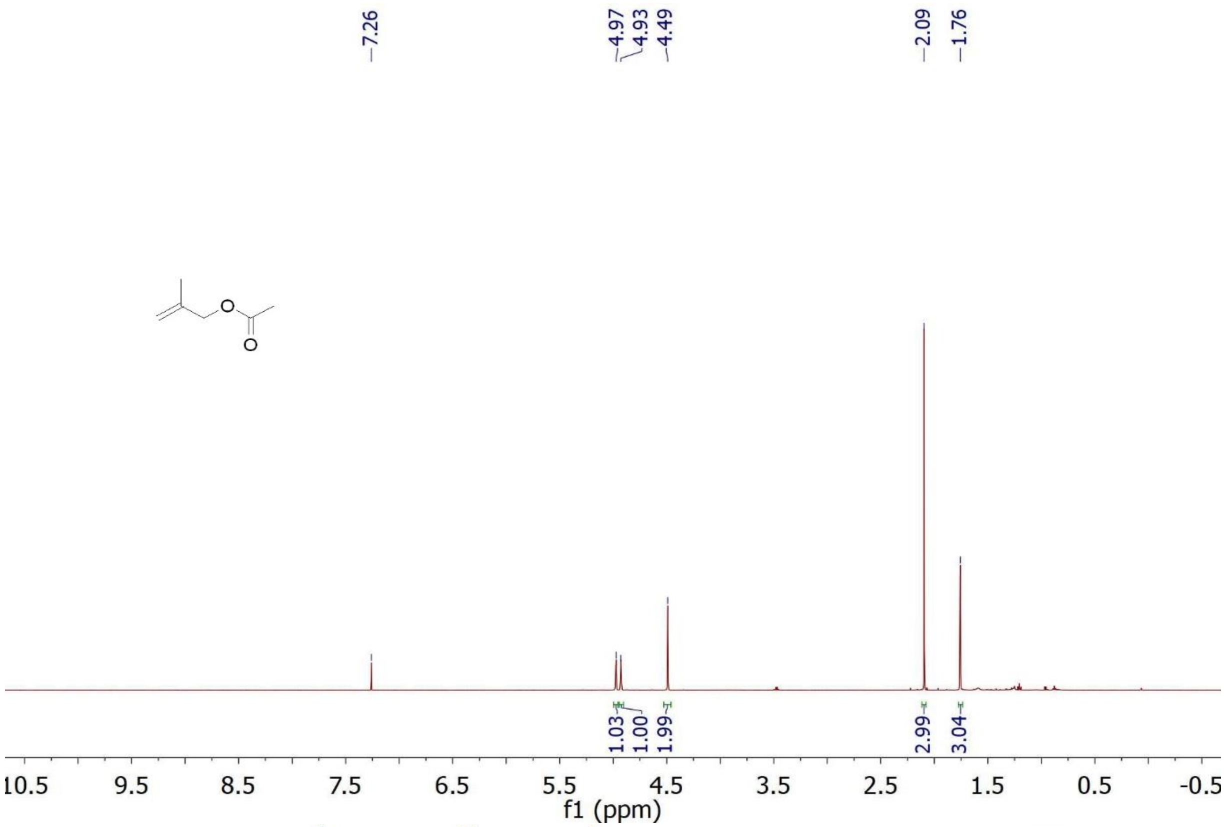


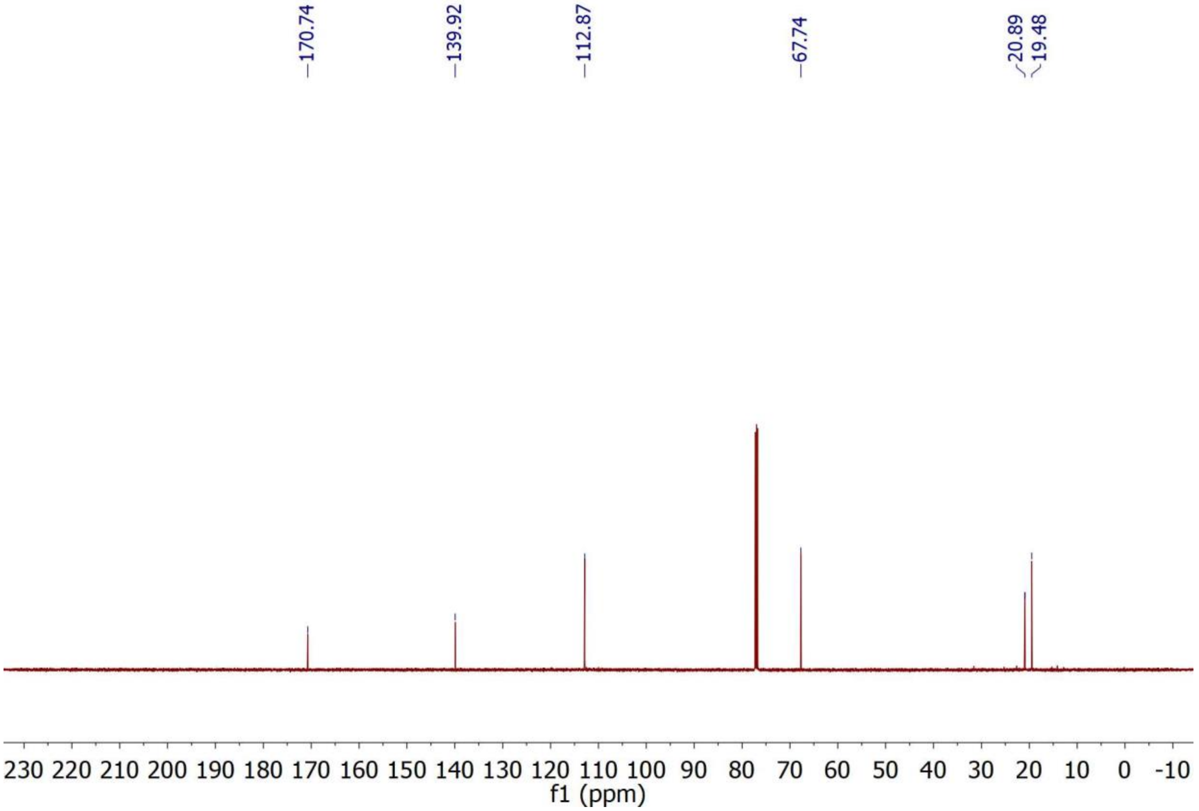


**2-chloroallyl acetate (2bb):**

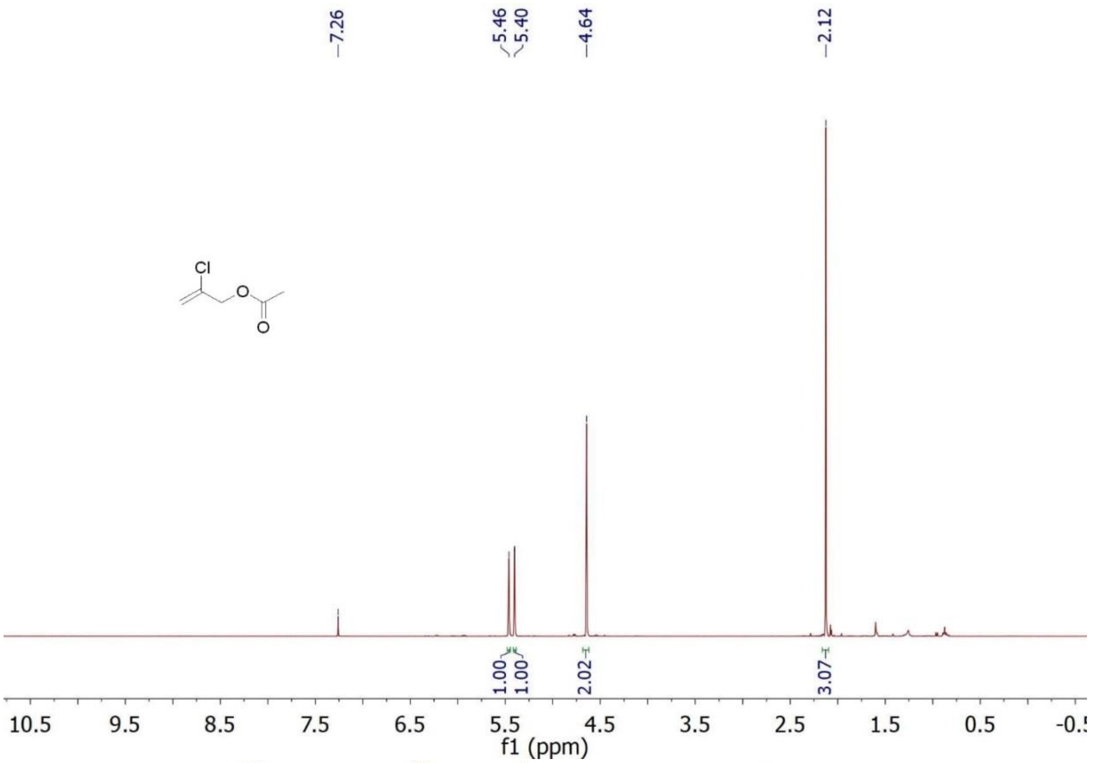


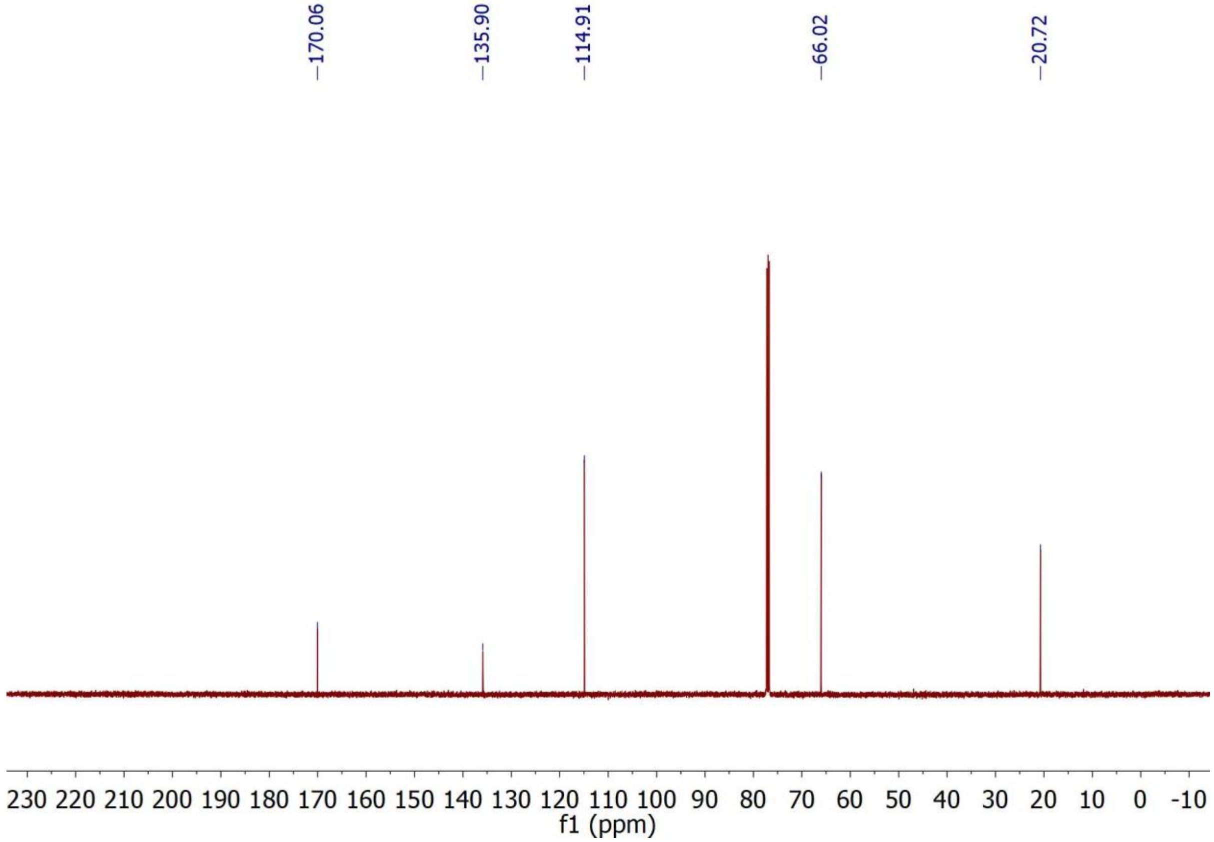


**2-phenylallyl acetate (2bc):**

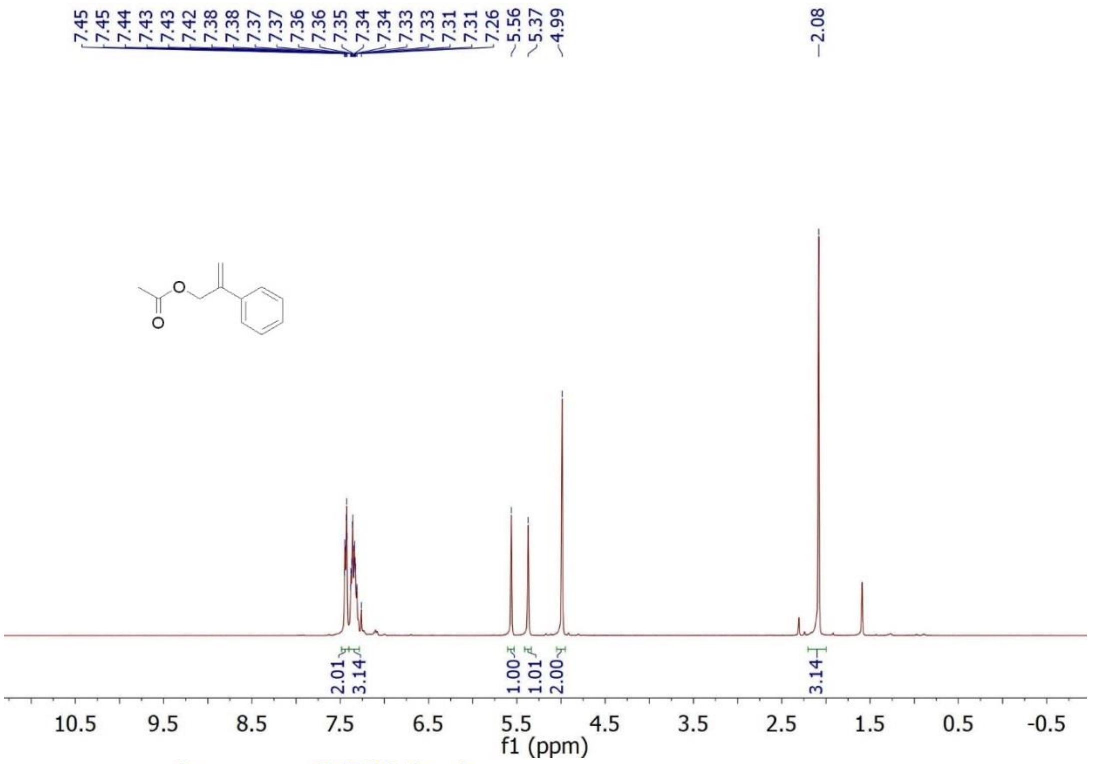


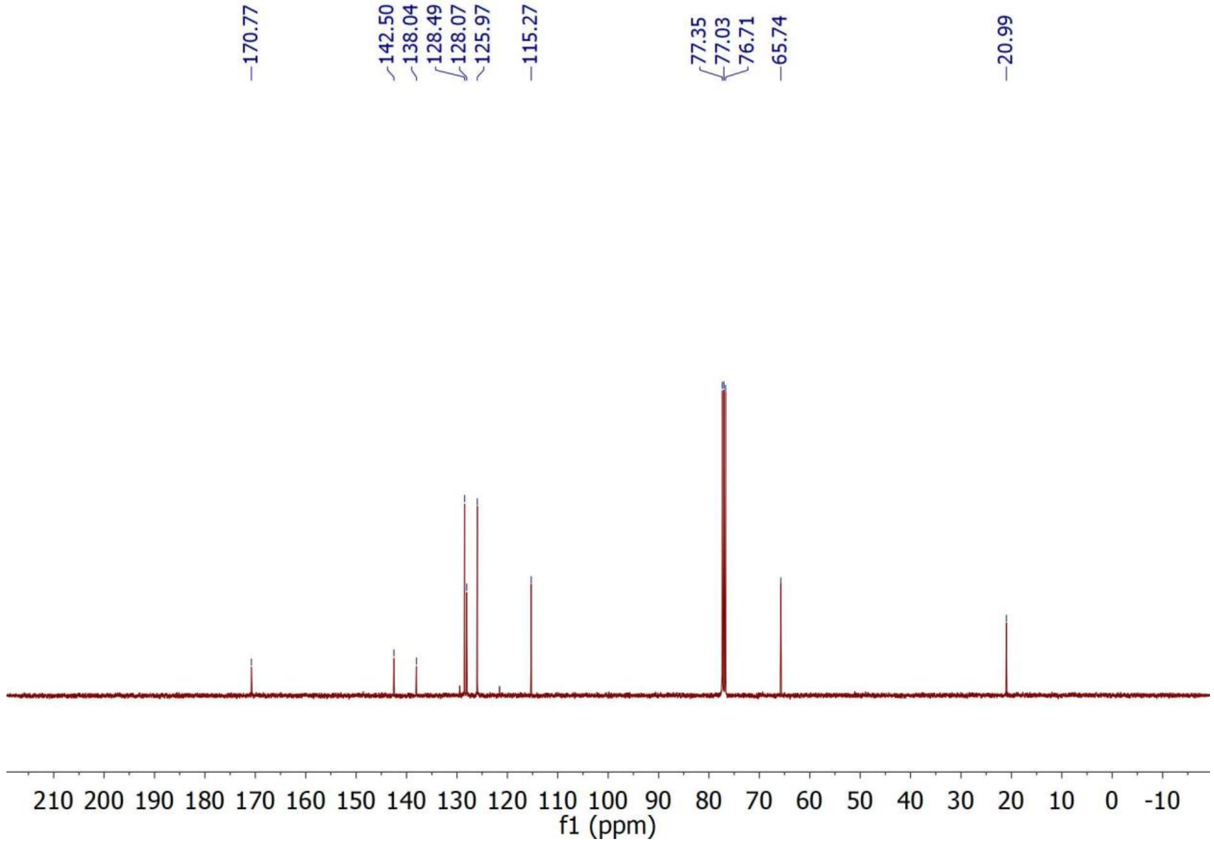


**2-(4-chlorophenyl)allyl acetate (2bd):**

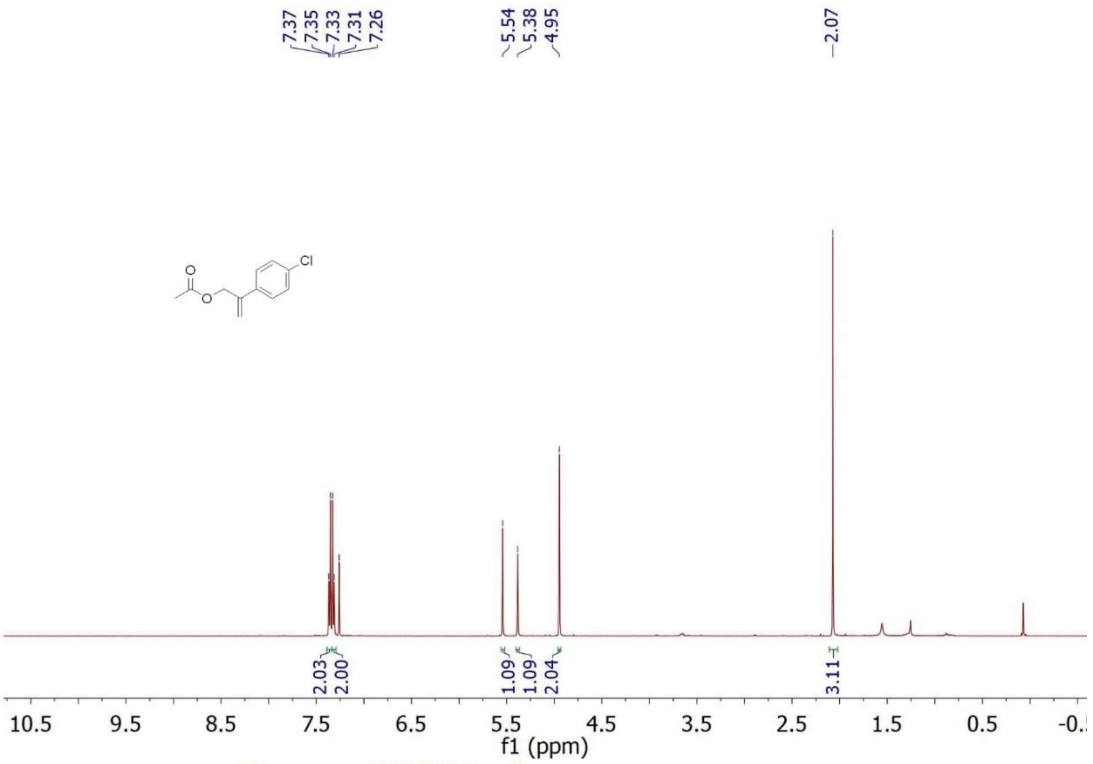


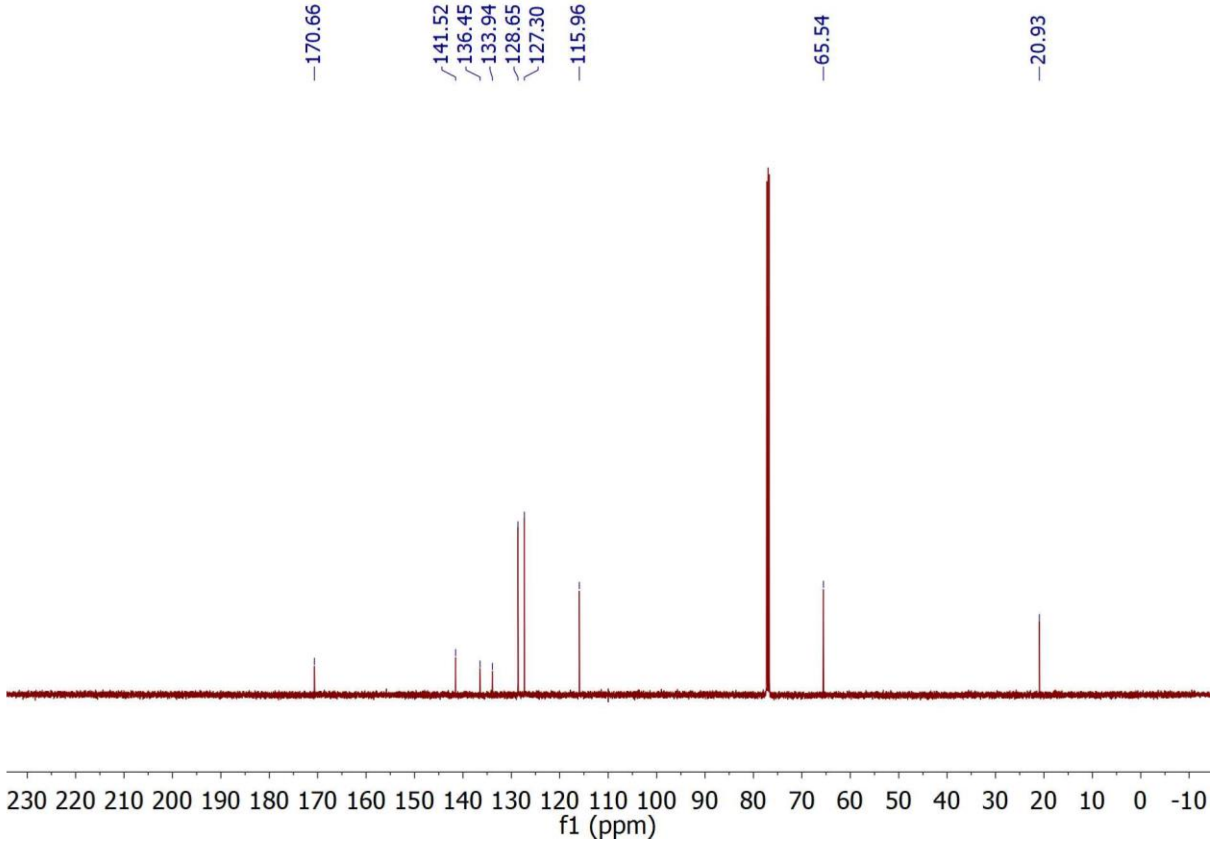


**2-(4-methoxyphenyl)allyl acetate (2be):**

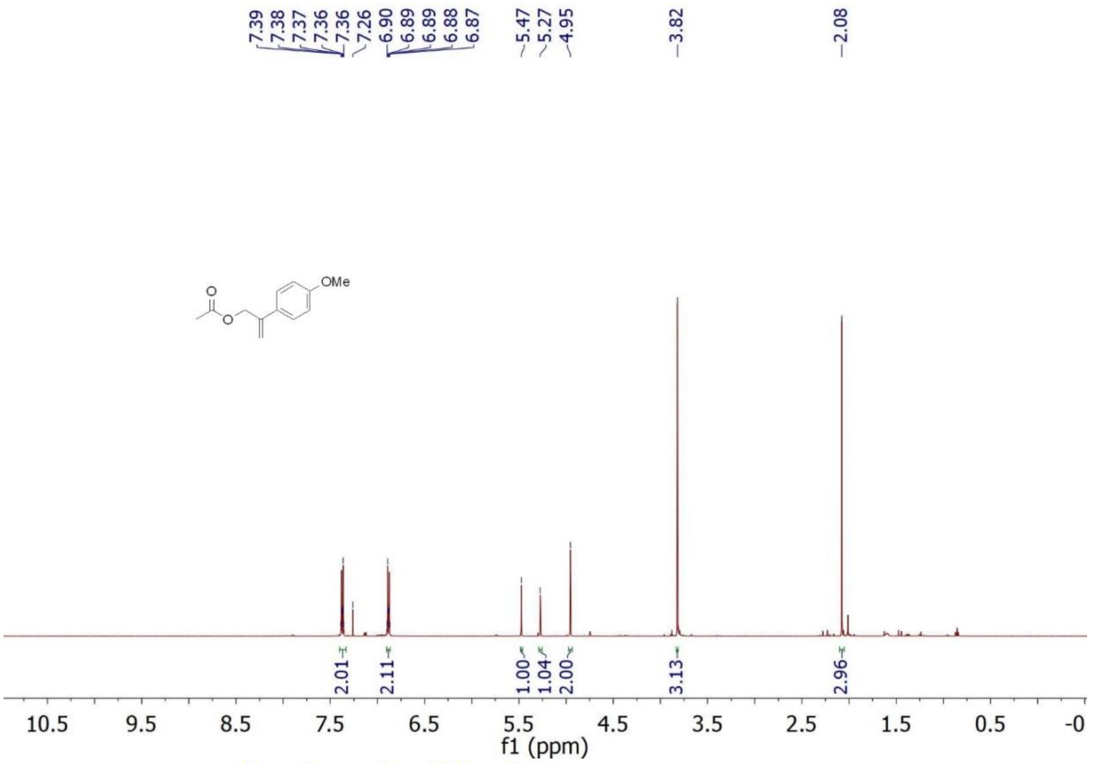


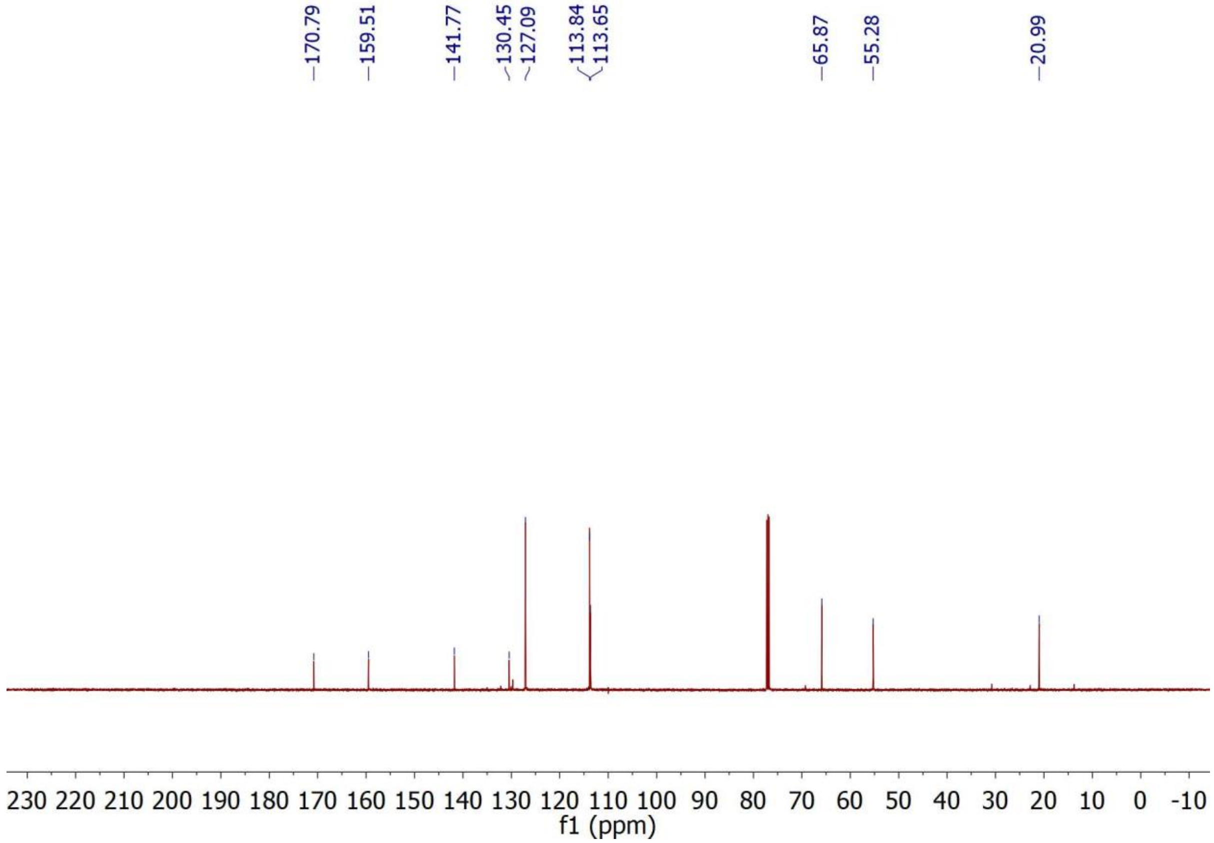


**2-(2,4-dimethylphenyl)allyl acetate (2bf):**

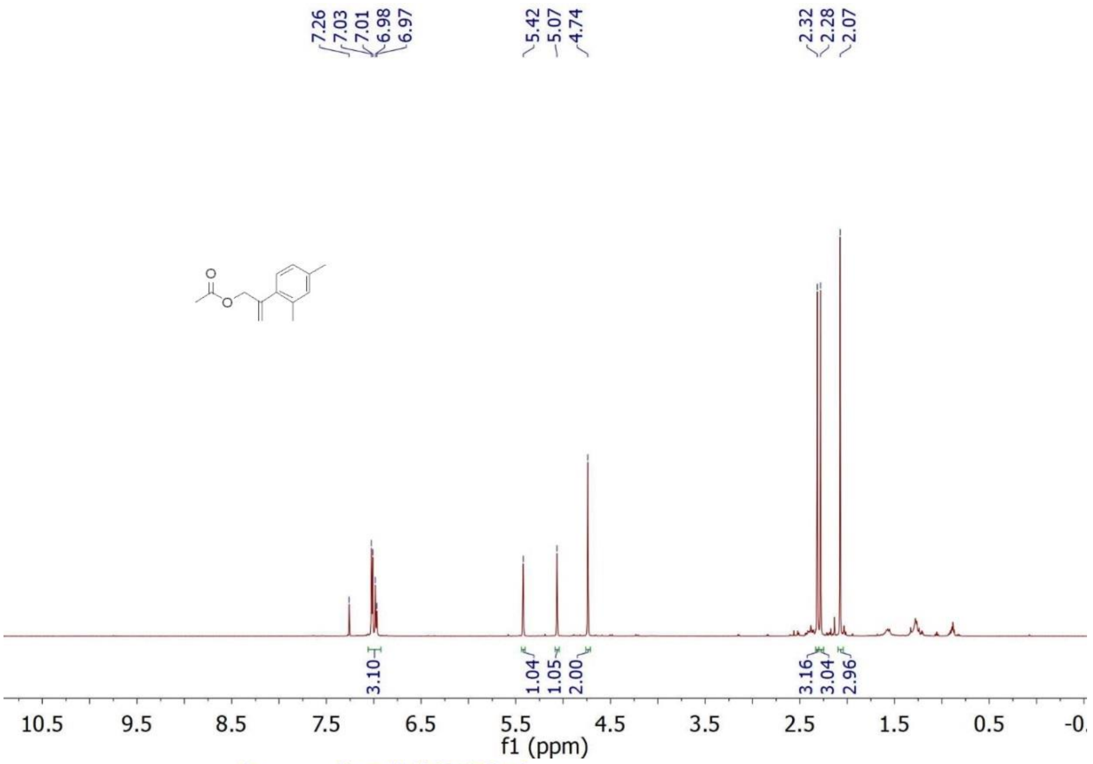


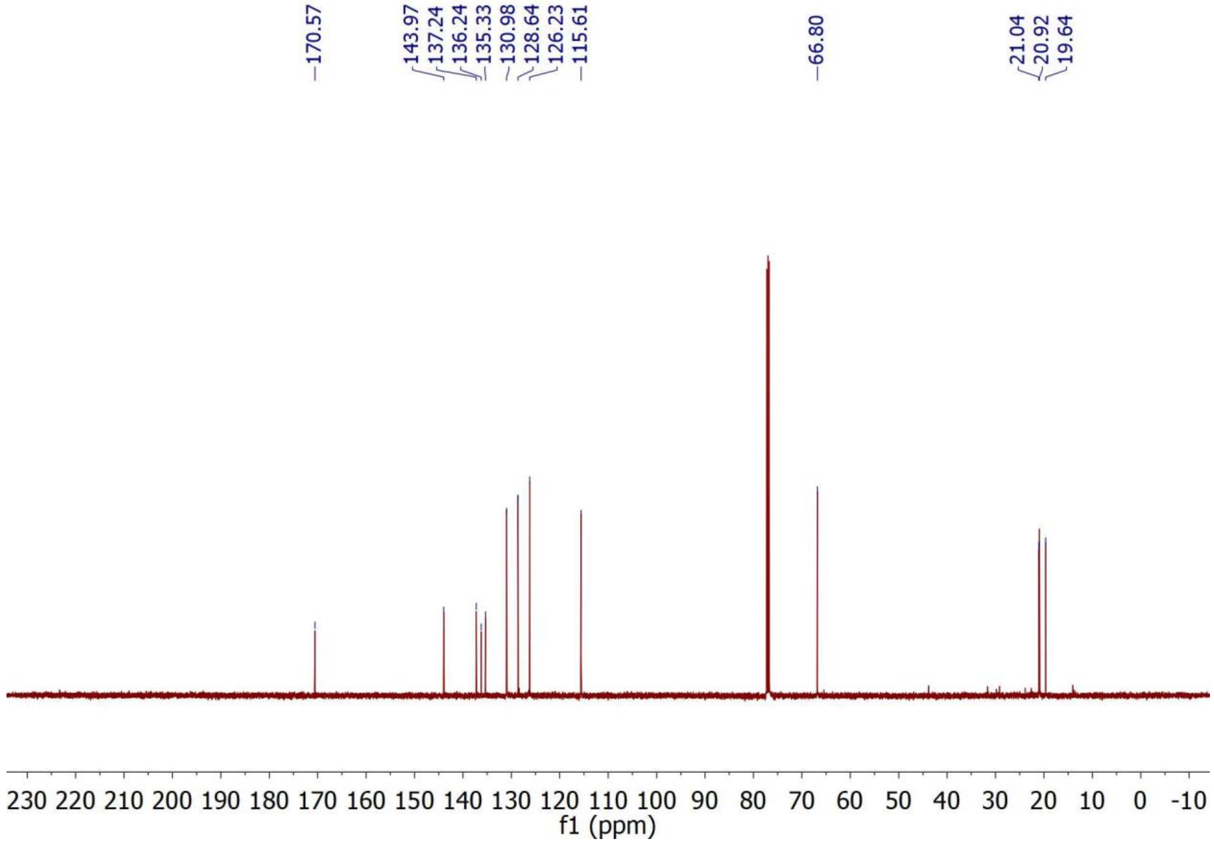


**2-(4-cyanophenyl)allyl acetate (2bg)**

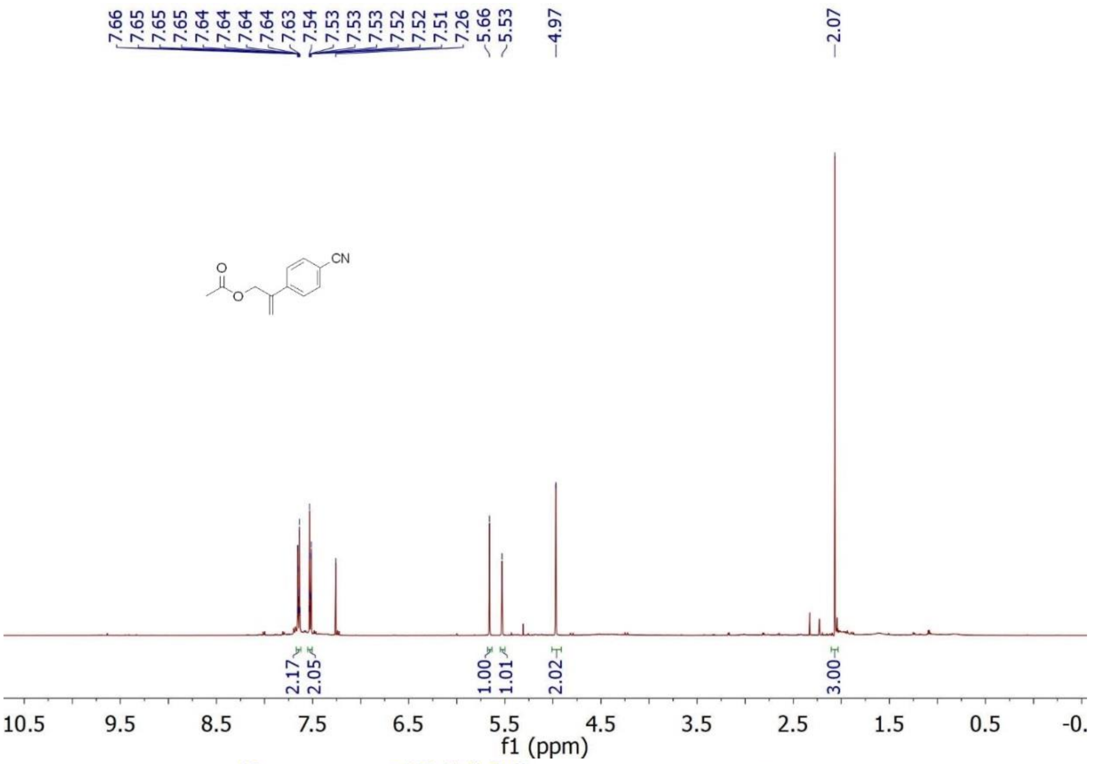

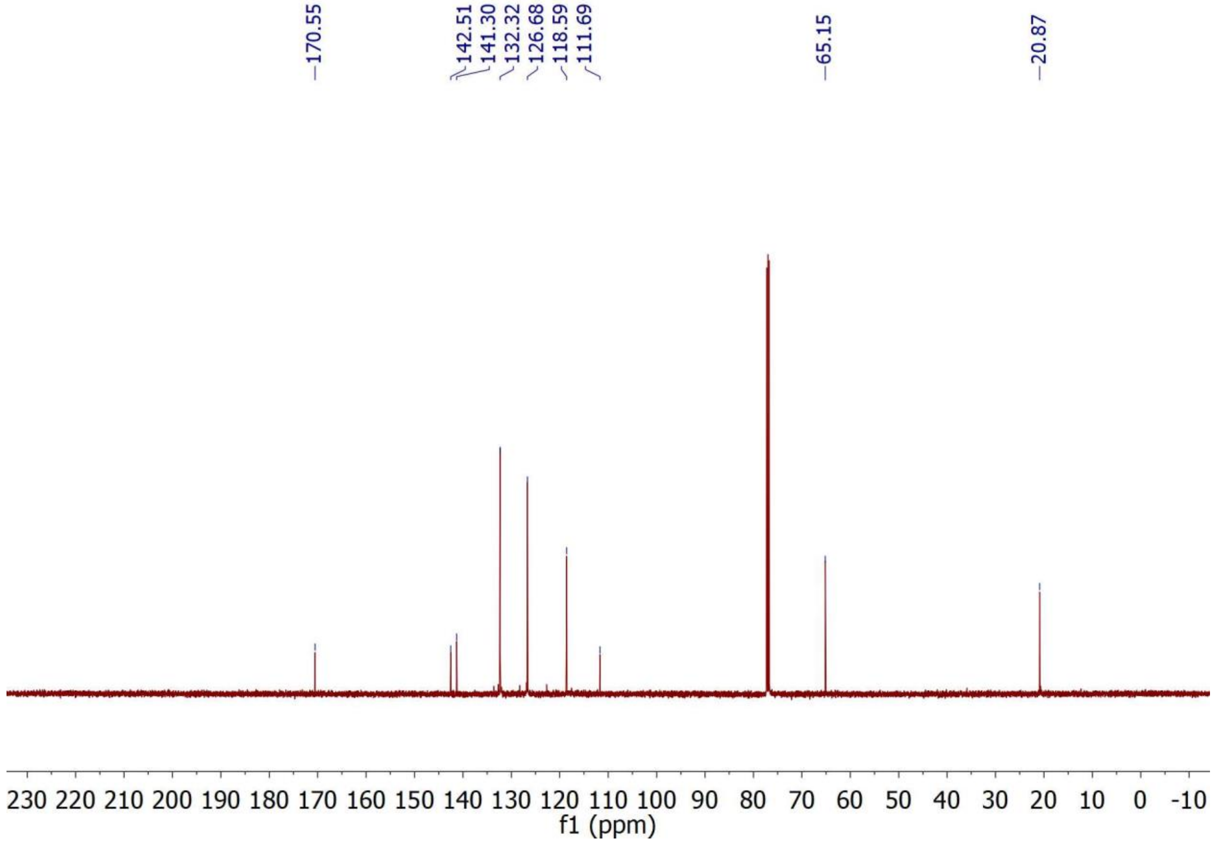

**2-(4-(2,2,2-trifluoroacetamido)phenyl)allyl acetate (2bh):**

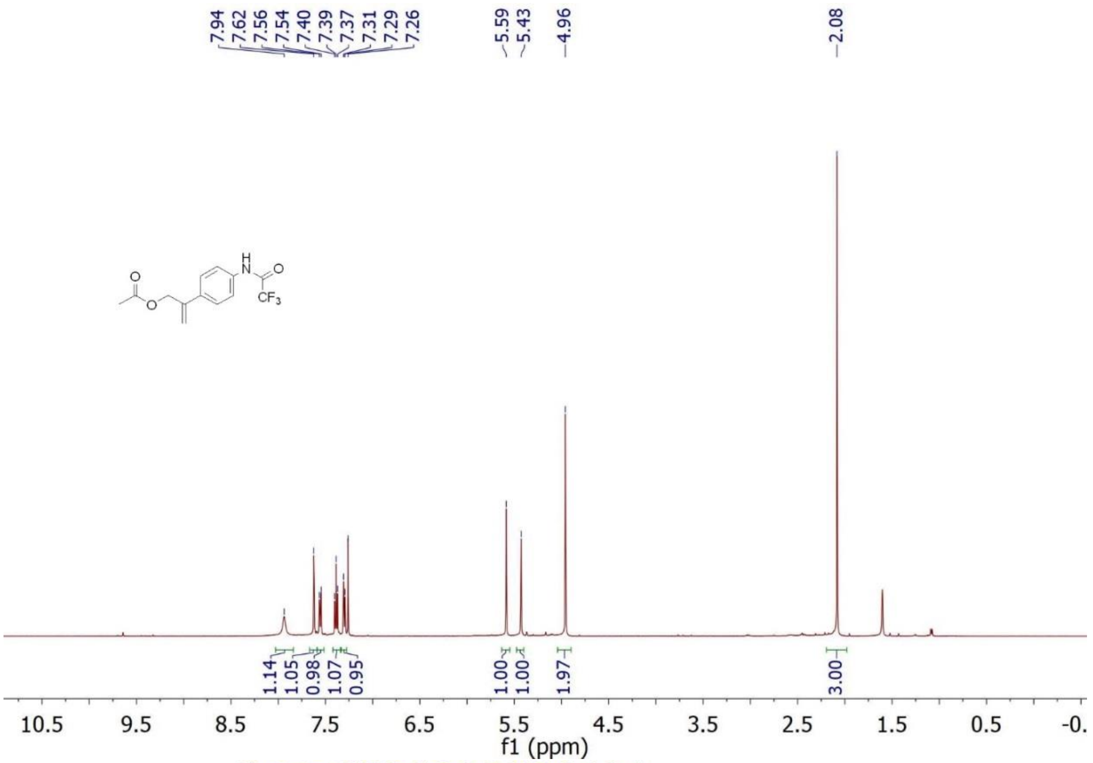


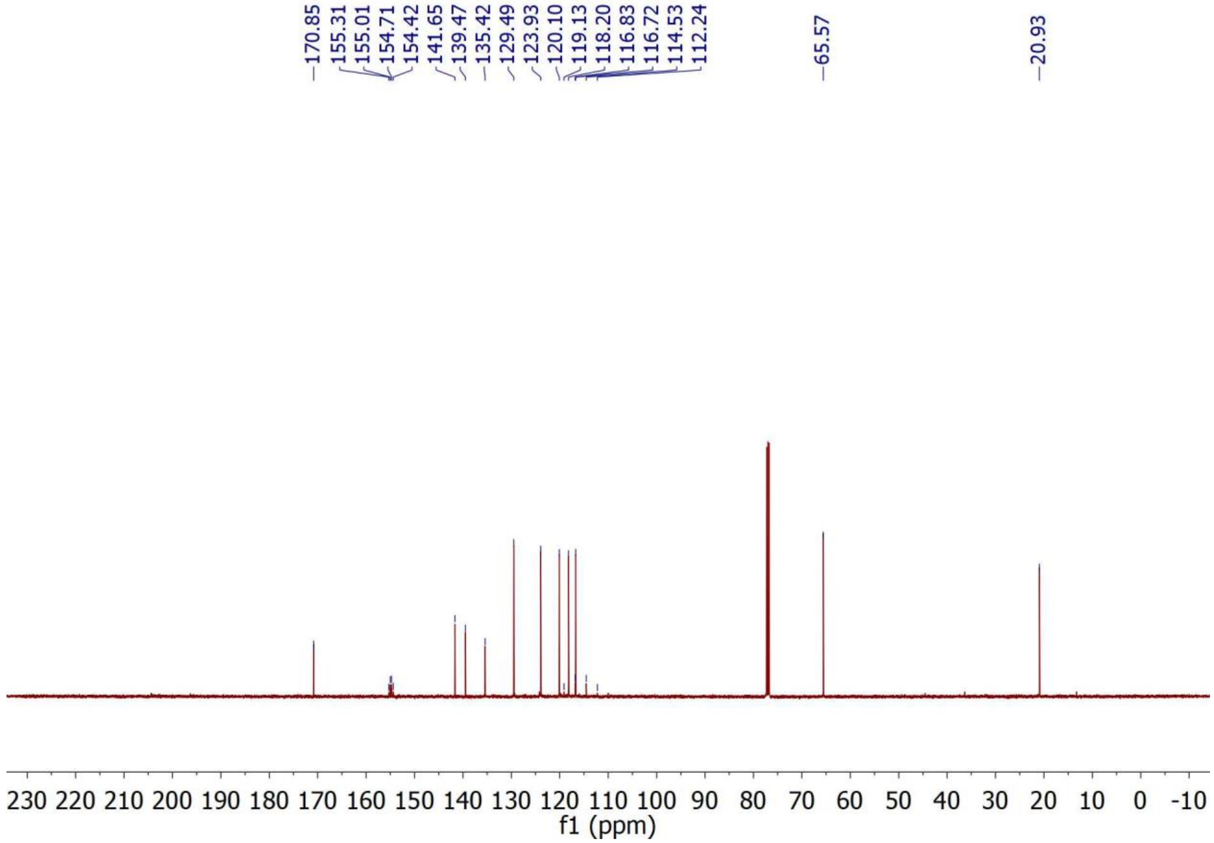


**2-(2-fluoro-4-methoxyphenyl)allyl acetate (2bi):**

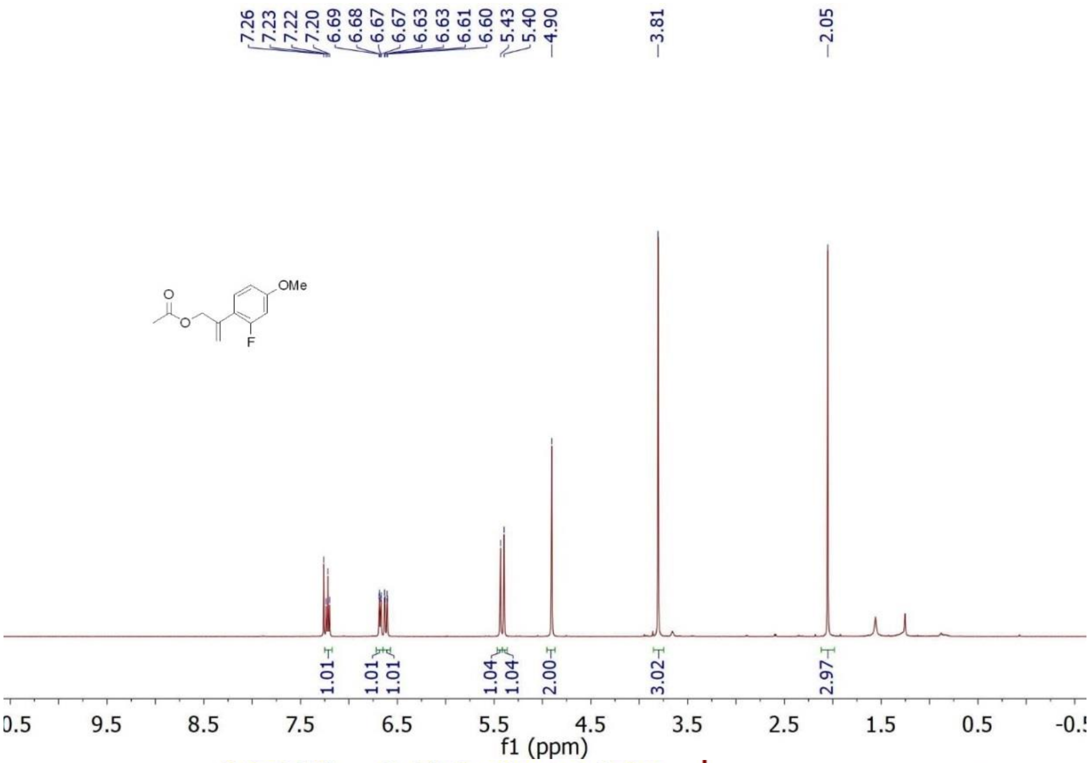


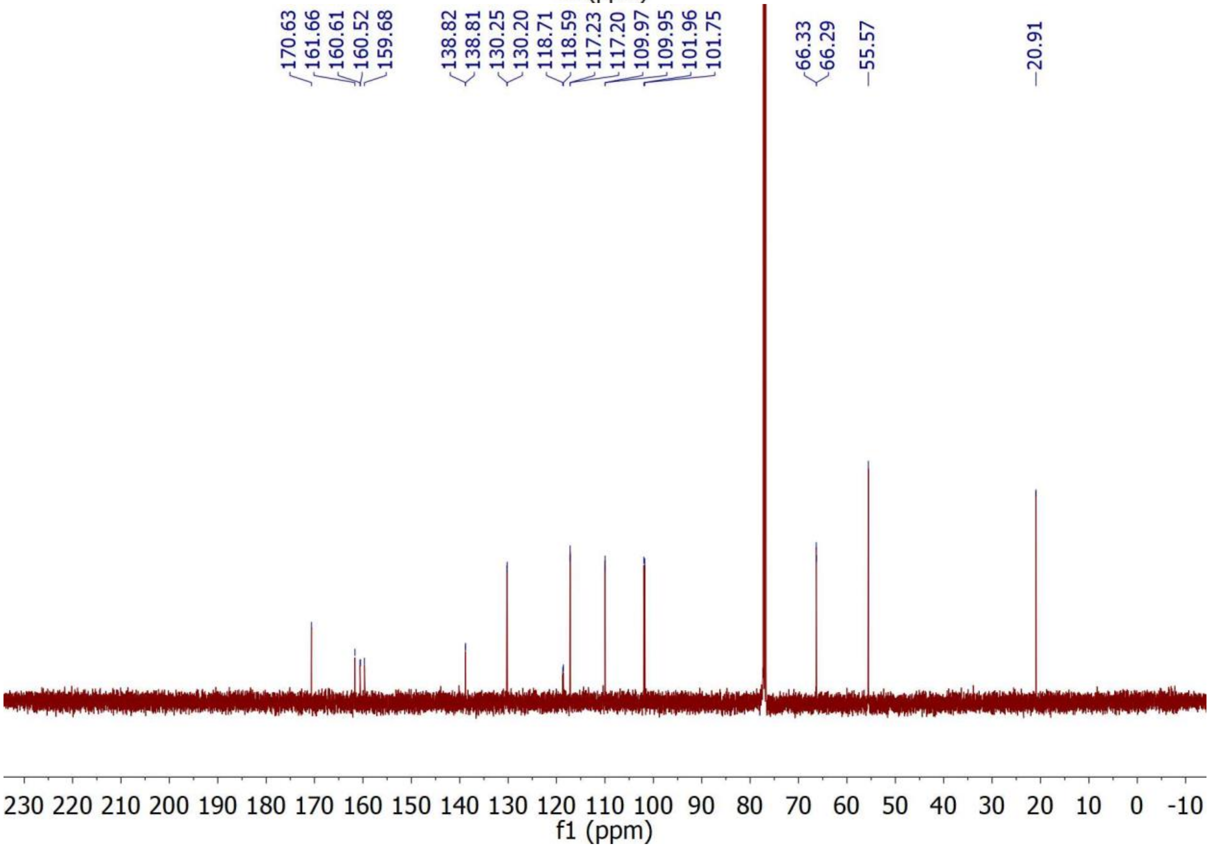


**2-(3-(trifluoromethyl)phenyl)allyl acetate (2bl):**

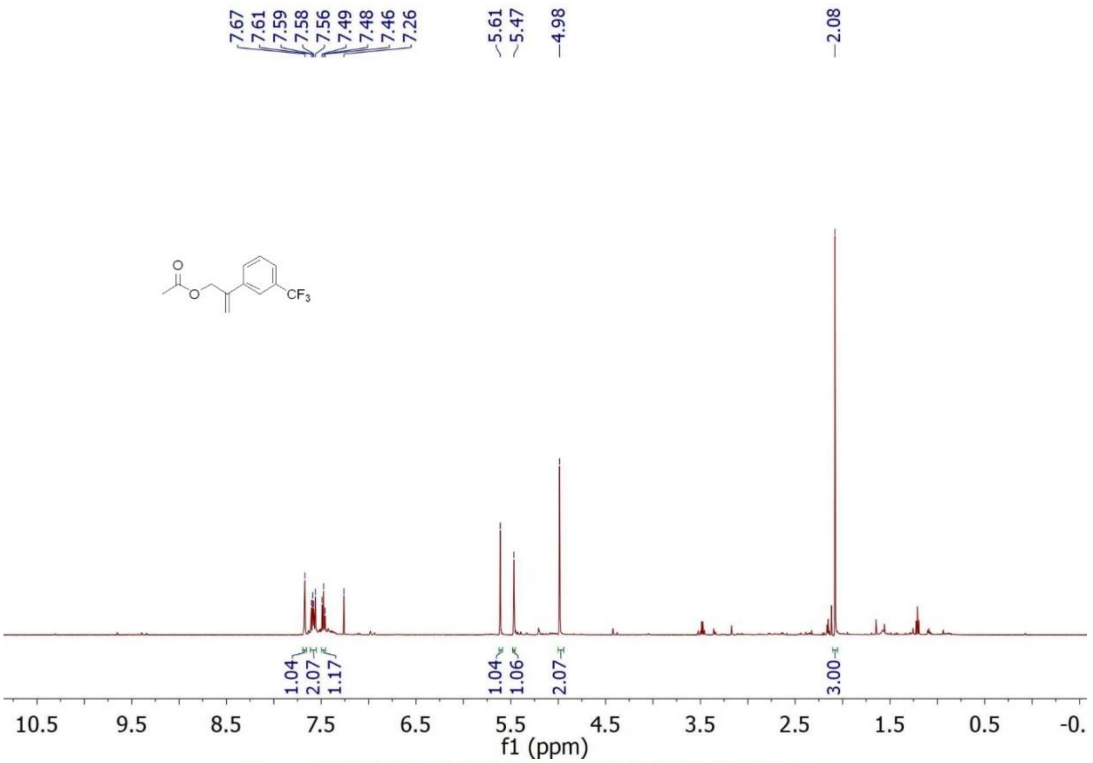


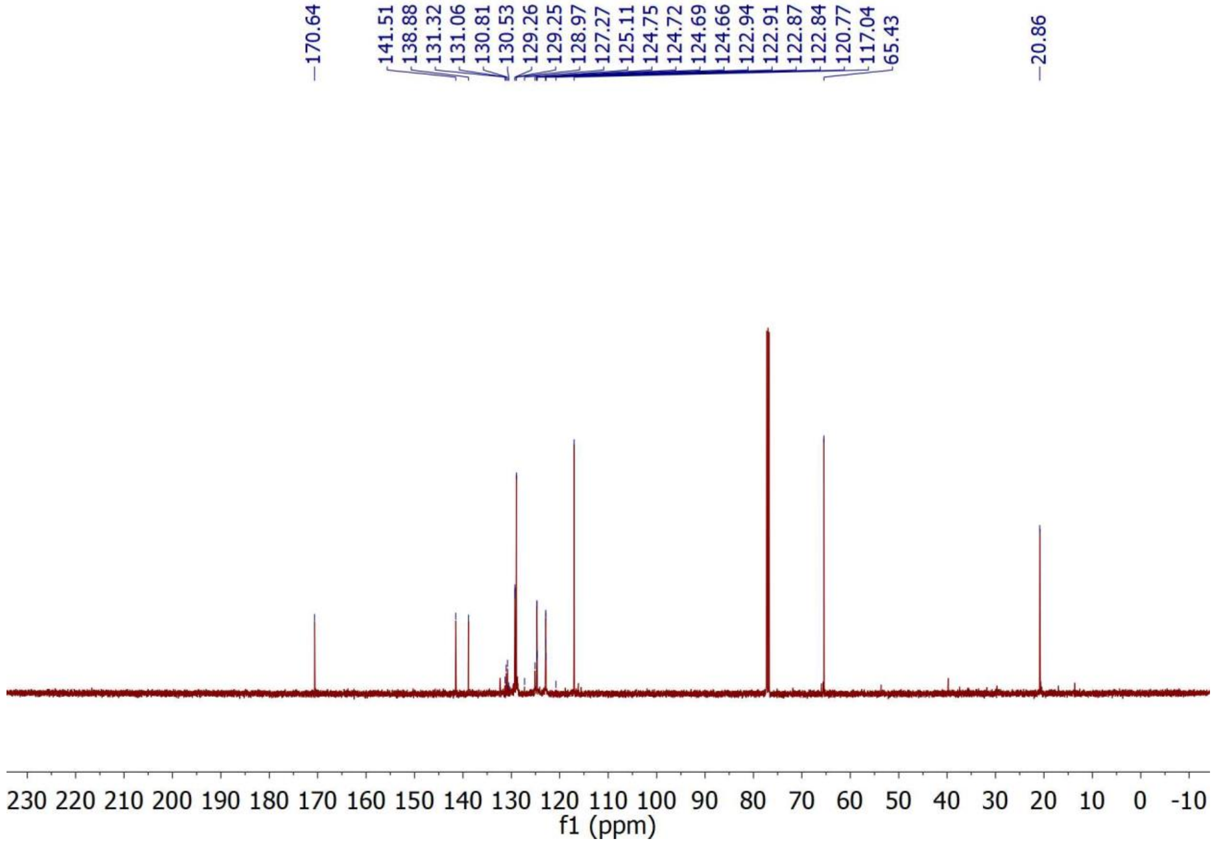


**2-benzylallyl acetate (2bm)**

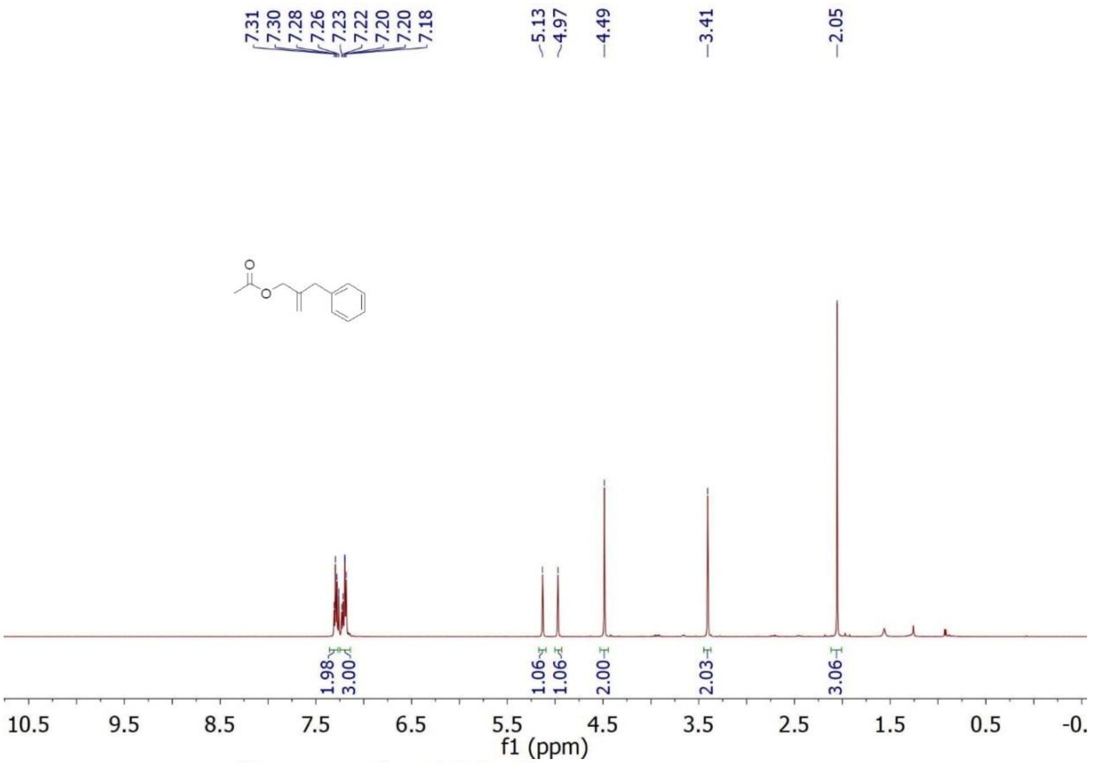


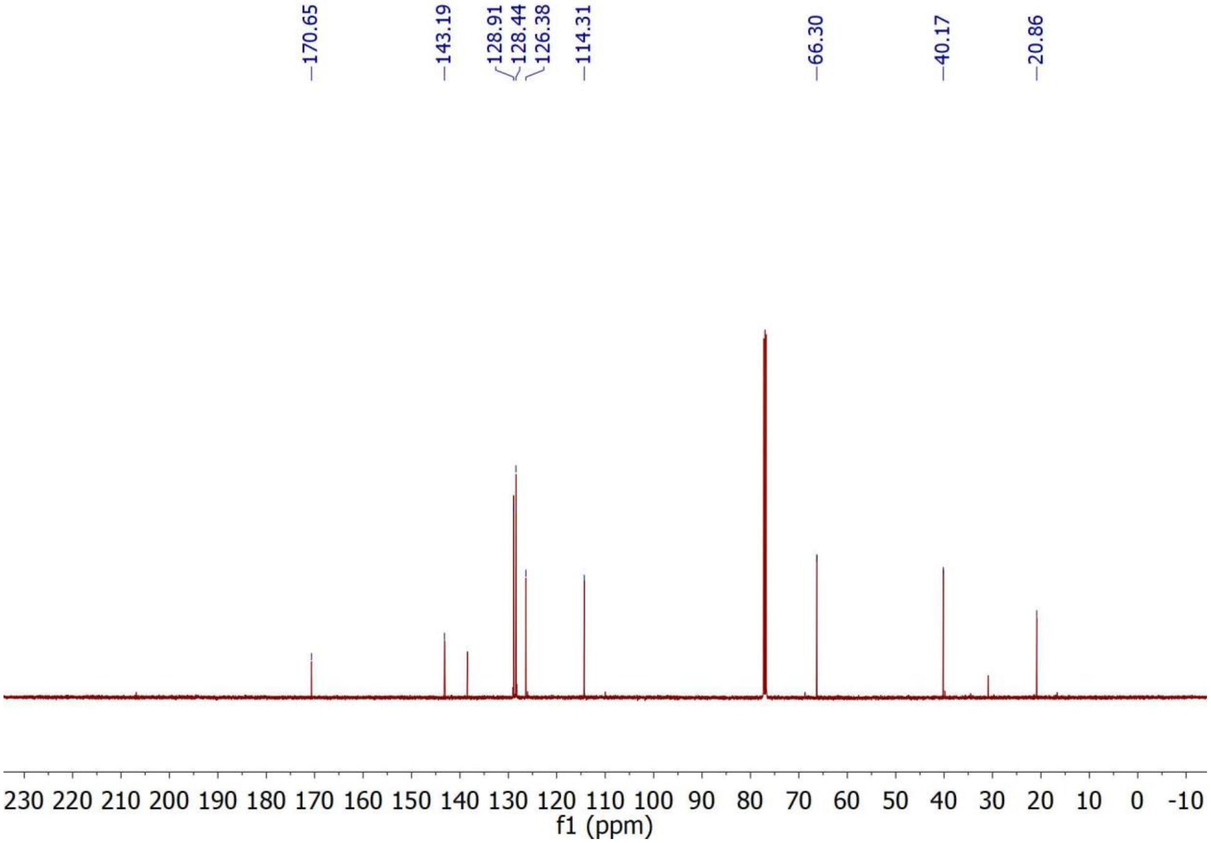


**2-(thiophen-2-yl)allyl acetate (2bo)**

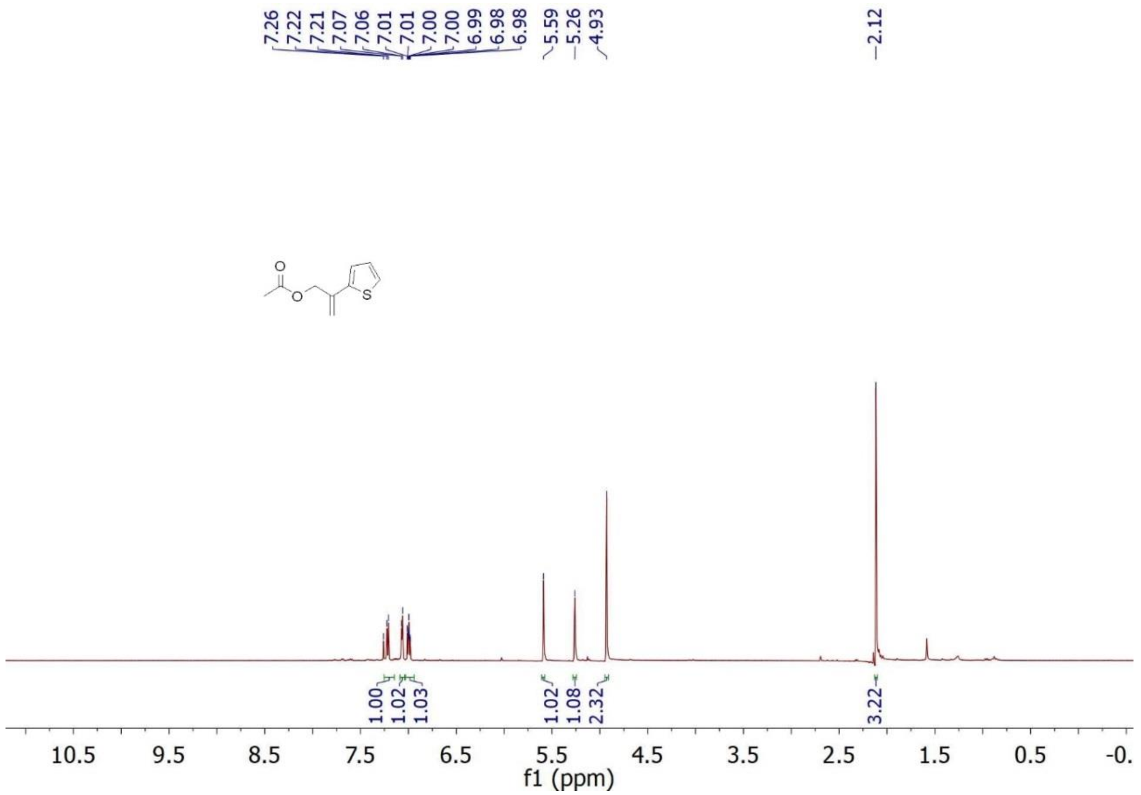

**2-methylene-4-(triisopropylsilyl)but-3-yn-1-yl acetate (2bp)**

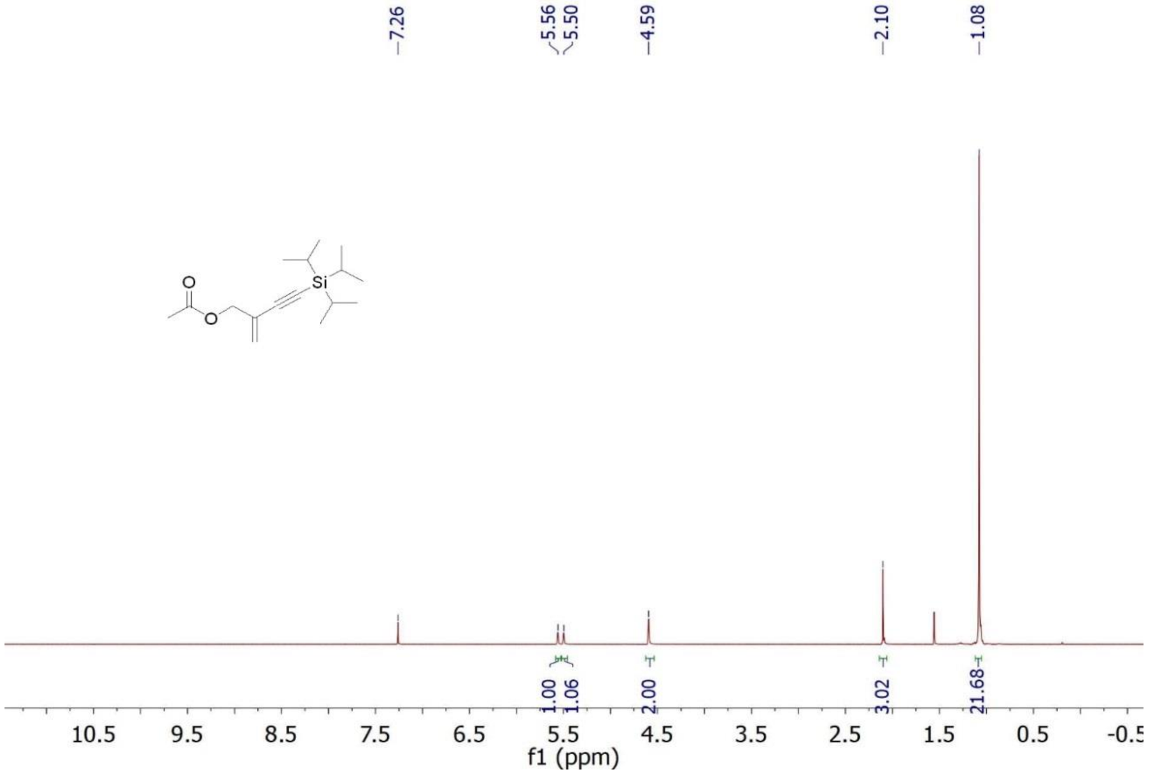

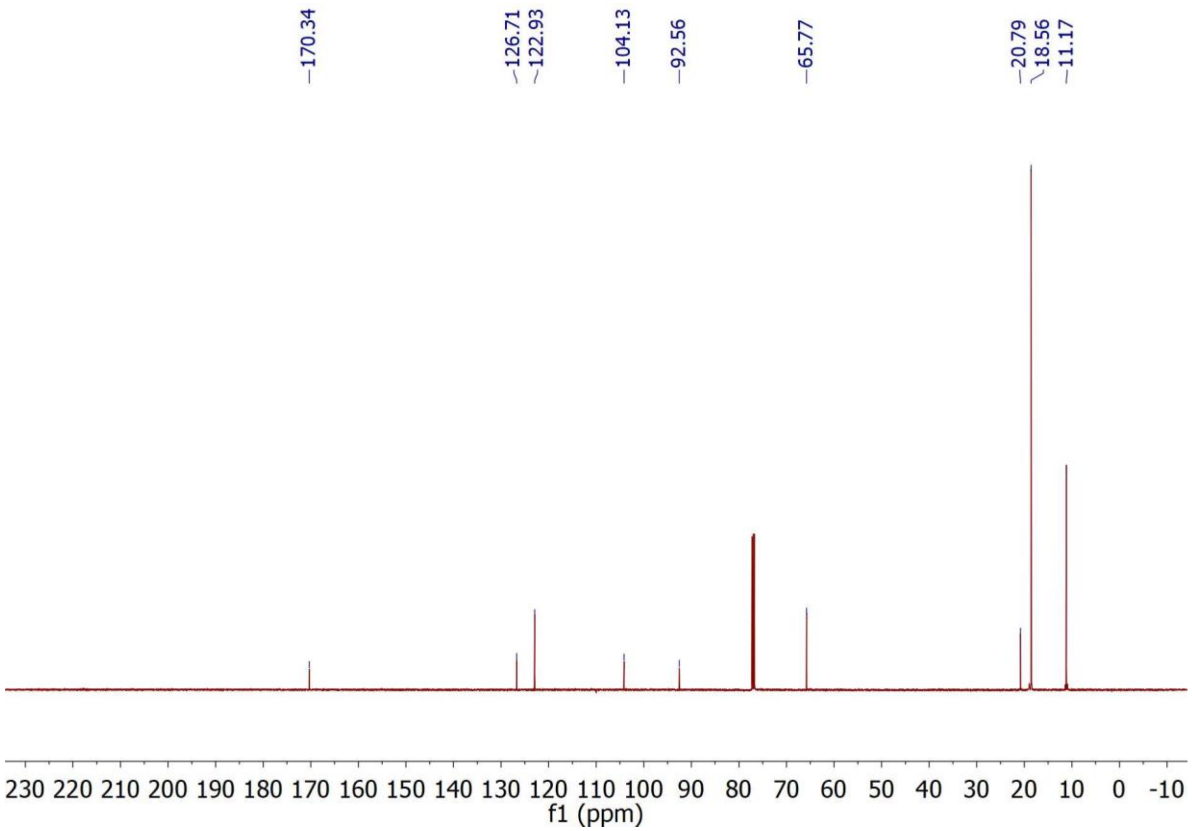


**1-phenylallyl acetate (2bq):**

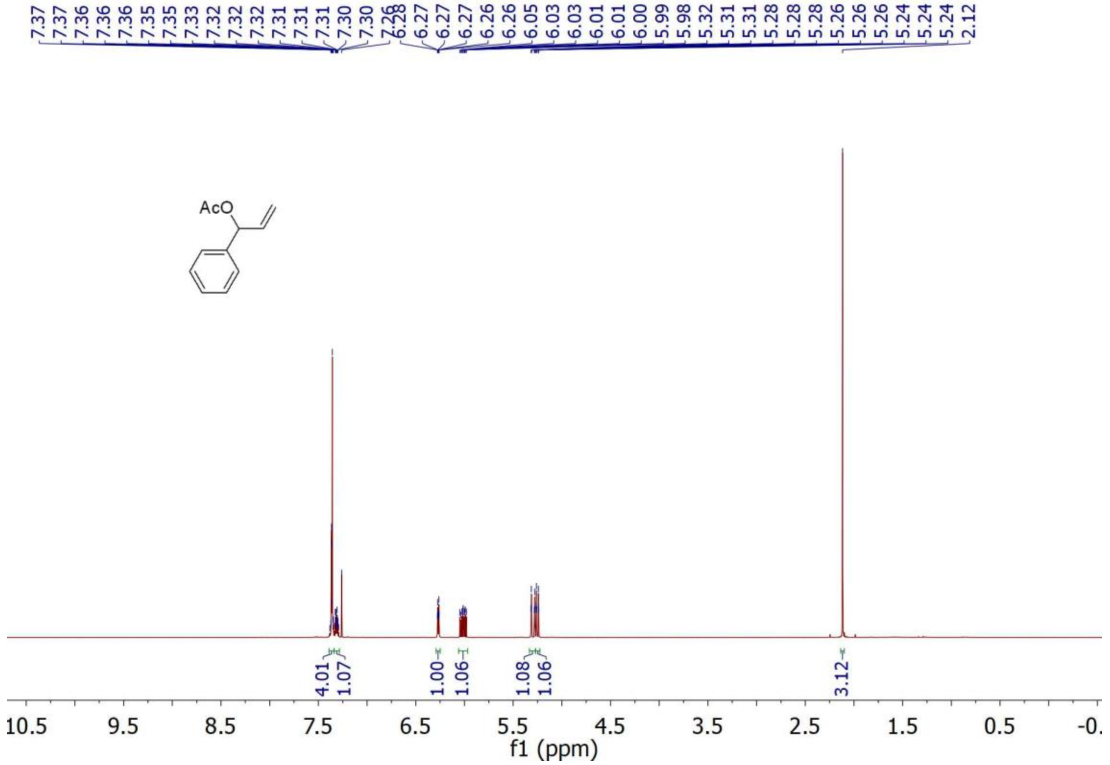

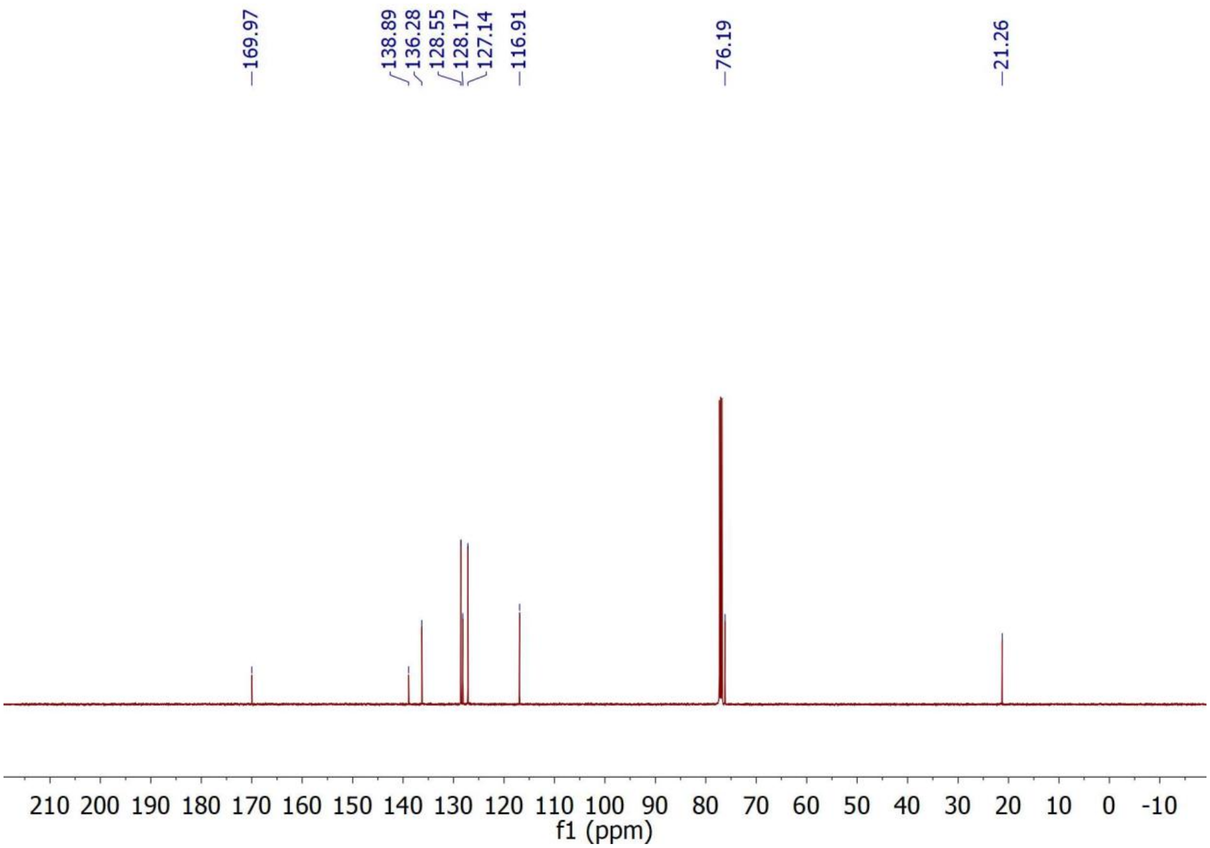


**1-(3-chlorophenyl)allyl acetate (2br):**

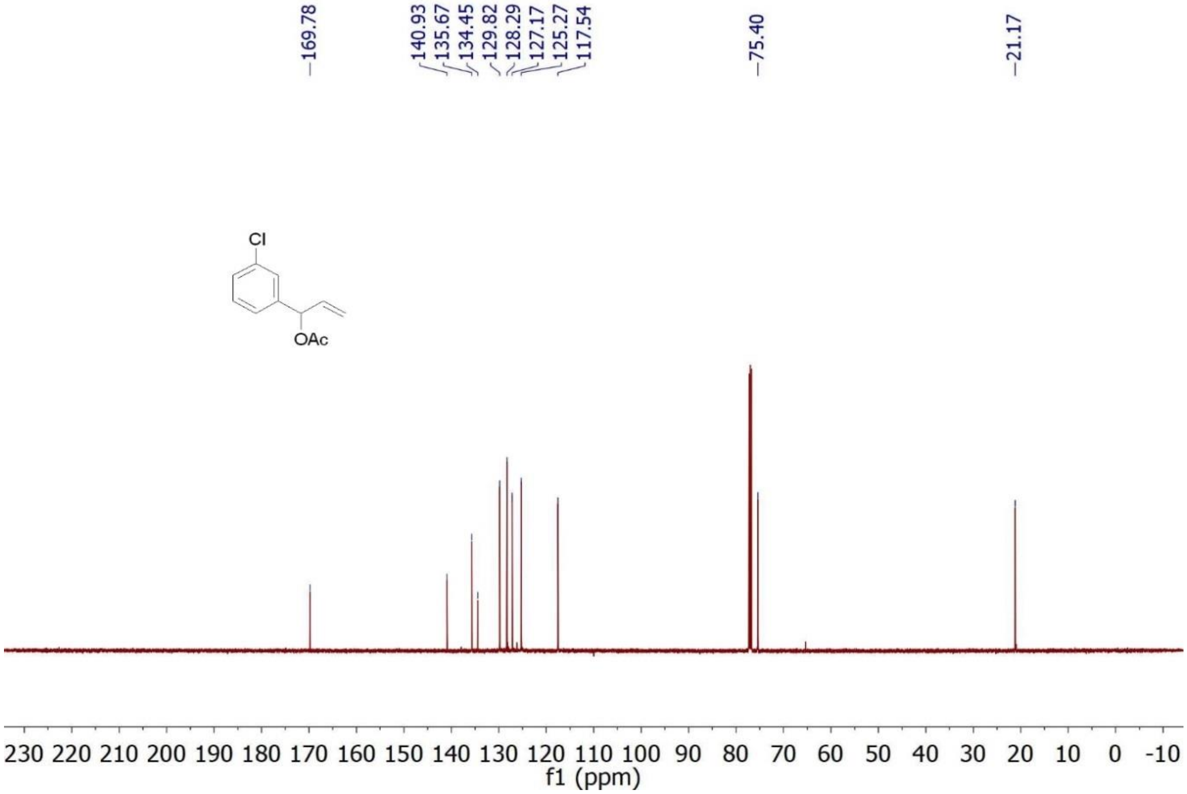

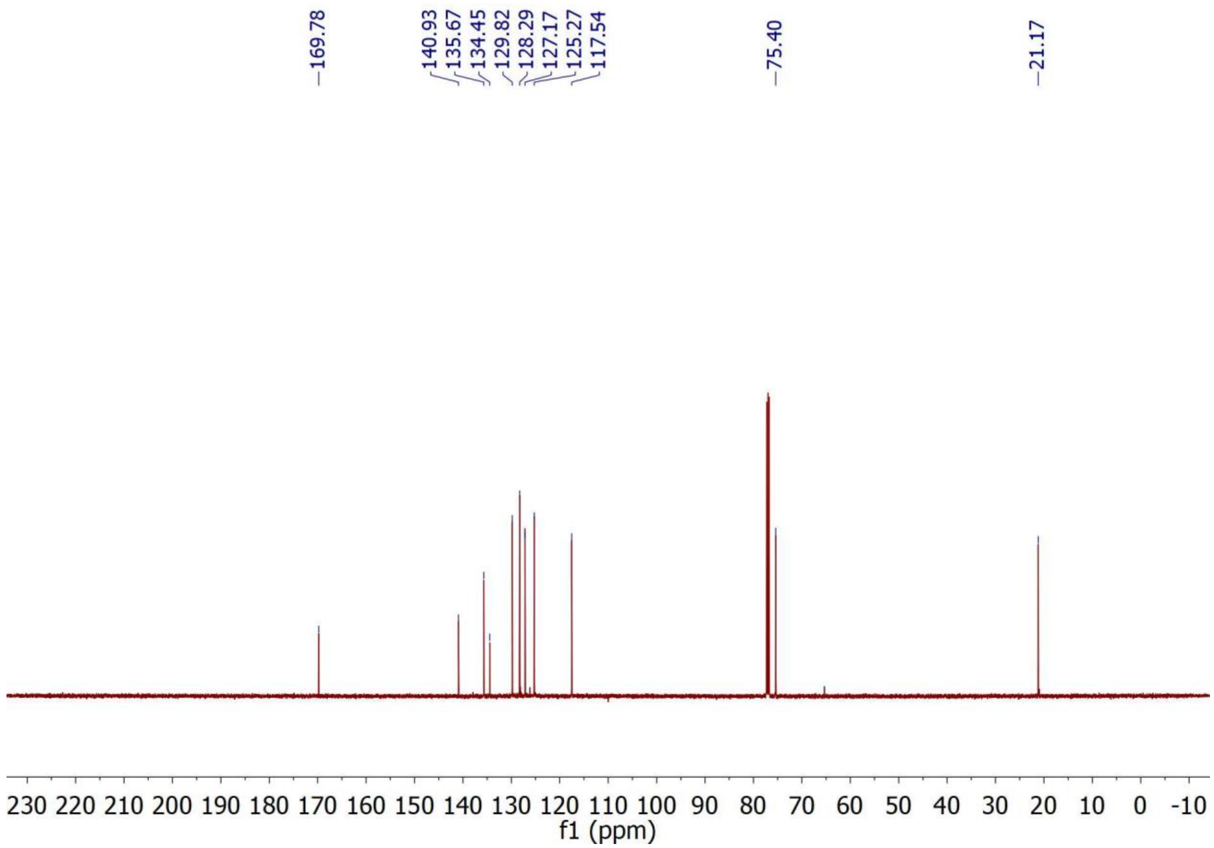


**1-(3-(trifluoromethyl)phenyl)allyl acetate (2bs):**

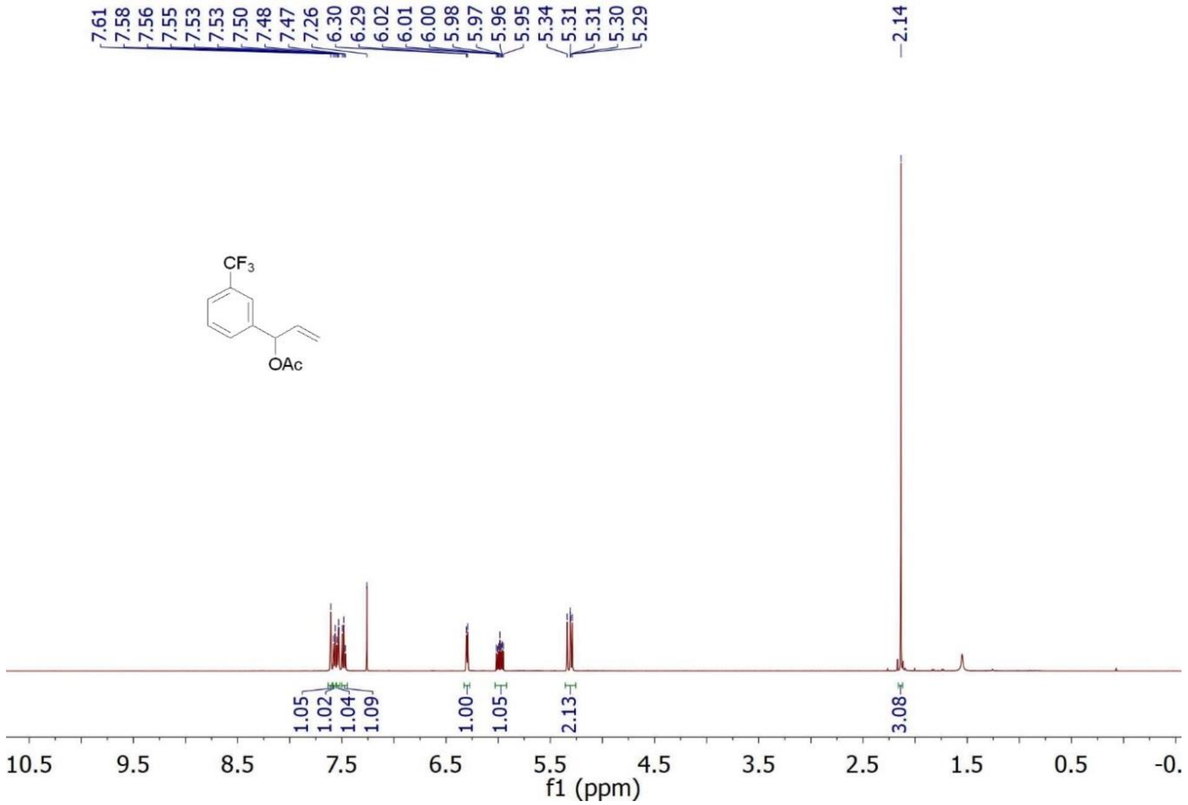

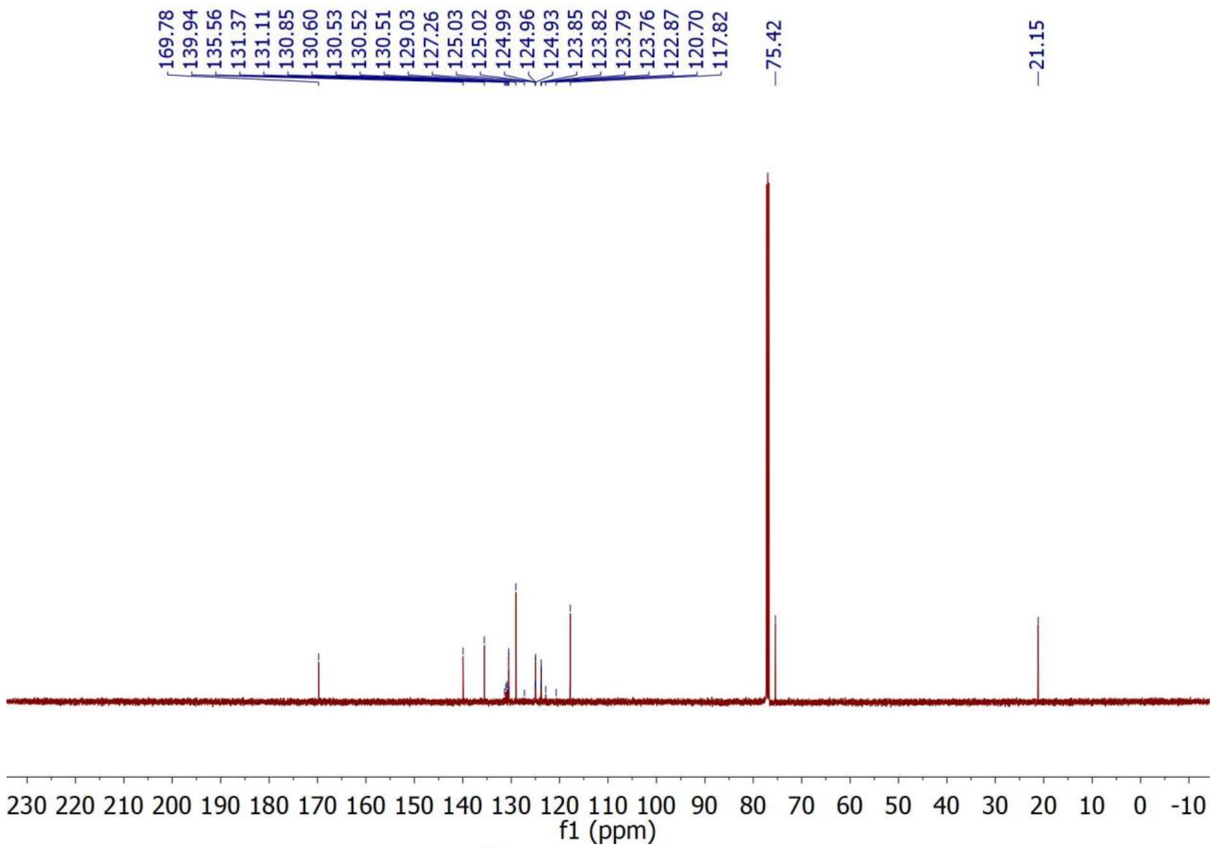


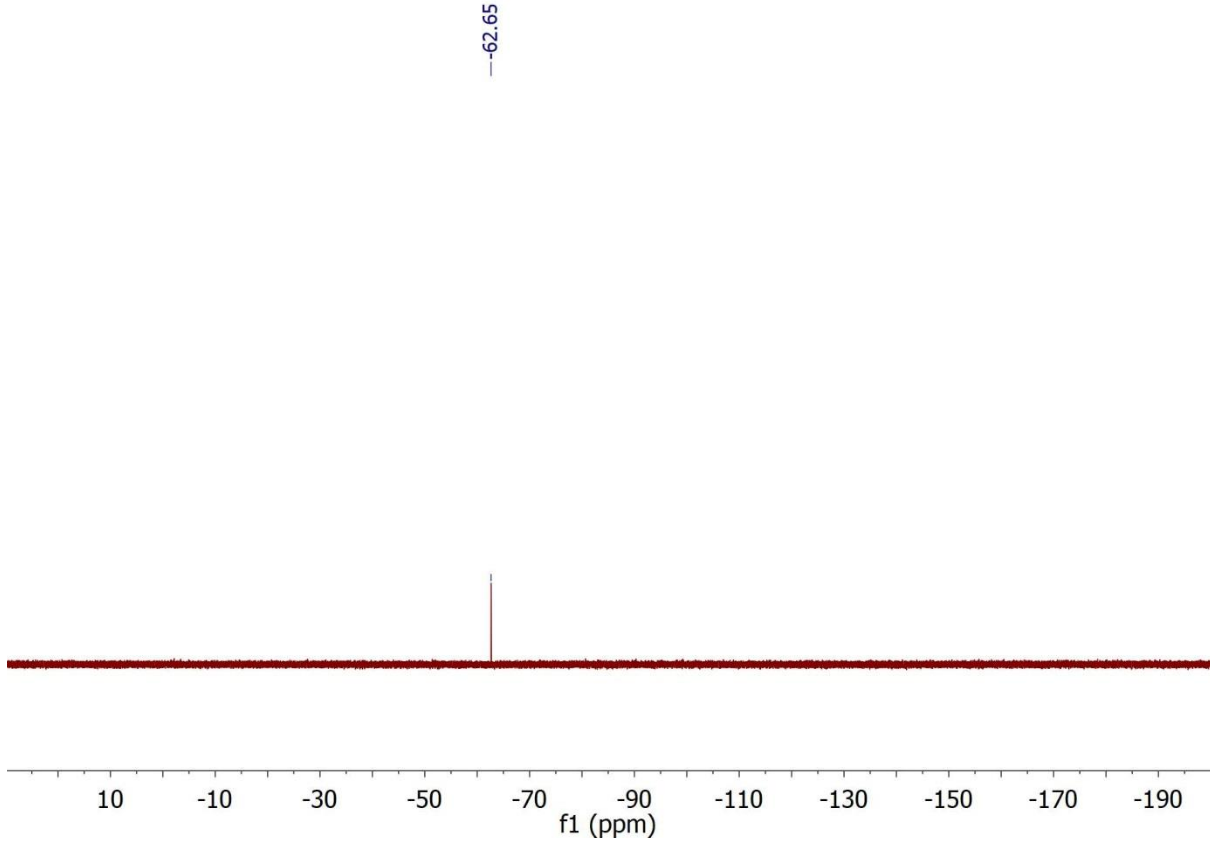


**1-(4-(methylthio)phenyl)allyl acetate (2bt):**

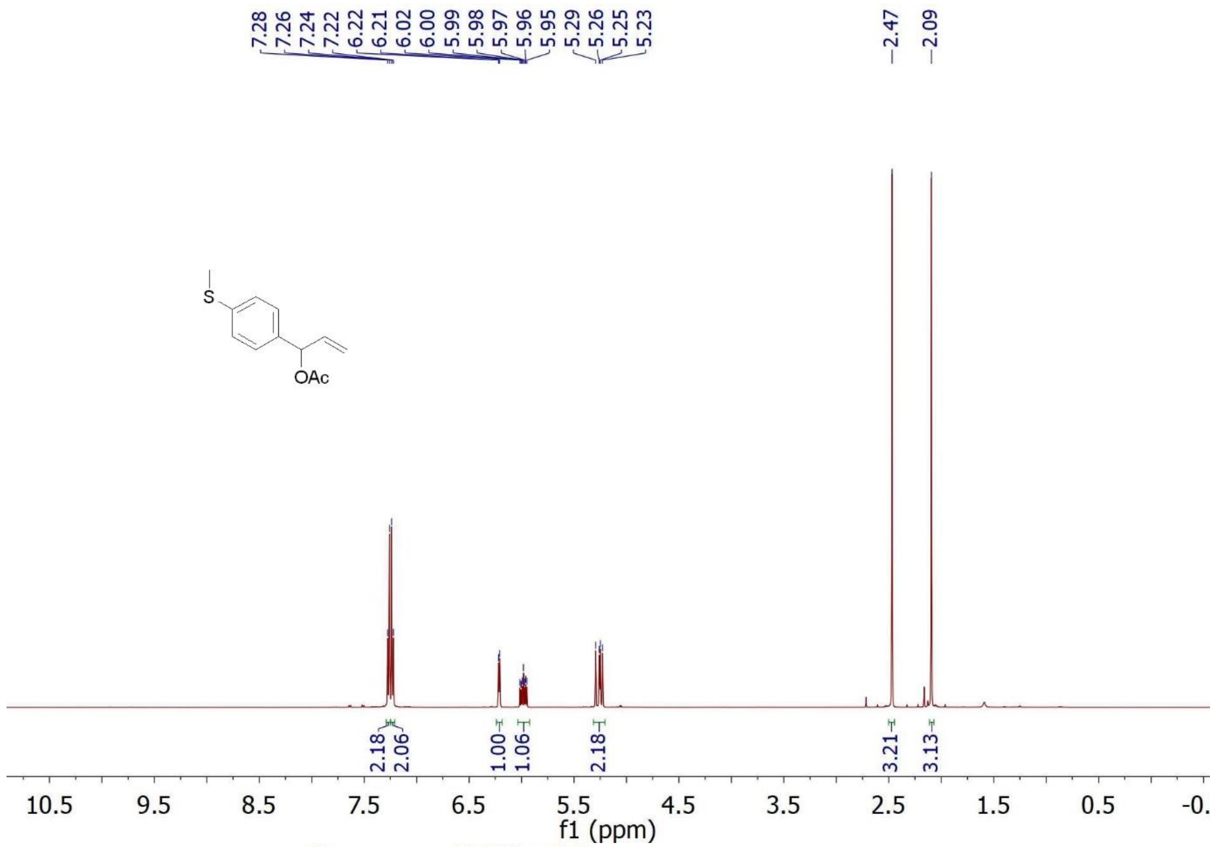


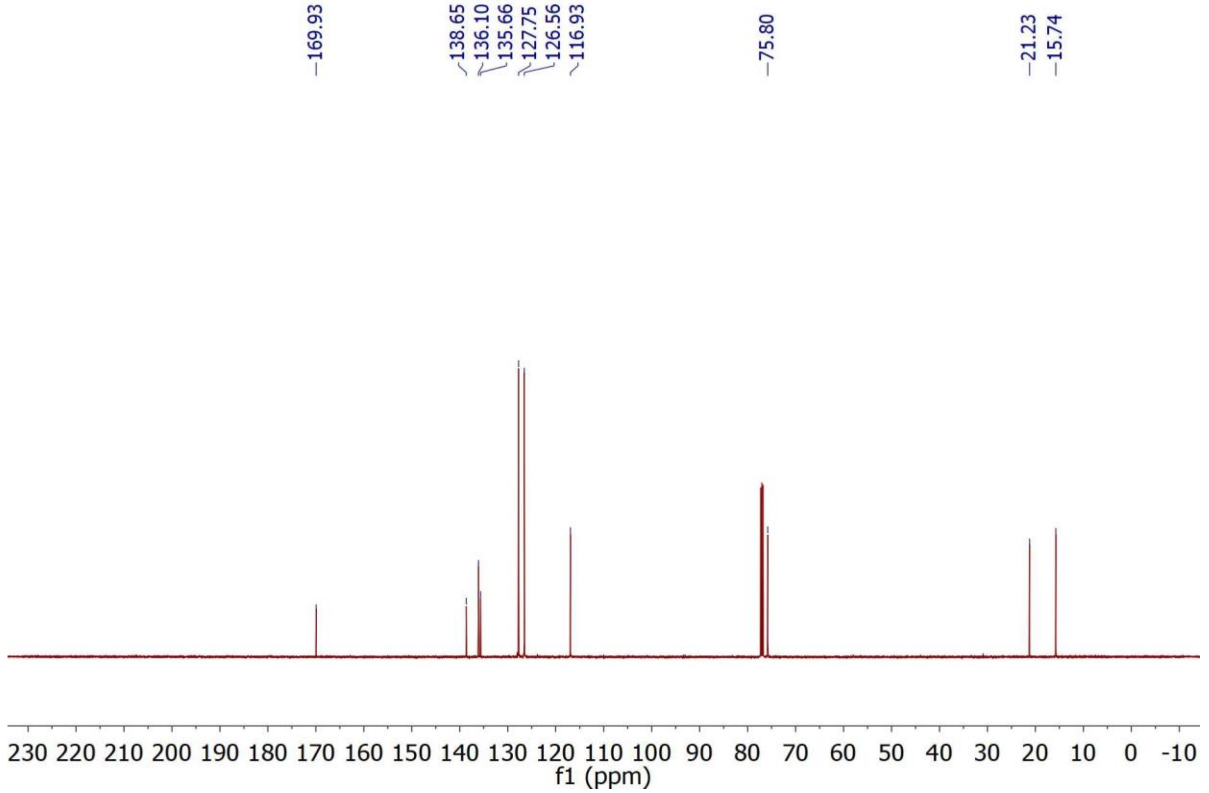


**1-(thiophen-3-yl)allyl acetate(2bu):**

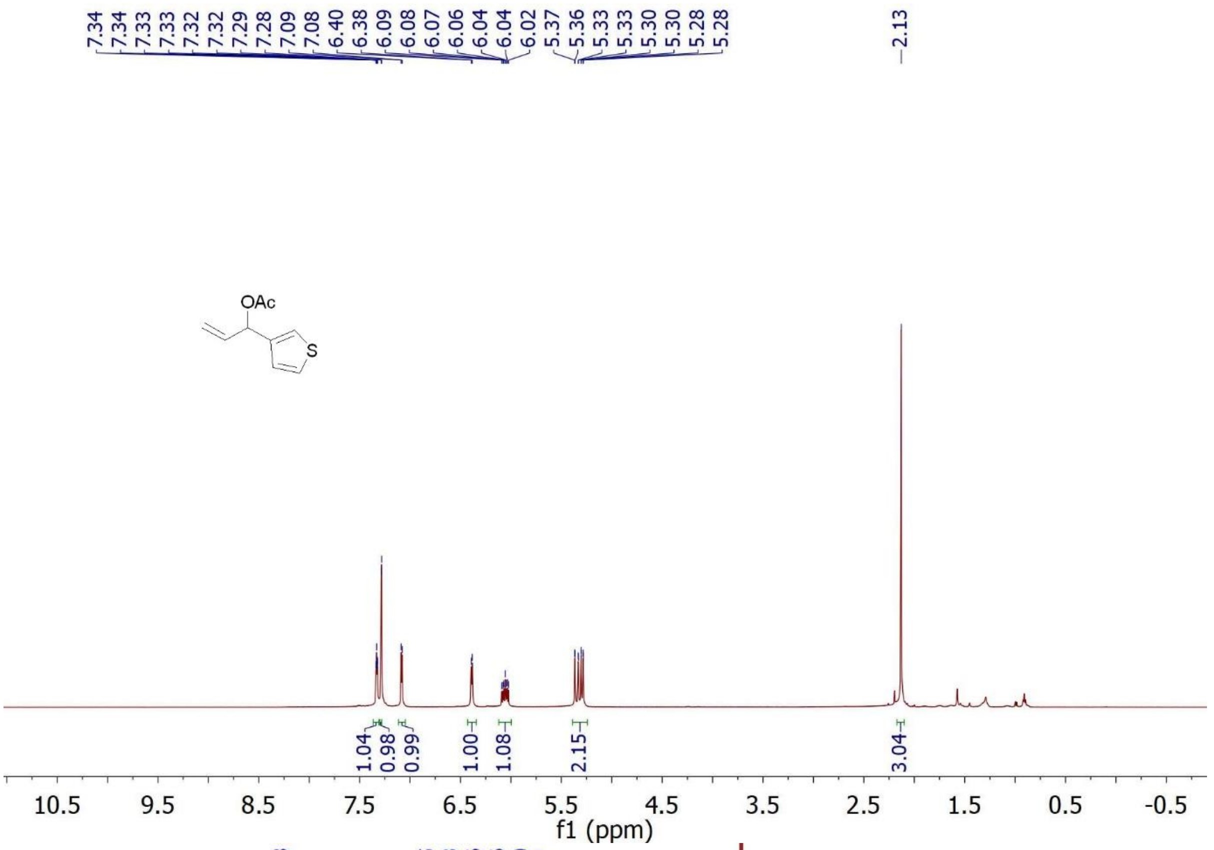


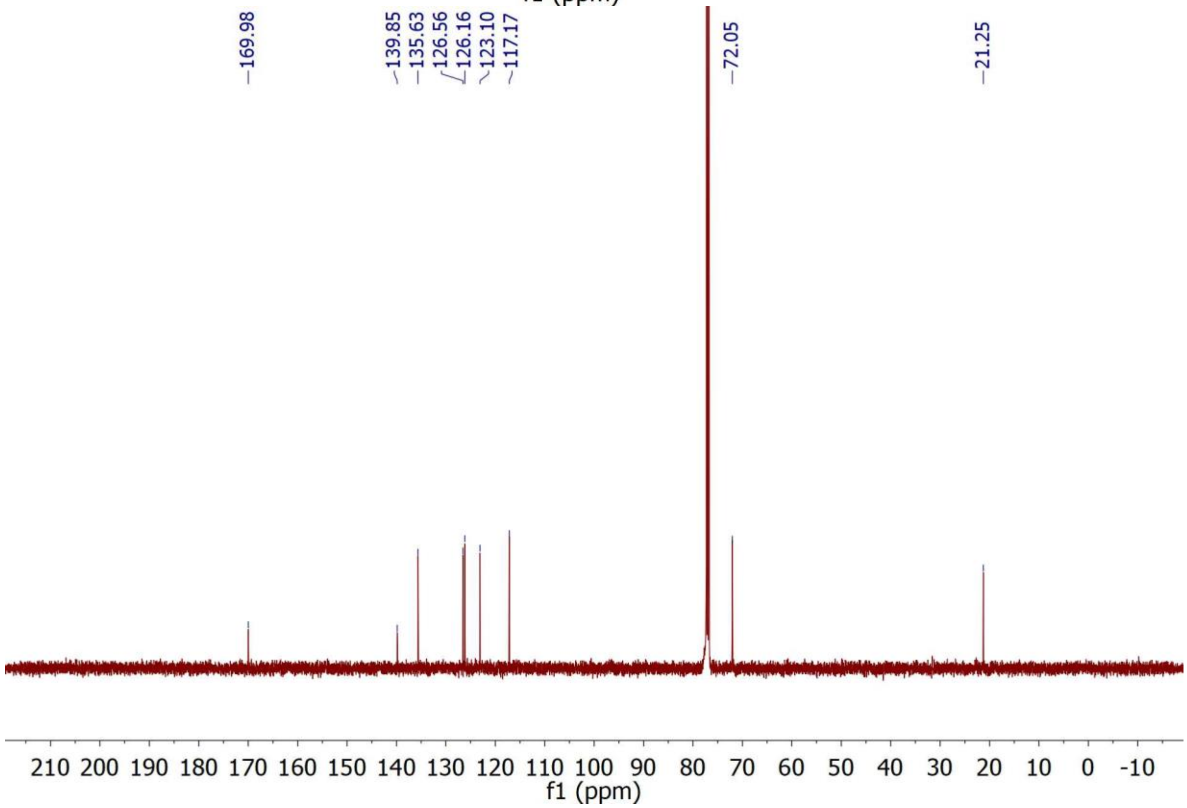


**2-((8R,9S,13S,14S)-13-methyl-17-oxo-7,8,9,11,12,13,14,15,16,17-decahydro-6H-cyclopenta[a]phenanthren-3-yl)allyl acetate (2bv):**

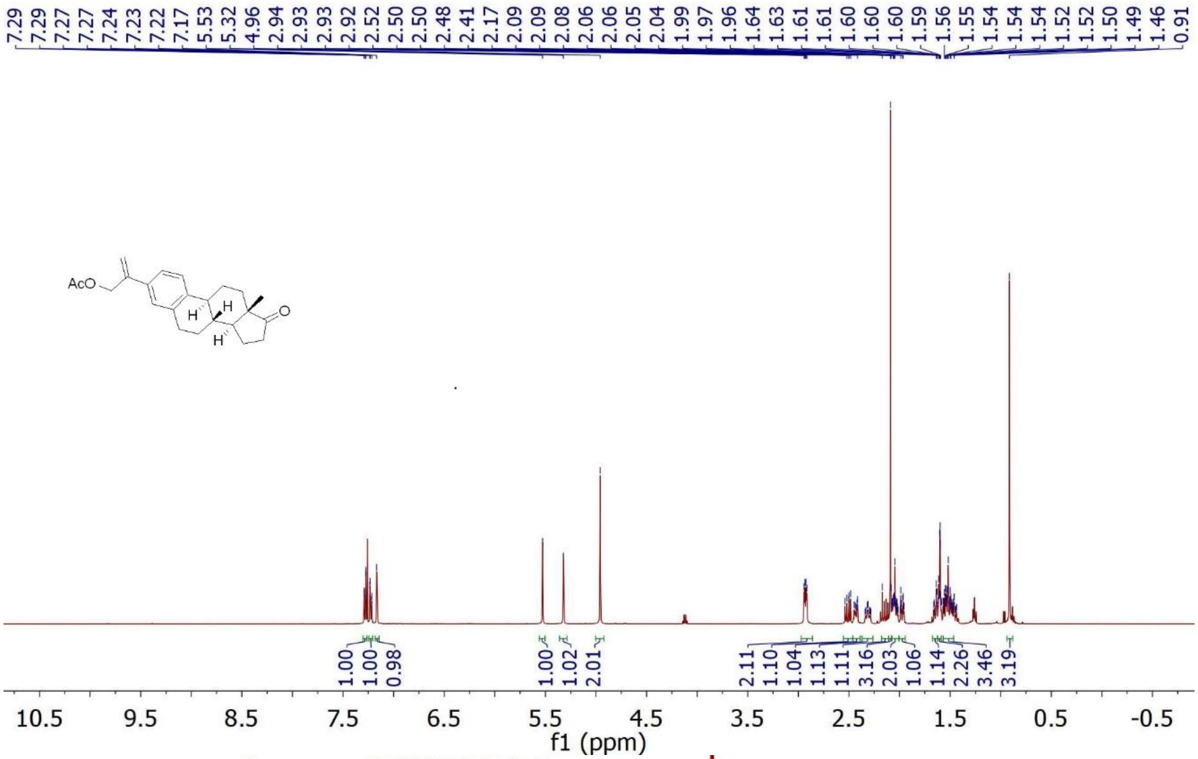


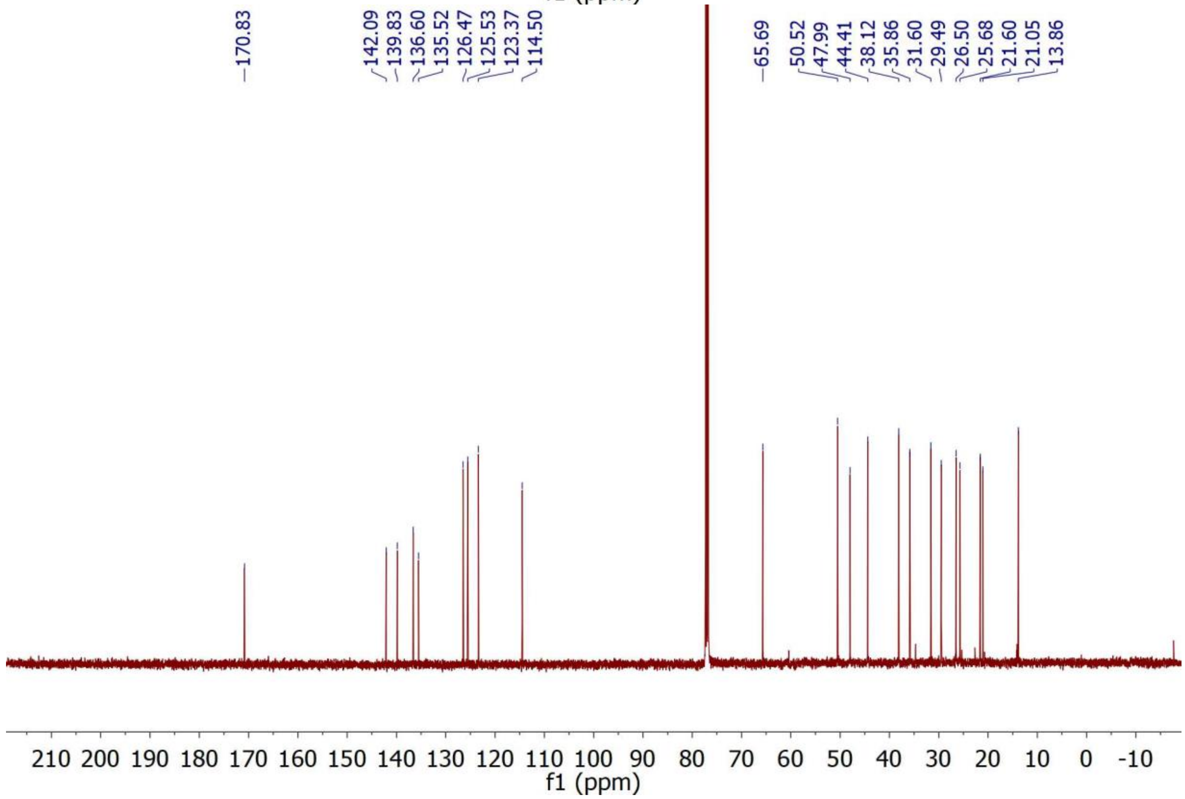


**methyl (S)-3-(4-(3-acetoxyprop-1-en-2-yl)phenyl)-2-((tert-utoxycarbonyl)amino)propanoate (2bw):**

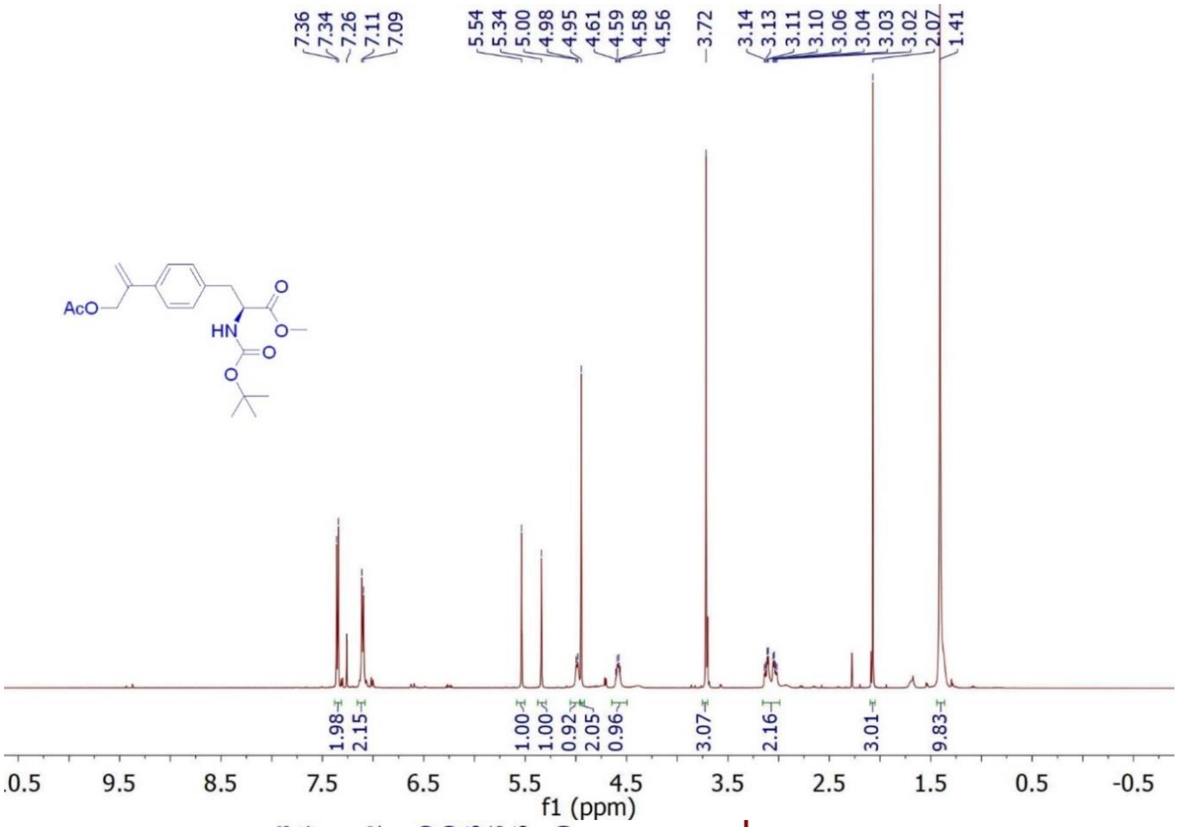


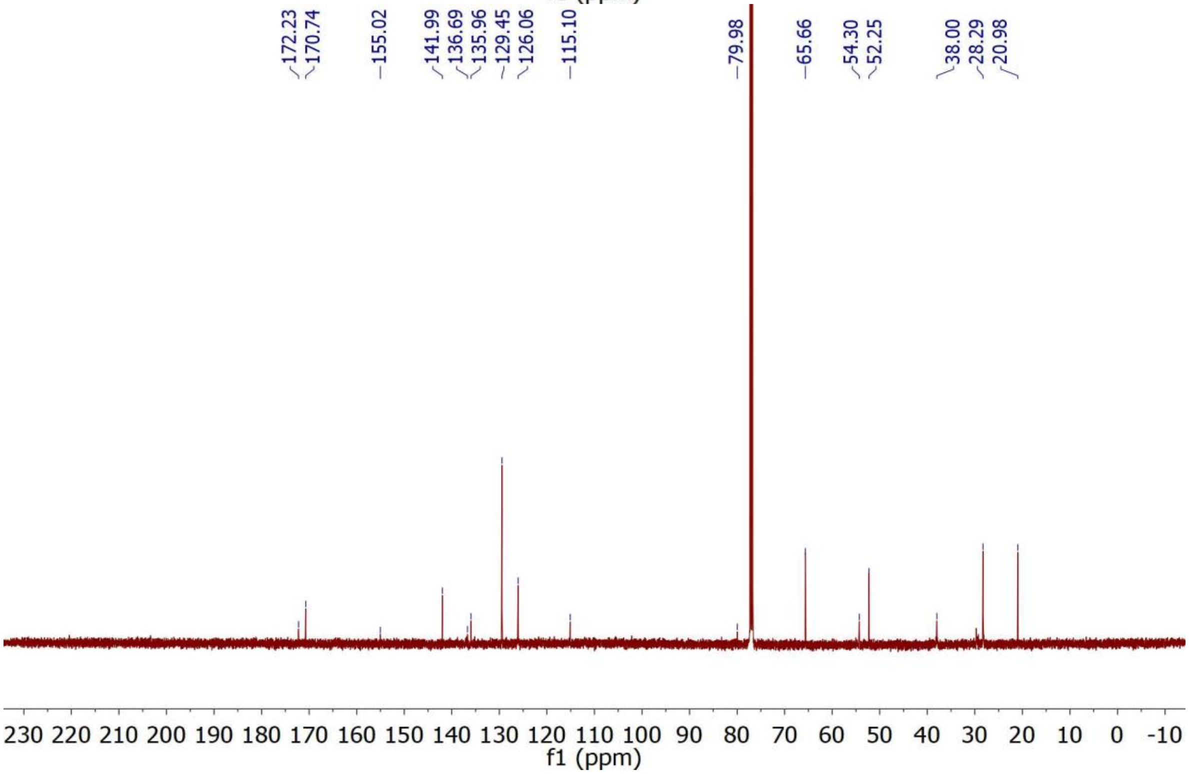


**1,4-phenylenebis(prop-2-ene-2,1-diyl) diacetate (2bx):**

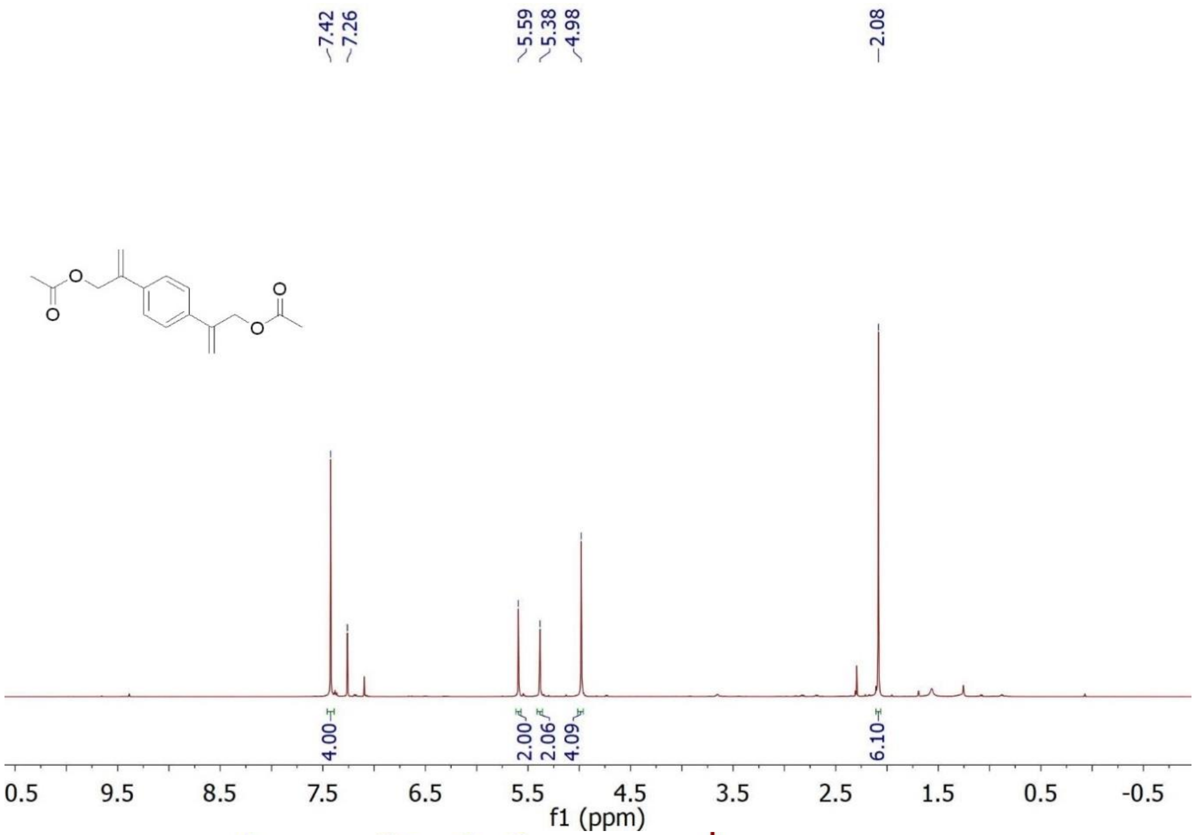


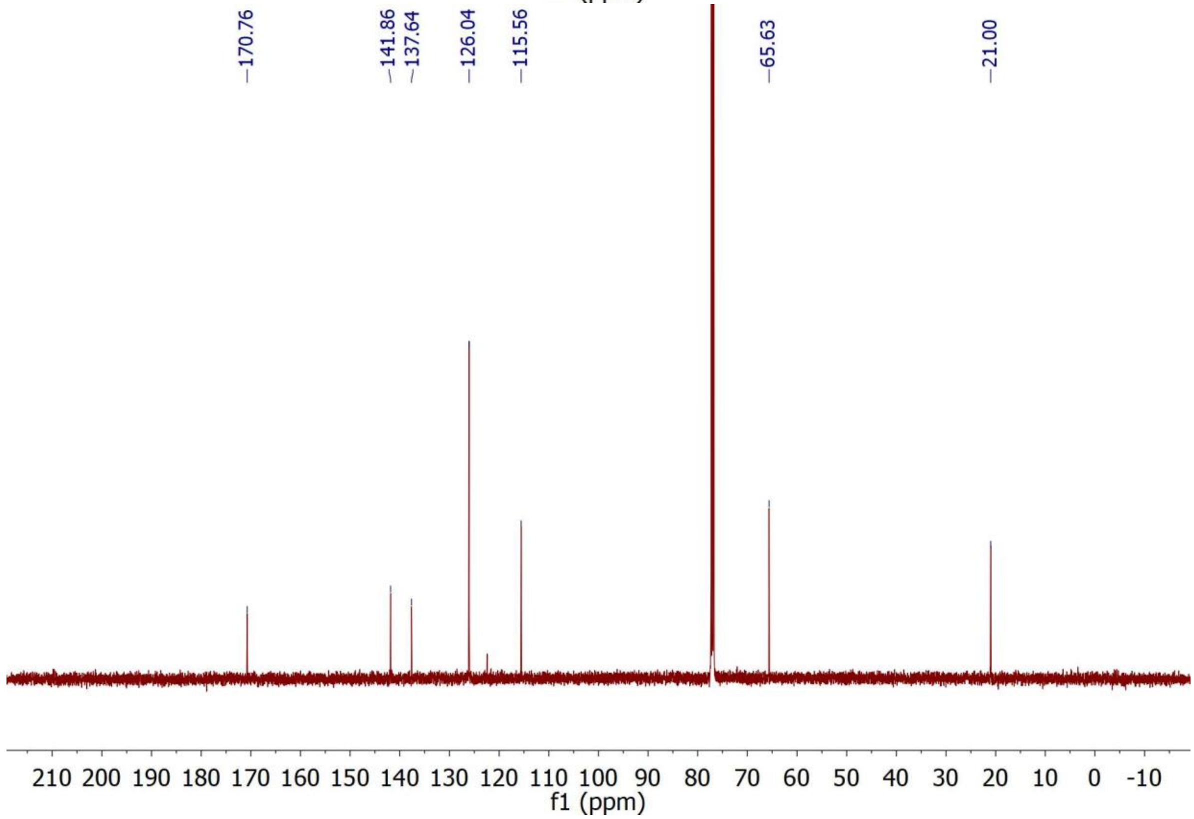


**(R)-6-vinyl-5,6-dihydro-2H-pyran-2-one (2by):**

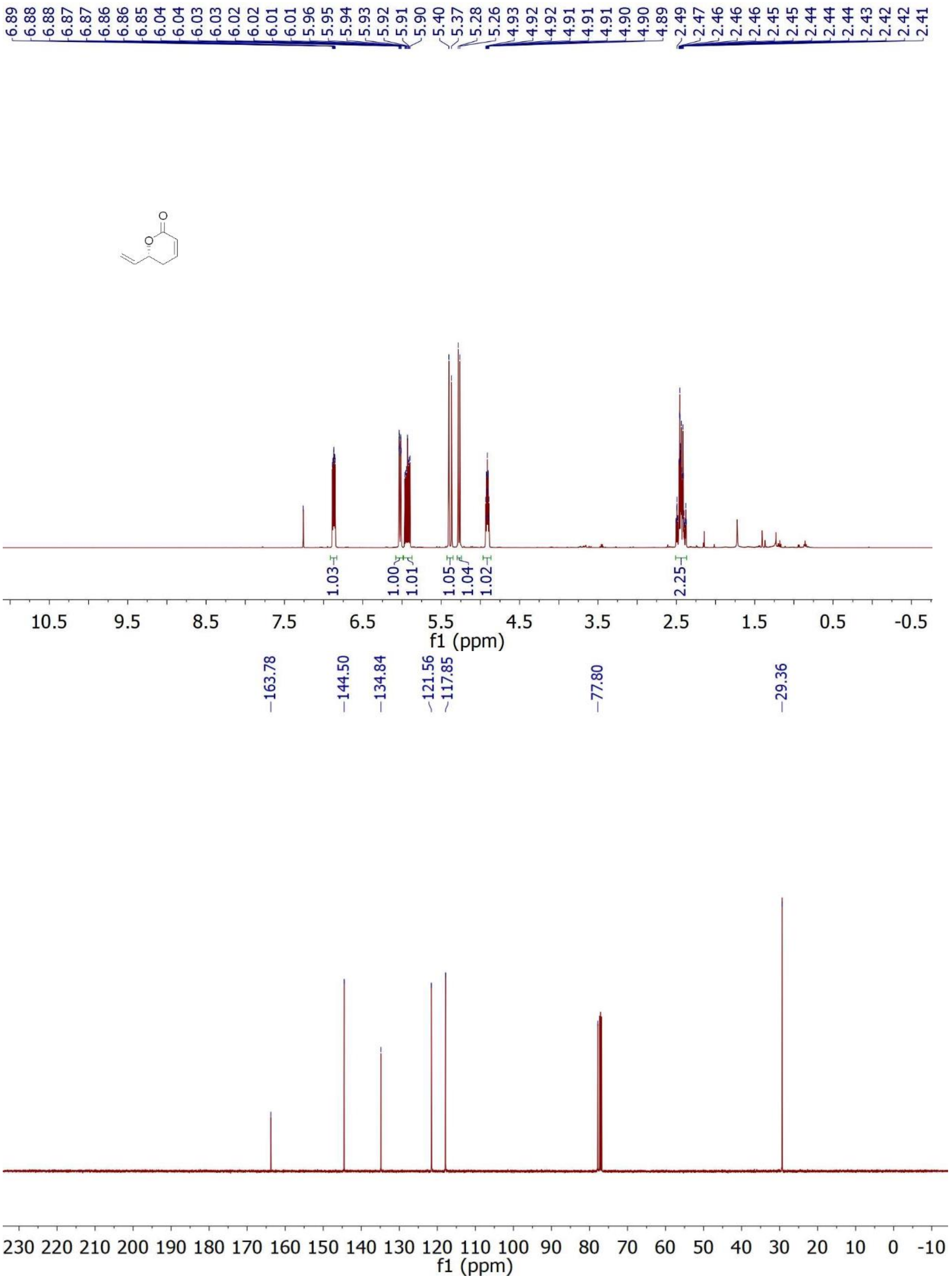


**(5*S*,6*S*,7*R*)-5,6,7,8-tetramethoxyoct-1-ene (3aa):**

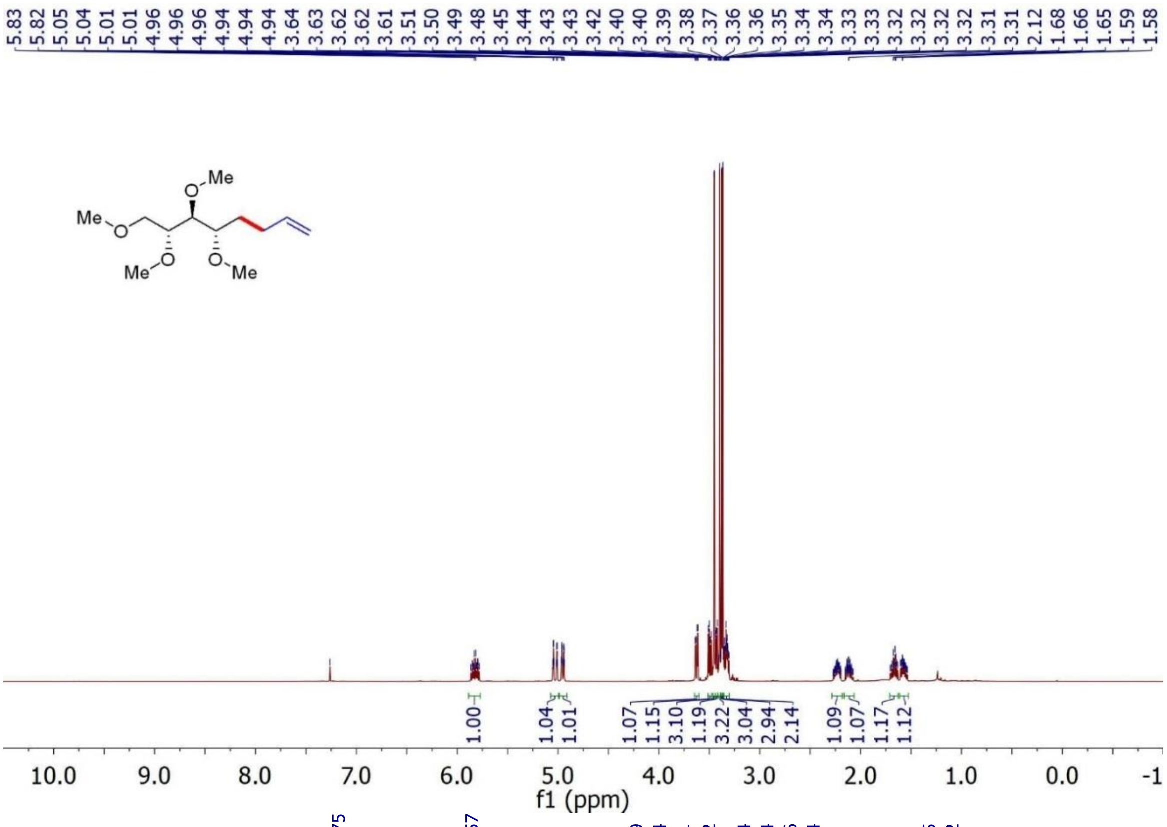

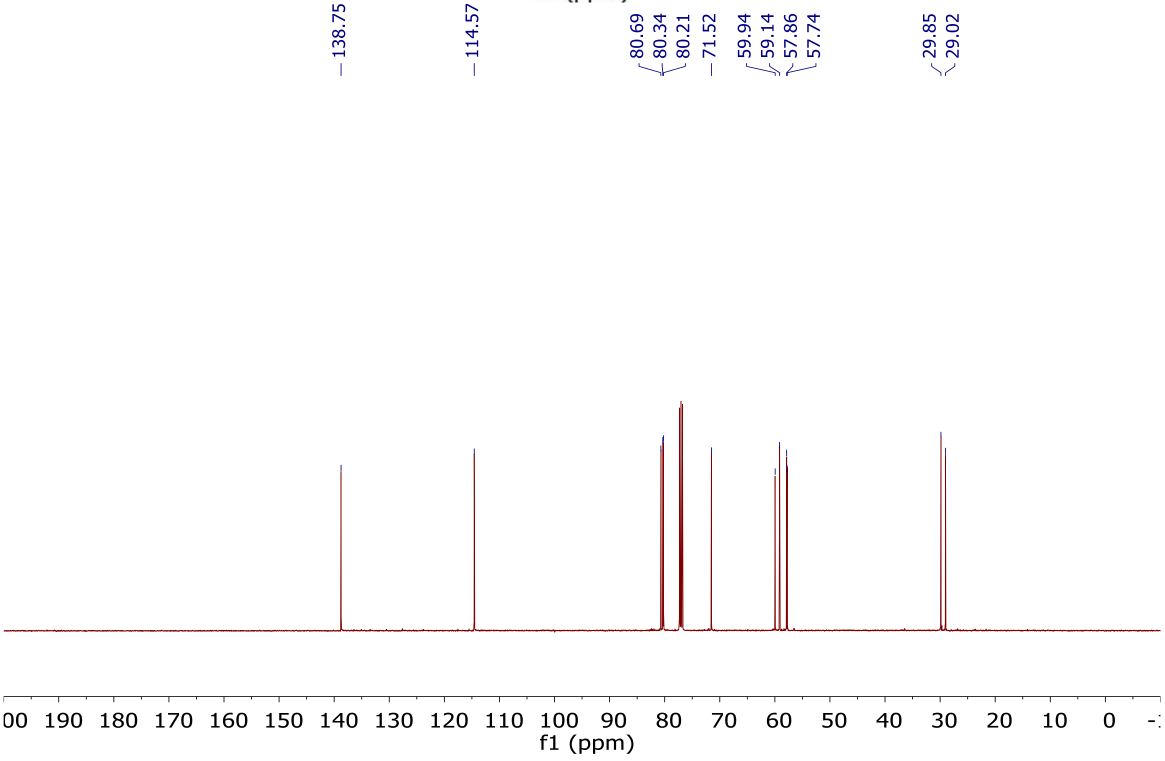

**(5*S*,6*R*,7*S*)-5,6,7,8-tetramethoxyoct-1-ene (3ab):**

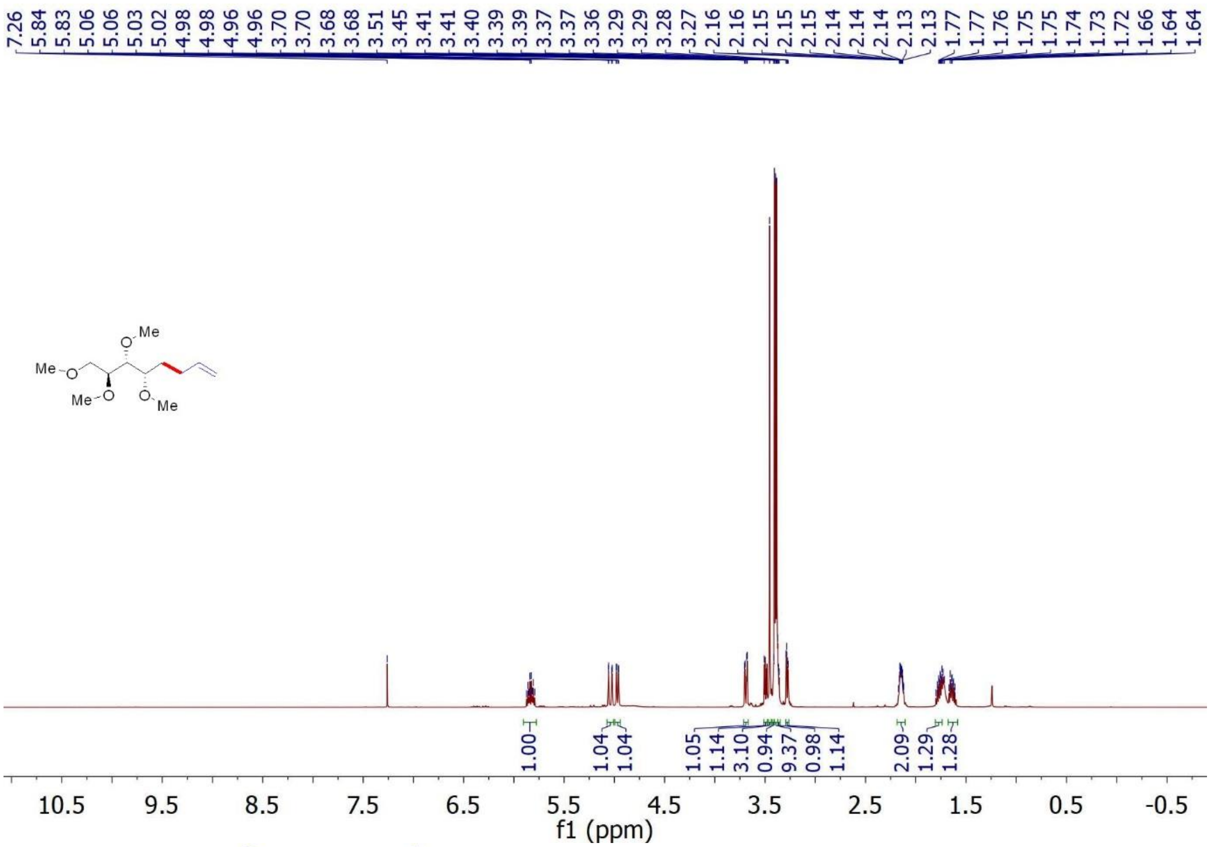


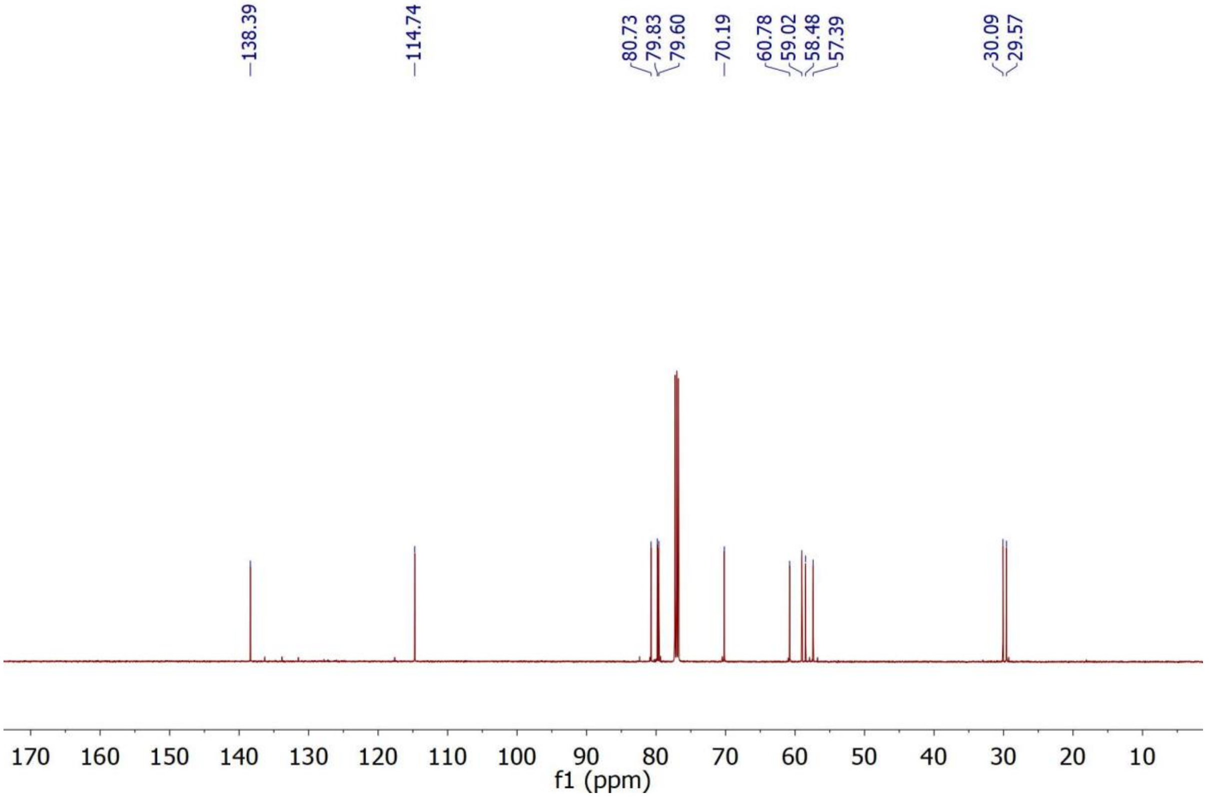


**(5*S*,6*R*,7*R*)-5,6,7,8-tetramethoxyoct-1-ene (3ac):**

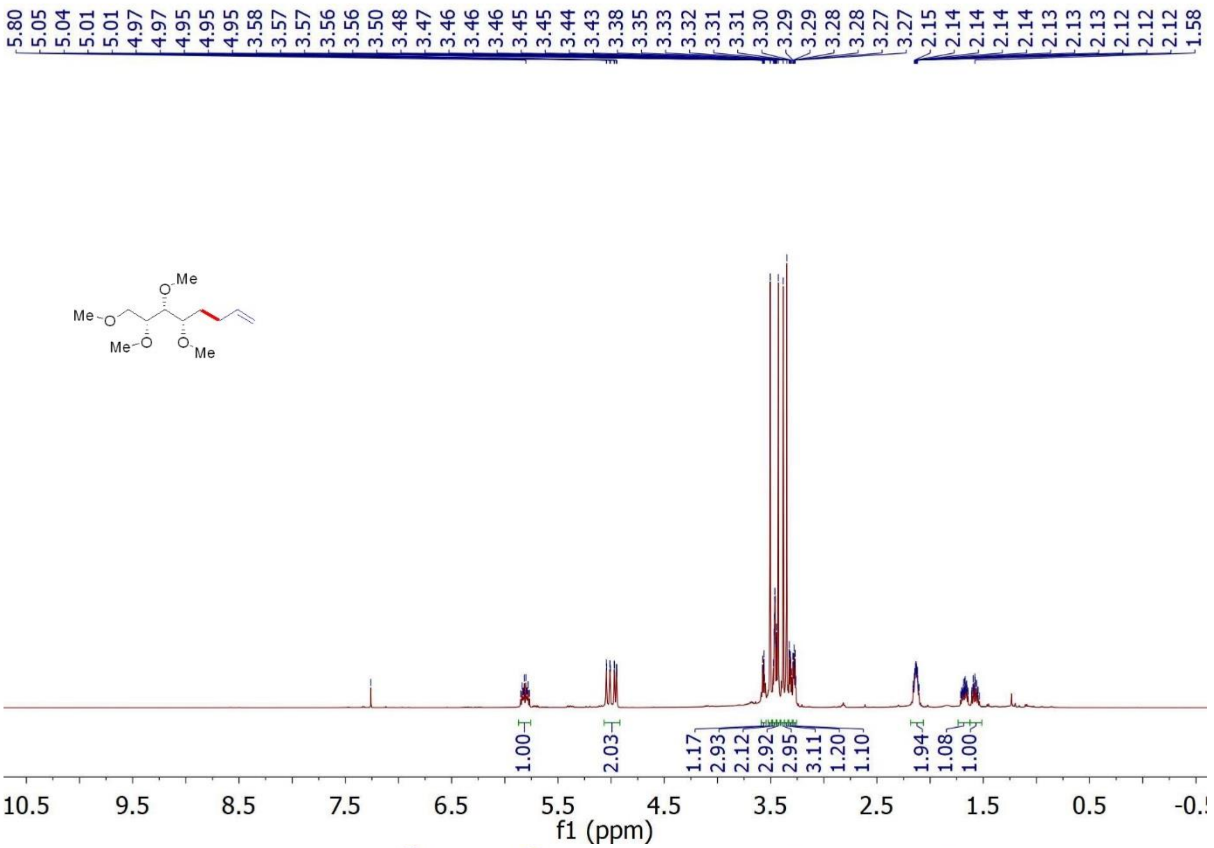


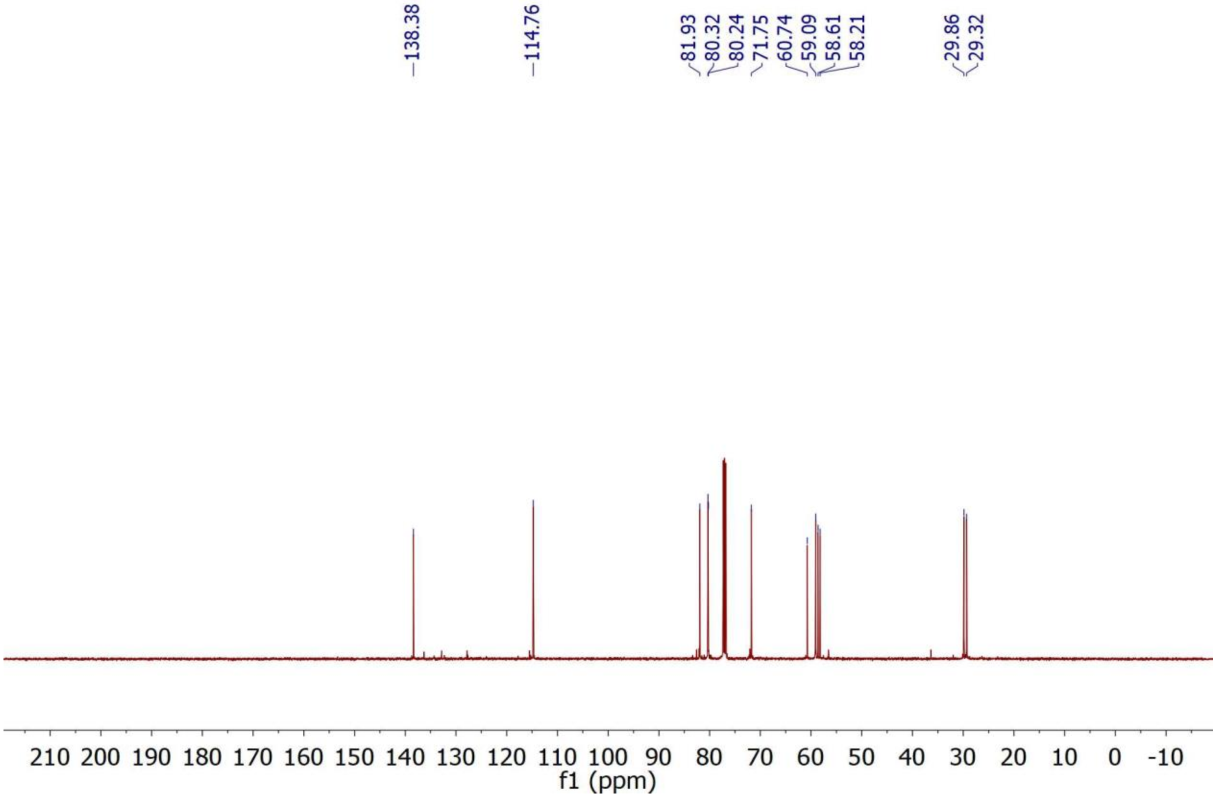


**(5*S*,6*R*,7*S*,8*R*)-5,6,7,8,9-pentamethoxynon-1-ene (3ad):**

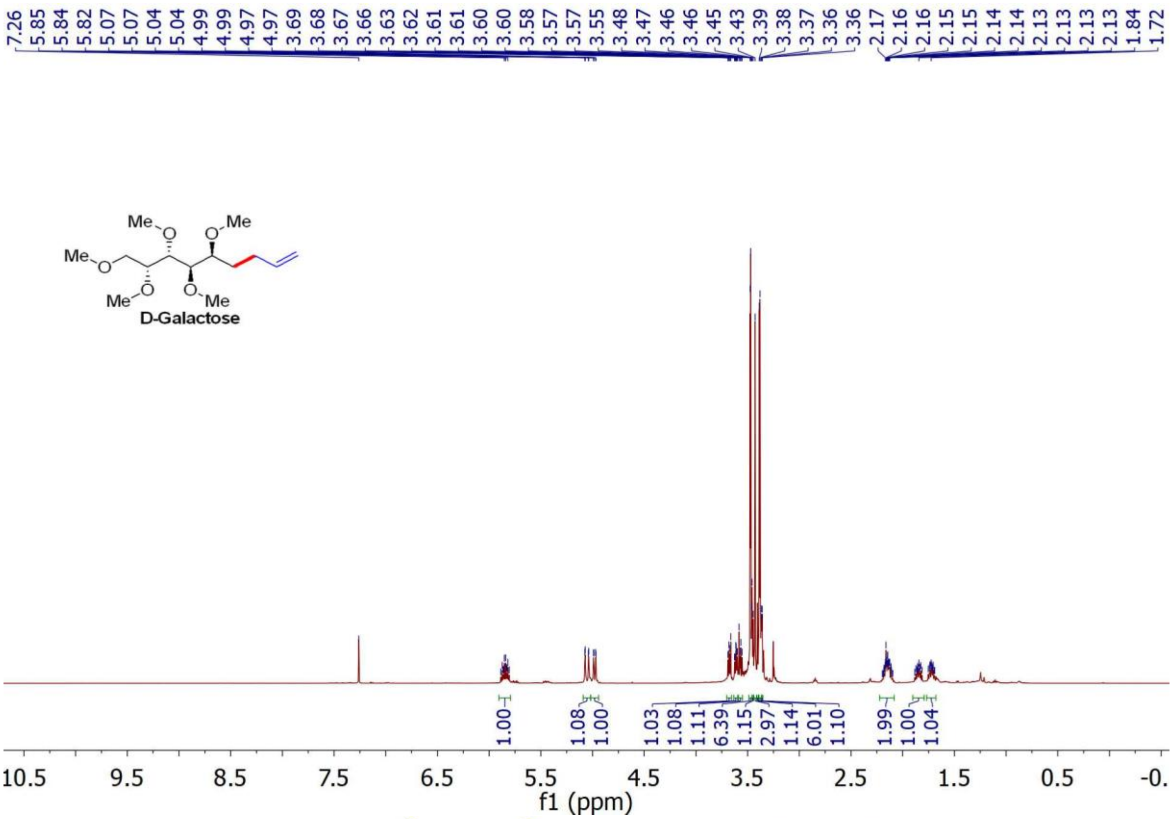


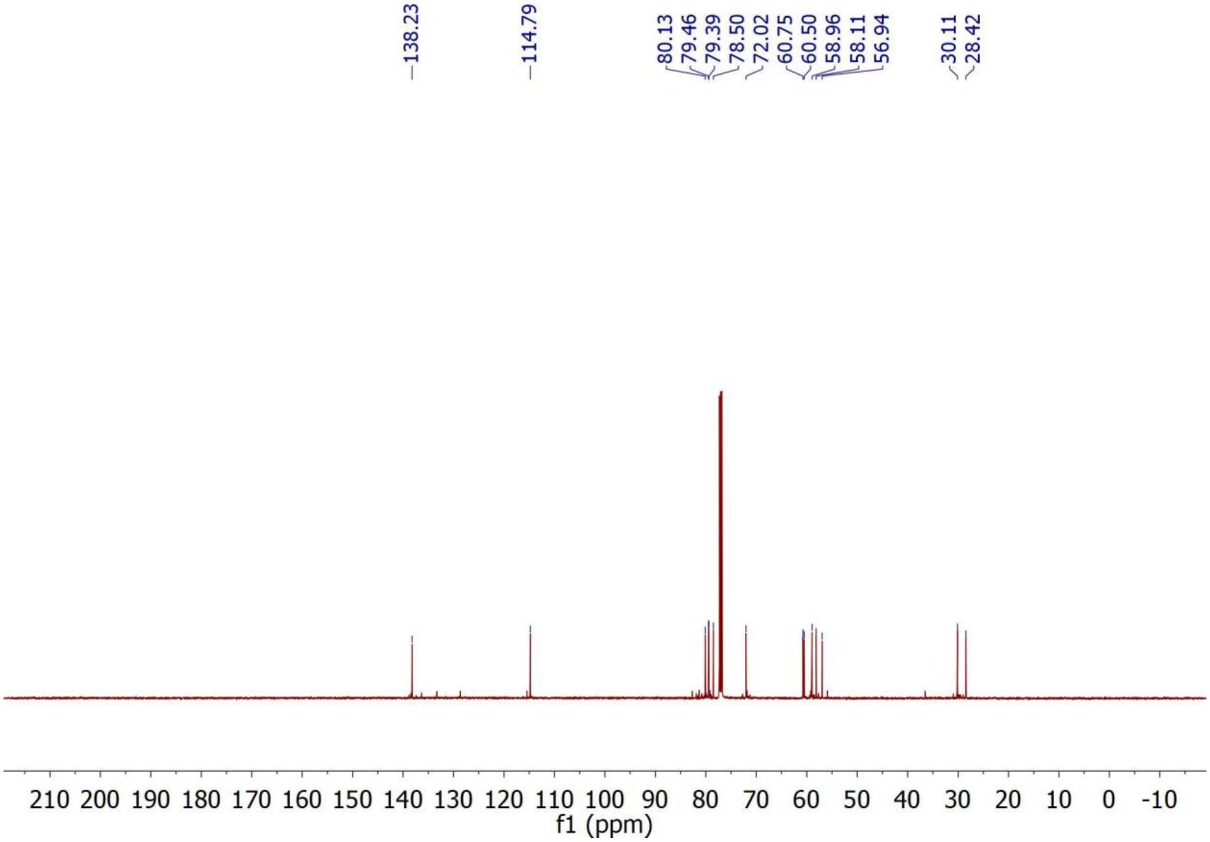


**(5*S*,6*R*,7*R*,8*R*)-5,6,7,8,9-pentamethoxynon-1-ene (3ae):**

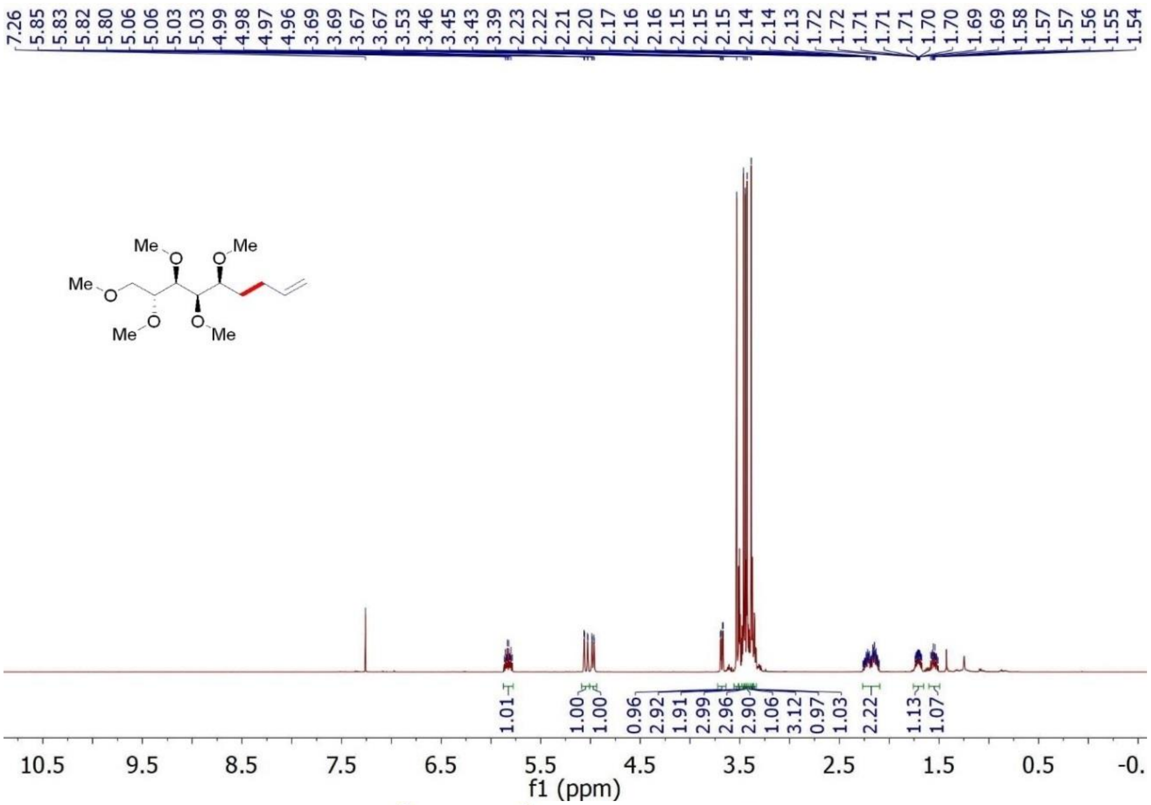

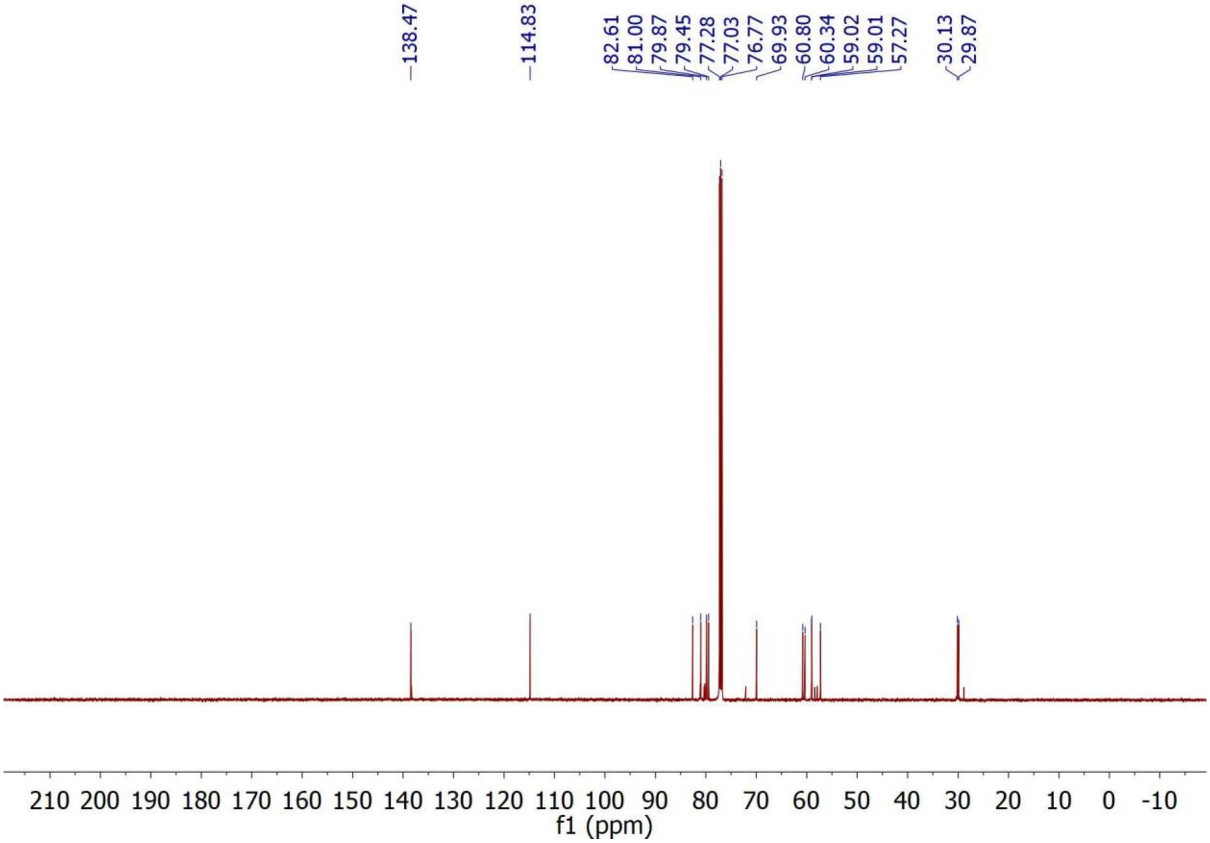

**(5*R*,6*S*,7*R*,8*S*)-5,6,7,8-tetramethoxynon-1-ene (3af):**

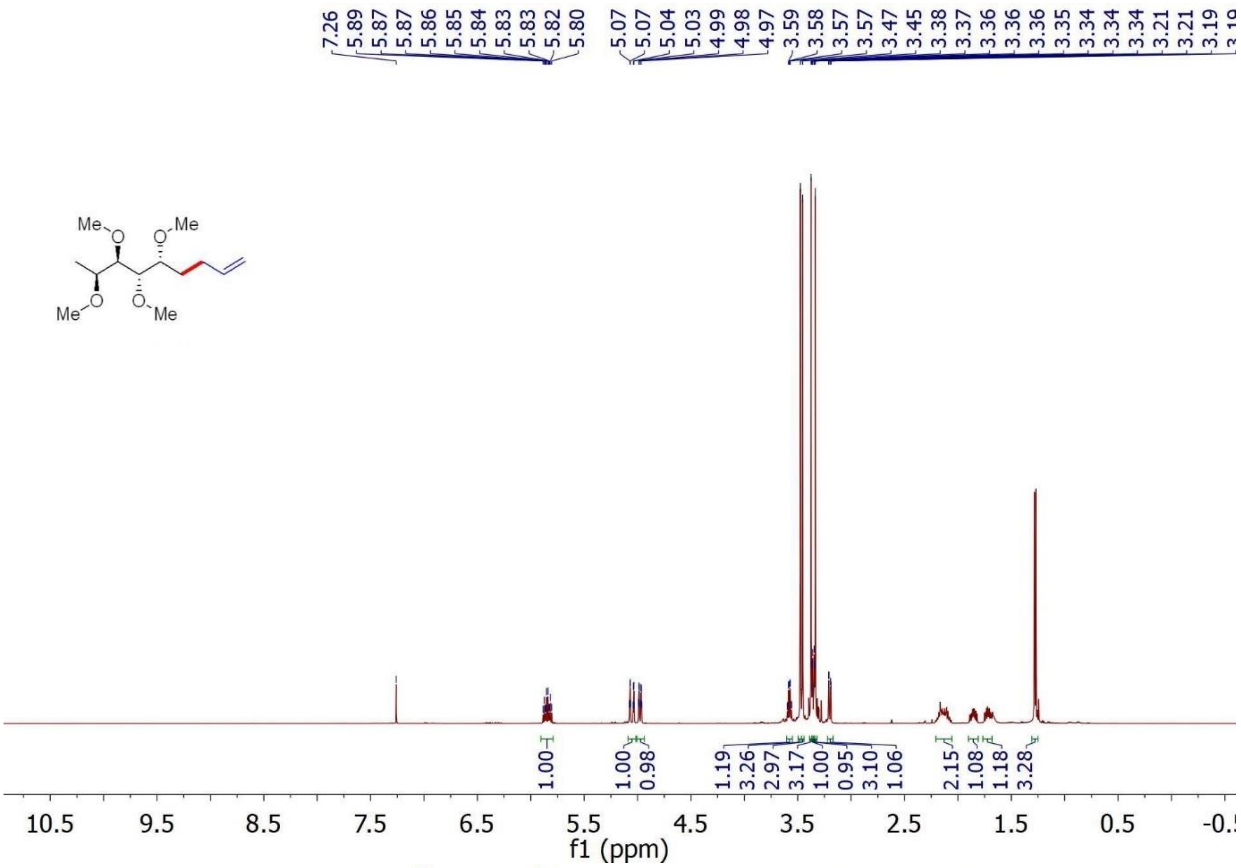


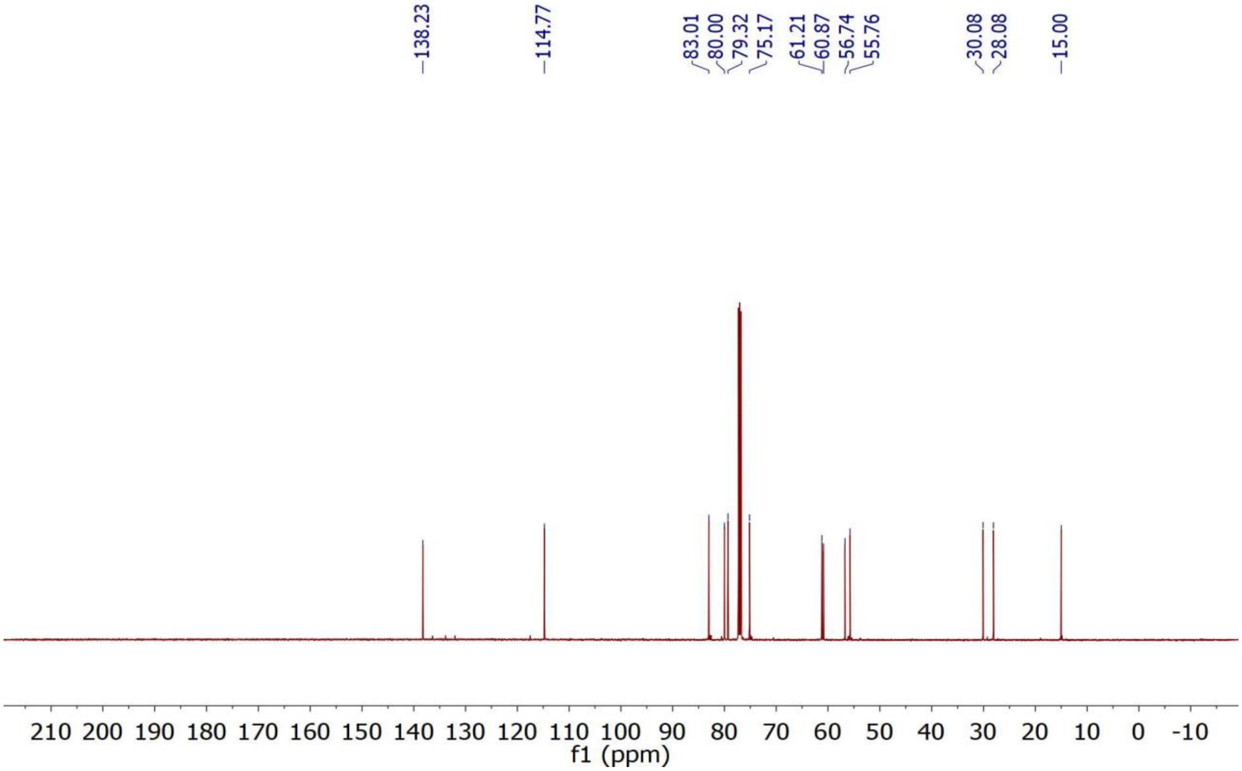


**(4*S*,4'*R*,5*R*)-5-(but-3-en-1-yl)-2,2,2',2'-tetramethyl-4,4'-bi(1,3-dioxolane) (3ag):**

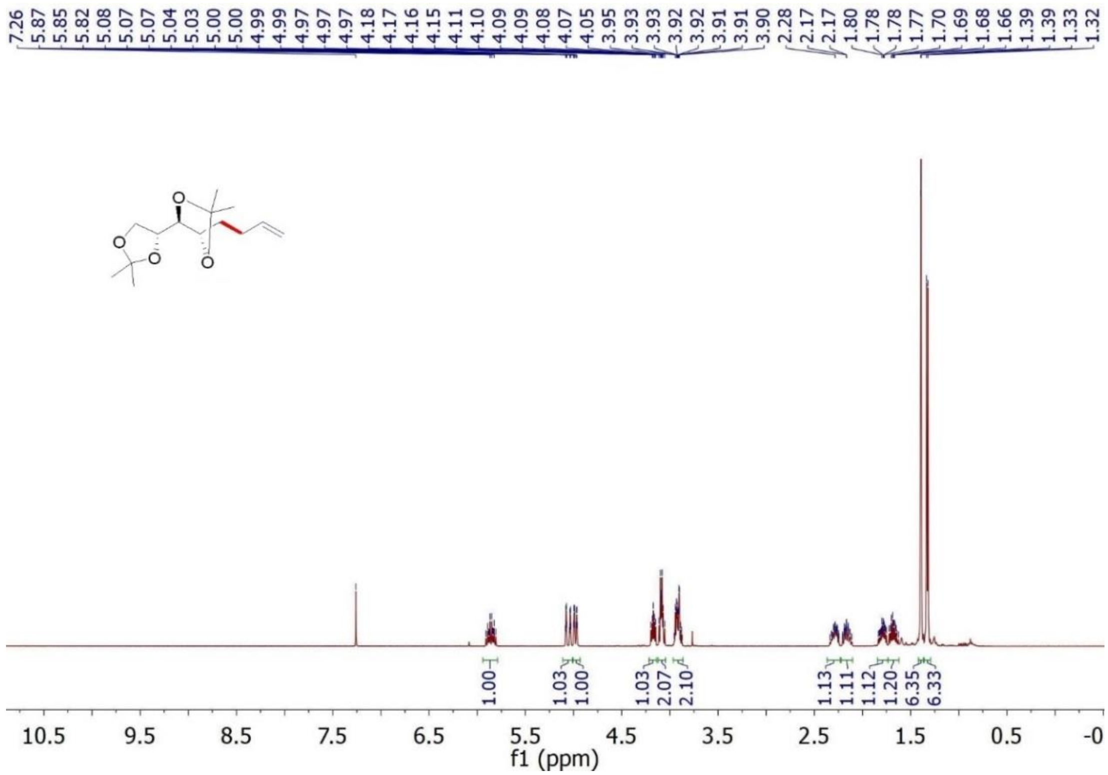


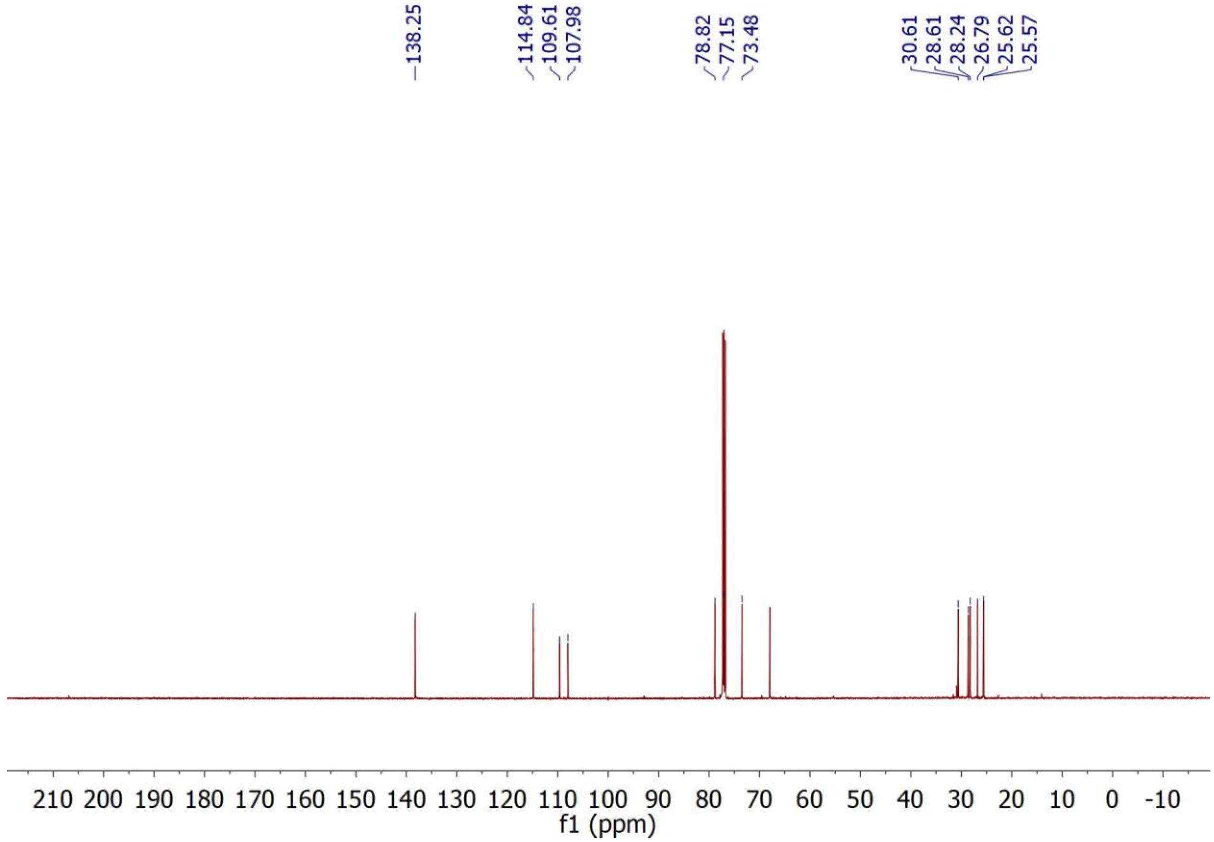


**(4*R*,4'*S*,5*S*)-5-(but-3-en-1-yl)-2,2,2',2'-tetramethyl-4,4'-bi(1,3-dioxolane) (3ah):**

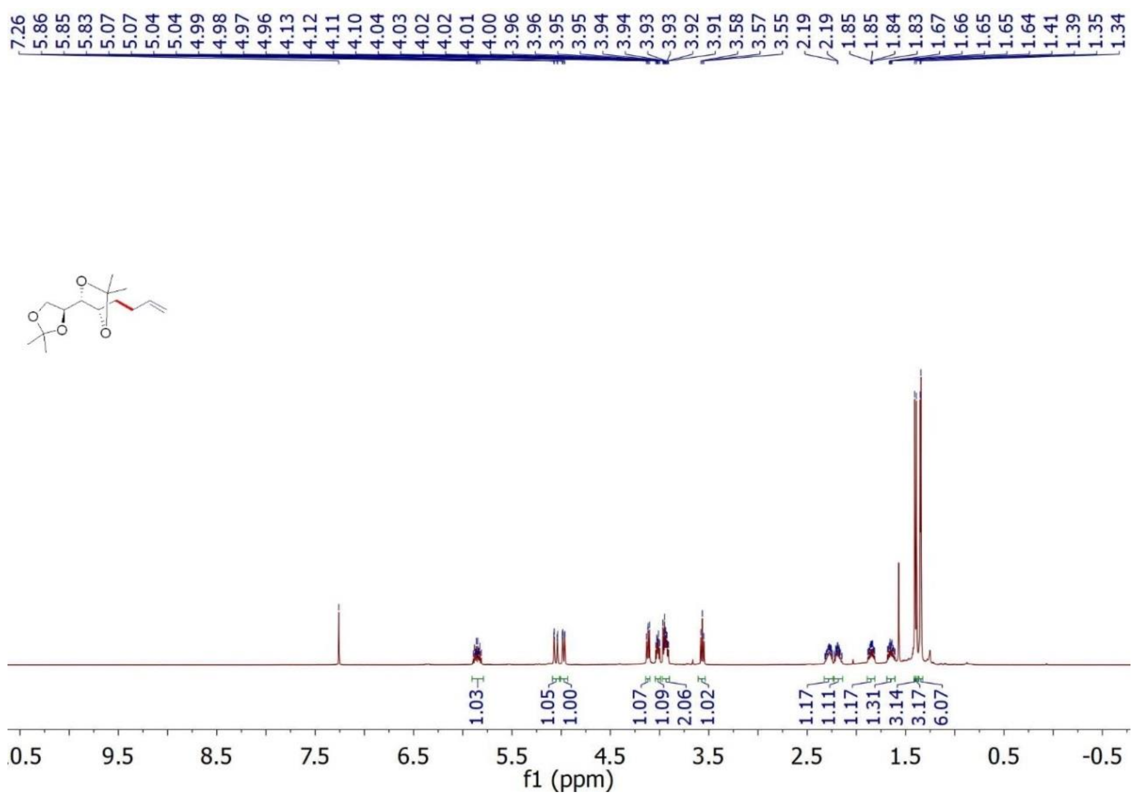
7.26 5.86 5.85 5.83 5.07 5.07 5.04 4.99 4.98 4.97 4.96 4.13 4.12 4.11 4.10 4.04 4.03 4.02 4.02 4.01 4.00 3.96 3.96 3.95 3.95 3.94 3.94 3.93 3.93 3.92 3.91 3.58 3.57 3.55 2.19 2.19 1.85 1.85 1.84 1.83 1.67 1.66 1.65 1.65 1.64 1.41 1.39 1.35 1.34
1.03 1.05 1.00 1.07 1.09 2.06 1.02 1.17 1.11 1.17 1.31 3.14 3.17 6.07
.0.5 9.5 8.5 7.5 6.5 5.5 4.5 3.5 2.5 1.5 0.5 -0.5
f1 (ppm)

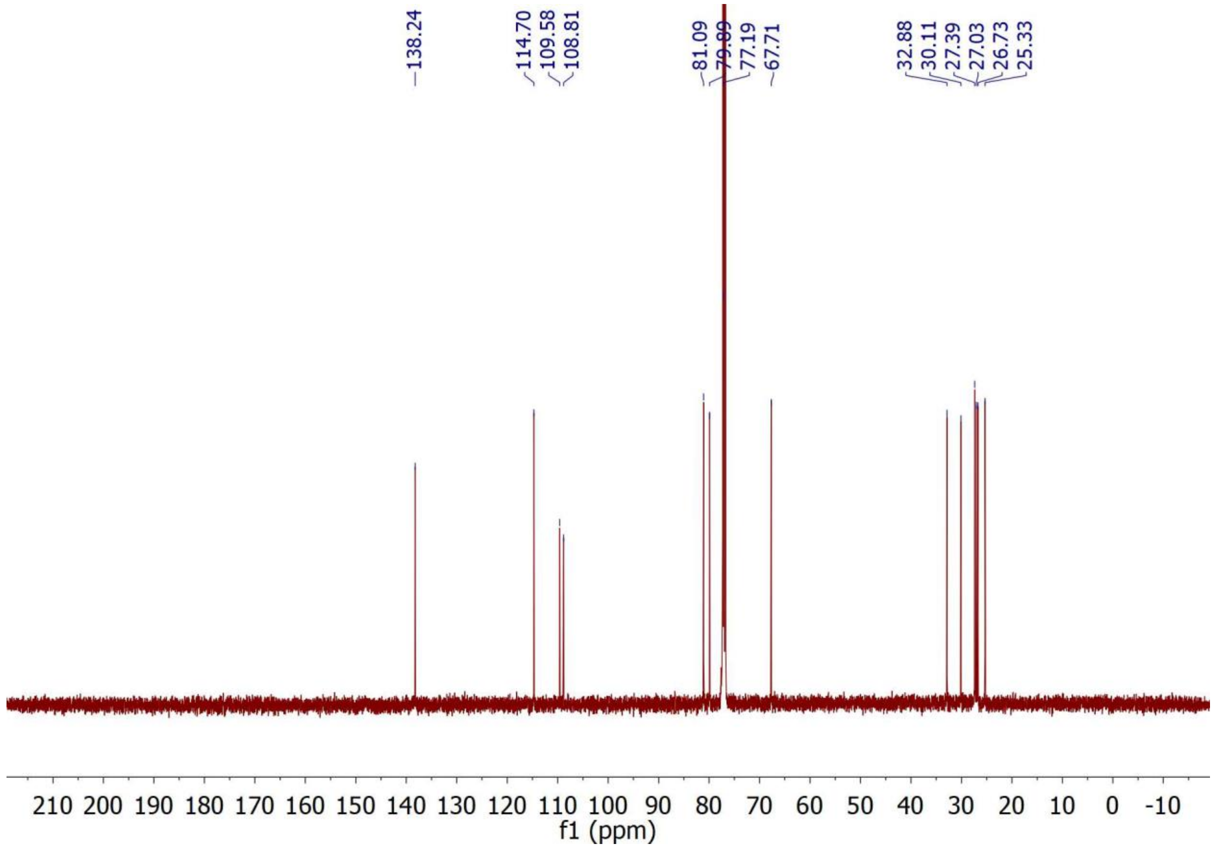
138.24 114.70 109.58 108.81 81.09 79.89 77.19 67.71 32.88 30.11 27.39 27.03 26.73 25.33
210 200 190 180 170 160 150 140 130 120 110 100 90 80 70 60 50 40 30 20 10 0 -10
f1 (ppm)

**(4*S*,5*R*)-4-(but-3-en-1-yl)-5-((*S*)-((*R*)-2,2-dimethyl-1,3-dioxolan-4-yl)(methoxy)methyl)-2,2-dimethyl-1,3- dioxolane (3ai):**

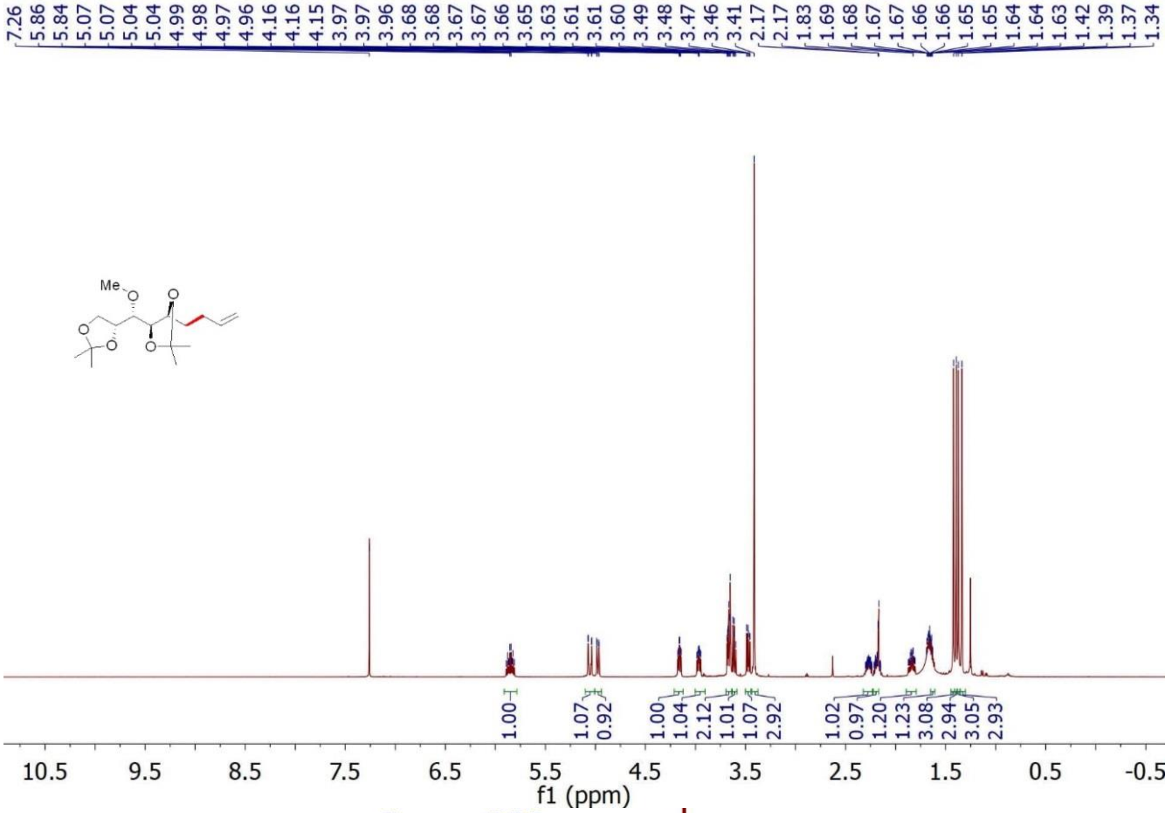


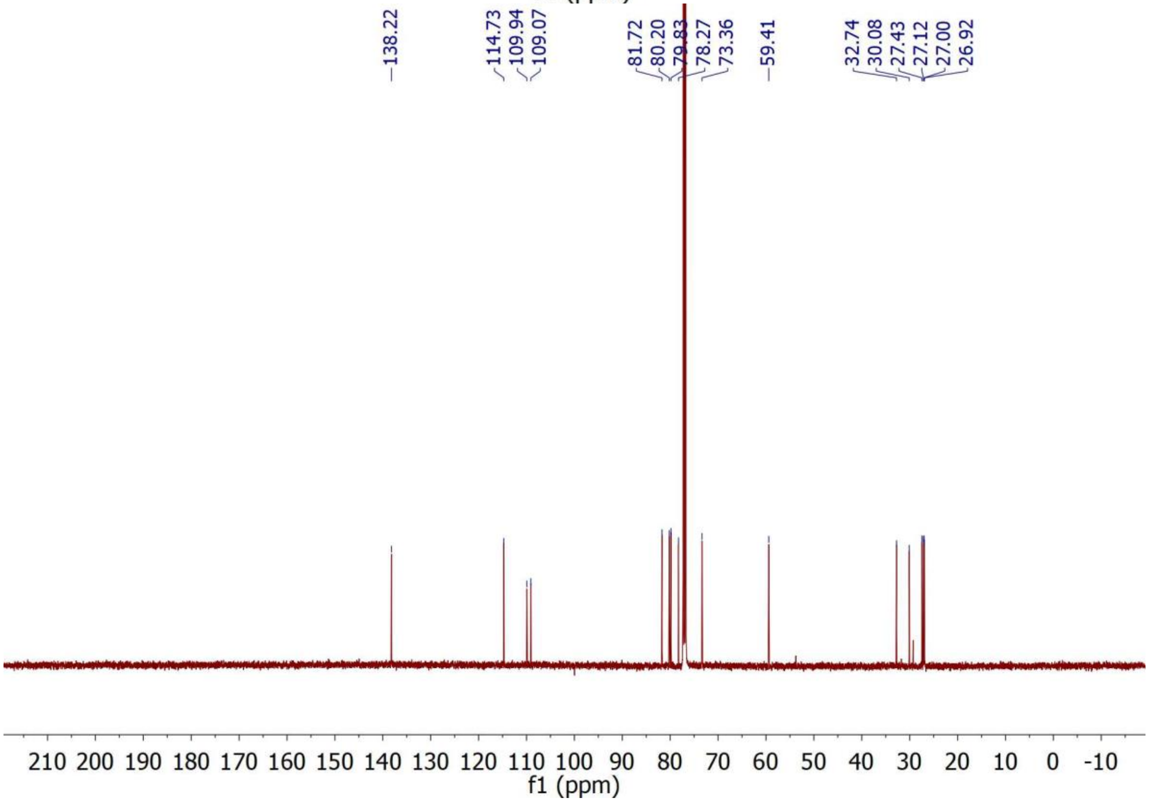

**(3a*R*,5*R*,5a*S*,8a*S*,8b*R*)-5-(but-3-en-1-yl)-2,2,7,7-tetramethyltetrahydro-5H-bis([1,3]dioxolo)[4,5-b:4',5'-d]pyran (3aj):**

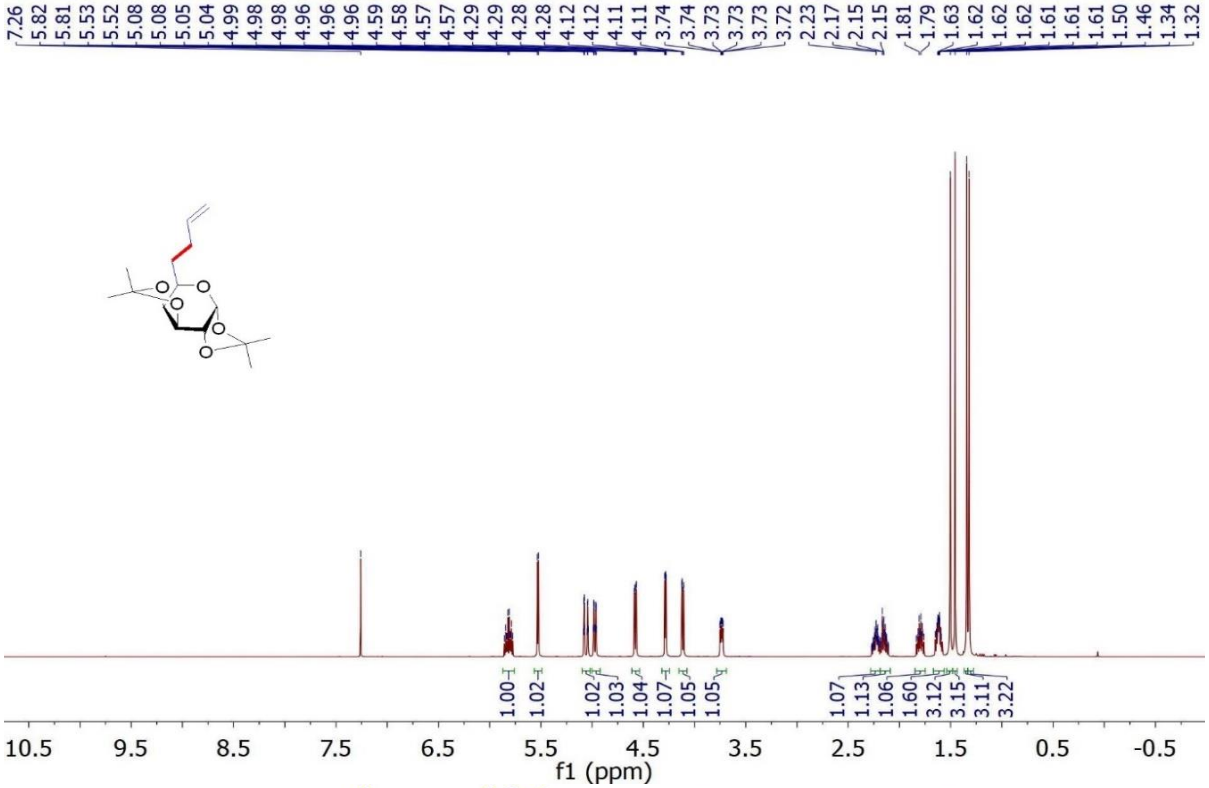


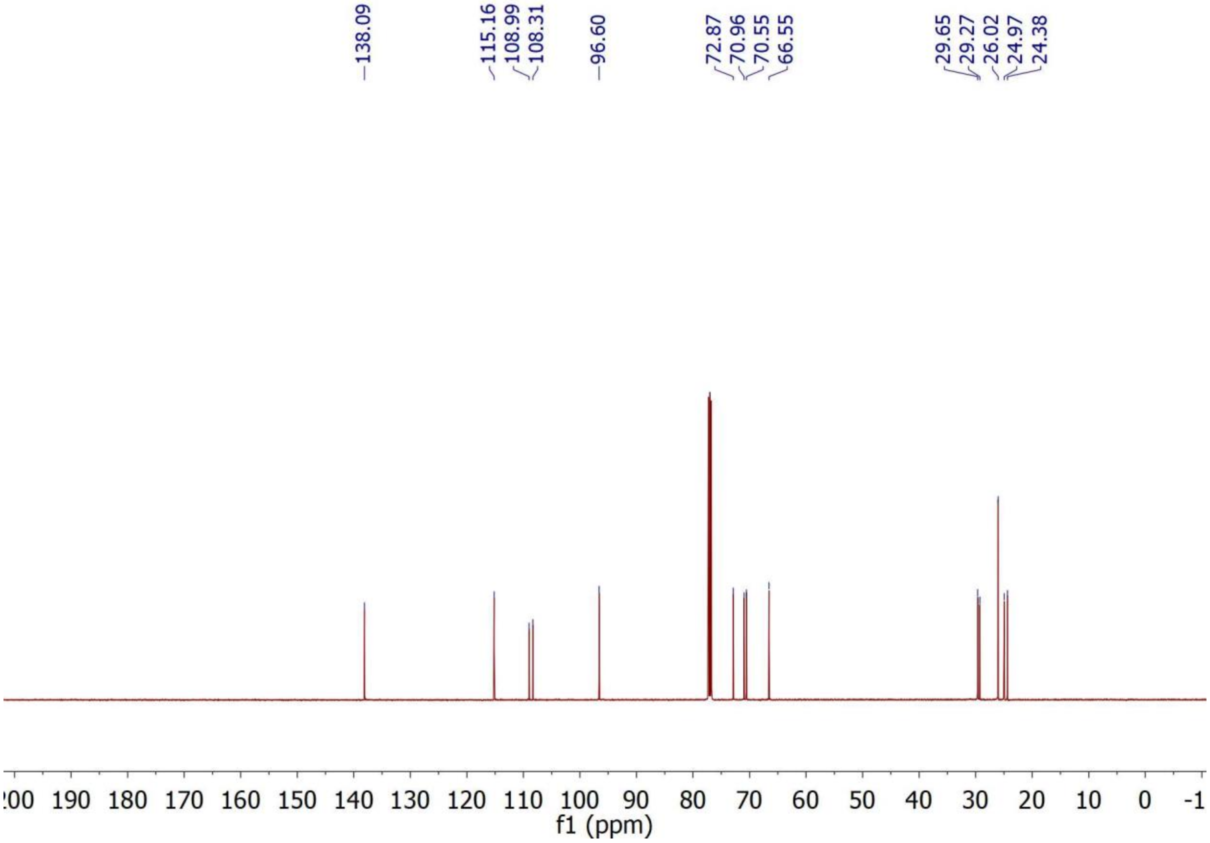

**(3a*S*,5a*R*,8a*R*,8b*S*)-3a-(but-3-en-1-yl)-2,2,7,7-tetramethyltetrahydro-5H-bis([1,3]dioxolo)[4,5-b:4',5'-d]pyran (3ak):**

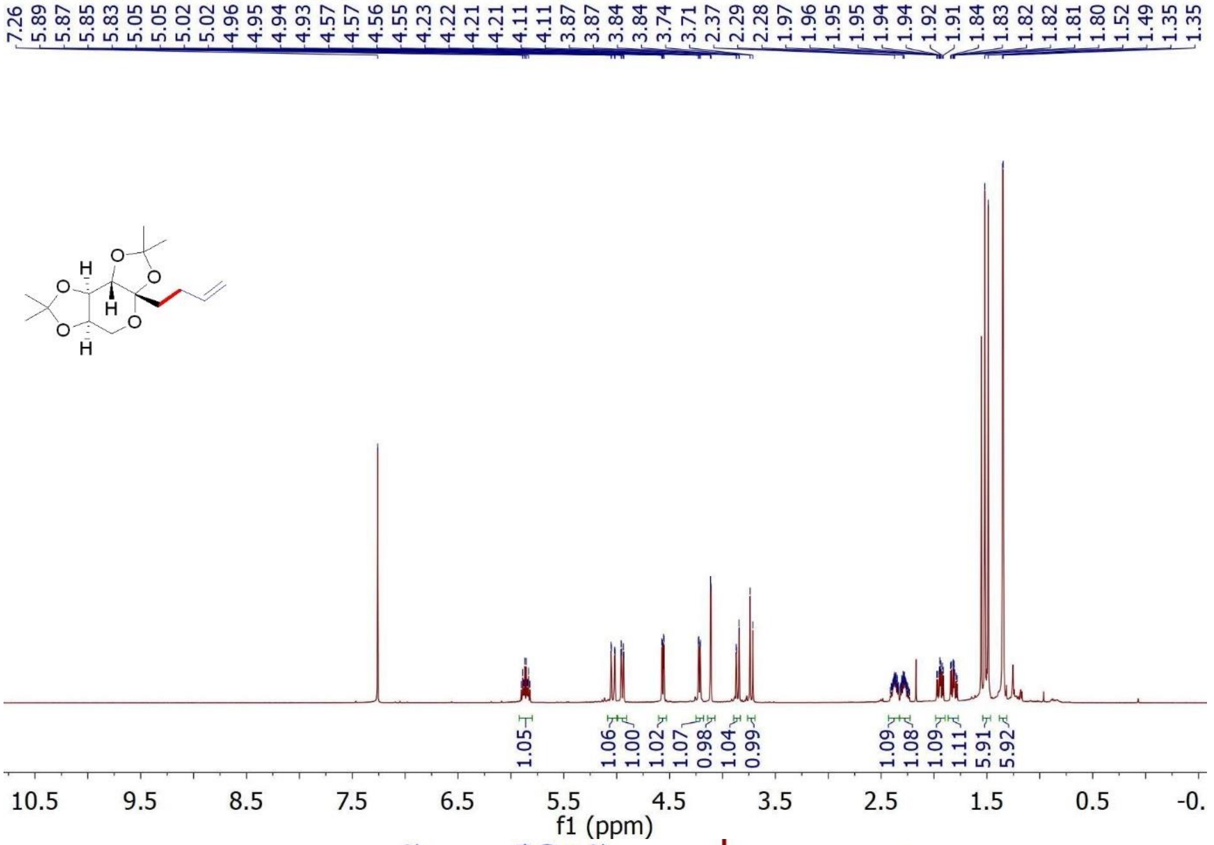

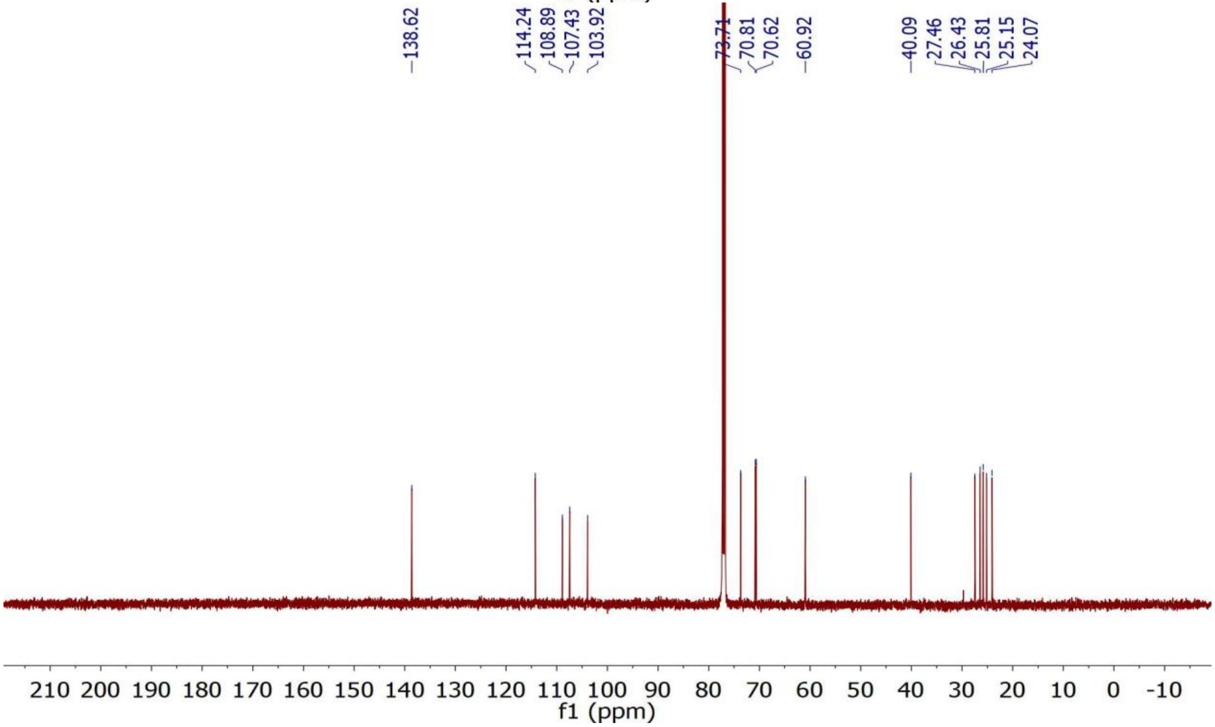

**(3a*R*,4*R*,6*R*,6a*R*)-4-(but-3-en-1-yl)-6-methoxy-2,2-dimethyltetrahydrofuro[3,4-d][1,3]dioxole (3al):**

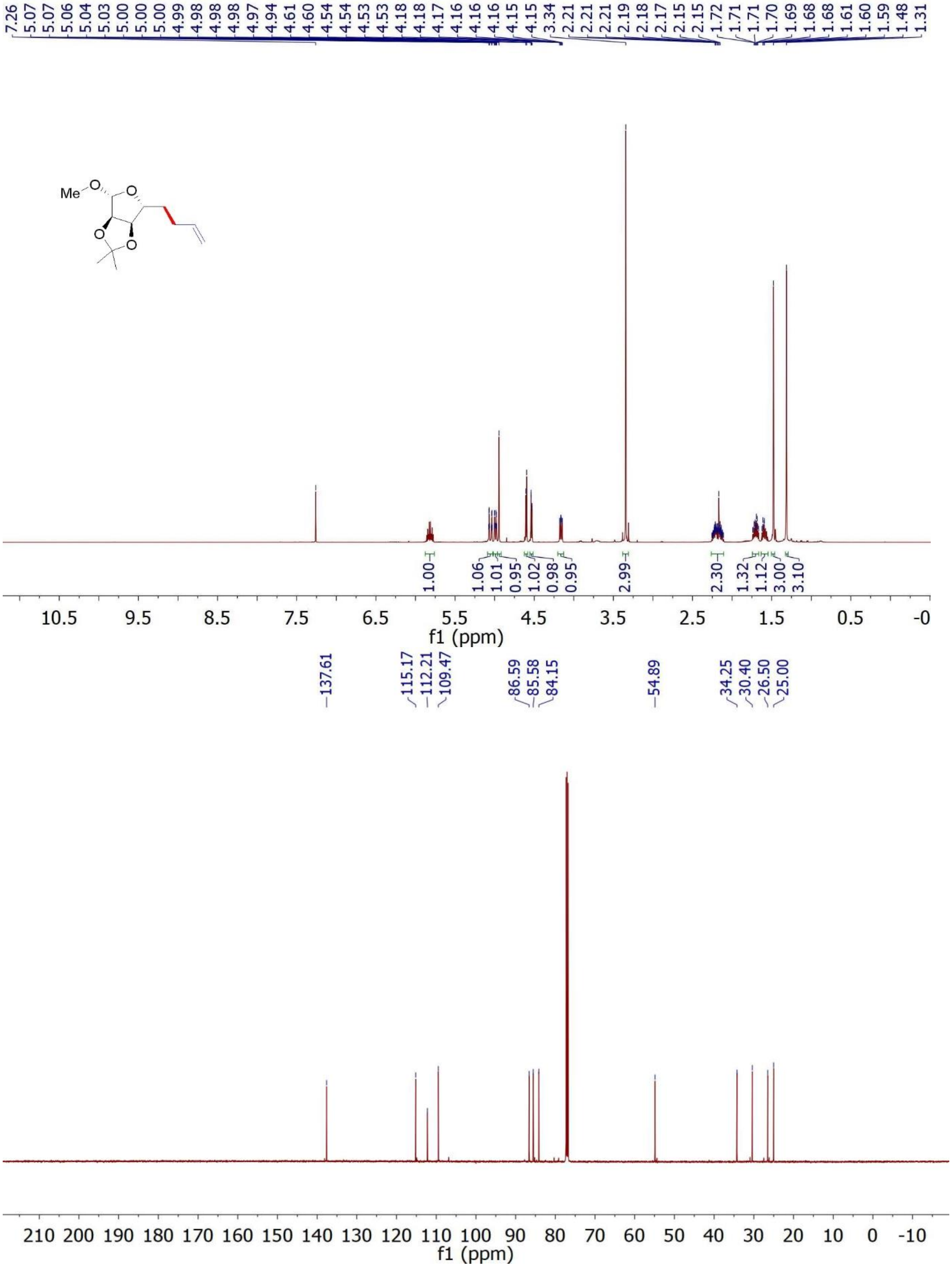

**(2*S*,3*R*,4*S*,5*R*)-non-8-ene-2,3,4,5-tetraol (3am):**

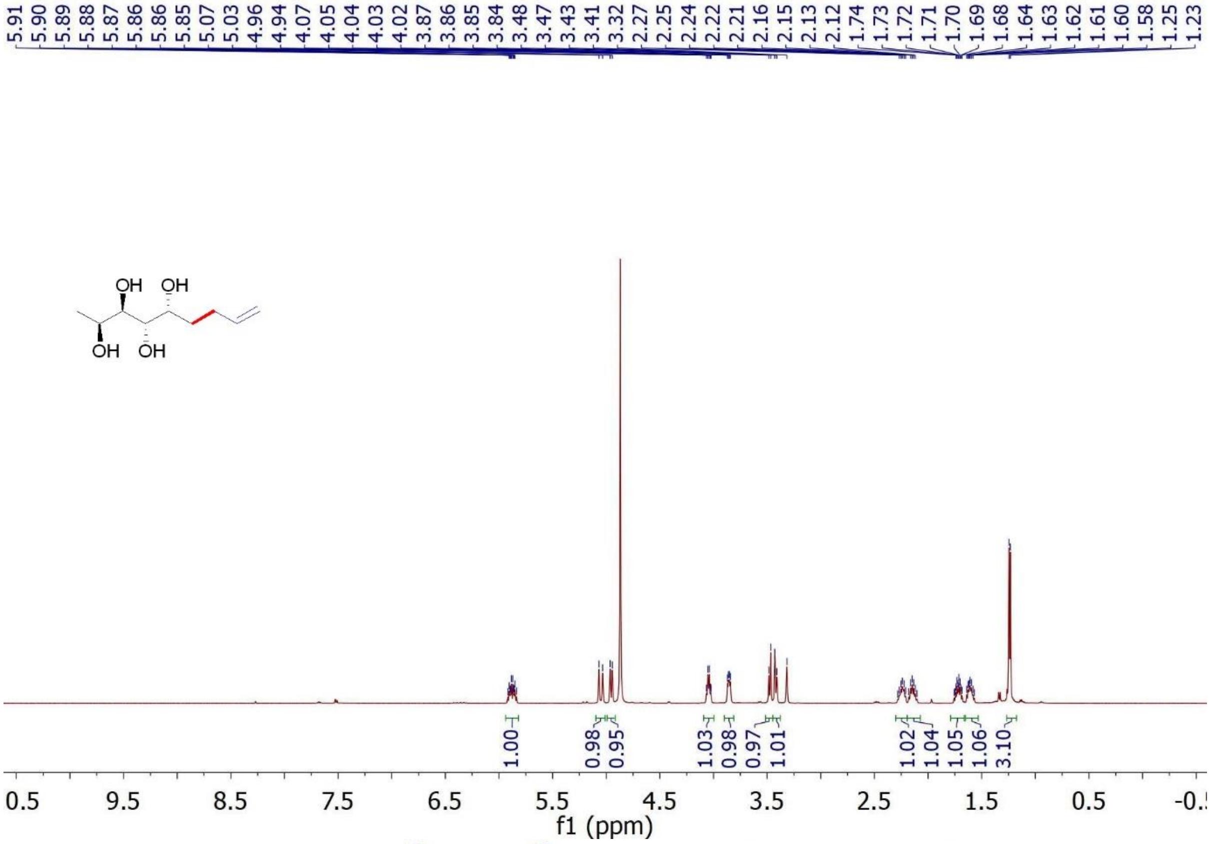


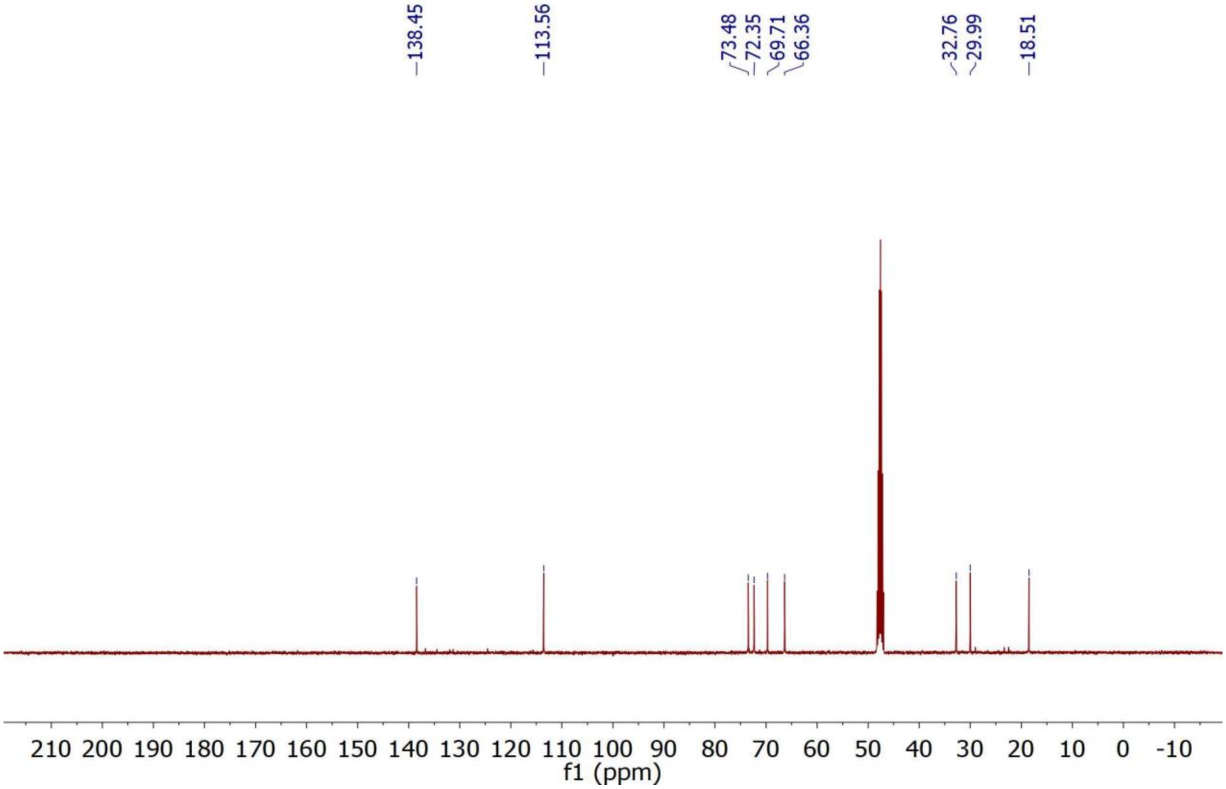


**(2*R*,3*S*,4*R*)-oct-7-ene-1,2,3,4-tetraol (3an):**

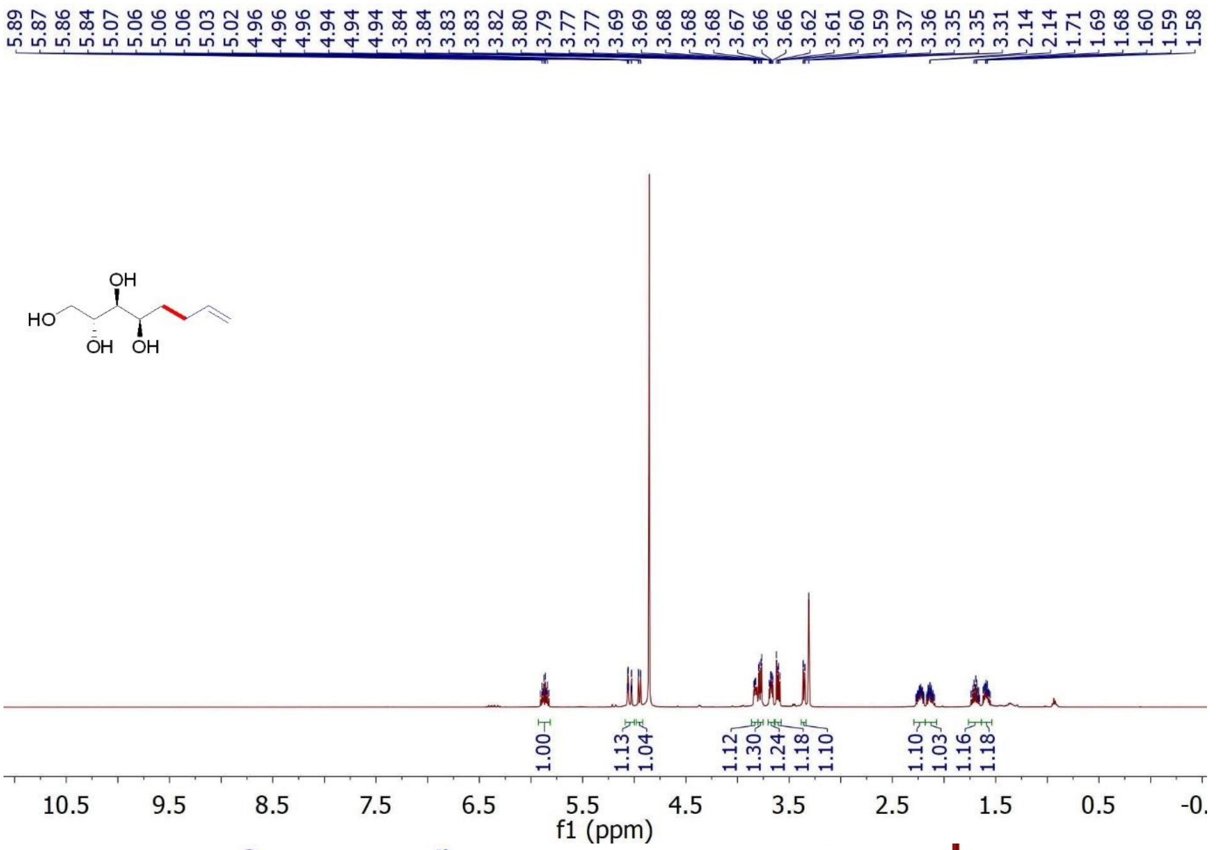

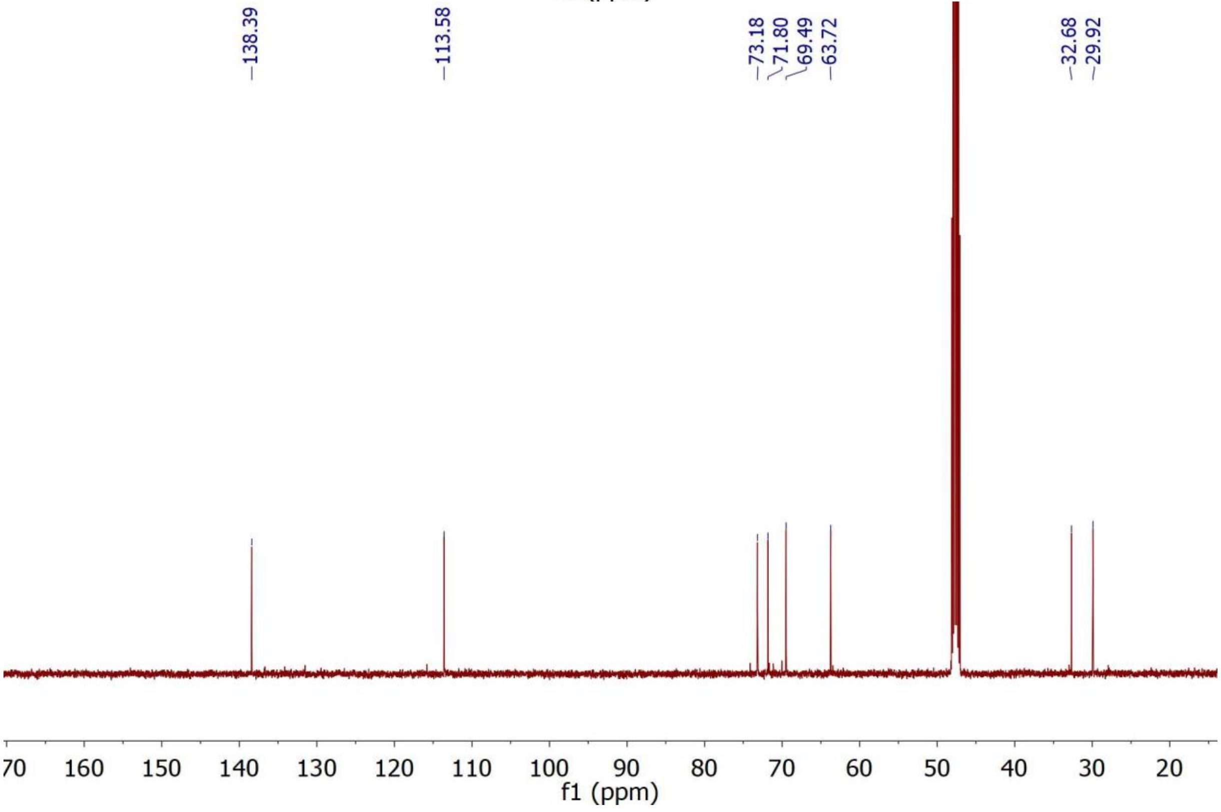


**(2*S*,3*R*,4*S*)-oct-7-ene-1,2,3,4-tetraol (3ao):**

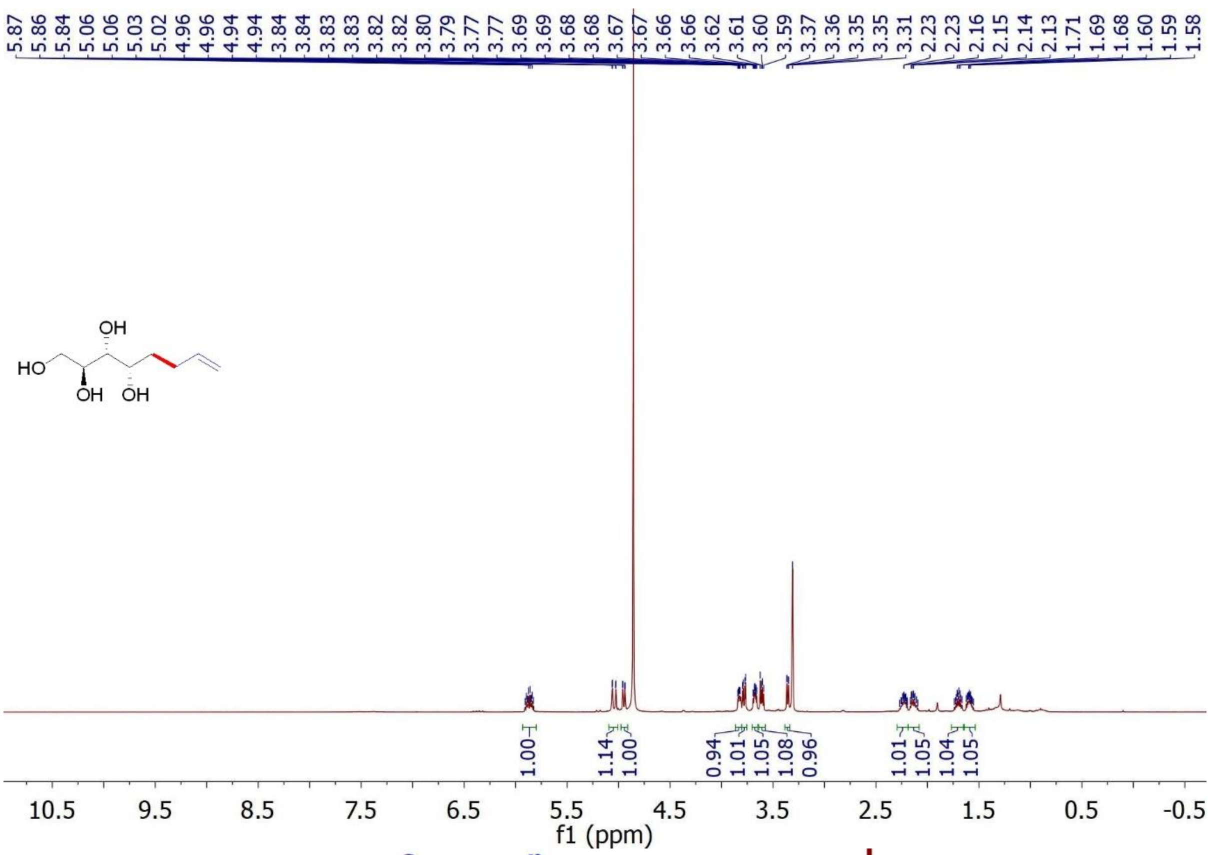
5.87 5.86 5.84 5.06 5.06 5.03 5.02 4.96 4.94 4.94 3.84 3.84 3.83 3.83 3.82 3.82 3.80 3.79 3.77 3.77 3.69 3.69 3.68 3.68 3.67 3.67 3.66 3.66 3.62 3.61 3.60 3.59 3.37 3.36 3.35 3.35 3.31 2.23 2.23 2.16 2.15 2.14 2.13 1.71 1.69 1.68 1.60 1.59 1.58
OH
HO
OH OH
1.00
1.14
1.00
0.94
1.01
1.05
1.08
0.96
1.01
1.05
1.04
1.05
10.5 9.5 8.5 7.5 6.5 5.5 4.5 3.5 2.5 1.5 0.5 -0.5
f1 (ppm)
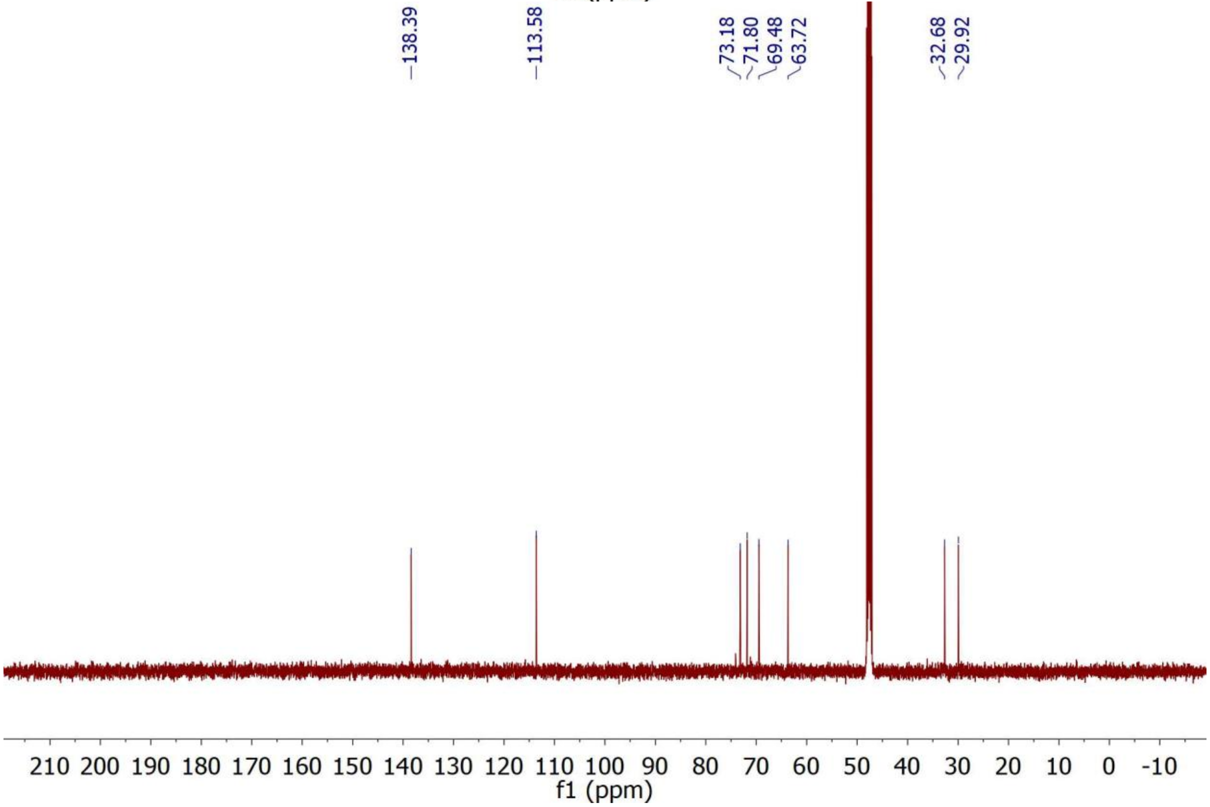
138.39
113.58
73.18
71.80
69.48
63.72
32.68
29.92
210 200 190 180 170 160 150 140 130 120 110 100 90 80 70 60 50 40 30 20 10 0 -10
f1 (ppm)

**(2*R*,3*S*, *E*)-1,3-bis(benzyloxy)octa-4,7-dien-2-ol (3as):**

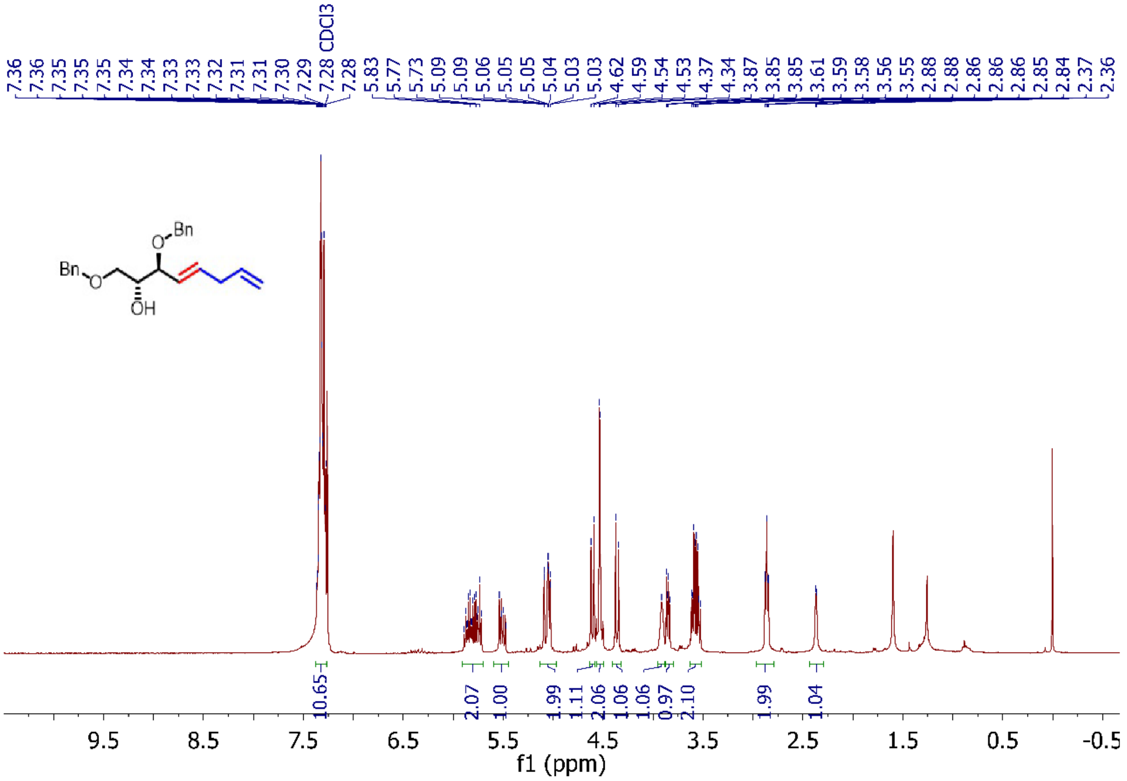


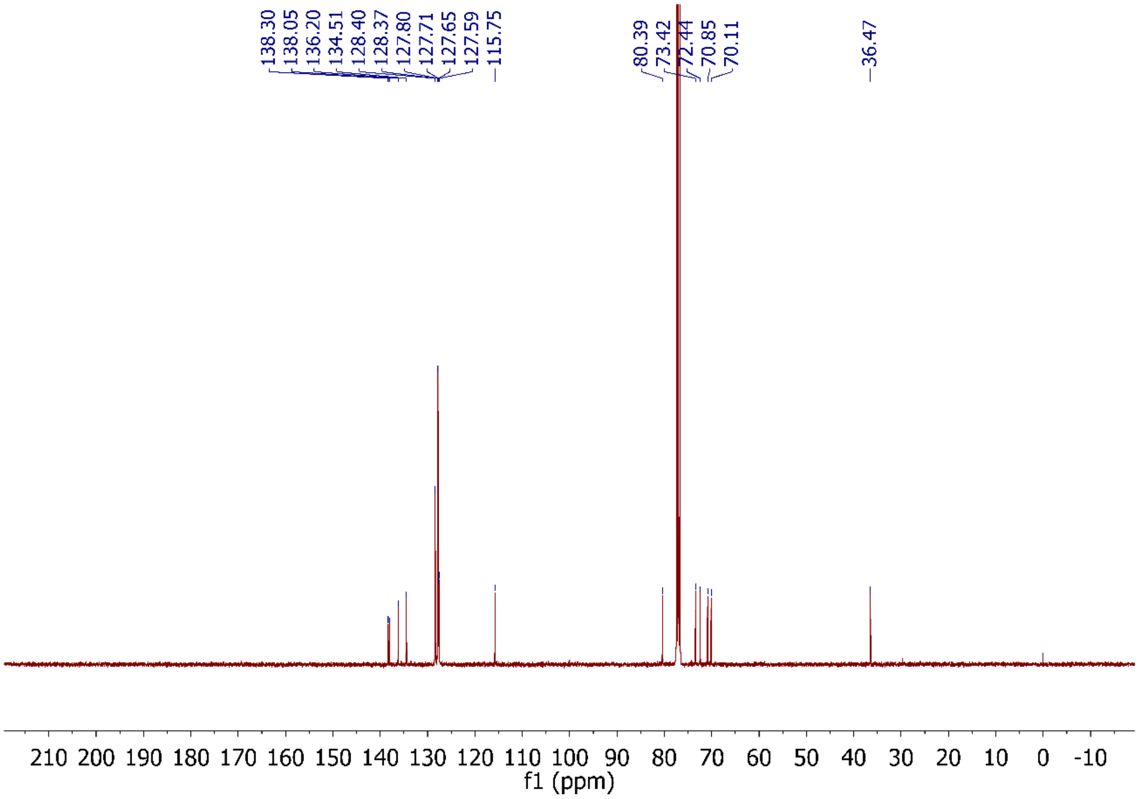

**(5*S*,6*S*,7*R*, *E*)-5,6,7,8-tetramethoxyocta-1,3-diene (4aa):**

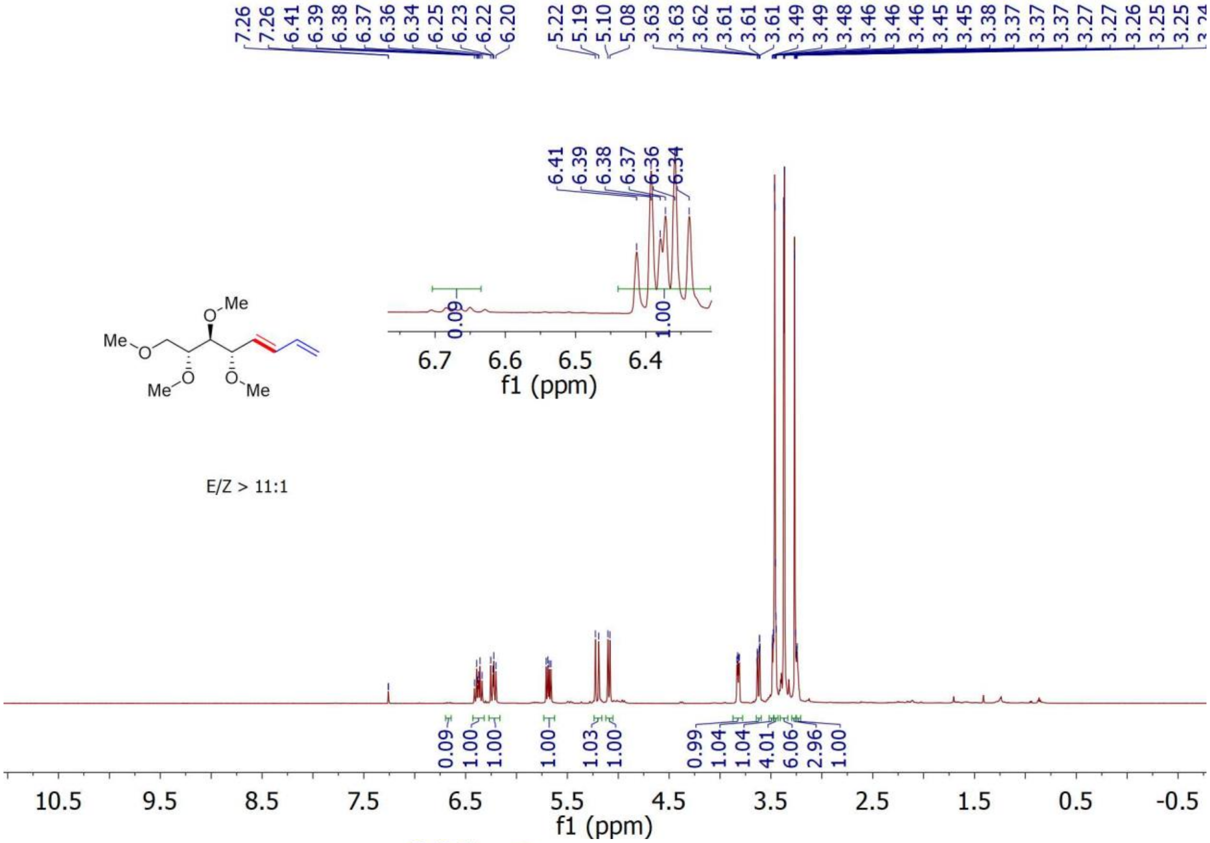


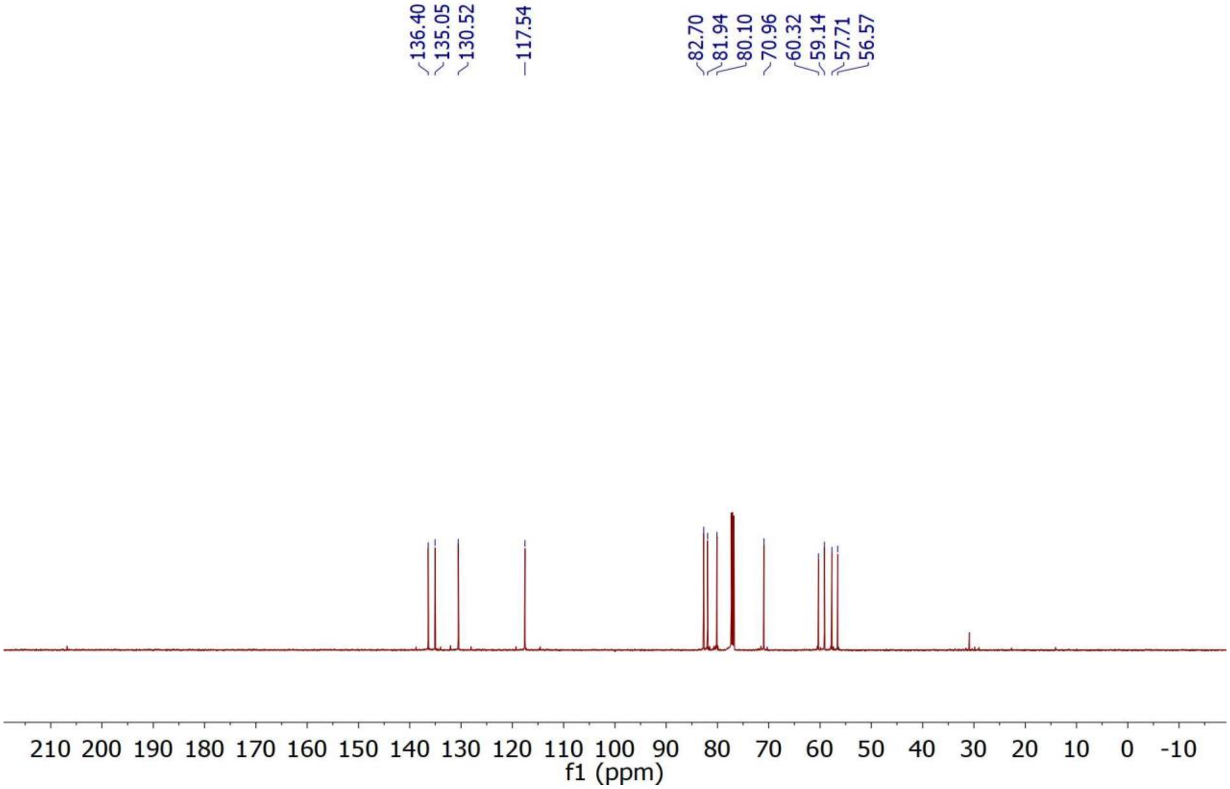

**(5*S*,6*R*,7*S*, *E*)-5,6,7,8-tetramethoxyocta-1,3-diene (4ab):**

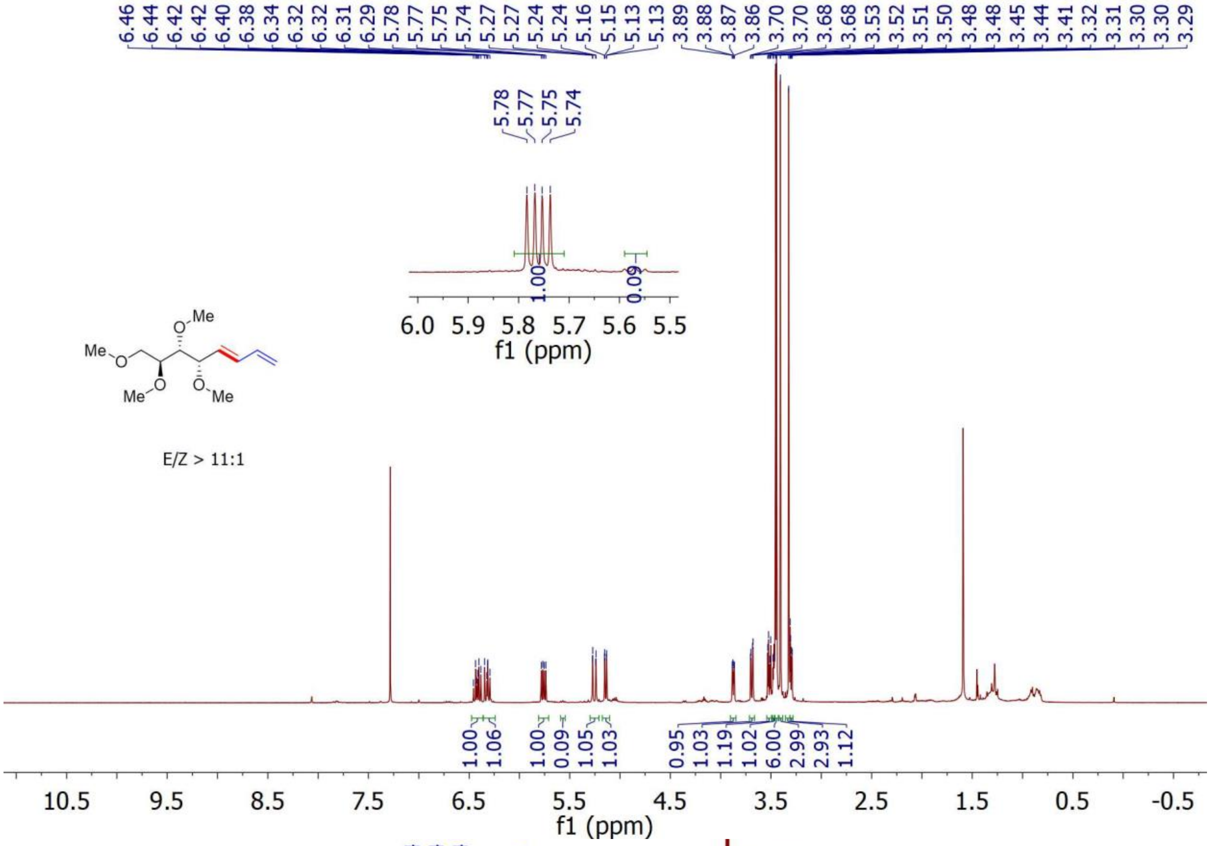


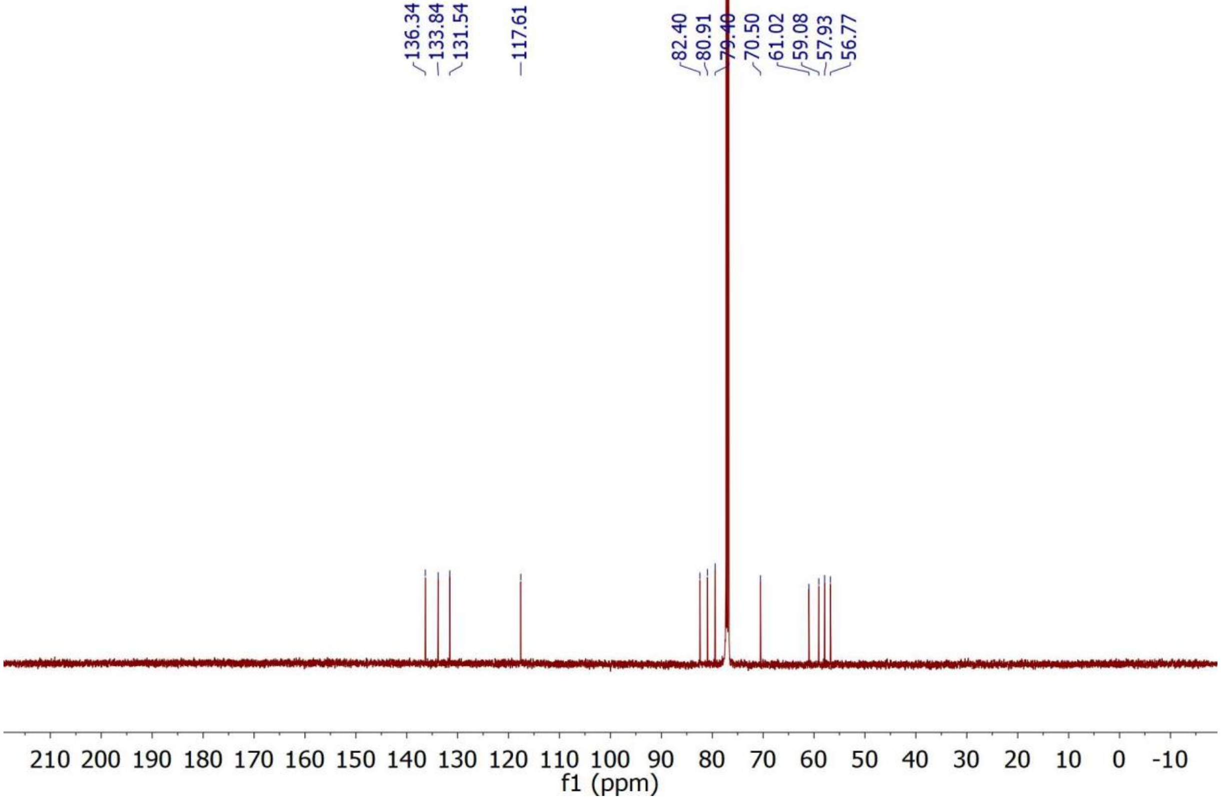


**(5R,6S,7R,8S, E)-5,6,7,8-tetramethoxynona-1,3-diene (4ac):**

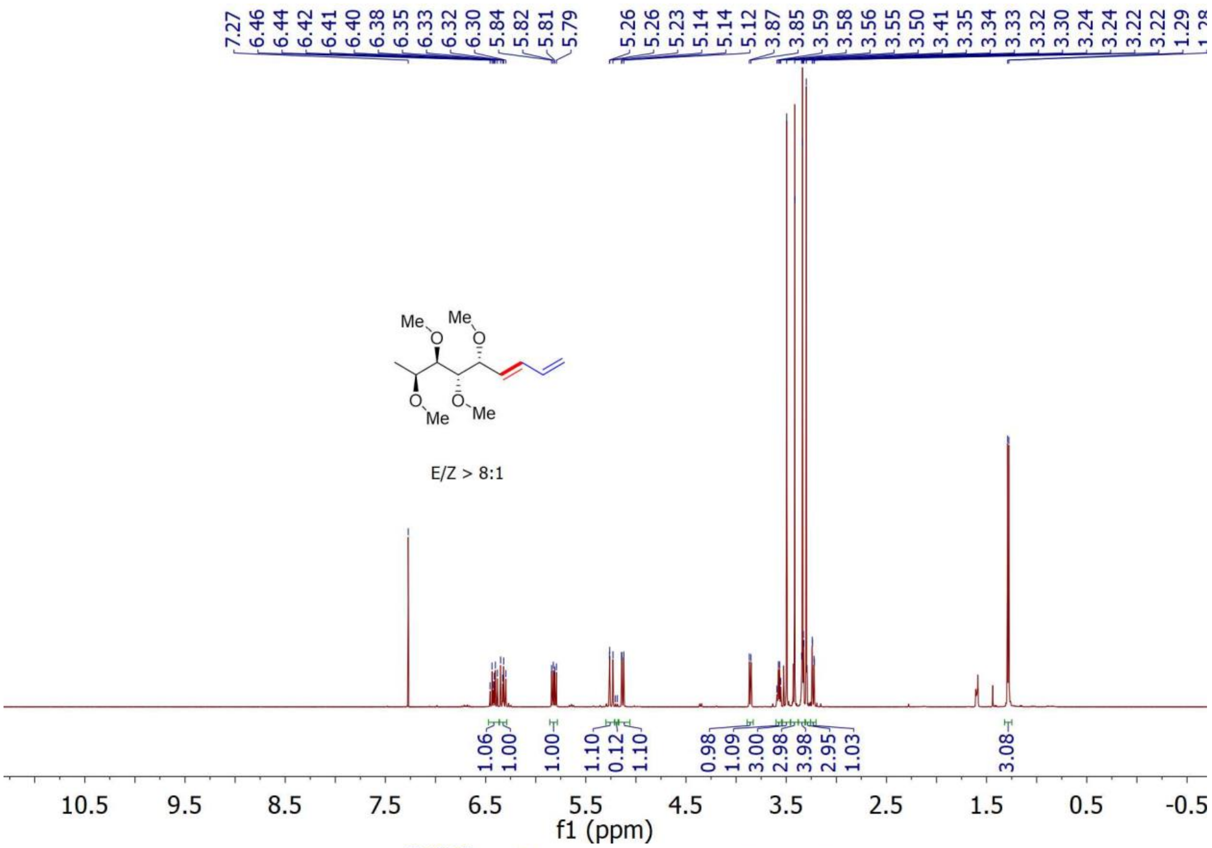


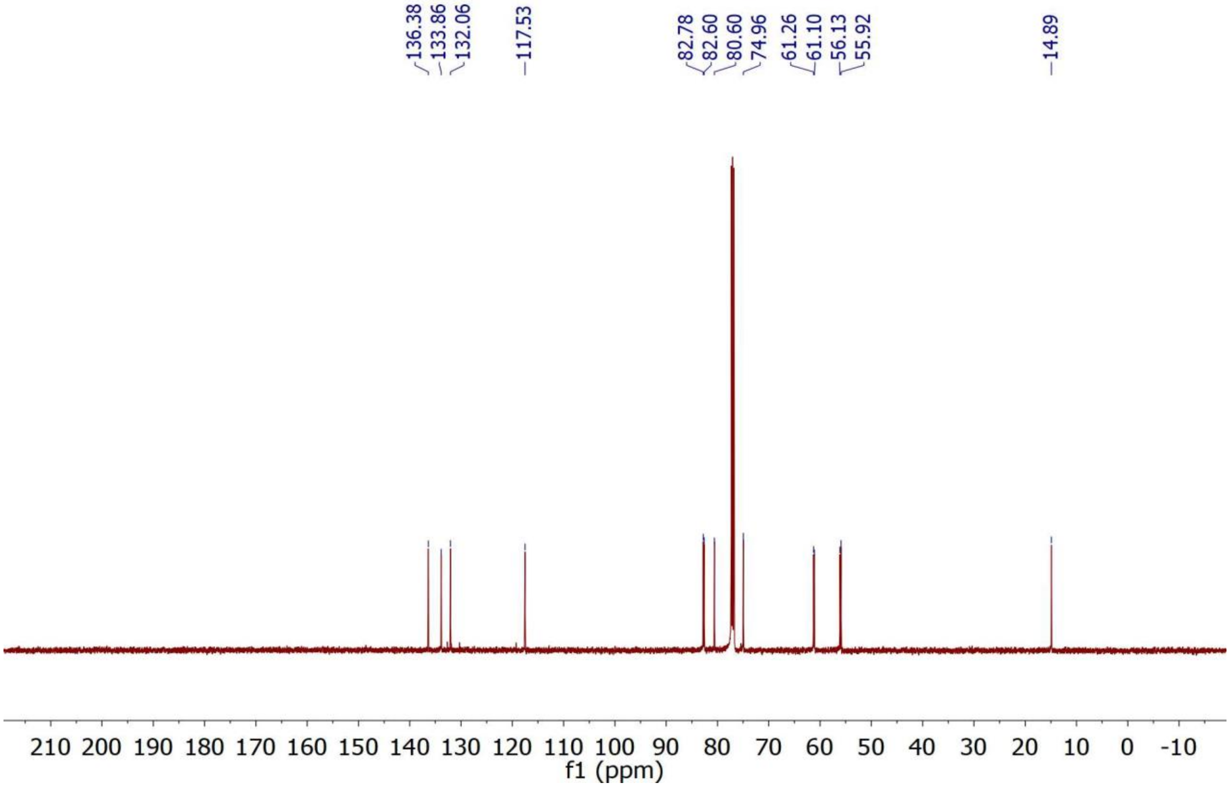


**(5*S*,6*R*,7*R*, *E*)-5,6,7,8-tetramethoxyocta-1,3-diene (4ad):**

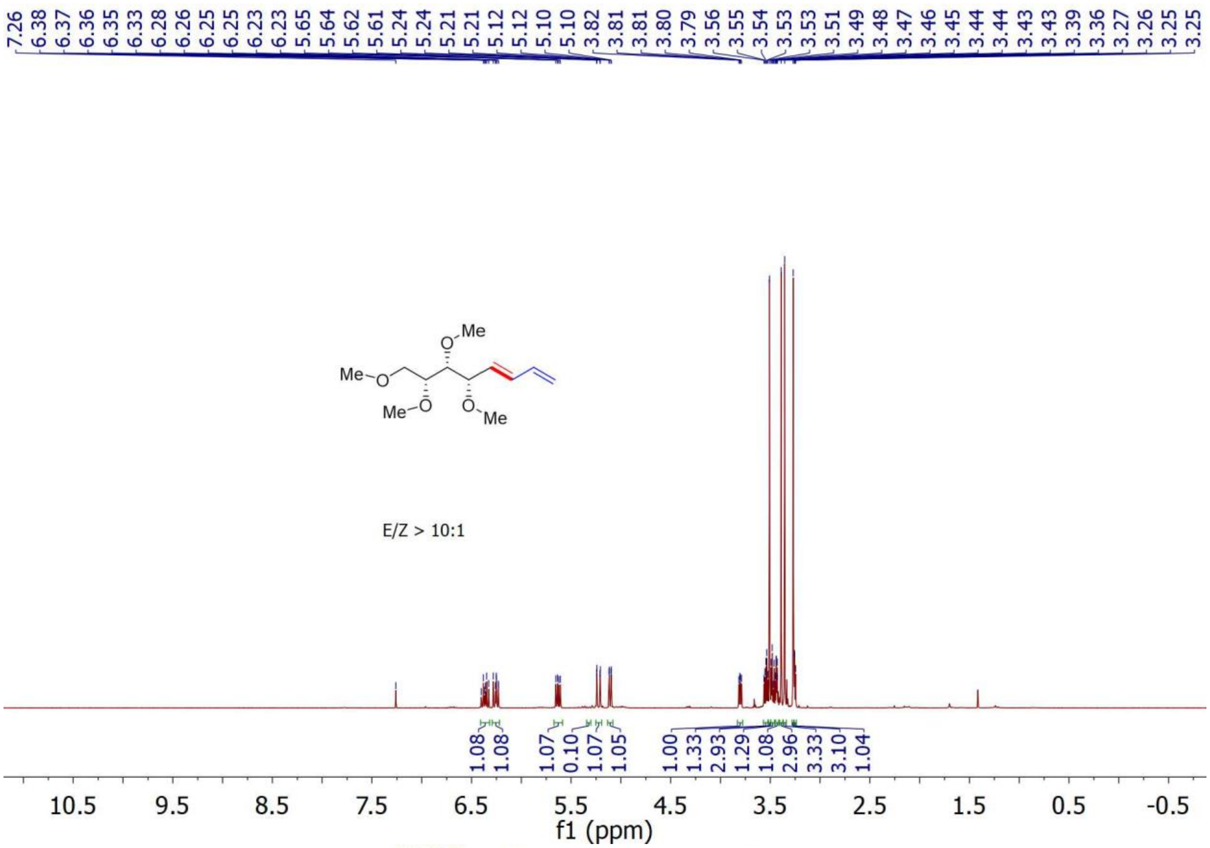

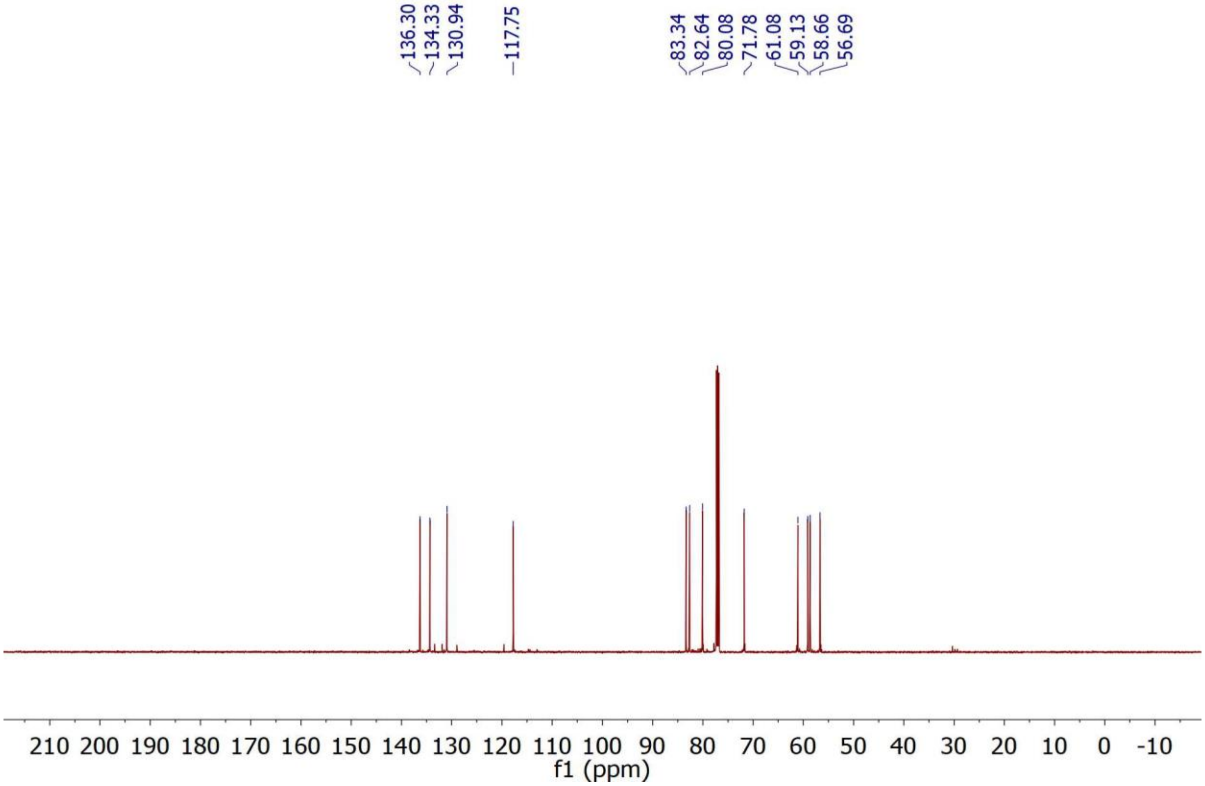

**(4S,4'R,5R)-5-((E)-buta-1,3-dien-1-yl)-2,2,2',2'-tetramethyl-4,4'-bi(1,3-dioxolane) (4ae):**

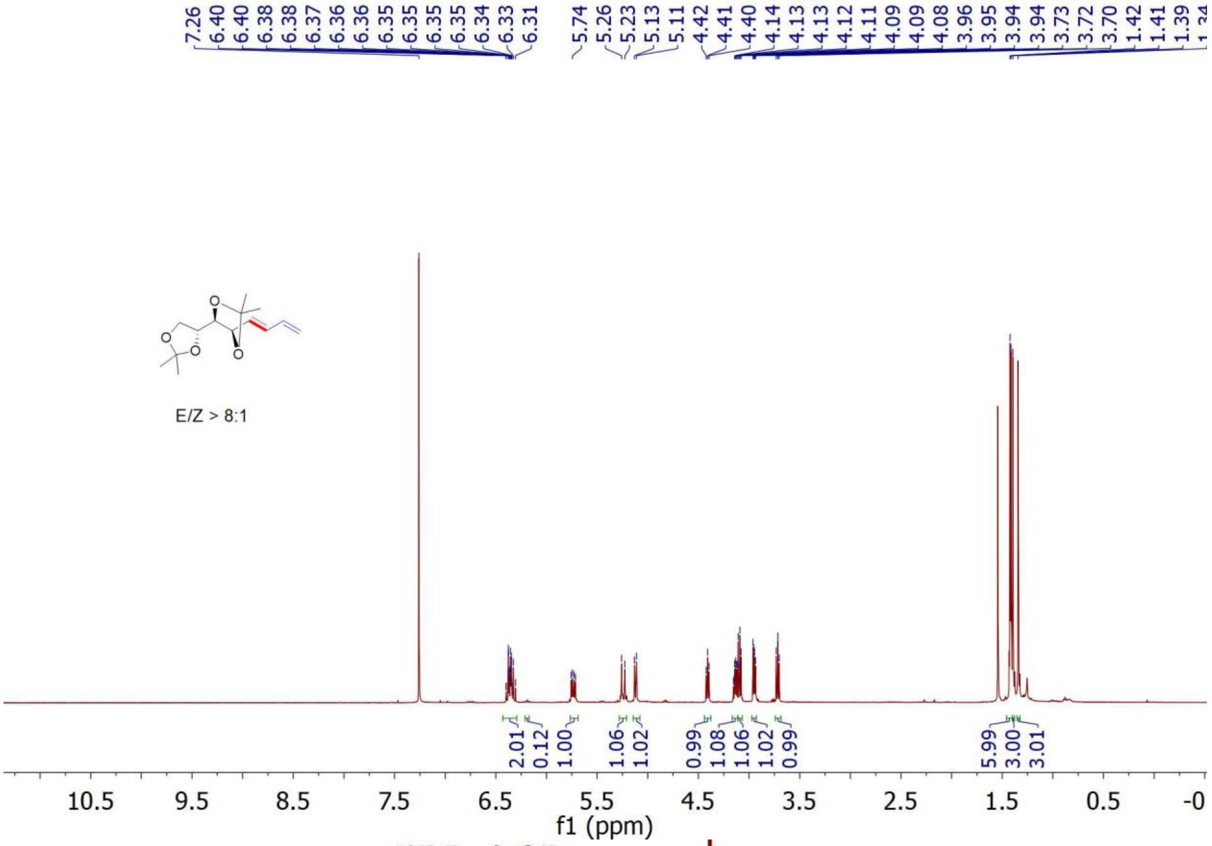


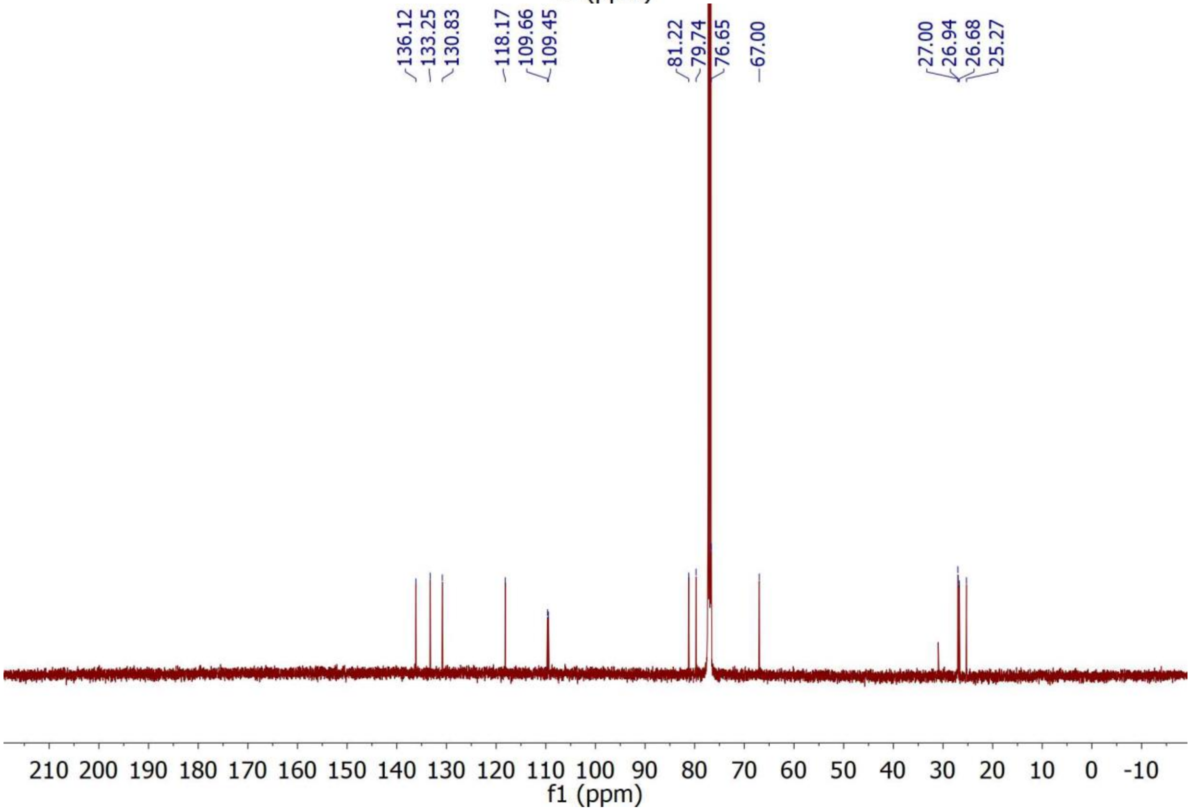


**(4R,4'S,5S)-5-((E)-buta-1,3-dien-1-yl)-2,2,2',2'-tetramethyl-4,4'-bi(1,3-dioxolane) (4af):**

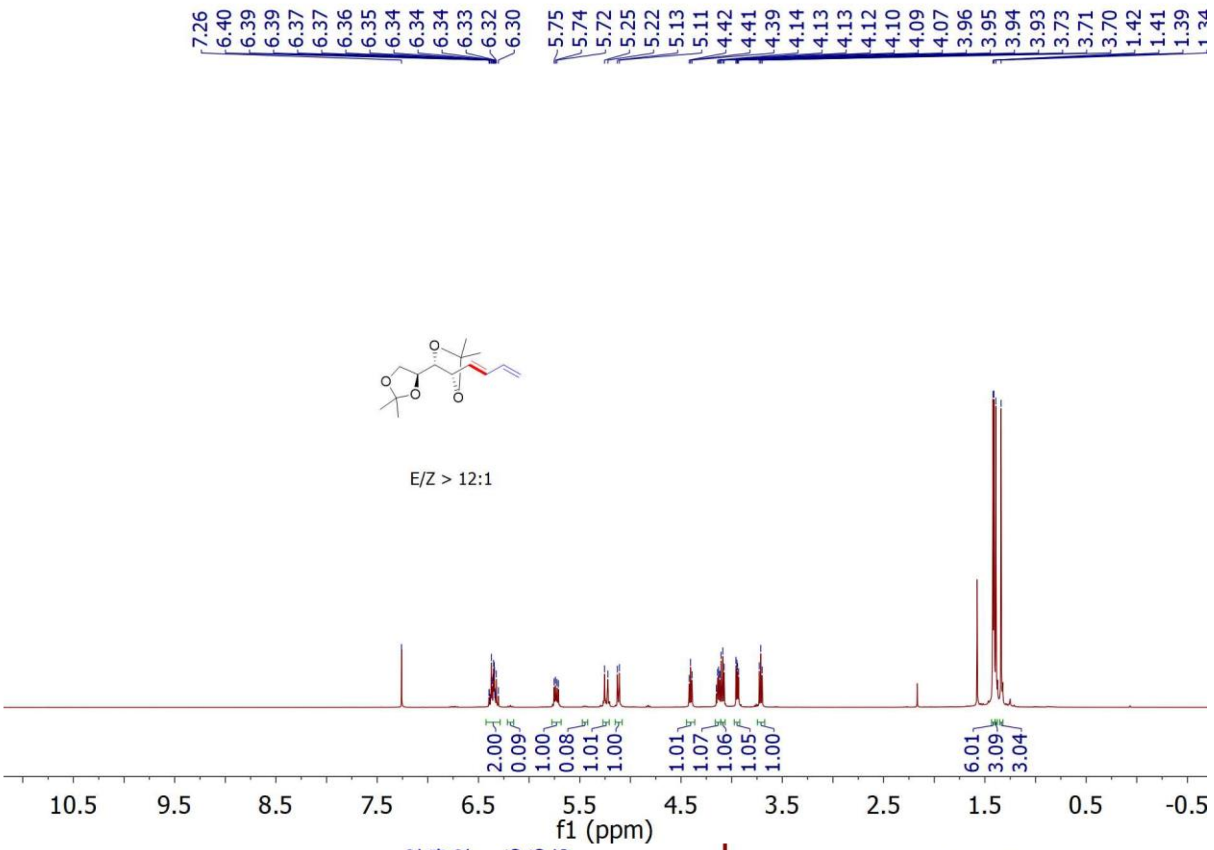

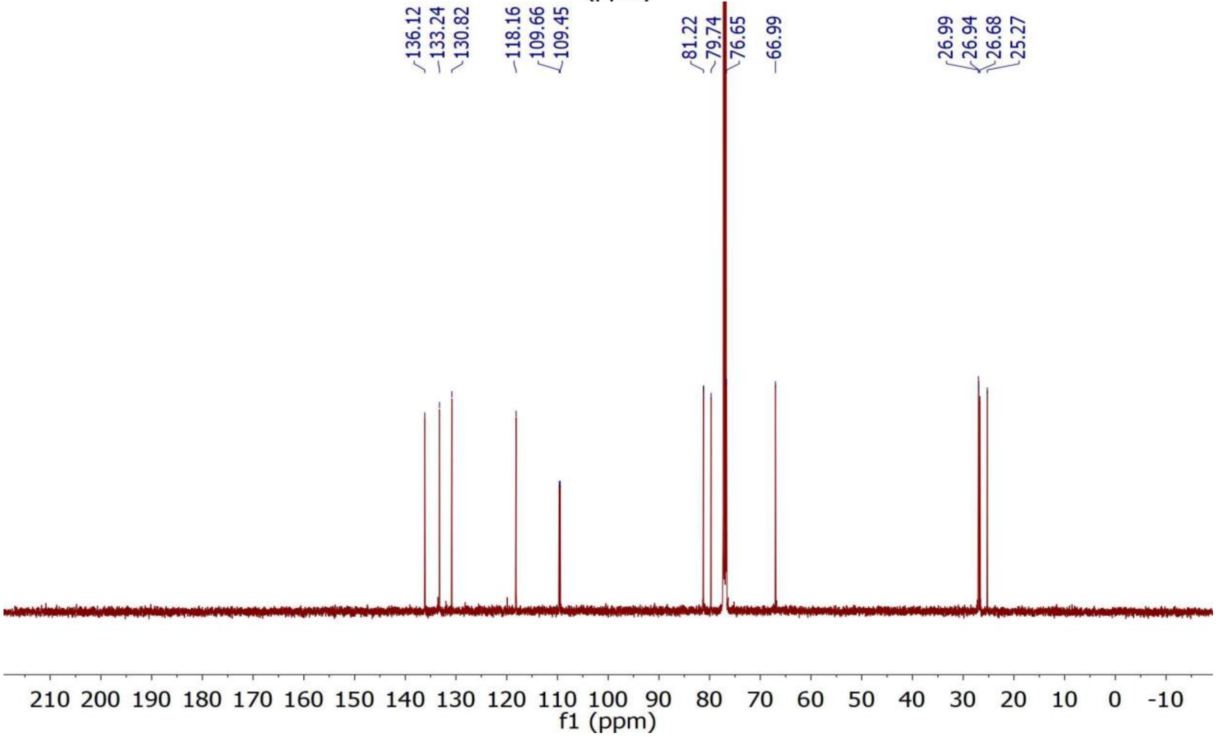

**(3a*S*,4*S*,8a*R*)-4-((*E*)-buta-1,3-dien-1-yl)-2,2,6,6-tetramethyltetrahydro-[1,3]dioxolo[4,5-*e*][1,3]dioxepine (4ag):**

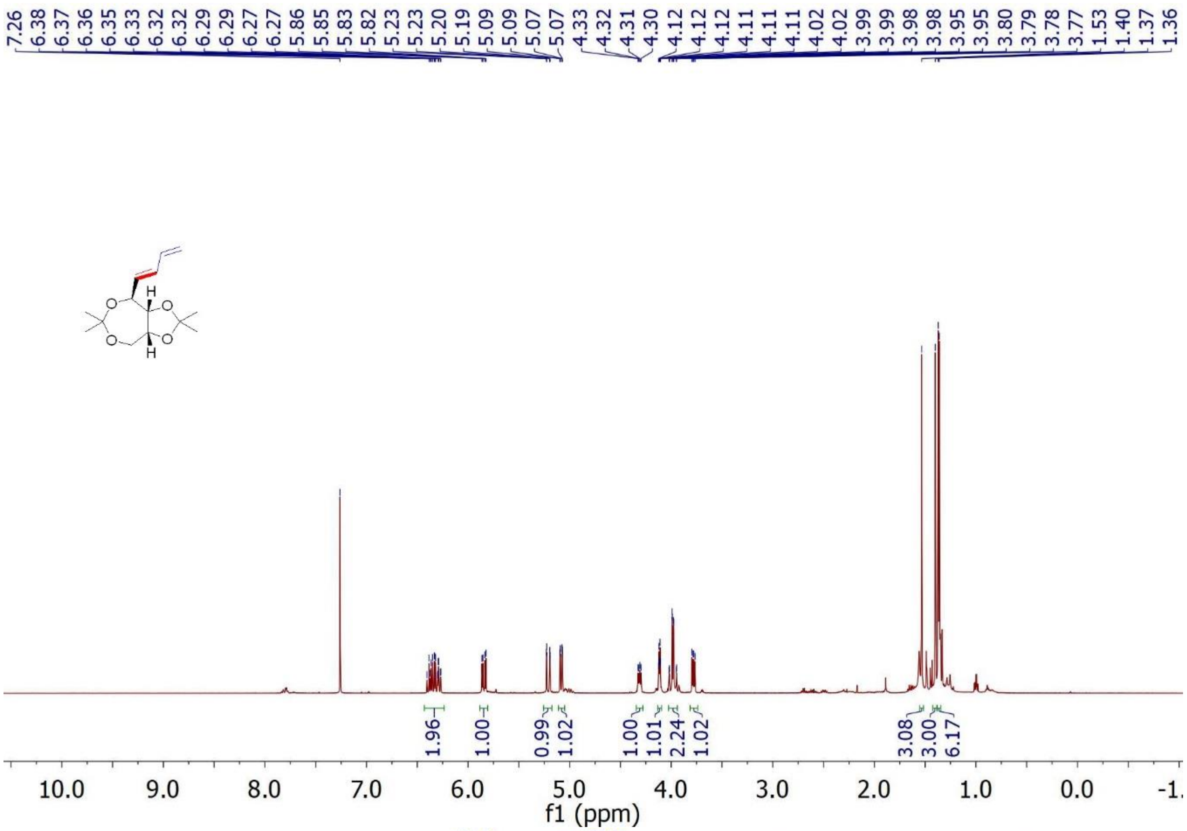


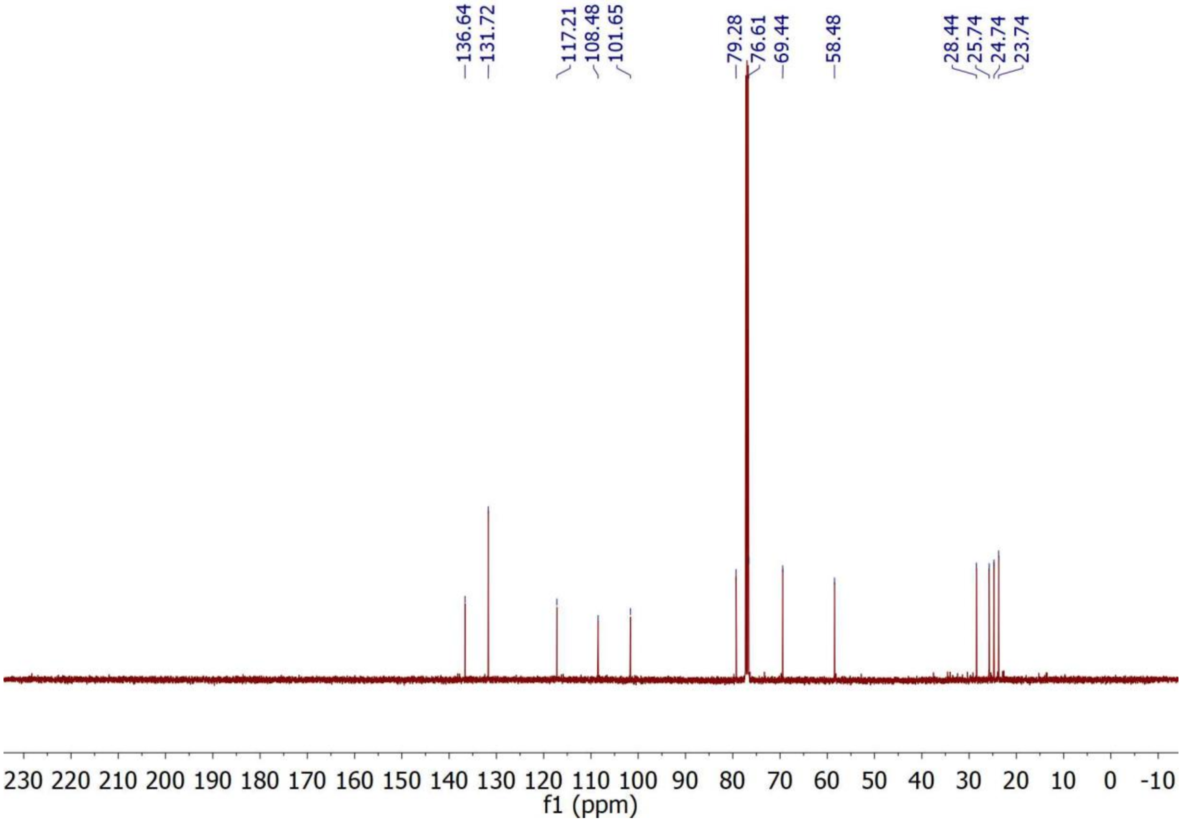


**(*S*, *E*)-4-(buta-1,3-dien-1-yl)-2,2-dimethyl-1,3-dioxolane (4ah):**

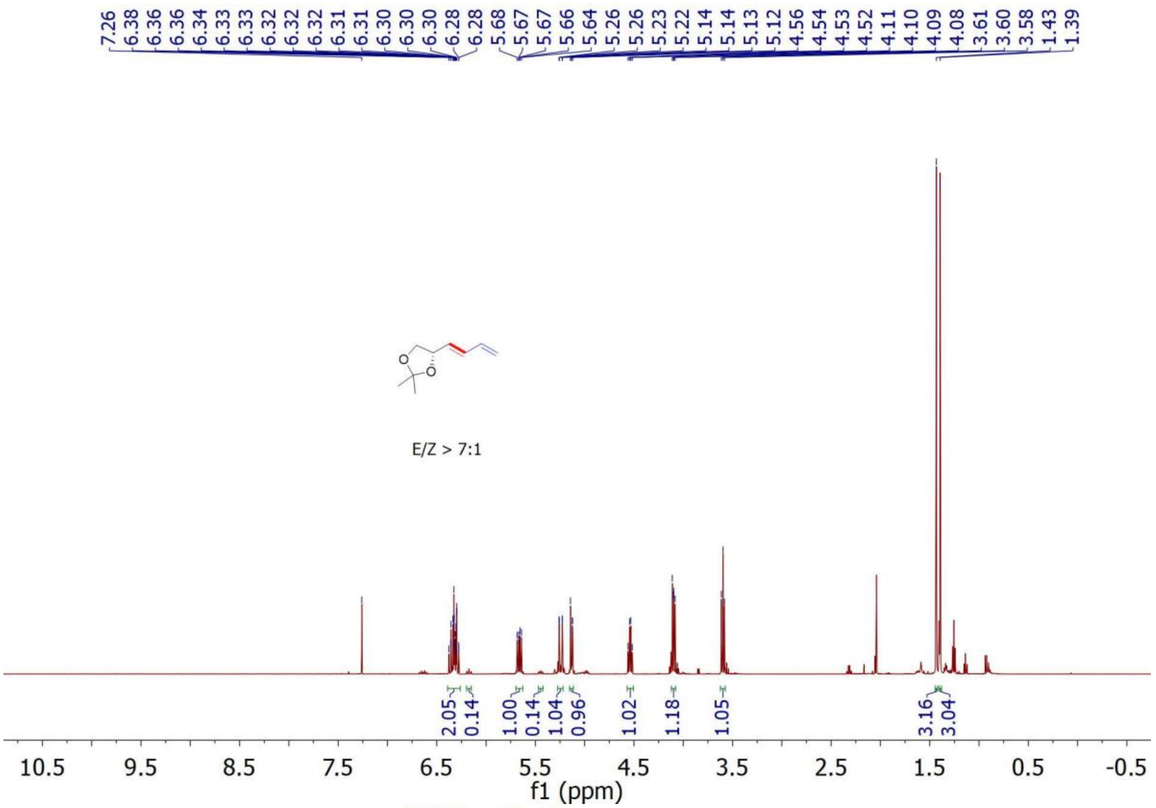


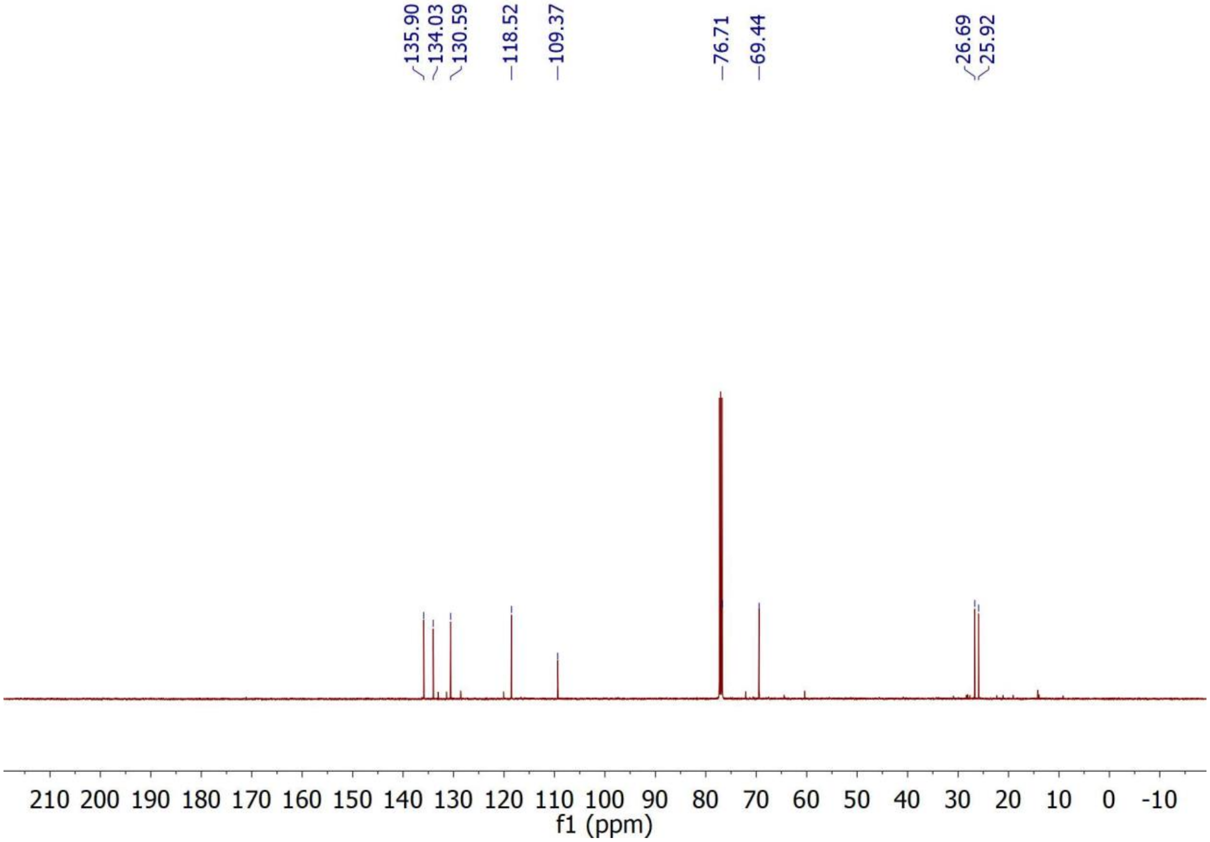


**(3a*R*,5*R*,5a*S*,8a*S*,8b*R*)-5-((*E*)-buta-1,3-dien-1-yl)-2,2,7,7-tetramethyltetrahydro-5*H*-bis([1,3]dioxolo)[4,5-*b*:4',5'-*d*]pyran (4ai):**

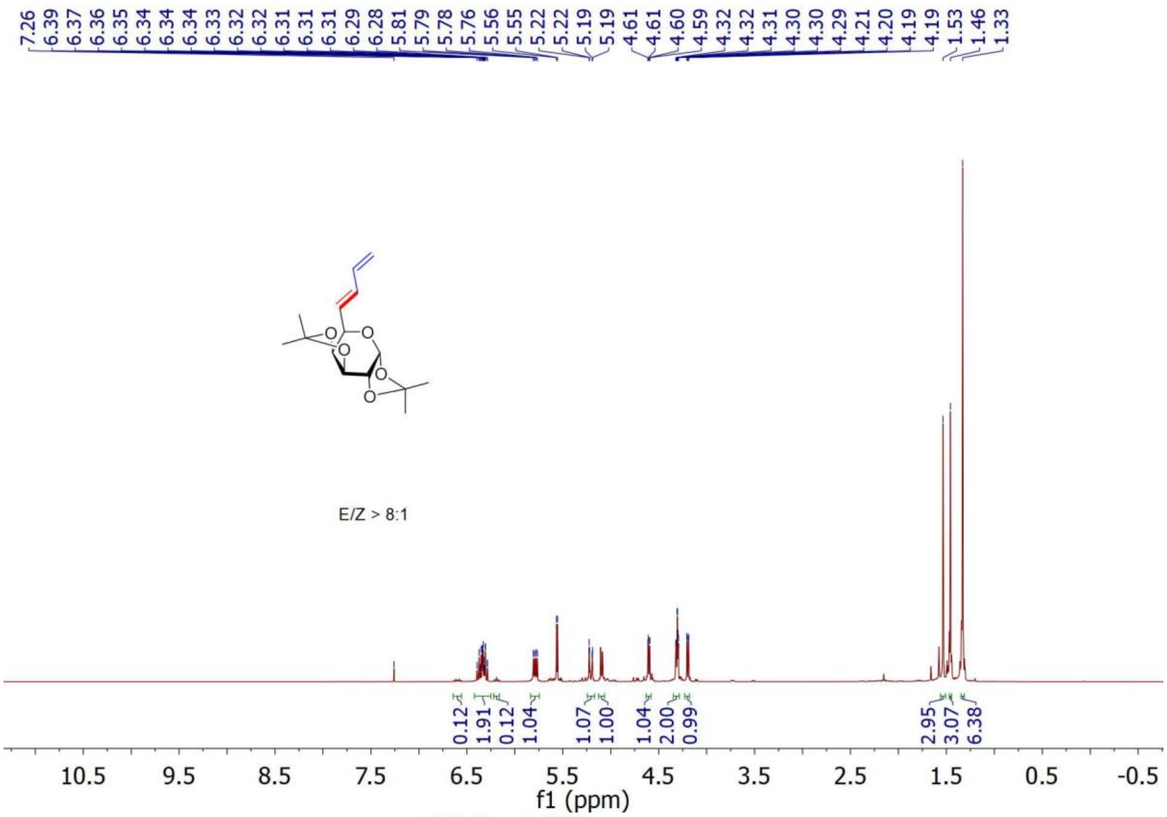


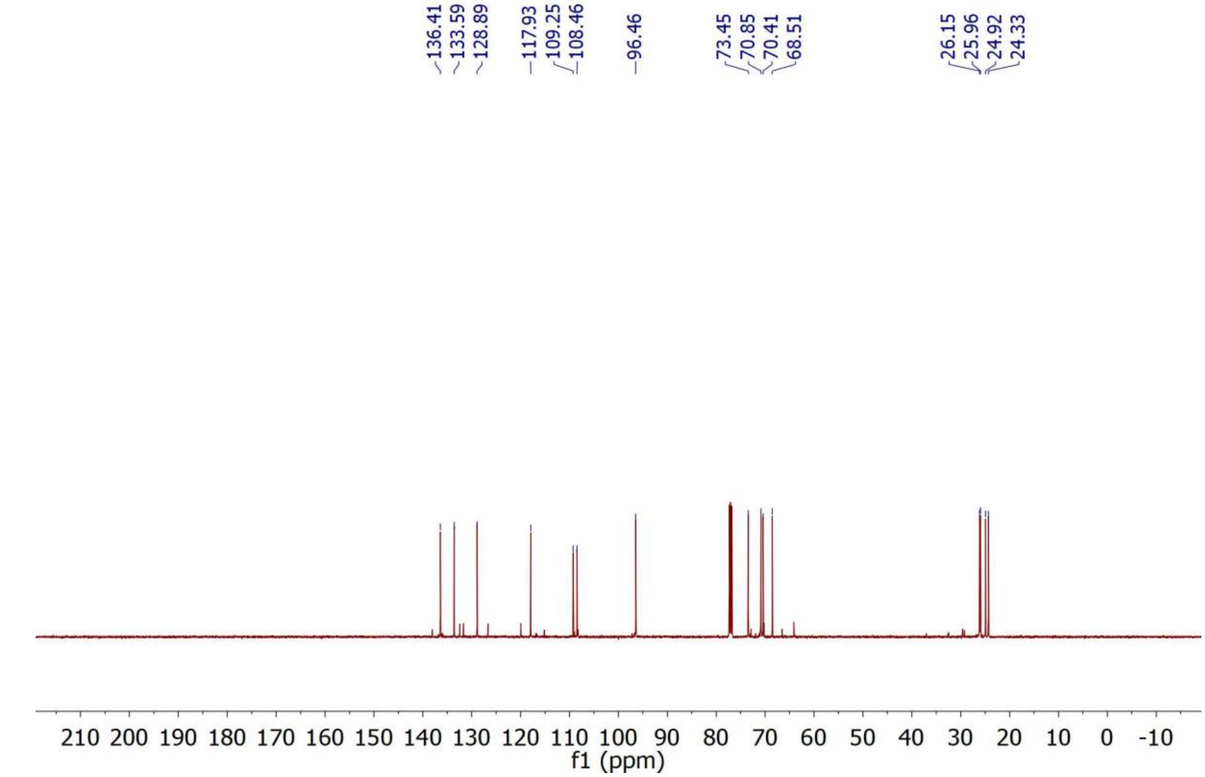


**(3a*S*,5a*R*,8a*R*,8b*S*)-3a-((*E*)-buta-1,3-dien-1-yl)-2,2,7,7-tetramethyltetrahydro-5*H*-bis([1,3]dioxolo)[4,5-*b*:4',5'-*d*]pyran (4aj):**

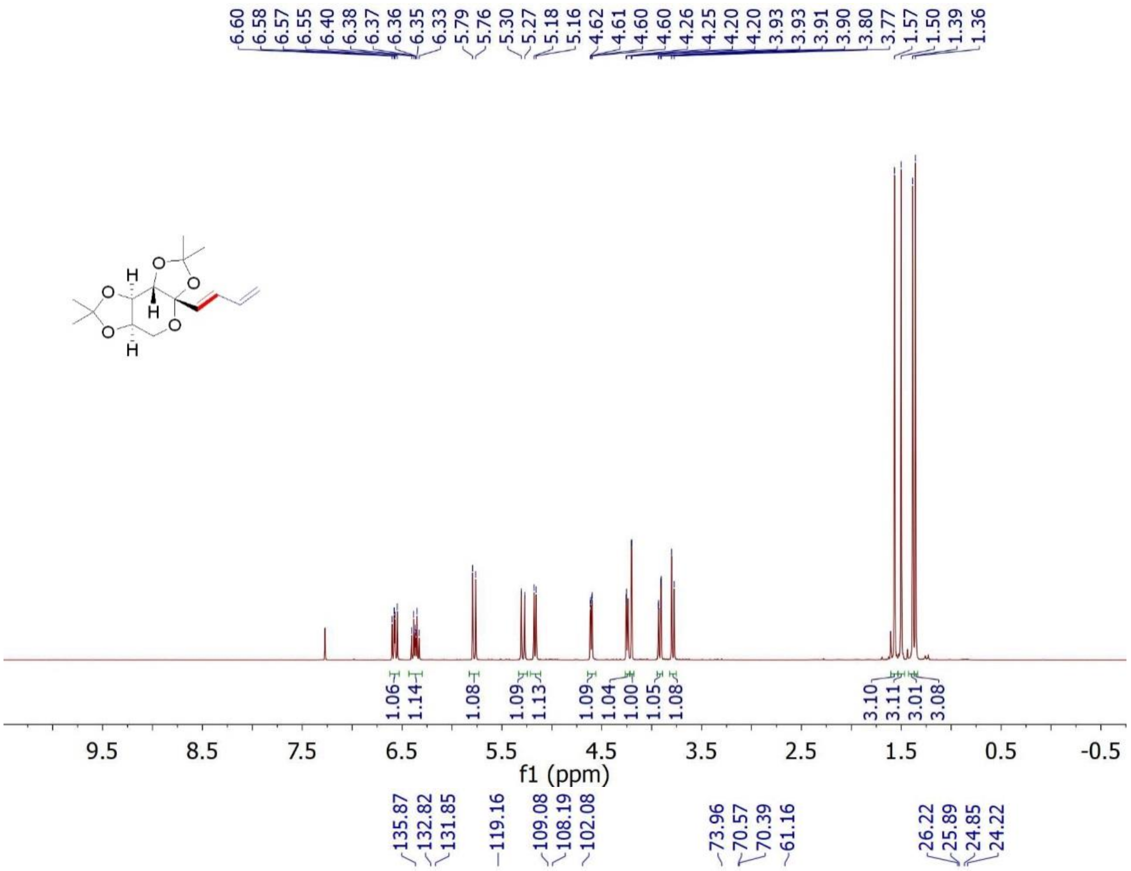


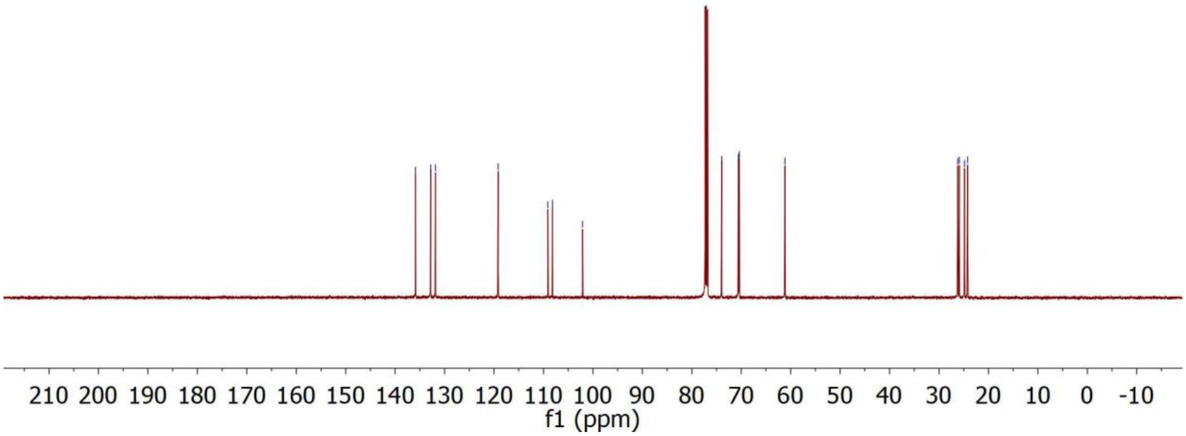


**(3a*R*,4*R*,6*R*,6a*R*)-4-((*E*)-buta-1,3-dien-1-yl)-6-methoxy-2,2-dimethyltetrahydrofuro[3,4-*d*][1,3]dioxole (4ak):**

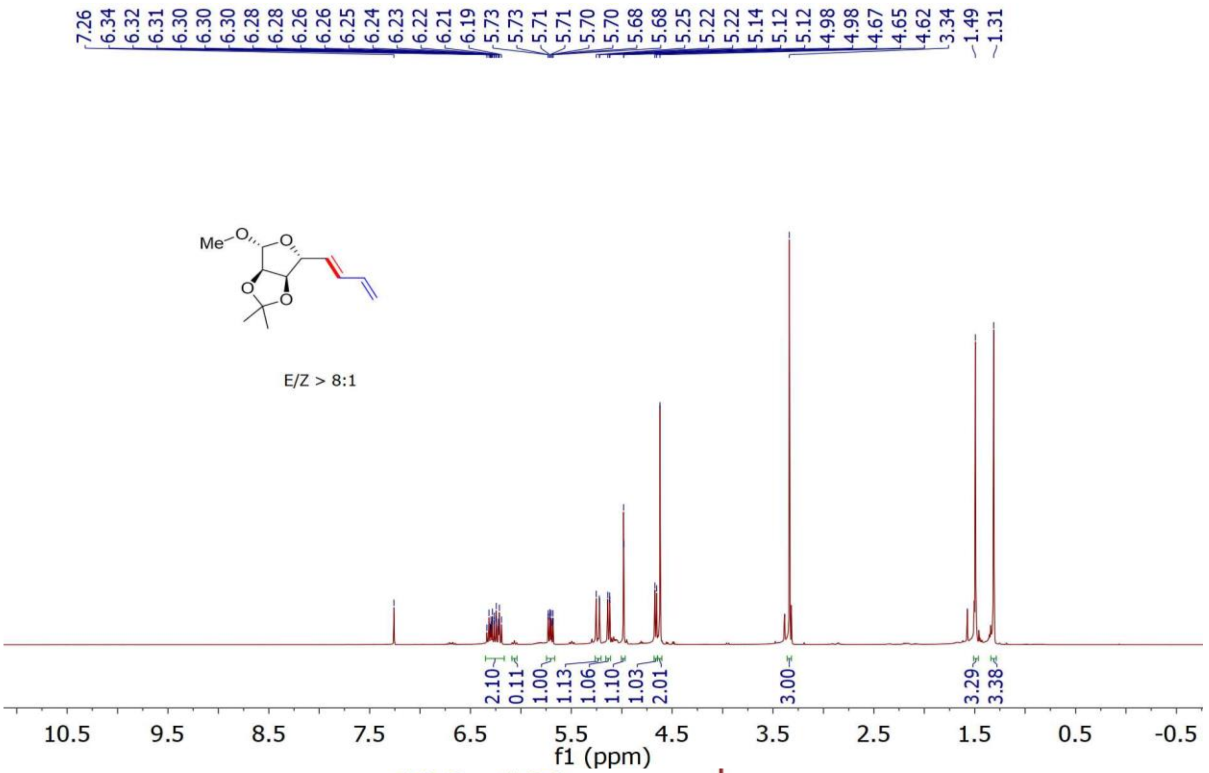
7.26 6.34 6.32 6.31 6.30 6.30 6.30 6.28 6.28 6.26 6.26 6.25 6.24 6.23 6.22 6.21 6.19 5.73 5.73 5.71 5.71 5.70 5.70 5.68 5.68 5.25 5.22 5.22 5.14 5.12 5.12 4.98 4.98 4.67 4.65 4.62 3.34 1.49 1.31
Me
O
O
O
O
E/Z > 8:1
2.10 0.11 1.00 1.13 1.06 1.10 1.03 2.01 3.00 3.29 3.38
10.5 9.5 8.5 7.5 6.5 5.5 4.5 3.5 2.5 1.5 0.5 -0.5
f1 (ppm)

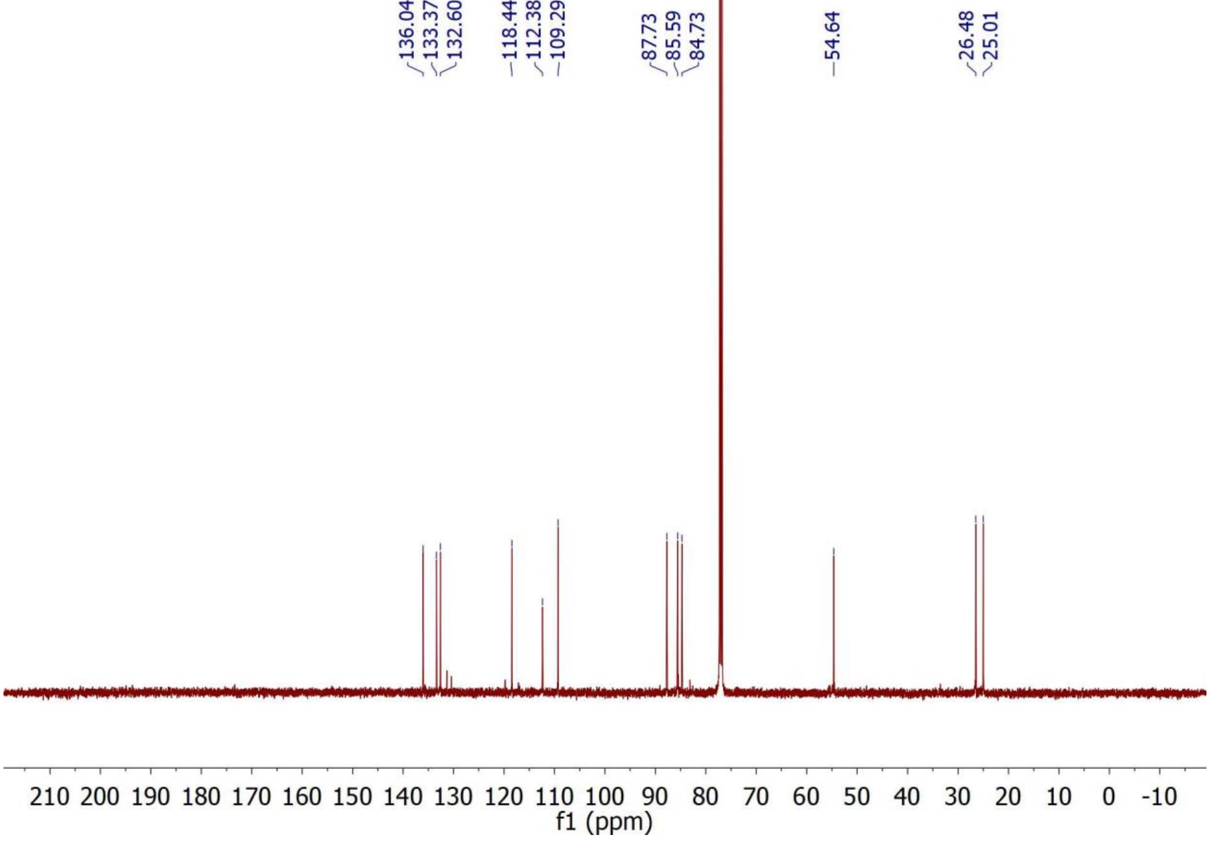
136.04 133.37 132.60 118.44 112.38 109.29 87.73 85.59 84.73 54.64 26.48 25.01
210 200 190 180 170 160 150 140 130 120 110 100 90 80 70 60 50 40 30 20 10 0 -10
f1 (ppm)

**(2S,3R,4S,E)-octa-5,7-diene-1,2,3,4-tetraol (4al):**

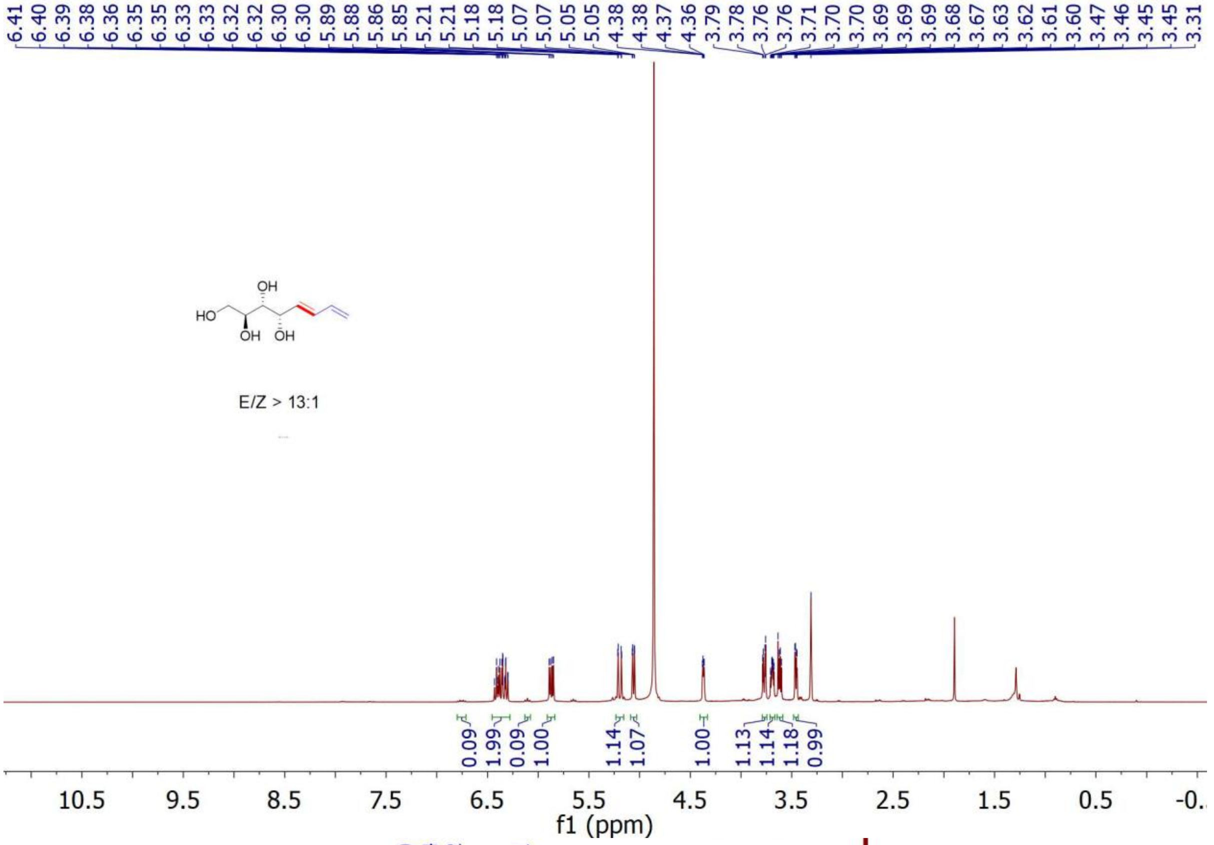


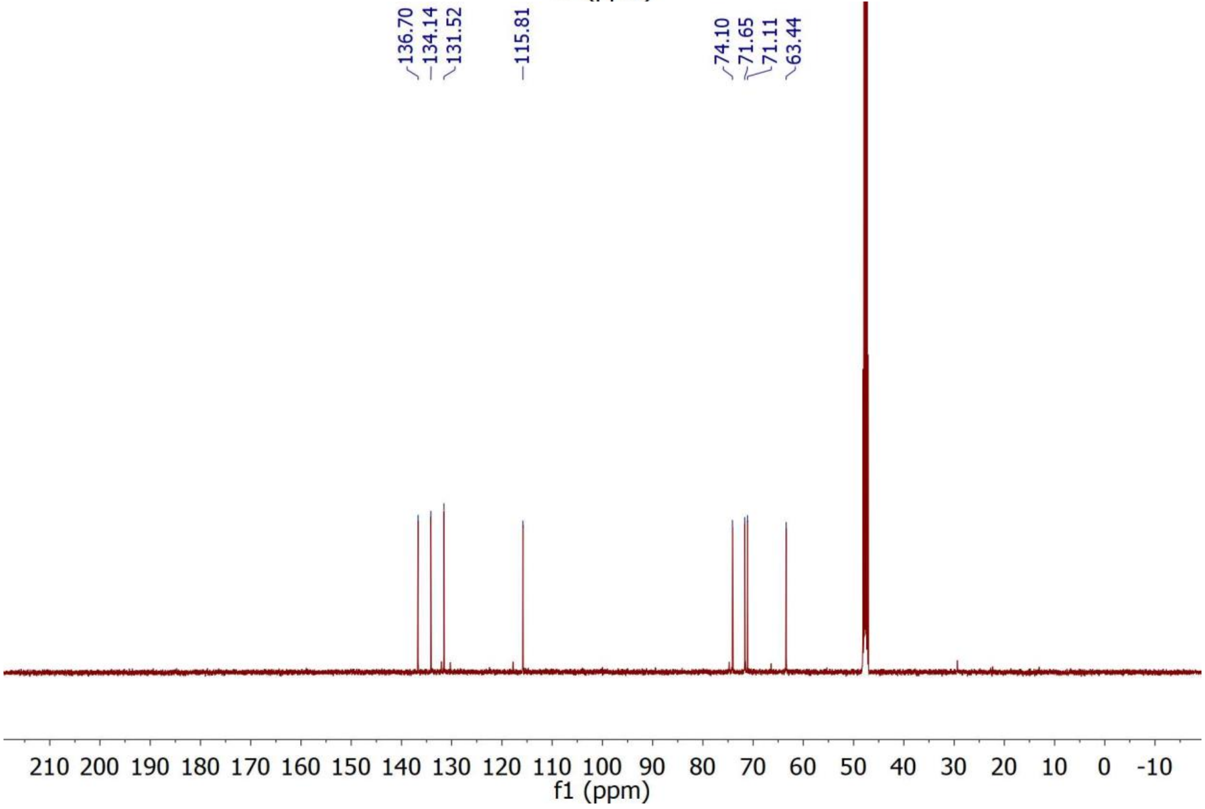

**(2*S*,3*R*,4*S*,5*R*, *E*)-nona-6,8-diene-2,3,4,5-tetraol (4am):**

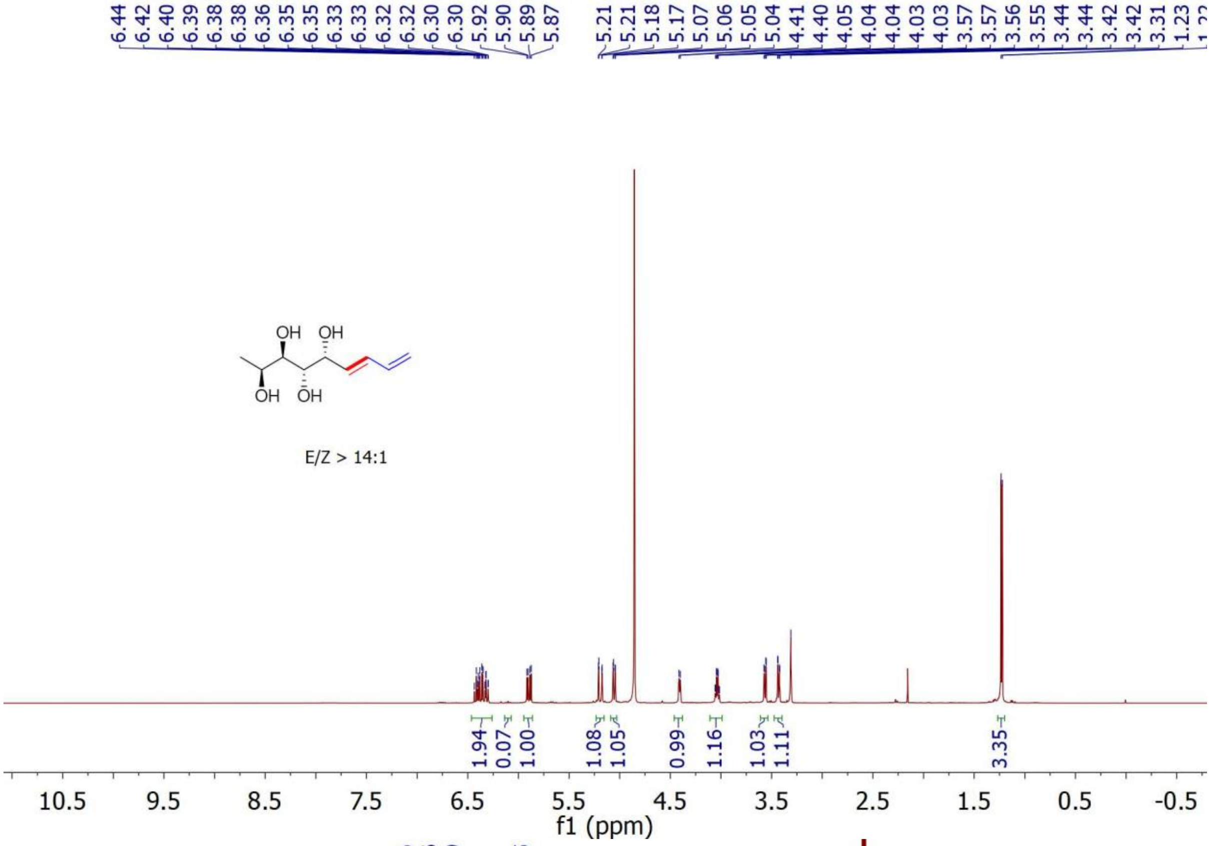


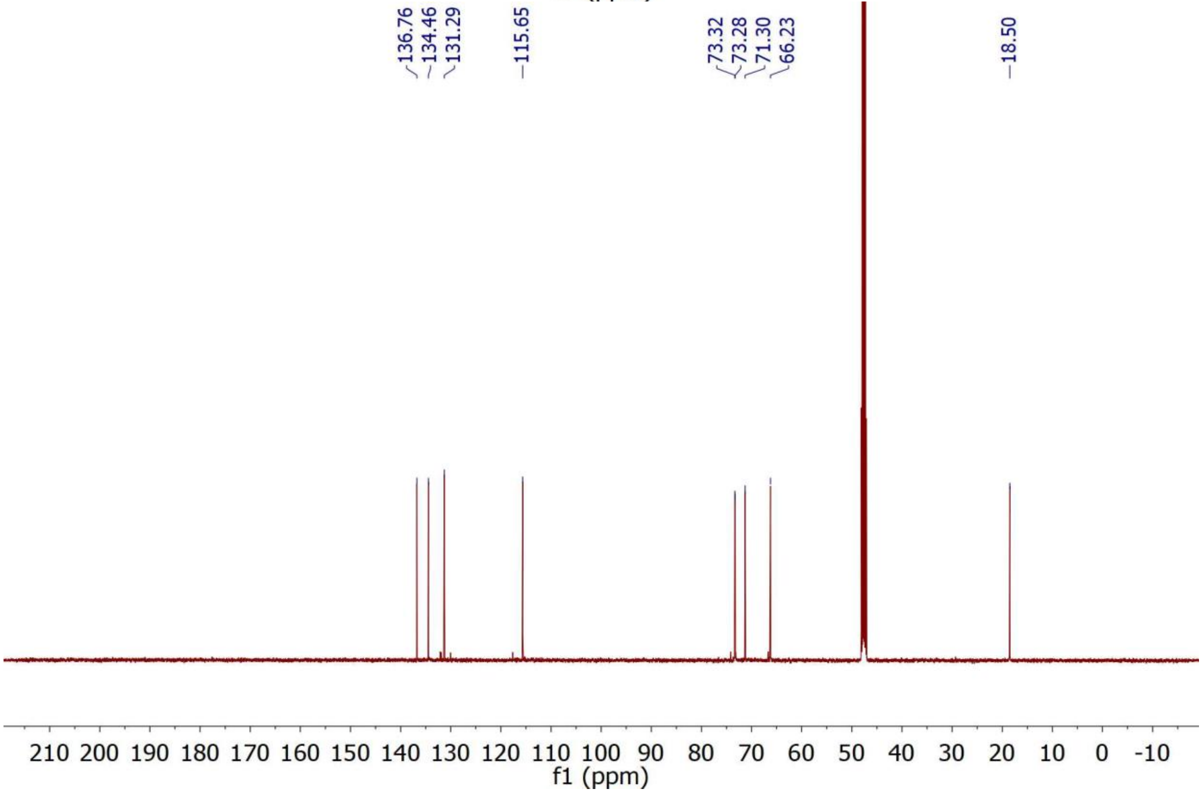


**(2*R*,3*S*,4*R*, *E*)-octa-5,7-diene-1,2,3,4-tetraol (4an):**

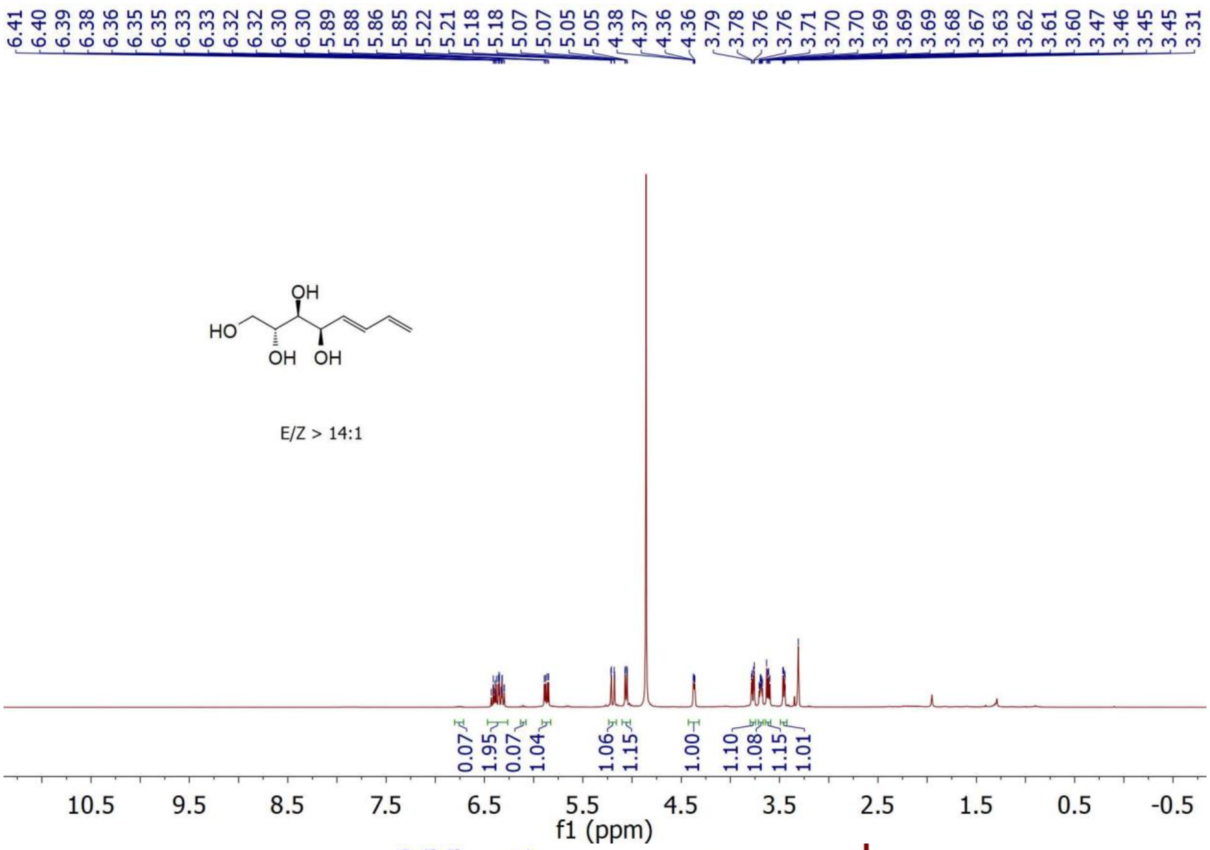


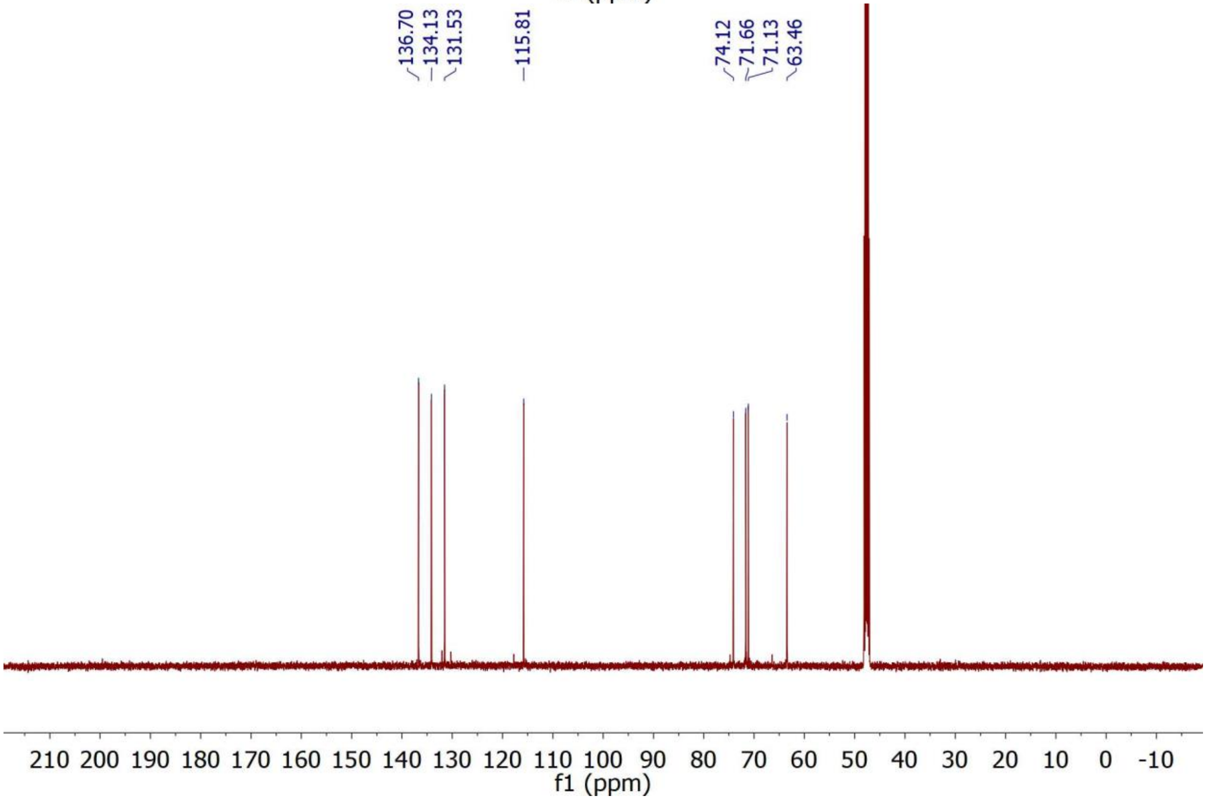


**(2S,3S,4S,5S, E)-nona-6,8-diene-2,3,4,5-tetraol (4ao):**

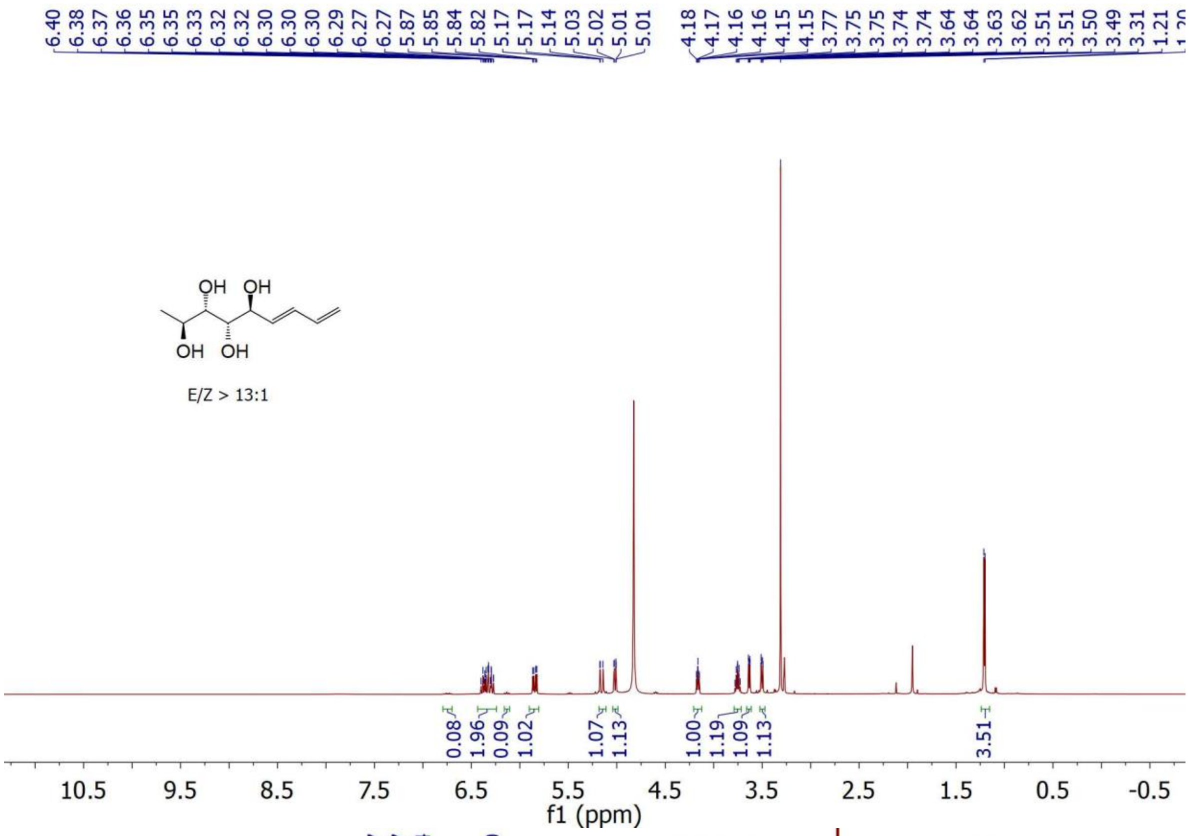
6.40 6.38 6.37 6.36 6.35 6.35 6.33 6.32 6.32 6.30 6.30 6.29 6.27 6.27 5.87 5.85 5.84 5.82 5.17 5.17 5.14 5.03 5.02 5.01 5.01
4.18 4.17 4.16 4.16 4.15 4.15 3.77 3.75 3.75 3.74 3.74 3.64 3.64 3.63 3.62 3.51 3.51 3.50 3.49 3.31 1.21
OH OH
OH OH
E/Z > 13:1
0.08 1.96 0.09 1.02 1.07 1.13 1.00 1.19 1.09 1.13 3.51
10.5 9.5 8.5 7.5 6.5 5.5 4.5 3.5 2.5 1.5 0.5 -0.5
f1 (ppm)
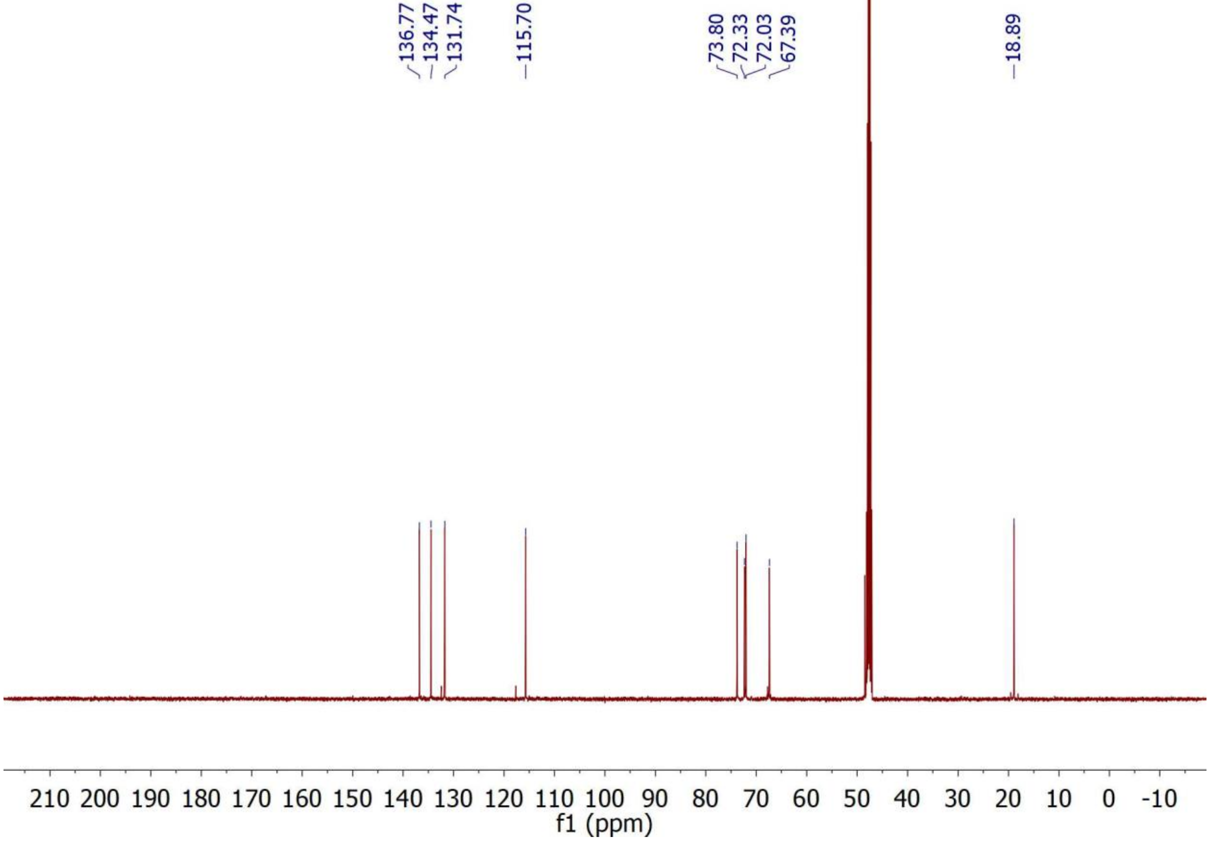
136.77 134.47 131.74 115.70 73.80 72.33 72.03 67.39 18.89
210 200 190 180 170 160 150 140 130 120 110 100 90 80 70 60 50 40 30 20 10 0 -10
f1 (ppm)

**(2*R*,3*R*,4*S*, *E*)-1,3,4-tris(benzyloxy)octa-5,7-dien-2-ol (4ap):**

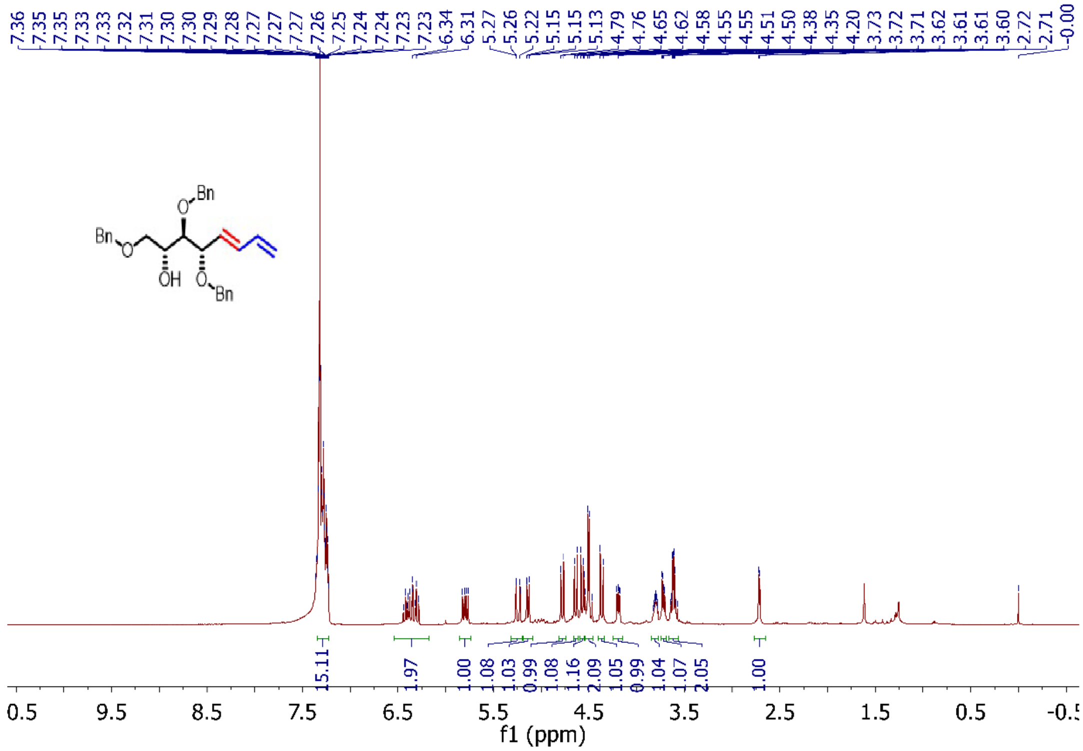

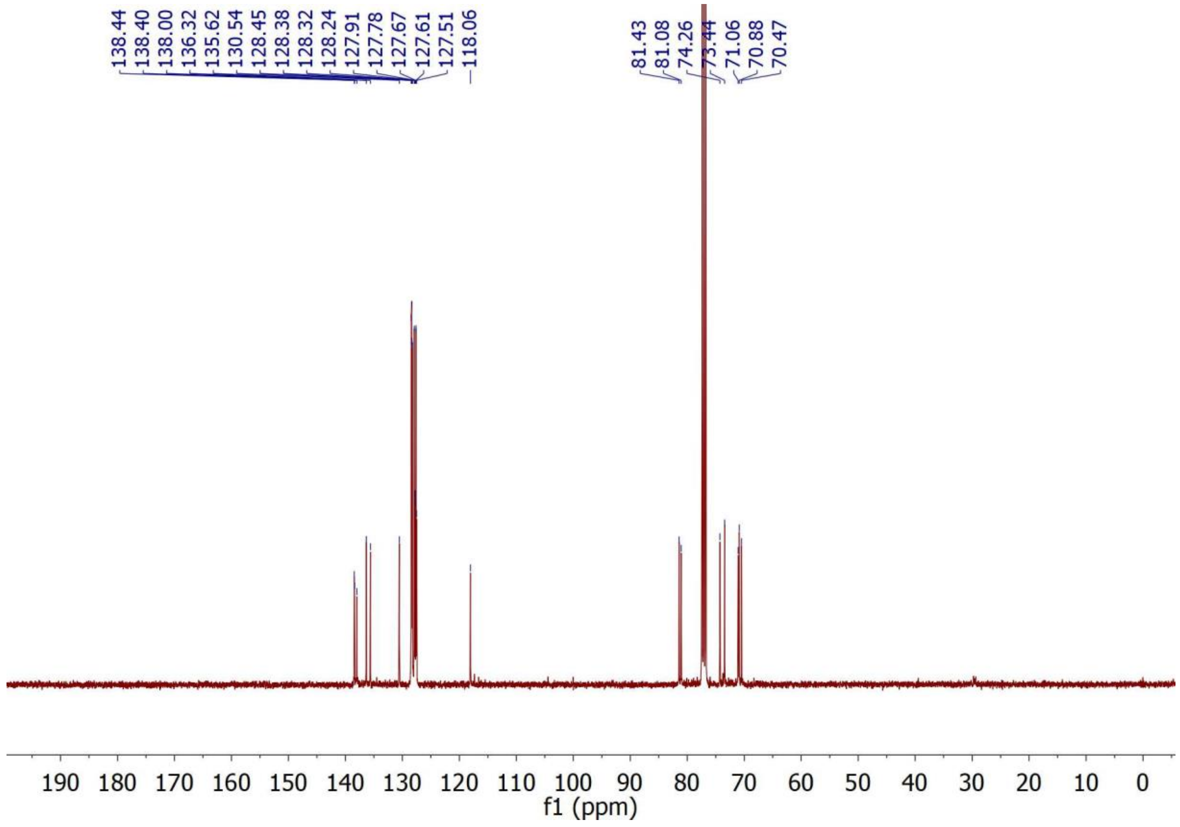

**(5S,6S,7R)-5,6,7,8-tetramethoxy-2-methyloct-1-ene (3ba):**

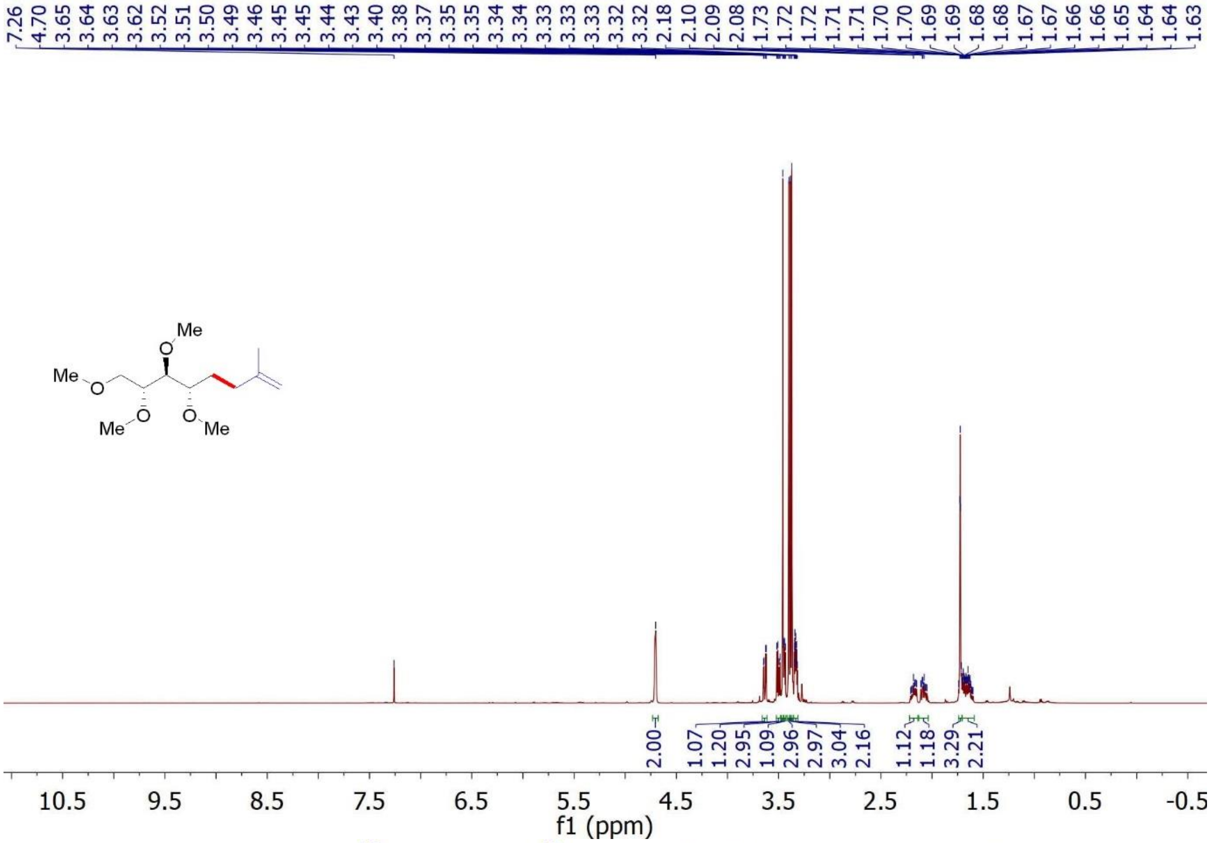


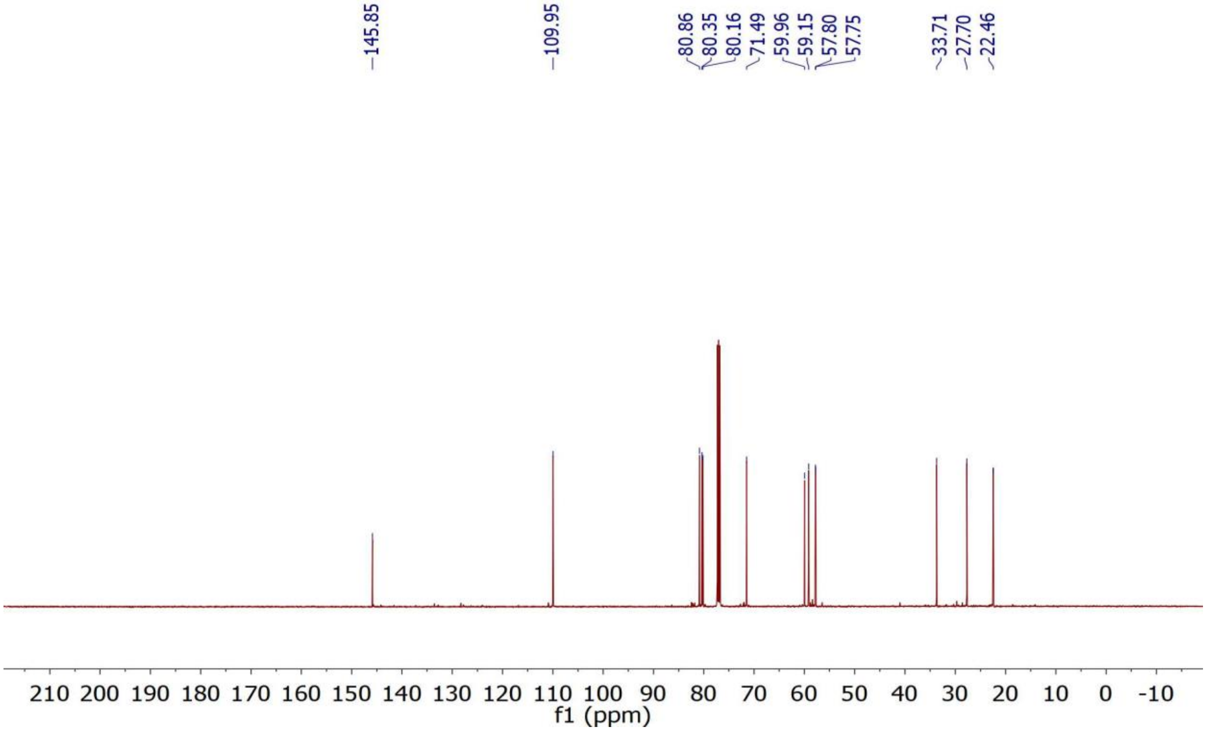

**(5S,6S,7R)-2-chloro-5,6,7,8-tetramethoxyoct-1-ene (3bb):**

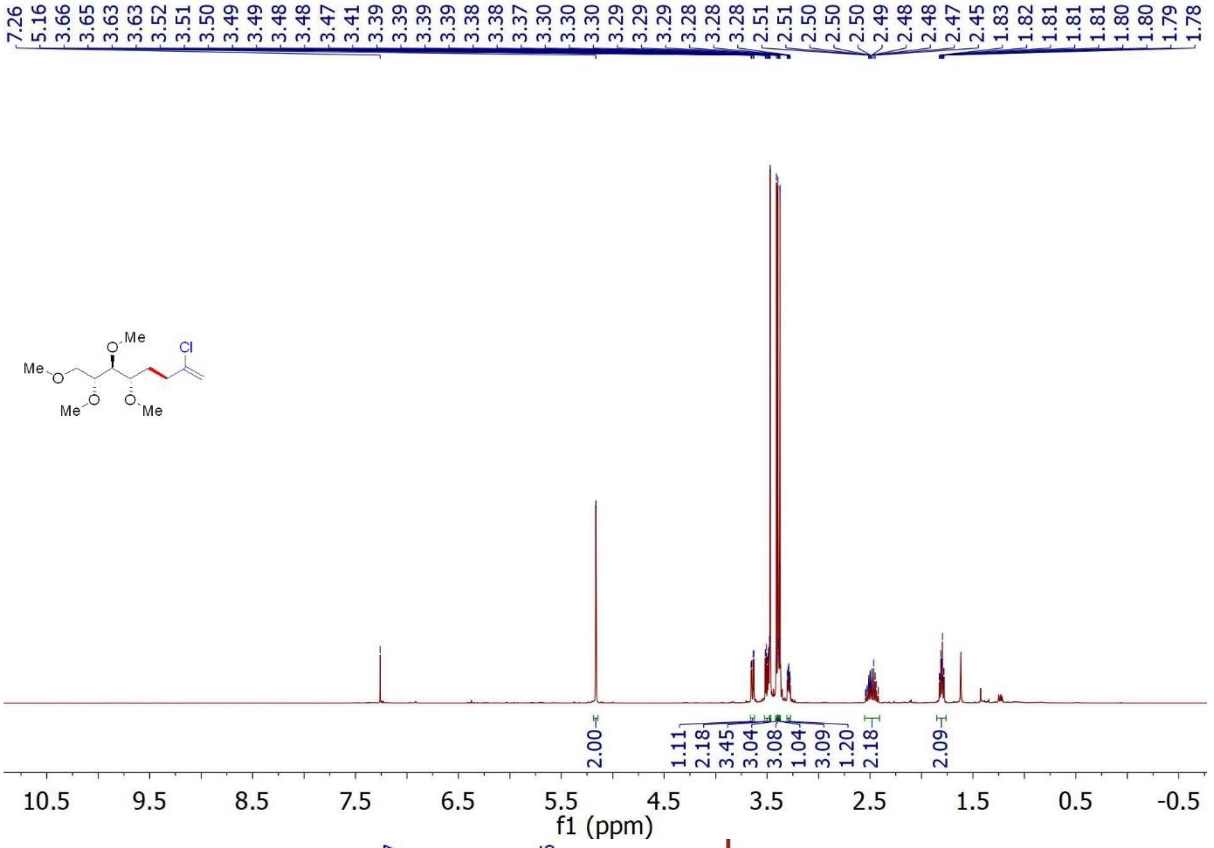


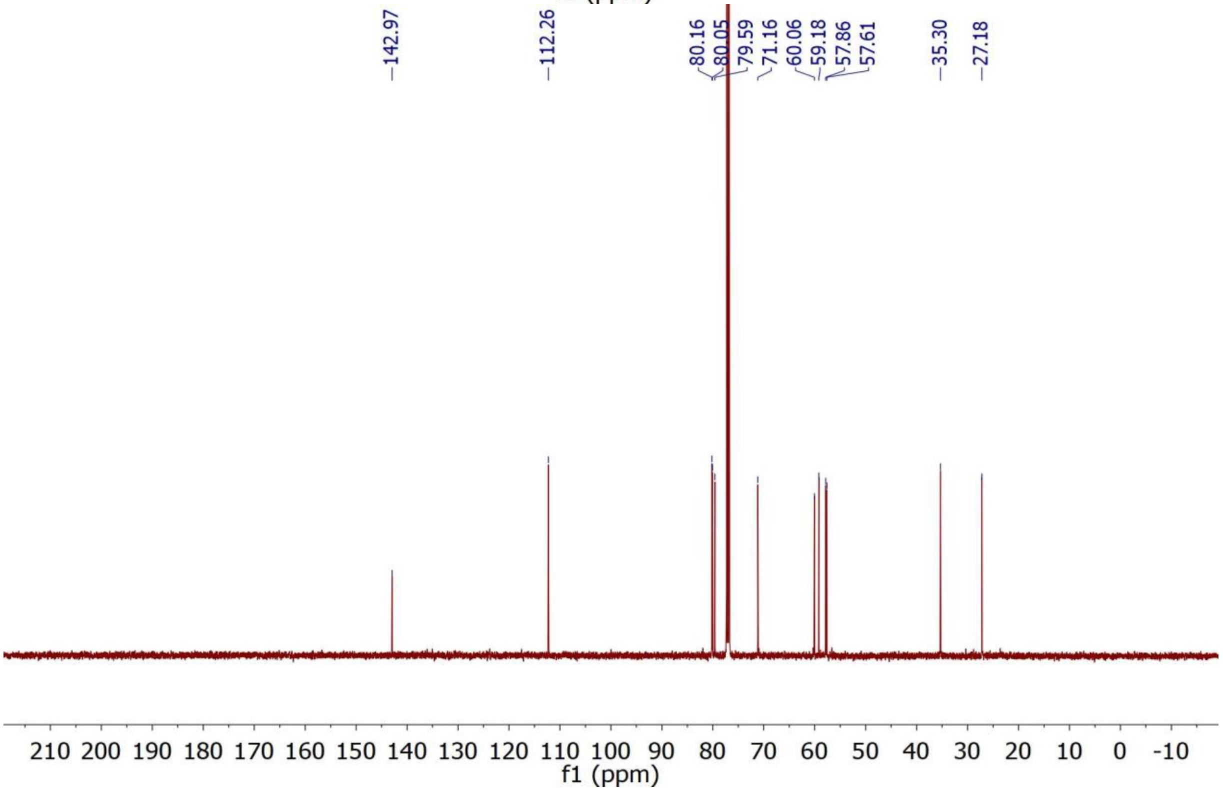

**((5S,6S,7R)-5,6,7,8-tetramethoxyoct-1-en-2-yl)benzene (3bc):**

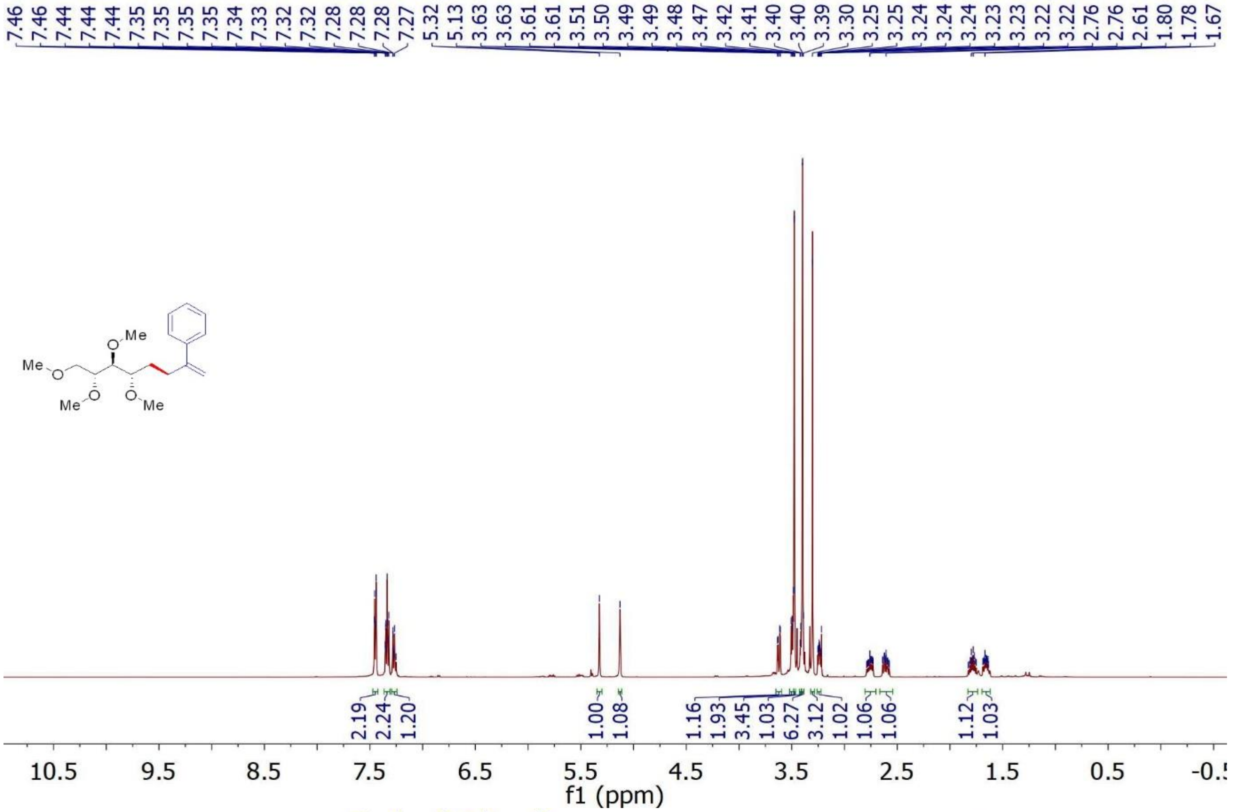


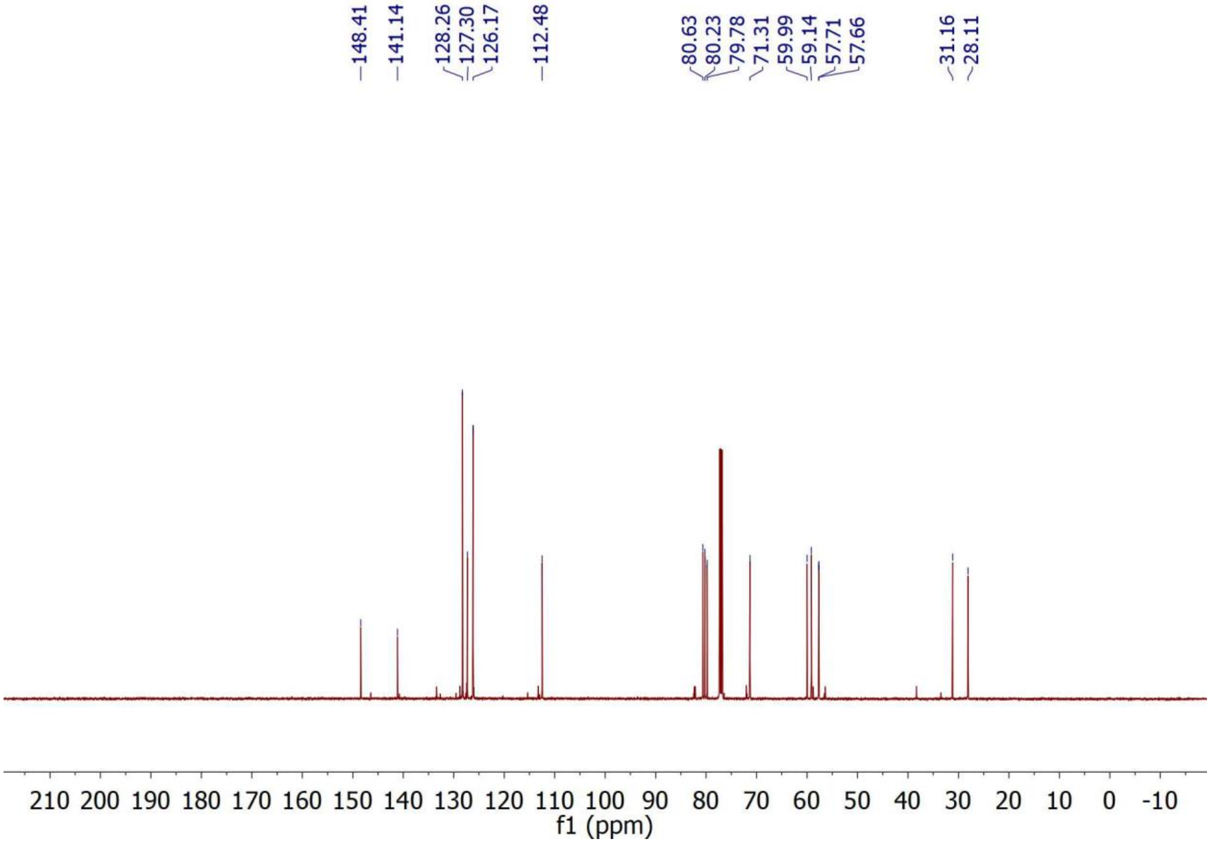

**1-chloro-4-((5S,6S,7R)-5,6,7,8-tetramethoxyoct-1-en-2-yl)benzene (3bd):**

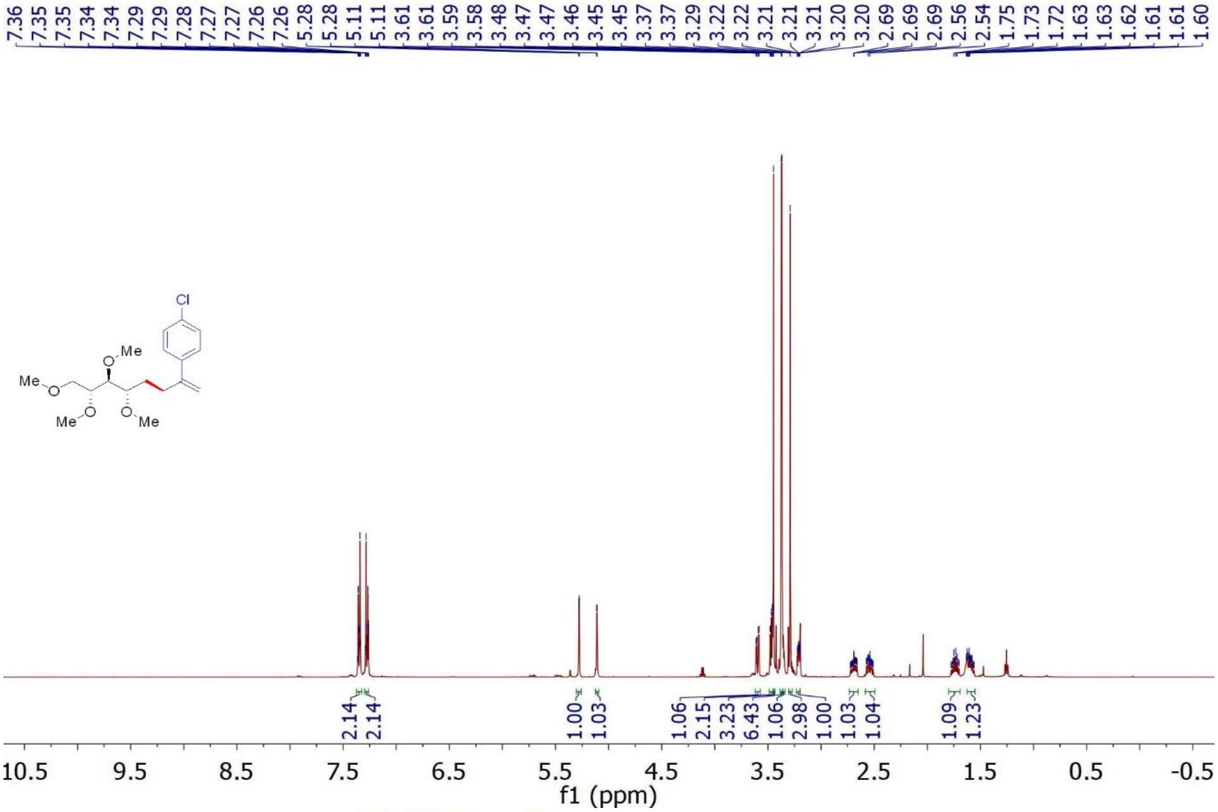


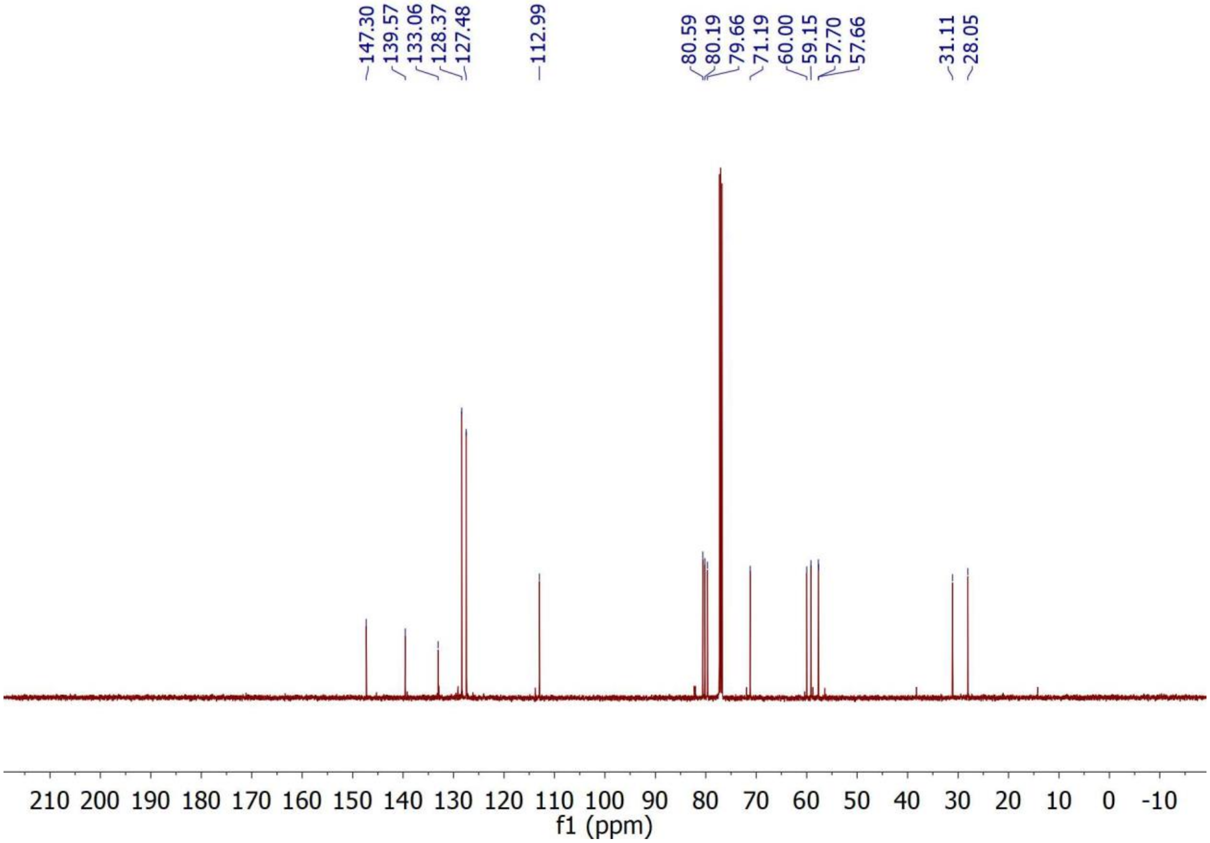

**1-methoxy-4-((5S,6S,7R)-5,6,7,8-tetramethoxyoct-1-en-2-yl)benzene (3be):**

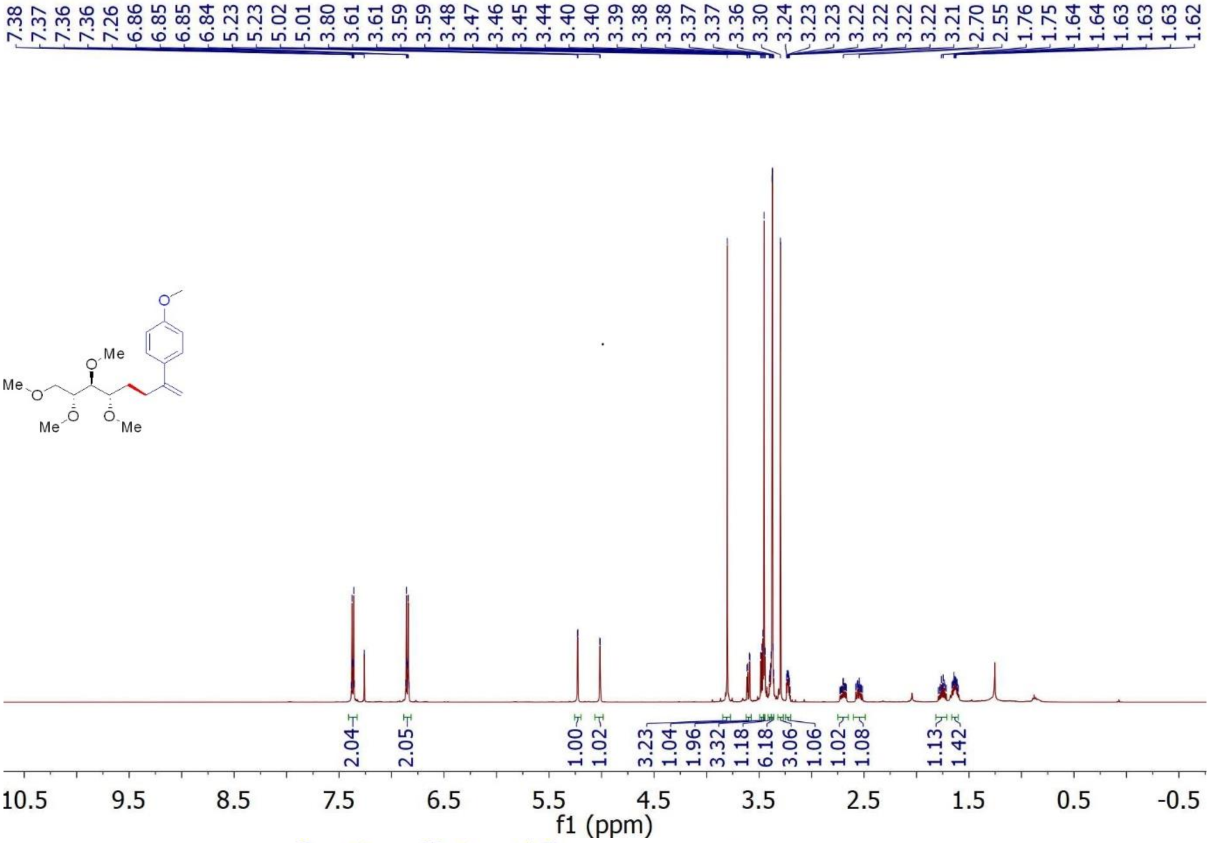


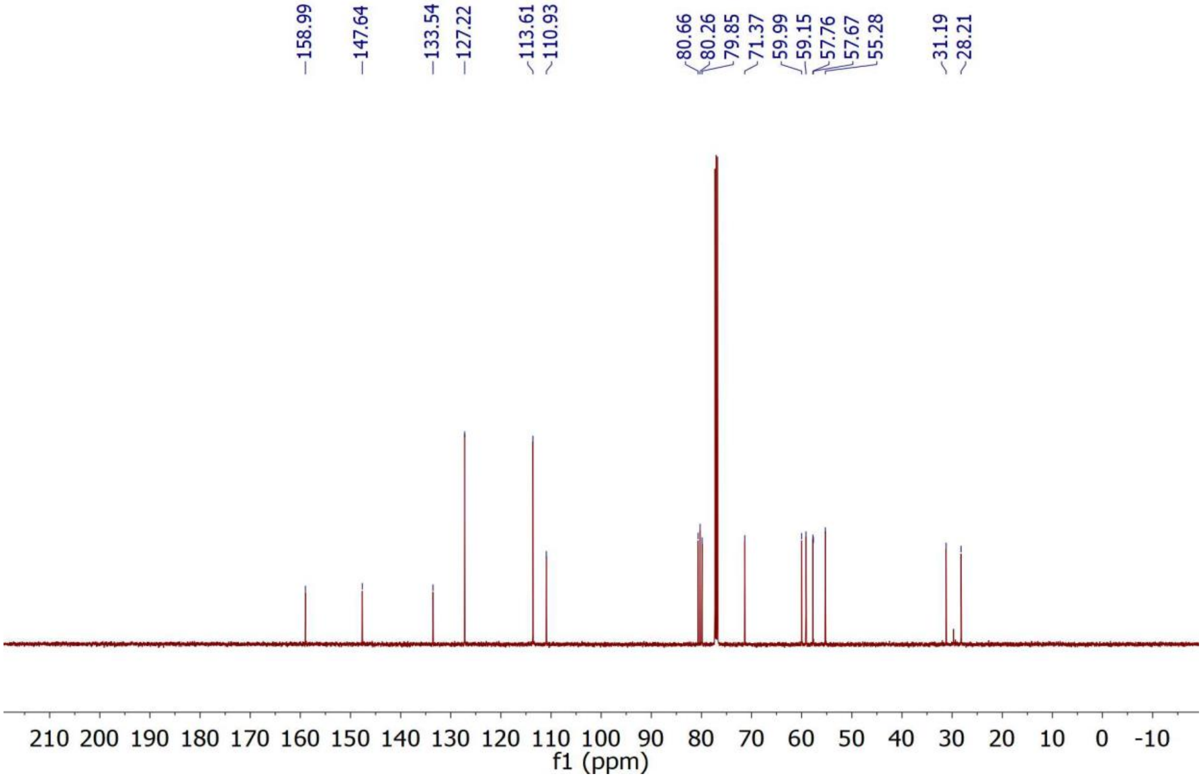

**2,4-dimethyl-1-((5S,6S,7R)-5,6,7,8-tetramethoxyoct-1-en-2-yl)benzene (3bf):**

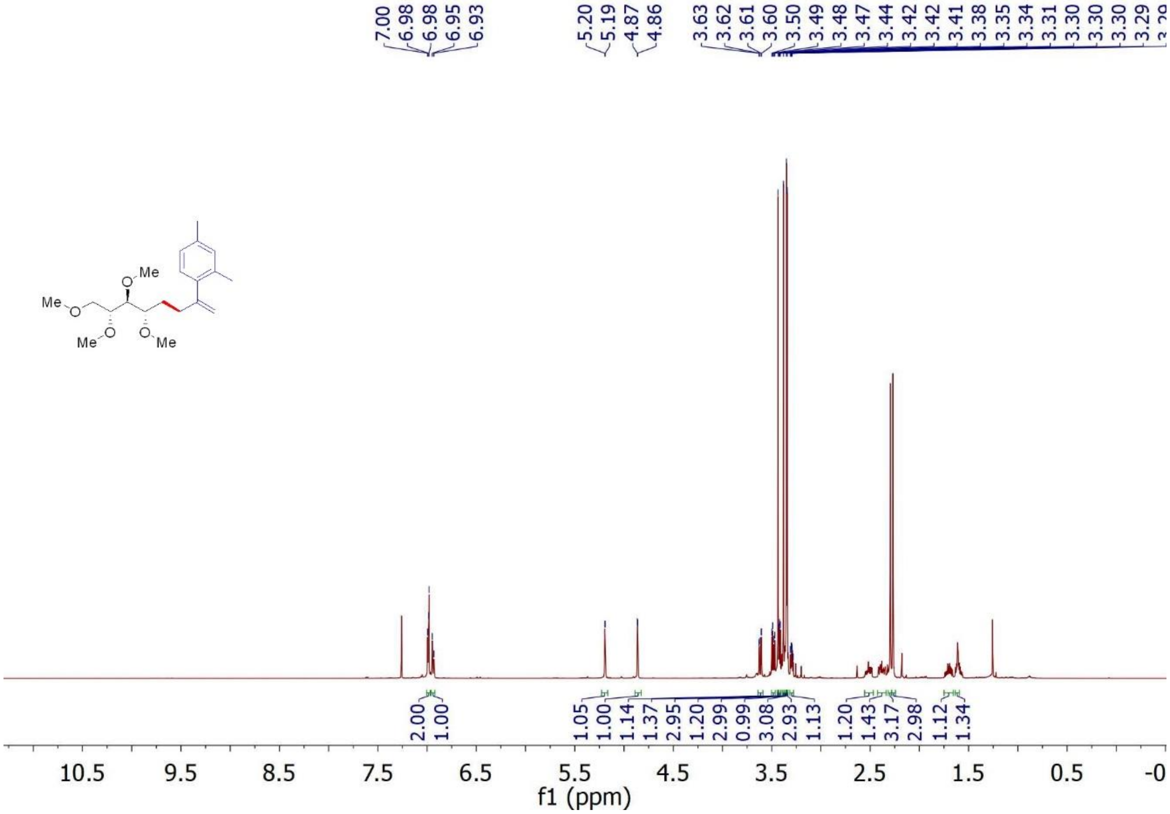

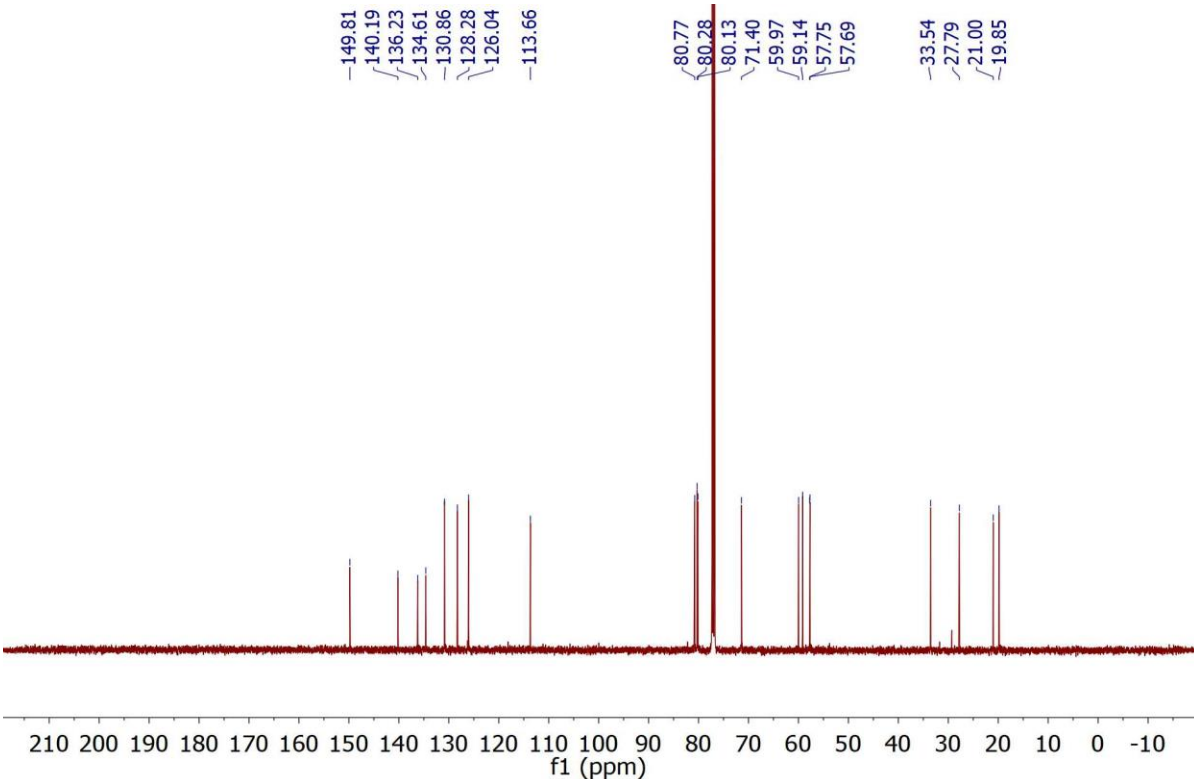

**4-((5*S*,6*S*,7*R*)-5,6,7,8-tetramethoxyoct-1-en-2-yl)benzonitrile (3bg):**

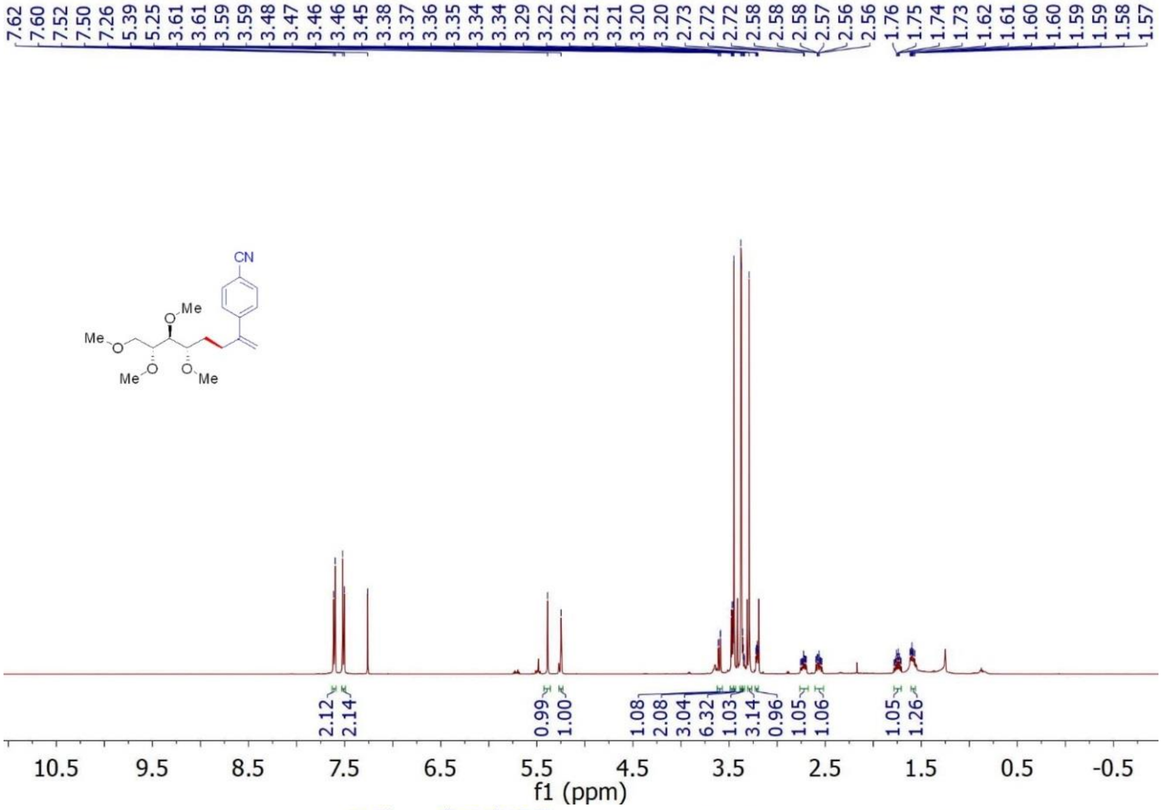


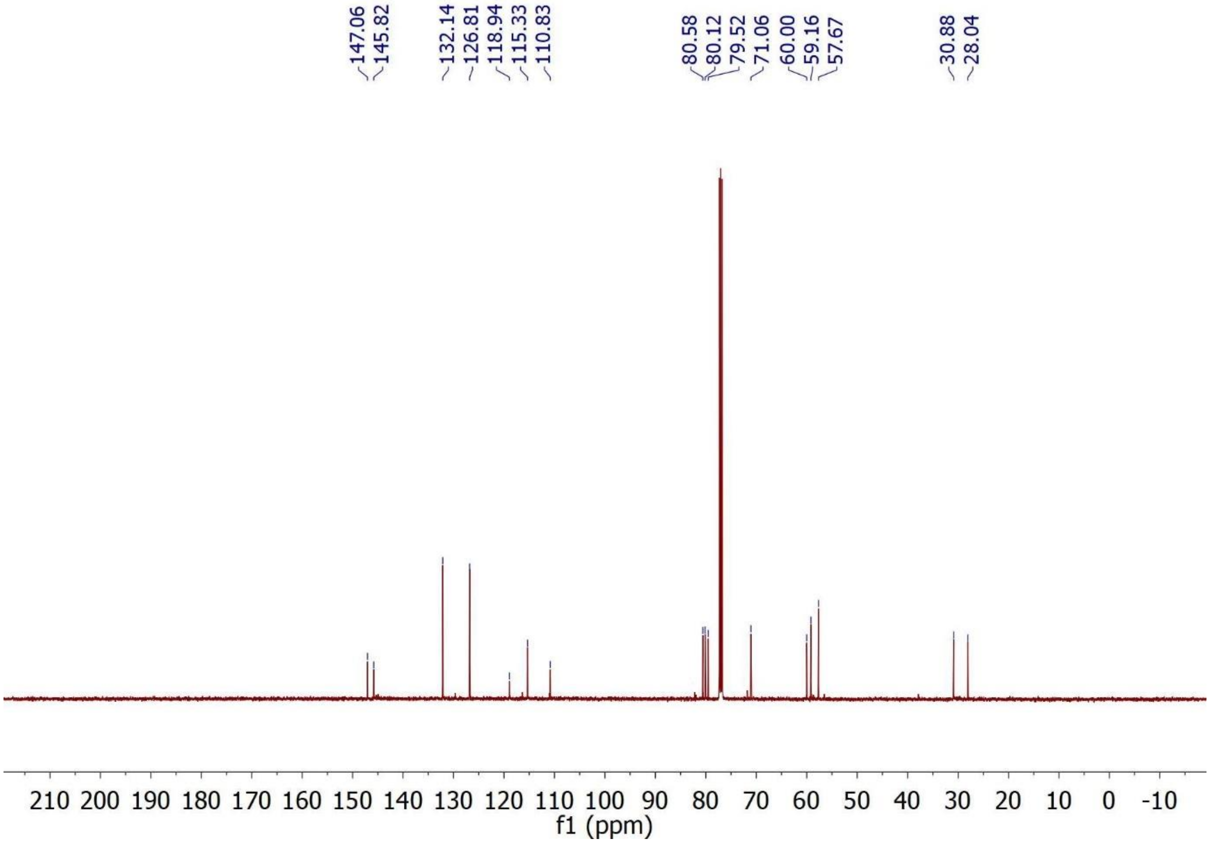

**2,2,2-trifluoro-N-(4-((5*S*,6*S*,7*R*)-5,6,7,8-tetramethoxyoct-1-en-2-yl)phenyl)acetamide (3bh):**

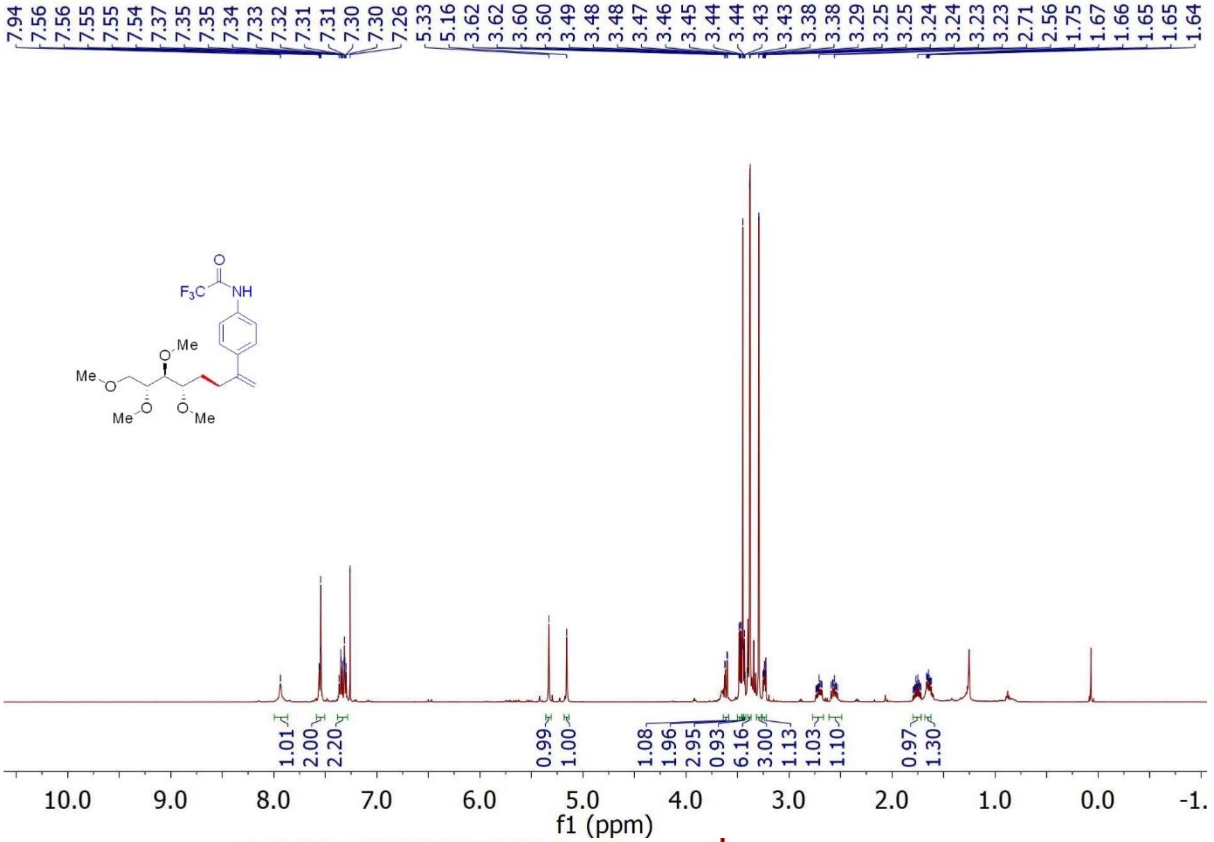


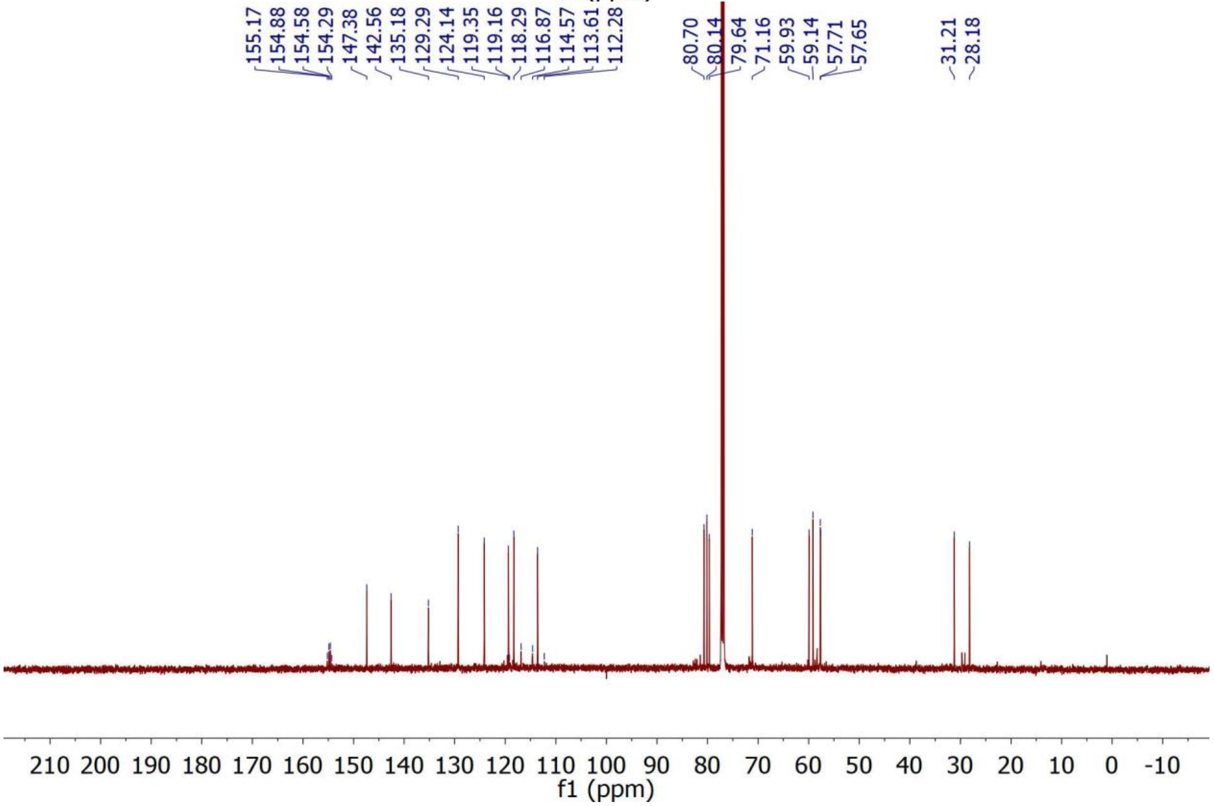

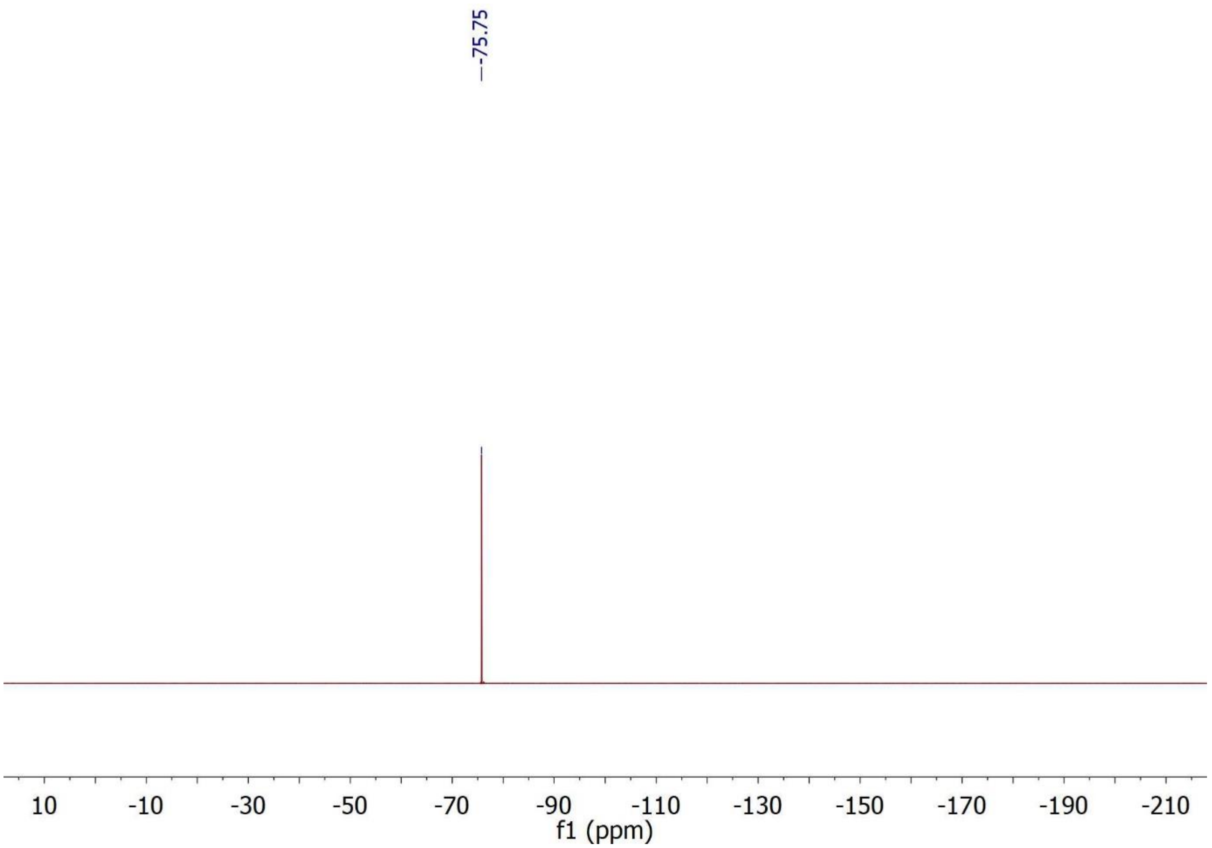


**2-fluoro-4-methoxy-1-((5*S*,6*S*,7*R*)-5,6,7,8-tetramethoxyoct-1-en-2-yl)benzene (3bi):**

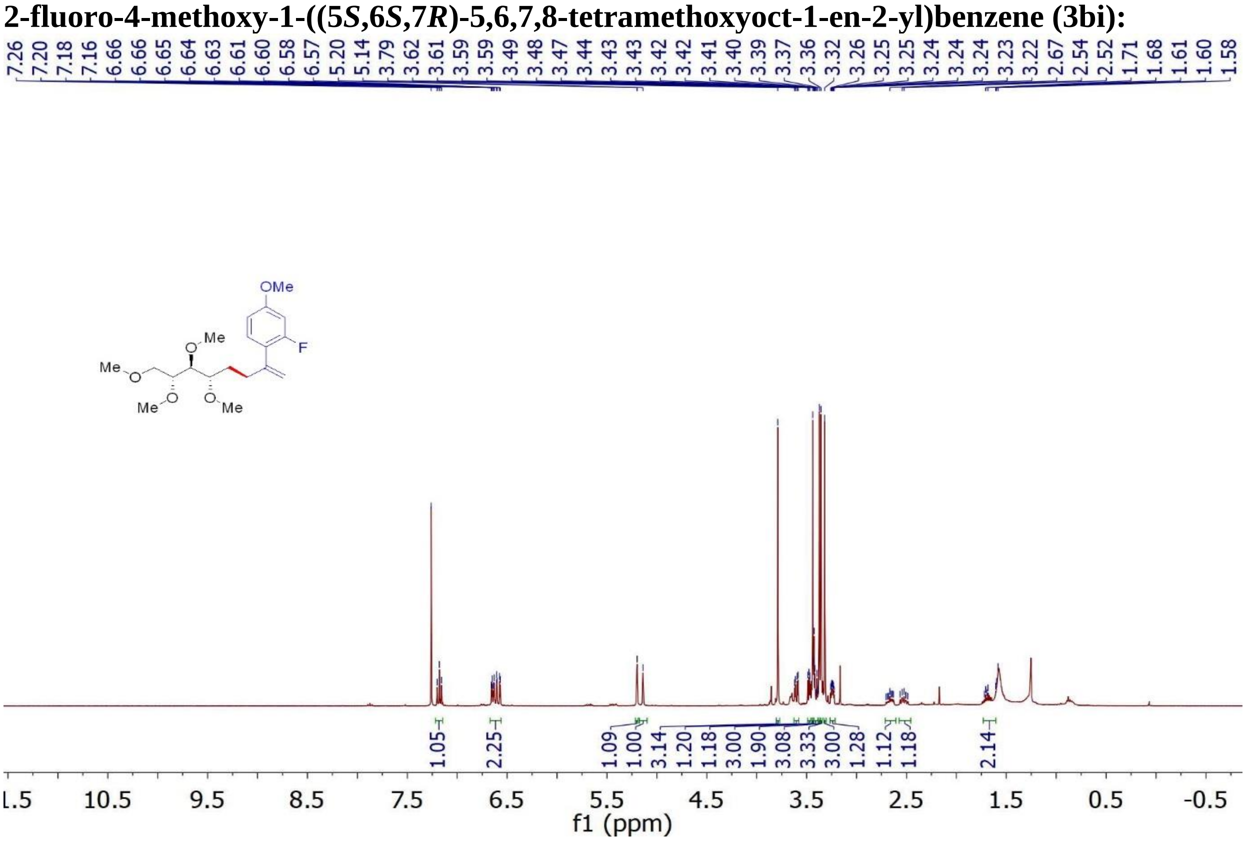

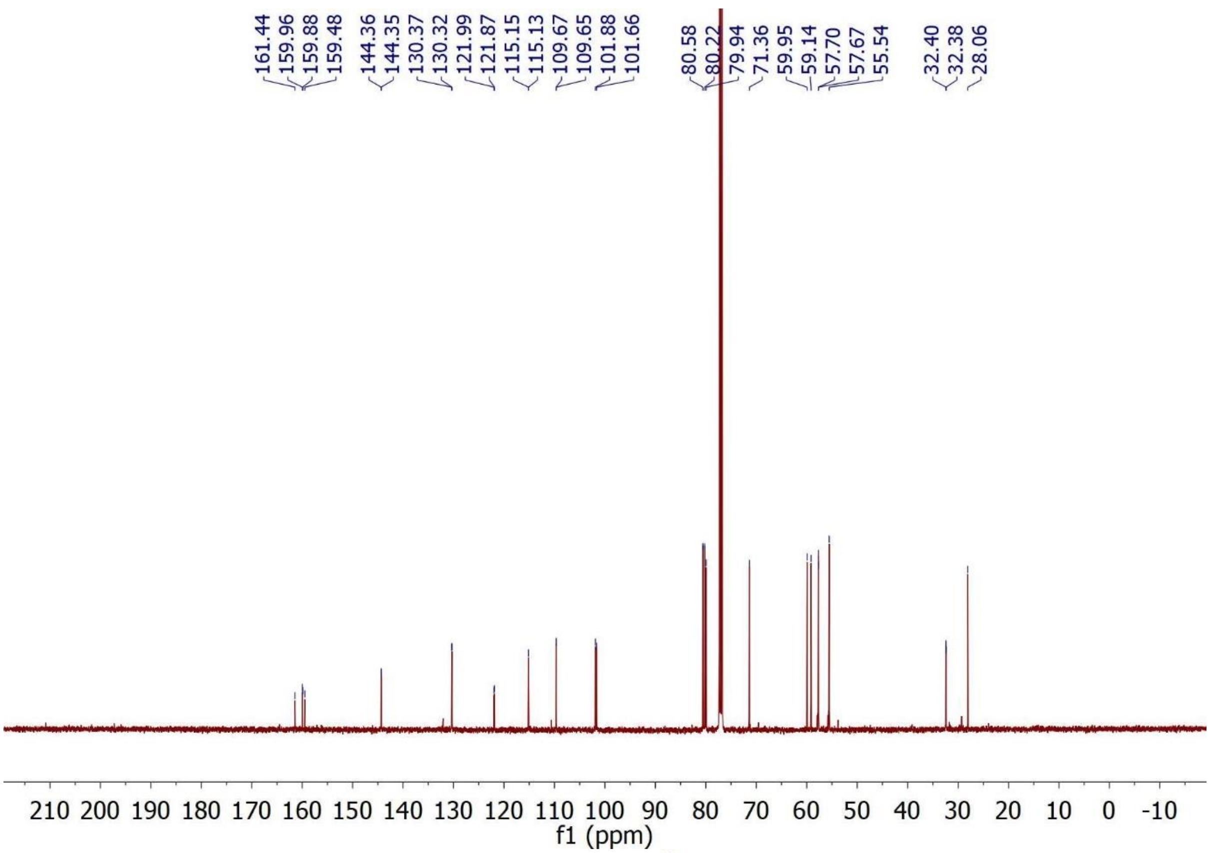
161.44
159.96
159.88
159.48
144.36
144.35
130.37
130.32
121.99
121.87
115.15
115.13
109.67
109.65
101.88
101.66
80.58
80.22
79.94
71.36
59.95
59.14
57.70
57.67
55.54
32.40
32.38
28.06
210 200 190 180 170 160 150 140 130 120 110 100 90 80 70 60 50 40 30 20 10 0 -10
f1 (ppm)

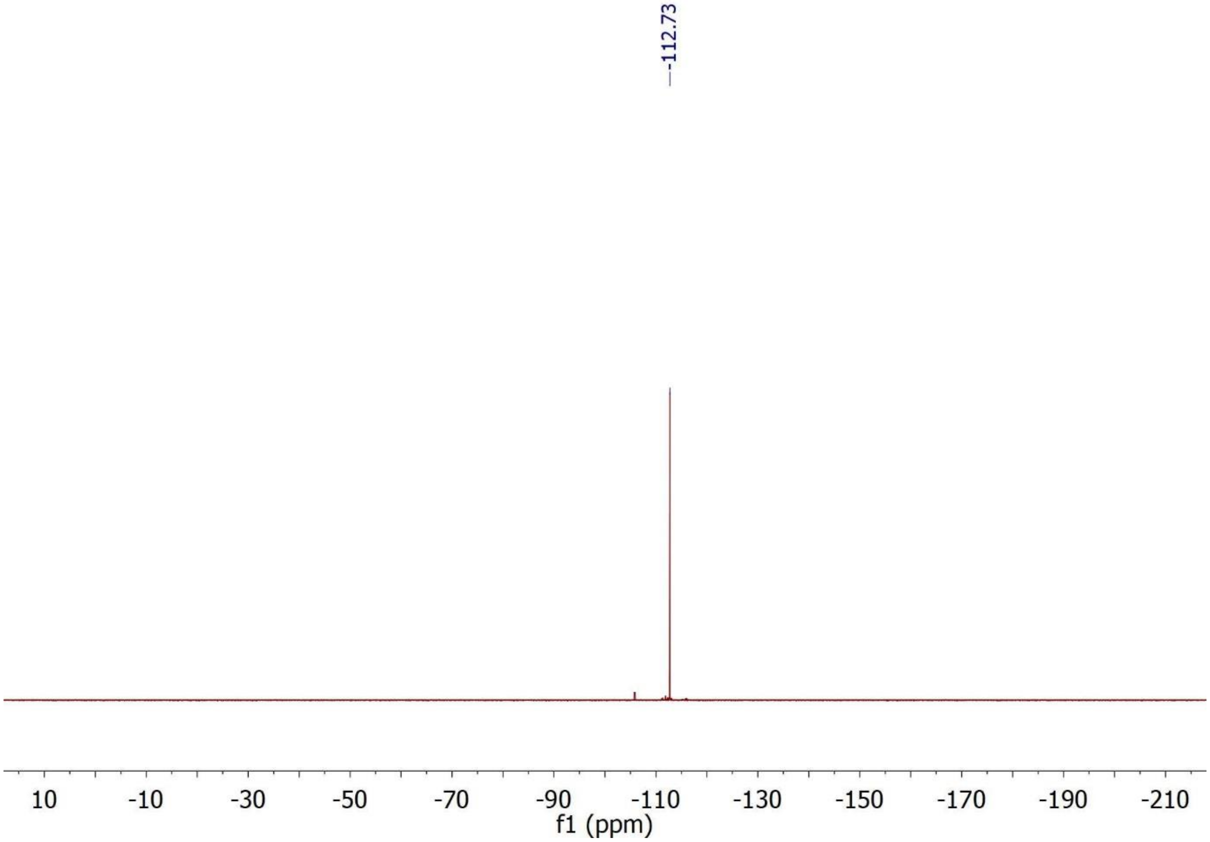
-112.73
10 -10 -30 -50 -70 -90 -110 -130 -150 -170 -190 -210
f1 (ppm)

**(4R,4'S,5S)-2,2,2',2'-tetramethyl-5-(3-phenylbut-3-en-1-yl)-4,4'-bi(1,3-dioxolane) (3bj):**

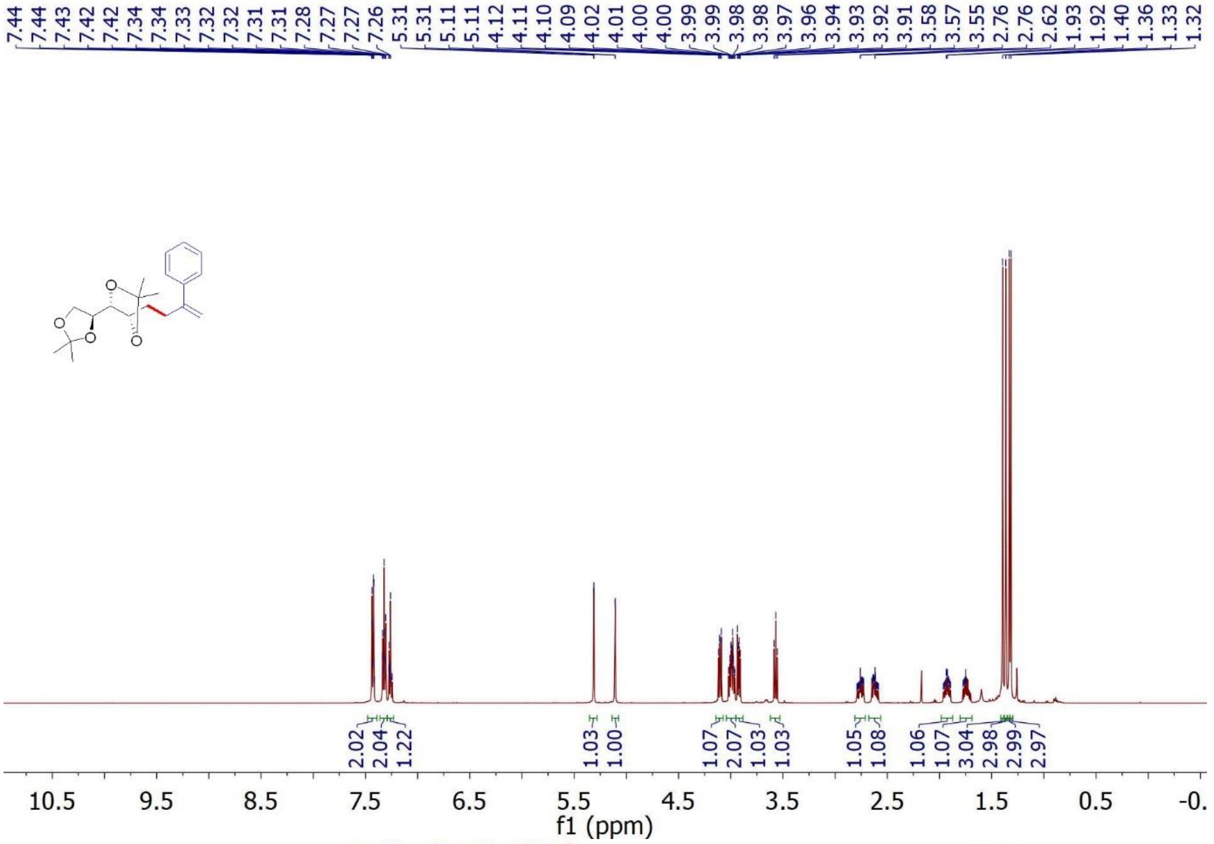


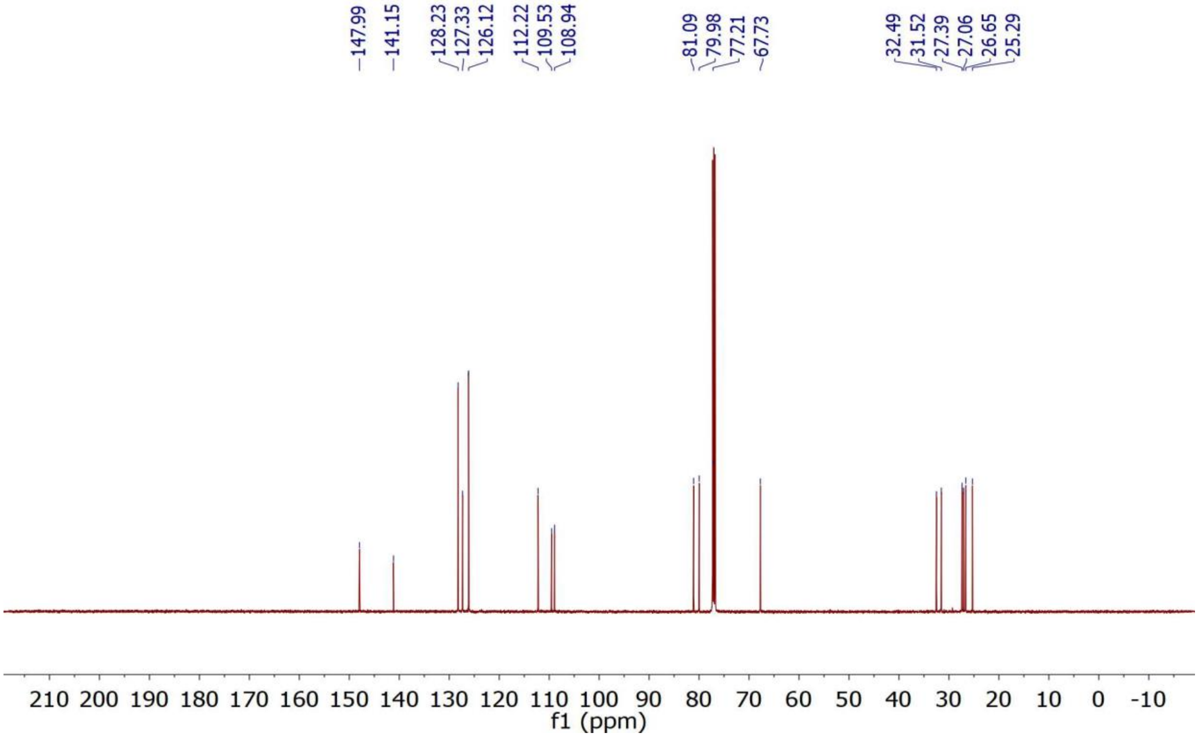


**(4R,4'S,5S)-2,2,2',2'-tetramethyl-5-(3-(4-(trifluoromethyl)phenyl)but-3-en-1-yl)-4,4'-bi(1,3-dioxolane) (3bk):**

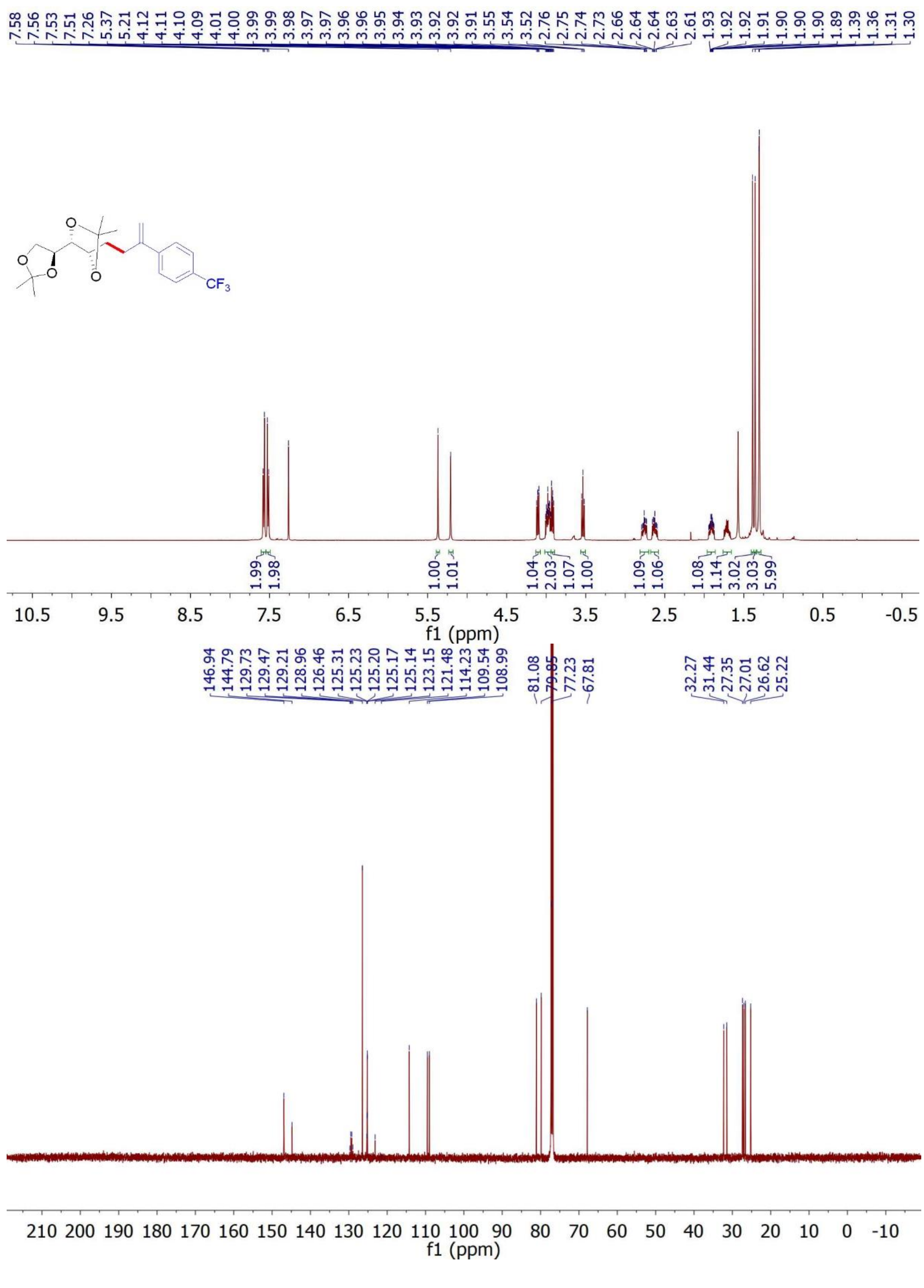

7.58 7.56 7.53 7.51 7.26 5.37 5.21 4.12 4.11 4.10 4.09 4.01 4.00 3.99 3.99 3.98 3.97 3.97 3.96 3.96 3.95 3.94 3.93 3.92 3.92 3.91 3.55 3.54 3.52 2.76 2.75 2.74 2.73 2.66 2.64 2.64 2.63 2.61 1.93 1.92 1.92 1.91 1.90 1.90 1.90 1.89 1.39 1.36 1.31 1.30
CF3
1.99 1.98 1.00 1.01 1.04 2.03 1.07 1.00 1.09 1.06 1.08 1.14 3.02 3.03 5.99
10.5 9.5 8.5 7.5 6.5 5.5 4.5 3.5 2.5 1.5 0.5 -0.5
f1 (ppm)
146.94 144.79 129.73 129.47 129.21 128.96 126.46 125.31 125.23 125.20 125.17 125.14 123.15 121.48 114.23 109.54 108.99
81.08 79.85 77.23 67.81
32.27 31.44 27.35 27.01 26.62 25.22
210 200 190 180 170 160 150 140 130 120 110 100 90 80 70 60 50 40 30 20 10 0 -10
f1 (ppm)

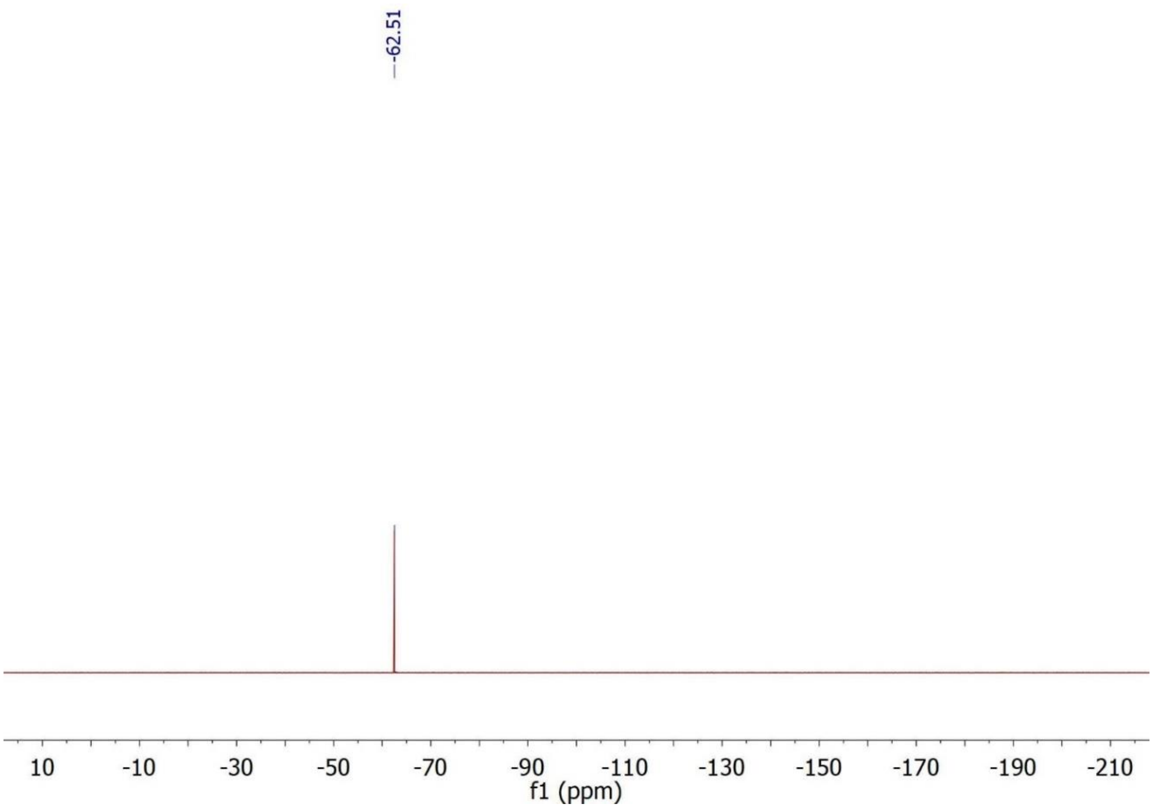


**(4*R*,4'*S*,5*S*)-2,2,2',2'-tetramethyl-5-(3-(3-(trifluoromethyl)phenyl)but-3-en-1-yl)-4,4'-bi(1,3-dioxolane) (3bl):**

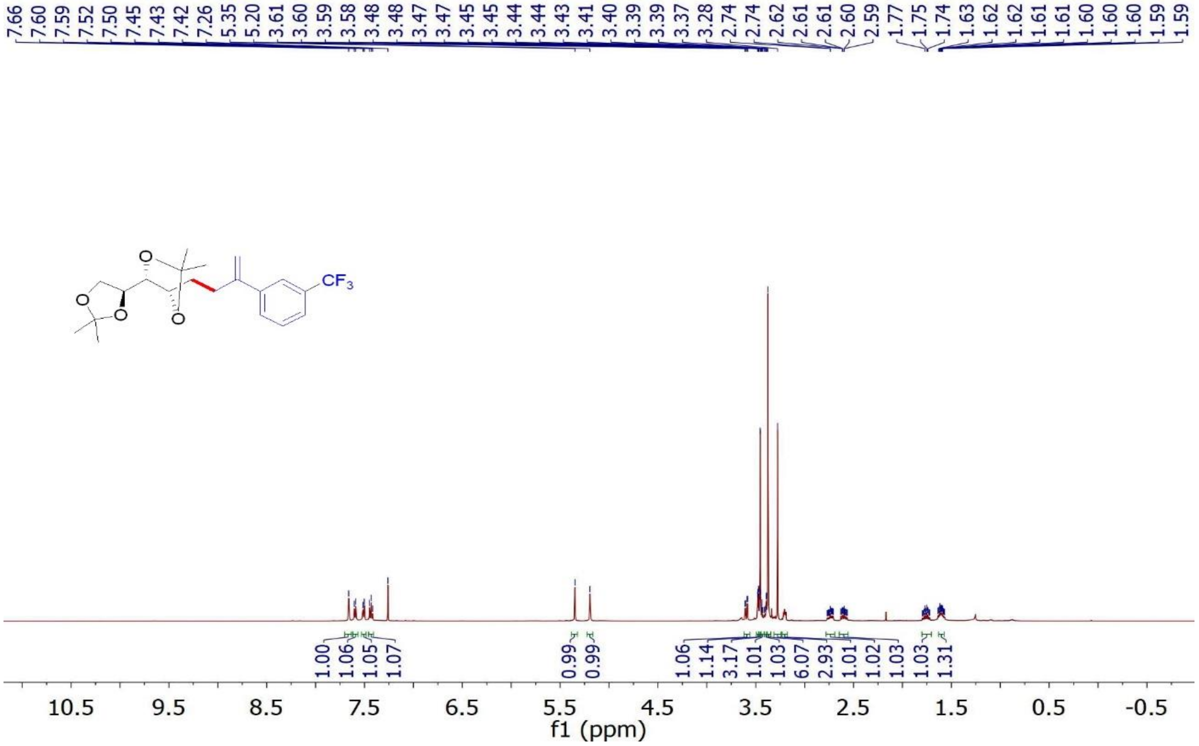

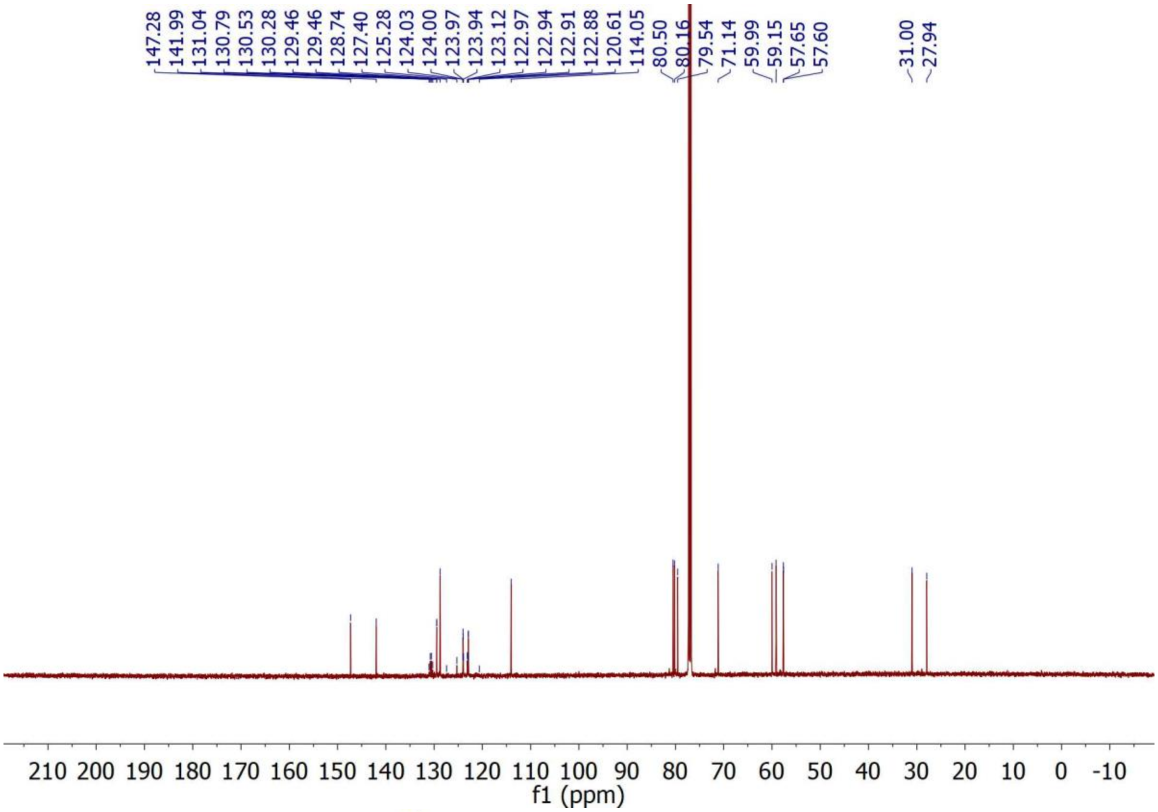


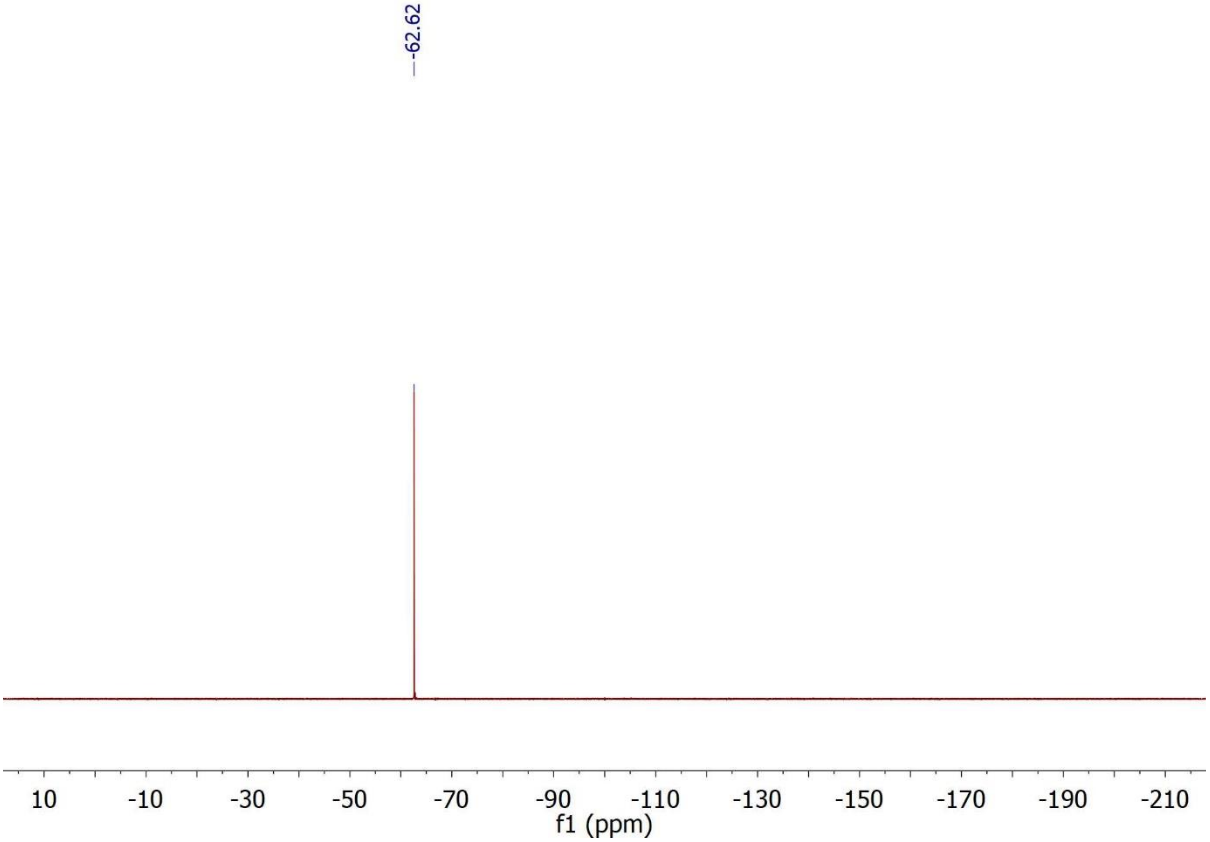


**(4R,4'S,5S)-5-(3-benzylbut-3-en-1-yl)-2,2,2',2'-tetramethyl-4,4'-bi(1,3-dioxolane) (3bm):**

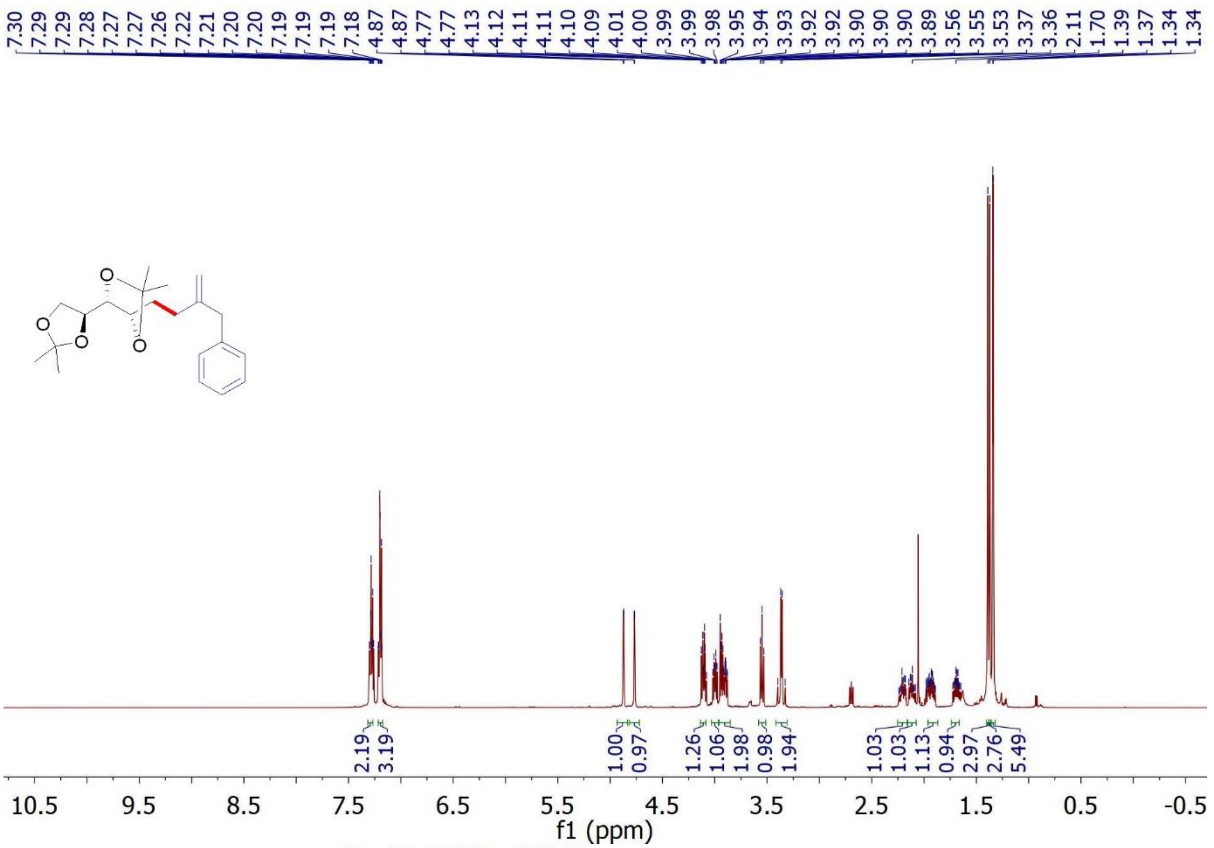


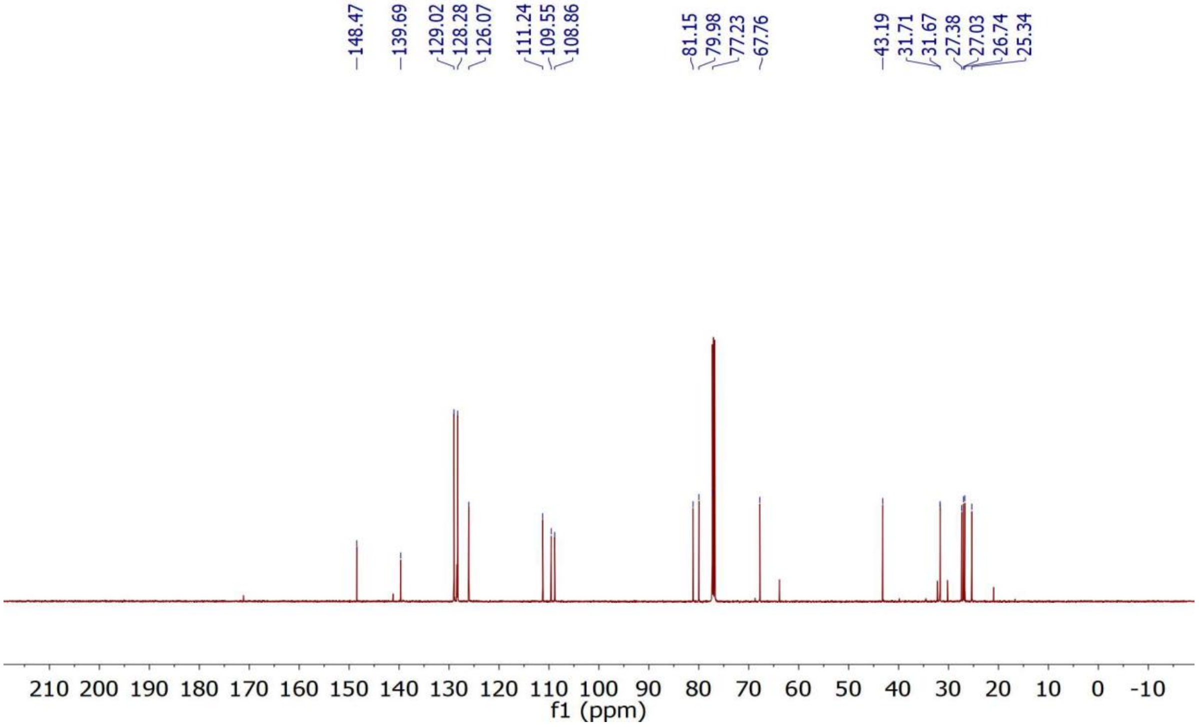


**(3aR,5R,5aS,8aS,8bR)-2,2,7,7-tetramethyl-5-(3-phenylbut-3-en-1-yl)tetrahydro-5H-bis([1,3]dioxolo)[4,5-b:4',5'-d]pyran (3bn):**

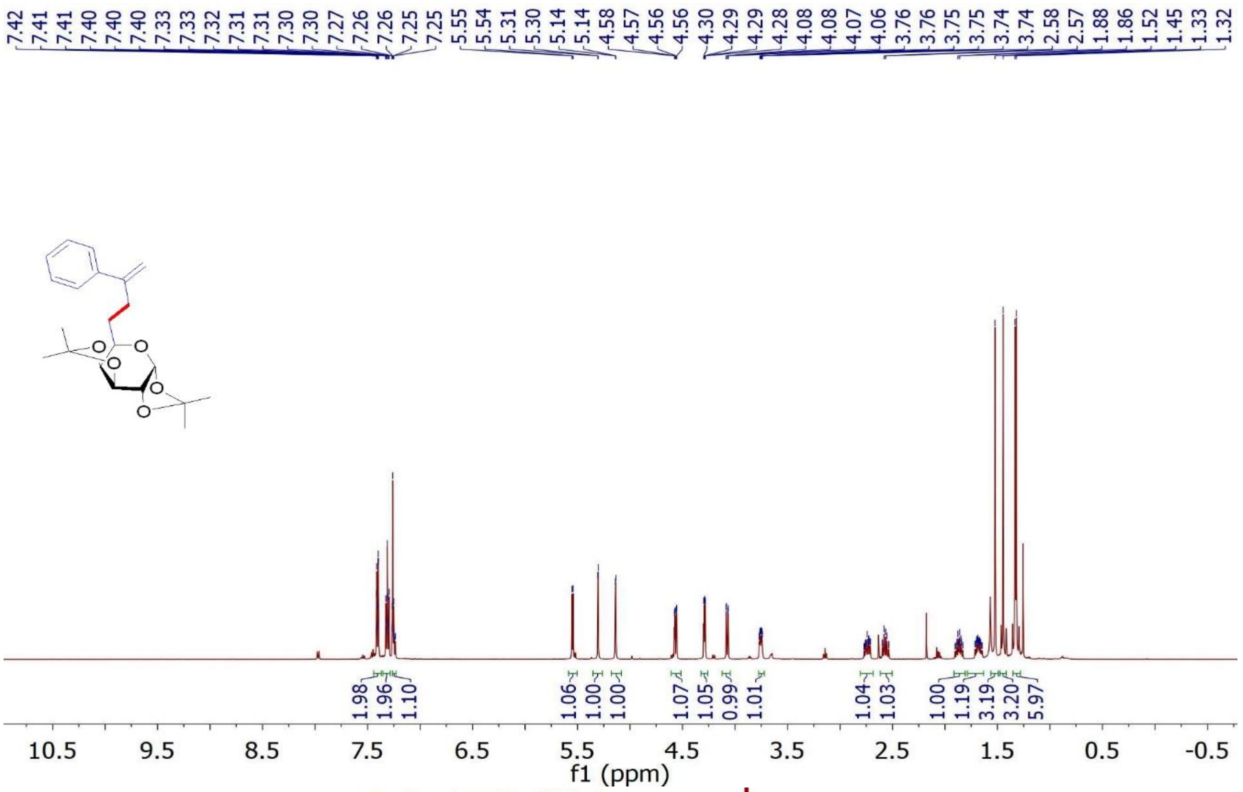


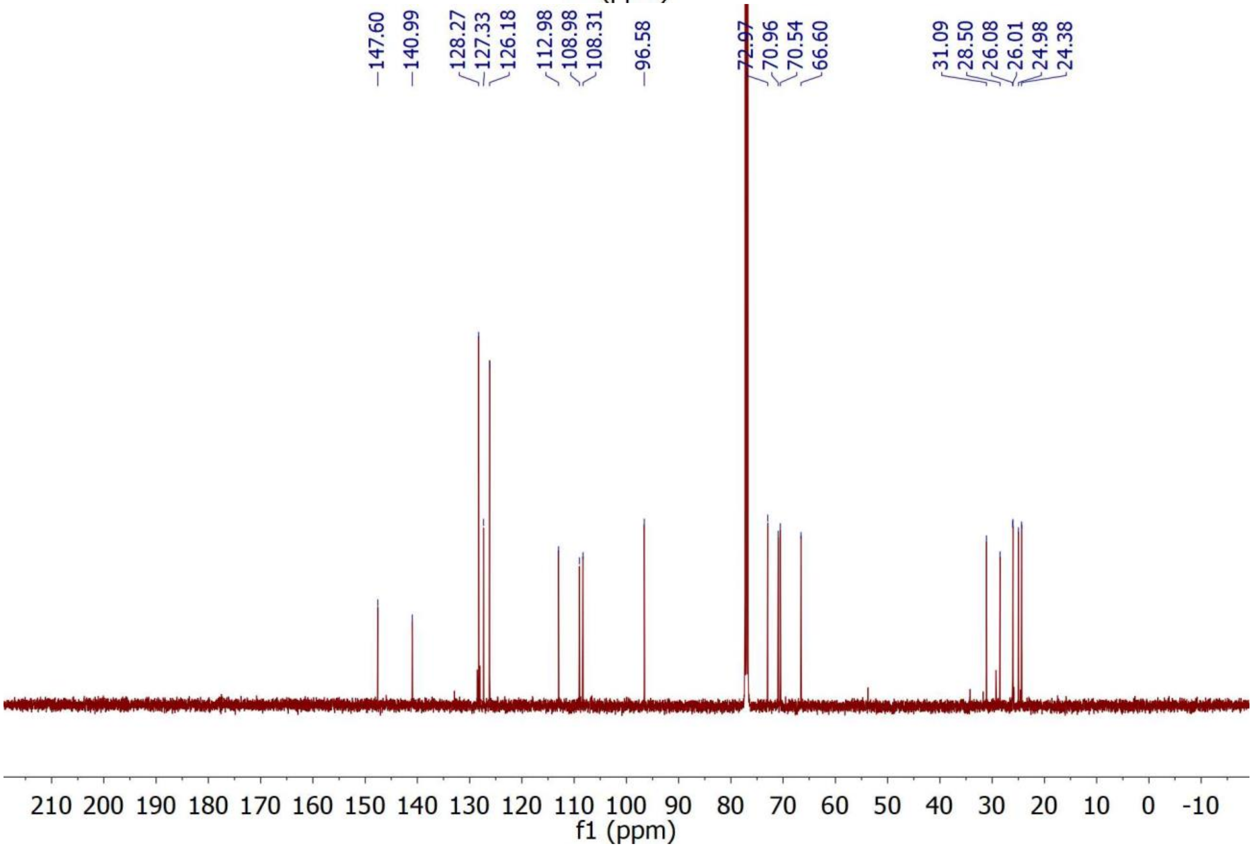


**(4R,4'S,5S)-2,2,2',2'-tetramethyl-5-(3-(thiophen-2-yl)but-3-en-1-yl)-4,4'-bi(1,3-dioxolane) (3bo):**

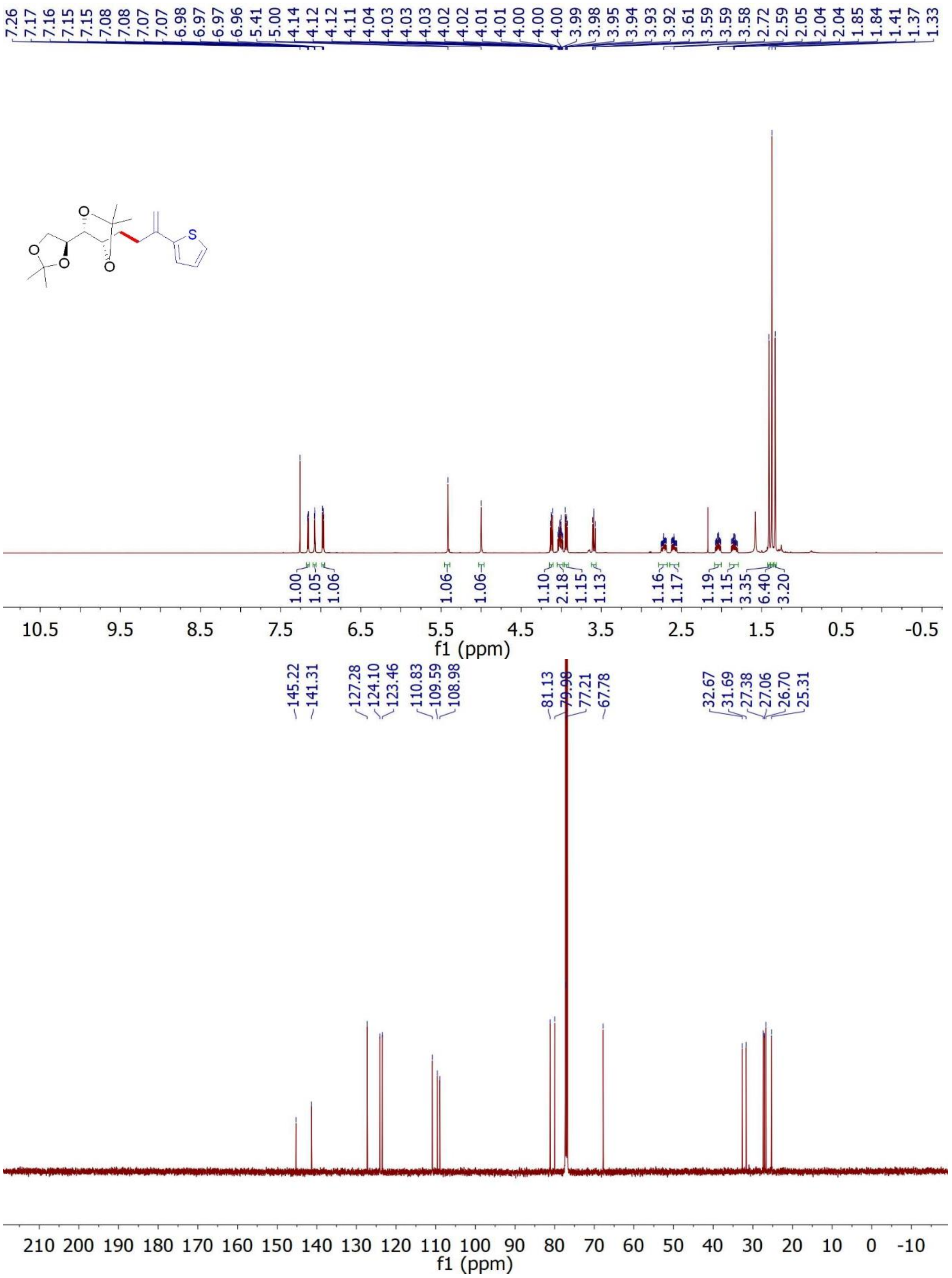
7.26 7.17 7.16 7.15 7.15 7.08 7.08 7.07 7.07 6.98 6.97 6.96 5.41 5.00 4.14 4.12 4.12 4.11 4.04 4.03 4.03 4.03 4.02 4.02 4.01 4.01 4.00 4.00 4.00 3.99 3.98 3.95 3.94 3.93 3.92 3.61 3.59 3.59 3.58 2.72 2.59 2.05 2.04 1.85 1.84 1.41 1.37 1.33
S
O
1.00 1.05 1.06 1.06 1.06 1.10 2.18 1.15 1.13 1.16 1.17 1.19 1.15 3.35 6.40 3.20
10.5 9.5 8.5 7.5 6.5 5.5 4.5 3.5 2.5 1.5 0.5 -0.5
f1 (ppm)
145.22 141.31 127.28 124.10 123.46 110.83 109.59 108.98 81.13 79.98 77.21 67.78 32.67 31.69 27.38 27.06 26.70 25.31
210 200 190 180 170 160 150 140 130 120 110 100 90 80 70 60 50 40 30 20 10 0 -10
f1 (ppm)

**Triisopropyl(3-methylene-5-((4*R*,4'*S*,5*S*)-2,2,2',2'-tetramethyl-[4,4'-bi(1,3-dioxolan)]-5-yl)pent-1-yn-1-yl)silane (3bp):**

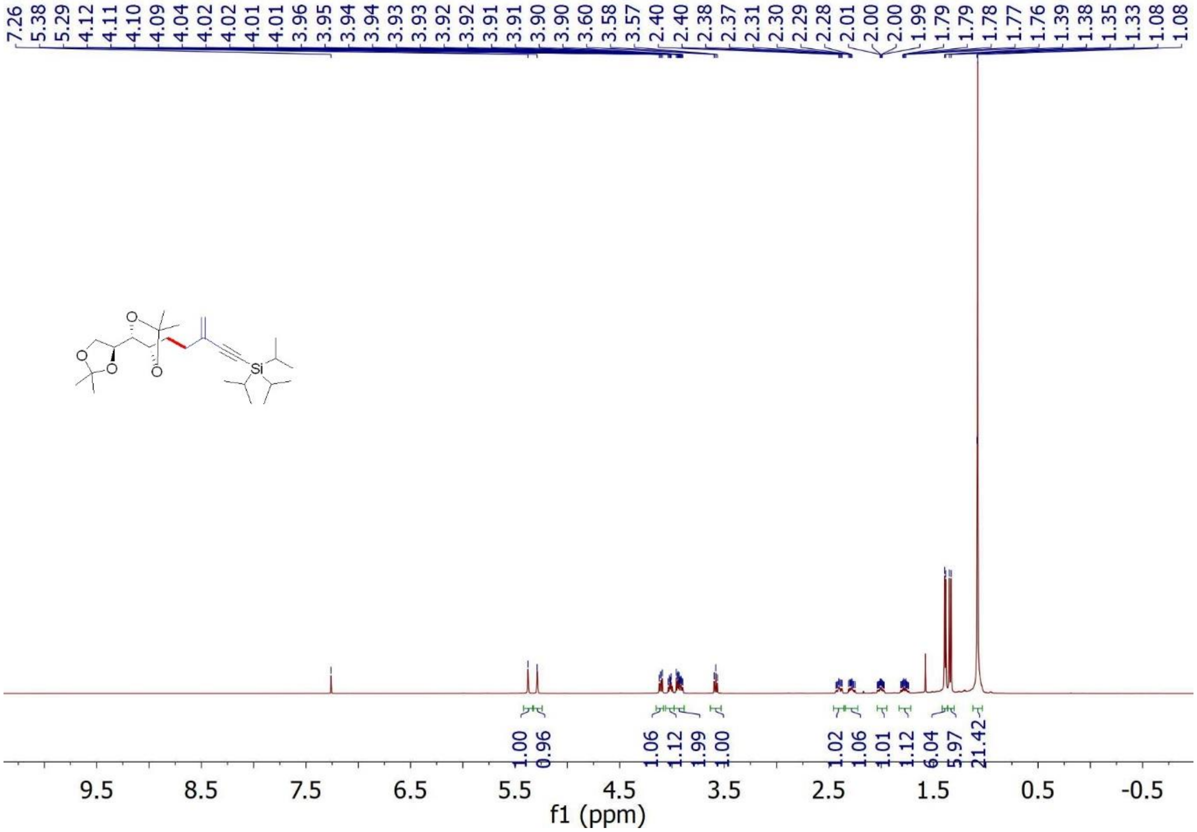


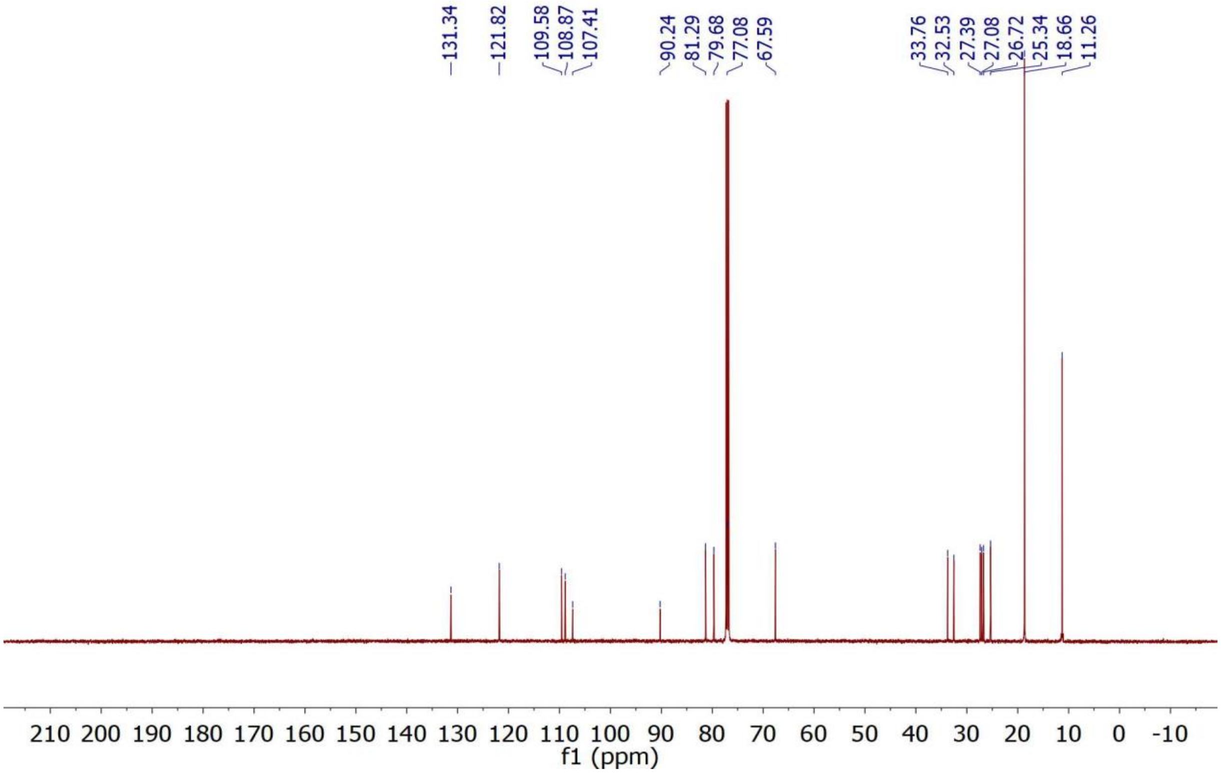

**triisopropyl(3-methylene-5-((3aR,5R,5aS,8aS,8bR)-2,2,7,7-tetramethyltetrahydro-5H-bis([1,3]dioxolo)[4,5-b:4',5'-d]pyran-5-yl)pent-1-yn-1-yl)silane (3bq):**

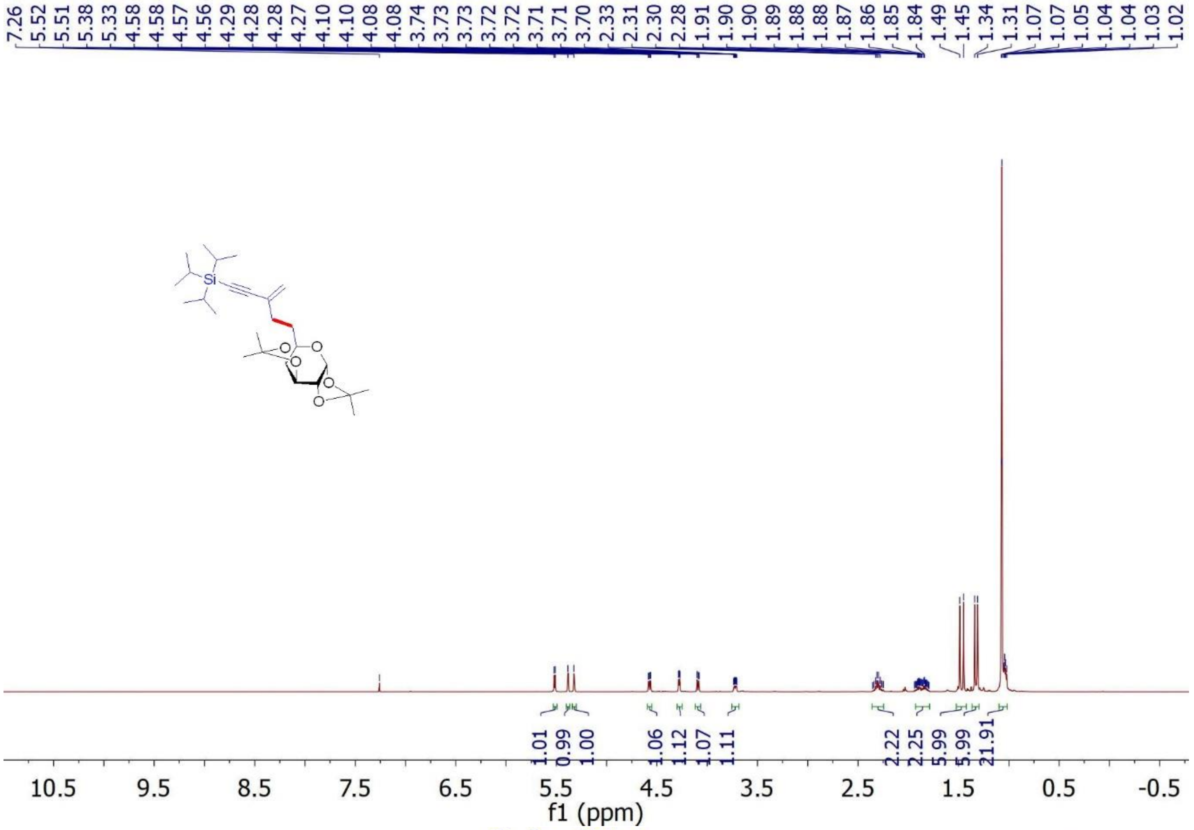


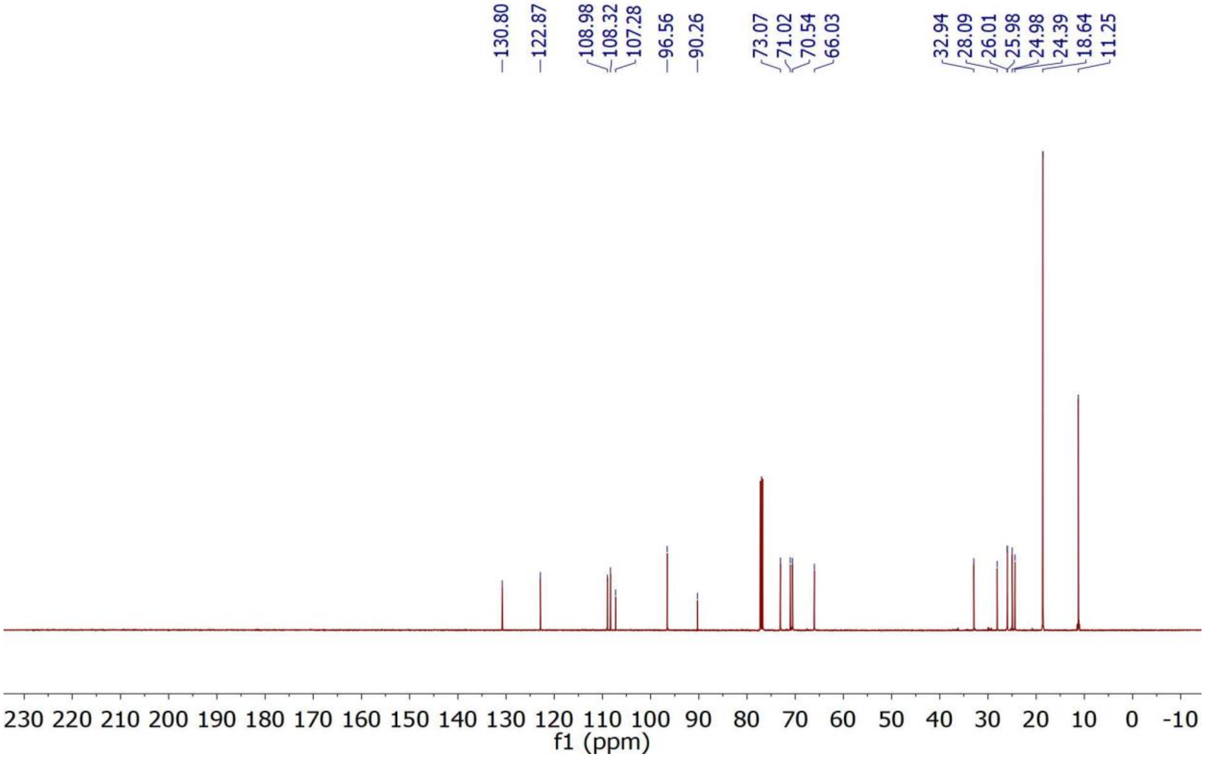


**(*E*)-4-phenylhexa-2,5-dien-1-ol (3br):**

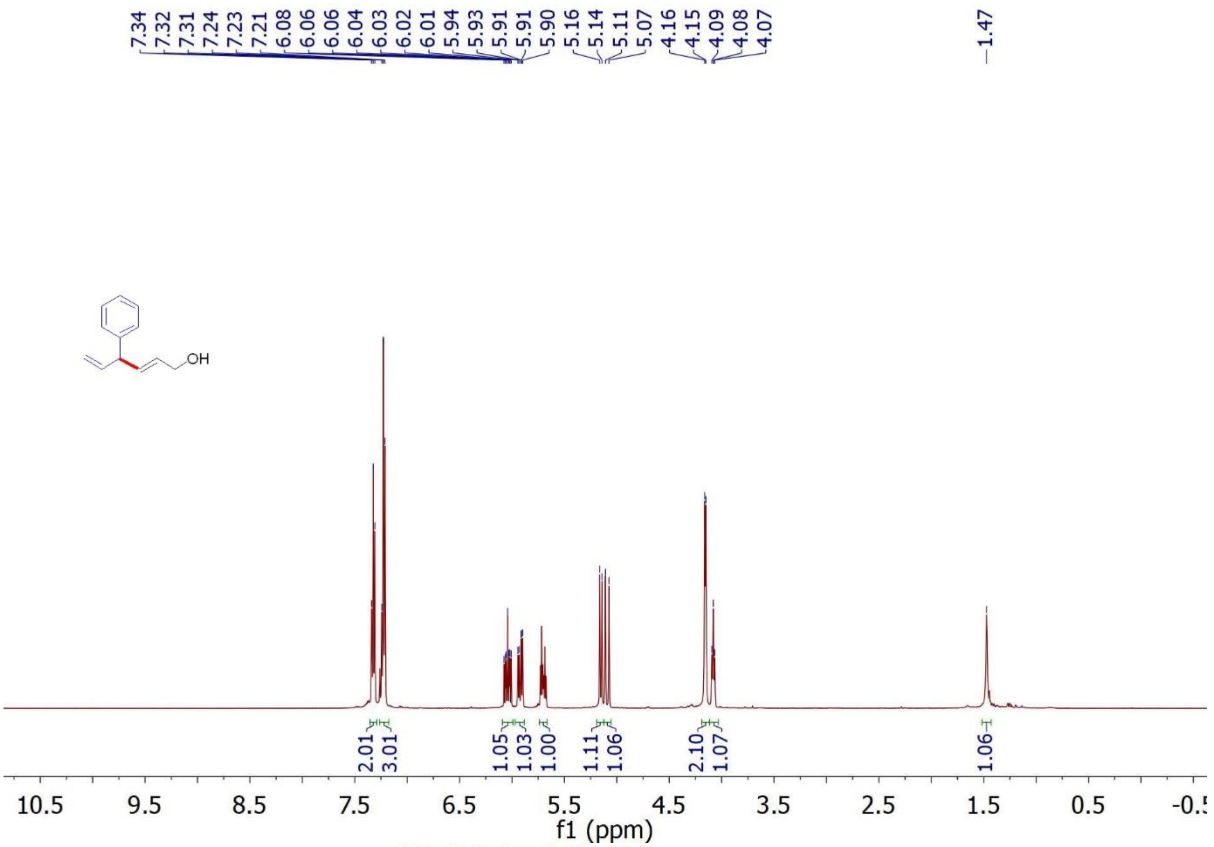


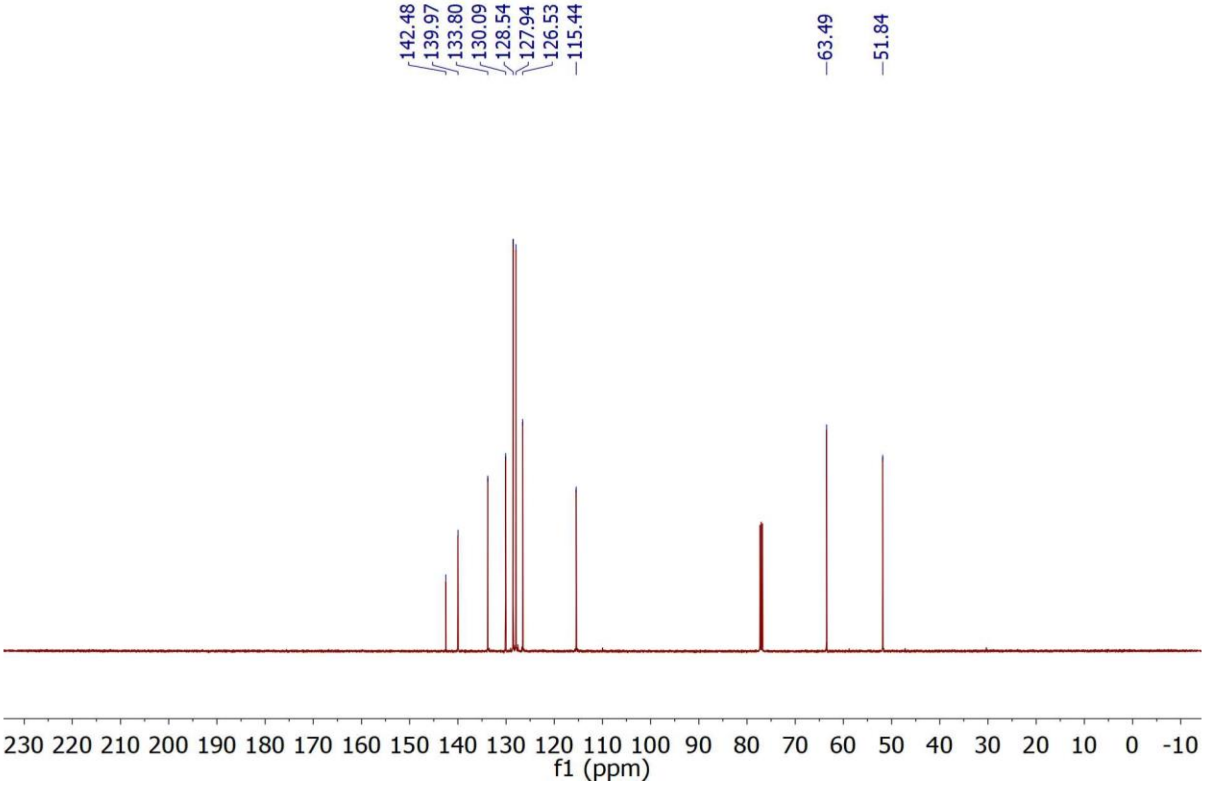


**(*E*)-4-(3-chlorophenyl)hexa-2,5-dien-1-ol (3bs)：**

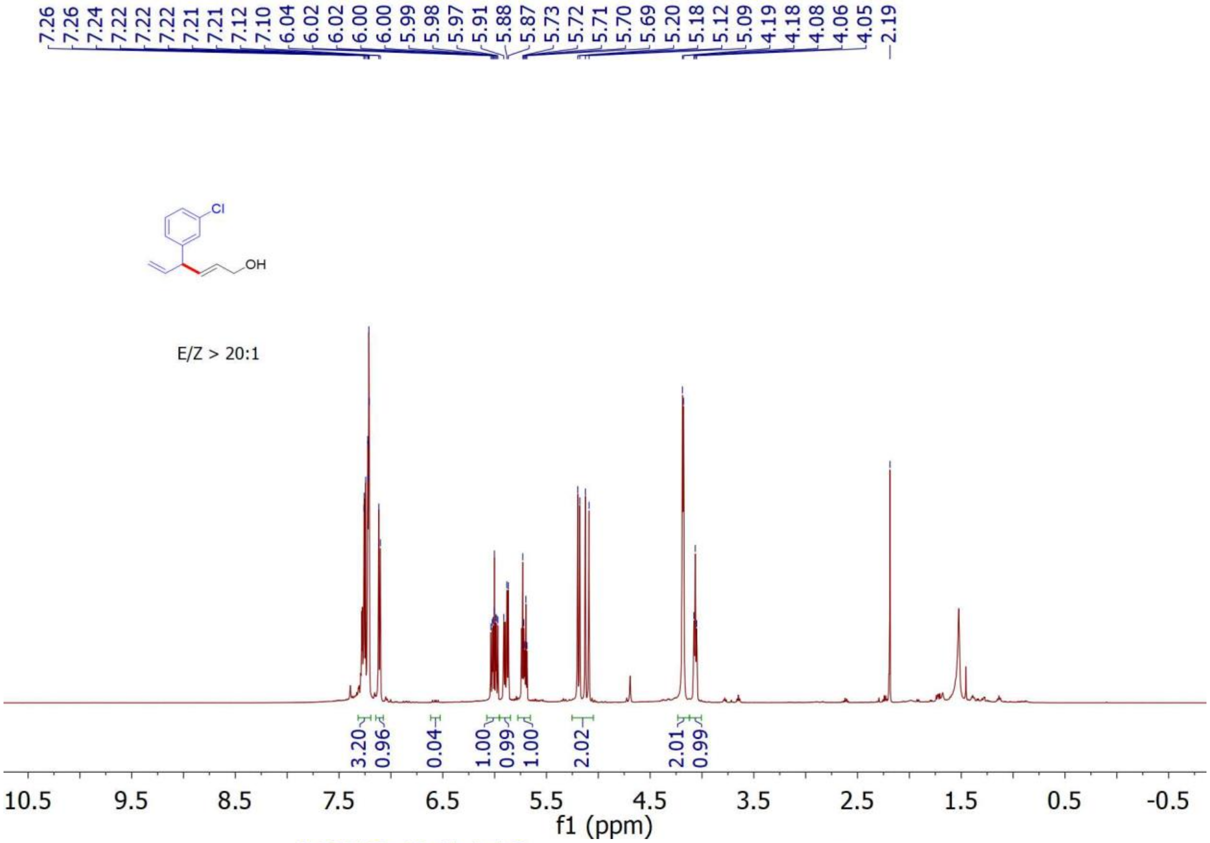


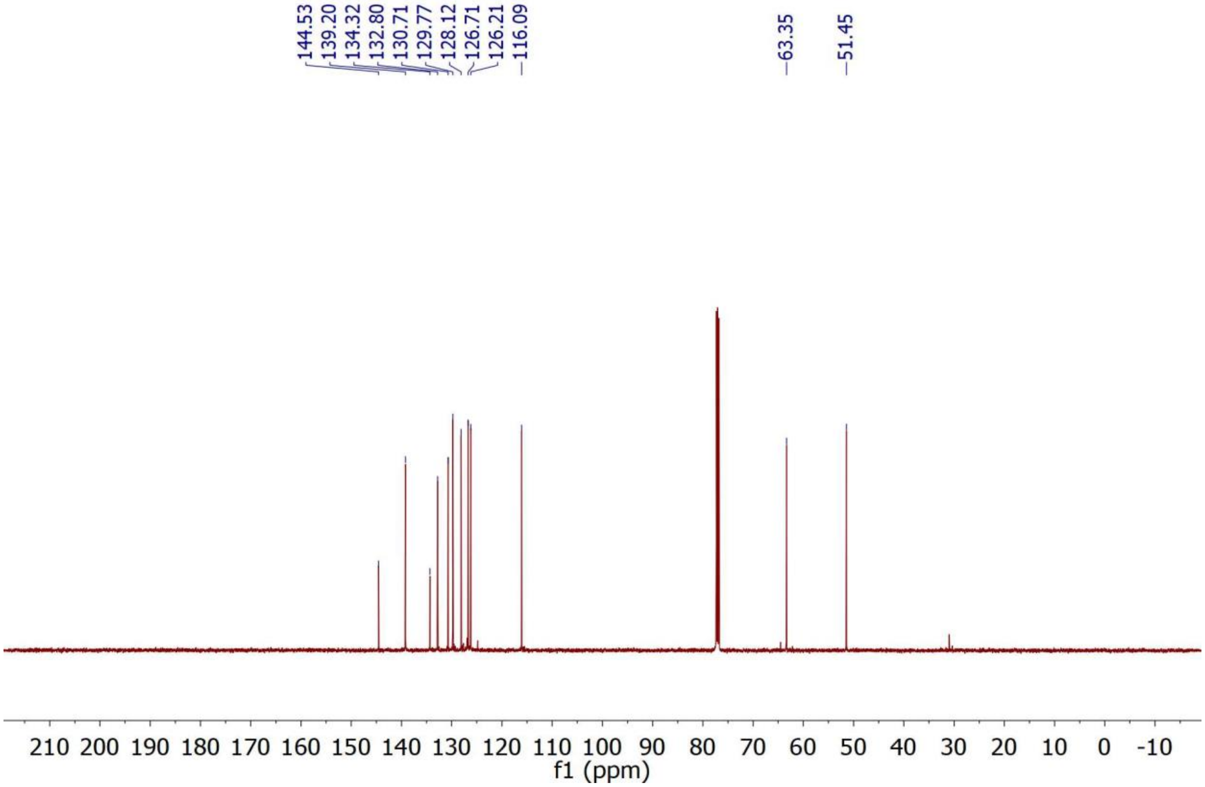


**(*E*)-4-(3-(trifluoromethyl)phenyl)hexa-2,5-dien-1-ol (3bt):**

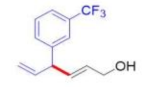

CF3
OH


E/Z = 20:1

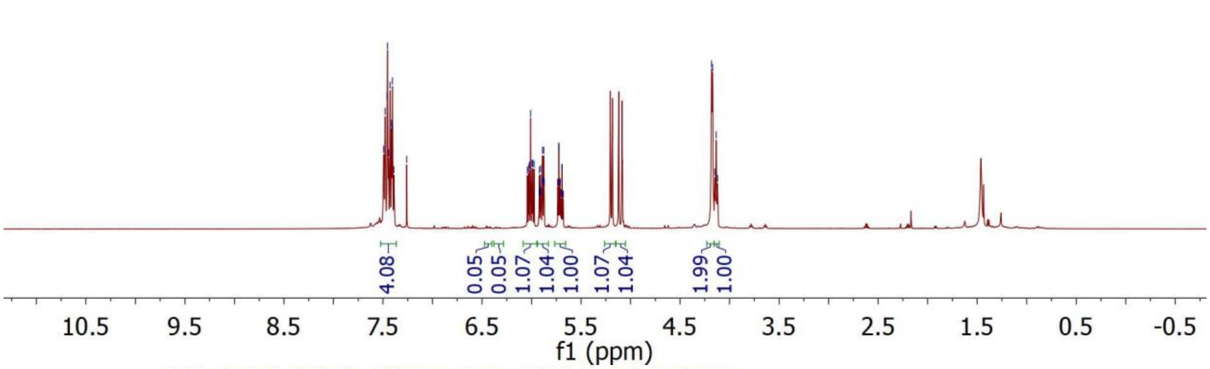

7.49 7.48 7.46 7.45 7.44 7.43 7.41 7.41 7.40 7.39 7.26
6.04 6.03 6.02 6.01 5.99 5.99 5.97 5.92 5.91 5.91 5.90 5.89 5.89 5.89 5.88 5.88 5.87 5.74 5.73 5.72 5.72 5.71 5.71 5.69 5.69 4.18 4.17 4.15 4.14
4.08
0.05 0.05 1.07 1.04 1.00 1.07 1.04
1.99 1.00
10.5 9.5 8.5 7.5 6.5 5.5 4.5 3.5 2.5 1.5 0.5 -0.5
f1 (ppm)


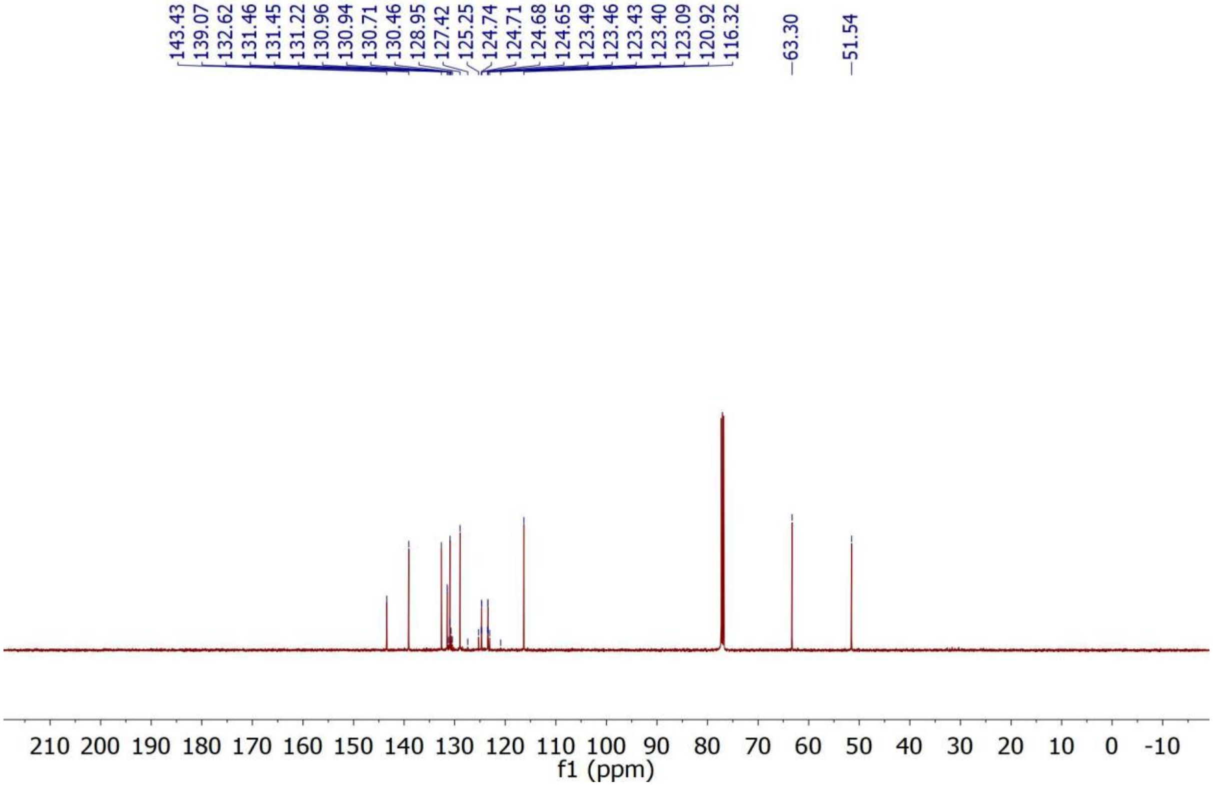

143.43 139.07 132.62 131.46 131.45 131.22 130.96 130.94 130.71 130.46 128.95 127.42 125.25 124.74 124.71 124.68 124.65 123.49 123.46 123.43 123.40 123.09 120.92 116.32
63.30
51.54
210 200 190 180 170 160 150 140 130 120 110 100 90 80 70 60 50 40 30 20 10 0 -10
f1 (ppm)

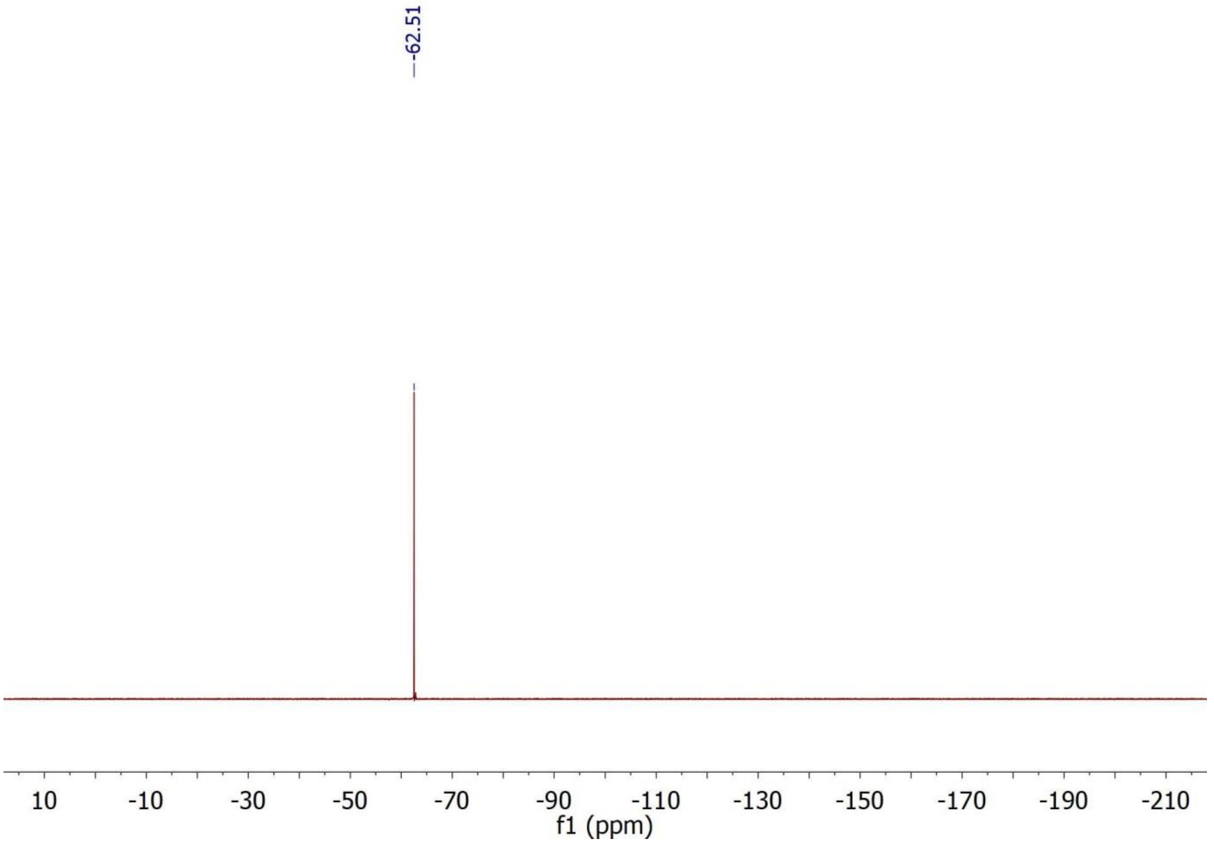


**(*E*)-4-(3-(trifluoromethyl)phenyl)hexa-2,5-dien-1-ol (3bu)**:

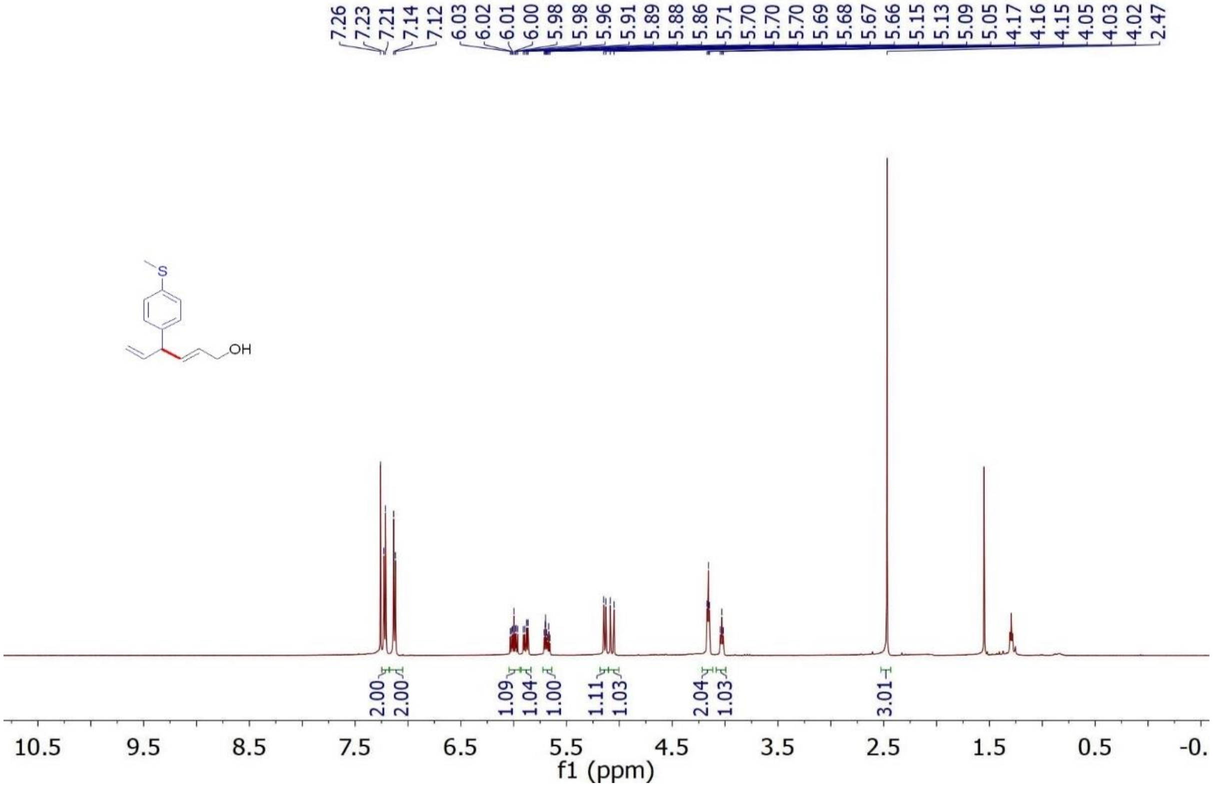

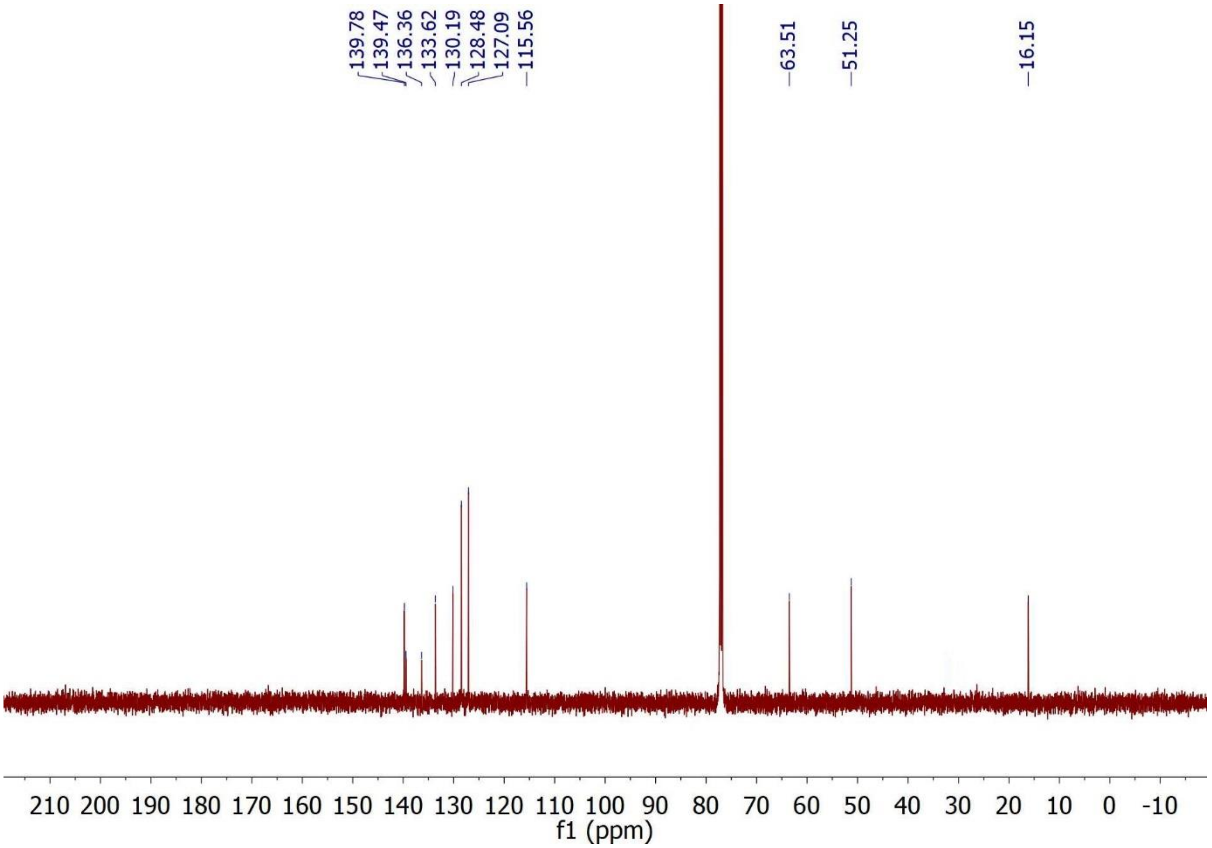


**(*E*)-4-(thiophen-3-yl)hexa-2,5-dien-1-ol (3bv)：**

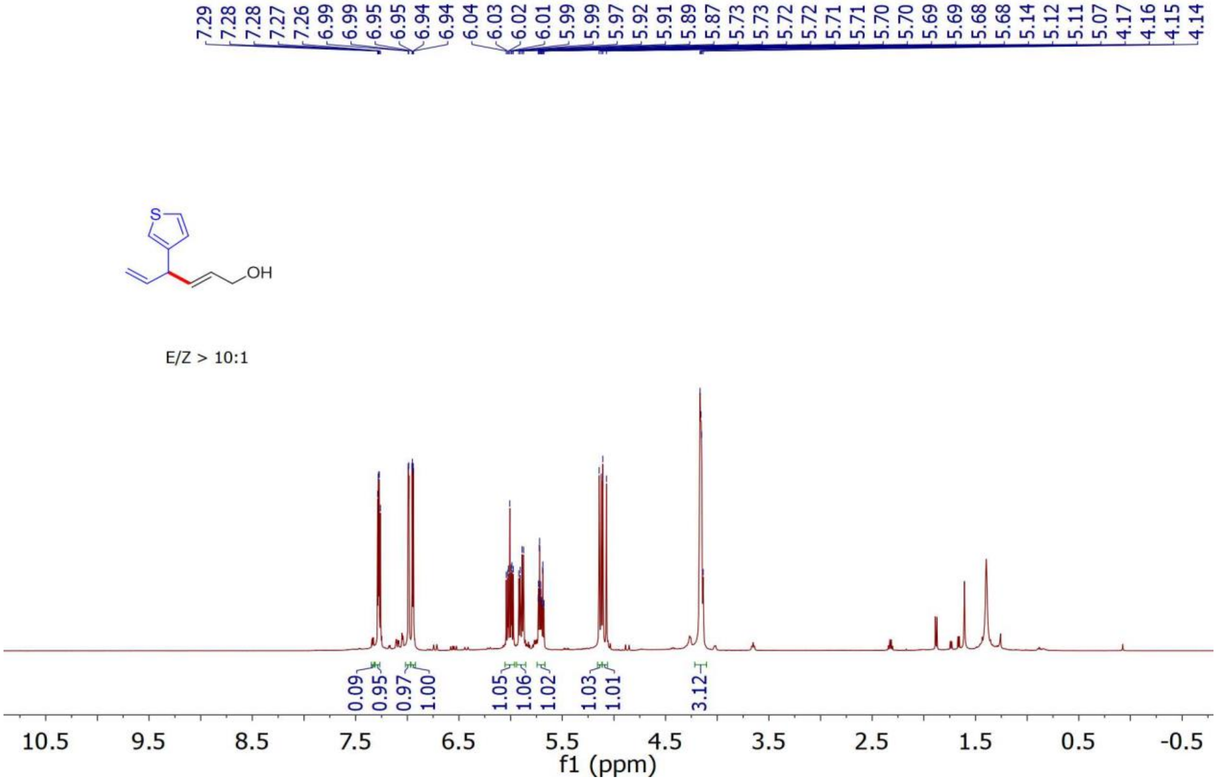

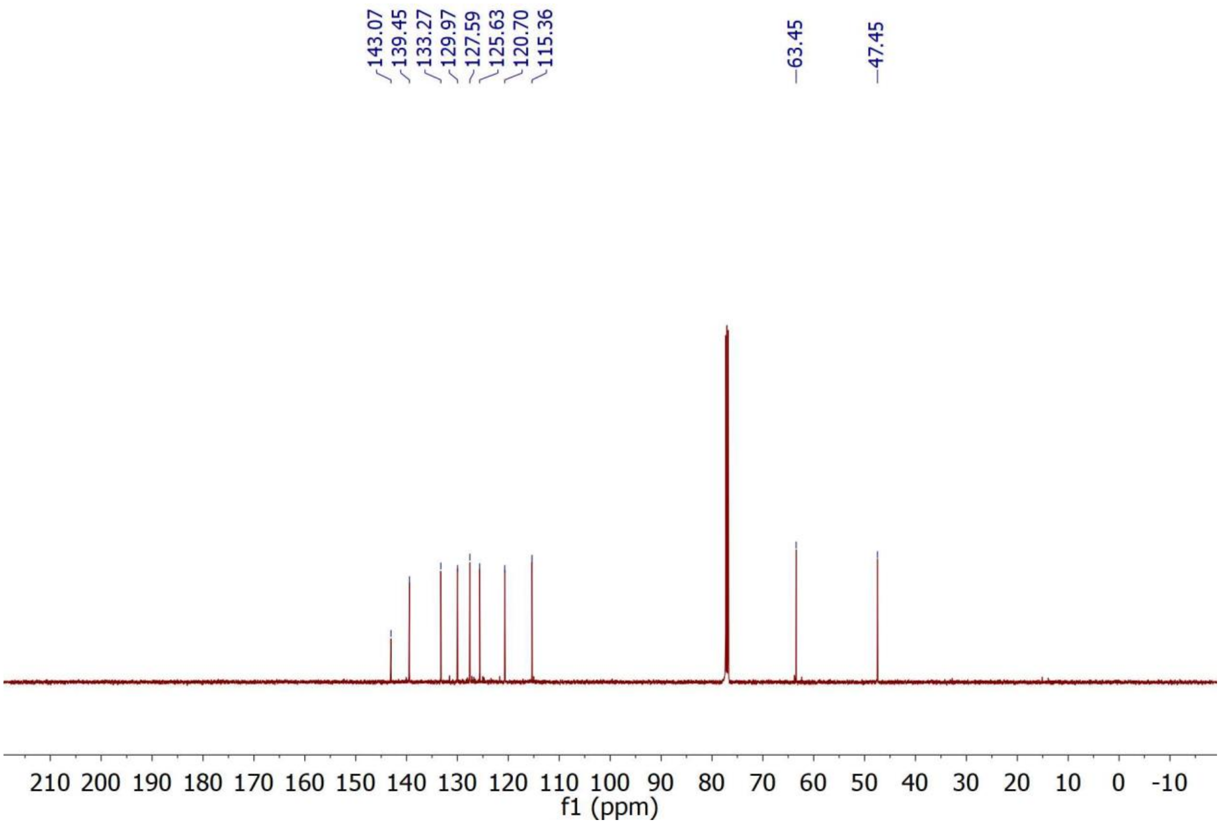


**(4R,4'S,5S)-2,2,2',2'-tetramethyl-5-((E)-3-methylbuta-1,3-dien-1-yl)-4,4'-bi(1,3-dioxolane) (4ba)：**

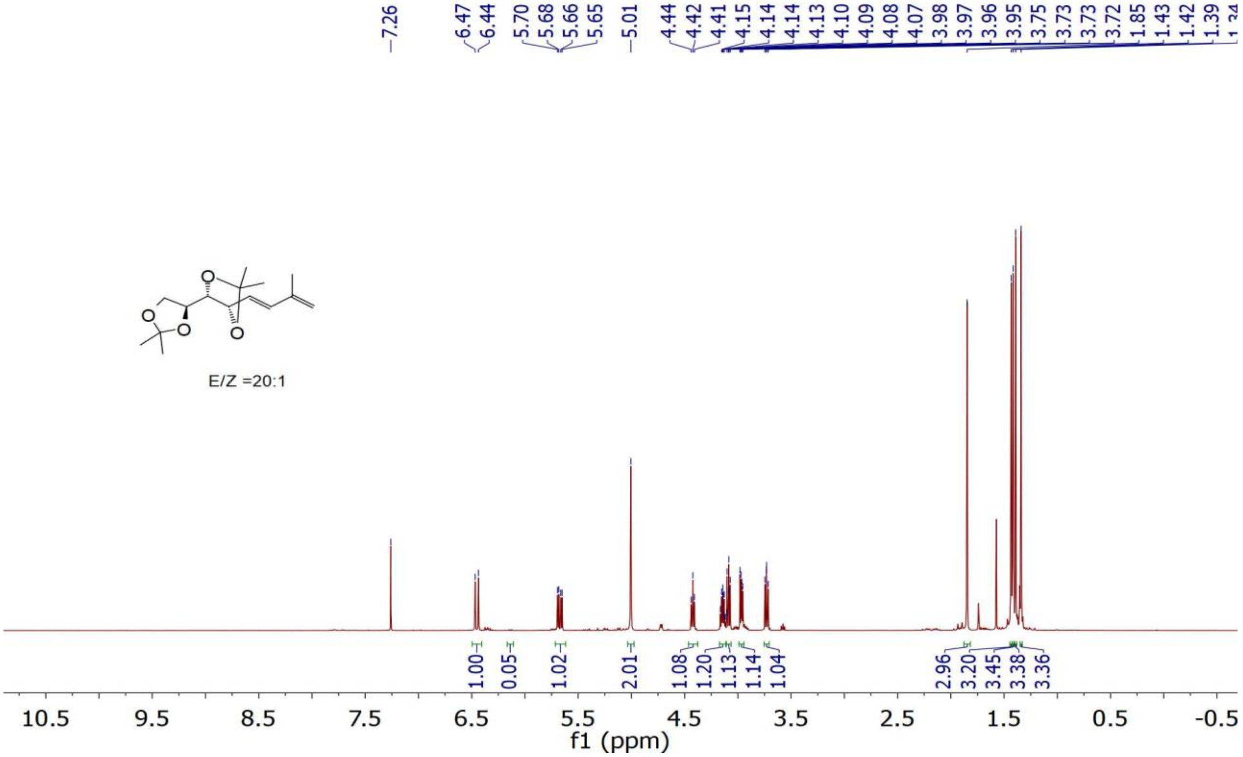

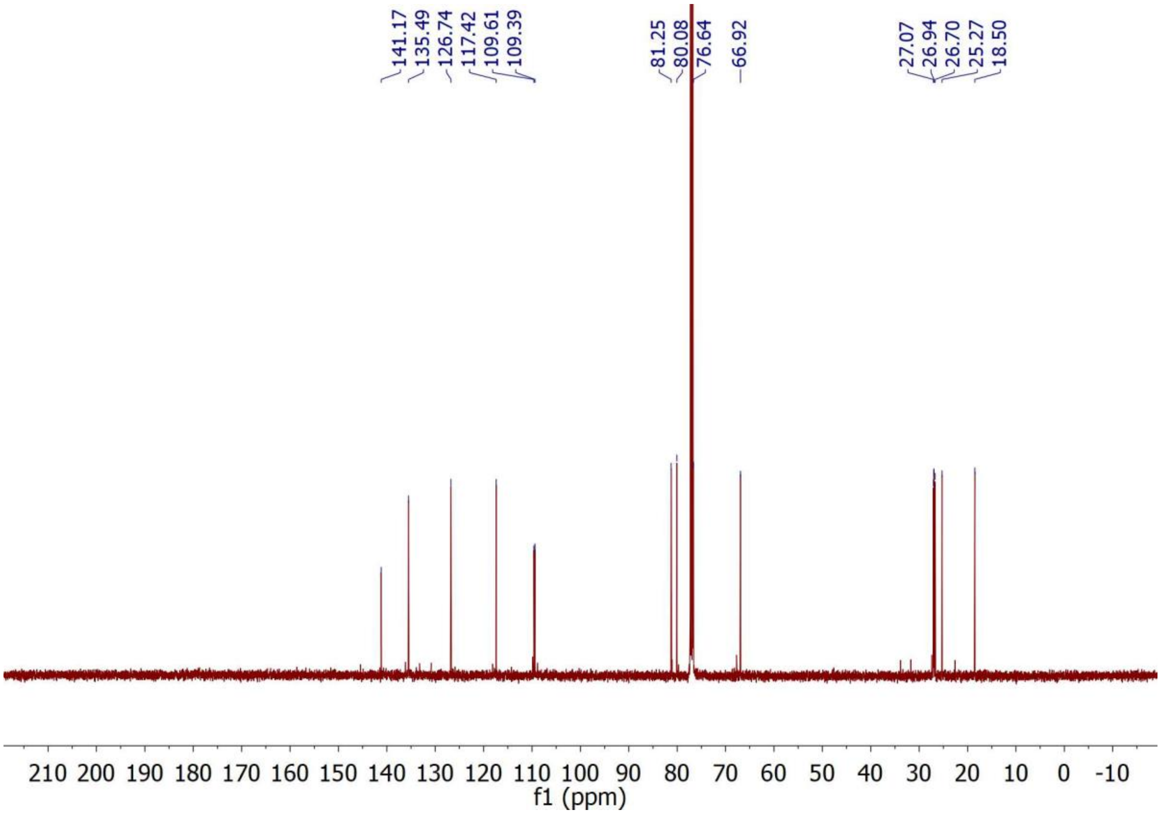


**(4*R*,4'*S*,5*S*)-5-((*E*)-3-benzylbuta-1,3-dien-1-yl)-2,2,2',2'-tetramethyl-4,4'-bi(1,3-dioxolane) (4bb)：**

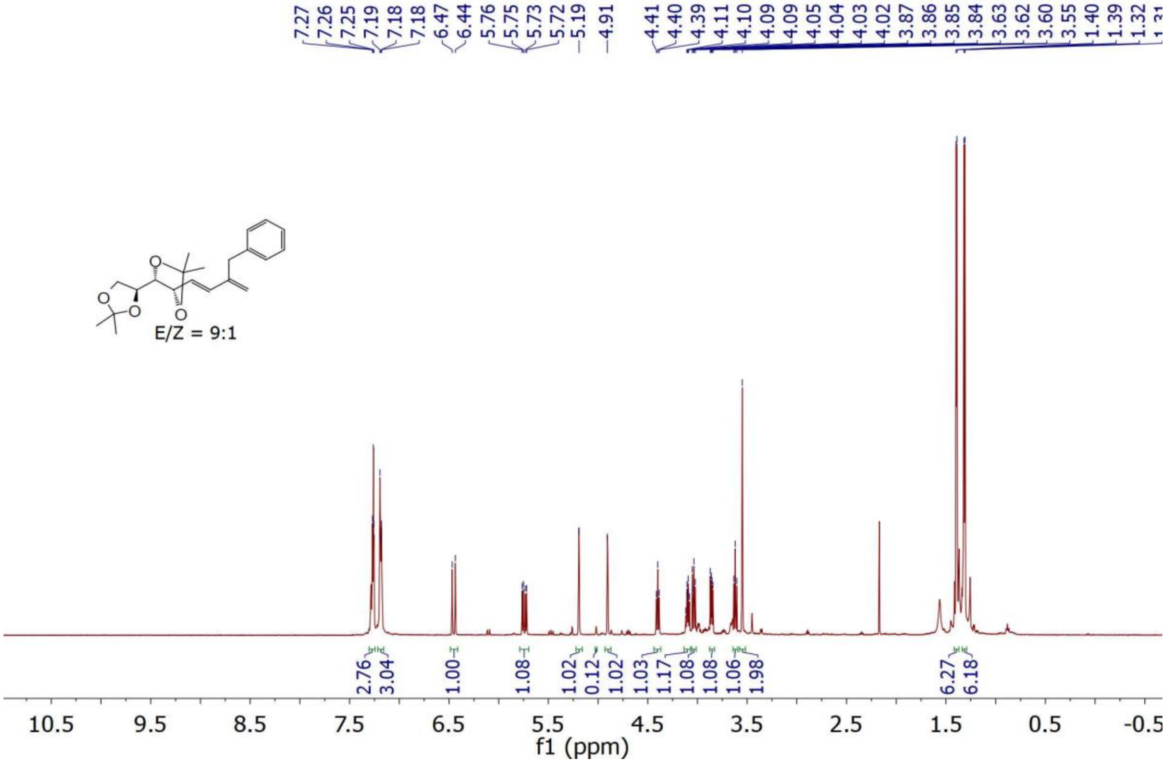

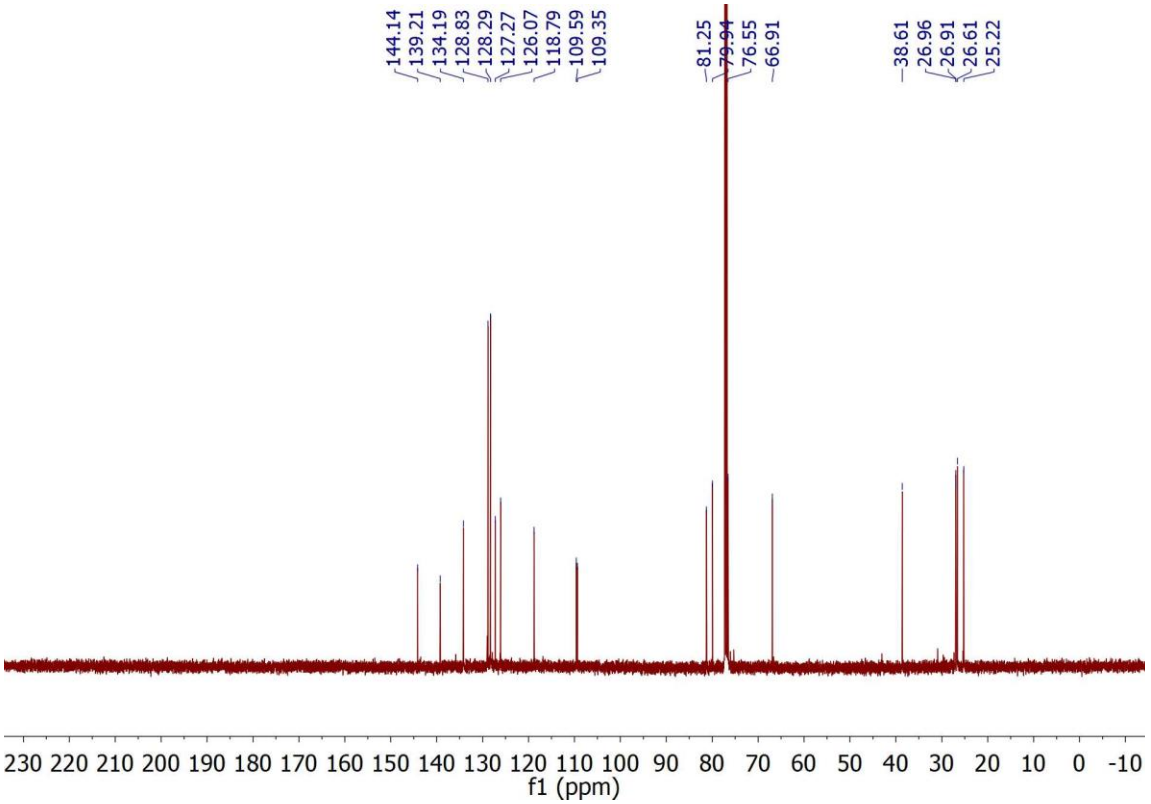

**Estrone derivative (5aa):**

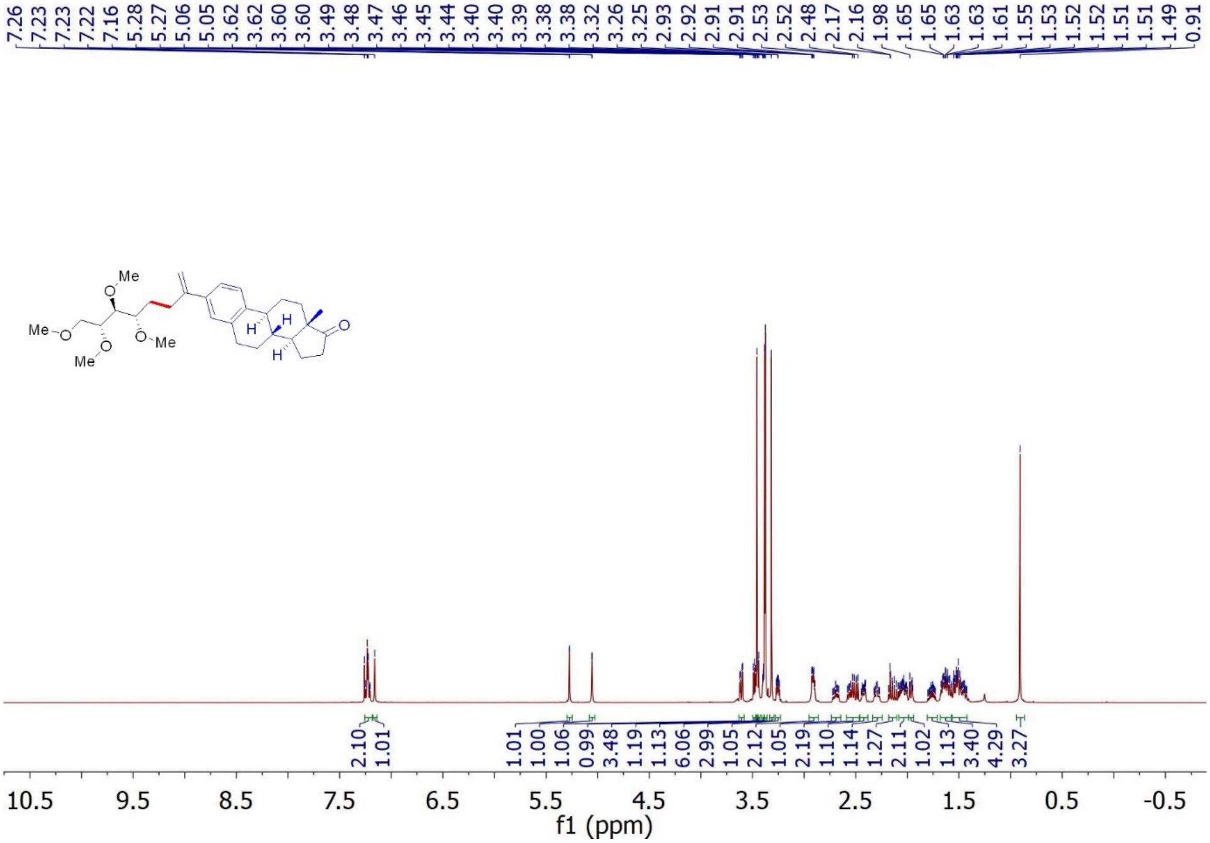

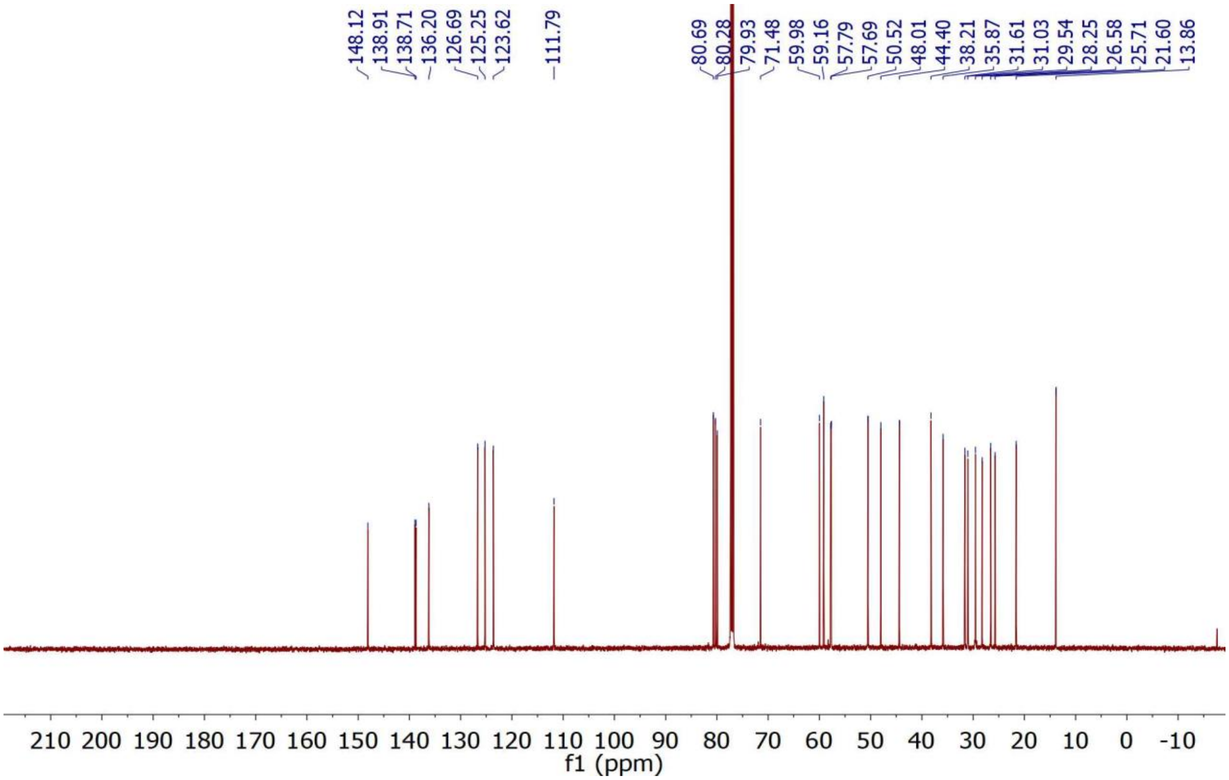

**Estrone derivative (5ab):**

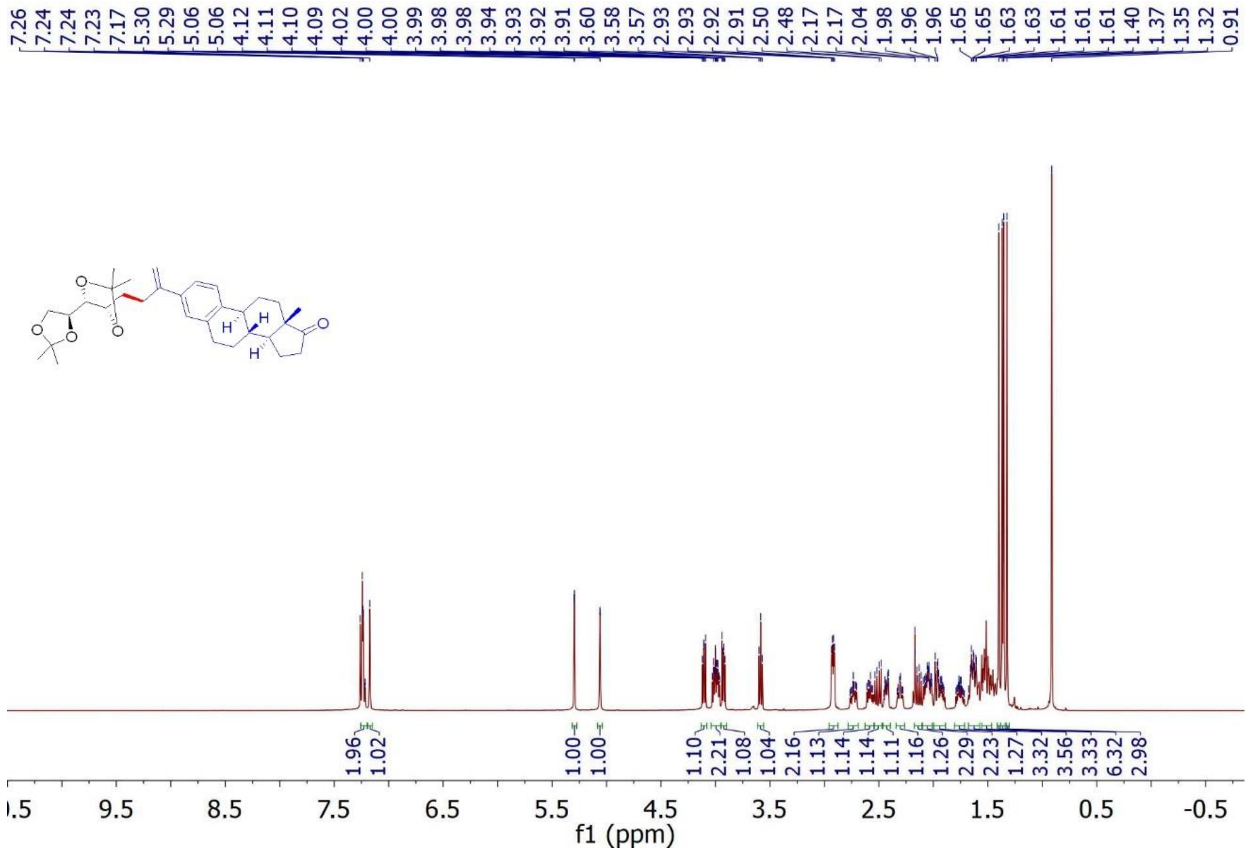

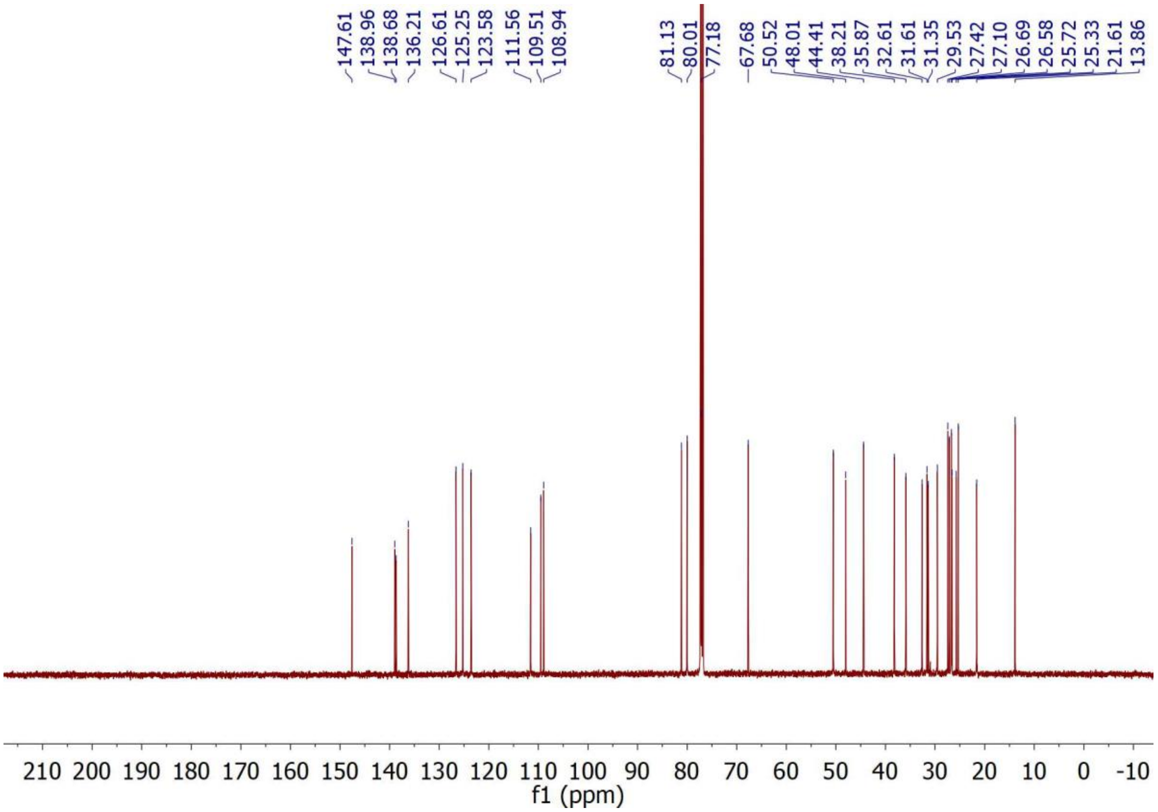


**Tyrosine derivative (5ac):**

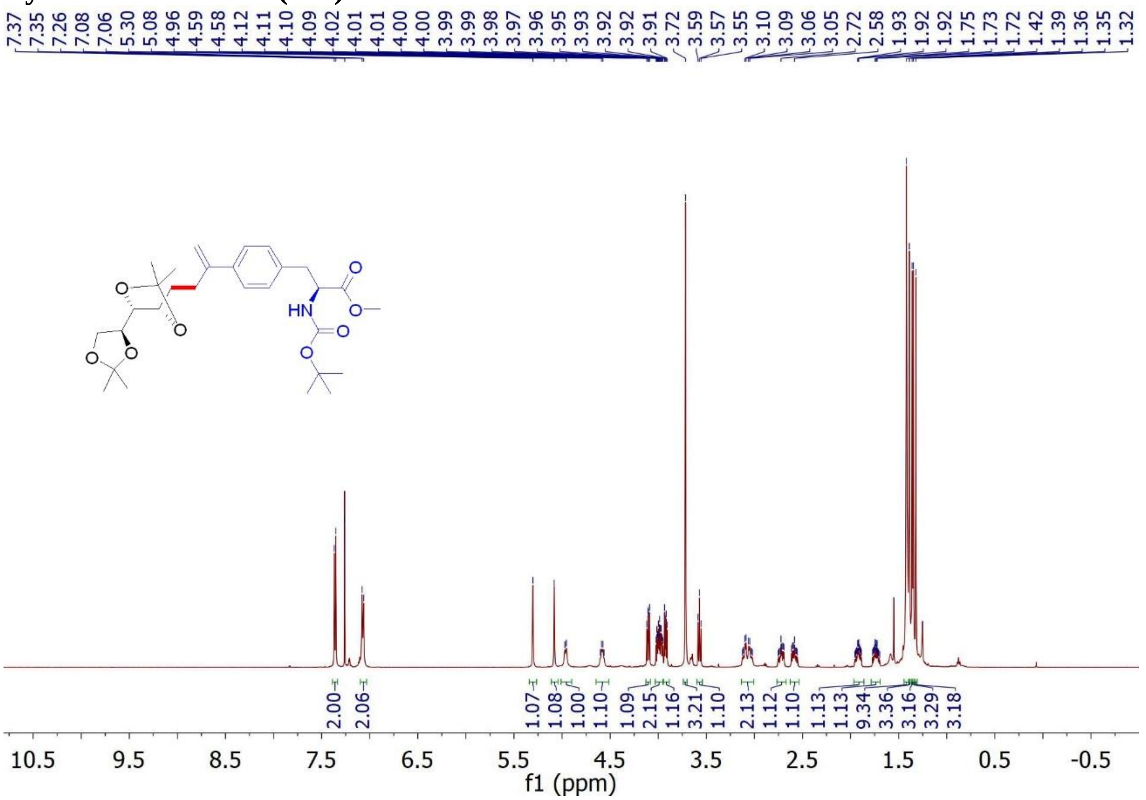

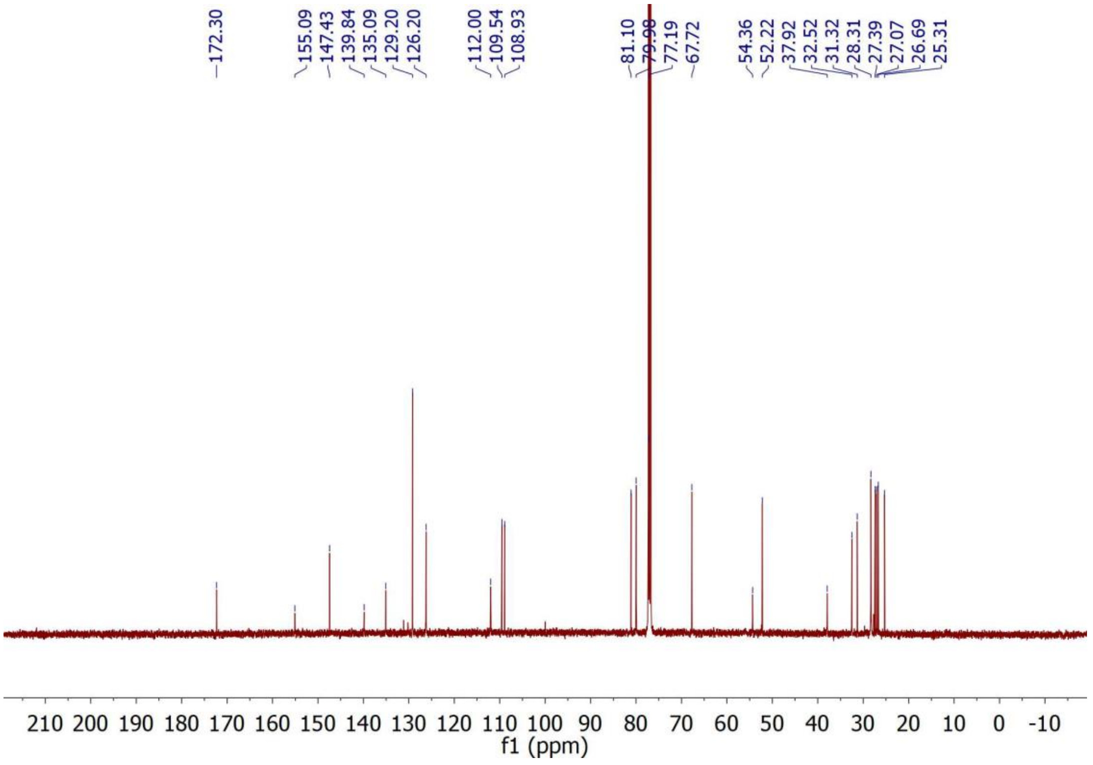


**1-(4-((4*R*,4'*S*,5*S*)-2,2,2',2'-tetramethyl-[4,4'-bi(1,3-dioxolan)]-5-yl)but-1-en-2-yl)-4-(4-((4*S*,4'*S*,5*R*)-2,2,2',2'-tetramethyl-[4,4'-bi(1,3-dioxolan)]-5-yl)but-1-en-2-yl)benzene (5ad):**

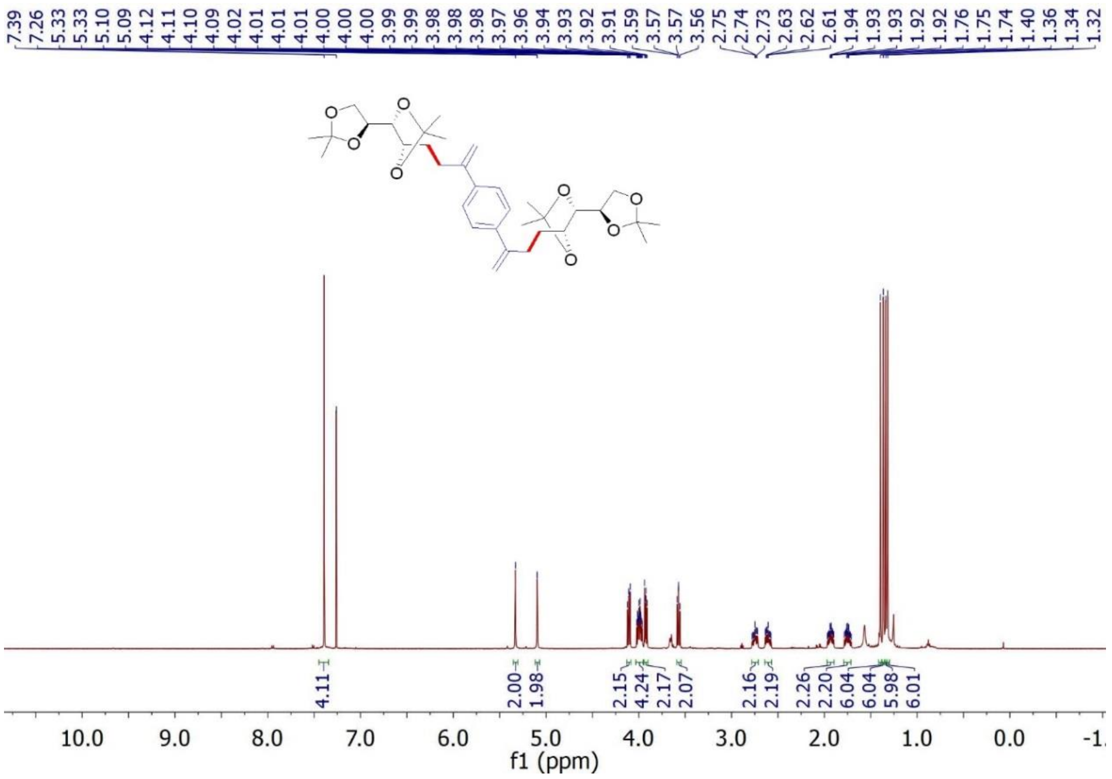

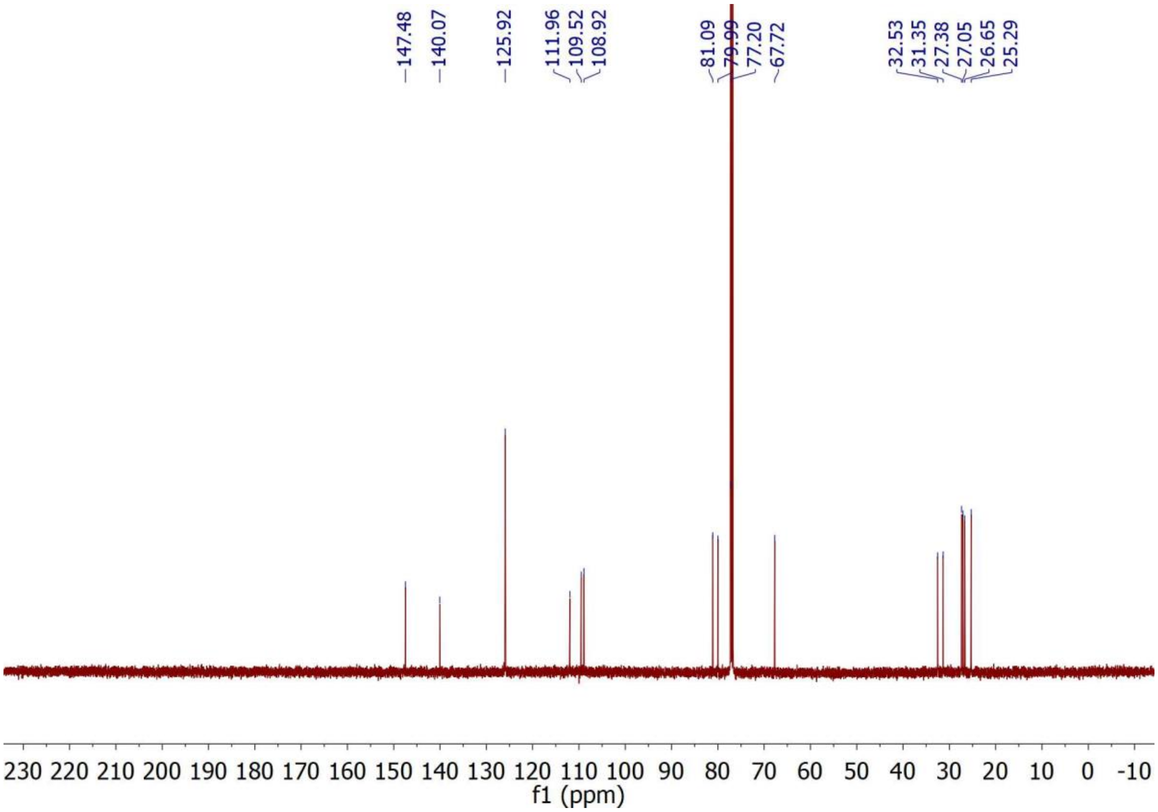


**(3a*R*,5a*S*,8a*S*,8b*R*)-2,2,7,7-tetramethyl-5-(3-(4-(4-((4*R*,4'*S*,5*S*)-2,2,2',2'-tetramethyl-[4,4'-bi(1,3-dioxolan)]-5-yl)but-1-en-2-yl)phenyl)but-3-en-1-yl)tetrahydro-5H-bis([1,3]dioxolo)[4,5-b:4',5'-d]pyran (5ae):**

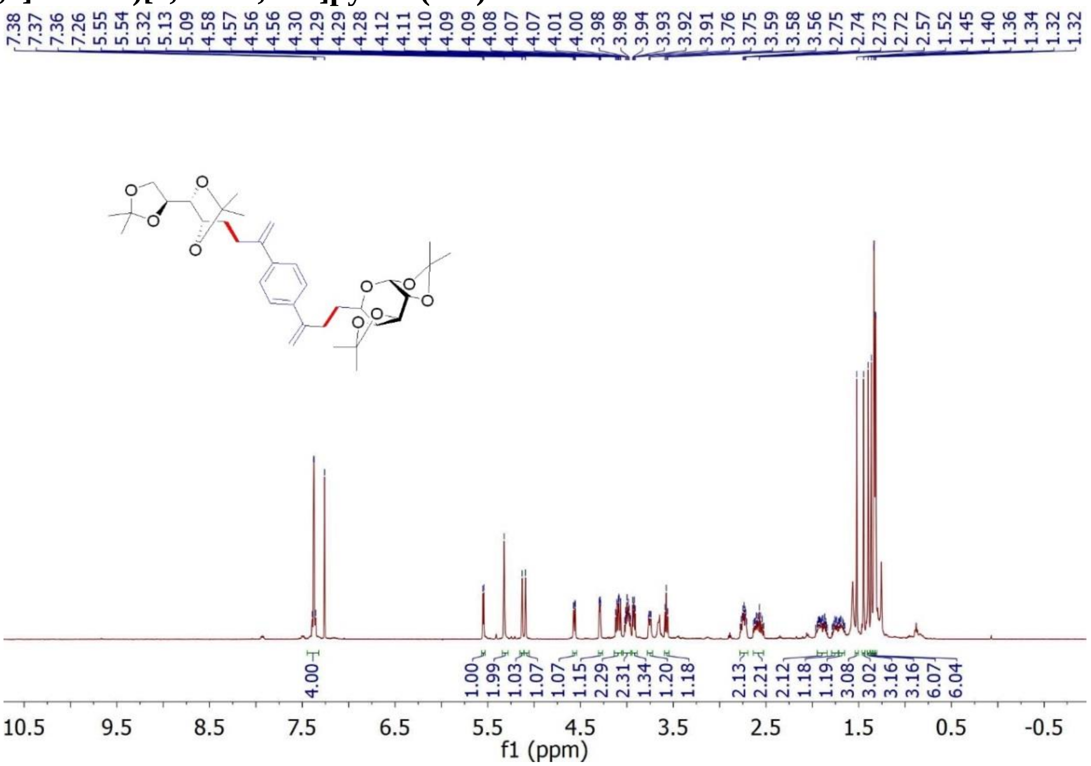

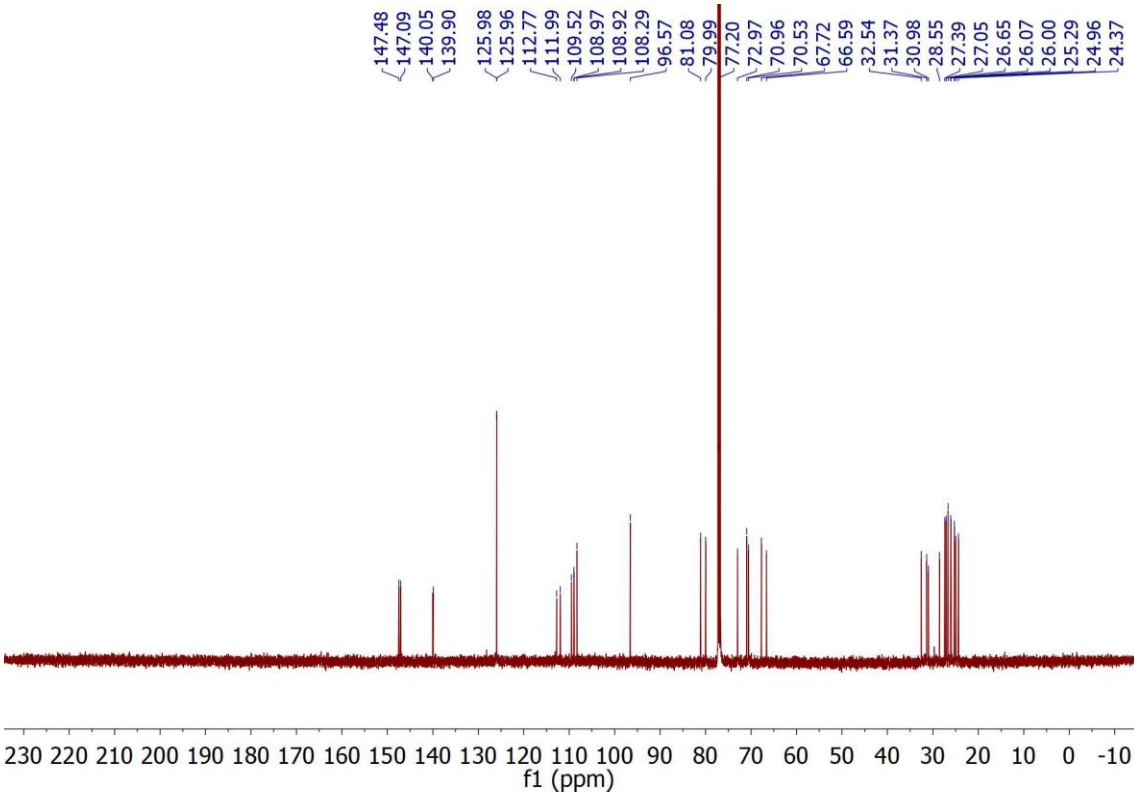


**(*R*)-6-((5S,6S,7R,E)-5,6,7,8-tetramethoxyoct-1-en-1-yl)-5,6-dihydro-2H-pyran-2-one (5af):**

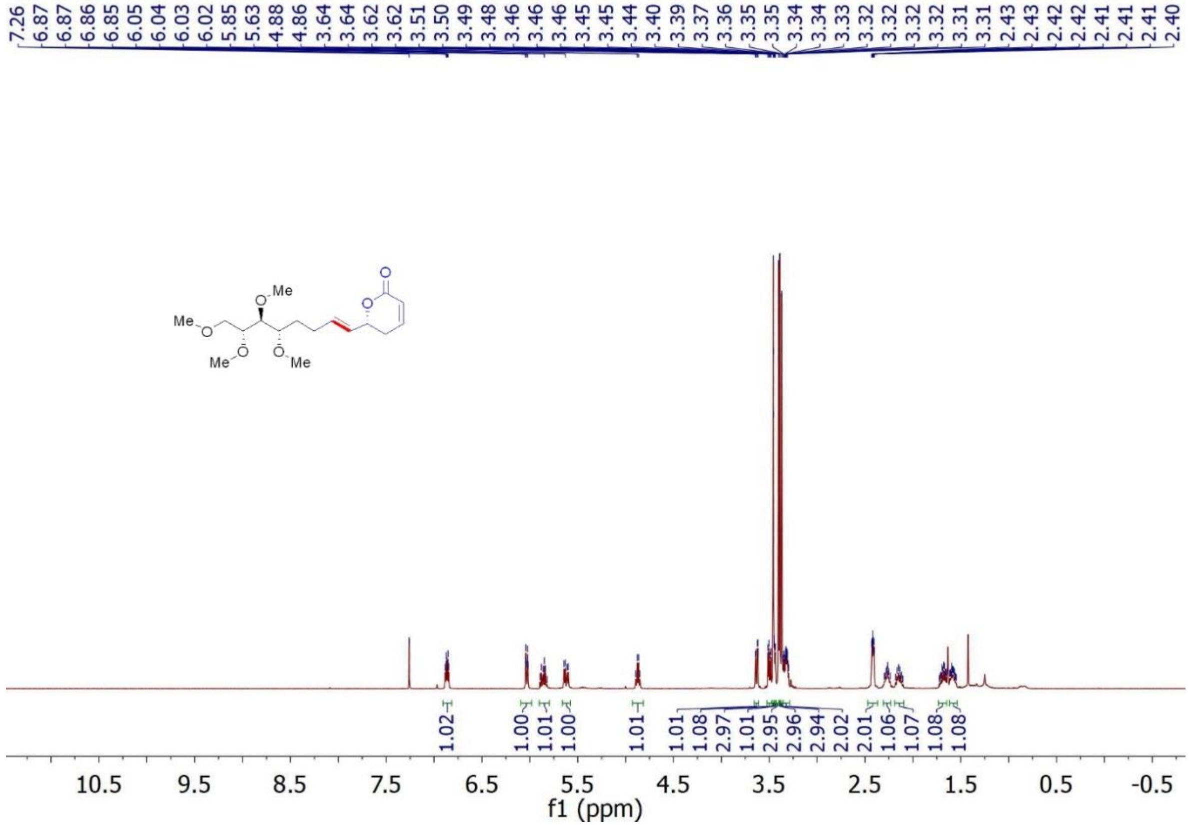

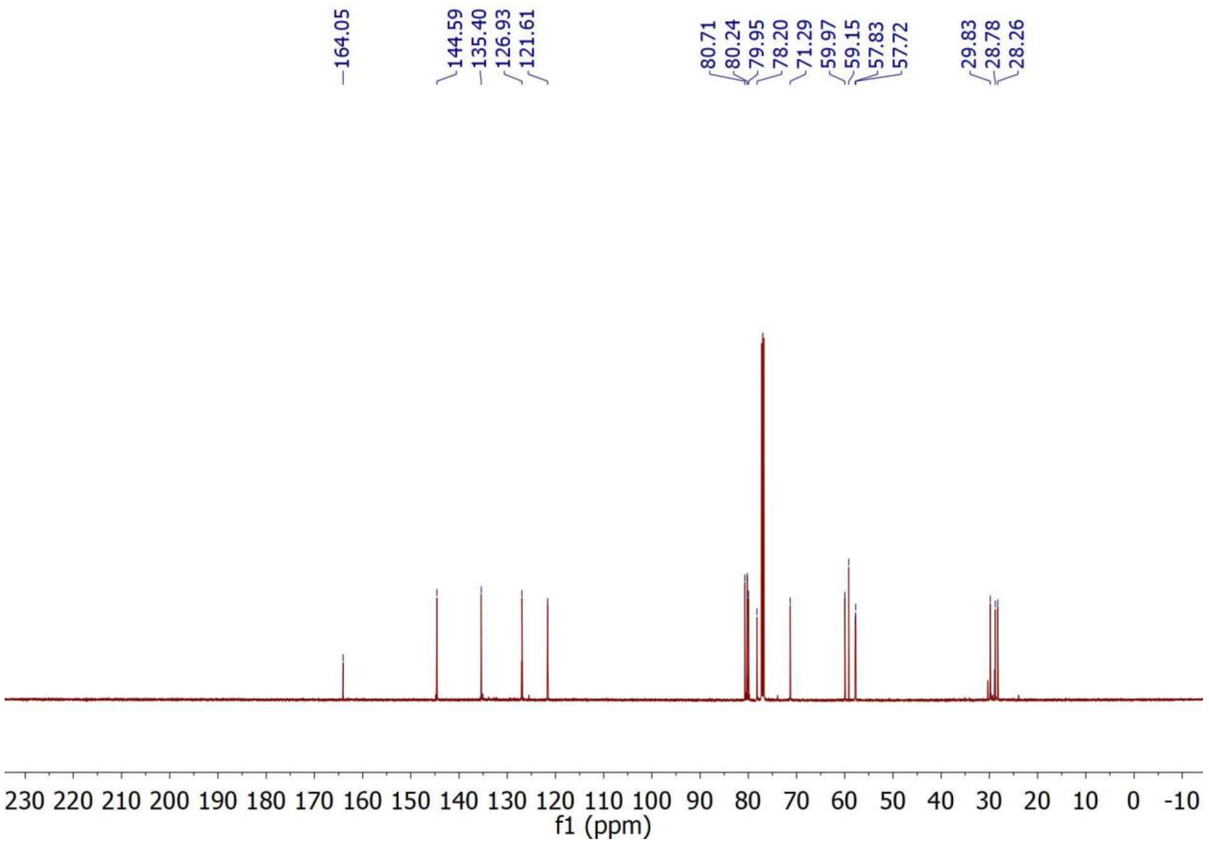

**(R)-6-((E)-4-((4R,4'S,5S)-2,2,2',2'-tetramethyl-[4,4'-bi(1,3-dioxolan)]-5-yl)but-1-en-1-yl)-5,6-dihydro-2H-pyran-2-one (5ag):**

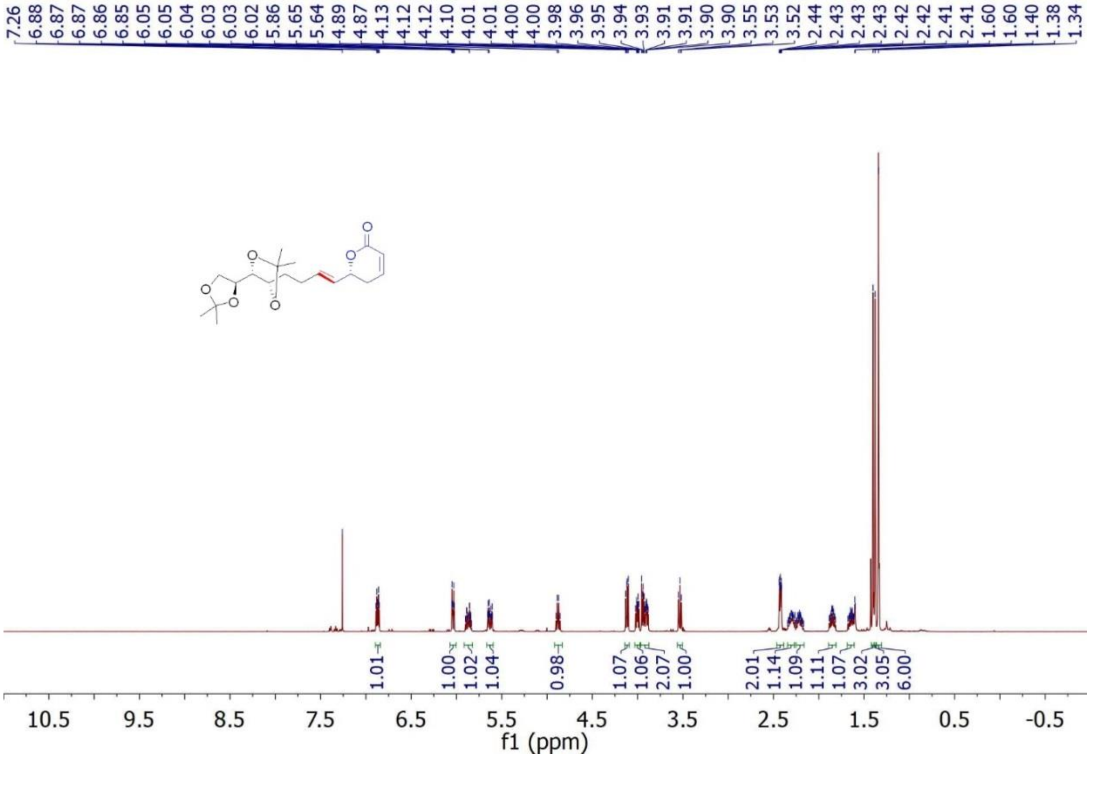

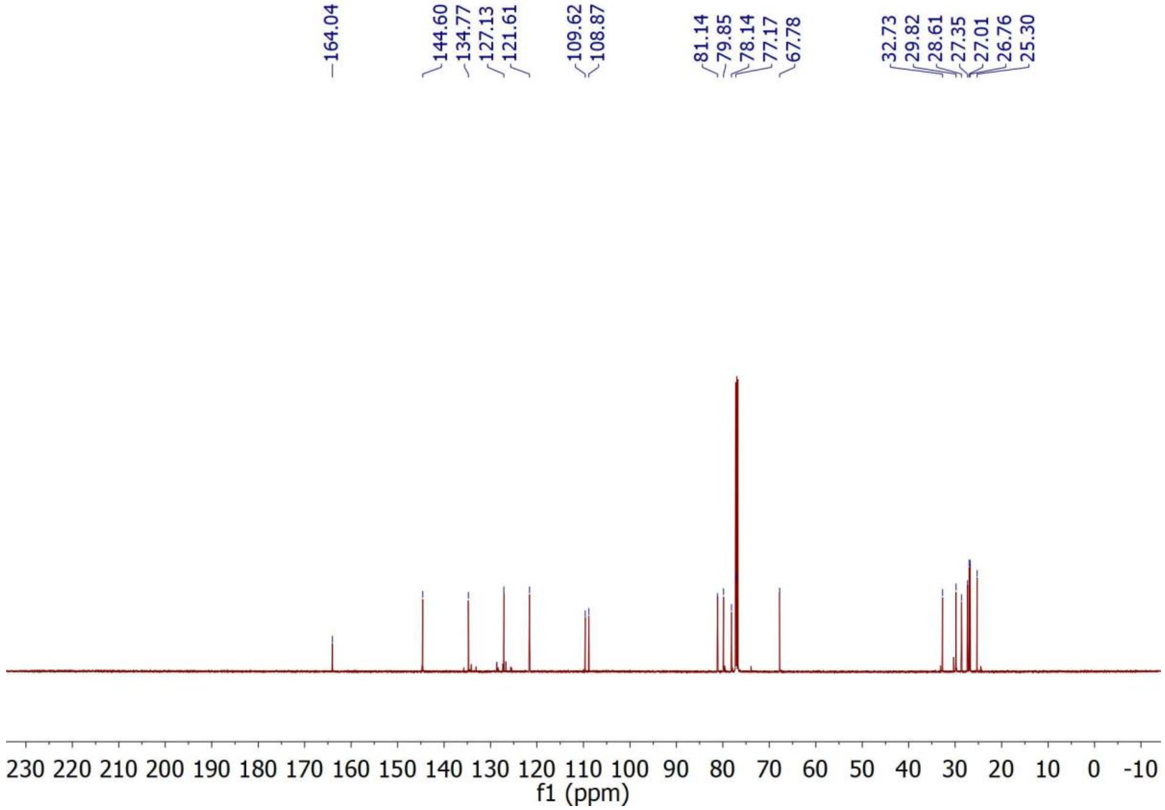


**(2R,3S,4R,5S,E)-9-((R)-6-oxo-3,6-dihydro-2H-pyran-2-yl)non-8-ene-2,3,4,5-tetrayl tetraacetate (5ah):**

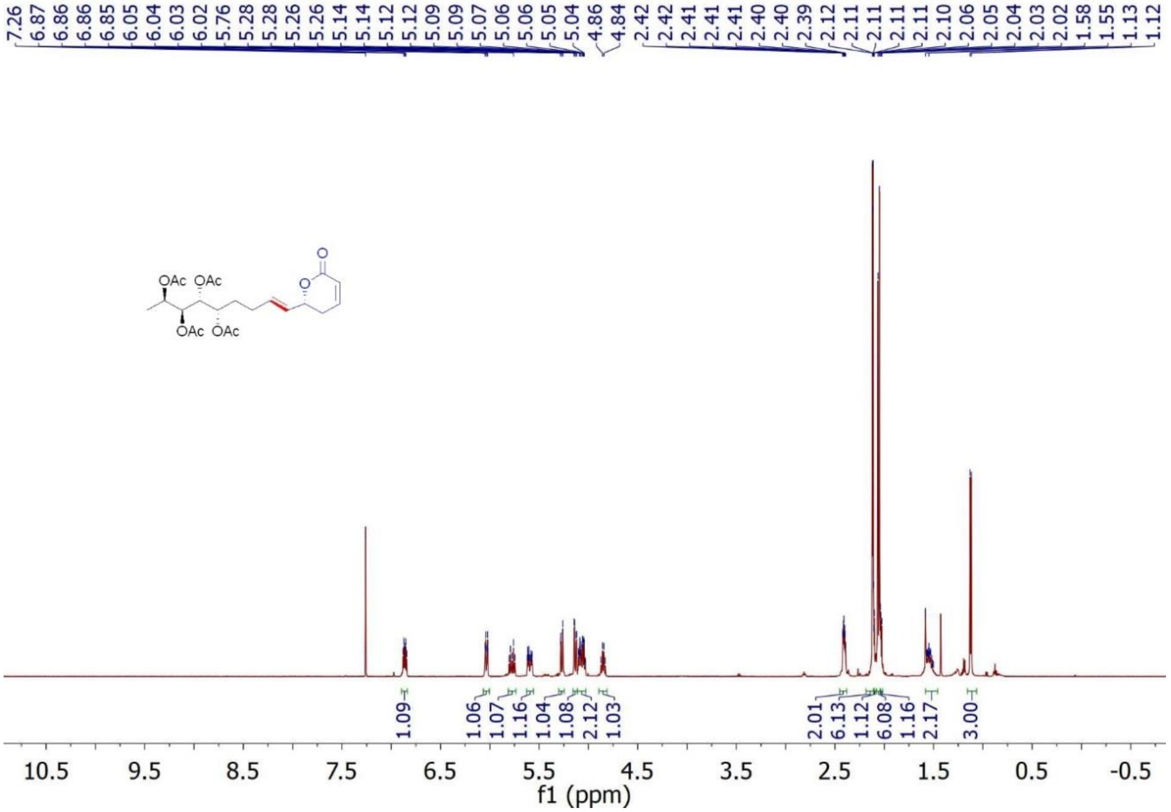

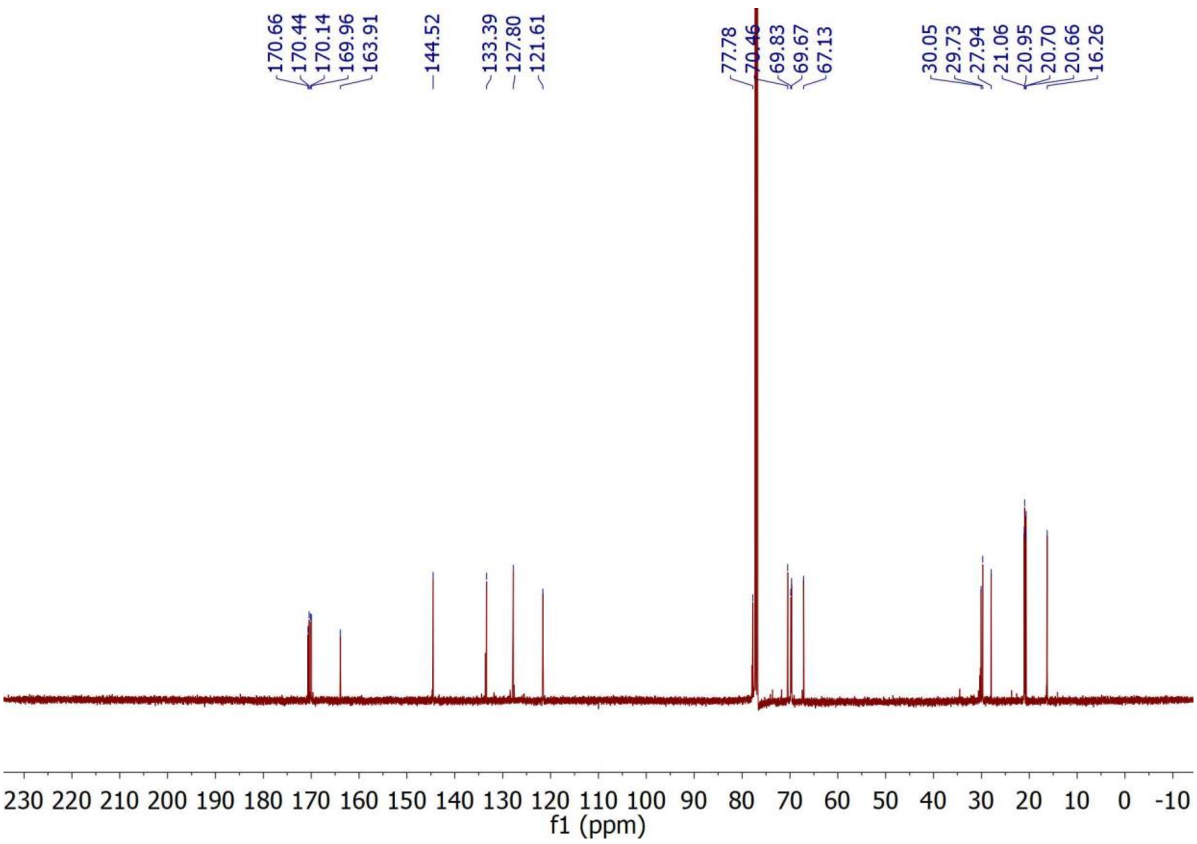
170.66
170.44
170.14
169.96
163.91
144.52
133.39
127.80
121.61
77.78
70.46
69.83
69.67
67.13
30.05
29.73
27.94
21.06
20.95
20.70
20.66
16.26
230 220 210 200 190 180 170 160 150 140 130 120 110 100 90 80 70 60 50 40 30 20 10 0 -10
f1 (ppm)

*VIII. 2D NOESY spectra*

**(3a*R*,5*R*,5a*S*,8a*S*,8b*R*)-5-(but-3-en-1-yl)-2,2,7,7-tetramethyltetrahydro-5H-bis([1,3]dioxolo)[4,5-b:4',5'-d]pyran (3aj):**

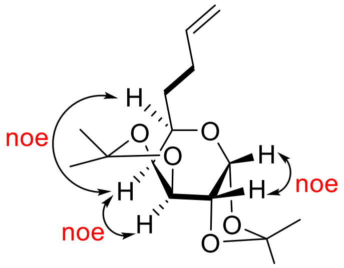


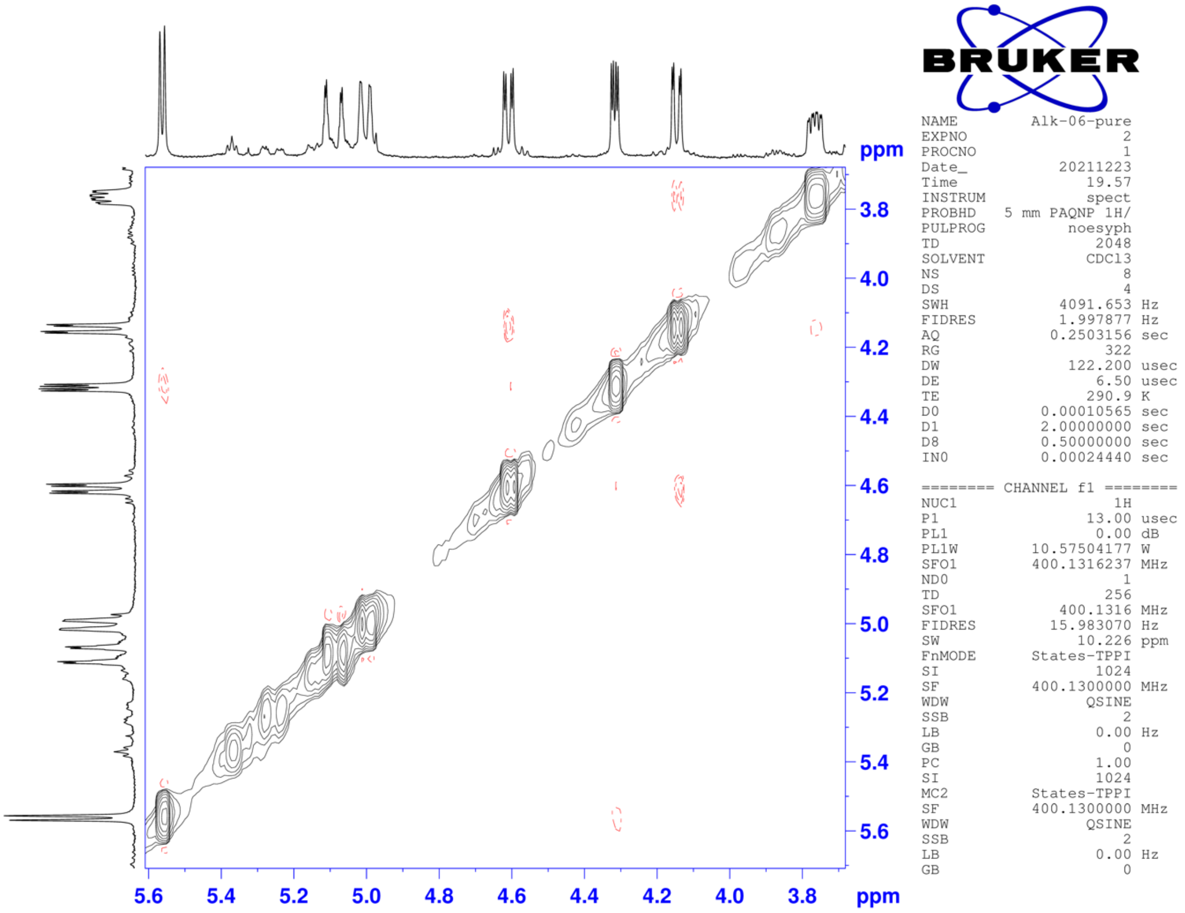

**(3a*R*,4*R*,6*R*,6a*R*)-4-(but-3-en-1-yl)-6-methoxy-2,2-dimethyltetrahydrofuro[3,4-d][1,3]dioxole (3al):**

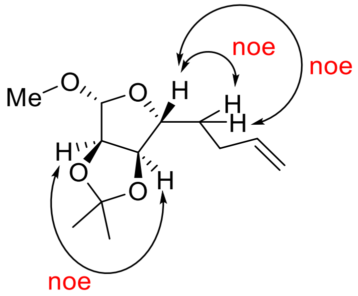


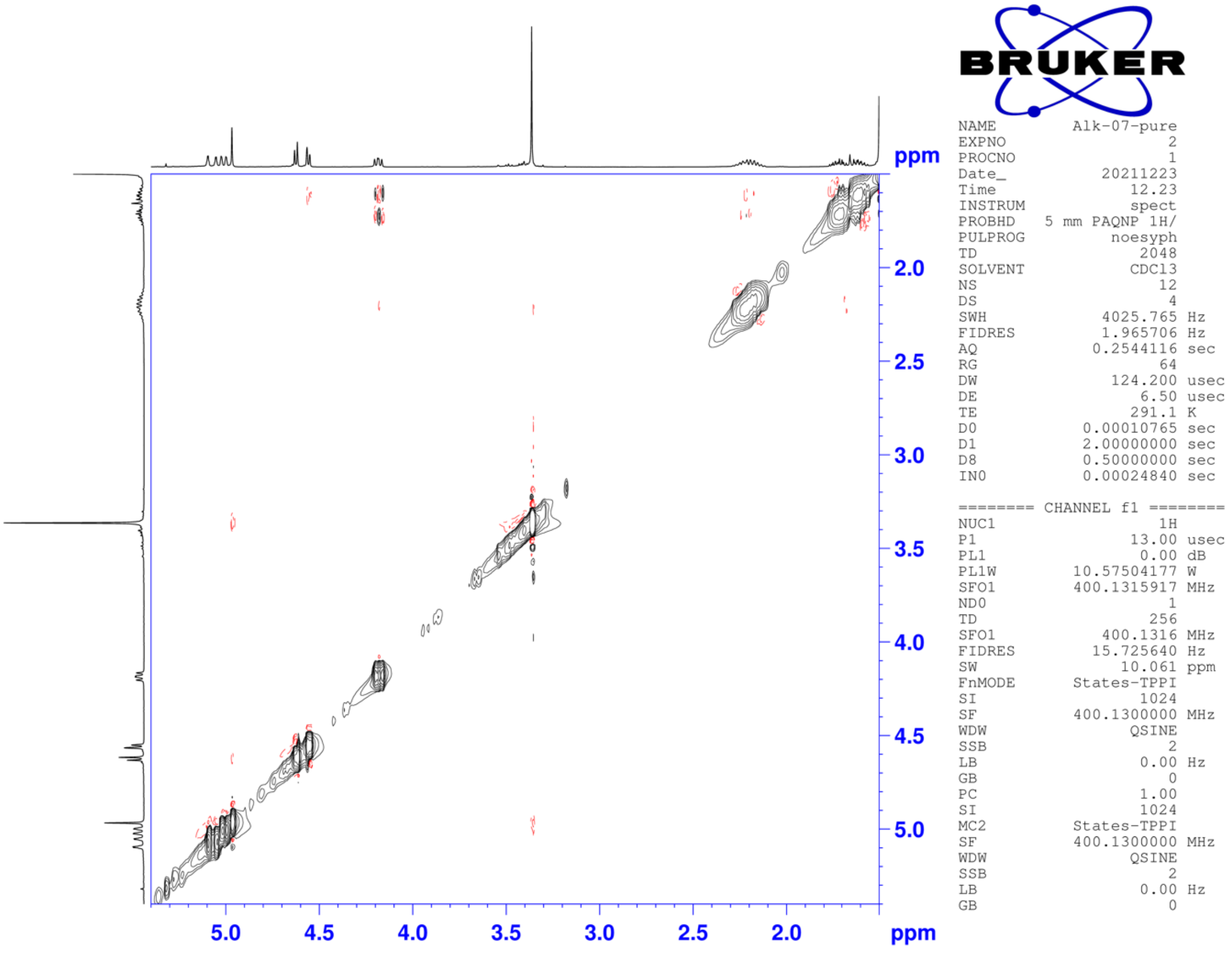

**(3a*S*,5a*R*,8a*R*,8b*S*)-3a-(but-3-en-1-yl)-2,2,7,7-tetramethyltetrahydro-5H-bis([1,3]dioxolo)[4,5-b:4',5'-d]pyran (3ak):**

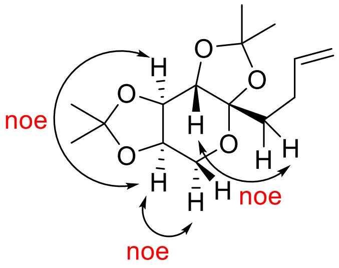


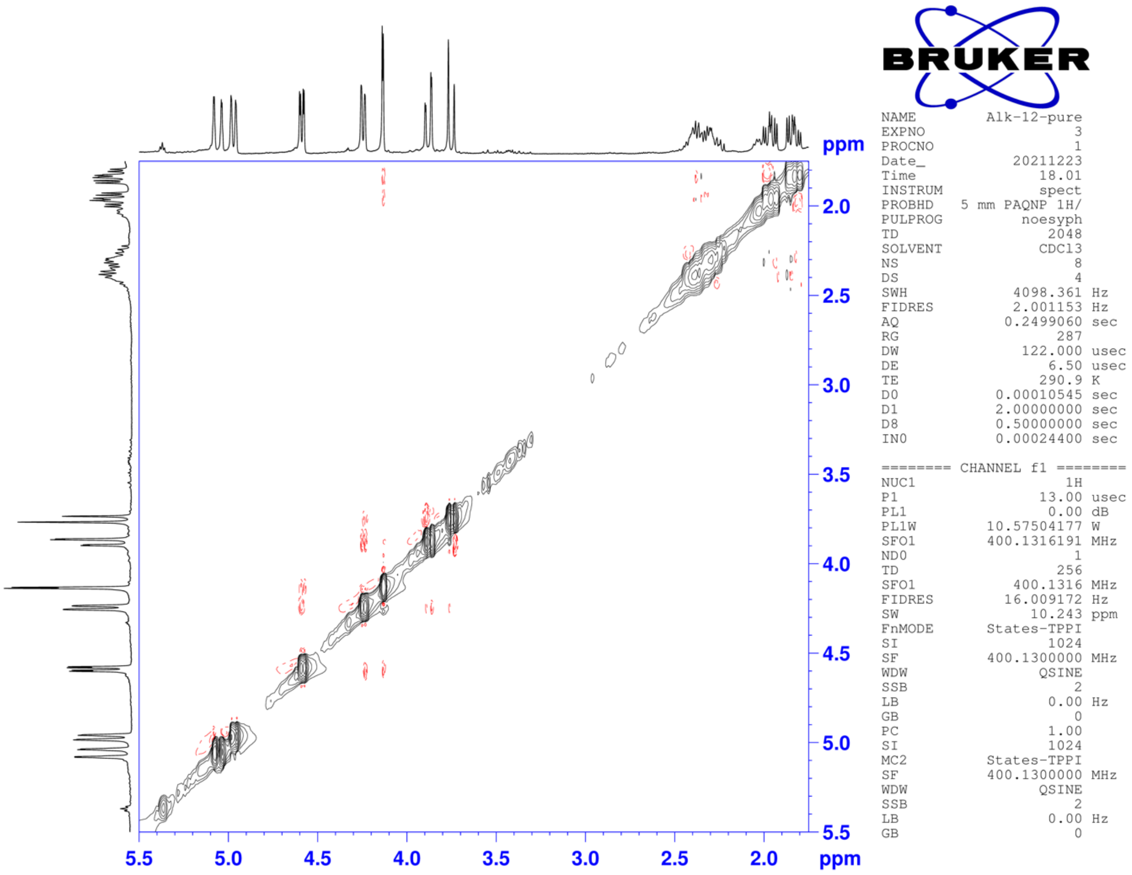

**(3a*R*,5*R*,5a*S*,8a*S*,8b*R*)-5-((*E*)-buta-1,3-dien-1-yl)-2,2,7,7-tetramethyltetrahydro-5*H*-bis([1,3]dioxolo)[4,5-*b*:4',5'-*d*]pyran (4ai):**

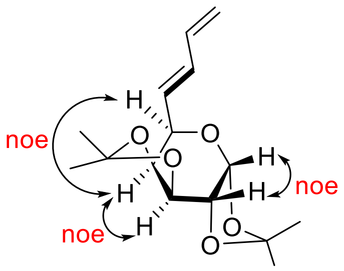


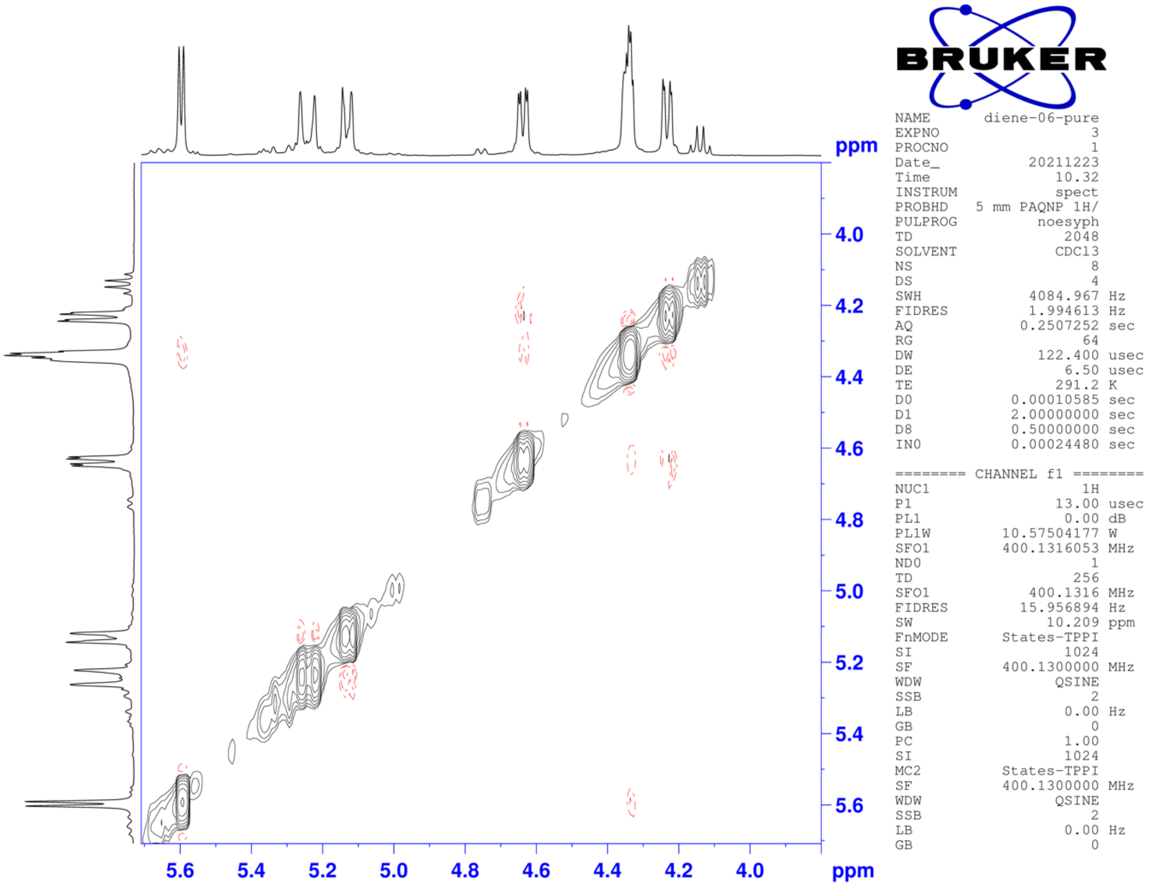

**(3a*S*,5a*R*,8a*R*,8b*S*)-3a-((*E*)-buta-1,3-dien-1-yl)-2,2,7,7-tetramethyltetrahydro-5*H*-bis([1,3]dioxolo)[4,5-*b*:4',5'-*d*]pyran (4aj):**

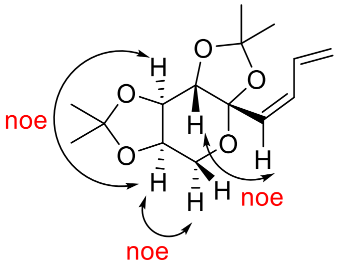


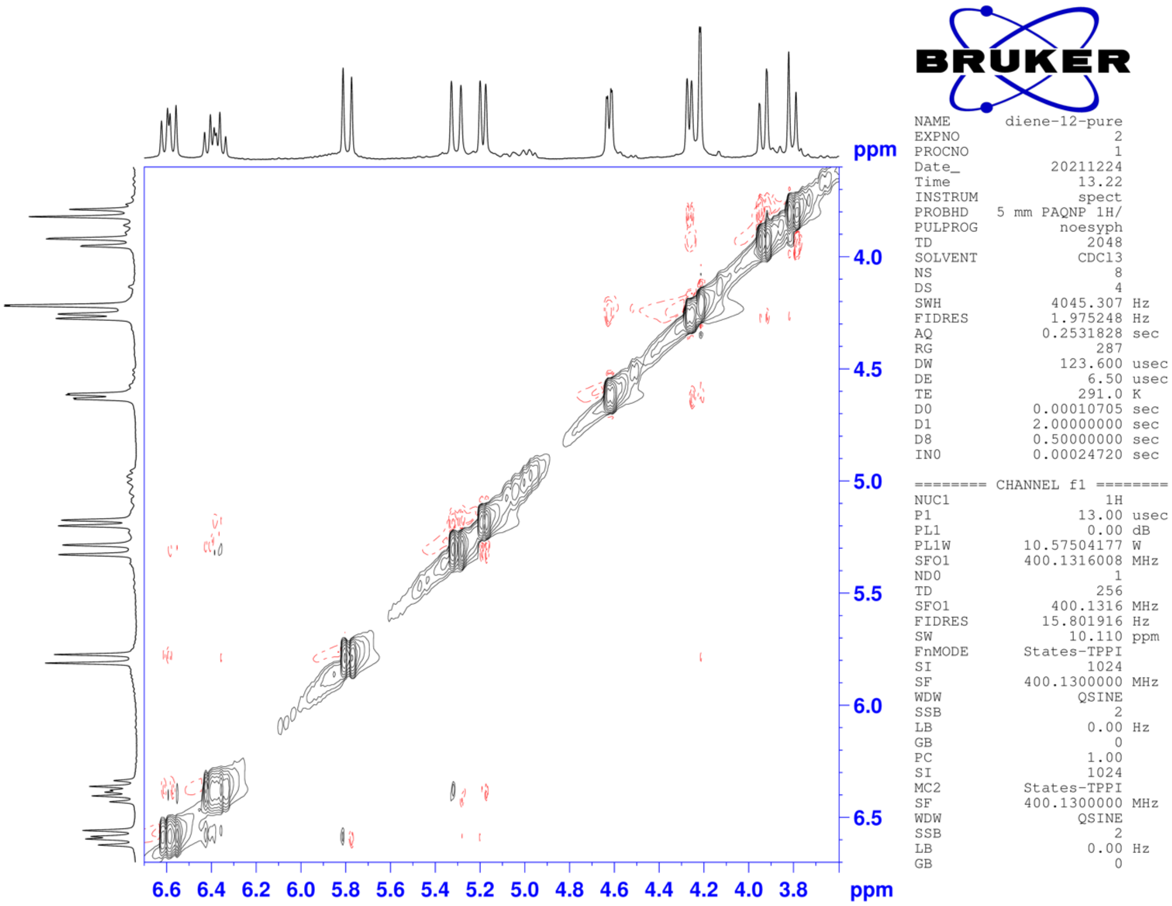

**(3a*R*,4*R*,6*R*,6a*R*)-4-((*E*)-buta-1,3-dien-1-yl)-6-methoxy-2,2-dimethyltetrahydrofuro[3,4-*d*][1,3]dioxole (4ak):**

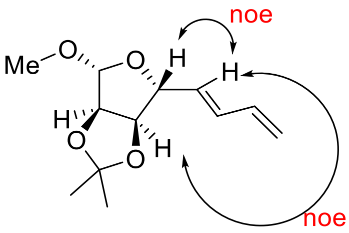


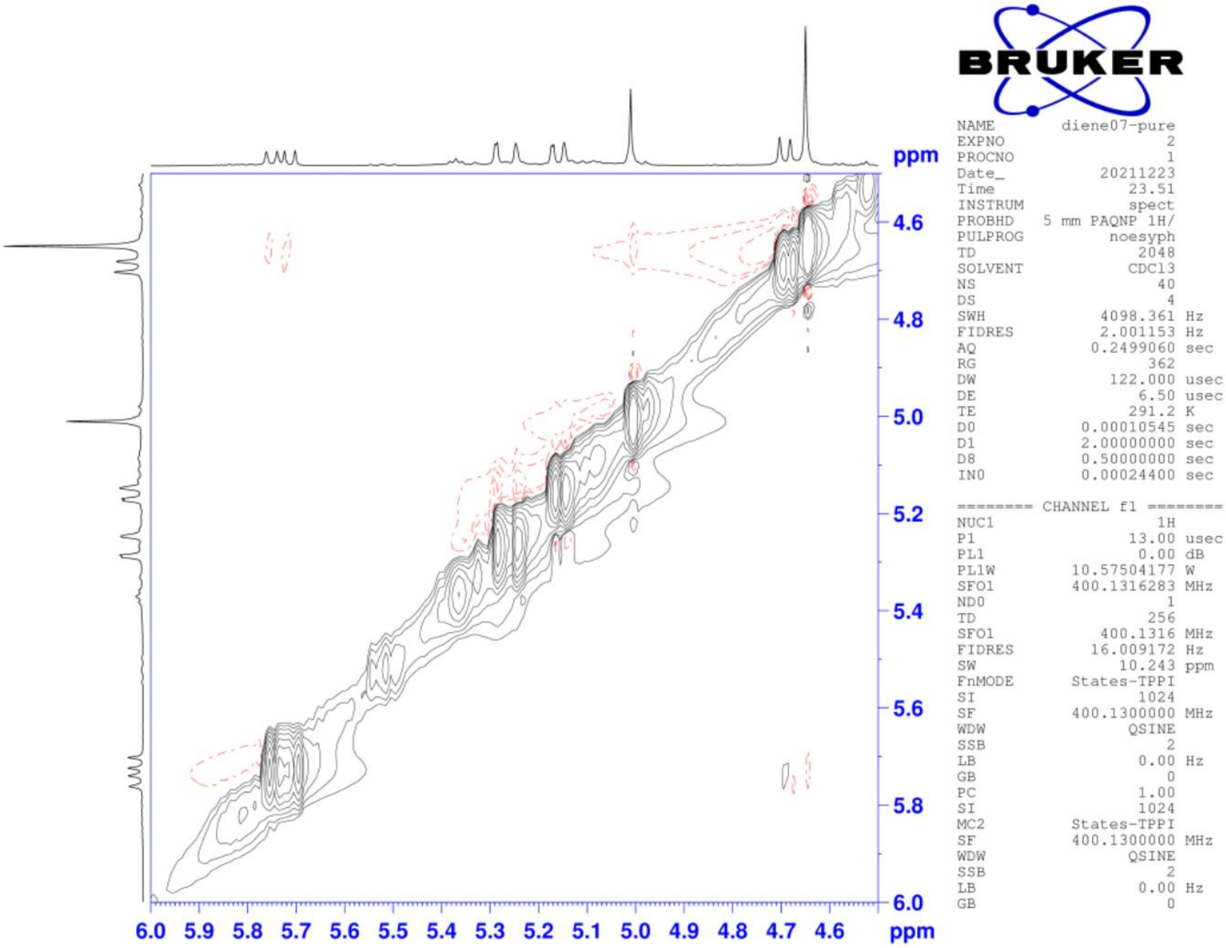

*IV. X-ray crystallographic data*

Single crystals of **3am** and **4am** were obtained through slow evaporation from their solutions in MeOH. The structures and absolute configurations of **3am** and **4am** were then determined by x-ray crystallographic analysis.

**Figure S1. X-ray structure of compound 3am.** (**CCDC 2112299**)

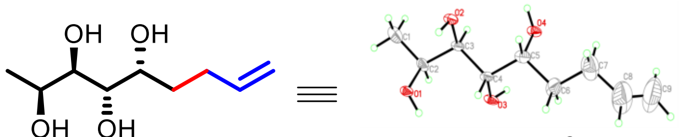


X-ray of **3am**

**Table S2 Crystal data of product 3am**

| | | |
|---|---|---|
| Identification code | Complex **3am** | |
| Empirical formula | C9 H18 O4 | |
| Formula weight | 190.23 | |
| Temperature | 150(2) K | |
| Wavelength | 0.71073 | |
| Crystal system | triclinic | |
| Space group | P1 | |
| Unit cell dimensions | a = 4.9077(4) Å | α = 107.254(5)° |
| | b = 6.0065(6) Å | β = 96.747(5)° |
| | c = 9.7569(12) Å | γ = 92.763(4)° |
| Volume | 271.72(5) Å$^3$ | |
| Z | 1 | |
| Density (calculated) | 1.163 g/cm$^3$ | |
| Absorption coefficient | 0.090 mm$^{-1}$ | |
| F(000) | 104.0 | |
| Crystal size | 0.30 x 0.10 x 0.10 mm$^3$ | |

| | |
|---|---|
| Theta range for data collection | 3.57 to 32.83° |
| Index ranges | -7<=H<=7, -9<=k<=8, -14<=l<=14 |
| Reflections collected | 10197 |
| Independent reflections | 3687 [R(int) = 0.0330] |
| Absorption correction | Semi-empirical from equivalents |
| Max. and min. transmission | 0.7039 and 0.7465 |
| Refinement method | Full-matrix least-squares on F2 |
| Data / restraints / parameters | 3687/4/124 |
| Goodness-of-fit on F2 | 1.054 |
| Final R indices [I>2sigma(I)] | R 1 =0.0580, wR2 = 0.1598 |
| R indices (all data) | R 1 = 0.0830, wR2 = 0.1842 |
| Largest diff. peak and Hole | 0.403 and -0.296 e.Å$^{-3}$ |

**Figure S2. X-ray structure of compound 4am (CCDC 2104006)**

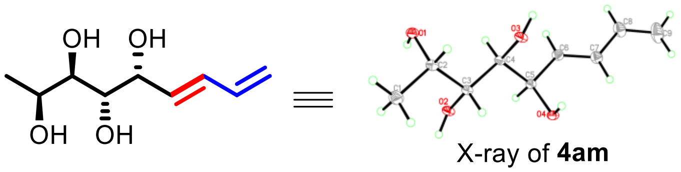


**Table S3 Crystal data of product 4am**

| | |
|---|---|
| Identification code | Complex **4am** |
| Empirical formula | C9 H16 O4 |
| Formula weight | 188.22 |
| Temperature | 150(2) K |
| Wavelength | 1.54178 |

| | | |
|---|---|---|
| Crystal system | Monoclinic | |
| Space group | C2 | |
| Unit cell dimensions | a = 6.6119(7) Å | α = 90.00° |
| | b = 7.1110(19) Å | β = 97.711(17)° |
| | c = 21.787(4) Å | γ = 90.00° |
| Volume | 1015.1(3) $Å^3$ | |
| Z | 4 | |
| Density (calculated) | 1.232 $g/cm^3$ | |
| Absorption coefficient | 0.802 $mm^{-1}$ | |
| F(000) | 408 | |
| Crystal size | 0.70 x 0.45 x 0.13 $mm^3$ | |
| Theta range for data collection | 3.81 to 30.55° | |
| Index ranges | -8<=H<=7, -8<=k<=8, -26<=l<=26 | |
| Reflections collected | 4939 | |
| Independent reflections | 1802 [R(int) = 0.0600] | |
| Absorption correction | Semi-empirical from equivalents | |
| Max. and min. transmission | 0.6036 and 0.9029 | |
| Refinement method | Full-matrix least-squares on F2 | |
| Data / restraints / parameters | 1802/1/118 | |
| Goodness-of-fit on F2 | 1.097 | |
| Final R indices [I>2sigma(I)] | R 1 =0.0528, wR2 = 0.1455 | |
| R indices (all data) | R 1 = 0.0551, wR2 = 0.1566 | |
| Largest diff. peak and Hole | 0.388 and -0.261 e.$Å^{-3}$ | |

## REFERENCES AND NOTES


1. H. G. Garg, M. K. Cowman, C. A. Hales, in *Carbohydrate Chemistry, Biology and Medical Applications* (Elsevier, 2008) pp. 1–405.

2. Y. Yang, B. Yu, Recent advances in the chemical synthesis of C-glycosides. *Chem. Rev.* **117**, 12281–12356 (2017).

3. V. Dimakos, M. S. Taylor, Site-selective functionalization of hydroxyl groups in carbohydrate derivatives. *Chem. Rev.* **118**, 11457–11517 (2018).

4. R. C. Larock, *Comprehensive Organic Transformations: A Guide to Functional Group Preparations* (VCH, ed. 2, 1999).

5. W. Erb, J. Zhu, From natural product to marketed drug: The tiacumicin odyssey. *Nat. Prod. Rep.* **30**, 161–174 (2013).

6. B. E. Maryanoff, A. B. Reitz, The Wittig olefination reaction and modifications involving phosphoryl-stabilized carbanions. Stereochemistry, mechanism, and selected synthetic aspects. *Chem. Rev.* **89**, 863–927 (1989).

7. P. A. Runeberg, P. C. Eklund, Tsuji-Wacker-type oxidation beyond methyl ketones: Reacting unprotected carbohydrate-based terminal olefins through the “Uemura system” to hemiketals and α, β-unsaturated diketones. *Org. Lett.* **21**, 8145–8148 (2019).

8. C. Denner, M. Gintner, H. Kählig, W. Schmid, Indium-mediated C-allylation of melibiose. *Beilstein J. Org. Chem.* **15**, 2458–2464 (2019).

9. B. M. Trost, Designing a receptor for molecular recognition in a catalytic synthetic reaction: Allylic alkylation. *Acc. Chem. Res.* **29**, 355–364 (1996).

10. B. M.Trost, D. L. Van Vranken, Asymmetric transition metal-catalyzed allylic alkylations. *Chem. Rev.* **96**, 395–422 (1996).

11. B.M.Trost, M. R. Machacek, A. Aponick, Predicting the stereochemistry of diphenylphosphino benzoic acid (DPPBA)-based palladium-catalyzed asymmetric allylic alkylation reactions: A working model. *Acc. Chem. Res*. **39**, 747–760 (2006).

12. Z. Lu, S. Ma, Metal-catalyzed enantioselective allylation in asymmetric synthesis. *Angew. Chem. Int. Ed.* **47**, 258–297 (2008).

13. M. Diéguez, O. Pàmies, Biaryl phosphites: New efficient adaptative ligands for Pd-catalyzed asymmetric allylic substitution reactions. *Acc.Chem. Res*. **43**, 312–322 (2010).

14. J. D. Weaver, A. Recio III, A. J. Grenning, J. A. Tunge, Transition metal-catalyzed decarboxylative allylation and benzylation reactions. *Chem. Rev.* **111**, 1846–1913 (2011).

15. L. Yu, L. Lv, Z. Qiu, Z. Chen, Z. Tan, Y.-F. Liang, C.-J. Li, Palladium-catalyzed formal hydroalkylation of aryl-substituted alkynes with hydrazones. *Angew. Chem. Int. Ed*. **59**, 14009–14013 (2020).

16. L. Lv, D. Zhu, Z. Qiu, J. Li, C.-J. Li, Nickel-catalyzed regioselective hydrobenzylation of 1,3-dienes with hydrazones. *ACS Catal.* **9**, 9199–9205 (2019).

17. L. Lv, L. Yu, Z. Qiu, C.-J. Li, Switch in selectivity for formal hydroalkylation of 1,3-dienes and enynes with simple hydrazones. *Angew. Chem. Int. Ed.* **59**, 6466–6472 (2020).

18. D. Zhu, L. Lv, C.-C. Li, S. Ung, J. Gao, C.-J. Li, Umpolung of carbonyl groups as alkyl organometallic reagent surrogates for palladium-catalyzed allylic alkylation. *Angew.Chem. Int. Ed.* **57**, 16520–16524 (2018).

19. J. Yao, Z. Chen, L. Yu, L. Lv, D. Cao, C.-J. Li, Palladium-catalyzed hydroalkylation of methylenecyclopropanes with simple hydrazones. *Chem. Sci.* **11**, 10759–10763 (2020).

20. L. Lv, C.-J. Li, Palladium-catalyzed defluorinative alkylation of gem-difluorocyclopropanes: Switching regioselectivity via simple hydrazones. *Angew. Chem. Int. Ed.* **60**, 13098–13104 (2021).

21. M. G. Organ, S. Avola, I. Dubovyk, N. Hadei, E. A. B. Kantchev,C. J. O’Brien, C. Valente, A user-friendly, all-purpose Pd–NHC (NHC=N-heterocyclic carbene) precatalyst for the Negishi reaction: A step towards a universal cross-coupling catalyst. *Chem. A Eur. J.* 12, 4749–4755 (2006).

22. The supplementary crystallographic data for the structures of **3am** and **4am** are available free of charge from the Cambridge Crystallographic Data Centre under accession numbers CCDC-2112299 and 2104006, respectively.

23. R. W. W. Hooft, L. H. Straver, A. L. Spek, Determination of absolute structure using Bayesian statistics on Bijvoet differences. *J. Appl. Cryst.* **41**, 96–103 (2008).

24. D. Gao, G. A. O'Doherty, De novo asymmetric synthesis of anamarine and its analogues. *J. Org. Chem.* **70**, 9932–9939 (2005).

25. D. Gao, G. A. O'Doherty, Enantioselective synthesis of 10-*epi*-anamarine via an iterative dihydroxylation sequence. *Org. Lett.* **7**, 1069–1072 (2005).

26. S. Wang, B. Y. Cheng, M. Srsen, B. Konig, Umpolung difunctionalization of carbonyls via visible-light photoredox catalytic radical-carbanion relay. *J. Am. Chem. Soc.* **142**, 7524–7531 (2020).

27. Y. Xia, D. Qiu, J.-B. Wang, Transition-metal-catalyzed cross-couplings through carbene migratory insertion. *Chem. Rev.* **117**, 13810–13889 (2017).

28. M. Bender, H. Mouritsen, J. Christoffers, A robust synthesis of 7,8-didemethyl-8-hydroxy-5-deazariboflavin. *Beilstein J. Org. Chem.* **12**, 912–917 (2016).

29. N. R. Lees, L.-C. Han, M. J. Byrne, J. A. Davies, A. E. Parnell, P. E. J. Moreland, J. E. M. Stach, M. W. van der Kamp, C. L. Willis, P. R. Race, An esterase-like lyase catalyzes acetate elimination in spirotetronate/spirotetramate biosynthesis. *Angew. Chem. Int. Ed.* **58**, 2305–2309 (2019).

30. Y. V. Mahidhar, M. Rajesh, A. Chaudhuri, Spacer-arm modulated gene delivery efficacy of novel cationic glycolipids: Design, synthesis, and in vitro transfection biology. *J. Med. Chem.* **47**, 3938–3948 (2004).

31. A. Jakas, A. Visnjevac, I. Jeric, Multicomponent approach to homo- and hetero-multivalent glycomimetics bearing rare monosaccharides. *J. Org. Chem.* **85**, 3766–3787 (2020).

32. R. F. Brady, Cyclic acetals of ketoses. *Carbohydr. Res.* **15**, 35–40 (1970).

33. J. D. More, N. S. Finney, A simple and advantageous protocol for the oxidation of alcohols with *O*-iodoxybenzoic acid (IBX). *Org. Lett.* **4**, 3001–3003 (2002).

34. H. Wang, X. J. Dai, C. J. Li, Aldehydes as alkyl carbanion equivalents for additions to carbonyl compounds. *Nat. Chem.* **9**,374–378 (2017).

35. N. Chen, X. J. Dai, H. Wang, C. J. Li, Umpolung addition of aldehydes to aryl imines. *Angew. Chem. Int. Ed.* **56**, 6260–6263 (2017).

36. X.-J. Dai, H. Wang, C.-J. Li, Carbonyls as latent alkyl carbanions for conjugate additions. *Angew. Chem. Int. Ed.* **56**, 6302–6306 (2017).

37. T. Katsina, S. P. Sharma, R. Buccafusca, D. J. Quinn, T. S. Moody, S. Arseniyadis, Sequential palladium-catalyzed allylic alkylation/retro-Dieckmann fragmentation strategy for the synthesis of α-substituted acrylonitriles. *Org. Lett.* **21**, 9348–9352 (2019).

38. Y. Kawato, A. Kubota, H. Ono, H. Egami, Y. Hamashima, Enantioselective bromocyclization of allylic amides catalyzed by BINAP derivatives. *Org. Lett.* **17**, 1244–1247 (2015).

39. E. Comer, M. G. Organ, S. J. Hynes, Allylic ionization versus oxidative addition into vinyl C-X bonds by Pd with polyfunctional olefin templates. *J. Am. Chem. Soc.* **126**, 16087–16092 (2004).

40. K. Kiyokawa, S. Yahata, T. Kojima, S. Minakata, Hypervalent iodine(III)-mediated oxidative decarboxylation of β,γ-unsaturated carboxylic acids. *Org. Lett.* **16**, 4646–4649 (2014).

41. C. Thongsornkleeb, R. L. Danheiser, A practical method for the synthesis of 2-alkynylpropenals. *J. Org. Chem.* **70**, 2364–2367 (2005).

42. N. Marion, R. Gealageas, S. P. Nolan, [(NHC)$Au^{I}$]-catalyzed rearrangement of allylic acetates. *Org. Lett.* **9**, 2653–2656 (2007).

43. X. Li, G. Wang, Z. Zhang, N. Wu, Q. Yang, S. Huang, X. Wang, A concise and straightforward approach to total synthesis of (+)-Strictifolione and formal synthesis of Cryptofolione via a unified strategy. *Synth. Commun.* 49, 1031–1039 (2019).
